# Lewis Acid-Catalyzed Cascade Ring Expansion and Intramolecular Friedel–Crafts Type Cyclization of α‑Dithioacetyl Propargyl Alcohols: Access to *S*,*S*‑Heterocycle-Fused Benzofulvenes and 3‑Benzylidene-1-indanones

**DOI:** 10.1021/acs.joc.6c00685

**Published:** 2026-07-01

**Authors:** Yigit Efe Turhan, Mehmet Aytug Sinmaz, Bilal Yenidogan, Hamdiye Ece, Ikbal Cal, Hüseyin Furkan Ürker, Dondu Karademir, Rimanur Malikoglu, Melda Tayanc, Kerem Kaya, Baris Yucel

**Affiliations:** 52971 Istanbul Technical University, Science Faculty, Department of Chemistry, Maslak 34469, Istanbul, Türkiye

## Abstract

The Lewis acid-catalyzed reaction of α-dithioacetyl propargyl alcohols has been reported. This cascade reaction involves the expansion of the dithioacetal ring, followed by a Friedel–Crafts cyclization of the resulting vinylic carbocation intermediates, efficiently producing six-, seven-, and eight-membered *S*,*S*-heterocycle-fused benzofulvenes in good yields (up to 98%) within short reaction times (5–40 min). When performed in the presence of water, the same reaction sequence afforded 3-benzylidene-1-indanone derivatives as single *E* isomers, albeit in lower yields (19–57%)

## Graphical abstract

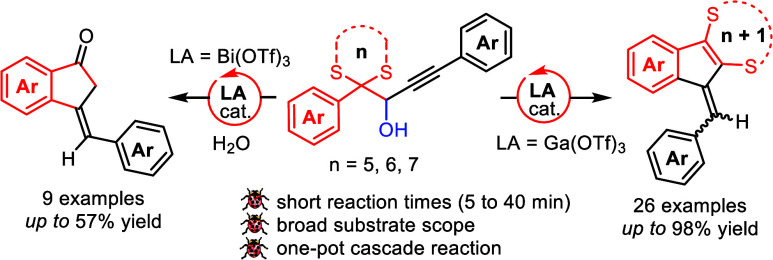

## Introduction

Sulfur-containing heterocycles, particularly seven-membered rings, have attracted more attention as key structural motifs in numerous U.S. FDA-approved drugs, such as diltiazem, bisulepin, quetiapine, and zaltoprofen.^1^ While the synthesis of smaller sulfur-containing aromatic heterocycles (e.g., thiophenes and thiazoles) is well-established,^2^ the construction of larger rings remains challenging due to unfavorable torsional strain and entropic constraints.^3^

1,3-Dithiolanes and 1,3-dithianes, five- and six-membered cyclic dithioacetals, are commonly used as protecting groups for carbonyl functionalities. The latter can be readily deprotonated and act as masked acyl anion equivalents in organic synthesis.^4^ Moreover, they serve as versatile building blocks for the synthesis of a broad range of sulfur-containing molecules and are particularly amenable to ring expansion reactions.^5^ Ring expansion strategies offer an effective approach for constructing kinetically and thermodynamically challenging medium-sized rings, providing advantages over conventional end-to-end cyclization or bimolecular cycloaddition methods.^6^

1,3-Dithiolane and 1,3-dithiane derivatives bearing a leaving group (e.g., Cl and Br) or a hydroxy group on the side chain have long been utilized to enable the synthesis of medium- and large-sized, generally unfunctionalized *S*,*S*-heterocycles.^7^ The ring expansion of these dithioacetals proceeds via bridged bicyclic sulfonium and thionium ion intermediates through a 1,2-sulfur rearrangement. Similarly, ring expansion reactions of 2-aryl- or 2-alkyl-substituted 1,3-dithiolanes and 1,3-dithianes in the presence of various electrophiles have been employed to afford structurally simple six- and seven-membered *S*,*S*-heterocycles via the formation of a thionium intermediate.^8^ However, more recently, efficient access to *S*,*S*-heterocycles of varying sizes and complexity has been achieved through the ring expansion of cyclic dithioacetals under radical-mediated^9^ and transition-metal-catalyzed reactions.^10^

Cascade or domino processes further enhance the synthetic potential of ring expansion reactions, enabling the one-pot construction of fused ring systems and macrocycles in a single operation.^11^ 1,3-Dithiolane and 1,3-dithiane derivatives have also been employed in cascade reactions to achieve *S*,*S*-heterocycle-fused ring systems. For example, Liu and co-workers demonstrated a Au-catalyzed cascade ring expansion–cyclization of 1,6-diynyl dithiolanes, affording benzo­[*a*]­fluorene derivatives (Scheme 1a).^12^ A Lewis acid-catalyzed (InCl_3_) cascade reaction of 2-aryl-1,3-dithiolanes and 2-aryl-1,3-dithianes with tertiary-substituted propargyl alcohols was reported by Muthusamy et al. This one-pot, two-component process effectively produced indenodithiepine and indenodithiocine derivatives (Scheme 1b).^13^ Moreover, very recently, Tang and co-workers reported that one-pot, two-step base-mediated cascade reactions of alkynyl-1,3-dithianes and α-diazoesters furnish various eight-membered *S*,*S*-heterocycles (Scheme 1c).^14^

**Scheme 1:**
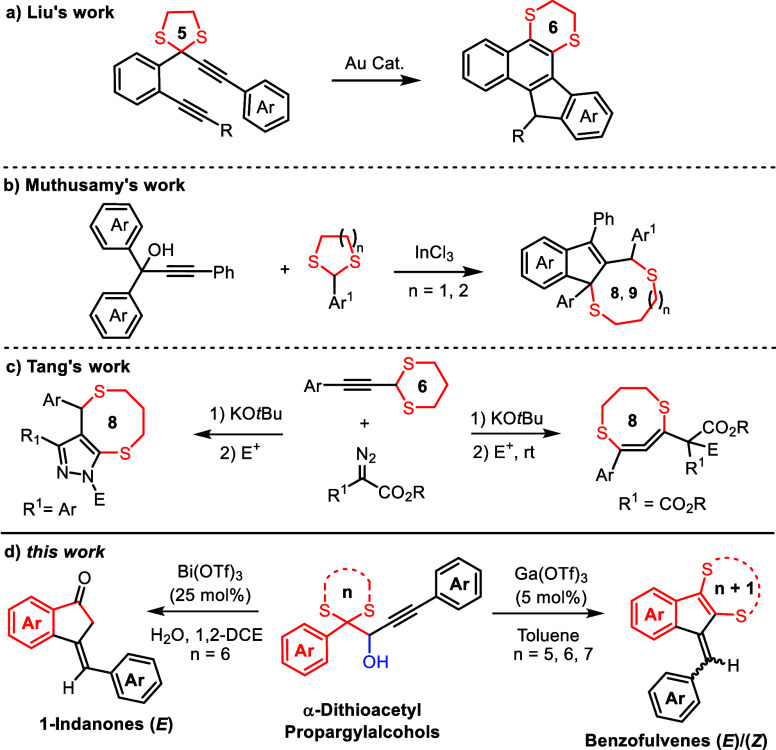
Ring Expansion Cascade Reactions of 1,3-Dithiolane and 1,3-Dithiane Derivatives

As part of our research program on the investigation of functional sulfur-containing heterocycles, herein, we develop a new strategy by performing a cascade ring expansion–Friedel–Crafts cyclization reaction of α-dithioacetyl propargyl alcohols under Lewis acid catalysis (5 mol % Ga­(OTf)_3_). This cascade reaction produces an *E*/*Z* mixture of six-, seven-, and eight-membered *S*,*S*-heterocycle-fused benzofulvenes in generally good yields (up to 98%) and short reaction times (Scheme 1d).

Although numerous synthetic approaches to benzofulvenes have been developed, the present method enables the straightforward synthesis of rarely reported tricyclic benzofulvenes.^15^ Benzofulvene derivatives are attractive targets because of their presence in marketed drugs such as sulindac and other bioactive compounds (Figure 1).^16^ The presented approach, combining essential features of *S*,*S*-heterocycles and benzofulvenes, could thus provide access to novel compounds with potential bioactivities and synthetic utility.

**Figure 1:**
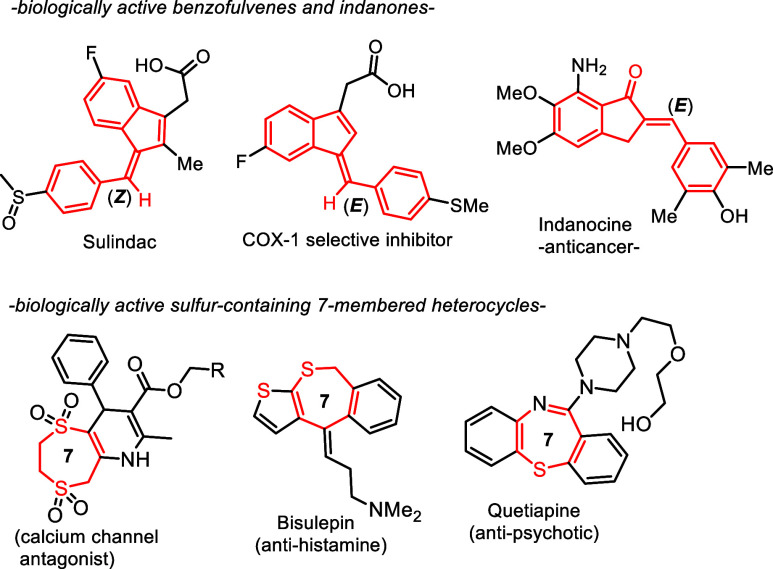
Selected examples of biologically active benzofulvenes, indanones, and sulfur-containing seven-membered heterocycles.

Finally, interestingly, conducting the cascade reaction in the presence of water affords 3-benzylidene-1-indanone derivatives as single *E* isomers, albeit in lower yields (Scheme 1d).^17^ Indanones are important scaffolds in pharmaceuticals, agrochemicals, and natural products and, together with benzofulvenes, serve as functional materials in organic electronics.^18^

## Results and Discussion

We selected propargyl alcohol **1a** as a model substrate and screened various Brønsted and Lewis acids, in both catalytic and stoichiometric amounts, to optimize the cascade conversion of **1a** into benzofulvene **2a** (Table 1; see Table S1 for the extended optimization data). The reactions were performed in different solvents at temperatures varying between 0 and 110 °C and for reaction times ranging from 2 min to 24 h. Additionally, we carried out some reactions in a one-pot, two-step fashion by gradually adding acids to the substrate solution or vice versa. We found that most of the acids tested could convert **1a** into benzofulvene **2a** in generally good yields, and that the *E*:*Z* ratio varied significantly depending on the reaction conditions (entries 1–10, Table 1). The reaction gave the best yields in toluene at 110 °C and worked well with catalytic amounts of TfOH and Lewis acids such as In­(OTf)_3_, Al­(OTf)_3_, and Ga­(OTf)_3_ in just 5 or 10 min (entries 6–9, respectively). Interestingly, Bi­(OTf)_3_ produced **2a** in lower yield (51%) together with 3-benzylidene-1-indanone derivative **2a′** (10%) (entry 10). Similarly, varying amounts of indanone formation were observed depending on the conditions and the acid loading in reactions of **1a** with TfOH and *p*-TsOH·H_2_O. Considering the very short reaction time (5 min) and the high yield of **2a**, the optimal reaction conditions were established as those described in entry 9.

**Table 1:**
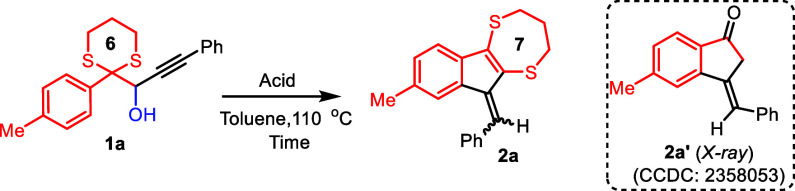
Optimization of the Reaction Conditions

entry	acid (equiv)	time	yield (%)	*E*:*Z* ratio of **2a**
1	H_2_SO_4_ (1.0)	15 min	37	8.8:1
2	CSA^a^ (1.0)	1 h	76	1.8:1
3^b^	MSA^c^ (1.0)	15 min	68	7.1:1
4	TFA^d^ (1.0)	15 min	–	–
5	*p*-TsOH·H_2_O (1.0)	10 min	63	18:1
6	TfOH^e^ (0.01)	5 min	77	2.2:1
7	In(OTf)_3_ (0.05)	5 min	74	1:1
8	Al(OTf)_3_ (0.05)	10 min	84	1.8:1
9	Ga(OTf)_3_ (0.05)	5 min	85	1.4:1
10^f^	Bi(OTf)_3_ (0.05)	5 min	51	4.5:1
11	Sc(OTf)_3_ (1.0)	1 h	40	1:1
12	Yt(OTf)_3_ (1.0)	2 h	–	–
13	Zn(OTf)_3_ (0.1)	3 h	–	–
14	Yb(OTf)_3_ (1.0)	4 h	trace	–
15	InCl_3_ (1.0)	15 min	–	–

a (±)-10-Camphorsulfonic acid.

b MSA was added dropwise to the solution of **1a**.

c Methanesulfonic acid.

d Trifluoroacetic acid.

e Triflic acid.

f 1-Indanone **2a′** (10%) was obtained.

Under the optimized conditions, the scope and limitations of the cascade reaction were examined using diversely substituted 1-(1,3-dithian-2-yl)­propargyl alcohols **1a**–**v** (Scheme 2). The propargyl alcohols were prepared in good yields via the reaction of 1,3-dithiane-2-carbaldehydes with lithium acetylides (see Table S2).

**Scheme 2:**
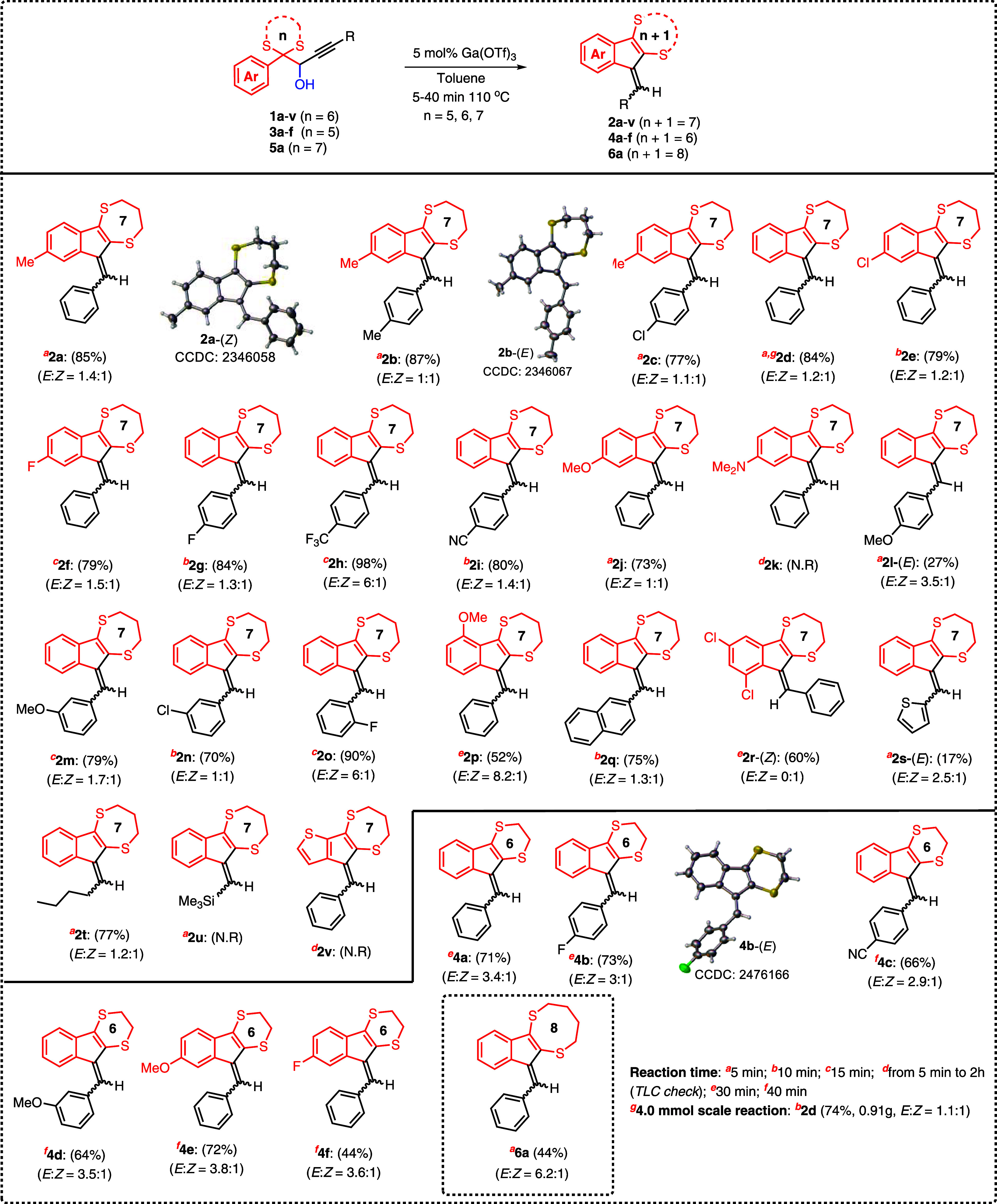
Substrate Scope for Six-, Seven-, and Eight-Membered *S*,*S*-Heterocycle-Fused Benzofulvenes

Except for **2l**, **2r**, and **2s**, all successful examples were isolated as a mixture of *E* and *Z* isomers generally in good yields (52–98%, average of 79% for 17 examples), regardless of the electronic properties or the positions of the substituents on the benzo core and exocyclic double bond. The *E*:*Z* ratios of the benzofulvenes were determined by ^1^H NMR spectroscopy, and with the exception of **2i** and **2t**, the isomers could be separated by column chromatography on silica gel.

We observed that the *E*:*Z* ratios of benzofulvenes change at different rates under slightly acidic conditions. Both pure *E* and *Z* isomers undergo isomerization even in CDCl_3_, which is known to contain residual acidic impurities. The *Z* isomers isomerize especially rapidly, making it difficult to obtain NMR spectra of the pure isomers unless the CDCl_3_ is neutralized prior to measurement.

For example, a pure sample of **2c**-(*Z*) in CDCl_3_ underwent isomerization over 3 days at room temperature, resulting in an *E*/Z mixture of **2c** (*E*:*Z* = 7.3:1). In contrast, pure **2c**-(*E*) was isomerized under the same conditions over 11 days, yielding an *E*/*Z* mixture (*E*:*Z* = 7.1:1). No further changes in the *E*:*Z* ratios were observed for either sample (Scheme 3 and Figures S1–S7).

**Scheme 3:**
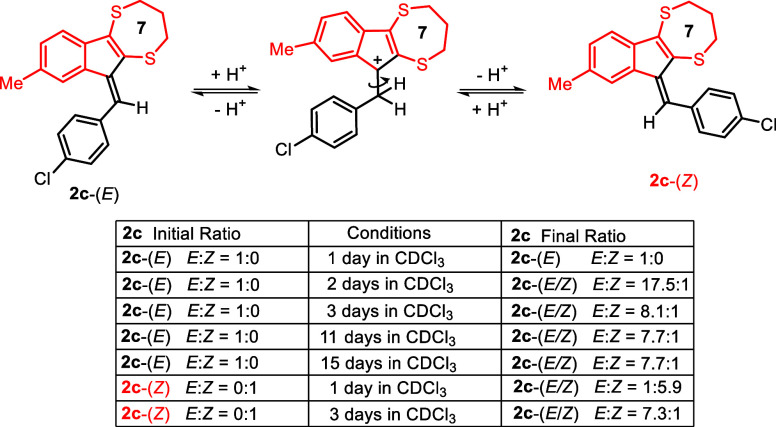
Observations of *E*/*Z* Isomerization of **2c**

These observations imply that *S*,*S*-heterocycle-fused benzofulvenes are rather sensitive to acidic conditions and are difficult to produce with high *E*/*Z* selectivity. The stereoisomerization can be rationalized by protonation of the exocyclic double bond of **2c**, forming a benzyl cation intermediate, which then undergoes sequential internal rotation and deprotonation (Scheme 3).

In two examples, **2a** and **2b**, the solid isomers were slowly crystallized from a hexane/CH_2_Cl_2_ mixture and then analyzed by X-ray crystallography. The isomers of benzofulvenes **2a** and **2b** were identified as *Z* and *E*, respectively. Based on this information, *E* and *Z* configurations of benzofulvenes **2a**–**s** were assigned according to the NMR spectral similarities to **2a**-(*E*) and **2b**-(*Z*). In particular, the *E* and *Z* isomers of benzofulvenes can be readily distinguished by the chemical shifts of the methylene protons adjacent to the two sulfur atoms in the ring.

In the *E* isomers, the methylene proton signals appear as two triplets at approximately 3.50 and 3.40 ppm. On the other hand, in the *Z* isomers, the methylene protons exhibit downfield and upfield shifts relative to those of the *E* isomers, appearing as two triplets at around 3.65  and 3.35 ppm, respectively. Notably, the reaction of 3,5-dichlorophenyl-substituted propargyl alcohol **1r** required 30 min to reach completion and produced solely the *Z* isomer of benzofulvene **2r**-(*Z*), featuring a sterically encumbered benzo core, in 60% yield.

The reaction of propargyl alcohols **1c**, **1e**–**i**, **1n**, and **1o** bearing electron-withdrawing groups (-F, -Cl, -CF_3_, and -CN) proceeded efficiently irrespective of their positions on either phenyl ring, furnishing benzofulvenes **2c**, **2e**–**i**, **2n**, and **2o**, respectively, in high yields (70–98%, average of 82% for eight examples) within 10–15 min, as judged by TLC.

To understand the effect of electron donation, cascade reactions of propargyl alcohols bearing -Me, -OMe, and -NMe_2_ substituents at various positions on the phenyl rings of the dithiane and propargyl moieties were examined. *p*- and *o*-OMe substitutions on the dithiane phenyl ring (**1j** and **1p**, respectively) afforded benzofulvenes **2j** and **2p** as *E*/*Z* mixtures in 73% and 52% yields, respectively, whereas the strongly electron-donating *p*-NMe_2_ substituent (**1k**) completely suppressed the formation of benzofulvene **2k**, even after prolonged reaction times (30 min and 2 h). *p*-OMe substitution on the propargyl phenyl ring (**1l**) led to benzofulvene **2l** as an *E*/*Z* mixture with unidentified byproducts, from which only the *E* isomer, **2l**-(*E*), was isolated in 27% yield. Moreover, *p*-Me and *m*-OMe substituents were well tolerated, delivering **2b** and **2m** in 87% and 79% yields, respectively.

In addition, naphthyl-, thienyl, and butyl-substituted propargyl alcohols **1q**, **1s**, and **1t**, respectively, were examined. Propargyl alcohols **1q** and **1t** reacted smoothly, affording **2q** and **2t** in 75% and 77% yields, respectively. The configurations of the inseparable *E*/*Z* isomers of **2t** were assigned by selective 1D NOE and 1D TOCSY NMR experiments (see Figures S8–S11). In contrast, propargyl alcohol **1s** afforded benzofulvene **2s** as an *E*/*Z* mixture accompanied by unidentified byproducts, from which only the *E* isomer could be isolated in 17% yield. Trimethylsilyl-substituted propargyl alcohol **1u** reacted rapidly under the standard conditions, reaching completion within 5 min; however, benzofulvene **2u** could not be isolated, likely due to desilylation followed by polymerization, as indicated by the crude NMR spectra. Finally, thienofulvene **2v** formation was examined to further assess the scope of the cascade reaction. The reaction of **1v** proceeded sluggishly, and after 2 h, the crude mixture consisted primarily of unreacted **1v**, along with trace amounts of thienofulvene **2v** and unidentified byproducts.

We next investigated the reaction of 1,3-dithiolanyl-substituted propargyl alcohols (**3a**–**f**) to furnish 1,4-dithiane-fused benzofulvenes (**4a**–**f**) (Scheme 2). Propargyl alcohols **3a**–**f** were obtained in good yields from 1,3-dithiolane-2-carbaldehydes and lithium acetylides (Table S2). Overall, the cascade reaction of propargyl alcohols **3a**–**f** required longer reaction times (30–40 min) to reach completion and produced benzofulvenes **4a**–**f**, respectively, as a mixture of *E*/*Z* isomers in 44–73% yields. The *E*:*Z* isomer ratios of the benzofulvenes were determined by ^1^H NMR spectroscopy. The isomers could not be readily separated by silica gel column chromatography; however, one isomer of benzofulvene **4b** was partially isolated and characterized by NMR spectroscopy. The isolated solid isomer of **4b** was slowly crystallized, and its configuration was unequivocally established as *E* by single-crystal X-ray structure analysis. Based on the ^1^H NMR spectrum of the pure *E* isomer of **4b**, the *E* and *Z* isomers of the other benzofulvenes were readily assigned. The methylene protons adjacent to the two sulfur atoms in the 1,4-dithiane ring were particularly useful for distinguishing the isomers, appearing as two closely spaced multiplets at around 3.40 and 3.35 ppm in the *E* isomers.

We also tested the reaction of propargyl alcohol **5a** containing a seven-membered dithiepane ring under the optimized conditions (5 min, 110 °C). The reaction afforded benzofulvene derivative **6a**, bearing an eight-membered *S*,*S*-heterocycle, in 44% yield as a mixture of *E* and *Z* isomers (Scheme 2). The isomers were separated by column chromatography on silica gel, and their configurations were identified by selective 1D NOE experiments. In the *E* isomer, irradiation of the double bond proton (δ = 7.67 ppm) clearly enhanced the signal of the methylene protons on the eight-membered ring at 3.02 ppm (see Figures S12 and S13). In contrast, in the *Z* isomer, saturation of the double bond proton (δ = 7.55 ppm) showed no NOE correlation with methylene protons on the ring (see Figure S14). We noticed that propargyl alcohol **5a** furnished exclusively the *E* isomer of **6a** when a longer reaction time (30 min) or a higher catalyst loading (10 mol %) was employed, albeit in a lower yield (36% or 38%, respectively).

To gain deeper insight and support the proposed reaction mechanism, we conducted control experiments, particularly regarding the nature of the active catalytic species. Considering the role of water in Lewis acid-catalyzed reactions, as well as the in situ formation of water as a byproduct of this cascade process, we investigated the reaction of propargyl alcohol **1a** in the presence of 2.0 equiv of H_2_O under standard conditions (5 mol % Ga­(OTf)_3_, 110 °C, 5 min, toluene). We observed that, in the presence of water, the reaction of **1a** did not reach completion within 5 min, and crude ^1^H NMR analysis revealed a mixture of **1a** along with products **2a** and **2a**-**IV** (Scheme 4a and Figure S15). Next, we repeated the reaction in the presence of 2.0 equiv of D_2_O with a reaction time of 20 min, as monitored by TLC. The reaction afforded an *E*/*Z* mixture of **2a-**H/D in 86% yield. The *E* isomer, exhibiting 30% deuterium incorporation at the methine position of the exocyclic double bond, was separated by column chromatography on silica gel (Scheme 4b and Figure S16). When propargyl alcohol **1b** was subjected to the same reaction conditions, **2b**-H/D was obtained in 71% yields as separable *E* and *Z* mixtures of isomers, **2b**-(*E*)-H/D and **2b**-(*Z*)-H/D, each exhibiting 30% deuteration at the methine group (Scheme 4c and Figures S18 and S20).

**Scheme 4:**
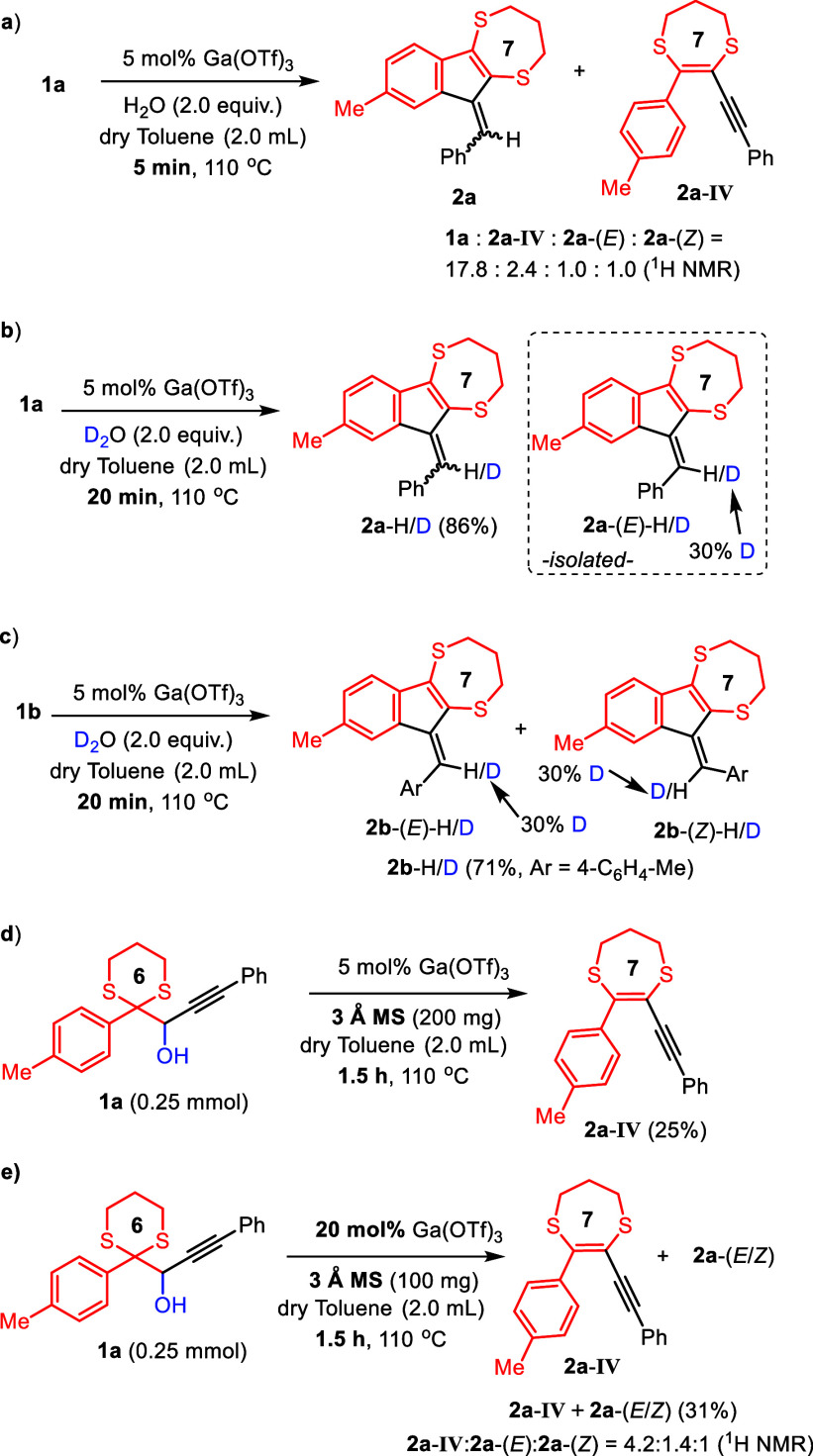
Control Experiments

Moreover, we examined the effect of water removal on the reaction outcome. The reactions of **1a** were carried out with different loadings of Ga­(OTf)_3_ (5 and 20 mol %) in the presence of freshly activated 3 Å molecular sieves. We determined that the extent of the reaction was significantly altered by the addition of 200 mg of molecular sieves under the standard conditions (5 mol % Ga­(OTf)_3_). The reaction of **1a** in toluene at 110 °C did not yield benzofulvene **2a**, even after a prolonged reaction time (1.5 h). Instead, ring expansion intermediate **2a**-**IV** and propargyl alcohol **1a** were isolated (Scheme 4d and Figures S22 and S23). When the reaction was carried out using 20 mol % Ga­(OTf)_3_ and 100 mg of 3 Å molecular sieves, a mixture of benzofulvene **2a** (*E*/*Z*) isomers and ring expansion intermediate **2a**-**IV** as a major component was obtained in 31% overall yield [4.2:1.4:1.0 **2a**-**IV**:**2a**-(*E*):**2a**-(*Z*), based on ^1^H NMR analysis] (Scheme 4e and Figure S24).

Altogether, these results strongly suggest that the formation of hydrated gallium species [Ga­(OTf)_3_·*n*H_2_O] is necessary for promoting the Friedel–Crafts type cyclization step leading to benzofulvene formation. Thus, the presence of water in the reaction medium is crucial. The hydration of Lewis acids such as Bi­(OTf)_3_ is energetically more favorable than their hydrolysis to triflic acid in the presence of water.^19^ The hydrated metal triflates with a strongly acidic hydrogen atom serve as the active catalytic species. This phenomenon is well-documented in the literature.^19a,20^

Based on the experimental results and the literature precedent, we propose a plausible mechanism for the formation of benzofulvenes via a Lewis acid-catalyzed cascade reaction of α-dithioacetyl propargyl alcohols (Scheme 5).

**Scheme 5:**
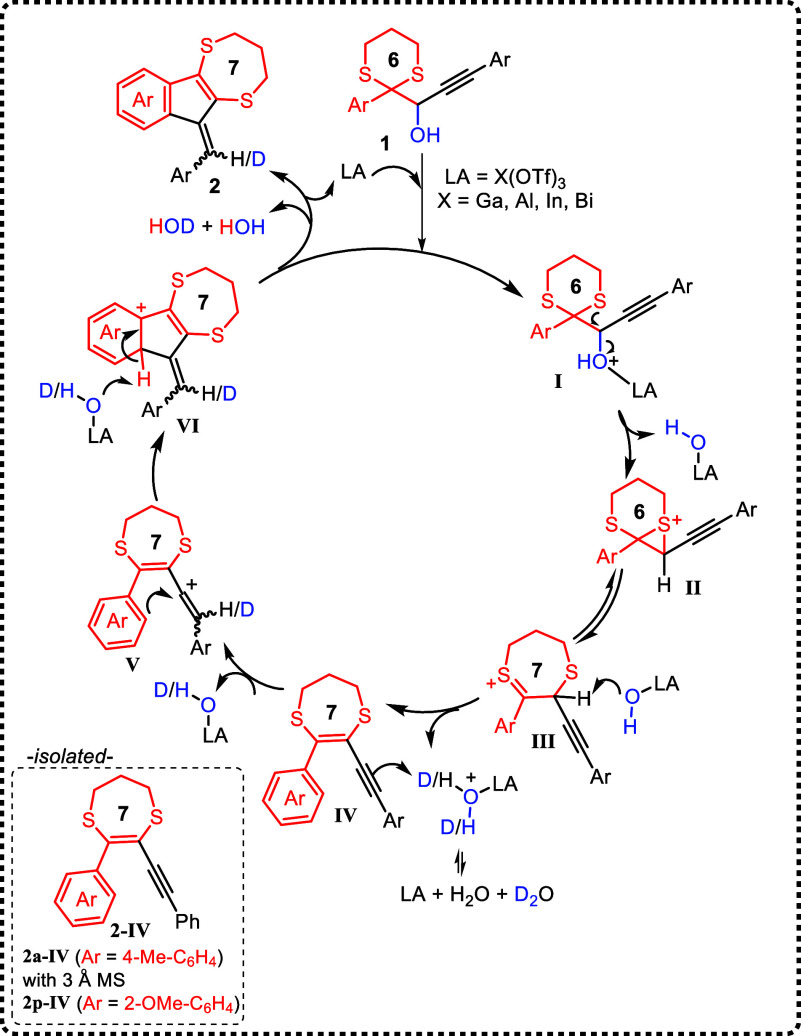
Catalytic Cycle

In the first step, propargyl alcohol **1** is activated by a Lewis acid to form intermediate **I**. Intramolecular nucleophilic attack of the dithiane sulfur atom affords bicyclic sulfonium ion **II**, which rapidly undergoes ring expansion via a 1,2-sulfur rearrangement to form thionium ion **III**. Deprotonation of **III** yields seven-membered *S*,*S*-ring intermediate **IV** and hydrated Lewis acid species as an active catalyst. Subsequent electrophilic activation of **IV** generates vinylic cation **V**, which undergoes a Friedel–Crafts cyclization to produce **VI**. Finally, elimination of a proton from **VI** yields benzofulvene **2**. Consistent with the proposed reaction pathway, intermediate **IV** (i.e., **2p-IV**) was isolated in 38% yield, together with benzofulvene **2p** (32% yield, 1.1:1 *E*:*Z*), when propargyl alcohol **1p** was heated at 110 °C in the presence of Ga­(OTf)_3_ for 5 min.

To add another dimension to this study, we aimed to access 3-benzylidene-1-indanone derivatives, previously observed during optimization studies of benzofulvenes (Table 1), in improved yields via a one-pot reaction of propargyl alcohols. We hypothesized that indanone **2a′** is a secondary product formed by *in situ* hydrolysis of benzofulvene **2a**. To verify this hypothesis, we first carried out test reactions using the *E* and *Z* isomers of **2a** in the presence of Bi­(OTf)_3_ with added H_2_O (Scheme 6a). Treatment of **2a**-(*E*) and **2a**-(*Z*) with Bi­(OTf)_3_ (25 mol %) and 2.0 equiv of H_2_O selectively afforded indanone **2a′** in the *E* configuration, in 39% and 74% yields, respectively. At the same time, both **2a**-(*E*) and **2a**-(*Z*) underwent isomerization, and benzofulvene **2a** was isolated as a mixture of *E* and *Z* isomers in 9% and 21% yields, respectively (Scheme 6a). We then briefly screened various H_2_O-containing conditions for propargyl alcohol **1a** to afford indanone **2a′** in a one-pot reaction (see Table S3). We found that increasing the amount of Bi­(OTf)_3_ or H_2_O did not improve the yield; the best result (57% yield for **2a′**) was obtained with Bi­(OTf)_3_ (25 mol %) and 2.0 equiv of H_2_O in 1,2-DCE in 10 min (as judged by TLC) at 110 °C (Scheme 6b). In the reaction mixture, trace amounts of **2a**-(*E*) were detected as an additional identifiable product, based on the crude ^1^H NMR spectrum. Accordingly, variously substituted propargyl alcohols **1b**–**e**, **1g**, **1h**, **1o**, and **1q** were converted into the corresponding indanones in generally moderate yields (33–50%), with the exception of *o*-fluoro-substituted **2o′** obtained in only 19% yield (Scheme 6b). Under these conditions, small amounts of the corresponding benzofulvenes were also formed but could not be isolated in pure form. Notably, 1,3-dithiolanyl-substituted propargyl alcohol **3a** failed to produce indanone **2d′** under the same conditions, even after extended reaction times (up to 2 h).

**Scheme 6:**
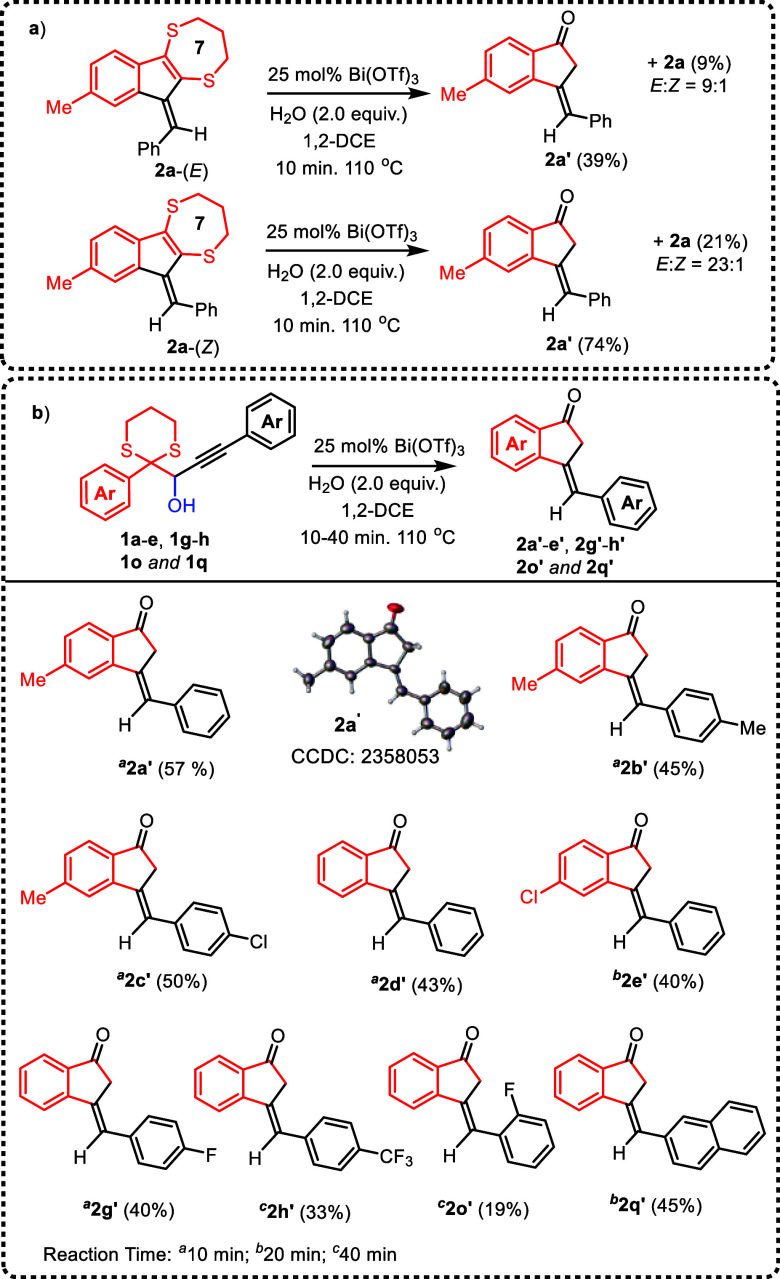
Synthesis of 3-Benzylidene-1-indanones

## Conclusion

In conclusion, we have developed an efficient approach for the synthesis of medium-sized *S*,*S*-heterocycle-fused benzofulvenes from readily accessible α-dithioacetyl propargyl alcohols. These reactions generally proceeded in high yields, displayed a broad substrate scope, and required short reaction times. The Lewis acid-catalyzed cascade reaction involves the expansion of the dithioacetal ring, followed by an intramolecular Friedel–Crafts type cyclization of vinyl carbocation intermediates. Furthermore, by slightly modifying the reaction conditions and introducing water, this strategy was successfully extended to the synthesis of 3-benzylidene-1-indanones.

## Experimental Section

### General Information

All reagents were used as purchased from commercial suppliers without further purification unless otherwise indicated. Air- and moisture-sensitive solutions were handled under nitrogen and transferred via syringe. Tetrahydrofuran (THF) was freshly distilled from sodium/benzophenone ketyl. Toluene was dried over sodium metal stored over activated molecular sieves (3 or 4 Å). 1,2-Dichloroethane was distilled over phosphorus pentoxide and stored over activated molecular sieves (3 or 4 Å). Molecular sieves were predried at 300 °C for 24 h immediately before use. Solvents for column chromatography, ethyl acetate and hexanes, were distilled in a rotary evaporator. TLC was performed with Merck TLC Silicagel60 F_254_ plates, and detection was achieved under UV light at 254 nm. Chromatographic separations were performed with Merck Silica 60 (200–400 or 70–230 mesh). NMR spectra were recorded with Varian Inova 500 (500 MHz for ^1^H and 126 MHz for ^13^C NMR) instruments. Chemical shifts (δ) are given in parts per million relative to residual peaks of deuterated solvents, and coupling constants (*J*) are given in hertz. The following abbreviations are used to describe spin multiplicities in ^1^H NMR spectra: s, singlet; bs, broad singlet; d, doublet; t, triplet; q, quartet; dd, doublet of doublets; m, multiplets. Multiplicities in ^13^C NMR spectra were determined by APT (attached proton test) measurements. High-resolution mass spectra (HRMS) were recorded on Waters Synapt Q-TOF-MS and Thermo Scientific Q Exactive Hybrid Quadrupole-Orbitrap MS spectrometers and Agilant 6530 Accurate-Mass Q-TOF LC/MS spectrometers.

### General Procedure for the Synthesis of Propargyl Alcohols (**1a**–**v** and **5a**)

To a solution of acetylene (3.0 equiv) in THF cooled to −78 °C was added dropwise *n*-BuLi (2.5 M in hexanes, 3.2 equiv) under a nitrogen atmosphere. The solution was allowed to stir for 30 min before being transferred via cannula into a solution of 2-aryl-1,3-dithiane-2-carbaldehyde (1.0 equiv) [for **5a**, 2-phenyl-1,3-dithiepane-2-carbaldehyde (1.0 equiv)] in THF under a nitrogen atmosphere cooled to −78 °C. The resulting mixture was allowed to stir for 3 h at −78 °C. The reaction was quenched with a 10% NH_4_Cl (20 mL) solution, and the mixture extracted with CH_2_Cl_2_ (2 × 100 mL). The combined organic layers were dried with anhydrous Na_2_SO_4_ and filtered. To the solution was added silica gel (2 g), and the solvent was removed in a rotatory evaporator. The residue was subjected to column chromatography on silica gel using a mixture of hexanes and ethyl acetate as the eluent to yield propargyl alcohols **1a**–**v** and **5a**.

### General Procedure for the Synthesis of Propargyl Alcohols (**3a**–**f**)

To a solution of acetylene (1.5 equiv) in THF cooled to −78 °C was added dropwise *n*-BuLi (2.5 M in hexanes, 1.0 equiv) under a nitrogen atmosphere. The solution was allowed to stir for 30 min before being transferred via cannula into a solution of 2-aryl-1,3-dithiolane-2-carbaldehyde (1.0 equiv) in THF under a nitrogen atmosphere cooled to −78 °C. The resulting mixture was allowed to stir for 3 h at −78 °C. The reaction was quenched with a 10% NH_4_Cl (20 mL) solution, and the mixture extracted with CH_2_Cl_2_ (2 × 100 mL). The combined organic layers were dried with anhydrous Na_2_SO_4_ and filtered. To the solution was added silica gel (2 g), and the solvent was removed in a rotatory evaporator. The residue was subjected to column chromatography on silica gel using a mixture of hexanes and ethyl acetate as the eluent to yield propargyl alcohols **3a–f**.

### General Procedure for the Synthesis of Six-, Seven-, and Eight-Membered *S*,*S*-Heterocycle-Fused Benzofulvenes

An oven-dried 15 mL screw-cap reaction vial equipped with a stirring bar was charged with an α-dithioacetyl propargyl alcohol derivative (1.0 equiv), and then the vial was placed in a glovebox. The reaction vial was charged with Ga­(OTf)_3_ (0.05 equiv, 5 mol %) and anhydrous toluene. The vial was tightly closed, wrapped with a strip of Parafilm, and removed from the glovebox. After the reaction mixture had been stirred for the given time at 110 °C in a preheated oil bath, the vial was cooled to room temperature. The reaction mixture was placed in a 50 mL flask, and the solvent was removed in a rotatory evaporator. The remaining residue was dissolved in CH_2_Cl_2_ and mixed with silica gel (about 0.5–1.0 g). After the evaporation of CH_2_Cl_2_, the remaining silica gel was directly loaded onto a column and purified by flash chromatography on silica gel using a mixture of hexanes and ethyl acetate as the eluent to yield products **2a**–**t**, **4a–f**, and **6a**.

### General Procedure for the Synthesis of 3-Benzylidene-1-indanones (**2a′–e′**, **2g′**, **2h′**, **2o′**, and **2q′**)

An oven-dried 15 mL screw-cap reaction vial equipped with a stirring bar was charged with an α-dithioacetyl propargyl alcohol derivative (1.0 equiv) and water (2.0 equiv) by a micropipette, and then the vial was placed in a glovebox. The reaction vial was charged with Bi­(OTf)_3_ (0.25 equiv, 25 mol %) and anhydrous 1,2-dichloroethane. The vial was tightly closed, wrapped with a strip of Parafilm, and removed from the glovebox. After the reaction mixture had been stirred for the given time at 110 °C in a preheated oil bath, the vial was cooled to room temperature. The reaction mixture was placed in a 50 mL flask, and the solvent was removed in a rotatory evaporator. The remaining residue was dissolved in CH_2_Cl_2_ and mixed with silica gel (about 0.5–1.0 g). After the evaporation of CH_2_Cl_2_, the remaining silica gel was directly loaded onto a column and purified by flash chromatography on silica gel using a mixture of hexanes and ethyl acetate as the eluent to yield products **2a′–e′**, **2g′**, **2h′**, **2o′**, and **2q′**.

## Supplementary Material















## Data Availability

The data underlying this study are available in the published article and its .

## Notes

FAIR data, including the primary NMR FID files, for compounds ^1^H NMR **1a**–**v**, **2a**-(*E*)–**2h**-(*E*), **2j**-(*E*)–**2q**-(*E*), **2s**-(*E*), **2a**-(*Z*)–**2h**-(*Z*)**, 2j**-(*Z*)–**2r**-(*Z*), **2i**-(*E*/*Z*), **2t**-(*E*/*Z*), **3a**–**f**, **4a**-(*E*), **4b**-(*E*), **4e**-(*E*), **4f**-(*E*), **4a**–**d**-(*E*/*Z*), **4e**-(*Z*), **4f**-(*Z*), **5a**, **6a**-(*E*), **6a**-(*Z*), **2a′–e′**, **2g′**, **2h′**, **2o′**, **2q′**, **2a-IV**, **2p-IV**, **2a**-(*E*)-H/D, **2b**-(*E*)-H/D, **2b**-(*Z*)-H/D, **S1d**, **S1g**, **S1h**, **S3b**, and **S3c** (ZIP)
FAIR data, including the primary NMR FID files, for compounds^13^C NMR **1a**–**v**, **2a**-(*E*)–**2h**-(*E*), **2j**-(*E*)–**2q**-(*E*), **2s**-(*E*), **2a**-(*Z*)–**2h**-(*Z*)**, 2j**-(*Z*)–**2r**-(*Z*), **2i**-(*E*/*Z*), **2t**-(*E*/*Z*), **3a**–**f**, **4a**-(*E*), **4b**-(*E*), **4e**-(*E*), **4f**-(*E*), **4a**–**d**-(*E*/*Z*), **4e**-(*Z*), **4f**-(*Z*), **5a**, **6a**-(*E*), **6a**-(*Z*), **2a′–e′**, **2g′**, **2h′**, **2o′**, **2q′**, **2a-IV**, **2p-IV**, **2a**-(*E*)-H/D, **2b**-(*E*)-H/D, **2b**-(*Z*)-H/D, **S1d**, **S1g**, **S1h**, **S3b**, and **S3c** (ZIP
FAIR data, including the primary NMR FID files, for compounds ^13^C APT NMR **1a**–**v** (ZIP)
FAIR data, including the primary NMR FID files, for compounds ^13^C APT NMR **2a**-(*E*)–**2h**-(*E*), **2j**-(*E*)–**2q**-(*E*), **2s**-(*E*), **2a**-(*Z*)–**2h**-(*Z*)**, 2j**-(*Z*)–**2r**-(*Z*), **2i**-(*E*/*Z*), **2t**-(*E*/*Z*), **2a-IV**, **2p-IV** (ZIP)
FAIR data, including the primary NMR FID files, for compounds ^13^C APT NMR **2a′–e′**, **2g′**, **2h′**, **2o′**, **2q′** (ZIP)
FAIR data, including the primary NMR FID files, for compounds ^13^C APT NMR **3a** – **f** , ****4a**-**(*E*), **4b**-(*E*), **4e** -(*E*), **4f** -(*E*), **4a** –****d**-**(*E*/*Z*), **4e** -(*Z*), **4f** -(*Z*), **5a** , **6a** -(*E*)**,**6a****-(*Z*), **S1d**, **S1g**, **S1h**, **S3b**, and **S3c** (ZIP)
All experimental procedures, NMR spectral data, HRMS spectral data, X-ray crystallographic analysis of compounds **2a**-(*Z*), **2b**-(*E*), **4b**-(*E*), and **2a′**, and copies of ^1^H and ^13^C­{^1^H} NMR spectra of **1a**–**v**, **2a**-(*E*)–**2h**-(*E*), **2j**-(*E*)–**2q**-(*E*), **2s**-(*E*), **2a**-(*Z*)–**2h**-(*Z*)**, 2j**-(*Z*)–**2r**-(*Z*), **2i**-(*E*/*Z*), **2t**-(*E*/*Z*), **3a**–**f**, **4a**-(*E*), **4b**-(*E*), **4e**-(*E*), **4f**-(*E*), **4a**–**d**-(*E*/*Z*), **4e**-(*Z*), **4f**-(*Z*), **5a**, **6a**-(*E*), **6a**-(*Z*), **2a′–e′**, **2g′**, **2h′**, **2o′**, **2q′**, **2a-IV**, **2p-IV**, **2a**-(*E*)-H/D, **2b**-(*E*)-H/D, **2b**-(*Z*)-H/D, **S1d**, **S1g**, **S1h**, **S3b**, and **S3c** (PDF)

## Notes

This paper is dedicated to the memory of Professor Armin de Meijere, the “Lord of the Small Rings”, who was an amazing chemist, educator, and mentor.
