# Supplementary material for: Lewis Acid-Catalyzed Cascade Ring Expansion and Intramolecular Friedel–Crafts Type Cyclization of α‑Dithioacetyl Propargyl Alcohols: Access to S,S‑Heterocycle-Fused Benzofulvenes and 3‑Benzylidene-1-indanones
Source: J Org Chem. 2026 Jul 1;91(27):9529–37. doi: 10.1021/acs.joc.6c00685 (PMC13366585; doi:10.1021/acs.joc.6c00685)

SUPPORTING INFORMATION

Lewis Acid-Catalyzed Cascade Ring Expansion and Intramolecular Friedel-Crafts Type Cyclization of α-Dithioacetyl Propargyl Alcohols: Access to *S,S*-Heterocycle-Fused Benzofulvenes and 3-Benzylidene-1-indanones

Yigit Efe Turhan, Mehmet Aytug Sinmaz, Bilal Yenidogan, Hamdiye Ece, Ikbal Cal, Hüseyin Furkan Ürker, Dondu Karademir, Rimanur Malikoglu, Melda Tayanc, Kerem Kaya, Baris Yucel*

Istanbul Technical University, Science Faculty, Department of Chemistry, Maslak 34469, Istanbul, Türkiye.

TABLE OF CONTENTS

1. General Methods ……………………………………………………….……….…………….S2

2. Optimization of reaction conditions for Lewis acid-catalyzed ring expansion and intramolecular Friedel-Crafts type cyclization of α-dithioacetyl propargyl alcohols ………………….……….......S3

3. Table S1. Optimization of reaction conditions for product **2a**…....……….….……...……..…..S3

4. Synthesis of 1,3-dithiane-2-carbaldehyde derivatives (**S1a–i**) …………... …………….………S5

5. Synthesis of 2-aryl-1,3-dithiolane-2-carbaldehydes (**S3a-c**) and 2-phenyl-1,3-dithiepane-2-carbaldehyde (**S4a**) ……………………………………………………………………….……...S8

6. Synthesis of propargyl alcohols (**1a-v**, **3a**–**f** and **5a**)…….…………………………..……….S12

7. Table S2. Substrate scope of propargyl alcohols (**1a-v, 3a**–**f** and **5a**)………….……………...…..S12

8. Synthesis of for 6-, 7-, and 8-membered *S,S*-heterocycle-fused benzofulvenes ……...…...…… S27

9. Large Scale Preparation of Benzofulvene **2d**……………………………………………………...S47

10. Optimization of reaction conditions for indanone **2a'**…..…………..…………………….......S48

11. Table S3. Optimization of reaction conditions for indanone **2a'**…………………….……….....… S48

12. Synthesis of 3-benzylidene-1-indanones ...………...……………………….………..…...S48

13. Observations of *E*/*Z* isomerization of benzofulvene **2c**……………………………….……….S55

14. 1D TOCSY NMR experiments of benzofulvene **2t**-(*E*/*Z*)………………………………...…S59

15. 1D NOE experiments of benzofulvene **2t**-(*E*/*Z*)…………………………………………….....S60

16. 1D NOE experiments for benzofulvane **6a**-(*E*/*Z*)……………………………………………...S61

17. Control experiments………………………………………………………………………...S63

18. X-ray Crystallography Data for compound **2a**-(*Z*), **2b**-(*E*), **4b**-(*E*) and **2a'** …..………………...S72

19. References ...………………...………………………………………….…………………..S77

20. NMR Spectra of compounds………...………………………………….…………………..S78

1. General Methods. All reagents were used as purchased from commercial suppliers without further purification unless otherwise indicated. Air- and moisture-sensitive solutions were handled under nitrogen and transferred via syringe. Tetrahydrofuran (THF) was freshly distilled from sodium/benzophenone ketyl. Toluene was dried over sodium metal stored over activated molecular sieves (3 or 4 Å). 1,2-Dichloroethane were distilled over phosphorus pentoxide and stored over activated molecular sieves (3 or 4 Å). Molecular sieves were pre-dried at 300 ^{o}C for 24 h immediately before use. Solvents for column chromatography, ethyl acetate and hexanes were distilled in a rotary evaporator. TLC was performed with Merck TLC Silicagel60 F_{254} plates and detection was under UV light at 254 nm. Chromatographic separations were performed with Merck Silica 60 (200–400 or 70–230 mesh). NMR spectra were recorded with a Varian Inova 500 (500 MHz for ^{1}H and 125 MHz for ^{13}C NMR) instruments. Chemical shifts δ were given in ppm relative to residual peaks of deuterated solvents and coupling constants, *J*, were given in Hertz. The following abbreviations are used to describe spin multiplicities in ^{1}H NMR spectra: s = singlet; bs = broad singlet; d = doublet; t = triplet; q = quartet; dd = doublet of doublets; m = multiplets. Multiplicities in ^{13}C NMR spectra were determined by APT (Attached Proton Test) measurements. High resolution mass spectra (HRMS) were obtained on a Waters Synapt Q-TOF-MS and Thermo Scientific™ Q Exactive Hybrid Quadrupole-Orbitrap MS spectrometers and Agilant 6530 Accurate-Mass Q-TOF LC/MS spectrometers.

2. Optimization of reaction conditions for Lewis acid-catalyzed ring expansion and intramolecular Friedel-Crafts type cyclization of α-dithioacetyl propargyl alcohols

Table S1. Optimization of reaction conditions for product 2a

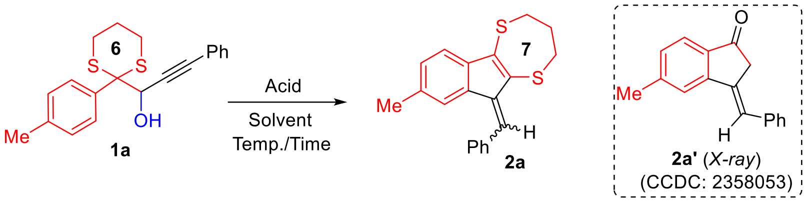

Entry	Acid (equiv.)	Temp./Time	Solvent	Yield (%)	E/Z
1[a]	H_2SO_4 (1.0)	100 °C / 15 min	Toluene	37%	8.8:1
2[b]	H_2SO_4 (1.0)	110 °C / 15 min	Toluene	47%	9.3:1
3[c]	H_2SO_4 (1.0)	110 °C / 15 min	Toluene	51%	10.6:1
4	H_2SO_4 (0.3)	110 °C / 24 h	Toluene	*Trace*	-
5[a]	CSA[d] (1.0)	110 °C / 1 h	Toluene	76%	1.8:1
6[c]	CSA (1.0)	110 °C / 1 h	Toluene	72%	9.0:1
7	MSA[e] (1.0)	110 °C / 15 min	Toluene	-	-
8[a,c]	MSA (1.0)	110 °C / 15 min	Toluene	68%	7.1:1
9[a]	TFA[f] (1.0)	110 °C / 15 min	Toluene	-	-
10[c]	TfOH[g] (1.0)	110 °C / 10 min	Toluene	-	-
11[c]	TfOH (1.0)	24 °C / 18 h.	Toluene	-	-
12[h]	TfOH (0.05)	110 °C / 5 min	Toluene	57%	8:1
13[a,h]	TfOH (0.01)	110 °C / 5 min	Toluene	77%	2.2:1
14[h]	TfOH (0.05)	80 °C / 15 min	Toluene	73%	1.4:1
15[h]	TfOH (0.025)	80 °C / 15 min	Toluene	74%	1.4:1
16[a,h]	*p*-TsOH.H_2O[i] (1.0)	110 °C / 10 min	Toluene	63%	18:1
17[j,h]	*p*-TsOH.H_2O (0.5)	110 °C / 10 min	Toluene	58%	16:1
18[j,h]	*p*-TsOH.H_2O (0.25)	110 °C / 10 min	Toluene	71%	3:1
19	$In(OTf)_3$ (1.0)	100 °C / 15 min	Toluene	46%	14:1
20[b]	$In(OTf)_3$ (1.0)	110 °C / 10 min	Toluene	68%	10:1
21	$In(OTf)_3$ (1.0)	0 °C / 24 h	DME[k]	*Trace*	-
22	$In(OTf)_3$ (1.0)	0 °C / 24 h	Toluene	*Trace*	-
23[a]	$In(OTf)_3$ (0.05)	110 °C / 5 min	Toluene	74%	1:1
24[h]	$Bi(OTf)_3$ (0.5)	110 °C / 15 min	Toluene	29%	4:1
25[h]	$Bi(OTf)_3$ (0.1)	110 °C / 10 min	Toluene	49%	12:1
26[a,l]	$Bi(OTf)_3$ (0.05)	110 °C / 5 min	Toluene	51%	4.5:1
27[a]	$Al(OTf)_3$ (0.05)	110 °C / 10 min	Toluene	84%	1.8:1
28	$Al(OTf)_3$ (0.05)	110 °C / 10 min	1,2-DCE[m]	75%	2:1
29	$Al(OTf)_3$ (0.1)	110 °C / 10 min	Toluene	84%	2.2:1
30	$Al(OTf)_3$ (0.1)	110 °C / 15 min	Dioxane	79%	1:1.4
31	$Al(OTf)_3$ (0.1)	110 °C / 15 min	THF	75%	1:1.4
32	$Al(OTf)_3$ (0.2)	110 °C / 5 min	Toluene	81%	1:1
33	$Al(OTf)_3$ (0.2)	110 °C / 10 min	Toluene	76%	1.8:1
34	$Al(OTf)_3$ (0.25)	110 °C / 5 min	Toluene	80%	1.2:1
35	$Al(OTf)_3$ (0.25)	50 °C / 3 h	1,2-DCE	95%	1.5:1

36	$Ga(OTf)_3$ (0.1)	110 °C / 5 min	Toluene	85%	1.5:1
**37[a]**	**$Ga(OTf)_3$ (0.05)**	**110 °C / 5 min**	**Toluene**	**85%**	**1.4:1**
38	$Ga(OTf)_3$ (0.05)	110 °C / 30 min	Toluene	81%	9.5/1
39	$Ga(OTf)_3$ (0.05)	110 °C / 2 min	1,2-DCE	55%	5.5:1
40	$Ga(OTf)_3$ (0.05)	80 °C / 5 min	1,2-DCE	65%	1.3:1
41	$Ga(OTf)_3$ (0.05)	80 °C / 30 min	Toluene	79%	1:1
42	$Yt(OTf)_3$ (1.0)	110 °C / 2 h	Toluene	-	-
43	$Sc(OTf)_3$ (1.0)	110 °C / 1 h	Toluene	40%	1:1
44[a]	$Sc(OTf)_3$ (1.0)	70 °C / 24 h	Toluene	35%	1:1.2
45[a]	$Zn(OTf)_3$ (0.1)	110 °C / 3 h	Toluene	-	-
46[a]	$Yb(OTf)_3$ (1.0)	110 °C / 4 h	Toluene	-	-
47[a]	$InCl_3$ (1.0)	110 °C / 15 min	Toluene	-	-

[a]Data is given in the manuscript; see Table 1, [b]**1a** was added dropwise to the solution of acid, [c]Acid solution was added dropwise to the solution of **1a**, [d](+/-)10-Camphorsulfonic acid. [e]Methanesulfonic acid, [f]Trifluoroasetic acid, [g] Triflic acid, [h]The formation of 1-indanone **2a'** was observed, [i]*p*-Toluenesulfonic acid, [j]*p*-TsOH.H_2O was crystallized before use, [k]1,2-Dimethoxyethane, [l]1-indanone **2a'** was isolated (10% yield), [m]1,2-Dichloroethane.

3. Synthesis of 1,3-dithiane-2-carbaldehyde derivatives (S1a–i)

1,3-Dithiane-2-carbaldehydes **S1a-d** and **S1g–i** were synthesized according to the slightly modified published procedure (General Procedure B) given below. For NMR data and spectra of compounds **S1a-c** and **S1e–f** and **S1i** please see: ref. S1 (*Eur. J. Org. Chem.* 2021, 29, 4107-4124) and ref. S2 (*Org. Lett.* **2023**, *25*, 4028−4032).

Scheme S1. Synthesis of 1,3-dithiane-2-carbaldehyde derivatives (**S1a–i**)

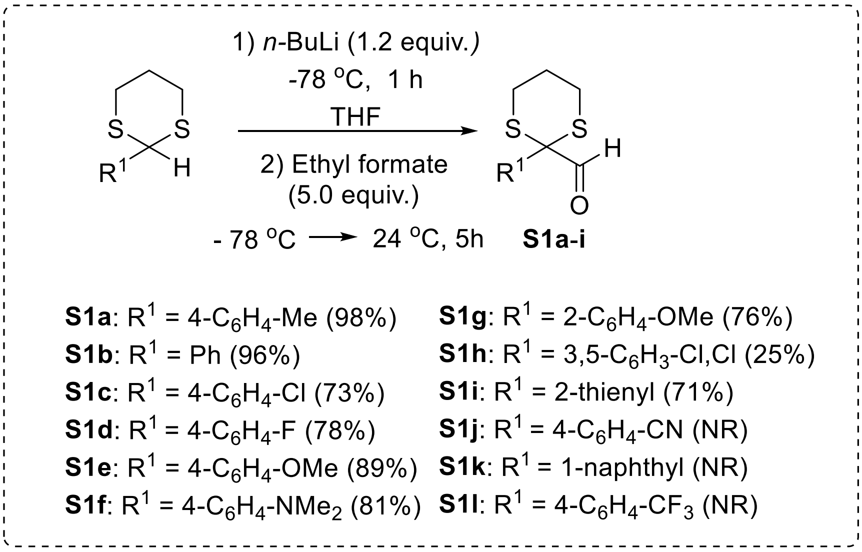

3.1. General Procedure A: To a solution of 1,3-dithiane derivative (1.0 equiv.) in THF, cooled to −78 °C under nitrogen atmosphere, was added dropwise a solution of *n*-butyllithium (1.2 equiv.) The solution was stirred for 1h at −78 °C and then ethyl formate (5.0 equiv.) was added dropwise at this temperature. The resulting mixture was slowly allowed to warm to room temperature and stirred for 5 h. The reaction mixture was quenched with water and extracted with ether. The organic phase was dried over $MgSO_4$, filtrated and the solvent was removed in a rotatory evaporator. The remaining residue was loaded onto a silica gel column and purified by flash chromatography.

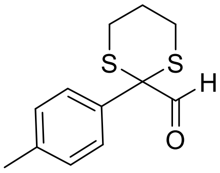

2-(*p*-Tolyl)-1,3-dithiane-2-carbaldehyde (**S1a**)**:** According to general procedure A, to a solution of 2-(*p*-tolyl)-1,3-dithiane (7.84 mmol, 1.65 g) in THF (55 mL) at −78 °C under nitrogen, *n*-BuLi (9.5 mL of a 2.5 M of hexane solution, 3.8 mmol) was added. The solution was stirred for 1h at −78 °C and then ethyl formate (42.8 mmol, 3.5 mL) was added. The resulting mixture was stirred for 5 h and the crude material was obtained as described in the general procedure and purified by flash chromatography on silica gel using hexanes as eluent to yield the product **S1a** (1.82 g, 98%) as a white solid, mp: 92-93 °C.

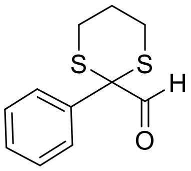

2-Phenyl-1,3-dithiane-2-carbaldehyde (**S1b**)**:** According to general procedure A, to a solution of 2-phenyl-1,3-dithiane (20.1 mmol, 4.0 g) in THF (60 mL) at −78 °C under nitrogen, *n*-BuLi (9.8 mL of a 2.5 M of hexane solution, 24.4 mmol) was added. The solution was stirred for 1h at −78 °C and then ethyl formate (0.1 mol, 8.0 mL) was added. The resulting mixture was stirred for 5 h and the crude material was obtained as described in the general procedure and purified by flash chromatography on silica gel using hexanes as eluent to yield the product **S1b** (4.4 g, 96%) as a white solid, mp: 91-92 °C.

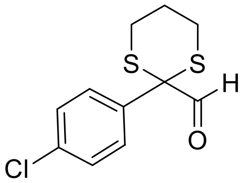

2-(4-Chlorophenyl)-1,3-dithiane-2-carbaldehyde (**S1c**)**:** According to general procedure A, to a solution of 2-(4-chlorophenyl)-1,3-dithiane (3.48 mmol, 0.8 g) in THF (20 mL) at −78 °C under nitrogen, *n*-BuLi (1.6 mL of a 2.5 M of hexane solution, 4.0 mmol) was added. The solution was stirred for 1h at −78 °C and then ethyl formate (18.6 mmol, 1.5 mL) was added. The resulting mixture was stirred for 5 h and the crude material was obtained as described in the general procedure and purified by flash chromatography on silica gel using 20:1 hexanes/ethyl acetate as eluent to yield the product **S1c** (0.66 g, 73%) as a white solid, mp: 96-97 °C.

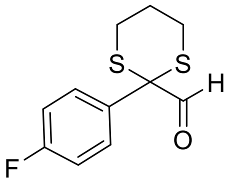

2-(4-Fluorophenyl)-1,3-dithiane-2-carbaldehyde (**S1d**)**:** According to general procedure A, to a solution of 2-(4-fluorophenyl)-1,3-dithiane (3.96 mmol, 0.85 g) in THF (22 mL) at −78 ºC under nitrogen, *n*-BuLi (1.9 mL of a 2.5 M of hexane solution, 4.75 mmol) was added. The solution was stirred for 1h at −78 ºC and then ethyl formate (19.8 mmol, 1.6 mL) was added. The resulting mixture was stirred for 5 h and the crude material was obtained as described in the general procedure and purified by flash chromatography on silica gel using 60:1 hexanes/ethyl acetate as eluent to yield the product **S1d** (0.75 g, 78%) as a white solid, mp: 86-87 ºC. ^{1}H NMR (500 MHz, $CDCl_3$): δ 9.12 (s, 1H), 7.48–7.45 (m, 2H), 7.12–7.09 (m, 2H), 3.18–3.12 (m, 2H), 2.72 (dt, J = 3.7, 14.4 Hz, 2H), 2.15–2.12 (m, 1H), 1.91–1.83 (m, 1H) ppm; ^{13}C{^{1}H} NMR (126 MHz, $CDCl_3$) δ 186.3, 163.0 (d, $^1J_{CF}$ = 250 Hz), 131.0 (d, $^4J_{CF}$ = 3.3 Hz), 129.6 (d, $^3J_{CF}$ = 8.5 Hz), 116.3 (d, $^2J_{CF}$ = 21.9 Hz), 60.1, 28.0, 23.5 ppm.

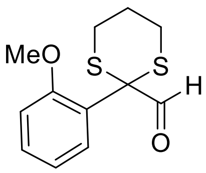

2-(2-Methoxyphenyl)-1,3-dithiane-2-carbaldehyde (**S1g**)**:** According to general procedure A, to a solution of 2-(2-methoxyphenyl)-1,3-dithiane (3.67 mmol, 0.83 g) in THF (20 mL) at −78 ºC under nitrogen, *n*-BuLi (1.8 mL of a 2.5 M of hexane solution, 4.5 mmol) was added. The solution was stirred for 1h at −78 ºC and then ethyl formate (17.33 mmol, 1.4 mL) was added. The resulting mixture was stirred for 5 h and the crude material was obtained as described in the general procedure and purified by flash chromatography on silica gel using 10:1 hexanes/ethyl acetate as eluent to yield the product **S1g** (0.71 g, 76%) as a white solid, mp: 101-102 ºC. ^{1}H NMR (500 MHz, $CDCl_3$) δ 8.96 (s, 1H), 7.83 (dd, J = 1.8, 7.8 Hz, 1H), 7.35 (dt, J = 1.7, 7.2 Hz, 1H), 7.06 (t, J = 8.6 Hz, 1H), 6.92 (d, J = 8.2 Hz, 1H), 3.85 (s, 3H), 3.35–3.29 (m, 2H), 2.66 (dt, J = 3.4, 14.4 Hz, 2H), 2.17–2.12 (m, 1H), 1.94–1.83 (m, 1H) ppm; ^{13}C{^{1}H} NMR (126 MHz, $CDCl_3$) δ 183.6, 156.3, 130.8, 129.7, 124.5, 121.9, 111.9, 58.3, 56.1, 27.8, 23.8 ppm; HRMS [TOF MS ES+]: m/z $[M + H]^+$ calcd. for $C_{12}H_{15}O_2S_2$ 255.0508, found 255.0509 (0.39 ppm).

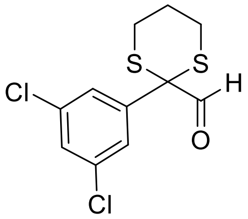

2-(3,5-Dichlorophenyl)-1,3-dithiane-2-carbaldehyde (**S1h**): According to general procedure A, to a solution of 2-(3,5-dichlorophenyl)-1,3-dithiane (3.96 mmol, 1.06 g) in THF (28 mL) at −78 °C under nitrogen, *n*-BuLi (1.9 mL of a 2.5 M of hexane solution, 4.75 mmol) was added. The solution was stirred for 1h at −78 °C and then ethyl formate (19.8 mmol, 1.6 mL) was added. The resulting mixture was stirred for 5 h and the crude material was obtained as described in the general procedure and purified by flash chromatography on silica gel using 80:1 hexanes/ethyl acetate as eluent to yield the product **S1h** (0.3 g, 25%) as a white solid, mp: 119-120 °C. ^{1}H NMR (500 MHz, $CDCl_3$): δ 9.11 (s, 1H), 7.32 (bs, 2H), 7.35 (bs, 1H), 3.14–3.09 (m, 2H), 2.71 (dt, *J* = 4.0, 14.4 Hz, 2H), 2.15–2.10 (m, 1H), 1.89–1.81 (m, 1H) ppm; ^{13}C{^{1}H} NMR (126 MHz, $CDCl_3$) δ 185.2, 138.5, 135.6, 129.5, 126.3, 60.6, 27.7, 23.3 ppm.

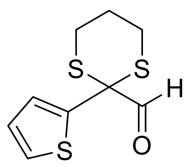

2-(Thiophen-2-yl)-1,3-dithiane-2-carbaldehyde (**S1i**): According to general procedure A, to a solution of 2-(thiophen-2-yl)-1,3-dithiane (4.15 mmol, 0.84 g) in THF (22 mL) at −78 °C under nitrogen, *n*-BuLi (2.0 mL of a 2.5 M of hexane solution, 4.98 mmol) was added. The solution was stirred for 1h at −78 °C and then ethyl formate (20.8 mmol, 1.7 mL) was added. The resulting mixture was stirred for 5 h and the crude material was obtained as described in the general procedure and purified by flash chromatography on silica gel using 10:1 hexanes/ethyl acetate as eluent to yield the product **S1i** (0.68 g, 71%) as a pale yellow solid mp: 63-64 °C.

4. Synthesis of 2-aryl-1,3-dithiolane-2-carbaldehydes (S3a-c) and 2-phenyl-1,3-dithiepane-2-carbaldehyde (S4a)

2-Phenyl-1,3-dithiolane-2-carbaldehyde (**S3a-c**) and 2-phenyl-1,3-dithiepane-2-carbaldehyde (**S4a**) were synthesized following the procedures given below. Alcohols **S3a-c-OH** and **S4a-OH** were prepared according to the published procedure, please see: ref. S3 (*J. Am. Chem. Soc*. **2018**, *140*, 2629−2642). For procedures and spectroscopic data of **S3a** and **S4a**, please see: ref. S2 (*Org. Lett.* **2023**, *25*, 4028－4032).

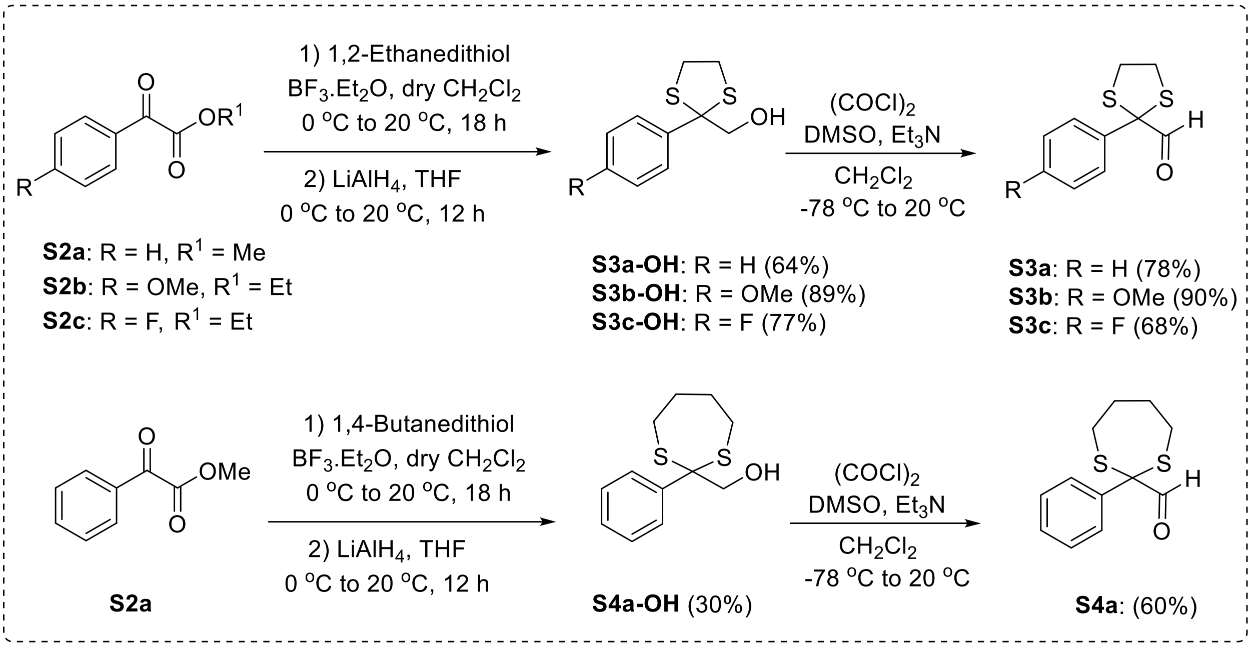

Scheme S2. Synthesis of 2-aryl-1,3-dithiolane-2-carbaldehydes (**S3b** and **S3c**)

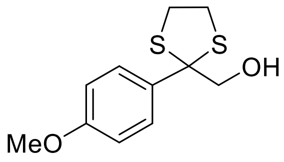

(2-(4-Methoxyphenyl)-1,3-dithiolan-2-yl)methanol (S3b-OH): To a stirred solution of ethyl 4-methoxybenzoylformate (**S2b**) (3.0 g, 14.4 mmol) and 1,2-ethanedithiol (1.2 mL, 14.4 mmol) in CH_2Cl_2 (30 mL) was added dropwise $BF_3 \cdot Et_2O$ (3.5 mL, 28.8 mmol) at 0 °C. Then, the resulting mixture was allowed to warm to room temperature and stirred overnight. The reaction mixture was quenched with water and extracted with CH_2Cl_2. The organic phase was dried over Na_2SO_4, filtrated and the solvent was removed in a rotatory evaporator. The remaining solid residue was dissolved in THF (15 mL) and the solution was dropwise added to a stirred suspension of $LiAlH_4$ (0.8 g, 21.2 mmol) in THF (20 mL) at 0 °C. The resulting mixture was allowed to warm to room temperature and stirred for 12 h. Then, the reaction mixture quenched with saturated aqueous solution of sodium tartrate tetrahydrate (Rochelle salt) (50 mL) and extracted with CH_2Cl_2. The organic layer was washed with 50 mL of water, dried over anhydrous Na_2SO_4, filtered, and the solvent was removed under reduced pressure. The remaining residue was purified by flash chromatography on silica gel using using 4:1 hexanes/ethyl acetate as eluent to yield the product **S3b-OH** (3.03 g, 89%) as a white solid mp: 82-83 °C. ^1H NMR (500 MHz, $CDCl_3$): δ 7.60 (d, J = 8.9 Hz, 2H), 6.85 (d, J = 8.9 Hz, 2H), 3.95 (s, 2H), 3.79 (s, 3H), 3.44–3.36 (m, 2H), 2.36 (bs, 1H) ppm. $^{13}C\{^1H\}$ NMR (126 MHz, $CDCl_3$) δ 158.9, 133.1, 128.9, 113.4, 74.5, 70.4, 55.2, 39.8 ppm.

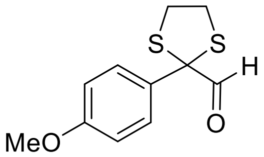

2-(4-Methoxyphenyl)-1,3-dithiolane-2-carbaldehyde (S3b): Dimethyl sulfoxide (4.0 mL, 56.1 mmol, 5.0 equiv.) was added slowly to oxalyl chloride (2.0 mL, 22.4 mmol, 2.0 equiv.) in CH_2Cl_2 (60 mL) at -70 °C. After 20 min, the alcohol **S3b-OH** (2.72 g, 11.2 mmol, 1.0 equiv.) in CH_2Cl_2 (20 mL) was added slowly. After 1 h, Et_3N (7.8 mL, 56.1 mmol, 5.0 equiv.) was added and the mixture was allowed to warm to room temperature. The reaction mixture was quenched with water and extracted with CH_2Cl_2. The organic phase was dried over Na_2SO_4, filtrated and the solvent was removed in a rotatory evaporator. The remaining residue was loaded onto a silica gel column and purified by flash chromatography on silica gel using 10:1 hexanes/ethyl acetate as eluent to yield the product **S3b** (2.42 g, 90%) as a colorless oil. ^{1}H NMR (500 MHz, $CDCl_3$): δ 9.24 (s, 1H), 7.40 (d, J = 8.8 Hz, 2H), 6.89 (d, J = 8.8 Hz, 2H), 3.79 (s, 3H), 3.48–3.42 (m, 2H), 3.35–3.29 (m, 2H) ppm. ^{13}C{^{1}H} NMR (126 MHz, $CDCl_3$): δ 186.3, 159.7, 129.3, 126.2, 114.0, 76.3, 55.2, 39.8 ppm. HRMS [TOF MS ES+]: m/z $[M + H]^+$ calcd. for $C_{11}H_{13}O_2S_2$ 241.0350, found 241.0351 (0.41 ppm).

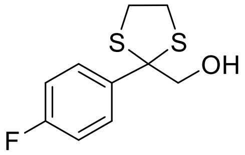

(2-(4-Fluorophenyl)-1,3-dithiolan-2-yl)methanol (S3c-OH): To a stirred solution of ethyl 4-fluorobenzoylformate (**S2c**) (1.6 g, 8.1 mmol) and 1,2-ethanedithiol (0.8 mL, 9.5 mmol) in CH_2Cl_2 (27 mL) was added dropwise $BF_3{\cdot}OEt$ (2.0 mL, 16.2 mmol) at 0 °C. Then, the resulting mixture was allowed to warm to room temperature and stirred overnight. The reaction mixture was quenched with water and extracted with CH_2Cl_2. The organic phase was dried over Na_2SO_4, filtrated and the solvent was removed in a rotatory evaporator. The remaining solid residue was dissolved in THF (10 mL) and the solution was dropwise added to a stirred suspension of $LiAlH_4$ (0.41 g, 10.8 mmol) in THF (30 mL) at 0 °C. The resulting mixture was allowed to warm to room temperature and stirred for 12 h. Then, the reaction mixture quenched with saturated aqueous solution of sodium tartrate tetrahydrate (Rochelle salt) (50 mL) and extracted with CH_2Cl_2. The organic layer was washed with 50 mL of water, dried over anhydrous Na_2SO_4, filtered, and the solvent was removed under reduced pressure. The remaining residue was purified by flash chromatography on silica gel using hexanes as eluent to yield the product **S3c-OH** (1.61 g, 77%) as a white solid, mp: 73-74 ºC. ^{1}H NMR (500 MHz, $CDCl_3$): δ 7.66–7.63 (m, 2H), 7.01–

6.97 (m, 2H), 3.92 (s, 2H), 3.41–3.32 (m, 4H), 2.81 (bs, 1H) ppm. $^{13}C\{^1H\}$ NMR (126 MHz, $CDCl_3$) δ 161.8 (d, $^1J_{CF}$ = 247 Hz), 137.1 (d, $^4J_{CF}$ = 3.3 Hz), 129.4 (d, $^3J_{CF}$ = 8.0 Hz), 114.6 (d, $^2J_{CF}$ = 21.3 Hz), 73.9, 70.2, 39.7 ppm.

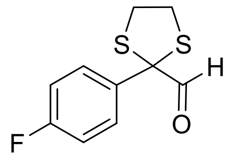

2-(4-Fluorophenyl)-1,3-dithiolane-2-carbaldehyde (S3c): Dimethyl sulfoxide (5.9 mL, 82.7 mmol, 5.0 equiv.) was added slowly to oxalyl chloride (2.84 mL, 33.1 mmol, 2.0 equiv.) in CH_2Cl_2 (80 mL) at -70 °C. After 20 min, the alcohol **S3c-OH** (3.81 g, 16.5 mmol, 1.0 equiv.) in CH_2Cl_2 (40 mL) was added slowly. After 1 h, Et_3N (11.5 mL, 82.7 mmol, 5.0 equiv.) was added and the mixture was allowed to warm to room temperature. The reaction mixture was quenched with water and extracted with CH_2Cl_2. The organic phase was dried over Na_2SO_4, filtrated and the solvent was removed in a rotatory evaporator. The remaining residue was loaded onto a silica gel column and purified by flash chromatography on silica gel using 10:1 hexanes/ethyl acetate as eluent to yield the product **S3c** (2.56 g, 68%) as a yellowish oil. ^1H NMR (500 MHz, $CDCl_3$): δ 9.25 (s, 1H), 7.49–7.46 (m, 2H), 7.06 (t, J = 8.5 Hz, 2H), 3.50–3.44 (m, 2H), 3.40–3.34 (m, 2H) ppm. $^{13}C\{^1H\}$ NMR (126 MHz, $CDCl_3$) δ 186.1, 162.6 (d, $^1J_{CF}$ = 249 Hz), 130.8 (d, $^4J_{CF}$ = 3.3 Hz), 130.0 (d, $^3J_{CF}$ = 8.3 Hz), 115.5 (d, $^2J_{CF}$ = 21.8 Hz), 75.8, 40.2 ppm. HRMS [TOF MS ES+]: m/z $[M + H]^+$ calcd. for $C_{10}H_{10}FOS_2$ 229.0152, found 229.0152 (0 ppm).

5. Synthesis of propargyl alcohols (1a-v, 3a–f and 5a)

Propargyl alcohols (**1a-v, 3a–f** and **5a**) were synthesized according to the General Procedure B given below.

Table S2. Substrate scope of propargyl alcohols (**1a-v, 3a–f** and **5a**)

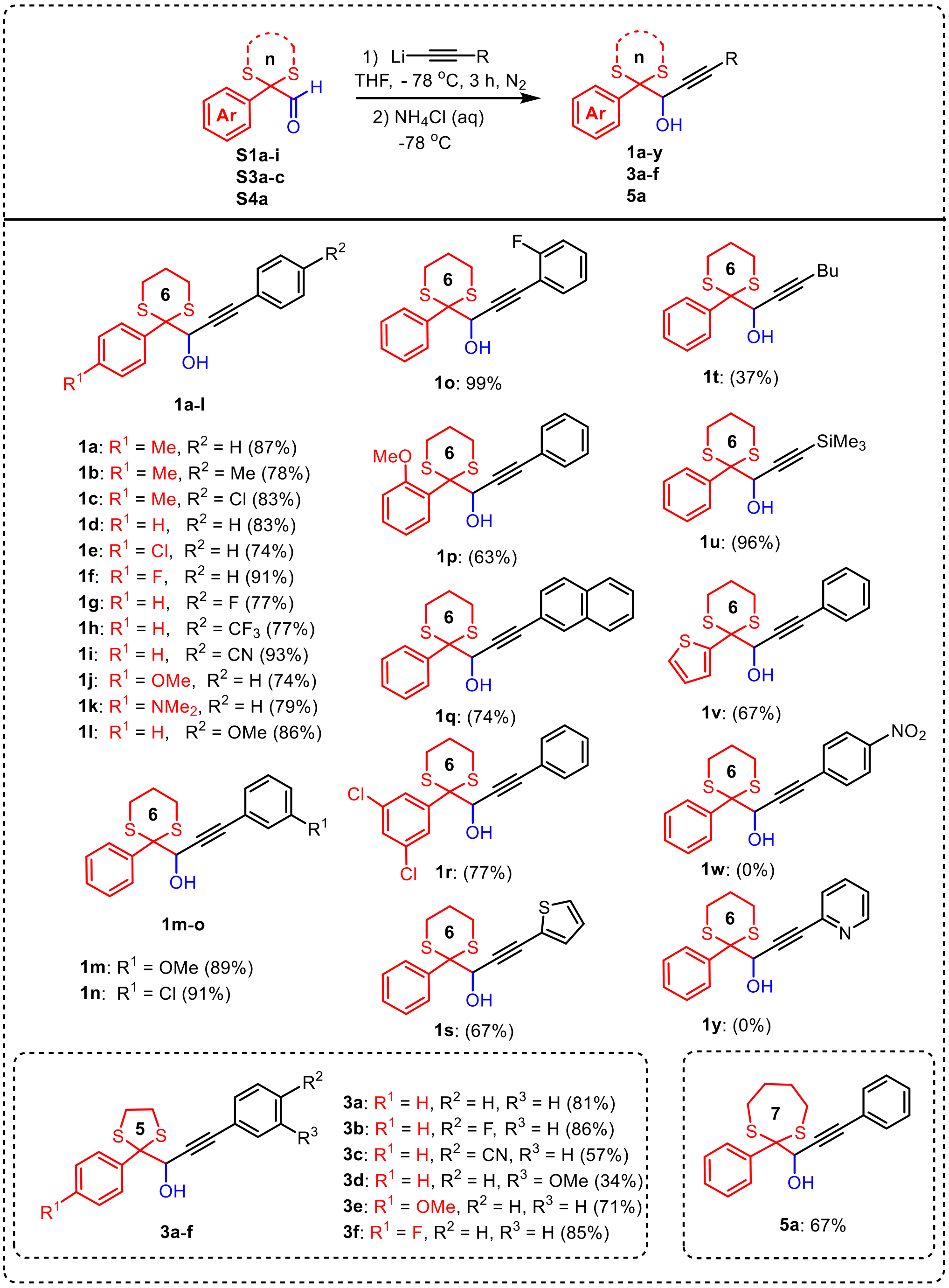

5.1. General Procedure B: To a solution of acetylene (3.0 equiv) in THF cooled to −78 °C was added dropwise *n*-BuLi (2.5 M in hexanes, 3.2 equiv) under nitrogen atmosphere. The solution was allowed to stir for 30 min before being transferred via cannula into a solution of 1,3-dithioacetyl-2-carbaldehyde (1.0 equiv) in THF under nitrogen atmosphere cooled to −78 °C. The resulting mixture was allowed to stir for 3h at −78 °C. The mixture was quenched with 10% NH_4Cl (20 mL) solution and extracted with CH_2Cl_2 (2 × 100 mL). The combined organic layers were dried with anhydrous Na_2SO_4 and filtered. To the solution silica gel (2 g) was added and the solvent was removed in a rotatory evaporator. The residue was subjected to column chromatography on silica gel using a mixture of hexanes and ethyl acetate as eluent to yield propargyl alcohols **1a-y**.

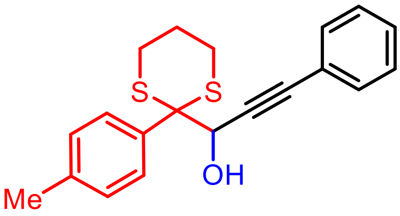

3-Phenyl-1-(2-(p-tolyl)-1,3-dithian-2-yl)prop-2-yn-1-ol (1a): The reaction was performed following the general procedure B with phenylacetylene (0.64 g, 6.3 mmol) in THF (15 mL), *n*-BuLi (6.7 mmol, 2.68 mL of a 2.5 M of hexane solution) and **S1a**, (0.5 g, 2.10 mmol) in THF (10 mL). After the resulting mixture was allowed to stir for 3h at −78 °C, the crude material was obtained as described in the general procedure and subjected to column chromatography on silica gel using 10:1 hexanes/ethyl acetate as eluent to yield **1a** (0.62 g, 87%, white solid, mp: 103-104 °C). ^{1}H-NMR (500 MHz, $CDCl_3$) δ 7.98 (d, *J* = 8.0 Hz, 2H), 7.39−7.37 (m, 2H), 7.31−7.27 (m, 3H), 7.24 (d, *J* = 8.0 Hz, 2H), 4.92 (s, 1H), 2.86−2.80 (m, 2H), 2.75−2.69 (m, 2H), 2.38 (s, 3H), 1.99−1.93 (m, 2H) ppm. $^{13}C\{^1H\}$ NMR (126 MHz, $CDCl_3$, APT) δ 137.7, 134.1, 131.7, 130.2, 129.1, 128.5, 128.2, 122.2, 87.9, 85.8, 70.5, 64.4, 27.4, 27.3, 24.7, 21.0 ppm. HRMS [TOF MS ES+]: m/z $[M + H]^+$ calcd. for $C_{20}H_{21}OS_2$ 341.1028, found 341.1034 (1.76 ppm).

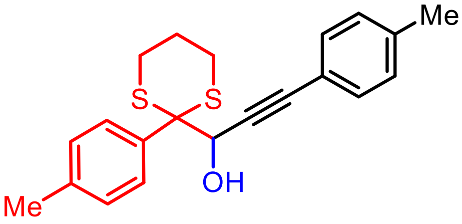

3-(*p*-Tolyl)-1-(2-(*p*-tolyl)-1,3-dithian-2-yl)prop-2-yn-1-ol (1b): The reaction was performed following the general procedure B with 1-ethynyl-4-methylbenzene (1.46 g, 12.6 mmol) in THF (30 mL), *n*-BuLi (13.4 mmol, 5.4 mL of a 2.5 M of hexane solution) and **S1a**, (1.0 g, 4.20 mmol) in THF (30 mL). After the resulting mixture was allowed to stir for 3h at −78 °C, the crude material was obtained as described in the general

procedure and subjected to column chromatography on silica gel using 10:1 hexanes/ethyl acetate as eluent to yield **1b** (1.16 g, 78%, white solid, mp: 102-103 °C). ^{1}H-NMR (500 MHz, $CDCl_3$) δ 7.98 (d, *J* = 8.0 Hz, 2H), 7.27 (d, *J* = 8.0 Hz, 2H), 7.23 (d, *J* = 8.0 Hz, 2H), 7.09 (d, *J* = 8.0 Hz, 2H), 4.91 (s, 1H), 2.86−2.79 (m, 2H), 2.74−2.68 (m, 2H), 2.37 (s, 3H), 2.33 (s, 3H), 1.98−1.92 (m, 2H) ppm. ^{13}C{^{1}H} NMR (126 MHz, $CDCl_3$, APT) δ 138.7, 137.7, 134.1, 131.6, 130.3, 129.1, 128.9, 119.2, 88.1, 85.1, 70.6, 64.4, 27.4, 27.3, 24.7, 21.5, 21.0 ppm. HRMS [TOF MS ES+]: m/z $[M + H]^+$ calcd. for $C_{21}H_{22}OS_2$ 355.1185, found 355.1190 (1.42 ppm).

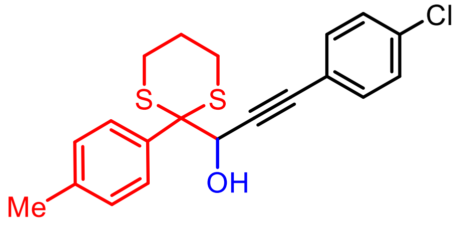

3-(4-chlorophenyl)-1-(2-(p-tolyl)-1,3-dithian-2-yl)prop-2-yn-1-ol (1c): The reaction was performed following the general procedure B with 1-ethynyl-4-chlorobenzene (1.72 g, 12.6 mmol) in THF (25 mL), *n*-BuLi (13.4 mmol, 5.4 mL of a 2.5 M of hexane solution) and **S1a**, (1.0 g, 4.20 mmol) in THF (30 mL). After the resulting mixture was allowed to stir for 3h at −78 °C, the crude material was obtained as described in the general procedure and subjected to column chromatography on silica gel using 10:1 hexanes/ethyl acetate as eluent to yield **1c** (1.30 g, 83%, white solid, mp: 111-112 °C). ^{1}H-NMR (500 MHz, $CDCl_3$) δ 7.98−7.96 (m, 2H), 7.31−7.23 (m, 6H), 4.91 (s, 1H), 2.86−2.80 (m, 2H), 2.75−2.70 (m, 2H), 2.39 (s, 3H), 2.33 (bs, 1H), 1.99−1.93 (m, 2H) ppm. ^{13}C{^{1}H} NMR (126 MHz, $CDCl_3$, APT) δ 137.8, 134.6, 134.0, 132.9, 130.2, 129.1, 128.5, 120.7, 86.8, 86.7, 70.5, 64.4, 27.4, 27.3, 24.6, 21.0 ppm. HRMS [TOF MS ES+]: m/z $[M + H]^+$ calcd. for $C_{20}H_{20}ClOS_2$ 375.0639, found 375.0644 (1.33 ppm).

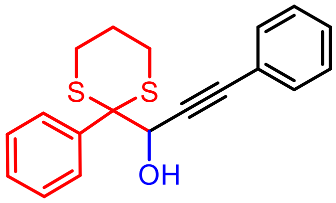

3-Phenyl-1-(2-phenyl-1,3-dithian-2-yl)prop-2-yn-1-ol (1d): The reaction was performed following the general procedure B with phenylacetylene (2.73 g, 26.7 mmol) in THF (40 mL), *n*-BuLi (28.5 mmol, 11.4 mL of a 2.5 M of hexane solution) and **S1b**, (2.0 g, 8.90 mmol) in THF (40 mL). After the resulting mixture was allowed to stir for 3h at −78 °C, the crude material was obtained as described in the general procedure and subjected to column chromatography on silica gel using 10:1 hexanes/ethyl acetate as eluent to yield **1d** (2.41 g, 83%, white solid, mp: 97-98 °C). ^{1}H-NMR (500 MHz, $CDCl_3$) δ 8.14−8.11 (m, 2H), 7.47−7.43 (m, 2H), 7.37−7.33 (m, 3H),

7.32−7.27 (m, 3H), 4.95 (s, 1H), 2.88−2.82 (m, 2H), 2.76−2.70 (m, 2H), 2.01−1.95 (m, 2H) ppm. $^{13}C\{^1H\}$ NMR (126 MHz, $CDCl_3$, APT) δ 137.3, 131.7, 130.4, 128.6, 128.4, 128.2, 128.0, 122.2, 88.0, 85.7, 70.6, 64.6, 27.4, 27.3, 24.7 ppm. HRMS [TOF MS ES+]: m/z $[M + H]^+$ calcd. for $C_{19}H_{19}OS_2$ 327.0872, found 327.0877 (1.53 ppm).

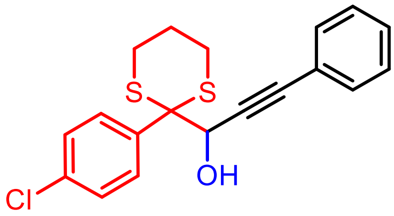

1-(2-(4-chlorophenyl)-1,3-dithian-2-yl)-3-phenylprop-2-yn-1-ol (1e): The reaction was performed following the general procedure B with phenylacetylene (0.74 g, 7.2 mmol) in THF (20 mL), *n*-BuLi (8.3 mmol, 3.3 mL of a 2.5 M of hexane solution) and **S1c**, (0.6 g, 2.32 mmol) in THF (10 mL). After the resulting mixture was allowed to stir for 3h at −78 °C, the crude material was obtained as described in the general procedure and subjected to column chromatography on silica gel using 10:1 hexanes/ethyl acetate as eluent to yield **1e** (0.62 g, 74%, white solid, mp: 107-108 °C). ^1H NMR (500 MHz, $CDCl_3$) δ 8.06 (d, J = 8.7 Hz, 2H), 7.40–7.35 (m, 4H), 7.33−7.27 (m, 3H), 4.94 (d, J = 7.8 Hz, 1H), 2.84–2.78 (m, 2H), 2.70–2.64 (m, 3H), 1.97–1.93 (m, 2H) ppm. $^{13}C\{^1H\}$ NMR (126 MHz, $CDCl_3$, APT) δ 135.8, 133.6, 131.8, 131.4, 128.4, 128.0, 127.9, 121.7, 87.9, 85.3, 70.0, 63.7, 27.0, 26.9, 24.2 ppm. HRMS [TOF MS ES+]: m/z $[M + H]^+$ calcd. for $C_{19}H_{18}ClOS_2$ 361.0482, found 361.0487 (1.38 ppm).

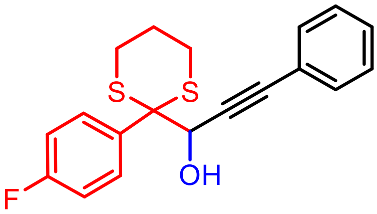

1-(2-(4-fluorophenyl)-1,3-dithian-2-yl)-3-phenylprop-2-yn-1-ol (1f): The reaction was performed following the general procedure B with phenylacetylene (0.81 g, 7.9 mmol) in THF (20 mL), *n*-BuLi (8.5 mmol, 3.4 mL of a 2.5 M of hexane solution) and **S1d**, (0.63 g, 2.32 mmol) in THF (10 mL). After the resulting mixture was allowed to stir for 3h at −78 °C, the crude material was obtained as described in the general procedure and subjected to column chromatography on silica gel using 30:1 hexanes/ethyl acetate as eluent to yield **1f** (0.82 g, 91%, white solid, mp: 94-95 °C). ^1H NMR (500 MHz, $CDCl_3$) δ 8.11−8.07 (m, 2H), 7.36–7.27 (m, 5H), 7.12−7.08 (m, 2H), 4.93 (d, J = 8.1 Hz, 1H), 2.87–2.81 (m, 2H), 2.75–2.66 (m, 2H). 2.48 (d, J = 8.0 Hz, 1H), 2.00–1.93 (m, 2H) ppm. $^{13}C\{^1H\}$ NMR (126 MHz, $CDCl_3$, APT) δ 162.4 (d, $^1J_{CF}$ = 248 Hz, $C_{quat.}$), 133.0 (d, $^4J_{CF}$ = 3.1 Hz), 132.4 (d, $^3J_{CF}$ = 8.0 Hz), 131.7, 128.7, 128.2, 115.1 (d, $^2J_{CF}$ = 21.4 Hz), 88.3,

85.4, 70.5, 63.9, 27,4, 27.3, 24.6 ppm. HRMS [TOF MS ES+]: m/z $[M + H]^+$ calcd. for $C_{19}H_{18}FOS_2$ 345.0778, found 345.0783 (1.45 ppm).

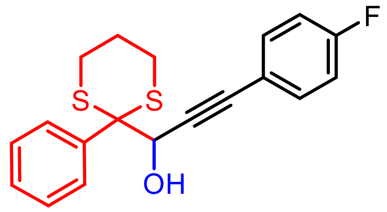

3-(4-Fluorophenyl)-1-(2-phenyl-1,3-dithian-2-yl)prop-2-yn-1-ol (**1g**): The reaction was performed following the general procedure B with 1-ethynyl-4-fluorobenzene (0.96 g, 8.0 mmol) in THF (20 mL), *n*-BuLi (8.5 mmol, 3.4 mL of a 2.5 M of hexane solution) and **S1b**, (0.6 g, 2.67 mmol) in THF (10 mL). After the resulting mixture was allowed to stir for 3h at −78 °C, the crude material was obtained as described in the general procedure and subjected to column chromatography on silica gel using 10:1 hexanes/ethyl acetate as eluent to yield **1g** (0.71 g, 77%, white solid, mp: 94-95 °C). ^1H NMR (500 MHz, $CDCl_3$) δ 8.11−8.09 (m, 2H), 7.45–7.42 (m, 2H), 7.36–7.31 (m, 3H), 6.99−6.95 (m, 2H), 4.92 (d, J = 7.7 Hz, 1H), 2.86–2.80 (m, 2H), 2.75–2.69 (m, 2H). 2.48 (d, J = 7.9 Hz, 1H), 2.00–1.93 (m, 2H) ppm. $^{13}C\{^1H\}$ NMR (126 MHz, $CDCl_3$, APT) δ 162.7 (d, $^1J_{CF}$ = 250 Hz, $C_{quat.}$), 137.3, 133.6 (d, $^3J_{CF}$ = 8.6 Hz), 130.3, 128.4, 128.0, 118.2 (d, $^4J_{CF}$ = 3.5 Hz, $C_{quat.}$), 115.5 (d, $^2J_{CF}$ = 22.1 Hz), 87.0, 85.4 (d, $^5J_{CF}$ = 1.5 Hz), 70.5, 64.6, 27,4, 27.3, 24.6 ppm. HRMS [TOF MS ES+]: m/z $[M + H]^+$ calcd. for $C_{19}H_{18}FOS_2$ 345.0778, found 345.0783 (1.45 ppm).

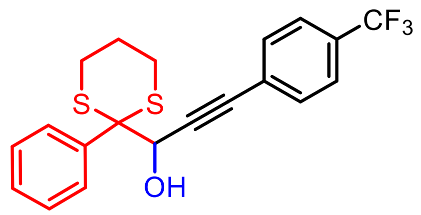

1-(2-phenyl-1,3-dithian-2-yl)-3-(4-(trifluoromethyl)phenyl)-prop-2-yn-1-ol (**1h**): The reaction was performed following the general procedure B with 1-ethynyl-4-trifluorobenzene (1.13 g, 6.6 mmol) in THF (15 mL), *n*-BuLi (7.1 mmol, 2.8 mL of a 2.5 M of hexane solution) and **S1b**, (0.5 g, 2.2 mmol) in THF (15 mL). After the resulting mixture was allowed to stir for 3h at −78 °C, the crude material was obtained as described in the general procedure and subjected to column chromatography on silica gel using 10:1 hexanes/ethyl acetate as eluent to yield **1h** (0.68 g, 77%, white solid, mp: 99-100 °C). ^1H-NMR (500 MHz, $CDCl_3$) δ 8.10−8.09 (m, 2H), 7.54−7.53 (m, 2H), 7.45−7.42 (m, 4H), 7.36−7.34 (m, 1H), 4.94 (s, 1H), 2.87−2.80 (m, 2H), 2.76−2.69 (m, 2H), 2.00−1.94 (m, 2H) ppm. $^{13}C\{^1H\}$ NMR (126 MHz, $CDCl_3$, APT) δ 137.2, 131.9, 130.3 (q, $^2J_{CF}$ = 32 Hz, C_{quat}), 130.2, 128.4, 128.1, 126.0 (q, $^5J_{CF}$ = 1.4 Hz, C_{quat}), 125.1 (q, $^3J_{CF}$ = 3.8

Hz), 123.8 (q, $^1J_{CF}$ = 272.0 Hz, CF_3), 88.1, 86.5, 70.5, 64.6, 27.4, 27.3, 24.6 ppm. HRMS [TOF MS ES+]: m/z $[M + H]^+$ calcd. for $C_{20}H_{18}F_3OS_2$ 395.0747, found 395.0753 (1.52 ppm).

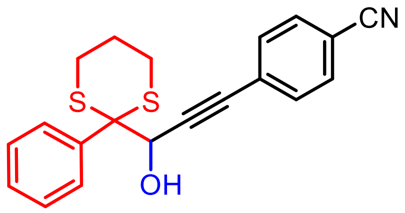

4-(3-Hydroxy-3-(2-phenyl-1,3-dithian-2-yl)prop-1-yn-1-yl)benzonitrile (**1i**): The reaction was performed following the general procedure B with 1-ethynyl-4-cyanobenzene (1.01 g, 8.0 mmol) in THF (40 mL), *n*-BuLi (8.5 mmol, 3.4 mL of a 2.5 M of hexane solution) and **S1b**, (0.6 g, 2.67 mmol) in THF (15 mL). After the resulting mixture was allowed to stir for 3h at −78 °C, the crude material was obtained as described in the general procedure and subjected to column chromatography on silica gel using 10:1 hexanes/ethyl acetate as eluent to yield **1i** (0.86 g, 93%, white solid, mp: 118-119 °C). ^{1}H NMR (500 MHz, $CDCl_3$) δ 8.08 (d, J = 8.2 Hz, 2H), 7.56 (d, J = 8.2 Hz, 2H), 7.45–7.41 (m, 4H), 7.37–7.34 (m, 1H), 4.93 (d, J = 7.8 Hz, 1H), 2.87–2.81 (m, 2H), 2.76–2.69 (m, 2H), 2.58 (d, J = 8.0 Hz, 1H), 2.00–1.95 (m, 2H) ppm. $^{13}C\{^1H\}$ NMR (126 MHz, $CDCl_3$, APT) δ 137.2, 132.2, 131.9, 130.1, 128.5, 128.2, 127.1, 118.3, 112.0, 90.2, 86.1, 70.5, 64.5, 27.4, 27.3, 24.5 ppm. HRMS [TOF MS ES+]: m/z $[M + H]^+$ calcd. for $C_{20}H_{18}NOS_2$ 352.0824, found 352.0831 (1.99 ppm).

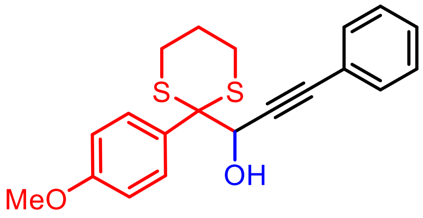

1-(2-(4-Methoxyphenyl)-1,3-dithian-2-yl)-3-phenylprop-2-yn-1-ol (**1j**): The reaction was performed following the general procedure B with phenylacetylene (0.72 g, 7.1 mmol) in THF (20 mL), *n*-BuLi (7.6 mmol, 3.0 mL of a 2.5 M of hexane solution) and **S1e**, (0.66 g, 2.59 mmol) in THF (10 mL). After the resulting mixture was allowed to stir for 3h at −78 °C, the crude material was obtained as described in the general procedure and subjected to column chromatography on silica gel using 10:1 hexanes/ethyl acetate as eluent to yield **1j** (0.62 g, 74%, white solid, mp: 121-122 °C). ^{1}H NMR (500 MHz, $CDCl_3$) δ 8.03 (d, J = 9.0 Hz, 2H), 7.39–7.37 (m, 2H), 7.33−7.27 (m, 3H), 6.96 (d, J = 9.0 Hz, 2H), 4.93 (d, J = 8.1 Hz, 1H), 3.84 (s, 3H), 2.87–2.81 (m, 2H), 2.76–2.70 (m, 2H), 2.50 (d, J = 8.3 Hz, 1H), 2.00–1.95 (m, 2H) ppm. $^{13}C\{^1H\}$ NMR (126 MHz, $CDCl_3$, APT) δ 159.2, 131.7, 131.6, 128.9, 128.6, 128.2, 122.2, 113.6, 87.9, 85.8,

70.6, 64.1, 55.9, 27.4, 27.3, 24.7 ppm. HRMS [TOF MS ES+]: m/z $[M + H]^+$ calcd. for $C_{20}H_{21}O_2S_2$ 357.0977, found 357.0982 (1.40 ppm).

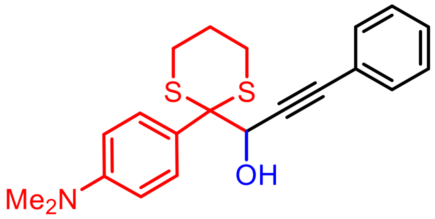

1-(2-(4-(Dimethylamino)phenyl)-1,3-dithian-2-yl)-3-phenylprop-2-yn-1-ol (**1k**): The reaction was performed following the general procedure B with phenylacetylene (0.66 g, 6.4 mmol) in THF (15 mL), *n*-BuLi (6.3 mmol, 2.5 mL of a 2.5 M of hexane solution) and **S1f**, (0.5 g, 2.0 mmol) in THF (10 mL). After the resulting mixture was allowed to stir for 3h at −78 °C, the crude material was obtained as described in the general procedure and subjected to column chromatography on silica gel using 10:1 hexanes/ethyl acetate as eluent to yield **1k** (0.56 g, 79%, white solid, mp: 113-114 °C). ^{1}H NMR (500 MHz, $CDCl_3$) δ 7.94 (d, J = 9.0 Hz, 2H), 7.42–7.40 (m, 2H), 7.30−7.27 (m, 3H), 6.75 (d, J = 9.0 Hz, 2H), 4.91 (bs, 1H), 2.98 (s, 6H), 2.85–2.71 (m, 4H), 2.52 (bs, 1H), 1.97–1.92 (m, 2H) ppm. $^{13}C\{^1H\}$ NMR (126 MHz, $CDCl_3$, APT) δ 149.8, 131.7, 131.2, 128.4, 128.1, 123.7, 122.4, 111.8, 87.7, 86.1, 70.6, 64.3, 40.3, 27.4, 27.3, 24.8 ppm. HRMS [TOF MS ES+]: m/z $[M + H]^+$ calcd. for $C_{21}H_{24}NOS_2$ 370.1294, found 370.1299 (1.35 ppm).

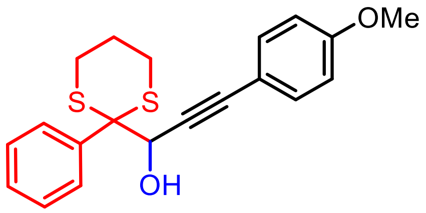

3-(4-Methoxyphenyl)-1-(2-phenyl-1,3-dithian-2-yl)prop-2-yn-1-ol (**1l**): The reaction was performed following the general procedure B with 1-ethynyl-4-methoxybenzene (1.06 g, 8.0 mmol) in THF (15 mL), *n*-BuLi (8.5 mmol, 3.4 mL of a 2.5 M of hexane solution) and **S1b**, (0.6 g, 2.67 mmol) in THF (15 mL). After the resulting mixture was allowed to stir for 3h at −78 °C, the crude material was obtained as described in the general procedure and subjected to column chromatography on silica gel using 10:1 hexanes/ethyl acetate as eluent to yield **1l** (0.82 g, 86%, white solid, mp: 117-118 °C). ^{1}H-NMR (500 MHz, $CDCl_3$) δ 8.13−8.11 (m, 2H), 7.46−7.42 (m, 2H), 7.36−7.33 (m, 1H), 7.31−7.28 (m, 2H), 6.82−6.79 (m, 2H), 4.93 (d, J = 6.6 Hz, 1H), 3.79 (s, 3H), 2.86−2.80 (m, 2H), 2.74−2.69 (m, 2H), 2.50 (bs, 1H), 1.99−1.94 (m, 2H) ppm. $^{13}C\{^1H\}$ NMR (126 MHz, $CDCl_3$, APT) δ 159.8, 137.3, 133.1, 130.4, 128.3, 127.9,

114.2, 113.8, 88.0, 84.3, 70.6, 64.6, 55.2, 27.4, 27.3, 24.6 ppm. HRMS [TOF MS ES+]: m/z $[M + H]^+$ calcd. for $C_{20}H_{21}O_2S_2$ 357.0977, found 357.0983 (1.68 ppm).

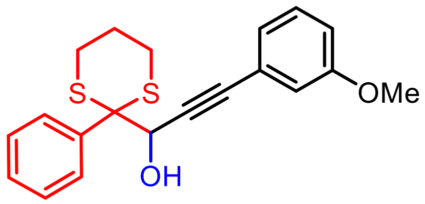

3-(3-Methoxyphenyl)-1-(2-phenyl-1,3-dithian-2-yl)prop-2-yn-1-ol (1m): The reaction was performed following the general procedure B with 1-ethynyl-3-methoxybenzene (1.06 g, 8.0 mmol) in THF (15 mL), *n*-BuLi (8.5 mmol, 3.4 mL of a 2.5 M of hexane solution) and **S1b**, (0.6 g, 2.67 mmol) in THF (15 mL). After the resulting mixture was allowed to stir for 3h at −78 °C, the crude material was obtained as described in the general procedure and subjected to column chromatography on silica gel using 10:1 hexanes/ethyl acetate as eluent to yield **1m** (0.85 g, 89%, white solid, mp: 95-96 °C). ^{1}H-NMR (500 MHz, $CDCl_3$) δ 8.11 (d, *J* = 7.9 Hz, 2H), 7.44 (t, *J* = 8.0 Hz, 2H), 7.34 (t, *J* = 7.0 Hz, 1H), 7.18 (t, *J* = 7.9 Hz, 1H), 6.95 (d, *J* = 7.5 Hz, 1H), 6.87−6.85 (m, 2H), 4.93 (d, *J* = 8.2 Hz, 1H), 3.77 (s, 3H), 2.87−2.80 (m, 2H), 2.75−2.69 (m, 2H), 2.50 (d, *J* = 8.5 Hz, 1H), 2.00−1.94 (m, 2H) ppm. $^{13}C\{^1H\}$ NMR (126 MHz, $CDCl_3$, APT) δ 159.1, 137.3, 130.3, 129.3, 128.3, 128.0, 124.2, 123.1, 116.5, 115.2, 87.9, 85.5, 70.6, 64.6, 55.2, 27.4, 27.3, 24.6 ppm. HRMS [TOF MS ES+]: m/z $[M + H]^+$ calcd. for $C_{20}H_{21}O_2S_2$ 357.0977, found 357.0983 (1.68 ppm).

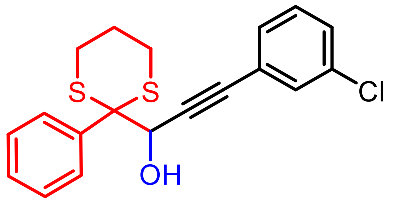

3-(3-Chlorophenyl)-1-(2-phenyl-1,3-dithian-2-yl)prop-2-yn-1-ol (1n): The reaction was performed following the general procedure B with 1-ethynyl-3-chlorobenzene (1.10 g, 8.0 mmol) in THF (15 mL), *n*-BuLi (8.5 mmol, 3.4 mL of a 2.5 M of hexane solution) and **S1b**, (0.6 g, 2.67 mmol) in THF (15 mL). After the resulting mixture was allowed to stir for 3h at −78 °C, the crude material was obtained as described in the general procedure and subjected to column chromatography on silica gel using 10:1 hexanes/ethyl acetate as eluent to yield **1n** (0.87 g, 91%, white solid, mp: 83-84 °C). ^{1}H-NMR (500 MHz, $CDCl_3$) δ 8.09 (d, *J* = 7.4 Hz, 2H), 7.44 (t, *J* = 7.8 Hz, 2H), 7.36−7.33 (m, 2H), 7.29−7.26 (m, 1H), 7.23−7.18 (m, 2H), 4.92 (d, *J* = 8.1 Hz, 1H), 2.86−2.80 (m, 2H), 2.75−2.68 (m, 2H), 2.56 (d, *J* = 8.2 Hz, 1H), 2.01−1.93 (m, 2H) ppm. $^{13}C\{^1H\}$ NMR (126 MHz, $CDCl_3$, APT) δ 137.2, 134.0, 131.5, 130.2, 129.8, 129.4, 128.9, 128.4, 128.0, 123.8,

86.9, 86.5, 70.5, 64.5, 27.4, 27.3, 24.6 ppm. HRMS [TOF MS ES+]: m/z $[M + H]^+$ calcd. for $C_{19}H_{18}ClOS_2$ 361.0482, found 361.0489 (1.94 ppm).

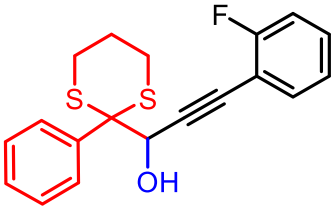

3-(2-Fluorophenyl)-1-(2-phenyl-1,3-dithian-2-yl)prop-2-yn-1-ol (1o): The reaction was performed following the general procedure B with 1-ethynyl-2-fluorobenzene (0.96 g, 8.0 mmol) in THF (20 mL), *n*-BuLi (8.5 mmol, 3.4 mL of a 2.5 M of hexane solution) and **S1b**, (0.6 g, 2.67 mmol) in THF (10 mL). After the resulting mixture was allowed to stir for 3h at −78 °C, the crude material was obtained as described in the general procedure and subjected to column chromatography on silica gel using 10:1 hexanes/ethyl acetate as eluent to yield **1o** (0.9 g, 99%, white solid, mp: 71-72 °C). ^1H NMR (500 MHz, $CDCl_3$) δ 8.13 (d, J = 8.0 Hz, 2H), 7.46–7.42 (m, 2H), 7.38–7.33 (m, 2H), 7.31–7.27 (m, 1H), 7.08–7.02 (m, 2H), 5.00 (d, J = 7.6 Hz, 1H), 2.89–2.83 (m, 2H), 2.76–2.69 (m, 2H), 2.58 (d, J = 8.4 Hz, 1H), 2.03–1.93 (m, 2H) ppm. $^{13}C\{^1H\}$ NMR (126 MHz, $CDCl_3$, APT) δ 162.8 (d, $^1J_{CF}$ = 253 Hz, $C_{quat.}$), 137.0, 133.7 (d, $^3J_{CF}$ = 2.5 Hz), 130.4, 130.3, 128.3, 127.9, 123.8 (d, $^3J_{CF}$ = 3.8 Hz), 115.4 (d, $^2J_{CF}$ = 21 Hz), 110.7 (d, $^2J_{CF}$ =16 Hz, $C_{quat.}$), 90.7 (d, $^3J_{CF}$ = 3.2 Hz, $C_{quat.}$), 81.6, 70.5, 64.2, 27,3, 27.2, 24.6 ppm. HRMS [TOF MS ES+]: m/z $[M + H]^+$ calcd. for $C_{19}H_{18}FOS_2$ 345.0778, found 345.0782 (1.16 ppm).

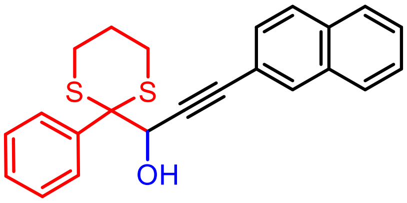

3-(Naphthalen-2-yl)-1-(2-phenyl-1,3-dithian-2-yl)prop-2-yn-1-ol (1q): The reaction was performed following the general procedure B with 1-ethynylnaphthalene (1.2 g, 8.0 mmol) in THF (25 mL), *n*-BuLi (8.5 mmol, 3.4 mL of a 2.5 M of hexane solution) and **S1b**, (0.6 g, 2.67 mmol) in THF (10 mL). After the resulting mixture was allowed to stir for 3h at −78 °C, the crude material was obtained as described in the general procedure and subjected to column chromatography on silica gel using 10:1 hexanes/ethyl acetate as eluent to yield **1q** (0.74 g, 74%, white solid, mp: 134-135 °C). ^1H NMR (500 MHz, $CDCl_3$) δ 8.16 (d, J = 7.2 Hz, 2H), 7.89 (s, 1H), 7.80–7.76 (m, 2H), 7.74 (d, J = 8.5 Hz, 1H), 7.50–7.44 (m, 4H), 7.40–7.35 (m, 2H), 5.00 (d, J = 8.2 Hz, 1H), 2.89–2.83 (m, 2H), 2.77–2.71 (m, 2H), 2.52 (d, J = 8.4 Hz, 1H), 2.02–1.94 (m, 2H) ppm. $^{13}C\{^1H\}$ NMR (126 MHz, $CDCl_3$, APT) δ 137.3, 132.9, 132.7, 131.7, 130.4, 128.4, 128.2, 128.0,

127.8, 127.7, 127.6, 126.7, 126.5, 119.4, 88.4, 86.0, 70.7, 64.6, 27.4, 27.3, 24.6 ppm. HRMS [TOF MS ES+]: m/z [M + H]$^+$ calcd. for $C_{23}H_{21}OS_2$ 377.1028, found 377.1034 (1.59 ppm).

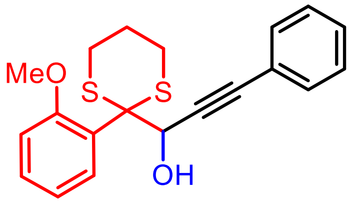

1-(2-(2-Methoxyphenyl)-1,3-dithian-2-yl)-3-phenylprop-2-yn-1-ol (**1p**): The reaction was performed following the general procedure B with phenylacetylene (0.8 g, 7.9 mmol) in THF (22 mL), *n*-BuLi (8.3 mmol, 3.3 mL of a 2.5 M of hexane solution) and **S1g**, (0.66 g, 2.6 mmol) in THF (10 mL). After the resulting mixture was allowed to stir for 3h at −78 °C, the crude material was obtained as described in the general procedure and subjected to column chromatography on silica gel using 10:1 hexanes/ethyl acetate as eluent to yield **1p** (0.55 g, 63%, white solid, mp: 97-98 °C). ^{1}H NMR (500 MHz, $CDCl_3$) δ 8.13 (dd, *J* = 7.9, 1.8 Hz, 1H), 7.40–7.35 (m, 1H), 7.27−7.21 (m, 5H), 7.06−7.03 (m, 1H), 6.97 (d, *J* = 8.1 Hz, 1H), 5.60 (d, *J* = 8.3 Hz, 1H), 3.90 (s, 3H), 3.53 (d, *J* = 8.4 Hz, 1H), 3.15–3.05 (m, 2H), 2.79–2.66 (m, 2H), 2.06–1.98 (m, 1H), 1.94-1.86 (m, 1H) ppm. ^{13}C{^{1}H} NMR (126 MHz, $CDCl_3$, APT) δ 157.1, 131.1, 130.6, 129.0, 127.8, 127.7, 122.1, 120.2, 112.5, 87.3, 85.7, 68.2, 65.3, 55.4, 26.7, 26.2, 23.1 ppm. HRMS [TOF MS ES+]: m/z [M + H]$^+$ calcd. for $C_{20}H_{21}O_2S_2$ 357.0977, found 357.0983 (1.68 ppm).

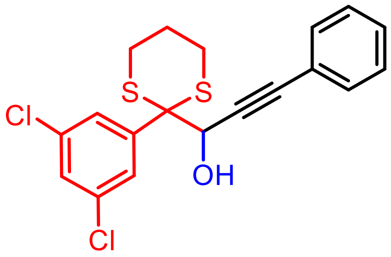

1-(2-(3,5-Dichlorophenyl)-1,3-dithian-2-yl)-3-phenylprop-2-yn-1-ol (**1r**): The reaction was performed following the general procedure B with phenylacetylene (0.56 g, 5.47 mmol) in THF (22 mL), *n*-BuLi (6.0 mmol, 2.5 mL of a 2.5 M of hexane solution) and **S1h**, (0.55 g, 1.79 mmol) in THF (10 mL). After the resulting mixture was allowed to stir for 3h at −78 °C, the crude material was obtained as described in the general procedure and subjected to column chromatography on silica gel using 10:1 hexanes/ethyl acetate as eluent to yield **1r** (0.54 g, 77%, white solid, mp: 132-133 °C). ^{1}H NMR (500 MHz, $CDCl_3$) δ 8.06 (d, *J* = 1.9 Hz, 2H), 7.41–7.38 (m, 2H), 7.36 (t, *J* = 1.9 Hz, 1H), 7.33−7.28 (m, 3H), 4.88 (d, *J* = 7.9 Hz, 1H), 2.86–2.79 (m, 2H), 2.72–2.65 (m, 2H), 2.44 (d, *J* = 8.0 Hz, 1H), 2.03–1.91 (m, 2H) ppm. ^{13}C{^{1}H} NMR (126 MHz, $CDCl_3$, APT) δ 141.5, 135.0, 131.8, 129.2, 128.9, 128.3, 128.2, 121.6, 89.0,

84.7, 70.5, 63.8, 27.4, 27.3, 24.3 ppm. HRMS [TOF MS ES+]: m/z $[M + H]^+$ calcd. for $C_{19}H_{17}Cl_2OS_2$ 395.0092, found 395.0099 (1.77 ppm).

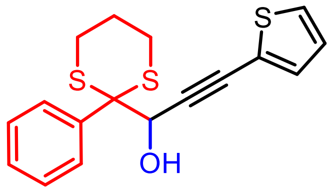

1-(2-Phenyl-1,3-dithian-2-yl)-3-(thiophen-2-yl)prop-2-yn-1-ol (**1s**): The reaction was performed following the general procedure B with 2-etynylthiophene (0.86 g, 8.0 mmol) in THF (22 mL), *n*-BuLi (8.5 mmol, 3.4 mL of a 2.5 M of hexane solution) and **S1b**, (0.6 g, 2.67 mmol) in THF (10 mL). After the resulting mixture was allowed to stir for 3h at −78 °C, the crude material was obtained as described in the general procedure and subjected to column chromatography on silica gel using 10:1 hexanes/ethyl acetate as eluent to yield **1s** (0.59 g, 67%, white solid, mp: 84-85 °C). ^1H NMR (500 MHz, $CDCl_3$) δ 8.1 (d, *J* = 7.3 Hz, 2H), 7.45–7.42 (m, 2H), 7.35–7.32 (m, 1H), 7.24 (d, *J* = 5.1 Hz, 1H), 7.15 (d, *J* = 3.7 Hz, 1H), 6.93 (dd, *J* = 5.0, 3.5 Hz, 1H), 4.94 (d, *J* = 8.3 Hz, 1H), 2.87–2.80 (m, 2H), 2.74–2.68 (m, 2H), 2.51–2.49 (m, 1H), 1.99–1.93 (m, 2H) ppm. $^{13}C\{^1H\}$ NMR (126 MHz, $CDCl_3$, APT) δ 137.1, 132.4, 130.2, 128.4, 128.0, 127.5, 126.8, 122.0, 89.7, 81.5, 70.6, 64.4, 27.4, 27.3, 24.6 ppm. HRMS [TOF MS ES+]: m/z $[M + H]^+$ calcd. for $C_{17}H_{17}OS_3$ 333.0436, found 333.0444 (2.40 ppm).

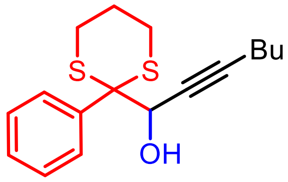

1-(2-Phenyl-1,3-dithian-2-yl)hept-2-yn-1-ol (**1t**): The reaction was performed following the general procedure B with 1-hexyne (0.66 g, 8.0 mmol) in THF (15 mL), *n*-BuLi (8.5 mmol, 3.4 mL of a 2.5 M of hexane solution) and **S1b**, (0.6 g, 2.67 mmol) in THF (10 mL). After the resulting mixture was allowed to stir for 3h at −78 °C, the crude material was obtained as described in the general procedure and subjected to column chromatography on silica gel using 10:1 hexanes/ethyl acetate as eluent to yield **1t** (0.31 g, 37%, white solid, mp: 73-74 °C). ^1H NMR (500 MHz, $CDCl_3$) δ 8.03−8.01 (m, 2H), 7.40–7.36 (m, 2H), 7.31−7.28 (m, 1H), 4.69–4.66 (m, 1H), 2.80–2.74 (m, 2H), 2.69–2.63 (m, 2H), 2.34–2.30 (m, 1H), 2.14 (td, *J* =10,0, 20.0 Hz, 2H), 1.94–1.89 (m, 2H), 1.44–1.38 (m, 2H), 1.36–1.28 (m, 2H), 2.14 (t, *J* = 7.2 Hz, 3H) ppm. $^{13}C\{^1H\}$ NMR (126 MHz, $CDCl_3$, APT) δ 137.3, 130.3, 128.1, 127.6, 89.2, 76.4, 70.2, 64.6, 30.2, 27.3, 27.1, 24.6, 21.7, 18.3, 13.4 ppm. HRMS [TOF MS ES+]: m/z $[M + H]^+$ calcd. for $C_{17}H_{23}OS_2$ 307.1185, found 307.1190 (1.63 ppm).

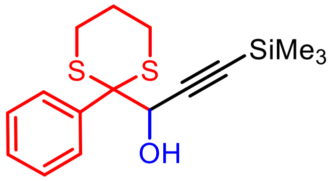

1-(2-Phenyl-1,3-dithian-2-yl)-3-(trimethylsilyl)prop-2-yn-1-ol (**1u**): The reaction was performed following the general procedure B with ethynyltrimethylsilane (0.81 g, 8.2 mmol) in THF (22 mL), *n*-BuLi (9.0 mmol, 3.6 mL of a 2.5 M of hexane solution) and **S1b**, (0.6 g, 2.67 mmol) in THF (10 mL). After the resulting mixture was allowed to stir for 3h at −78 °C, the crude material was obtained as described in the general procedure and subjected to column chromatography on silica gel using 10:1 hexanes/ethyl acetate as eluent to yield **1u** (0.83 g, 96%, colorless oil). ^{1}H NMR (500 MHz, $CDCl_3$) δ 8.01 (d, *J*=7.9 Hz, 2 H), 7.36 (t, *J*=7.6 Hz, 2 H), 7.28 (t, *J*=7.5 Hz, 1 H), 4.66 (d, *J*=7.7 Hz, 1 H), 2.88-2.73 (m, 2 H), 2.66-2.60 (m, 2 H), 2.58 (d, *J*=8.2 Hz, 1 H), 1.91-1.87 (m, 2 H), 0.10 (s, 9 H). ^{13}C{^{1}H} NMR (126 MHz, $CDCl_3$, APT) δ 136.7, 130.1, 127.7, 127.4, 101.4, 92.8, 69.9, 63.7, 26.9, 26.8, 24.2, −0.8 ppm. HRMS [TOF MS ES+]: m/z $[M + H]^+$ calcd. for $C_{16}H_{23}OSiS_2$ 323.0954, found 323.0960 (1.86 ppm).

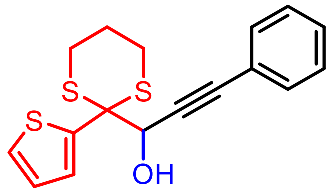

3-Phenyl-1-(2-(thiophen-2-yl)-1,3-dithian-2-yl)prop-2-yn-1-ol (**1v**): The reaction was performed following the general procedure B with phenylacetylene (0.84 g, 8.2 mmol) in THF (20 mL), *n*-BuLi (8.7 mmol, 3.5 mL of a 2.5 M of hexane solution) and **S1i**, (0.63 g, 2.7 mmol) in THF (10 mL). After the resulting mixture was allowed to stir for 3h at −78 °C, the crude material was obtained as described in the general procedure and subjected to column chromatography on silica gel using 10:1 hexanes/ethyl acetate as eluent to yield **1v** (0.6 g, 67%, white solid, mp: 77-78 °C). ^{1}H NMR (500 MHz, $CDCl_3$) δ 7.44−7.42 (m, 3 H), 7.40 (dd, *J*=1.4, 5.1 Hz, 1 H), 7.33−7.27 (m, 3 H), 7.07 (dd, *J*=3.6, 5.2 Hz, 1 H), 4.93 (d, *J*=9.2 Hz, 1 H), 2.98−2.82 (m, 4 H), 2.47 (d, *J*= 9.1 Hz, 1 H), 2.07−2.02 (m, 1 H), 1.98−1.89 (m, 1 H). ^{13}C{^{1}H} NMR (126 MHz, $CDCl_3$, APT) δ 144.4, 131.8, 130.1, 128.7, 128.2, 128.1, 127.4, 122.1, 88.4, 85.3, 71.01, 60.8, 27.9, 27.7, 24.6 ppm. HRMS [TOF MS ES+]: m/z $[M + H]^+$ calcd. for $C_{17}H_{17}OS_3$ 333.0436, found 333.0442 (1.81 ppm).

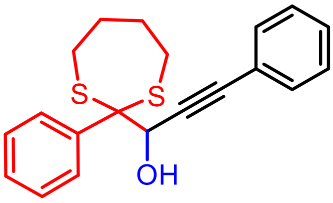

3-Phenyl-1-(2-phenyl-1,3-dithiepan-2-yl)prop-2-yn-1-ol (5a): The reaction was performed following the general procedure B with phenylacetylene (0.83 g, 8.1 mmol) in THF (20 mL), *n*-BuLi (8.6 mmol, 3.5 mL of a 2.5 M of hexane solution) and **S4a**, (0.65 g, 2.7 mmol) in THF (20 mL). After the resulting mixture was allowed to stir for 3h at −78 °C, the crude material was obtained as described in the general procedure and subjected to column chromatography on silica gel using 10:1 hexanes/ethyl acetate as eluent to yield **5a** (0.61 g, 67%, white solid, mp: 80-81 °C). ^{1}H-NMR (500 MHz, $CDCl_3$) δ 7.98 (d, J = 7.9 Hz, 2H), 7.42−7.39 (m, 2H), 7.33−7.25 (m, 6H), 4.95 (s, 1H), 3.29−3.23 (m, 1H), 3.15−3.10 (m, 1H), 3.04 (bs, 1H), 2.82−2.77 (m, 1H), 2.70−2.65 (m, 1H), 2.08−2.01 (m, 1H), 1.92−1.81 (m, 3H) ppm. ^{13}C{^{1}H} NMR (126 MHz, $CDCl_3$, APT) δ 140.9, 131.5, 128.8, 128.3, 128.1, 128.0, 127.7, 122.2, 87.0, 86.7, 73.0, 72.2, 66.9, 31.2, 31.1, 30.4, 30.3 ppm. HRMS [TOF MS ES+]: m/z $[M + H]^+$ calcd. for $C_{20}H_{21}OS_2$ 341.1028, found 341.1030 (0.59 ppm).

5.2. General Procedure C: To a solution of acetylene (1.5 equiv) in THF cooled to −78 °C was added dropwise *n*-BuLi (2.5 M in hexanes, 1.0 equiv) under nitrogen atmosphere. The solution was allowed to stir for 30 min before being transferred via cannula into a solution of 1,3-dithioacetyl-2-carbaldehyde (1.0 equiv) in THF under nitrogen atmosphere cooled to −78 °C. The resulting mixture was allowed to stir for 3h at −78 °C. The mixture was quenched with 10% NH_4Cl (20 mL) solution and extracted with CH_2Cl_2 (2 × 100 mL). The combined organic layers were dried with anhydrous Na_2SO_4 and filtered. To the solution silica gel (2 g) was added and the solvent was removed in a rotatory evaporator. The residue was subjected to column chromatography on silica gel using a mixture of hexanes and ethyl acetate as eluent to yield propargyl alcohols **3a–f**.

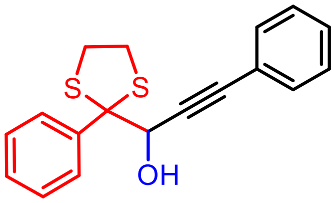

3-Phenyl-1-(2-phenyl-1,3-dithiolan-2-yl)prop-2-yn-1-ol (3a): The reaction was performed following the general procedure C with phenylacetylene (0.44 g, 4.28 mmol) in THF (15 mL), *n*-BuLi (2.85 mmol, 1.14 mL of a 2.5 M of hexane solution) and **S3a**, (0.6 g, 2.85

mmol) in THF (15 mL). After the resulting mixture was allowed to stir for 3h at −78 °C, the crude material was obtained as described in the general procedure and subjected to column chromatography on silica gel using 10:1 hexanes/ethyl acetate as eluent to yield **3a** (0.72 g, 81%, white solid, mp: 100-101 °C). ^{1}H-NMR (500 MHz, $CDCl_3$) δ 7.80−7.78 (m, 2H), 7.39−7.27 (m, 8H), 5.04 (s, 1H), 3.53−3.46 (m, 2H), 3.39−3.34 (m, 2H), 2.97 (bs, 1H) ppm. ^{13}C{^{1}H} NMR (126 MHz, $CDCl_3$, APT) δ 141.2, 131.5, 128.5, 128.3, 128.1, 127.8, 122.1, 87.6, 86.5, 79.4, 72.2, 40.5, 39.9 ppm. HRMS [TOF MS ES+]: m/z $[M + H]^+$ calcd. for $C_{18}H_{17}OS_2$ 313.0715, found 313.0721 (1.92 ppm).

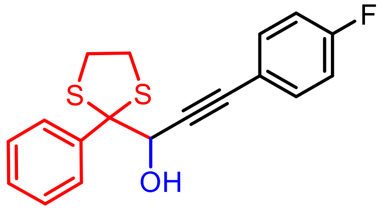

3-(4-Fluorophenyl)-1-(2-phenyl-1,3-dithiolan-2-yl)prop-2-yn-1-ol (**3b**): The reaction was performed following the general procedure C with 1-ethynyl-4-fluorobenzene (0.57 g, 4.7 mmol) in THF (30 mL), *n*-BuLi (3.25 mmol, 1.3 mL of a 2.5 M of hexane solution) and **S3a**, (0.66 g, 3.13 mmol) in THF (10 mL). After the resulting mixture was allowed to stir for 3h at −78 °C, the crude material was obtained as described in the general procedure and subjected to column chromatography on silica gel using 10:1 hexanes/ethyl acetate as eluent to yield **3b** (0.89 g, 86%, white solid, mp: 88-89 °C). ^{1}H NMR (500 MHz, $CDCl_3$) δ 7.78−7.75 (m, 2H), 7.78–7.35 (m, 2H), 7.33–7.27 (m, 3H), 6.99−6.95 (m, 2H), 5.00 (d, J = 8.3 Hz, 1H), 3.54–3.48 (m, 2H), 3.40–3.35 (m, 2H), 2.93 (d, J = 8.3 Hz, 1H) ppm. ^{13}C{^{1}H} NMR (126 MHz, $CDCl_3$, APT) δ 162.3 (d, $^{1}J_{CF}$ = 250 Hz, $C_{quat.}$), 141.1, 133.3 (d, $^{3}J_{CF}$ = 8.5 Hz), 128.2, 127.7, 127.6, 118.0 (d, $^{4}J_{CF}$ = 3.5 Hz, $C_{quat.}$), 115.3 (d, $^{2}J_{CF}$ = 22.0 Hz), 87.3 (d, $^{5}J_{CF}$ = 1.5 Hz, C_{quat}), 85.3, 79.1, 72.0, 40,3, 39.8 ppm. HRMS [TOF MS ES+]: m/z $[M + H]^+$ calcd. for $C_{18}H_{16}FOS_2$ 331.0621, found 331.0627 (1.81 ppm).

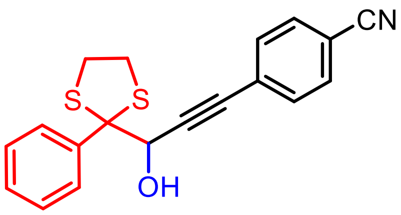

4-(3-Hydroxy-3-(2-phenyl-1,3-dithiolan-2-yl)prop-1-yn-1-yl)benzonitrile (**3c**): The reaction was performed following the general procedure C with 1-ethynyl-4-cyanobenzene (0.77 g, 6.1 mmol) in THF (30 mL), *n*-BuLi (4.1 mmol, 1.6 mL of a 2.5 M of hexane solution) and **S3a**, (0.86 g, 4.1 mmol) in THF (15 mL). After the resulting mixture was allowed to stir for 3h at −78 °C, the crude material was obtained as described in the general procedure and subjected to column chromatography on silica gel using 10:1 hexanes/ethyl acetate as eluent to

yield **3c** (0.79 g, 57%, white solid, mp: 112-113 ºC). ^{1}H NMR (500 MHz, $CDCl_3$) δ 7.73 (d, *J* = 8.6 Hz, 2H), 7.54 (d, *J* = 8.2 Hz, 2H), 7.37–7.34 (m, 4H), 7.32–7.29 (m, 1H), 5.01 (s, 1H), 3.52–3.46 (m, 2H), 3.39–3.34 (m, 2H), 3.07 (bs, 1H) ppm. ^{13}C{^{1}H} NMR (126 MHz, $CDCl_3$, APT) δ 141.0, 132.1, 131.9, 128.2, 128.0, 127.0, 118.3, 111.9, 92.0, 84.8, 79.3, 72.1, 40.6, 40.1 ppm. HRMS [TOF MS ES+]: m/z $[M + H]^+$ calcd. for $C_{19}H_{16}NOS_2$ 338.0668, found 338.0673 (1.48 ppm).

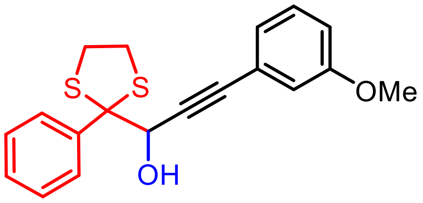

3-(3-Methoxyphenyl)-1-(2-phenyl-1,3-dithiolan-2-yl)prop-2-yn-1-ol (**3d**): The reaction was performed following the general procedure C with 1-ethynyl-3-methoxybenzene (0.48 g, 3.6 mmol) in THF (22 mL), *n*-BuLi (2.25 mmol, 0.9 mL of a 2.5 M of hexane solution) and **S3a**, (0.48 g, 2.26 mmol) in THF (15 mL). After the resulting mixture was allowed to stir for 3h at −78 ºC, the crude material was obtained as described in the general procedure and subjected to column chromatography on silica gel using 10:1 hexanes/ethyl acetate as eluent to yield **3d** (0.42 g, 34%, white solid, mp: 91-92 ºC). ^{1}H-NMR (500 MHz, $CDCl_3$) δ 7.77−7.75 (m, 2H), 7.37−7.34 (m, 2H), 7.32−7.28 (m, 1H), 7.18 (t, *J* = 8.9 Hz, 1H), 6.91−6.90 (m, 1H), 6.87−6.83 (m, 2H), 5.00 (s, 1H), 3.77 (s, 3H), 3.53−3.48 (m, 2H), 3.39−3.34 (m, 2H), 2.92 (d, *J* = 9.5 Hz, 1H) ppm. ^{13}C{^{1}H} NMR (126 MHz, $CDCl_3$, APT) δ 159.2, 141.3, 129.3, 128.4, 127.9, 127.8, 124.1, 123.1, 116.5, 115.1, 87.4, 86.5, 79.5, 72.2, 55.2, 40.6, 40.0 ppm. HRMS [TOF MS ES+]: m/z $[M + H]^+$ calcd. for $C_{19}H_{19}O_2S_2$ 343.0821, found 343.0826 (1.46 ppm).

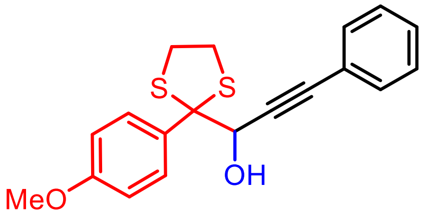

1-(2-(4-Methoxyphenyl)-1,3-dithiolan-2-yl)-3-phenylprop-2-yn-1-ol (**3e**): The reaction was performed following the general procedure C with phenylacetylene (0.57 g, 5.6 mmol) in THF (20 mL), *n*-BuLi (3.8 mmol, 1.5 mL of a 2.5 M of hexane solution) and **S3b**, (0.9 g, 3.8 mmol) in THF (10 mL). After the resulting mixture was allowed to stir for 3h at −78 ºC, the crude material was obtained as described in the general procedure and subjected to column chromatography on silica gel using 10:1 hexanes/ethyl acetate as eluent to yield **3e** (0.91 g, 71%, white solid, mp: 110-111 ºC). ^{1}H NMR (500 MHz, $CDCl_3$) δ 7.71–7.68 (m, 2H), 7.34−7.26 (m, 5H), 6.90−6.87 (m, 2H), 4.98 (d, *J* = 8.3 Hz, 1H), 3.81 (s, 3H), 3.52–3.45 (m, 2H), 3.39–3.33 (m, 2H),

2.90 (d, J = 8.5 Hz, 1H) ppm. $^{13}C\{^1H\}$ NMR (126 MHz, $CDCl_3$, APT) δ 159.1, 131.3, 131.6, 129.7, 128.5, 128.2, 122.2, 113.1, 87.7, 86.5, 78.9, 72.2, 55.3, 40.6, 40.1 ppm. HRMS [TOF MS ES+]: m/z $[M + H]^+$ calcd. for $C_{19}H_{19}O_2S_2$ 343.0832, found 343.0819 (–3.79 ppm).

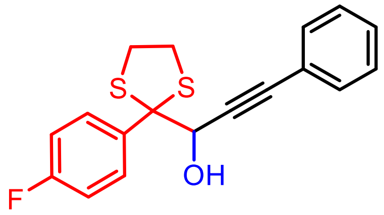

1-(2-(4-fluorophenyl)-1,3-dithian-2-yl)-3-phenylprop-2-yn-1-ol (3f): The reaction was performed following the general procedure C with phenylacetylene (0.58 g, 5.7 mmol) in THF (20 mL), *n*-BuLi (3.77 mmol, 1.5 mL of a 2.5 M of hexane solution) and **S3c**, (0.86 g, 3.77 mmol) in THF (10 mL). After the resulting mixture was allowed to stir for 3h at −78 °C, the crude material was obtained as described in the general procedure and subjected to column chromatography on silica gel using 10:1 hexanes/ethyl acetate as eluent to yield **3f** (1.06 g, 85%, white solid, mp: 82-83 °C). ^1H NMR (500 MHz, $CDCl_3$) δ 7.78−7.75 (m, 2H), 7.34–7.27 (m, 5H), 7.06−7.02 (m, 2H), 4.99 (s, 1H), 3.53–3.47 (m, 2H), 3.59–3.34 (m, 2H) ppm. $^{13}C\{^1H\}$ NMR (126 MHz, $CDCl_3$, APT) δ 162.2 (d, $^1J_{CF}$ = 247 Hz), 137.2 (d, $^4J_{CF}$ = 3.3 Hz), 131.6, 130.3 (d, $^3J_{CF}$ = 8.0 Hz), 128.7, 128.2, 121.9 114.5 (d, $^2J_{CF}$ = 21.3 Hz), 87.4, 86.8, 78.7, 72.1, 40.6, 40.1 ppm. HRMS [TOF MS ES+]: m/z $[M + H]^+$ calcd. for $C_{18}H_{16}FOS_2$ 331.0621, found 331.0621 (0.0 ppm).

6. Synthesis of 6-, 7-, and 8-Membered *S,S*-Heterocyle-Fused Benzofulvenes

6.1. General procedure D: An oven-dried 15 mL screw-cap reaction vial equipped with a stirring bar was charged with α-dithioacetyl propargyl alcohol derivative (1.0 equiv.) and then the vial was brought into a glovebox. The reaction vial was charged with $Ga(OTf)_3$ (0.05 equiv. 5mol%) and anhydrous toluene. The vial was tightly closed, wrapped with a strip of Parafilm, and taken out of the glovebox. After having stirred the reaction mixture for the given time at 110 °C in a pre-heated oil bath, the vial was cooled to room temperature. The reaction mixture was taken into a 50 mL flask and the solvent was removed in a rotatory evaporator. The remaining residue was dissolved in CH_2Cl_2 and mixed with silica gel (about 0.5-1.0 g). After evaporating CH_2Cl_2, the remaining silica gel was directly loaded onto a column and purified by flash chromatography on silica gel using hexanes/ethyl acetate mixture as eluent to yield the products **(2a-v, 4a–f and 6a)**.

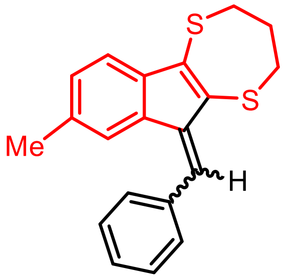

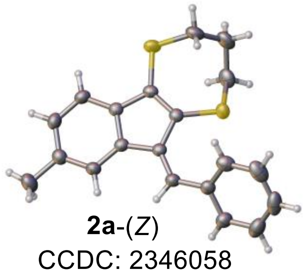

2a-(*Z*)
CCDC: 2346058

(*E*)/(*Z*)-10-Benzylidene-8-methyl-3,4-dihydro-2*H*,10*H*-indeno[1,2-*b*][1,4]dithiepine (2a): The reaction was performed following General Procedure D with propargyl alcohol (**1a**) (0.25 mmol, 85 mg) and Ga(OTf)$_3$ (0.0125 mmol, 6.5 mg) in anhydrous toluene (2.0 mL). The reaction was stirred for 5 min at 110 ^{o}C in an oil bath. The workup was performed following the general procedure. The crude material was purified by flash chromatography on silica gel using 60:1 hexanes/ethyl acetate as eluent to yield the product **2a** (68.5 mg, 85%, red oil) as a mixture of *E*/*Z* isomers (68.5 mg, 85%, red oil). Isomers are partially separated by flash chromatography on silica gel using hexanes as eluent. **2a-(*E*)**: ^{1}H-NMR (500 MHz, CDCl$_3$) δ 7.56–7.55 (m, 2H), 7.46–7.43 (m, 2H), 7.40–7.37 (m, 1H), 7.34 (s, 1H), 7.20 (s, 1H), 7.11 (d, J = 7.5 Hz, 1H, AB$_{system}$, δ_A), 7.03 (d, J = 7.5 Hz, 1H, AB$_{system}$, δ_B), 3.47 (t, J = 6.0 Hz, 2H), 3.41 (t, J = 6.0 Hz, 2H), 2.43–2.36 (m, 2H), 2.20 (s, 3H) ppm. ^{13}C{^{1}H} NMR (126 MHz, CDCl$_3$, APT) δ 140.8, 139.8, 136.6, 135.6, 134.6, 133.8, 131.4, 129.6, 129.4, 128.4, 128.2, 128.1, 123.0, 117.6, 33.0, 32.8, 31.0, 21.5 ppm. HRMS [TOF MS ES+]: m/z [M + H]$^+$ calcd. for $C_{20}H_{19}S_2$ 323.0923, found 323.0922 (– 0.3 ppm). **2a-(*Z*)**: ^{1}H-NMR (500 MHz, CDCl$_3$) δ 7.44–7.39 (m, 5H), 7.37–7.34 (m, 2H), 7.08 (s, 2H), 3.65 (t, J = 6.1 Hz, 2H), 3.33 (t, J = 6.1 Hz, 2H), 2.41 (s, 3H), 2.24–2.1 (m, 2H) ppm. ^{13}C{^{1}H} NMR (126 MHz, CDCl$_3$, APT) δ 139.8, 138.4, 138.2, 137.0, 136.6, 135.0, 130.2, 128.1, 128.0, 127.8, 125.4, 125.2, 118.8, 117.2, 34.1, 31.2, 30.2, 21.6 ppm. HRMS [TOF MS ES+]: m/z [M + H]$^+$ calcd. for $C_{20}H_{19}S_2$ 323.0923, found 323.0927 (1.24 ppm).

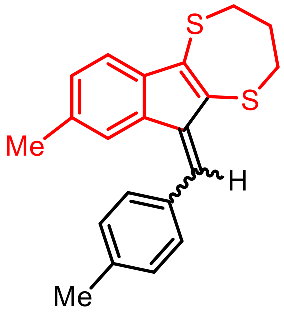

(*E*)/(*Z*)-8-Methyl-10-(4-methylbenzylidene)-3,4-dihydro-2*H*,10*H*-indeno[1,2-*b*][1,4]dithiepine (2b): The reaction was performed following General Procedure D with propargyl alcohol (**1b**) (0.25 mmol, 89 mg) and Ga(OTf)$_3$ (0.0125 mmol, 6.5 mg) in anhydrous toluene (2.0 mL). The reaction was stirred for 5 min at 110 °C in an oil bath. The workup was performed following the general procedure. The crude material was purified by flash chromatography on silica gel using 60:1 hexanes/ethyl acetate as eluent to yield the product **2b** as a mixture of *E*/*Z* isomers (73.5 mg, 87%, red oil). Isomers are partially separated by flash chromatography on silica gel using hexanes as eluent. **2b-(*E*)**: ^{1}H-NMR (500 MHz, CDCl$_3$) δ 7.45 (d, *J* = 8.0 Hz, 2H), 7.30 (s, 1H), 7.29 (s, 1H), 7.23 (d, *J* = 8.0 Hz, 2H), 7.09 (d, *J* = 7.7 Hz, 1H, AB$_{system}$, δ$_A$), 7.01 (d, *J* = 7.7 Hz, 1H, AB$_{system}$, δ$_B$), 3.44 (t, *J* = 6.0 Hz, 2H), 3.38 (t, *J* = 6.0 Hz, 2H), 2.43 (s, 3H), 2.41−2.35 (m, 2H), 2.20 (s, 3H) ppm. ^{13}C{^{1}H} NMR (126 MHz, CDCl$_3$, APT) δ 140.8, 139.2, 138.1, 135.2, 134.6, 133.9, 133.6, 131.8, 130.0, 129.5, 129.0, 128.4, 123.0, 117.6, 32.9, 32.8, 31.0, 21.6, 21.4 ppm. HRMS [TOF MS ES+]: m/z [M + H]$^+$ calcd. for $C_{21}H_{21}S_2$ 337.1079, found 337.1084 (1.48 ppm). **2b-(*Z*):** ^{1}H-NMR (500 MHz, CDCl$_3$) δ 7.44 (s, 1H), 7.38−7.36 (m, 3H), 7.24 (d, *J* = 7.9 Hz, 2H), 7.12−7.08 (m, 2H), 3.67 (t, *J* = 6.1 Hz, 2H), 3.36 (t, *J* = 6.1 Hz, 2H), 2.44 (s, 6H), 2.24 (p, *J* = 6.1 Hz, 2H) ppm. ^{13}C{^{1}H} NMR (126 MHz, CDCl$_3$, APT) δ 139.2, 138.2, 138.0, 137.8, 137.0, 134.9, 133.6, 130.3, 128.6, 127.9, 125.4, 125.3, 118.7, 117.1, 34.1, 31.2, 30.1, 21.6, 21.4 ppm. HRMS [TOF MS ES+]: m/z [M + H]$^+$ calcd. for $C_{21}H_{21}S_2$ 337.1079, found 337.1076 (– 0.89 ppm).

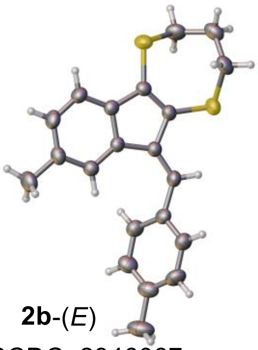

2b-(*E*)
CCDC: 2346067

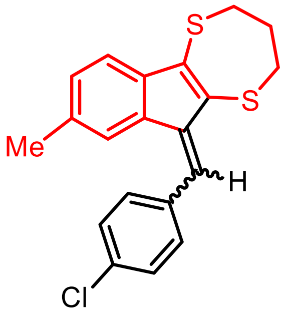

(*E*)/(*Z*)-10-(4-Chlorobenzylidene)-8-methyl-3,4-dihydro-2*H*,10*H*-indeno[1,2-*b*][1,4]dithiepine (2c): The reaction was performed following General Procedure D with propargyl alcohol (**1c**) (0.25 mmol, 94 mg) and Ga(OTf)$_3$ (0.0125 mmol, 6.5 mg) in anhydrous toluene (2.0 mL). The reaction was stirred for 5 min at 110 °C in an oil bath. The workup was performed following the general procedure. The crude material was

purified by flash chromatography on silica gel using 60:1 hexanes/ethyl acetate as eluent to yield the product **2b** (69 mg, 77%, red oil) as a mixture of *E*/*Z* isomers. Isomers are partially separated by flash chromatography on silica gel using hexanes as eluent. **2c-(*E*)**: ^{1}H-NMR (500 MHz, $CDCl_3$): δ 7.47−7.46 (m, 2H, AA'BB'$_{system}$, $\delta_{AA'}$), 7.40−7.38 (m, 2H, AA'BB'$_{system}$, $\delta_{BB'}$), 7.20 (s, 1H), 7.16 (s, 1H), 7.08 (d, *J* = 7.5 Hz, 2H, AB$_{system}$, δ_A), 7.02 (d, *J* = 7.5 Hz, 2H, AB$_{system}$, δ_B), 3.46 (t, *J* = 6.0 Hz, 2H), 3.39 (t, *J* = 6.1 Hz, 2H), 2.35−2.40 (m, 2H), 2.20 (s, 3H) ppm. ^{13}C{^{1}H} NMR (126 MHz, $CDCl_3$, APT) δ 140.8, 140.3, 136.0, 135.1, 134.9, 133.9, 133.6, 131.2, 130.8, 128.7, 128.5, 127.9, 122.9, 117.8, 32.8, 32.7, 30.9, 21.6 ppm. HRMS [TOF MS ES+]: m/z $[M + H]^+$ calcd. for $C_{20}H_{18}ClS_2$ 357.0533, found 357.0537 (1.12 ppm). **Bzf-7c-(*Z*):** ^{1}H-NMR (500 MHz, $CDCl_3$) δ 7.38−7.34 (m, 4H), 7.33−7.32 (m, 2H), 7.10−7.07 (m, 2H), 3.64 (t, *J* = 6.1 Hz, 2H), 3.34 (t, *J* = 6.1 Hz, 2H), 2.41 (s, 3H), 2.22 (p, *J* = 6.1 Hz, 2H) ppm. ^{13}C{^{1}H} NMR (126 MHz, $CDCl_3$, APT) δ 140.2, 138.8, 138.3, 136.8, 135.1, 134.9, 133.9, 131.4, 128.2, 128.0, 124.9, 123.6, 118.9, 117.2, 34.0, 31.1, 30.2, 21.6 ppm. HRMS [TOF MS ES+]: m/z $[M + H]^+$ calcd. for $C_{20}H_{18}ClS_2$ 357.0533, found 357.0549 (4.48 ppm).

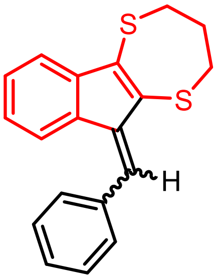

(*E*)/(*Z*)-10-Benzylidene-3,4-dihydro-2*H*,10*H*-indeno[1,2-*b*][1,4]dithiepine (2d): The reaction was performed following General Procedure D with propargyl alcohol (**1d**) (0.50 mmol, 163 mg) and $Ga(OTf)_3$ (0.025 mmol, 13.0 mg) in anhydrous toluene (3.0 mL). The reaction was stirred for 5 min at 110 °C in an oil bath. The workup was performed following the general procedure. The crude material was purified by flash chromatography on silica gel using 60:1 hexanes/ethyl acetate as eluent to yield the product **2d** as a mixture of *E*/*Z* isomers (130 mg, 84%, red oil). Isomers are partially separated by flash chromatography on silica gel using hexanes as eluent. **2d-(*E*)**: ^{1}H-NMR (500 MHz, $CDCl_3$) δ 7.49 (d, *J* = 7.7 Hz, 2H), 7.37−7.40 (m, 2H), 7.31−7.34 (m, 3H), 7.15−7.20 (m, 2H), 6.86−6.91 (m, 1H), 3.47 (t, *J* = 6.0 Hz, 2H), 3.37 (t, *J* = 6.0 Hz, 2H), 2.31−2.35 (m, 2H) ppm. ^{13}C{^{1}H} NMR (126 MHz, $CDCl_3$, APT) δ 143.2, 139.7, 136.5, 135.3, 133.5, 132.3, 130.0, 129.3, 128.3, 128.1, 127.9, 124.9, 122.0, 117.9, 32.7, 32.6, 30.1 ppm. HRMS [TOF MS ES+]: m/z $[M + H]^+$ calcd. for $C_{19}H_{17}S_2$ 309.0766, found 309.0768 (0.65 ppm). **2d-(*Z*)**: ^{1}H-NMR (500 MHz, $CDCl_3$) δ 7.55 (d, *J* = 7.3 Hz, 1H), 7.37−7.49 (m, 6H), 7.28−7.31 (m, 1H), 7.21−7.23 (m, 2H), 3.68 (t, *J* = 6.1

Hz, 2H), 3.36 (t, J = 6.1 Hz, 2H), 2.33 (p, J = 6.0 Hz, 2H) ppm. $^{13}C\{^1H\}$ NMR (126 MHz, $CDCl_3$, APT) δ 140.7, 139.6, 138.0, 136.7, 136.4, 130.2, 128.1, 127.8, 127.5, 126.3, 125.8, 125.2, 117.9, 117.4, 34.0, 31.1, 30.2 ppm. HRMS [TOF MS ES+]: m/z $[M + H]^+$ calcd. for $C_{19}H_{17}S_2$ 309.0766, found 309.0768 (0.65 ppm).

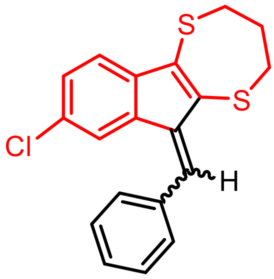

(*E*)/(*Z*)-10-Benzylidene-8-chloro-3,4-dihydro-2*H*,10*H*-indeno[1,2-b][1,4]dithiepine (2e): The reaction was performed following General Procedure D with propargyl alcohol (**1e**) (0.50 mmol, 180 mg) and $Ga(OTf)_3$ (0.025 mmol, 13.0 mg) in anhydrous toluene (3.0 mL). The reaction was stirred for 10 min at 110 °C in an oil bath. The workup was performed following the general procedure. The crude material was purified by flash chromatography on silica gel using 60:1 hexanes/ethyl acetate as eluent to yield the product **2e** (136 mg, 79%, red solid, mp: 98-99 °C) as a mixture of *E*/*Z* isomers. Isomers are partially separated by flash chromatography on silica gel using hexanes as eluent. **2e-(*E*)**: ^1H NMR (500 MHz, $CDCl_3$) δ 7.52–7.51 (m, 2H), 7.47–7.44 (m, 2H), 7.42–7.39 (m, 2H), 7.32 (d, J = 1.9 Hz, 1H), 7.17 (dd, J = 1.9, 8.1 Hz, 1H, AB_{system}, δ_A), 7.09 (d, J = 8.1 Hz, 1H, AB_{system}, δ_B), 3.47 (t, J = 5.9 Hz, 2H), 3.42 (t, J = 6.0 Hz, 2H), 2.39–2.33 (m, 2H). $^{13}C\{^1H\}$ NMR (126 MHz, $CDCl_3$, APT) δ 141.5, 138.7, 135.8, 134.9, 134.6, 132.8, 131.2, 130.8, 129.3, 128.5, 128.4, 127.6, 122.2, 118.6, 32.6, 32.5, 31.0 ppm. HRMS [TOF MS ES+]: m/z $[M + H]^+$ calcd. for $C_{19}H_{16}ClS_2$ 343.0376, found 343.0386 (2.92 ppm). **2e-(*Z*)**: ^1H NMR (500 MHz, $CDCl_3$) δ 7.49 (d, J = 1.8 Hz, 1H), 7.44–7.36 (m, 6H), 7.23 (dd, J = 2.1, 8.0 Hz, 1H, AB_{system}, δ_A), 7.08 (d, J = 8.0 Hz, 1H, AB_{system}, δ_B), 3.65 (t, J = 6.0 Hz, 2H), 3.34 (t, J = 6.0 Hz, 2H), 2.22 (t, J = 6.0 Hz, 2H). $^{13}C\{^1H\}$ NMR (126 MHz, $CDCl_3$, APT) δ 139.1, 138.6, 138.3, 137.3, 136.0, 131.2, 130.2, 128.4, 127.9, 127.8, 127.2, 126.9, 126.8, 118.4, 118.2, 34.0, 31.0, 30.3 ppm. HRMS [TOF MS ES+]: m/z $[M + H]^+$ calcd. for $C_{19}H_{16}ClS_2$ 343.0376, found 343.0386 (2.92 ppm).

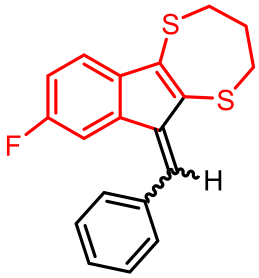

(*E*)/(*Z*)-10-Benzylidene-8-fluoro-3,4-dihydro-2*H*,10*H*-indeno[1,2-*b*][1,4]dithiepine (2f): The reaction was performed following General Procedure D with propargyl alcohol (**1f**) (0.42 mmol, 143 mg) and $Ga(OTf)_3$ (0.021 mmol, 11.0 mg) in anhydrous toluene (3.0 mL). The reaction was stirred for 15 min at 110 °C in an oil bath. The workup was performed following the general procedure. The crude material was purified by flash chromatography on silica gel using 60:1 hexanes/ethyl acetate as eluent to yield the product **2f** (108 mg, 79%, red oil) as a mixture of *E*/*Z* isomers. Isomers are partially separated by flash chromatography on silica gel using hexanes as eluent. **2f-(*E*)**: 7.50–7.49 (m, 2H), 7.46–7.43 (m, 2H), 7.41–7.38 (m, 2H), 7.10 (dd, *J* = 5.2, 8.2 Hz, 1H), 7.03 (dd, *J* = 2.5, 9.8 Hz, 1H), 6.89 (dt, *J* = 2.4, 9.0 Hz, 1H), 3.47 (t, *J* = 6.0 Hz, 2H), 3.40 (t, *J* = 6.0 Hz, 2H), 2.41–2.36 (m, 2H) ppm. $^{13}C\{^1H\}$ NMR (126 MHz, $CDCl_3$, APT) δ 161.2 (d, *J* = 242 Hz), 139.2 (d, *J* = 2.3 Hz), 139.0 (d, *J* = 2.6 Hz), 136.0, 135.2 (d, *J* = 9.2 Hz), 134.8 (d, *J* = 1.5 Hz), 132.0 (d, *J* = 3.8 Hz), 131.1, 129.2, 128.5, 128.4, 118.4 (d, *J* = 8.8 Hz), 114.2 (d, *J* = 23.3 Hz), 110.0 (d, *J* = 25.6 Hz), 32.8, 32.7, 31.1 ppm. HRMS [TOF MS ES+]: m/z $[M + H]^+$ calcd. for $C_{19}H_{16}FS_2$ 327.0672, found 327.0679 (2.14 ppm). **2f-(*Z*)**: 7.45–7.37 (m, 6H), 7.24–7.22 (m, 1H), 7.12–7.10 (m, 1H), 6.99–6.95 (m, 1H), 3.66 (t, *J* = 6.0 Hz, 2H), 3.33 (t, *J* = 6.0 Hz, 2H), 2.25–2.20 (m, 2H) ppm. $^{13}C\{^1H\}$ NMR (126 MHz, $CDCl_3$, APT) δ 161.8 (d, *J* = 243 Hz), 138.8 (d, *J* = 2.6 Hz), 138.7 (d, *J* = 8.6 Hz), 137.4 (d, *J* = 1.5 Hz), 136.7 (d, *J* = 2.3 Hz), 136.0, 130.2, 128.3, 127.9, 126.6, 126.0 (d, *J* = 3.8 Hz), 118.1 (d, *J* = 8.5 Hz), 113.8 (d, *J* = 23.3 Hz), 105.9 (d, *J* = 24.6 Hz), 34.0, 31.1, 30.3 ppm. HRMS [TOF MS ES+]: m/z $[M + H]^+$ calcd. for $C_{19}H_{16}FS_2$ 327.0672, found 327.0676 (1.22 ppm).

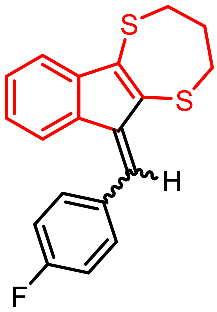

(*E*)/(*Z*)-10-(4-Fluorobenzylidene)-3,4-dihydro-2*H*,10*H*-indeno[1,2-b][1,4]dithiepine (2g): The reaction was performed following General Procedure D with propargyl alcohol (**1g**) (0.5 mmol, 172 mg) and $Ga(OTf)_3$ (0.025 mmol, 13.0 mg) in anhydrous toluene (3.0 mL). The reaction was stirred for 10 min at 110 °C in an oil bath. The workup was performed following the general procedure. The crude material was purified by flash chromatography on

silica gel using 60:1 hexanes/ethyl acetate as eluent to yield the product **2g** (137 mg, 84%, red solid, mp: 88-89 °C) as a mixture of *E*/*Z* isomers. Isomers are partially separated by flash chromatography on silica gel using hexanes as eluent. **2g-(*E*)**: ^{1}H NMR (500 MHz, $CDCl_3$) δ 7.51−7.49 (m, 2H), 7.30 (d, *J* = 7.7 Hz, 1 H), 7.28 (s, 1H), 7.23-7.20 (m, 2H), 7.12 (t, *J* = 8.5 Hz, 2H), 6.97−6.92 (m, 1H), 3.48 (t, *J* = 5.9 Hz, 2H), 3.42 (t, *J* = 6.0 Hz, 2H), 2.38 (t, *J* = 6.0 Hz, 2H) ppm. ^{13}C{^{1}H} NMR (126 MHz, $CDCl_3$, APT) δ 162.5 (d, *J* = 248 Hz), 143.2, 139.9 (d, *J* = 1.0 Hz), 135.5, 133.4, 132.5 (d, *J* = 3.5 Hz), 132.2, 131.2 (d, *J* = 8.0 Hz), 128.7, 128.0, 125.0, 121.9, 118.0, 115.4 (d, *J* = 21.5 Hz), 32.7, 32.6, 31.0 ppm. HRMS [TOF MS ES+]: m/z $[M + H]^+$ calcd. for $C_{19}H_{16}FS_2$ 327.0672, found 327.0673 (0.31 ppm). **2g-(*Z*):** ^{1}H NMR (500 MHz, $CDCl_3$) δ 7.55−7.52 (m, 2H), 7.43–7.40 (m, 3H), 7.30–7.28 (m, 1H), 7.23–7.20 (m, 2H), 7.11 (t, *J* = 8.7 Hz, 2H), 3.67 (t, *J* = 6.0 Hz, 2H), 3.37 (t, *J* = 6.1 Hz, 2H), 2.24 (t, *J* = 6.0 Hz, 2H). ^{13}C{^{1}H} NMR (126 MHz, $CDCl_3$, APT) δ 162.7 (d, *J* = 249 Hz), 140.7, 139.7 (d, *J* = 1.0 Hz), 138.2, 136.6, 132.4 (d, *J* = 3.4 Hz), 132.0 (d, *J* = 8.3 Hz), 127.6, 126.0, 125.3, 124.5, 117.9, 117.5, 115.0 (d, *J* = 21.6 Hz), 34.0, 31.1, 30.3 ppm. HRMS [TOF MS ES+]: m/z $[M + H]^+$ calcd. for $C_{19}H_{16}FS_2$ 327.0672, found 327.0674 (0.61 ppm).

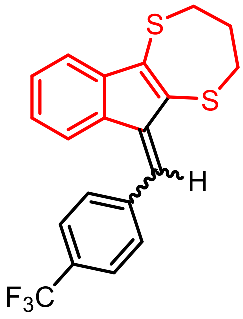

(*E*)/(*Z*)-10-(4-(Trifluoromethyl)benzylidene)-3,4-dihydro-2*H*,10*H*-indeno[1,2-*b*][1,4]dithiepine (**2h**): The reaction was performed following General Procedure D with propargyl alcohol (**1h**) (0.25 mmol, 102 mg) and $Ga(OTf)_3$ (0.0125 mmol, 6.5 mg) in anhydrous toluene (2.0 mL). The reaction was stirred for 15 min at 110 °C in an oil bath. The workup was performed following the general procedure. The crude material was purified by flash chromatography on silica gel using 60:1 hexanes/ethyl acetate as eluent to yield the product **2g** (92 mg, 98%, red solid, mp: 92-93 °C) as a mixture of *E*/*Z* isomers. Isomers are partially separated by flash chromatography on silica gel using hexanes as eluent. **2h-(*E*)**: ^{1}H-NMR (500 MHz, $CDCl_3$) δ 7.69−7.68 (m, 2H, AA'BB'$_{system}$, $\delta_{AA'}$), 7.64−7.62 (m, 2H, AA'BB'$_{system}$, $\delta_{BB'}$), 7.28 (s, 1H), 7.24−7.19 (m, 3H), 6.95 (dt, *J* = 2.0, 7.0 Hz, 2H), 3.51 (t, *J* = 6.0 Hz, 2H), 3.44 (t, *J* = 6.0 Hz, 2H), 2.39 (p, *J* = 6.0 Hz, 2H) ppm. ^{13}C{^{1}H} NMR (126 MHz, $CDCl_3$, APT) δ 143.3, 141.0, 140.4 (q, *J* = 1.5 Hz), 136.4, 133.2, 131.8, 129.9 (q, *J* = 32 Hz), 129.6, 128.4, 127.5, 125.31 (q, *J* = 3.8 Hz), 125.2, 124.1 (q, *J* =

272 Hz), 121.9, 118.1, 32.8, 32.5, 30.1 ppm. HRMS [TOF MS ES+]: m/z $[M + H]^+$ calcd. for $C_{20}H_{16}F_3S_2$ 377.0640, found 377.0632 (–2.12 ppm). **2h-(*Z*):** ^{1}H-NMR (500 MHz, $CDCl_3$) δ 7.66–7.64 (m, 2H, AA'BB'$_{system}$, $\delta_{AA'}$), 7.54–7.51 (m, 3H), 7.38 (s, 1H), 7.31–7.28 (m, 2H), 7.23–7.19 (m, 2H), 3.66 (t, *J* = 6.0 Hz, 2H), 3.35 (t, *J* = 6.0 Hz, 2H), 2.23 (p, *J* = 6.0 Hz, 2H) ppm. ^{13}C{^{1}H} NMR (126 MHz, $CDCl_3$, APT) δ 141.1, 140.9, 140.1 (q, *J* = 1.5 Hz), 139.5, 136.4, 130.3, 129.7 (q, *J* = 32 Hz), 128.0, 125.9, 125.5, 124.7 (q, *J* = 3.8 Hz), 124.2 (q, *J* = 272 Hz), 123.5, 118.0, 117.6, 34.0, 31.0, 30.2 ppm. HRMS [TOF MS ES+]: m/z $[M + H]^+$ calcd. for $C_{20}H_{16}F_3S_2$ 377.0640, found 377.0650 (2.65 ppm).

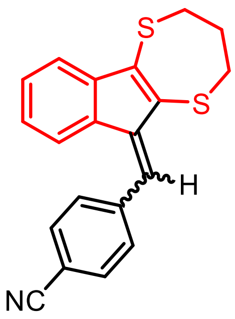

(*E*)/(*Z*)-4-((3,4-Dihydro-2*H*,10*H*-indeno[1,2-*b*][1,4]dithiepin-10-ylidene)methyl)benzonitrile (**2i**): The reaction was performed following General Procedure D with propargyl alcohol (**1i**) (0.50 mmol, 176 mg) and $Ga(OTf)_3$ (0.025 mmol, 13 mg) in anhydrous toluene (3.0 mL). The reaction was stirred for 10 min at 110 °C in an oil bath. The workup was performed following the general procedure. The crude material was purified by flash chromatography on silica gel using 60:1 hexanes/ethyl acetate as eluent to yield the product **2i** (133 mg, 80%, dark red solid, mp: 118-119 °C) as a mixture of *E*/*Z* isomers. **2i-(*E*)/(*Z*):** ^{1}H NMR (500 MHz, $CDCl_3$) δ 7.70–7.68 (m, 2H, AA'BB'$_{system}$, $\delta_{AA'}$), 7.66–7.64 (m, 2H, AA'BB'$_{system}$, $\delta_{AA'}$), 7.61–7.59 (m, 2H, AA'BB'$_{system}$, $\delta_{BB'}$), 7.51–7.49 (m, 3H), 7.32–7.27 (m, 2H), 7.24–7.17 (m, 6H), 6.94 (t, *J* = 7.5 Hz, 1H), 3.65 (t, *J* = 6.0 Hz, 2H), 3.51 (t, *J* = 6.0 Hz, 2H), 3.44 (t, *J* = 6.1 Hz, 2H), 2.39 (t, *J* = 6.0 Hz, 2H), 2.23 (t, *J* = 6.0 Hz, 2H) ppm. ^{13}C{^{1}H} NMR (126 MHz, $CDCl_3$, APT) δ 143.3, 141.6, 141.4 (2 × C_{quat}), 141.2, 140.7, 140.2, 136.9, 136.2, 133.0, 132.0, 131.4, 130.6, 130.0, 128.6, 128.1, 126.7, 125.6, 125.4, 125.2, 122.7, 121.8, 118.8, 118.6, 118.1, 118.0, 117.6, 111.4, 111.1, 34.0, 32.8, 32.4, 30.9, 30.8, 30.1 ppm. HRMS [TOF MS ES+]: m/z $[M + H]^+$ calcd. for $C_{20}H_{16}NS_2$ 334.0719, found 334.0727 (2.39 ppm).

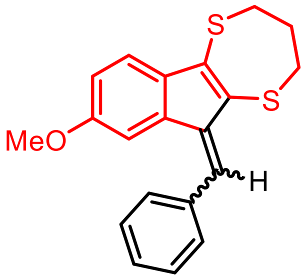

(*E*)/(*Z*)-10-Benzylidene-8-methoxy-3,4-dihydro-2*H*,10*H*-indeno[1,2-*b*][1,4]dithiepine (**2j**): The reaction was performed following General Procedure D with propargyl alcohol (**1j**) (0.5 mmol, 178 mg) and Ga(OTf)$_3$ (0.025 mmol, 13 mg) in anhydrous toluene (3.0 mL). The reaction was stirred for 5 min at 110 °C in an oil bath. The workup was performed following the general procedure. The crude material was purified by flash chromatography on silica gel using 60:1 hexanes/ethyl acetate as eluent to yield the product **2j** (124 mg, 73%, red oil) as a mixture of *E*/*Z* isomers. Isomers are partially separated by flash chromatography on silica gel using hexanes as eluent. **2j-(*E*)**: ^{1}H NMR (500 MHz, CDCl$_3$) δ 7.54 (d, *J* = 7.1 Hz, 2H), 7.44 (t, *J* = 7.3 Hz, 2H), 7.38–7.34 (m, 2H), 7.09 (d, *J* = 8.3 Hz, 1H), 6.95 (d, *J* = 2.4 Hz, 1H), 6.74 (dd, *J* = 2.4 Hz, 8.3 Hz, 1H), 3.60 (s, 3H), 3.45 (t, *J* = 5.9 Hz, 2H), 3.88 (t, *J* = 5.9 Hz, 2H), 2.38 (t, *J* = 5.9 Hz, 2H). ^{13}C{^{1}H} NMR (126 MHz, CDCl$_3$, APT) δ 157.9, 139.7, 136.5, 136.4, 135.7, 135.1, 130.3, 129.7, 129.1, 128.3, 128.1, 118.3, 112.6, 109.2, 55.2, 32.9, 32.8, 31.0 ppm. HRMS [TOF MS ES+]: m/z [M + H]$^+$ calcd. for $C_{20}H_{19}OS_2$ 339.0872, found 339.0876 (1.18 ppm). **2j-(*Z*):** ^{1}H NMR (500 MHz, CDCl$_3$) δ 7.46–7.35 (m, 6H), 7.14 (d, *J* = 2.5 Hz, 1H), 7.09 (d, *J* = 8.3 Hz, 1H), 6.62 (dd, *J* = 2.5 Hz, 8.3 Hz, 1H), 3.87 (s, 3H), 3.65 (t, *J* = 5.9 Hz, 2H), 3.33 (t, *J* = 6.1 Hz, 2H), 2.22 (p, *J* = 6.1 Hz, 2H). ^{13}C{^{1}H} NMR (126 MHz, CDCl$_3$, APT) δ 158.5, 139.5, 138.5, 138.2, 136.4, 134.2, 130.2, 128.0, 127.8, 125.2, 124.3, 118.0, 112.3, 105.0, 55.6, 34.1, 31.2, 30.1 ppm. HRMS [TOF MS ES+]: m/z [M + H]$^+$ calcd. for $C_{20}H_{19}OS_2$ 339.0872, found 339.0877 (1.47 ppm).

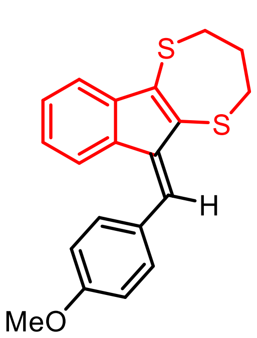

(*E*)-10-(4-methoxybenzylidene)-3,4-dihydro-2*H*,10*H*-indeno[1,2b][1,4]-dithiepine 2l-(*E*):The reaction was performed following General Procedure D with propargyl alcohol (**1l**) (0.5 mmol, 178 mg) and Ga(OTf)$_3$ (0.025 mmol, 13 mg) in anhydrous toluene (3.0 mL). The reaction was stirred for 5 min at 110 °C in an oil bath. The workup was performed following the general procedure. The crude material was purified by flash chromatography on silica gel using hexanes as eluent to yield the product **2l-(*E*)** (46 mg, 27%, red solid, mp: 105-106 °C). **2l-(*E*)**: ^{1}H-NMR (500 MHz, CDCl$_3$) δ 7.50–7.53 (m, 3H), 7.33 (s, 1H), 7.20–7.24 (m, 2H), 6.95–

6.98 (m, 3H), 3.88 (s, 3H), 3.45 (t, *J* = 5.9 Hz, 2H), 3.39 (t, *J* = 5.9 Hz, 2H), 2.38 (p, *J* = 5.9 Hz, 2H) ppm. $^{13}C\{^{1}H\}$ NMR (126 MHz, $CDCl_3$, APT) δ 159.7, 143.1, 138.6, 134.6, 133.6, 132.8, 131.1, 130.3, 128.7, 127.7, 124.8, 121.9, 117.9, 113.8, 55.3, 32.8, 32.7, 31.1 ppm. HRMS [TOF MS ES+]: m/z $[M + H]^+$ calcd. for $C_{20}H_{19}OS_2$ 339.0872, found 339.0872 (0 ppm).

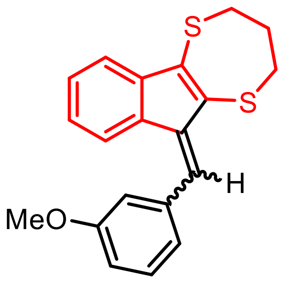

(*E*)/(*Z*)-10-(3-Methoxybenzylidene)-3,4-dihydro-2*H*,10*H*-indeno[1,2-*b*][1,4]dithiepine (2m): The reaction was performed following General Procedure D with propargyl alcohol (**1m**) (0.5 mmol, 178 mg) and $Ga(OTf)_3$ (0.025 mmol, 13 mg) in anhydrous toluene (3.0 mL). The reaction was stirred for 15 min at 110 °C in an oil bath. The workup was performed following the general procedure. The crude material was purified by flash chromatography on silica gel using 60:1 hexanes/ethyl acetate as eluent to yield the product **2m** (134 mg, 79%, red oil) as a mixture of *E*/*Z* isomers. Isomers are partially separated by flash chromatography on silica gel using hexanes as eluent. **2m-(*E*)**: ^{1}H-NMR (500 MHz, $CDCl_3$) δ 7.41 (d, *J* = 7.6 Hz, 1H), 7.33–7.36 (m, 2H), 7.21 (d, *J* = 4.0 Hz, 2H), 7.13 (d, *J* = 7.7 Hz, 1H), 7.08 (s, 1H), 6.92–6.98 (m, 2H), 3.82 (s, 3H), 3.49 (t, *J* = 6.1 Hz, 2H), 3.42 (t, *J* = 6.0 Hz, 2H), 2.39 (p, *J* = 6.1 Hz, 2H) ppm. $^{13}C\{^{1}H\}$ NMR (126 MHz, $CDCl_3$, APT) δ 159.4, 143.2, 139.8, 137.8, 135.4, 133.5, 132.3, 129.8, 129.4, 127.9, 125.0, 122.3, 121.8, 117.9, 114.2, 114.1, 55.2, 32.7, 32.6, 31.0 ppm. HRMS [TOF MS ES+]: m/z $[M + H]^+$ calcd. for $C_{20}H_{19}OS_2$ 339.0872, found 339.0876 (0 ppm). **2m-(*Z*):** ^{1}H-NMR (500 MHz, $CDCl_3$) δ 7.54–7.53 (m, 1H), 7.45 (s, 1H), 7.33 (t, *J* = 8.0 Hz, 1H), 7.30–7.27 (m, 1H), 7.22–7.19 (m, 2H), 7.05 (d, *J* = 7.5 Hz, 1H), 7.01 (s, 1H), 6.92 (dd, *J* = 2.6, 8.2 Hz, 1H), 3.88 (s, 3H), 3.67 (t, *J* = 6.0 Hz, 2H), 3.36 (t, *J* = 5.9 Hz, 2H), 2.23 (p, *J* = 6.0 Hz, 2H) ppm. $^{13}C\{^{1}H\}$ NMR (126 MHz, $CDCl_3$, APT) δ 158.9, 140.7, 139.7, 138.1, 137.7, 136.7, 128.8, 127.5, 126.3, 125.6, 125.2, 122.8, 117.9, 117.4, 115.3, 114.1, 55.2, 34.1, 31.0, 30.1 ppm. HRMS [TOF MS ES+]: m/z $[M + H]^+$ calcd. for $C_{20}H_{19}OS_2$ 339.0872, found 339.0874 (0.59 ppm).

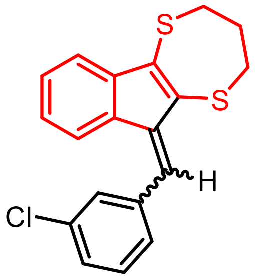

(*E*)/(*Z*)-10-(3-Chlorobenzylidene)-3,4-dihydro-2*H*,10*H*-indeno[1,2-*b*][1,4]dithiepine (**2n**): The reaction was performed following General Procedure D with propargyl alcohol (**1n**) (0.5 mmol, 181 mg) and $Ga(OTf)_3$ (0.025 mmol, 13 mg) in anhydrous toluene (3.0 mL). The reaction was stirred for 10 min at 110 °C in an oil bath. The workup was performed following the general procedure. The crude material was purified by flash chromatography on silica gel using 60:1 hexanes/ethyl acetate as eluent to yield the product **2n** (120 mg, 70%, red solid, mp: 98-99 °C) as a mixture of *E*/*Z* isomers. Isomers are partially separated by flash chromatography on silica gel using hexanes as eluent. **2n-(*E*)**: 7.52 (s, 1H), 7.42–7.39 (m, 1H), 7.38–7.35 (m, 2H), 7.28–7.20 (m, 4H), 6.98–6.94 (m, 1H), 3.50 (t, *J* = 5.9 Hz, 2H), 3.43 (t, *J* = 5.9 Hz, 2H), 2.39 (p, *J* = 5.9 Hz, 2H) ppm. $^{13}C\{^1H\}$ NMR (126 MHz, $CDCl_3$, APT) δ 143.3, 140.6, 138.4, 136.1, 134.3, 133.3, 131.9, 129.6, 129.2, 128.2, 128.1, 127.8, 127.5, 125.2, 122.0, 118.0, 32.8, 32.6, 30.9 ppm. HRMS [TOF MS ES+]: m/z $[M + H]^+$ calcd. for $C_{19}H_{16}ClS_2$ 343.0376, found 343.0382 (1.75 ppm). **2n-(*Z*)**: ^1H-NMR (500 MHz, $CDCl_3$): 7.50–7.52 (m, 1H), 7.45 (s, 1H), 7.28–7.35 (m, 5H), 7.20–7.23 (m, 2H), 3.67 (t, *J* = 6.0 Hz, 2H), 3.36 (t, *J* = 6.0 Hz, 2H), 2.24 (p, *J* = 6.0 Hz, 2H) ppm. $^{13}C\{^1H\}$ NMR (126 MHz, $CDCl_3$, APT) δ 140.8, 140.5, 139.0, 138.2, 136.4, 133.6, 130.0, 129.0, 128.3, 127.9, 127.7, 126.0, 125.3, 123.7, 117.9, 117.5, 34.0, 31.0, 30.2 ppm. HRMS [TOF MS ES+]: m/z $[M + H]^+$ calcd. for $C_{19}H_{16}ClS_2$ 343.0376, found 343.0372 (–1.17 ppm).

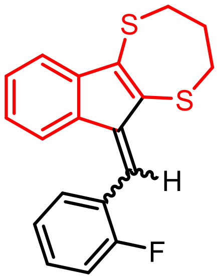

(*E*)/(*Z*)-10-(2-Fluorobenzylidene)-3,4-dihydro-2*H*,10*H*-indeno[1,2-*b*][1,4]dithiepine (**2o**): The reaction was performed following General Procedure D with propargyl alcohol (**1o**) (0.25 mmol, 87 mg) and $Ga(OTf)_3$ (0.125 mmol, 6.5 mg) in anhydrous toluene (2.0 mL). The reaction was stirred for 15 min at 110 °C in an oil bath. The workup was performed following the general procedure. The crude material was purified by flash chromatography on silica gel using 60:1 hexanes/ethyl acetate as eluent to yield the product **2o** (74 mg, 90%, red solid, mp: 79-80 °C) as a mixture of *E*/*Z* isomers. Isomers are partially separated by flash chromatography on silica gel using hexanes as eluent. **2o-(*E*)**: ^1H NMR (500 MHz, $CDCl_3$) δ 7.62 (t, *J* = 7.4 Hz, 1H), 7.40 (q, *J* = 7.0 Hz,

1H), 7.34–7.28 (m, 2H), 7.26–7.19 (m, 4H), 7.02–6.99 (m, 1H), 3.53 (t, *J* = 5.9 Hz, 2H), 3.46 (t, *J* = 6.0 Hz, 2H), 2.39 (t, *J* = 6.0 Hz, 2H) ppm. $^{13}C\{^1H\}$ NMR (126 MHz, $CDCl_3$, APT) δ 160.2 (d, *J* = 249 Hz), 143.2, 141.1, 136.0, 133.4, 132.0, 131.4 (d, *J* = 2.8 Hz), 130.1 (d, *J* = 8.1 Hz), 128.1, 125.1, 124.2 (d, *J* = 15.5 Hz), 123.7 (d, *J* = 3.7 Hz), 122.1 (d, *J* = 2.6 Hz), 121.8, 117.9, 115.7 (d, *J* = 21.5 Hz), 32.7, 32.5, 30.9 ppm. HRMS [TOF MS ES+]: m/z $[M + H]^+$ calcd. for $C_{19}H_{16}FS_2$ 327.0672, found 327.0675 (0.92 ppm). **2o-(*Z*):** ^1H NMR (500 MHz, $CDCl_3$) δ 7.57 (d, *J* = 7.4 Hz, 1H), 7.43 (t, *J* = 7.5 Hz, 1H), 7.38–7.35 (m, 2H), 7.29 (q, *J* = 7.4 Hz, 1H), 7.24–7.18 (m, 3H), 7.15–7.11 (m, 1H), 3.66 (t, *J* = 6.0 Hz, 2H), 3.37 (t, *J* = 6.0 Hz, 2H), 2.44 (t, *J* = 6.0 Hz, 2H) ppm. $^{13}C\{^1H\}$ NMR (126 MHz, $CDCl_3$, APT) δ 160.3 (d, *J* = 248 Hz), 141.2, 140.8, 138.7, 136.4, 133.1 (d, *J* = 2.7 Hz), 130.0 (d, *J* = 8.1 Hz), 127.8, 126.4, 125.4, 124.4 (d, *J* = 15.4 Hz), 123.3 (d, *J* = 3.5 Hz), 118.3 (d, *J* = 2.8 Hz), 118.2, 117.5, 115.1 (d, *J* = 21.5 Hz), 33.9, 31.1, 30.2 ppm. HRMS [TOF MS ES+]: m/z $[M + H]^+$ calcd. for $C_{19}H_{16}FS_2$ 327.0672, found 327.0674 (0.61 ppm).

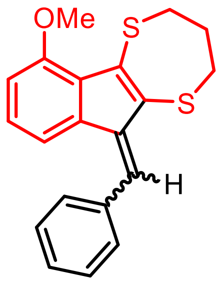

(*E*)/(*Z*)-10-benzylidene-6-methoxy-3,4-dihydro-2H,10H-indeno[1,2-b][1,4]dithiepine (**2p**): The reaction was performed following General Procedure D with propargyl alcohol (**1p**) (0.35 mmol, 124 mg) and $Ga(OTf)_3$ (0.018 mmol, 9.0 mg) in anhydrous toluene (2.5 mL). The reaction was stirred for 30 min at 110 °C in an oil bath. The workup was performed following the general procedure. The crude material was purified by flash chromatography on silica gel using 60:1 hexanes/ethyl acetate as eluent to yield the product **2p** (61.0 mg, 52%, red solid, mp: 99-100 °C) as a mixture of *E*/*Z* isomers. Isomers are partially separated by flash chromatography on silica gel using hexanes as eluent. **2p-(*E*)**: ^1H-NMR (500 MHz, $CDCl_3$) δ 7.50–7.49 (m, 2H), 7.42–7.36 (m, 2H), 7.35–7.33 (m, 1H), 7.25 (s, 1H), 6.98 (d, *J* = 8.0 Hz, 1H, AB_{system}, δ_A), 6.85 (t, *J* = 8.0 Hz, 1H), 6.73 (d, *J* = 8.0 Hz, 1H, AB_{system}, δ_B), 3.86 (s, 3H), 3.66 (t, *J* = 6.1 Hz, 2H), 3.55 (t, *J* = 6.0 Hz, 2H), 2.31 (p, *J* = 6.0 Hz, 2H) ppm. $^{13}C\{^1H\}$ NMR (126 MHz, $CDCl_3$, APT) δ 152.3, 140.0, 136.7, 135.9, 133.7, 130.5, 129.3, 129.2, 128.3, 127.9, 127.8, 126.2, 55.9, 33.1, 31.6, 31.0 ppm. HRMS [TOF MS ES+]: m/z $[M + H]^+$ calcd. for $C_{20}H_{19}OS_2$ 339.0872, found 339.0871 (–0.29 ppm). **2p-(*Z*):** ^1H-NMR (500 MHz, $CDCl_3$) δ 7.45–7.38 (m, 5H), 7.36–7.33 (m, 1H), 7.20 (d, *J* = 7.6 Hz, 1H, AB_{system}, δ_A), 7.15–

7.12 (m, 1H, AB$_{system}$, δ_B), 6.80 (d, *J* = 8.1 Hz, 1H), 3.90 (s, 3H), 3.70 (t, *J* = 6.1 Hz, 2H), 3.36 (t, *J* = 6.0 Hz, 2H), 2.17 (p, *J* = 6.0 Hz, 2H) ppm. $^{13}C\{^1H\}$ NMR (126 MHz, $CDCl_3$, APT) δ 152.4, 140.0, 139.1, 137.8, 136.6, 130.2, 128.1, 127.9, 127.8, 126.4, 125.4, 123.0, 111.5, 111.1, 55.8, 34.3, 30.6, 30.4 ppm. HRMS [TOF MS ES+]: m/z $[M + H]^+$ calcd. for $C_{20}H_{19}OS_2$ 339.0872, found 339.0874 (0.59 ppm).

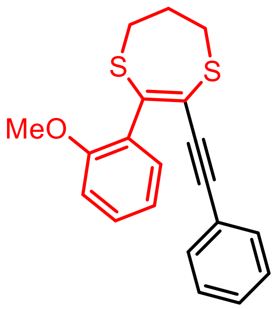

2-(2-Methoxyphenyl)-3-(phenylethynyl)-6,7-dihydro-5*H*-1,4-dithiepine (**2p-IV**): The reaction was performed following General Procedure D with propargyl alcohol (**1p**) (0.5 mmol, 178 mg) and $Ga(OTf)_3$ (0.025 mmol, 13 mg) in anhydrous toluene (3.0 mL). The reaction was stirred for 5 min at 110 °C in an oil bath. The workup was performed following the general procedure. The crude material was purified by flash chromatography on silica gel using 60:1 hexanes/ethyl acetate as eluent to yield the product **2p** (55.0 mg, 32%, red oil) as a mixture of *E*/*Z* isomers and **2p-IV** (65.0 mg, 38%, yellowish oil)**. 2p-IV:** ^1H-NMR (500 MHz, $CDCl_3$) δ 7.33–7.29 (m, 2H), 7.21–7.16 (m, 3H), 7.05–7.02 (m, 2H), 6.97 (dt, *J* = 1.2, 8.0 Hz, 1H), 6.93 (d, *J* = 8.0 Hz, 1H), 3.86 (s, 3H), 3.49 (bs, 4H), 2.23 (bs, 2H) ppm. $^{13}C\{^1H\}$ NMR (126 MHz, $CDCl_3$, APT) δ 156.8, 141.7, 131.0, 130.7, 130.0, 129.6, 128.0, 127.8, 123.1, 120.2, 115.2, 111.3, 93.2, 88.8, 55.9, 32.8, 32.5, 30.2 ppm. HRMS [TOF MS ES+]: m/z $[M + H]^+$ calcd. for $C_{20}H_{19}OS_2$ 339.0872, found 339.0868 (–1.18 ppm).

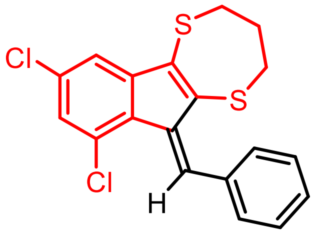

(*Z*)-10-Benzylidene-7,9-dichloro-3,4-dihydro-2*H*,10*H*-indeno[1,2-*b*][1,4]dithiepine 2r-(*Z*) : The reaction was performed following General Procedure D with propargyl alcohol (**1r**) (0.5 mmol, 198 mg) and $Ga(OTf)_3$ (0.025 mmol, 13 mg) in anhydrous toluene (3.0 mL). The reaction was stirred for 30 min at 110 °C in an oil bath. The workup was performed following the general procedure. The crude material was purified by flash chromatography on silica gel using hexanes as eluent to yield the product **2r-(*Z*)** (113 mg, 60%, red solid, mp: 114-115 °C) as the only isomer. **2r-(*Z*)**: ^1H-NMR (500 MHz, $CDCl_3$) δ 8.43 (s, 1H), 7.43–7.37 (m, 5H), 7.17 (d, *J* = 2.0 Hz, 1H, AB$_{system}$, δ_A), 7.11 (d, *J* = 2.0 Hz, 1H, AB$_{system}$, δ_B), 3.62 (t, *J* = 6.0 Hz, 2H), 3.30 (t,

J = 6.0 Hz, 2H), 21.9 (p, *J* = 6.0 Hz, 2H) ppm. $^{13}C\{^1H\}$ NMR (126 MHz, $CDCl_3$, APT) δ 144.4, 138.7, 136.7, 134.9, 133.0, 132.9, 130.4, 130.0, 129.4, 128.3, 127.9, 127.5, 126.8, 116.7, 34.0, 30.8, 30.4 ppm. HRMS [TOF MS ES+]: m/z $[M + H]^+$ calcd. for $C_{19}H_{15}Cl_2S_2$ 376.9987, found 376.9985 (–0.53 ppm).

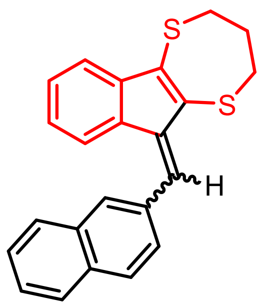

(*E*)/(*Z*)-10-(Naphthalen-2-ylmethylene)-3,4-dihydro-2*H*,10*H*-indeno[1,2-*b*][1,4]dithiepine (**2q**): The reaction was performed following General Procedure D with propargyl alcohol (**1q**) (0.50 mmol, 188 mg) and $Ga(OTf)_3$ (0.025 mmol, 13.0 mg) in anhydrous toluene (3.0 mL). The reaction was stirred for 10 min at 110 °C in an oil bath. The workup was performed following the general procedure. The crude material was purified by flash chromatography on silica gel using 60:1 hexanes/ethyl acetate as eluent to yield the product **2q** as a mixture of *E*/*Z* isomers (134 mg, 75%, red solid, mp: 121-122 °C). Isomers are partially separated by flash chromatography on silica gel using hexanes as eluent. **2q-(*E*)**: ^1H NMR (500 MHz, $CDCl_3$) δ 8.05 (s, 1H), 7.92 (d, *J* = 8.6 Hz, 2H), 7.86 (d, *J* = 5.9 Hz, 1H), 7.68 (d, *J* = 8.5 Hz, 1H), 7.57−7.55 (m, 3H), 7.45 (d, *J* = 7.5 Hz, 1H), 7.28–7.23 (m, 2H), 6.93 (t, *J* = 8.4 Hz, 1H), 3.52 (t, *J* = 5.9 Hz, 2H), 3.47 (t, *J* = 6.0 Hz, 2H), 2.42 (p, *J* = 5.9 Hz, 2H) ppm. $^{13}C\{^1H\}$ NMR (126 MHz, $CDCl_3$, APT) δ 143.2, 139.9, 135.4, 134.0, 133.5, 133.1, 132.9, 132.4, 130.0, 128.8, 128.1, 128.0, 127.9, 127.7, 127.3, 126.5, 126.4, 125.0, 122.0, 117.9, 32.7, 32.6, 31.0 ppm. HRMS [TOF MS ES+]: m/z $[M + H]^+$ calcd. for $C_{23}H_{19}S_2$ 359.0923, found 359.0924 (0.28 ppm). **2q-(*Z*)**: ^1H NMR (500 MHz, $CDCl_3$) δ 7.91–7.86 (m, 4H), 7.61 (s, 1H), 7.58 (t, *J* = 8.0 Hz, 2H), 7.54–7.50 (m, 2H), 7.31–7.29 (m, 1H), 7.24–7.21 (m, 2H), 3.68 (t, *J* = 6.0 Hz, 2H), 3.29 (t, *J* = 6.0 Hz, 2H), 2.20 (p, *J* = 6.0 Hz, 2H) ppm. $^{13}C\{^1H\}$ NMR (126 MHz, $CDCl_3$, APT) δ 140.7, 140.0, 138.1, 136.7, 133.9, 132.9, 132.8, 130.1, 128.3, 127.7, 127.6, 127.5, 127.3, 126.5, 126.4, 126.3, 125.8, 125.2, 117.9, 117.5, 34.0, 31.0, 30.2 ppm. HRMS [TOF MS ES+]: m/z $[M + H]^+$ calcd. for $C_{23}H_{19}S_2$ 359.0923, found 359.0925 (0.56 ppm).

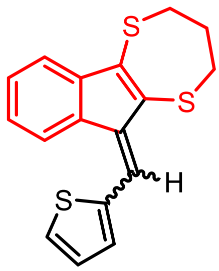

(*E*)/(*Z*)-10-(Thiophen-2-ylmethylene)-3,4-dihydro-2*H*,10*H*-indeno[1,2-*b*][1,4]dithiepine (**2s**): The reaction was performed following General Procedure D with propargyl alcohol (**1s**) (0.5 mmol, 166 mg) and Ga(OTf)$_3$ (0.025 mmol, 13 mg) in anhydrous toluene (3.0 mL). The reaction was stirred for 5 min at 110 °C in an oil bath. The workup was performed following the general procedure. The crude material was purified by flash chromatography on silica gel using hexanes as eluent to yield the product **2s-(*E*)** (26 mg, 17%, red oil). **2s-(*E*)**: ^{1}H NMR (500 MHz, CDCl$_3$) δ 7.96 (d, *J* = 7.6 Hz, 1H), 7.47 (dd, *J* = 1.1, 5.1 Hz, 1H), 7.41 (td, *J* = 1.1, 3.6 Hz, 1H), 7.34 (bs, 1H), 7.28–7.24 (m, 2H), 7.13 (dd, *J* = 3.6, 5.1 Hz, 1H), 7.09–7.06 (m, 1H), 3.46–3.44 (m, 2H), 3.39–3.36 (m, 2H), 2.41–2.37 (m, 2H) ppm. ^{13}C{^{1}H} NMR (126 MHz, CDCl$_3$, APT) δ 143.1, 139.3, 138.9, 135.5, 133.2, 132.8, 130.0, 128.1, 128.0, 127.6, 125.2, 122.1, 121.9, 118.1, 32.8, 32.7, 31.0 ppm. HRMS [TOF MS ES+]: m/z [M + H]$^+$ calcd. for $C_{17}H_{15}S_3$ 315.0330, found 315.0334 (1.27 ppm).

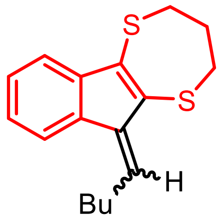

(*E*)/(*Z*)-10-Pentylidene-3,4-dihydro-2*H*,10*H*-indeno[1,2-*b*][1,4]dithiepine (**2t**): The reaction was performed following General Procedure D with propargyl alcohol (**1t**) (0.50 mmol, 153 mg) and Ga(OTf)$_3$ (0.025 mmol, 13 mg) in anhydrous toluene (3.0 mL). The reaction was stirred for 10 min at 110 °C in an oil bath. The workup was performed following the general procedure. The crude material was purified by flash chromatography on silica gel using 60:1 hexanes/ethyl acetate as eluent to yield the product **2t** (111 mg, 77%, yellow oil) as a mixture of *E*/*Z* isomers. **2t-(*E*)/(*Z*):** ^{1}H NMR (500 MHz, CDCl$_3$) δ 7.67 (d, *J* = 7.5 Hz, 1H, *E*), 7.45 (d, *J* = 7.5 Hz, 1H, *Z*), 7.31–7.24 [m, 2H (*E*), 1H (*Z*)], 7.22–7.16 [m, 1H (*E*), 2H (*Z*)], 6.55 (t, *J* = 8.0 Hz, 1H, *Z*), 6.46 (t, *J* = 7.5 Hz, 1H, *E*), 3.59–3.56 (m, 4H, *Z*), 3.42 (t, *J* = 6.1 Hz, 2H, *E*), 3.37 (t, *J* = 6.0 Hz, 2H, *E*), 2.88 (q, *J* = 7.6 Hz, 2H, *Z*), 2.80 (q, *J* = 7.6 Hz, 2H, *E*), 2.38–2.30 [m, 2H (*E*), 2H (*Z*)], 1.70–1.58 [m, 2H (*E*), 2H (*Z*)], 1.55–1.45 [m, 2H (*E*), 2H (*Z*)], 1.03–1.00 [m, 3H (*E*), 3H (*Z*)] ppm. ^{13}C{^{1}H} NMR (126 MHz, APT) δ 143.0, 140.6, 138.8, 137.7, 136.5, 135.6, 134.5, 134.3, 133.3, 132.6, 131.6, 129.0, 127.1, 126.7, 124.9, 124.6, 122.5, 118.0, 117.2 (2 × CH), 33.0, 32.9, 32.4, 32.1, 31.9, 31.5, 31.2, 30.8, 29.1, 29.0, 22.6, 22.5, 13.9 (2 × CH_3) ppm. HRMS [TOF MS ES+]: m/z [M + H]$^+$ calcd. for $C_{17}H_{21}S_2$ 289.1079, found 289.1076 (−1.04 ppm).

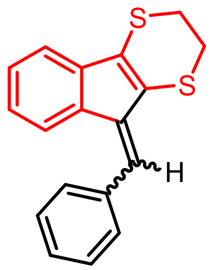

(*E*)/(*Z*)-9-Benzylidene-2,3-dihydro-9*H*-indeno[1,2-*b*][1,4]dithiine (4a): The reaction was performed following General Procedure D with propargyl alcohol (**3a**) (0.25 mmol, 78 mg) and Ga(OTf)$_3$ (0.0125 mmol, 6.5 mg) in anhydrous toluene (2.0 mL). The reaction was stirred for 30 min at 110 °C in an oil bath. The workup was performed following the general procedure. The crude material was purified by flash chromatography on silica gel using 60:1 hexanes/ethyl acetate as eluent to yield the product **4a** as a mixture of *E*/*Z* isomers (52 mg, 71%, red oil). Isomers are partially separated by flash chromatography on silica gel using hexanes as eluent. **4a-(*E*)**: ^{1}H-NMR (500 MHz, CDCl$_3$) δ 7.55 (d, *J* = 7.4 Hz, 2H), 7.44 (t, *J* = 7.4 Hz, 2H), 7.40–7.37 (m, 2H), 7.20 (t, *J* = 7.4 Hz, 1H), 7.11 (s, 1H), 7.08 (d, *J* = 7.4 Hz, 1H), 6.93 (t, *J* = 7.5 Hz, 1H), 3.41–3.40 (m, 2H), 3.35–3.34 (m, 2H) ppm. HRMS [TOF MS ES+]: m/z [M + H]$^+$ calcd. for $C_{18}H_{15}S_2$ 295.0610, found 295.0609 (–0.34 ppm). ^{13}C{^{1}H} NMR (126 MHz, CDCl$_3$, APT) δ 142.8, 138.9, 136.3, 133.3, 129.4, 128.3, 128.1, 127.9, 127.4, 127.2, 124.8, 123.5, 122.1, 116.5, 27.0, 26.8 ppm. **4a-(*Z*)**: ^{1}H-NMR (500 MHz, CDCl$_3$) δ 7.57 (d, *J* = 7.4 Hz, 1H), 7.47–7.42 (m, 4H), 7.39–7.35 (m, 2H), 7.29–7.26 (m, 1H), 7.22–7.11 (m, 1H), 7.14 (d, *J* = 7.4 Hz, 1H), 3.35–3.34 (m, 2H), 3.05–3.04 (m, 2H) ppm. ^{13}C{^{1}H} NMR (126 MHz, CDCl$_3$, APT) δ 140.3, 138.6, 136.1, 136.0, 131.5, 129.8, 128.2, 127.9, 127.5, 125.2, 124.9, 120.2, 118.1, 116.3, 27.8, 27.3 ppm. HRMS [TOF MS ES+]: m/z [M + H]$^+$ calcd. for $C_{18}H_{15}S_2$ 295.0610, found 295.0610 (–0.0 ppm) The sample for HRMS analysis is prepared form E/Z mixture of **4a**.

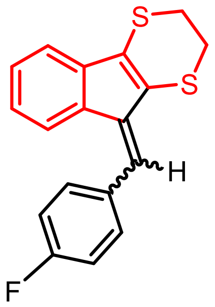

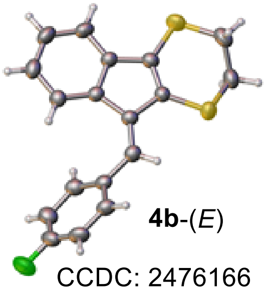

4b-(*E*)

CCDC: 2476166

9-(4-Fluorobenzylidene)-2,3-dihydro-9*H*-indeno[1,2-*b*][1,4]dithiine (**4b**): The reaction was performed following General Procedure D with propargyl alcohol (**3b**) (0.5 mmol, 165 mg) and Ga(OTf)$_3$ (0.025 mmol, 13 mg) in anhydrous toluene (3.0 mL). The reaction was stirred for 30 min at 110 °C in an oil bath. The workup was performed following the general procedure. The crude material was purified by flash chromatography on silica gel using 60:1 hexanes/ethyl acetate as eluent to yield the product **4b** as a mixture of *E*/*Z* isomers (114 mg, 73%, red oil). Isomers are partially separated by flash chromatography on silica gel using hexanes as eluent. **4b-(*E*)**: ^{1}H-NMR (500

MHz, $CDCl_3$) δ 7.51−7.48 (m, 2H), 7.31 (d, *J* = 7.6 Hz, 1H), 7.19 (dt, *J* = 1.1, 7.6 Hz, 1H), 7.14−7.10 (m, 2H), 7.07 (dt, *J* = 7.4 Hz, 1H), 7.01 (s, 1H), 6.93 (dt, *J* = 1.2, 7.6 Hz, 1H), 3.41−3.38 (m, 2H), 3.35−3.33 (m, 2H) ppm. $^{13}C\{^1H\}$ NMR (126 MHz, $CDCl_3$, APT) δ 162.5 (d, *J* = 248.0 Hz), 142.8, 139.1, 133.2, 132.2 (d, *J* = 3.5 Hz), 131.2 (d, *J* = 8.0 Hz), 128.1, 127.6, 125.9, 124.9, 123.4, 121.9, 116.6, 115.4 (d, *J* = 21.6 Hz), 27.0, 26.7 ppm. HRMS [TOF MS ES+]: m/z $[M + H]^+$ calcd. for $C_{18}H_{14}FS_2$ 313.0515, found 313.0520 (1.60 ppm). **4b-(*Z*)**: ^1H-NMR (500 MHz, $CDCl_3$) δ 7.54 (d, *J* = 7.5 Hz, 1H), 7.43−7.41 (m, 2H), 7.38 (s, 1H), 7.29−7.25 (m, 1H), 7.21−7.18 (m, 1H), 7.15−7.10 (m, 3H), 3.34−3.31 (m, 2H), 3.03−3.05 (m, 2H) ppm. $^{13}C\{^1H\}$ NMR (126 MHz, $CDCl_3$, APT) δ 162.6 (d, *J* = 248.0 Hz), 140.3, 138.6 (d, *J* = 1.0 Hz), 136.0, 131.9 (d, *J* = 3.3 Hz), 131.7, 131.5 (d, *J* = 8.3 Hz), 127.6, 125.2, 123.6, 119.9, 118.0, 116.3, 114.9 (d, *J* = 21.5 Hz), 27.6, 27.1 ppm. HRMS [TOF MS ES+]: m/z $[M + H]^+$ calcd. for $C_{18}H_{14}FS_2$ 313.0515, found 313.0513 (–0.64 ppm).

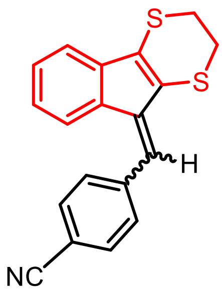

4-((2,3-Dihydro-9*H*-indeno[1,2-*b*][1,4]dithiin-9-ylidene)methyl)benzonitrile (**4c**): The reaction was performed following General Procedure D with propargyl alcohol (**3c**) (0.25 mmol, 84 mg) and $Ga(OTf)_3$ (0.125 mmol, 6.5 mg) in anhydrous toluene (2.0 mL). The reaction was stirred for 50 min at 110 °C in an oil bath. The workup was performed following the general procedure. The crude material was purified by flash chromatography on silica gel using 30:1 hexanes/ethyl acetate as eluent to yield the product **4c** as a mixture of *E*/*Z* isomers (53 mg, 66%, dark red solid, mp: 116-117 °C). **4c-(*E*)/(Z)**: ^1H-NMR (500 MHz, $CDCl_3$) δ 7.71−7.69 (m, 2H, AA'BB'$_{system}$, $δ_{AA'}$), *7.67−7.65 (m, 2H, AA'BB'$_{system}$, $δ_{AA'}$), 7.62−7.61 (m, 2H, AA'BB'$_{system}$, $δ_{BB'}$), *7.54−7.53 (m, 3H), *7.32 (s, 1H), *7.30−7.27 (m, 1H), 7.20−7.19 [m, 2H (*E*), 1 H (*Z*)], *7.12 (d, *J* = 7.4 Hz, 1H), 7.06 (d, *J* = 7.3 Hz, 1H), 6.95−6.91 (m, 2H), 3.42−3.39 (m, 2H), 3.37−3.33 [m, 2H (*E*), 2H (*Z*), 3.07−3.05 (m, 2H) ppm. *Peaks assigned for **4c**-(**Z**). $^{13}C\{^1H\}$ NMR (126 MHz, $CDCl_3$, APT) δ 143.0, 141.2, 140.9, 140.7, 140.4, 140.3, 135.7, 133.6, 132.8, 132.1, 131.5, 130.3, 130.1, 129.3, 128.5, 128.2, 125.6, 125.2, 124.1, 123.1, 121.9 (2 × *C*H), 119.4, 118.8, 118.7, 118.2, 116.8, 116.6, 111.4, 27.5, 27.2, 27.1, 26.6 ppm. One quaternary carbon signal was not observed or co-incident. HRMS [TOF MS ES+]: m/z $[M + H]^+$

calcd. for $C_{19}H_{14}NS_2$ 320.0562, found 320.0578 (5.0 ppm). The sample for HRMS analysis is prepared form E/Z mixture of **4c**.

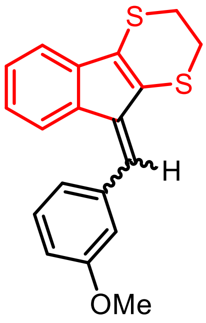

9-(3-Methoxybenzylidene)-2,3-dihydro-9*H*-indeno[1,2-*b*][1,4]dithiine (4d): The reaction was performed following General Procedure D with propargyl alcohol (**3d**) (0.25 mmol, 86 mg) and $Ga(OTf)_3$ (0.125 mmol, 6.5 mg) in anhydrous toluene (2.0 mL). The reaction was stirred for 40 min at 110 °C in an oil bath. The workup was performed following the general procedure. The crude material was purified by flash chromatography on silica gel using 30:1 hexanes/ethyl acetate as eluent to yield the product **4d** as a mixture of *E*/*Z* isomers (52 mg, 64%, red oil). **4d-(*E*)/(Z)**: ^{1}H-NMR (500 MHz, $CDCl_3$): δ *7.56 (d, *J* = 7.6 Hz, 1H), *7.44 (s, 1H), 7.42 (d, *J* = 7.7 Hz, 1H), 7.35 [t, *J* = 8.0 Hz, 1H (*E*), 1H (*Z*)], 7.29−7.26 (m, 1H), 7.19 [t, *J* = 7.4 Hz, 1H (*E*), 1H (*Z*)], 7.15−7.12 [m, 1H (*E*), 1 H (*Z*)], 7.08−7.03 [m, 3H (*E*), 2 H (*Z*)], 6.95−6.92 [m, 2H (*E*), 1H (*Z*)], *3.87 (s, 3H), 3.82 (s, 3H), 3.41−3.38 (m, 2H), 3.37−3.33 [m, 2H (*E*), 2H (*Z*)], *3.06−3.04 (m, 2H) ppm. **4d-(*E*)**: ^{13}C{^{1}H} NMR (126 MHz, $CDCl_3$, APT) δ 159.4, 142.8, 139.0, 137.6, 133.3, 129.4, 128.0, 127.5, 127.0, 124.9, 123.5, 122.3, 121.8, 116.4, 114.3, 114.2, 55.2, 27.0, 26.7 ppm. **4d-(*Z*)**: ^{13}C{^{1}H} NMR (126 MHz, $CDCl_3$, APT) δ 159.0, 140.3, 138.6, 137.3, 136.2, 131.8, 129.0, 127.6, 125.2, 124.7, 122.2, 120.2, 118.0, 116.3, 114.9, 114.2, 55.2, 27.8, 27.3 ppm. HRMS [TOF MS ES+]: m/z $[M + H]^+$ calcd. for $C_{19}H_{17}OS_2$ 325.0715, found 325.0715 (0.0 ppm). The sample for HRMS analysis is prepared form E/Z mixture of **4d**.

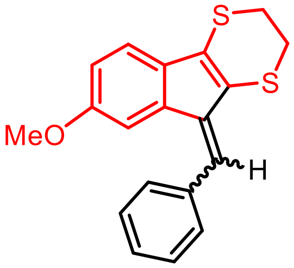

9-Benzylidene-7-methoxy-2,3-dihydro-9*H*-indeno[1,2-*b*][1,4]dithiine (4e): The reaction was performed following General Procedure D with propargyl alcohol (**3e**) (0.5 mmol, 171 mg) and $Ga(OTf)_3$ (0.025 mmol, 13 mg) in anhydrous toluene (3.0 mL). The reaction was stirred for 40 min at 110 °C in an oil bath. The workup was performed following the general procedure. The crude material was purified by flash chromatography on silica gel using 30:1 hexanes/ethyl acetate as eluent to yield the product **4e** as a mixture of *E*/*Z* isomers (116 mg, 72%, red oil). Isomers are partially separated by flash chromatography on silica gel using hexanes as eluent. **4e-**

(***E***): ^{1}H-NMR (500 MHz, $CDCl_3$) δ 7.55−7.52 (m, 2H), 7.45−7.42 (m, 2H), 7.38−7.35 (m, 1H), 7.05 (s, 1H), 6.97 (d, *J* = 2.4 Hz, 1H), 6.95 (d, *J* = 8.2 Hz, 1H), 6.70 (dd, *J* = 2.4, 8.2 Hz, 1H), 3.61 (s, 3H), 3.40−3.37 (m, 2H), 3.33−3.30 (m, 2H) ppm. ^{13}C{^{1}H} NMR (126 MHz, $CDCl_3$, APT) δ 157.8, 139.0, 136.2, 136.1, 134.9, 129.2, 128.3, 128.1, 127.5, 126.8, 121.2, 116.7, 112.2, 109.7, 55.2, 27.1, 26.6 ppm. HRMS [TOF MS ES+]: m/z $[M + H]^+$ calcd. for $C_{19}H_{17}OS_2$ 325.0715, found 325.0709 (– 1.85 ppm). **4e-(*Z*)**: ^{1}H-NMR (500 MHz, $CDCl_3$) δ 7.46−7.44 (m, 2H), 7.41−7.36 (m, 4H), 7.16 (d, *J* = 2.5 Hz, 1H), 7.02 (d, *J* = 8.1 Hz, 1H), 6.79 (dd, *J* = 2.5, 8.2 Hz, 1H), 3.86 (s, 3H), 3.35−3.33 (m, 2H), 3.03−3.01 (m, 2H) ppm. ^{13}C{^{1}H} NMR (126 MHz, $CDCl_3$, APT) δ 158.5, 138.5, 138.1, 136.0, 133.8, 131.7, 129.8, 128.1, 127.9, 123.8, 118.1, 116.8, 112.2, 105.5, 55.7, 27.7, 27.4 ppm. HRMS [TOF MS ES+]: m/z $[M]^+$ calcd. for $C_{19}H_{16}OS_2$ 324.0637, found 324.0635 (– 0.62 ppm).

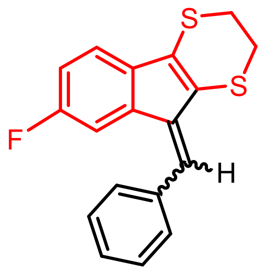

9-Benzylidene-7-fluoro-2,3-dihydro-9*H*-indeno[1,2-*b*][1,4]dithiine (4e): The reaction was performed following General Procedure D with propargyl alcohol (**3f**) (0.5 mmol, 165 mg) and $Ga(OTf)_3$ (0.025 mmol, 13 mg) in anhydrous toluene (3.0 mL). The reaction was stirred for 40 min at 110 °C in an oil bath. The workup was performed following the general procedure. The crude material was purified by flash chromatography on silica gel using 20:1 hexanes/ethyl acetate as eluent to yield the product **4e** as a mixture of *E*/*Z* isomers (69 mg, 44%, red oil). Isomers are partially separated by flash chromatography on silica gel using hexanes as eluent. **4e-(*E*)**: ^{1}H-NMR (500 MHz, $CDCl_3$) δ 7.51−7.49 (m, 2H), 7.46−7.43 (m, 2H), 7.41−7.38 (m, 1H), 7.11 (s, 1H), 7.06 (dd, *J* = 2.3, 10.0 Hz, 1H), 6.95 (dd, *J* = 5.2, 8.3 Hz, 1H), 6.88 (dt, *J* = 2.3, 8.3 Hz, 1H), 3.41−3.38 (m, 2H), 3.35−3.32 (m, 2H) ppm. ^{13}C{^{1}H} NMR (126 MHz, $CDCl_3$, APT) δ 161.1 (d, *J* = 242.0 Hz), 138.8 (d, *J* = 2.4 Hz), 138.3 (d, *J* = 2.6 Hz), 135.7, 135.1 (d, *J* = 9.0 Hz), 129.2, 128.5, 128.4, 128.2, 126.9 (d, *J* = 1.4 Hz), 123.1 (d, *J* = 3.7 Hz), 116.8 (d, *J* = 9.1 Hz), 114.1 (d, *J* = 23.4 Hz), 110.3 (d, *J* = 25.8 Hz), 27.1, 27.0 ppm. HRMS [TOF MS ES+]: m/z $[M]^+$ calcd. for $C_{18}H_{13}FS_2$ 312.0437, found 312.0435 (– 0.64 ppm). **4e-(*Z*)**: ^{1}H-NMR (500 MHz, $CDCl_3$) δ 7.46−7.38 (m, 6H), 7.27−7.25 (m, 1H), 7.03 (dd, *J* = 5.0, 8.0 Hz, 1H), 6.97−6.93 (m, 1H), 3.36−3.34 (m, 2H), 3.04−3.02 (m, 2H) ppm. ^{13}C{^{1}H} NMR (126 MHz, $CDCl_3$, APT) δ 161.8 (d, *J* = 243.0 Hz), 138.3 (d, *J* = 8.6 Hz), 137.9 (d, *J* = 2.7 Hz), 136.4 (d, *J*

= 2.3 Hz), 135.6, 131.0 (d, *J* = 1.5 Hz), 130.0, 128.4, 128.0, 125.7, 120.0 (d, *J* = 3.7 Hz), 117.0 (d, *J* = 8.8 Hz), 113.8 (d, *J* = 23.1 Hz), 27.7, 27.3 ppm. HRMS [TOF MS ES+]: m/z [M + H]$^+$ calcd. for $C_{18}H_{14}FS_2$ 313.0515, found 313.0518 (0.96 ppm).

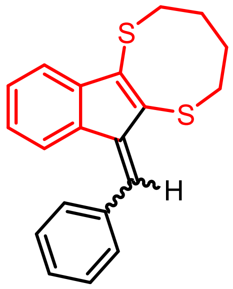

11-Benzylidene-2,3,4,5-tetrahydro-11*H*-indeno[1,2-*b*][1,4]dithiocine (6a): The reaction was performed following General Procedure D with propargyl alcohol (**5a**) (0.35 mmol, 120 mg) and $Ga(OTf)_3$ (0.0176 mmol, 9.0 mg) in anhydrous toluene (2.0 mL). The reaction was stirred for 5 min at 110 °C in an oil bath. The workup was performed following the general procedure. The crude material was purified by flash chromatography on silica gel using 30:1 hexanes/ethyl acetate as eluent to yield the product **6a** as a mixture of *E*/*Z* isomers (50 mg, 44%, orange oil). Isomers are partially separated by flash chromatography on silica gel using hexanes as eluent. **6a-(*E*)**: ^{1}H-NMR (500 MHz, $CDCl_3$) δ 7.67 (s, 1H), 7.58–7.56 (m, 2H), 7.50 (d, *J* = 7.8 Hz, 1H), 7.46–7.43 (m, 2H), 7.40–7.37 (m, 2H), 7.28 (dt, *J* = 1.1, 7.6 Hz, 1H), 7.06 (dt, *J* = 1.1, 7.6 Hz, 1H), 4.13 (t, *J* = 6.3 Hz, 2H), 3.02 (t, *J* = 5.8 Hz, 2H), 2.21–2.16 (m, 2H), 2.08–2.03 (m, 2H) ppm. ^{13}C{^{1}H} NMR (126 MHz, $CDCl_3$, APT) δ 143.6, 142.4, 140.8, 137.0, 133.8, 129.5, 128.8, 128.3, 127.8, 127.7, 125.8, 125.1, 122.3, 118.6, 36.1, 31.9, 29.3, 23.4 ppm. HRMS [TOF MS ES+]: m/z [M + H]$^+$ calcd. for $C_{20}H_{19}S_2$ 323.0923, found 323.0927 (1.24 ppm). **6a-(*Z*)**: ^{1}H-NMR (500 MHz, $CDCl_3$) δ 7.63 (d, *J* = 6.9 Hz, 1H), 7.55 (s, 1H), 7.46–7.44 (m, 2H), 7.42–7.41 (m, 1H), 7.39–7.32 (m, 4H), 7.29 (dt, *J* = 1.4, 7.4 Hz, 1H), 3.92 (t, *J* = 6.2 Hz, 2H), 2.91 (t, *J* = 5.8 Hz, 2H), 2.12–2.07 (m, 2H), 2.01–1.97 (m, 2H) ppm. ^{13}C{^{1}H} NMR (126 MHz, $CDCl_3$, APT) δ 146.3, 140.7, 140.1, 137.2, 136.6, 130.1, 127.6, 127.5, 127.2, 126.5, 126.0, 124.1, 118.4, 118.1, 35.5, 31.0, 30.0, 24.5 ppm. HRMS [TOF MS ES+]: m/z [M + H]$^+$ calcd. for $C_{20}H_{19}S_2$ 323.0923, found 323.0922 (–0.31 ppm).

7. Large Scale Preparation of Benzofulvene 2d

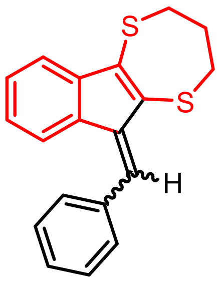

An oven-dried 100 mL schlenk flask equipped with a stirring bar was charged with propargyl alcohol **1d** (4.0 mmol, 1.31 g) and then the vial was brought into a glovebox. The reaction vial was charged with Ga(OTf)$_3$ (103.4 mg, 5mol%) and anhydrous toluene (24 mL). The flask was tightly closed with a rubber septa and wrapped with a strip of Parafilm, and taken out of the glovebox. After the reaction was stirred for 10 min (as judged by TLC) at 110 °C in an oil bath, the vial was cooled to room temperature. The reaction mixture was taken into a 100 mL flask and the solvent was removed in a rotatory evaporator. The remaining residue was dissolved in CH_2Cl_2 and mixed with silica gel (5 g). After evaporating CH_2Cl_2, the remaining silica gel was directly loaded onto a column and purified by flash chromatography on silica gel using 60:1 hexanes/ethyl acetate as eluent to yield the product **2d** as a mixture of *E*/*Z* isomers (0.91 g, 74% yield, red oil).

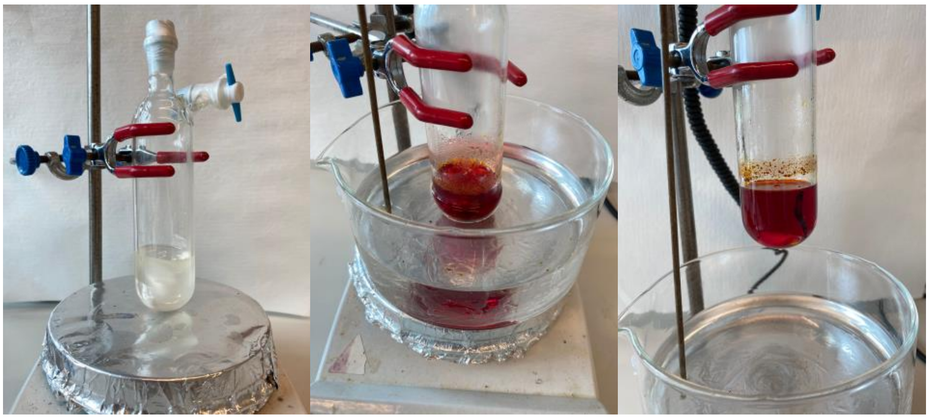

8. Optimization of reaction conditions for indanone 2a':

Table S3. Optimization of reaction conditions for indanone **2a'**

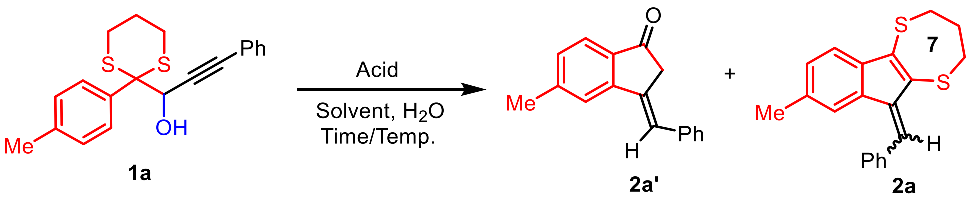

Entry	Acid (mol %)	H_2O (equiv.)	Solvent	Temp. (°C)/ Time (min)	**2a'** (% yield)	**2a**-(*E*:*Z*) (% yield)
1	$Bi(OTf)_3$ (25 mol %)	2.0	1,2-DCE	110°C/10 min	57%	trace
2	$Bi(OTf)_3$ (25 mol %)	4.0	1,2-DCE	110°C/10 min	35%	47% (1:0)
3	$Bi(OTf)_3$ (50 mol %)	2.0	1,2-DCE	110°C/10 min	53%	trace
4	$Bi(OTf)_3$ (25 mol %)	-	1,2-DCE	110 °C/10 min	31%	23% (16:1)
5	$Bi(OTf)_3$ (50 mol %)	2.0	Toluene	110 °C/20 min	53%	18% (3:1)
6	$Bi(OTf)_3$ (5 mol %)	-	Toluene	110 °C/5 min	Trace	51% (4.5:1)
7	$Ga(OTf)_3$ (5 mol %)	2.0	Toluene	110°C/80 min	31%	54% (10:1)
8	*p*-TsOH.H_2O (100 mol %)	1.0	Toluene	110°C/15 min	49%	15% (7.7:1)
9	TfOH (25 mol %)	-	Toluene	110°C/10 min	51%	18% (9:1)
10	TfOH (25 mol %)	2.0	Toluene	110°C/10 min	51%	14% (1:0)

9. Synthesis of 3-benzylidene-1-indanones

9.1. General procedure E: An oven-dried 15 mL screw-cap reaction vial equipped with a stirring bar was charged with α-dithioacetyl propargyl alcohol derivative (1.0 equiv.) and water (2.0 equiv.) by a micropipette and then the vial was brought into a glovebox. The reaction vial was charged with $Bi(OTf)_3$ (0.25 equiv. 25 mol%) and anhydrous 1,2-dichoroethane. The vial was tightly closed, wrapped with a strip of Parafilm, and taken out of the glovebox. After having stirred the reaction mixture for the given time at 110 °C in a pre-heated oil bath, the vial was cooled to room temperature. The reaction mixture was taken into a 50 mL flask and the solvent was removed in a rotatory

evaporator. The remaining residue was dissolved in CH_2Cl_2 and mixed with silica gel (about 0.5-1.0 g). After evaporating CH_2Cl_2, the remaining silica gel was directly loaded onto a column and purified by flash chromatography on silica gel using hexanes/ethyl acetate mixture as eluent to yield the product.

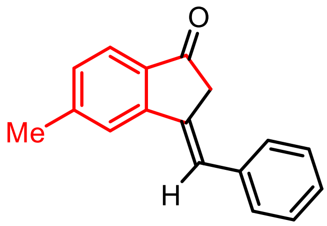

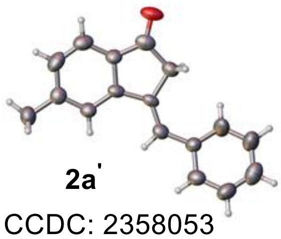

2a'
CCDC: 2358053

(*E*)-3-Benzylidene-5-methyl-2,3-dihydro-1*H*-inden-1-one (**2a'**): The reaction was performed following General Procedure E with propargyl alcohol (**1a**) (0.25 mmol, 85 mg) and H_2O (0.50 mmol, 9.0 µL). In glovebox, the reaction vial was charged with $Bi(OTf)_3$ (0.063 mmol, 41 mg) and anhydrous 1,2-dichloroethane (2.0 mL). The reaction was stirred for 10 min at 110 °C in an oil bath. The workup was performed following the general procedure. The crude material was purified by flash chromatography on silica gel using 30:1 hexanes/ethyl acetate as eluent to yield the product **2a'** (33.5 mg, 57%, yellowish-white solid, mp: 89-90 °C). **2a'**: ^{1}H-NMR (500 MHz, $CDCl_3$) δ 7.70−7.69 (m, 2H), 7.50−7.48 (m, 2H), 7.43−7.40 (m, 2H), 7.31−7.28 (m, 1H), 7.25−7.23 (m, 1H), 7.18−7.17 (m, 1H), 3.54 (d, *J* = 2.0 Hz, 2H), 2.51 (s, 3H) ppm. $^{13}C\{^1H\}$ NMR (126 MHz, $CDCl_3$, APT) δ 202.3, 152.1, 146.0, 136.6, 133.8, 132.4, 130.3, 129.0, 128.8, 127.7, 123.5, 122.8, 120.9, 41.9, 22.3 ppm. HRMS [TOF MS ES+]: m/z $[M + H]^+$ calcd. for $C_{17}H_{15}O$ 235.1117, found 235.1122 (2.13 ppm).

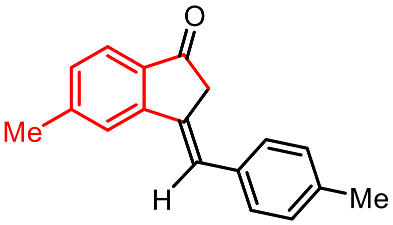

(*E*)-5-Methyl-3-(4-methylbenzylidene)-2,3-dihydro-1*H*-inden-1-one (**2b'**): The reaction was performed following General Procedure E with propargyl alcohol (**1b**) (0.25 mmol, 89 mg) and H_2O (0.50 mmol, 9.0 µL). In glovebox, the reaction vial was charged with $Bi(OTf)_3$ (0.063 mmol, 41 mg) and anhydrous 1,2-dichloroethane (2.0 mL). The reaction was stirred for 10 min at 110 °C in an oil bath. The workup was performed following the general procedure. The crude material was purified by flash chromatography on silica gel using 30:1 hexanes/ethyl acetate as eluent to yield the

product **2b'** (28.0 mg, 45%, white-yellowish solid, mp: 94-95 °C). **2b'**: ^{1}H-NMR (500 MHz, $CDCl_3$) δ 7.68−7.66 (m, 2H), 7.38 (d, *J* = 8.1 Hz, 2H), 7.22−7.21 (m, 3H), 7.13 (s, 1H), 3.50 (s, 2H), 2.49 (s, 3H), 2.38 (s, 3H) ppm. ^{13}C{^{1}H} NMR (126 MHz, $CDCl_3$, APT) δ 202.5, 152.3, 146.0, 137.7, 133.8, 133.7, 131.3, 130.0, 129.5, 128.9, 123.4, 122.7, 120.8, 41.9, 22.3, 21.2 ppm. HRMS [TOF MS ES+]: m/z $[M + H]^+$ calcd. for $C_{18}H_{17}O$ 249.1274, found 249.1273 (0.40 ppm).

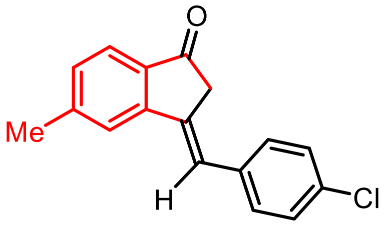

(*E*)-3-(4-Chlorobenzylidene)-5-methyl-2,3-dihydro-1*H*-inden-1-one (**2c'**): The reaction was performed following General Procedure E with propargyl alcohol (**1c**) (0.25 mmol, 94 mg) and H_2O (0.50 mmol, 9.0 μL). In glovebox, the reaction vial was charged with $Bi(OTf)_3$ (0.063 mmol, 41 mg) and anhydrous 1,2-dichloroethane (2.0 mL). The reaction was stirred for 10 min at 110 °C in an oil bath. The workup was performed following the general procedure. The crude material was purified by flash chromatography on silica gel using 30:1 hexanes/ethyl acetate as eluent to yield the product **2c'** (33.0 mg, 50%, orange solid, mp: 98-99 °C). **2c'**: ^{1}H-NMR (500 MHz, $CDCl_3$) δ ^{1}H-NMR (500 MHz, $CDCl_3$) δ 7.67 (d, *J* = 8.0 Hz, 1H), 7.64 (s, 1H), 7.39−7.34 (m, 4H), 7.23 (d, *J* = 7.9 Hz, 1H), 7.08 (bs, 1H), 3.45 (s, 2H), 2.49 (s, 3H) ppm. ^{13}C{^{1}H} NMR (126 MHz, $CDCl_3$, APT) δ 201.8, 151.7, 146.1, 135.0, 133.8, 133.4, 131.3, 130.0, 130.5, 130.0, 128.9, 123.5, 121.4, 120.9, 41.7, 22.3 ppm. HRMS [TOF MS ES+]: m/z $[M + H]^+$ calcd. for $C_{17}H_{14}ClO$ 269.0728, found 269.0729 (0.37 ppm).

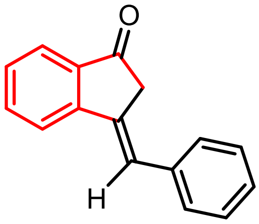

(*E*)-3-Benzylidene-2,3-dihydro-1*H*-indene-1-one (**2d'**): The reaction was performed following General Procedure E with propargyl alcohol (**1d**) (0.25 mmol, 82 mg) and H_2O (0.50 mmol, 9.0 μL). In glovebox, the reaction vial was charged with $Bi(OTf)_3$ (0.063 mmol, 41 mg) and anhydrous 1,2-dichloroethane (2.0 mL). The reaction was stirred for 10 min at 110 °C in an

oil bath. The workup was performed following the general procedure. The crude material was purified by flash chromatography on silica gel using 30:1 hexanes/ethyl acetate as eluent to yield the product **2d'** (24 mg, 43%, yellowish solid, mp: 88-89 °C). **2d'**: ^{1}H-NMR (500 MHz, $CDCl_3$) δ 7.99 (d, *J* = 7.9 Hz, 1H), 7.80 (d, *J* = 7.7 Hz, 1H), 7.66 (t, *J* = 7.9 Hz, 1H), 7.49–7.48 (m, 2H), 7.43–7.40 (m, 3H), 7.30 (t, *J* = 7.3 Hz, 1H), 7.20 (s, 1H), 3.54 (s, 2H) ppm. $^{13}C\{^1H\}$ NMR (126 MHz, $CDCl_3$, APT) δ 202.8, 151.7, 136.5, 135.8, 134.9, 132.2, 129.0, 128.8, 127.7, 123.6, 123.1, 120.8, 41.6 ppm. One methine carbon signal was not observed or co-incident. HRMS [TOF MS ES+]: m/z $[M + H]^+$ calcd. for $C_{16}H_{13}O$ 221.0961, found 221.0961 (0 ppm).

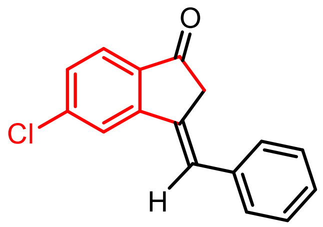

(*E*)-3-Benzylidene-5-chloro-2,3-dihydro-1*H*-inden-1-one (**2e'**): The reaction was performed following General Procedure E with propargyl alcohol (**1e**) (0.25 mmol, 90 mg) and H_2O (0.50 mmol, 9.0 μL). In glovebox, the reaction vial was charged with $Bi(OTf)_3$ (0.063 mmol, 41 mg) and anhydrous 1,2-dichloroethane (2.0 mL). The reaction was stirred for 20 min at 110 °C in an oil bath. The workup was performed following the general procedure. The crude material was purified by flash chromatography on silica gel using 30:1 hexanes/ethyl acetate as eluent to yield the product **2e'** (26.0 mg, 40%, orange solid, mp: 97-98 °C). **2e'**: ^{1}H-NMR (500 MHz, $CDCl_3$) δ 7.85 (d, *J* = 1.8 Hz, 1H), 7.72 (d, *J* = 8.2 Hz, 1H), 7.49–7.47 (m, 2H), 7.44–7.41 (m, 2H), 7.37 (dd, *J* = 1.8, 8.2 Hz, 1H), 7.34–7.31 (m, 1H), 7.16 (bs, 1H), 3.55 (d, *J* = 2.1 Hz, 2H) ppm. $^{13}C\{^1H\}$ NMR (126 MHz, $CDCl_3$, APT) δ 201.3, 153.0, 141.7, 136.0, 134.1, 130.9, 129.3, 129.1, 128.9, 128.2, 124.8, 124.4, 121.0, 41.7 ppm. HRMS [TOF MS ES+]: m/z $[M + H]^+$ calcd. for $C_{16}H_{12}ClO$ 255.0571, found 255.0571 (0 ppm).

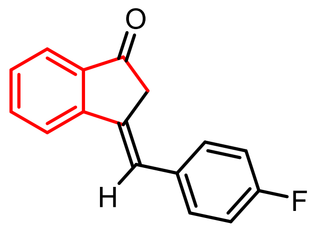

(*E*)-3-(4-Fluorobenzylidene)-2,3-dihydro-1*H*-inden-1-one (**2g'**): The reaction was performed following General Procedure E with propargyl alcohol (**1g**) (0.25 mmol, 86 mg) and H_2O (0.50 mmol, 9.0 µL). In glovebox, the reaction vial was charged with $Bi(OTf)_3$ (0.063 mmol, 41 mg) and anhydrous 1,2-dichloroethane (2.0 mL). The reaction was stirred for 10 min at 110 °C in an oil bath. The workup was performed following the general procedure. The crude material was purified by flash chromatography on silica gel using 30:1 hexanes/ethyl acetate as eluent to yield the product **2g'** (24.0 mg, 40%, yellowish solid, mp: 126-127 °C). **2g'**: ^{1}H-NMR (500 MHz, $CDCl_3$) δ 7.87 (d, *J* = 8.0 Hz, 1H), 7.79 (d, *J* = 7.7 Hz, 1H), 7.66 (t, *J* = 7.6 Hz, 1H), 7.47–7.40 (m, 3H), 7.15 (s, 1H), 7.12–7.08 (m, 2H), 3.50 (s, 2H) ppm. $^{13}C\{^1H\}$ NMR (126 MHz, $CDCl_3$, APT) δ 202.5, 162.1 (d, *J* = 249 Hz), 151.5, 135.8, 134.9, 132.7 (d, *J* = 3.4 Hz), 131.9 (d, *J* = 2.3 Hz), 130.5 (d, *J* = 8.1 Hz), 128.8, 123.7, 121.9, 120.7, 115.8 (d, *J* = 21.5 Hz), 41.4 ppm. HRMS [TOF MS ES+]: m/z $[M + H]^+$ calcd. for $C_{16}H_{12}FO$ 239.0867, found 239.0866 (–0.42 ppm).

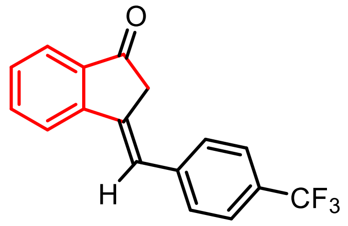

(*E*)-3-(4-(Trifluoromethyl)benzylidene)-2,3-dihydro-1*H*-inden-1-one (**2h'**): The reaction was performed following General Procedure E with propargyl alcohol (**1h**) (0.25 mmol, 99 mg) and H_2O (0.50 mmol, 9.0 µL). In glovebox, the reaction vial was charged with $Bi(OTf)_3$ (0.063 mmol, 41 mg) and anhydrous 1,2-dichloroethane (2.0 mL). The reaction was stirred for 40 min at 110 °C in an oil bath. The workup was performed following the general procedure. The crude material was purified by flash chromatography on silica gel using 30:1 hexanes/ethyl acetate as eluent to yield the product **2h'** (24.0 mg, 33%, yellowish-white solid, mp: 106-107 °C). **2h'**: ^{1}H-NMR (500 MHz, $CDCl_3$) δ 7.91 (d, *J* = 7.9 Hz, 1H), 7.82 (d, *J* = 7.6 Hz, 1H), 7.69 (t, *J* = 8.2 Hz, 1H), 7.67–7.65 (m, 2H, $AA'BB'_{system}$, $\delta_{AA'}$), 7.59–7.57 (m, 2H, $AA'BB'_{system}$, $\delta_{BB'}$), 7.47 (t, *J* = 7.5 Hz, 1H), 7.22 (s, 1H), 3.55 (s, 2H)

ppm. $^{13}C\{^1H\}$ NMR (126 MHz, $CDCl_3$, APT) δ 202.0, 151.0, 139.9, 136.1, 135.0, 134.9, 129.5, 129.2, 129.0, 125.7 (q, J = 4.0 Hz), 124.0 (q, J = 272 Hz), 123.8, 121.5, 121.0, 41.5 ppm. HRMS [TOF MS ES+]: m/z $[M + H]^+$ calcd. for $C_{17}H_{12}F_3O$ 289.0835, found 289.0834 (–0.35 ppm).

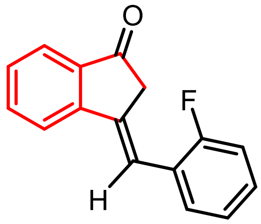

(*E*)-3-(2-Fluorobenzylidene)-2,3-dihydro-1*H*-inden-1-one (2o'): The reaction was performed following General Procedure E with propargyl alcohol (**1o**) (0.25 mmol, 86 mg) and H_2O (0.50 mmol, 9.0 μL). In glovebox, the reaction vial was charged with $Bi(OTf)_3$ (0.063 mmol, 41 mg) and anhydrous 1,2-dichloroethane (2.0 mL). The reaction was stirred for 40 min at 110 °C in an oil bath. The workup was performed following the general procedure. The crude material was purified by flash chromatography on silica gel using 30:1 hexanes/ethyl acetate as eluent to yield the product **2o'** (11.0 mg, 19%, yellowish solid, mp: 109-110 °C). **2o'**: ^1H-NMR (500 MHz, $CDCl_3$) δ 7.93 (d, J = 7.9 Hz, 1H), 7.80 (d, J = 7.7 Hz, 1H), 7.67 (t, J = 7.7 Hz, 1H), 7.52 (t, J = 7.7 Hz, 1H), 7.44 (t, J = 7.6 Hz, 1H), 7.38 (s, 1 H), 7.30−7.26 (m, 1H), 7.19 (t, J = 7.7 Hz, 1H), 7.13−7.10 (m, 1H), 3.49 (d, J = 1.9 Hz, 2H) ppm. $^{13}C\{^1H\}$ NMR (126 MHz, $CDCl_3$, APT) δ 202.4, 160.6 (d, J = 250 Hz), 151.2, 136.1, 135.0, 134.2 (d, J = 1.5 Hz), 129.3 (d, J = 8.6 Hz), 129.2, 129.0 (d, J = 2.8 Hz), 124.4 (d, J = 12.2 Hz), 124.2 (d, J = 3.7 Hz), 123.6, 121.1, 115.7 (d, J = 22.4 Hz), 114.7 (d, J = 5.5 Hz), 41.5 ppm. HRMS [TOF MS ES+]: m/z $[M + H]^+$ calcd. for $C_{16}H_{12}FO$ 239.0867, found 239.0869 (0.84 ppm).

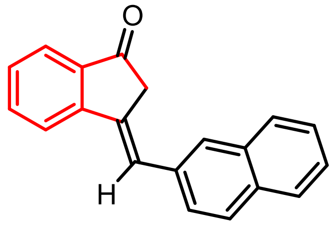

(*E*)-3-(naphthalen-2-ylmethylene)-2,3-dihydro-1*H*-inden-1-one (2q'): The reaction was performed following General Procedure E with propargyl alcohol (**1q**) (0.25 mmol, 94 mg) and H_2O (0.50 mmol, 9.0 μL). In glovebox, the reaction vial was charged with $Bi(OTf)_3$ (0.063 mmol, 41 mg) and anhydrous 1,2-dichloroethane (2.0 mL). The reaction was stirred for 20 min at 110 °C in an oil bath. The workup was performed following

the general procedure. The crude material was purified by flash chromatography on silica gel using 30:1 hexanes/ethyl acetate as eluent to yield the product **2q'** (31.0 mg, 45%, yellowish solid, mp: 138-139 °C). **2b'**: ^{1}H-NMR (500 MHz, $CDCl_3$) δ 7.94−7.93 (m, 2H), 7.87−7.81 (m, 4H), 7.68 (t, *J* = 7.5 Hz, 1H), 7.62 (d, *J* = 8.6 Hz, 1H), 7.52−7.47 (m, 2H), 7.43 (t, *J* = 7.4 Hz, 1H), 7.34 (s, 1H), 3.65 (s, 2H) ppm. ^{13}C{^{1}H} NMR (126 MHz, $CDCl_3$, APT) δ 202.7, 151.7, 135.8, 134.9, 134.0, 133.4, 132.6, 132.5, 128.8, 128.5, 128.3, 128.2, 127.6, 126.5, 126.4, 126.3, 123.6, 123.1, 120.8, 41.8 ppm. HRMS [TOF MS ES+]: m/z $[M + H]^+$ calcd. for $C_{20}H_{15}O$ 271.1117 found 271.1113 (– 1.48 ppm).

10. Observations of *E*/*Z* isomerization of benzofulvene 2c

Scheme S3.

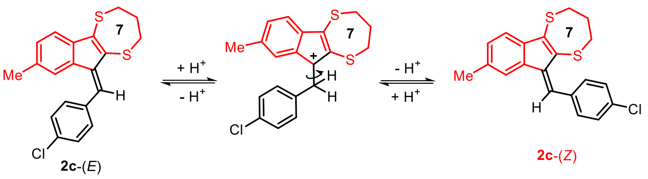

**2c** Initial Ratio	Conditions	**2c** Final Ratio
**2c**-(*E*) *E*:*Z* = 1:0	1 day in $CDCl_3$	**2c**-(*E*) *E*:*Z* = 1:0
**2c**-(*E*) *E*:*Z* = 1:0	2 days in $CDCl_3$	**2c**-(*E*/*Z*) *E*:*Z* = 17.5:1
**2c**-(*E*) *E*:*Z* = 1:0	3 days in $CDCl_3$	**2c**-(*E*/*Z*) *E*:*Z* = 8.1:1
**2c**-(*E*) *E*:*Z* = 1:0	11 days in $CDCl_3$	**2c**-(*E*/*Z*) *E*:*Z* = 7.7:1
**2c**-(*E*) *E*:*Z* = 1:0	15 days in $CDCl_3$	**2c**-(*E*/*Z*) *E*:*Z* = 7.7:1
**2c**-(*Z*) *E*:*Z* = 0:1	1 day in $CDCl_3$	**2c**-(*E*/*Z*) *E*:*Z* = 1:5.9
**2c**-(*Z*) *E*:*Z* = 0:1	3 days in $CDCl_3$	**2c**-(*E*/*Z*) *E*:*Z* = 7.3:1

^{1}H NMR spectra of pure *E* and *Z* isomers of benzofulvene **2c** were taken on each day in the given table and E/Z ratios are determined integrations of methylene peaks at 3.46 (t, *J* = 6.0 Hz, 2H), 3.39 (t, *J* = 6.1 Hz, 2H) ppm for *E* isomer, and 3.64 (t, *J* = 6.1 Hz, 2H), 3.34 (t, *J* = 6.1 Hz, 2H) ppm for *Z* isomer.

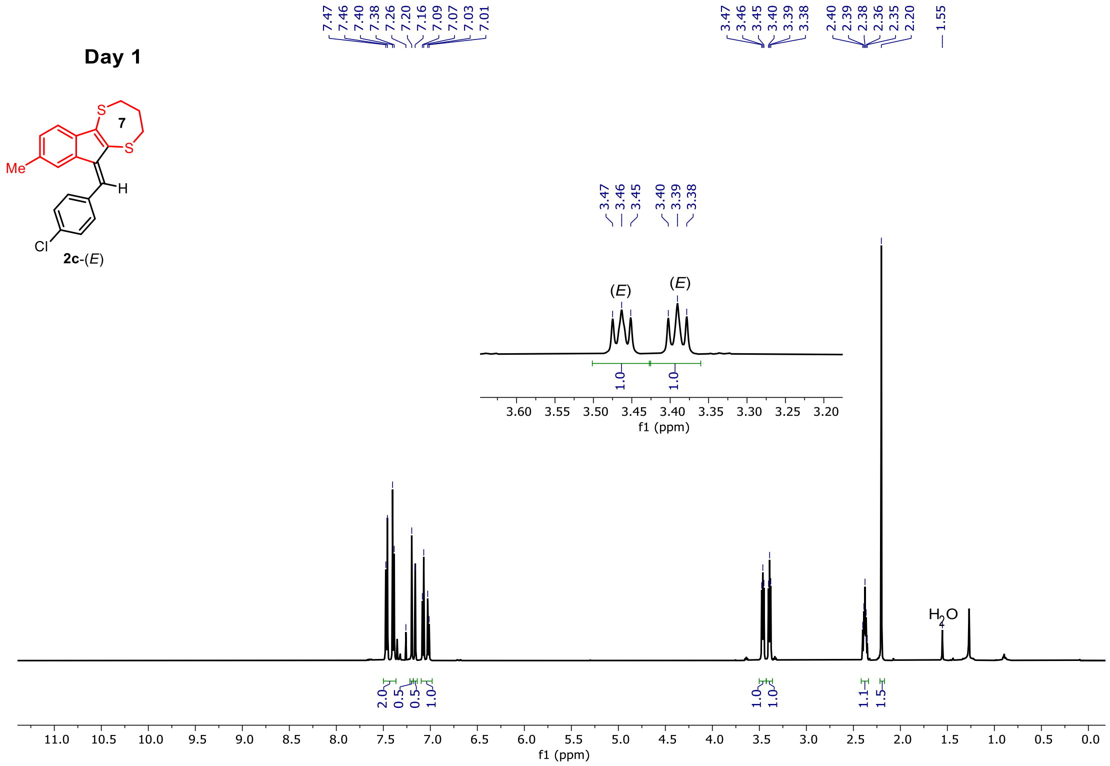

Figure S1. ^{1}H NMR (500 MHz, $CDCl_3$) spectra of **2c**-(*E*) (Day 1).

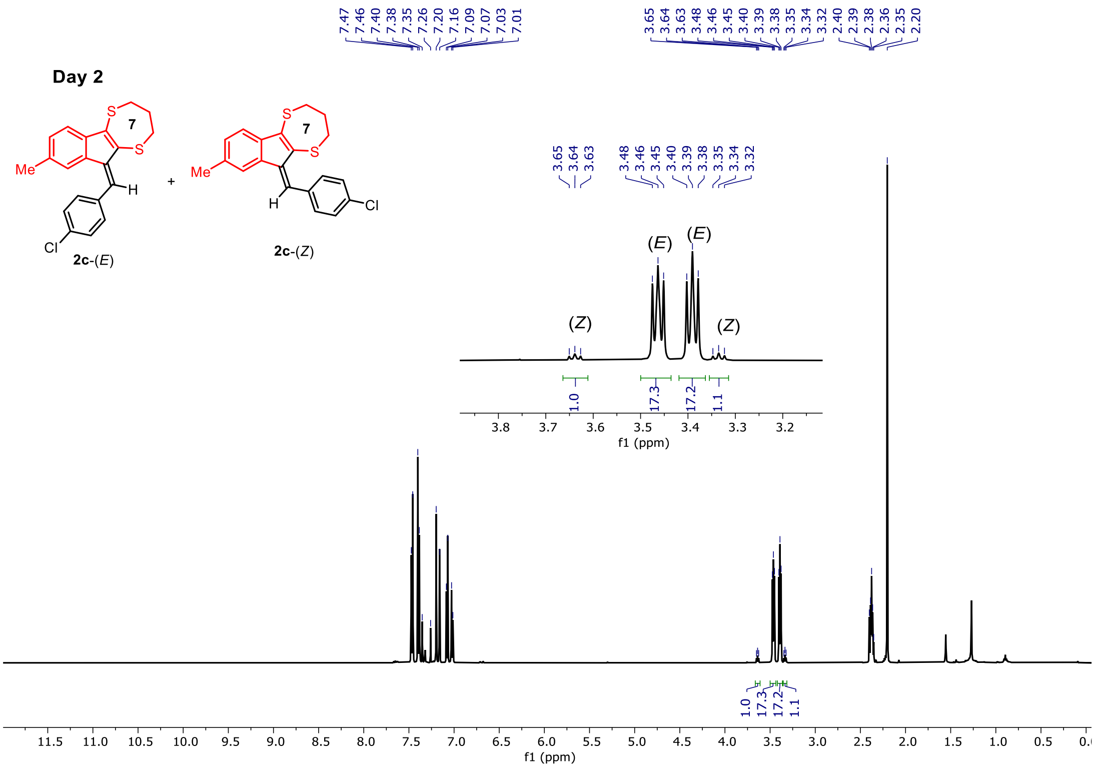

Figure S2. ^{1}H NMR (500 MHz, CDCl$_3$) spectra of **2c**-(*E*) (Day 2).

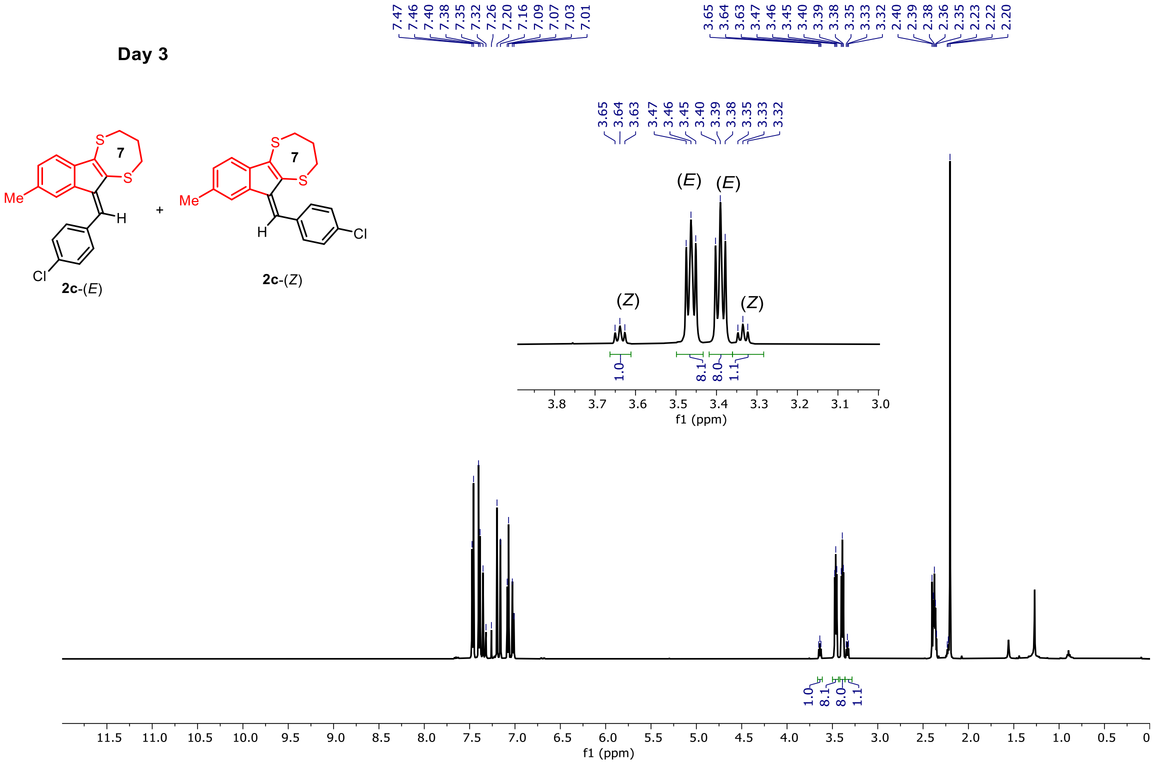

Figure S3. ^{1}H NMR (500 MHz, CDCl$_3$) spectra of **2c**-(*E*) (Day 3).

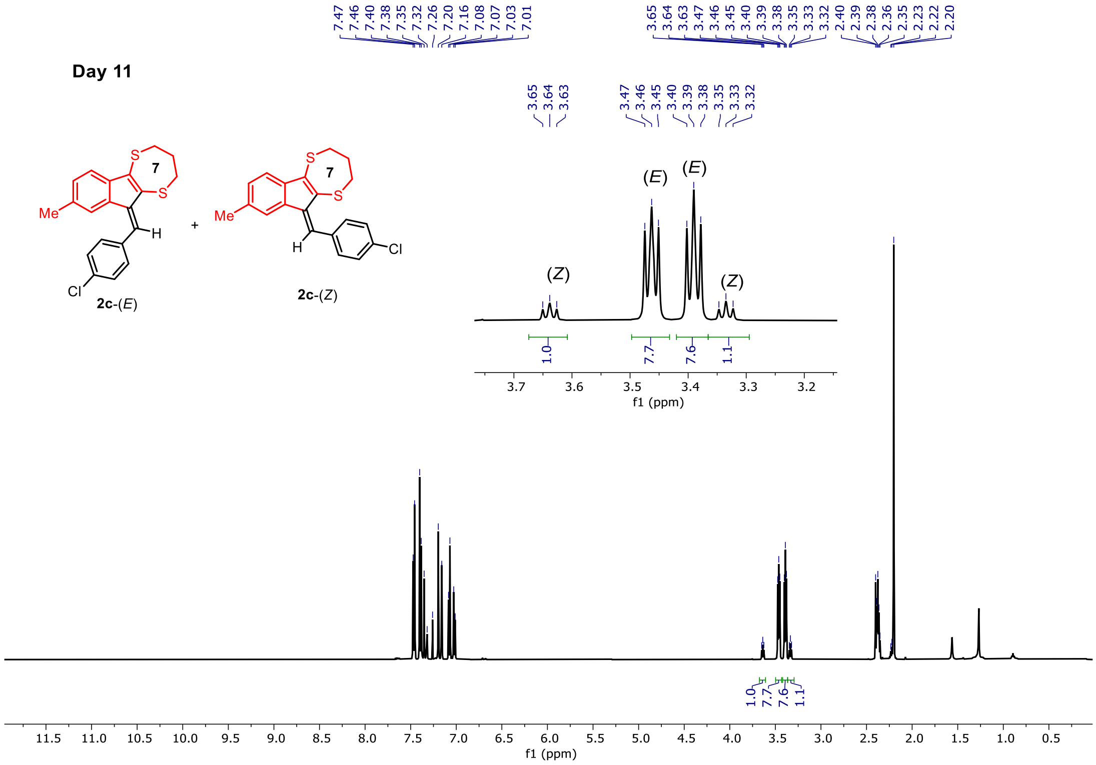

Figure S4. ^{1}H NMR (500 MHz, $CDCl_3$) spectra of **2c**-(*E*) (Day 11).

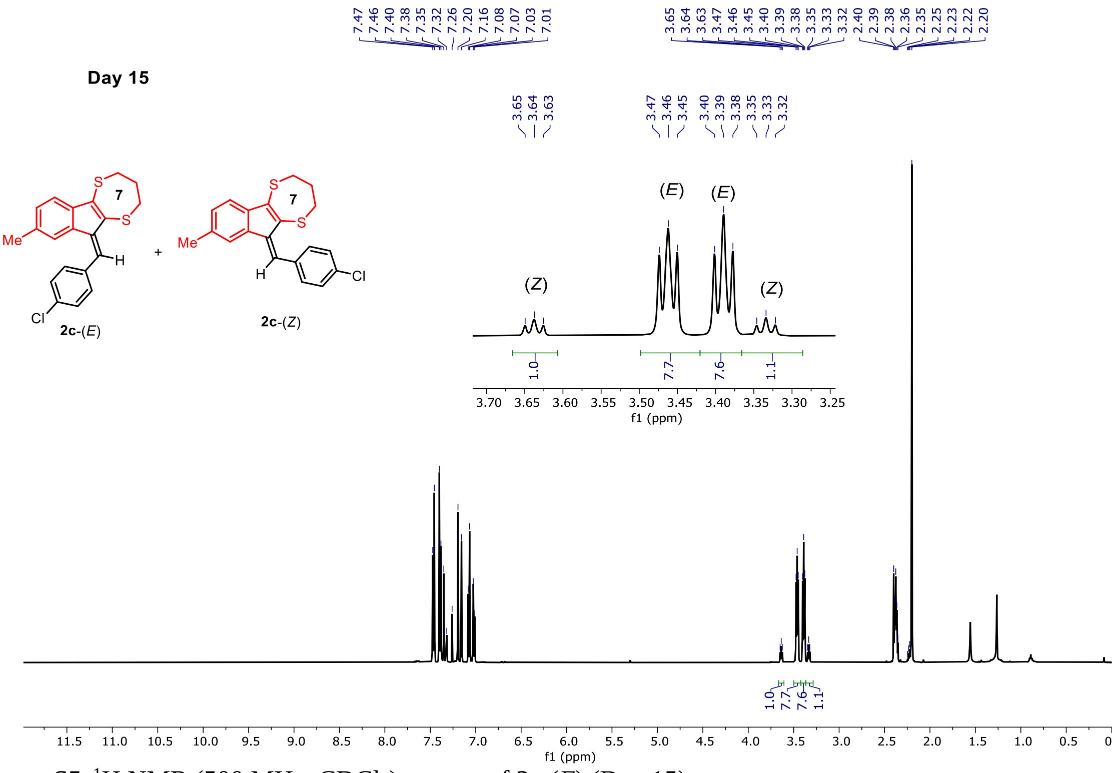

Figure S5. ^{1}H NMR (500 MHz, $CDCl_3$) spectra of **2c**-(*E*) (Day 15).

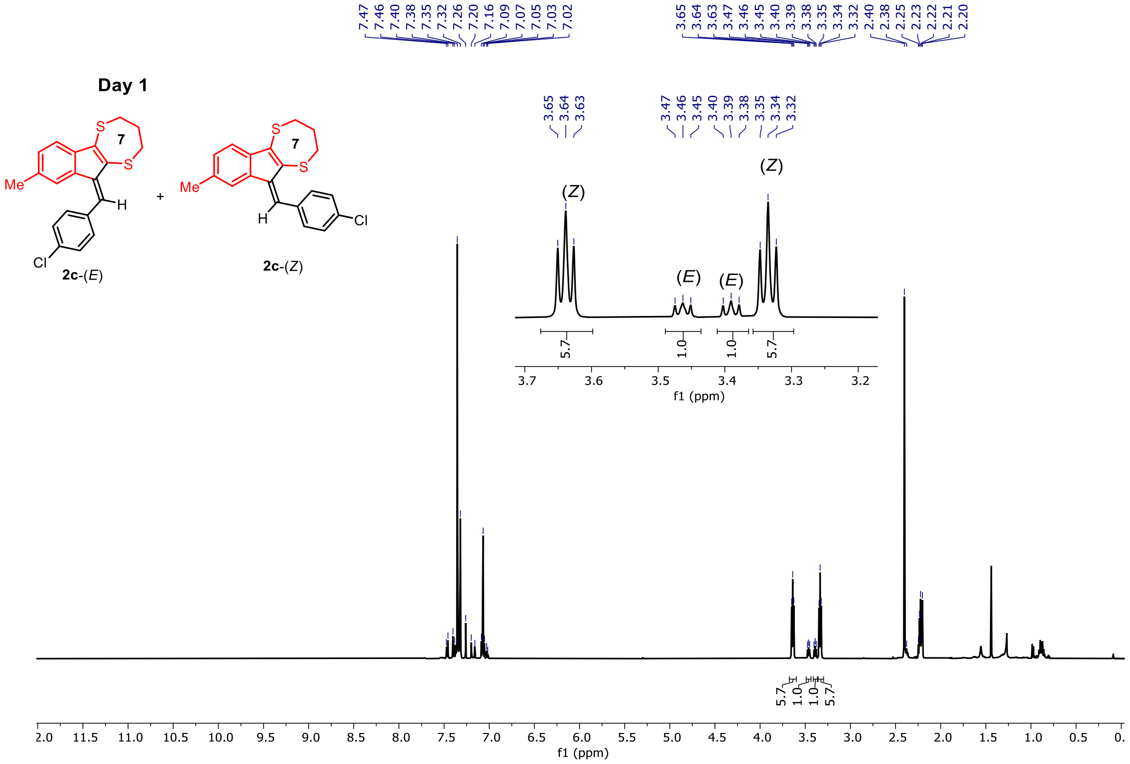

Figure S6. ^{1}H NMR (500 MHz, CDCl$_3$) spectra of **2c**-(*Z*) (Day 1).

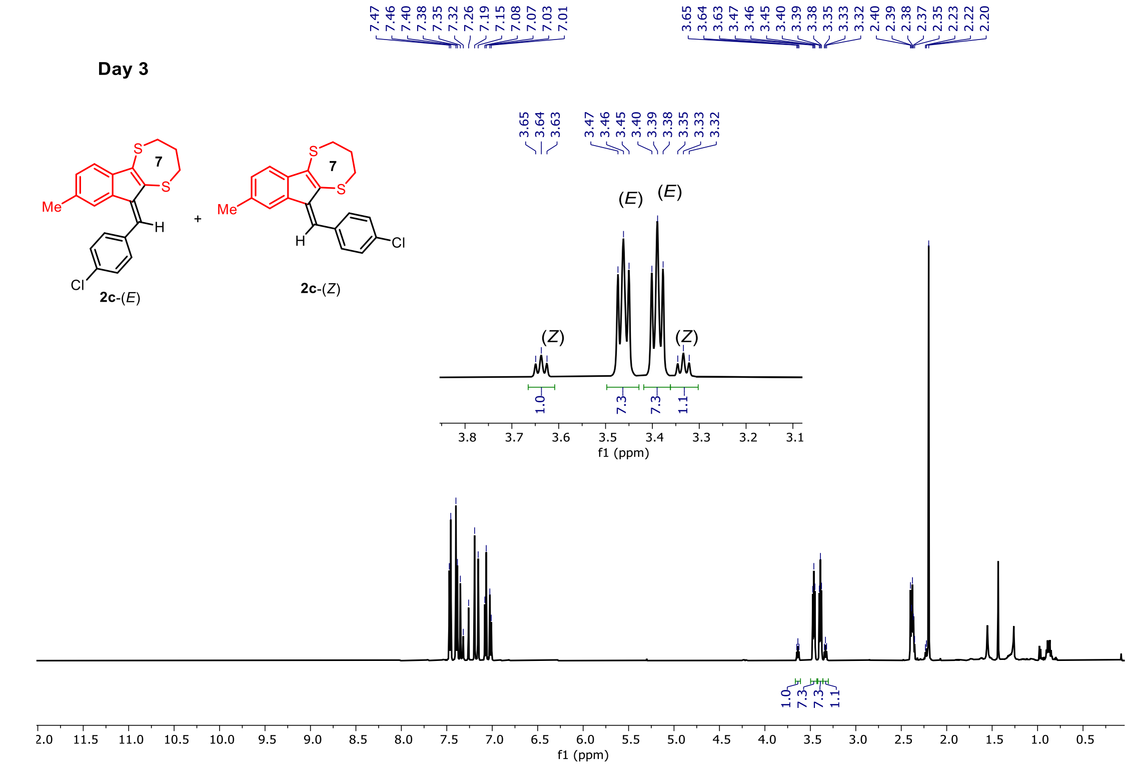

Figure S7. ^{1}H NMR (500 MHz, CDCl$_3$) spectra of **2c**-(*Z*) (Day 3).

11. 1D TOCSY NMR experiments for benzofulvene 2t-(*E*/*Z*)

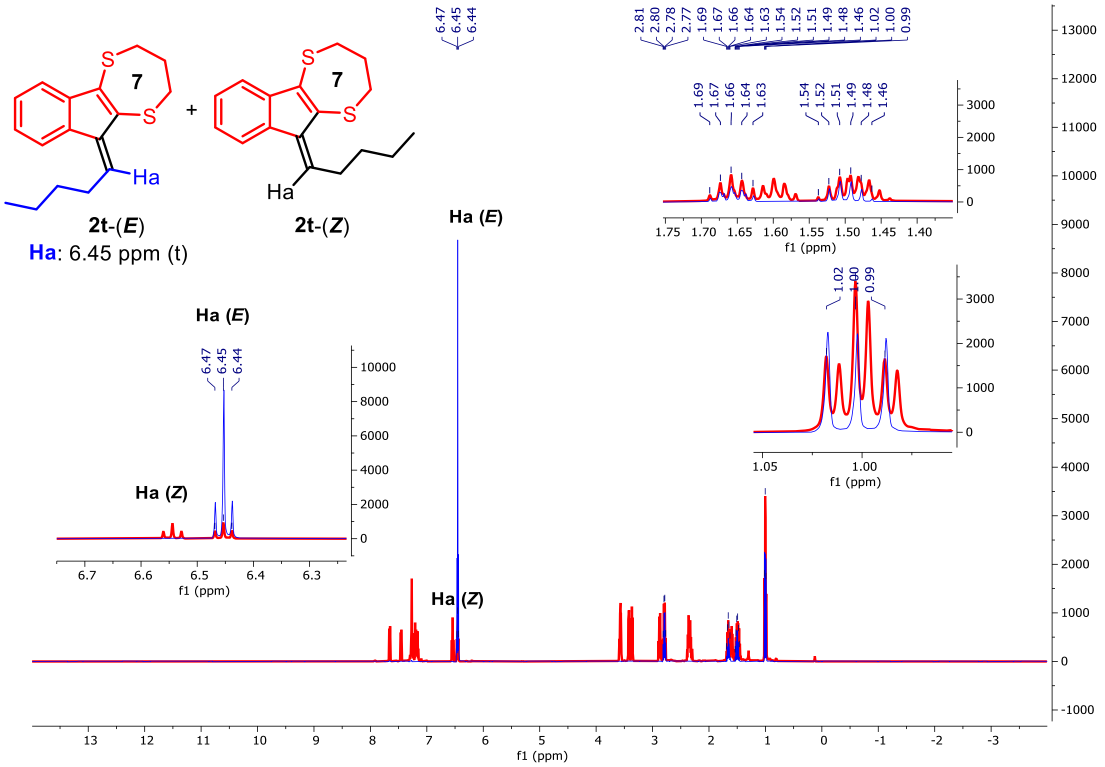

Figure S8. 1D TOCSY NMR experiment of **2t-(*E*/*Z*)**, selective excitation of **Ha** (***E***).

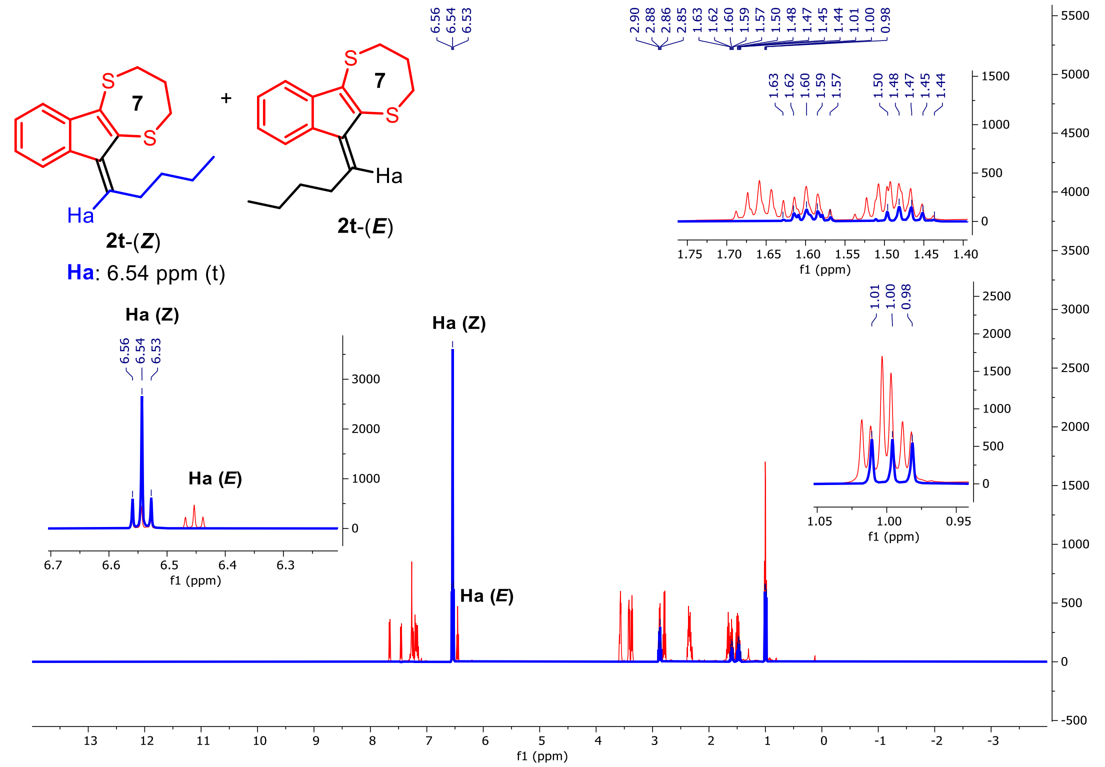

Figure S9. 1D TOCSY NMR experiment of **2t-(*E*/*Z*)**, selective excitation of **Ha** (***Z***).

12. 1D NOE NMR experiments for benzofulvane 2t-(*E*/*Z*)

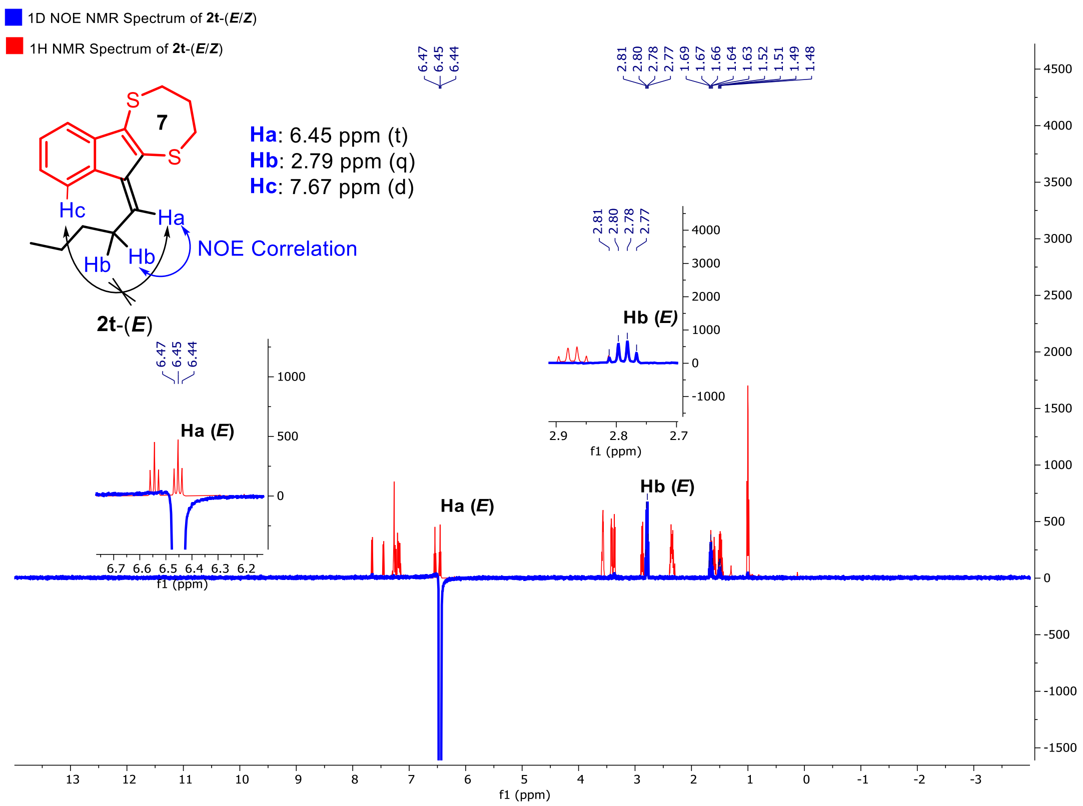

Figure S10. 1D NOE NMR experiment of **2t-(*E*/*Z*)**; saturation of **Ha** (***E***) (δ = 6.45)

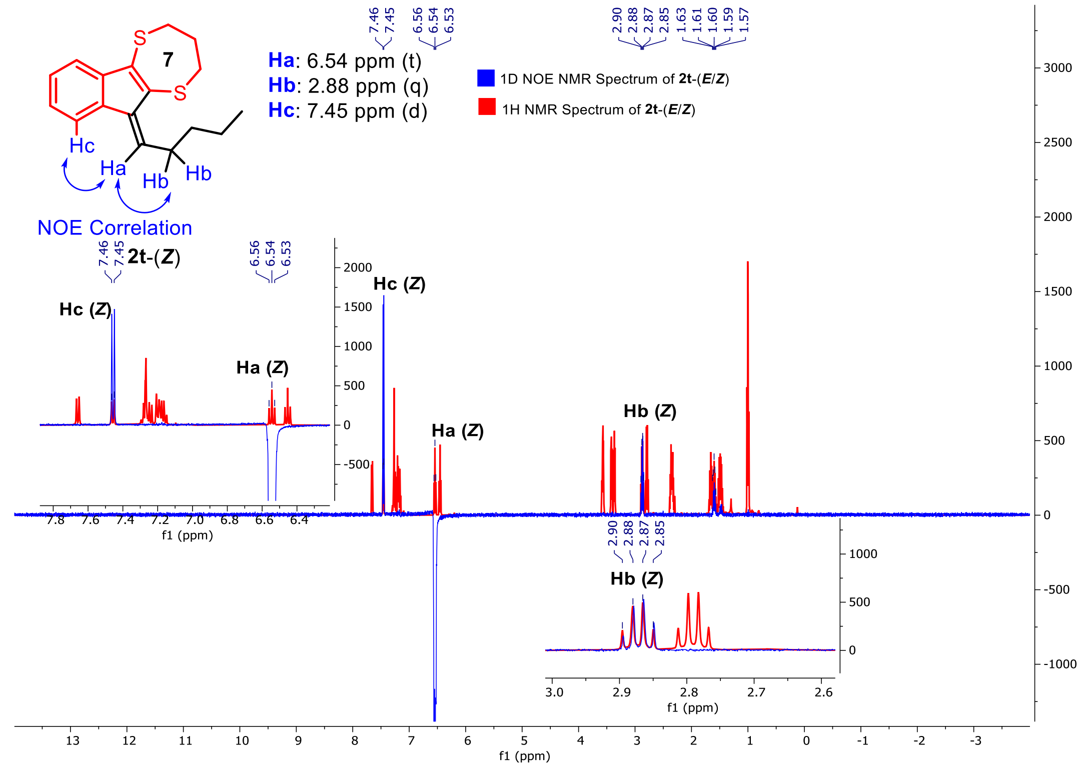

Figure S11. 1D NOE NMR experiment of **2t-(*E*/*Z*)**; saturation of **Ha** (***Z***) (δ = 6.54)

13. 1D NOE experiments for benzofulvane 6a-(*E*/*Z*)

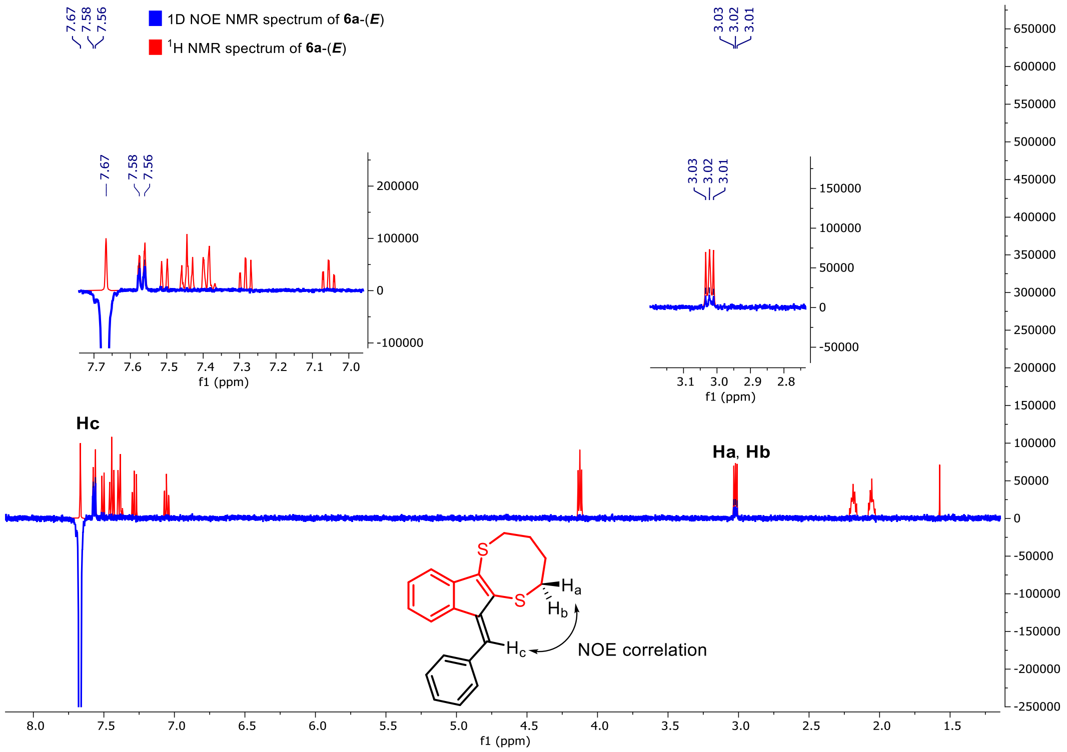

Figure S12. 1D NOE NMR experiment of **6a-(*E*)**; saturation of Hc (δ = 7.67)

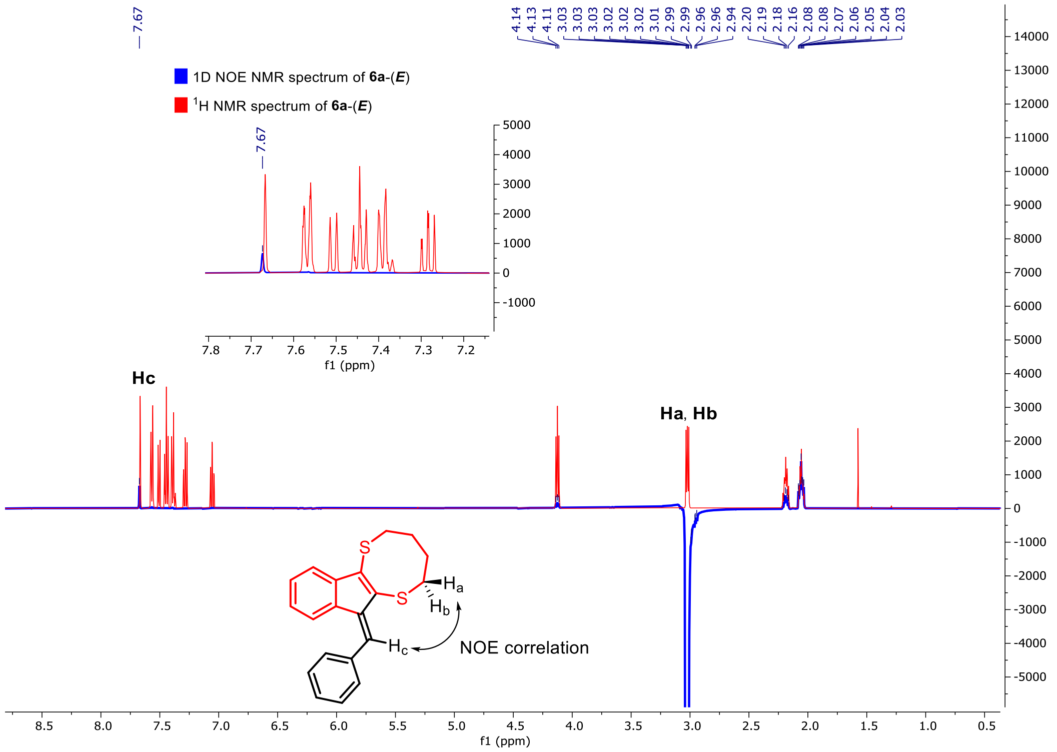

Figure S13. 1D NOE NMR experiment of **6a-(*E*)**; saturation of Ha, Hb (δ = 3.02)

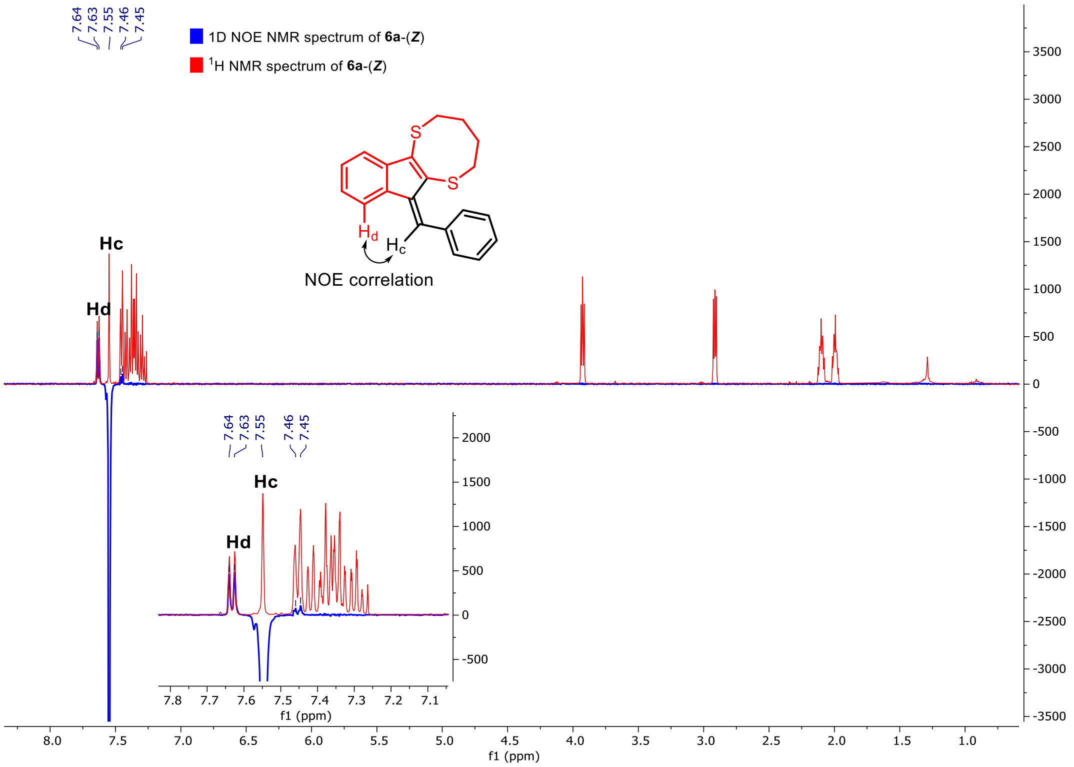

Figure S14. 1D NOE NMR experiment of **6a**-(***Z***); saturation of Hc ($\delta = 7.55$)

14. Control experiments

Scheme S4

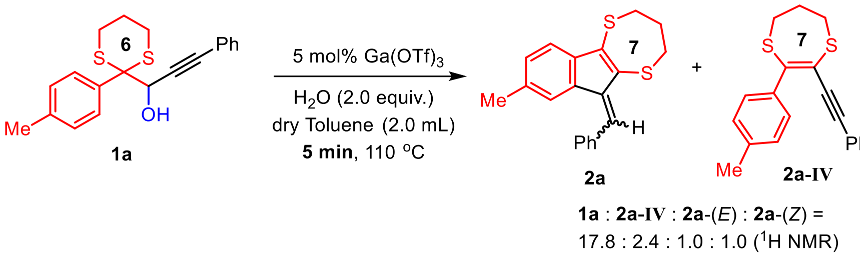

An oven-dried 15 mL screw-cap reaction vial equipped with a stirring bar was charged with propargyl alcohol (**1a**) (0.25 mmol, 85 mg) and water (0.50 mmol, 9.0 µL). Then the vial was brought into a glovebox. The reaction vial was charged with $Ga(OTf)_3$ (0.0125 mmol, 6.5 mg) and anhydrous toluene (2.0 mL). The vial was tightly closed, wrapped with a strip of Parafilm, and taken out of the glovebox. The reaction was stirred for 5 min at 110 °C in an oil bath. The vial was cooled to room temperature. The reaction mixture was taken into a 50 mL flask and the solvent was removed in a rotatory evaporator. The NMR sample was prepared from the crude mixture without further purification.

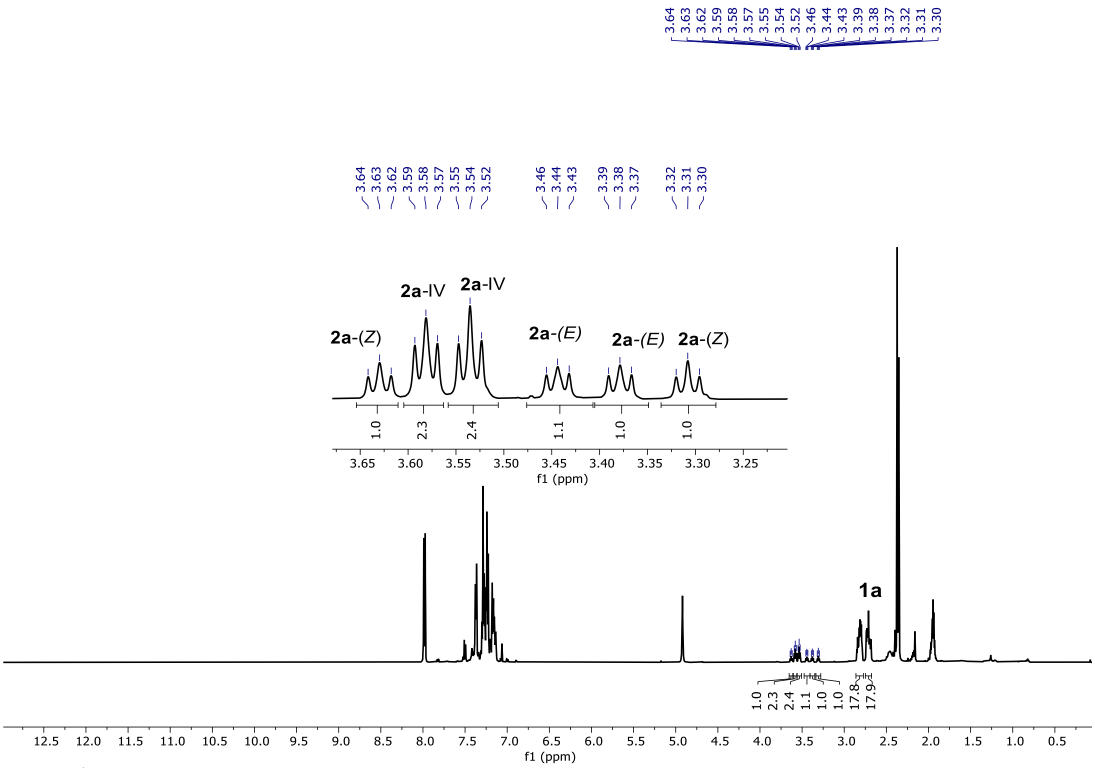

Figure S15. ^{1}H NMR (500 MHz, $CDCl_3$) spectra of the crude mixture of **2a**, **2a-IV** and **1a**.

Scheme S5.

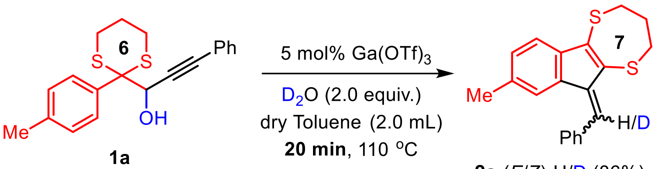

1a **2a**-(*E*/*Z*)-H/D (86%)

An oven-dried 15 mL screw-cap reaction vial equipped with a stirring bar was charged with propargyl alcohol (**1a**) (0.25 mmol, 85 mg) and D_2O (0.50 mmol, 9.0 µL). Then the vial was brought into a glovebox. The reaction vial was charged with $Ga(OTf)_3$ (0.0125 mmol, 6.5 mg) and anhydrous toluene (2.0 mL). The vial was tightly closed, wrapped with a strip of Parafilm, and taken out of the glovebox. The reaction was stirred for 20 min at 110 °C in an oil bath. The vial was cooled to room temperature. The reaction mixture was taken into a 50 mL flask and the solvent was removed in a rotatory evaporator. The remaining residue was dissolved in CH_2Cl_2 and mixed with silica gel (about 0.5-1.0 g). After evaporating CH_2Cl_2, the remaining silica gel was directly loaded onto a column and purified by flash chromatography on silica gel using 60:1 hexanes/ethyl acetate as eluent to yield the product **2a**-(*E*/*Z*)-H/D (69 mg, 86%, red oil) as a mixture of *E*/*Z* isomers. **2a**-(*E*)-H/D isomer is partially separated by flash chromatography on silica gel using hexanes as eluent. **2a**-(*E*)-H/D: ^{1}H-NMR (500 MHz, $CDCl_3$) δ 7.55−7.53 (m, 2H), 7.45−7.42 (m, 2H), 7.39−7.37 (m, 1H), 7.33 (s, 1H, CH, integral value: 40% less), 7.19 (d, J = 8.6 Hz, 1H, AB_{system}, δ_A), 7.10−7.09 (m, 1H), 7.02 (d, J = 8.6 Hz, 1H, AB_{system}, δ_B), 3.47 (t, J = 6.1 Hz, 2H), 3.40 (t, J = 6.1 Hz, 2H), 2.41−2.36 (m, 2H), 2.19 (s, 3H) ppm. ^{13}C{^{1}H} NMR (126 MHz, $CDCl_3$, APT) δ 140.8, 139.8, 136.6, 135.6, 134.7, 133.8, 131.5, 129.6, 129.4, 128.4, 128.3, 128.1, 123.0, 117.6, 33.0, 32.8, 31.0, 21.5 ppm.

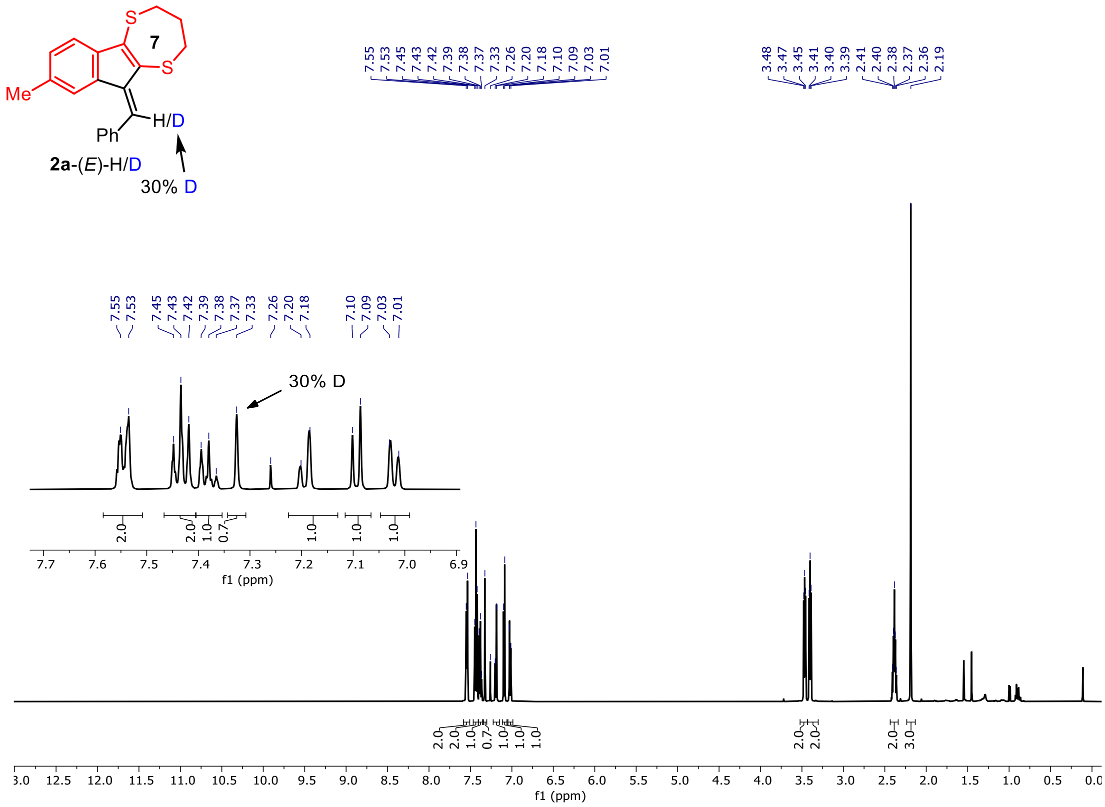

Figure S16. ^{1}H NMR (500 MHz, $CDCl_3$) spectra of **2a**-(*E*)-H/D.

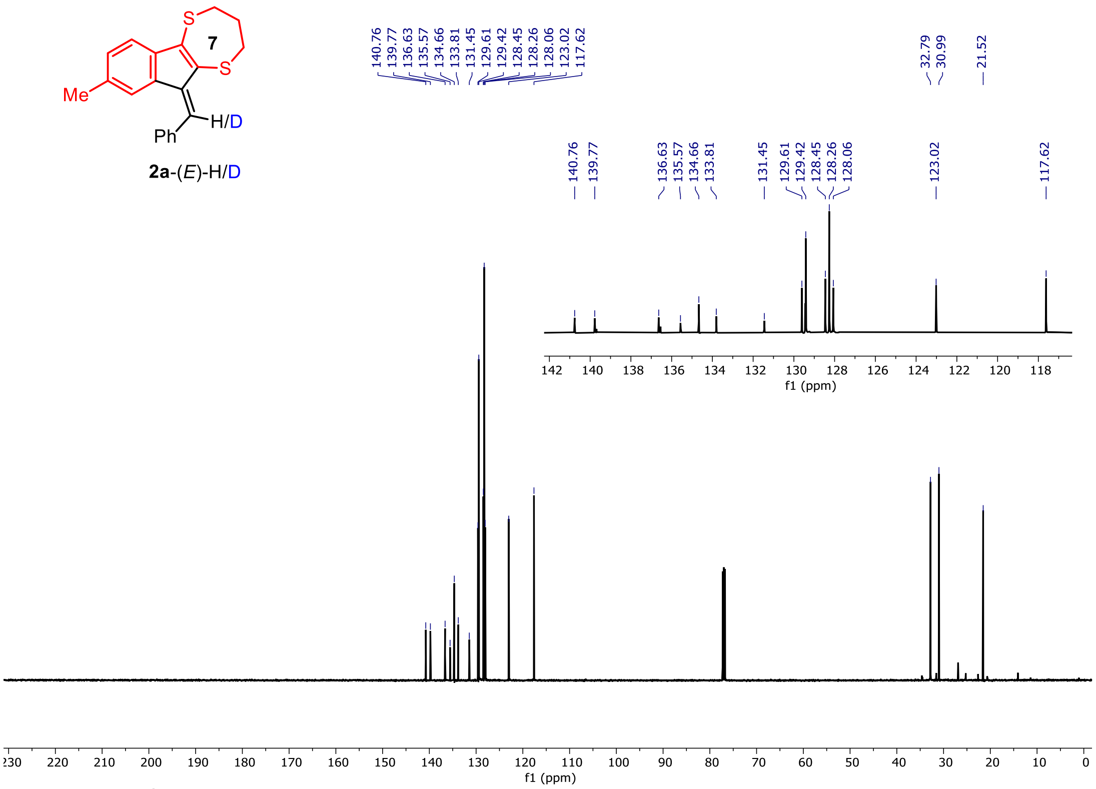

Figure S17. $^{13}C\{^{1}H\}$ NMR (126 MHz, $CDCl_3$) spectra of **2a**-(*E*)-H/D.

Scheme S6

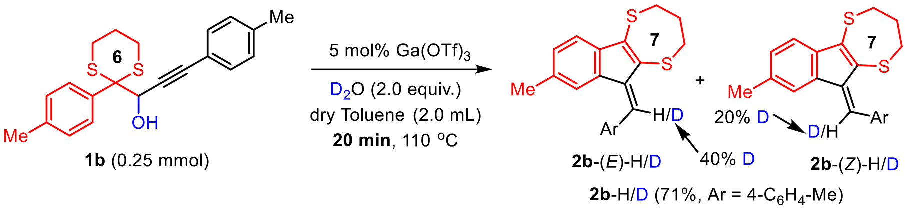

An oven-dried 15 mL screw-cap reaction vial equipped with a stirring bar was charged with propargyl alcohol (**1b**) (0.25 mmol, 89 mg) and D_2O (0.50 mmol, 9.0 µL). Then the vial was brought into a glovebox. The reaction vial was charged with $Ga(OTf)_3$ (0.0125 mmol, 6.5 mg) and anhydrous toluene (2.0 mL). The vial was tightly closed, wrapped with a strip of Parafilm, and taken out of the glovebox. The reaction was stirred for 20 min at 110 °C in an oil bath. The vial was cooled to room temperature. The reaction mixture was taken into a 50 mL flask and the solvent was removed in a rotatory evaporator. The remaining residue was dissolved in CH_2Cl_2 and mixed with silica gel (about 0.5-1.0 g). After evaporating CH_2Cl_2, the remaining silica gel was directly loaded onto a column and purified by flash chromatography on silica gel using 60:1 hexanes/ethyl acetate as eluent to yield the product **2b**-(*E*/*Z*)-H/D (60 mg, 71%, red oil) as a mixture of *E*/*Z* isomers. Isomers are partially separated by flash chromatography on silica gel using hexanes as eluent. **2b**-(*E*)-H/D: ^{1}H-NMR (500 MHz, $CDCl_3$) δ 7.47−7.45 (m, 2H), 7.33−7.32 (m, 1H), 7.30 (s, 1H, CH, integral value: 40% less), 7.23 (d, *J* = 7.9 Hz, 2H), 7.09 (d, *J* = 7.7 Hz, 1H, AB_{system}, δ_A), 7.02 (d, *J* = 7.6 Hz, 1H, AB_{system}, δ_B), 3.45 (t, *J* = 5.9 Hz, 2H), 3.38 (t, *J* = 5.9 Hz, 2H), 2.43 (s, 3H), 2.41−2.36 (m, 2H), 2.21 (s, 3H) ppm. $^{13C\{1H\}\ NMR}$ (125 MHz, $CDCl_3$, APT) δ 140.7, 139.2, 138.1, 135.2, 134.6, 133.9, 133.6, 131.8, 130.0, 129.5, 129.0, 128.4, 123.0, 117.6, 32.9, 32.8, 31.0, 21.6, 21.4 ppm. **2b**-(*Z*)-H/D: ^{1}H-NMR (500 MHz, $CDCl_3$) δ 7.40 (s, 1H, CH, integral value: 20% less), 7.34−7.32 (m, 3H), 7.21 (d, *J* = 8.0 Hz, 2H), 7.06 (bs, 2H), 3.65 (t, *J* = 6.0 Hz, 2H), 3.33 (t, *J* = 6.0 Hz, 2H), 2.40 (s, 6H), 2.22 (p, *J* = 6.0 Hz, 2H) ppm. ^{13}C{^{1}H} NMR (126 MHz, $CDCl_3$, APT) δ 139.2, 138.3, 138.1, 137.8, 137.1, 134.9, 133.6, 130.3, 128.6, 127.9, 125.5, 125.4, 118.8, 117.2, 34.1, 31.2, 30.2, 21.6, 21.5 ppm.

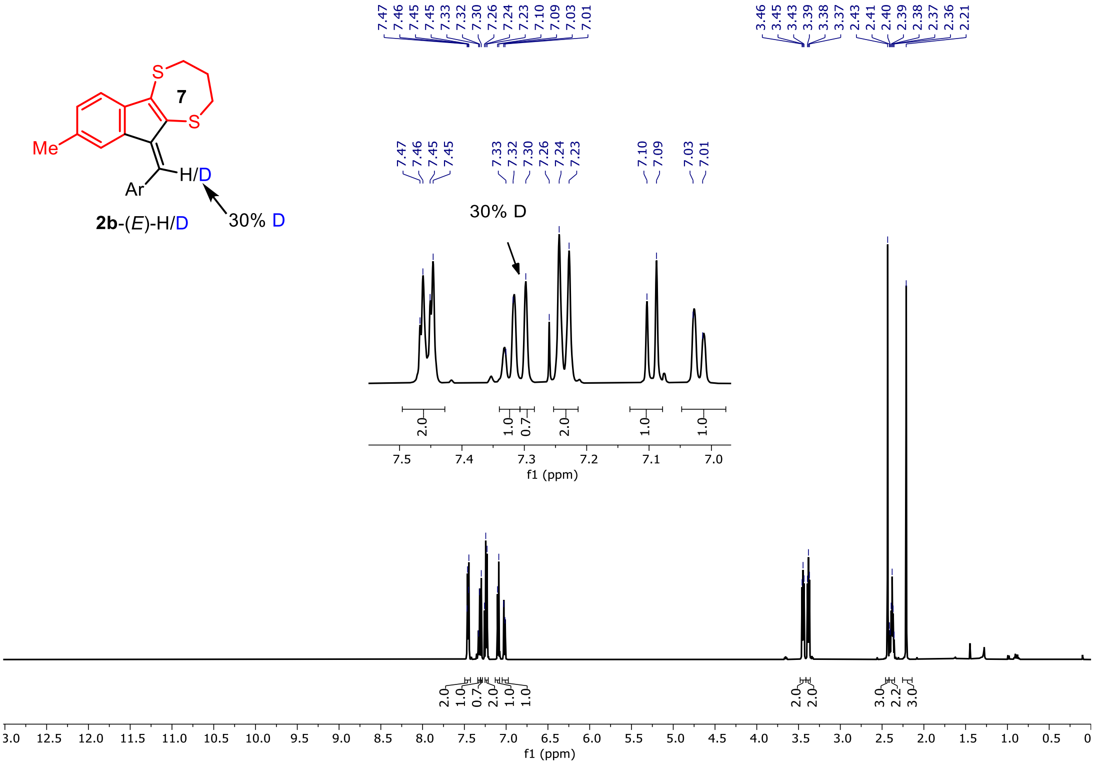

Figure S18. ^{1}H NMR (500 MHz, $CDCl_3$) spectra of **2b**-(*E*)-H/D

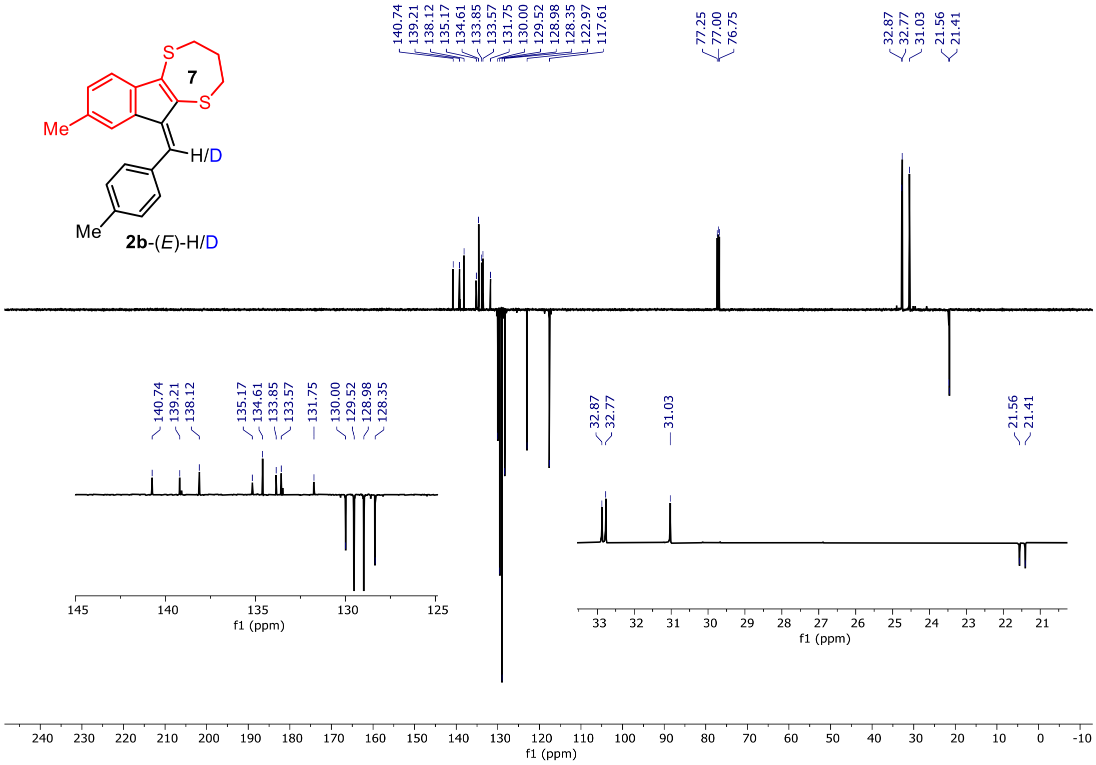

Figure S19. $^{13}C\{^1H\}$ NMR (126 MHz, $CDCl_3$, APT) spectra of **2b**-(*E*)-H/D.

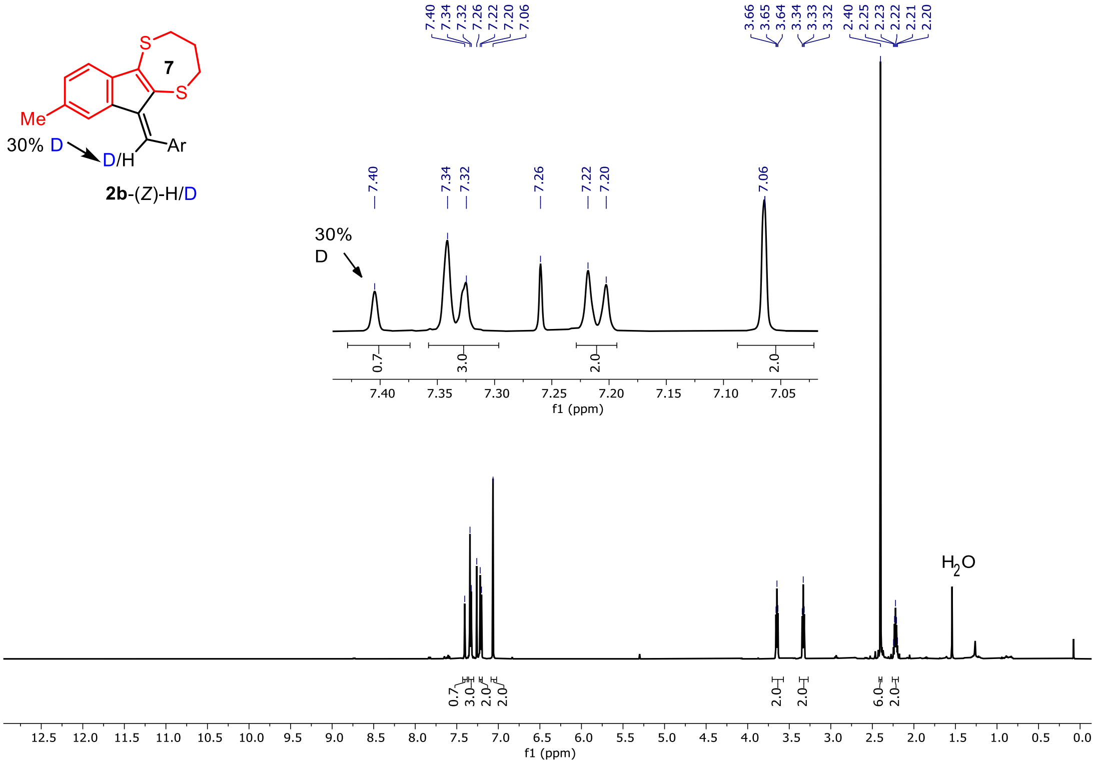

Figure S20. ^{1}H NMR (500 MHz, $CDCl_3$) spectra of **2b**-(*Z*)-H/D.

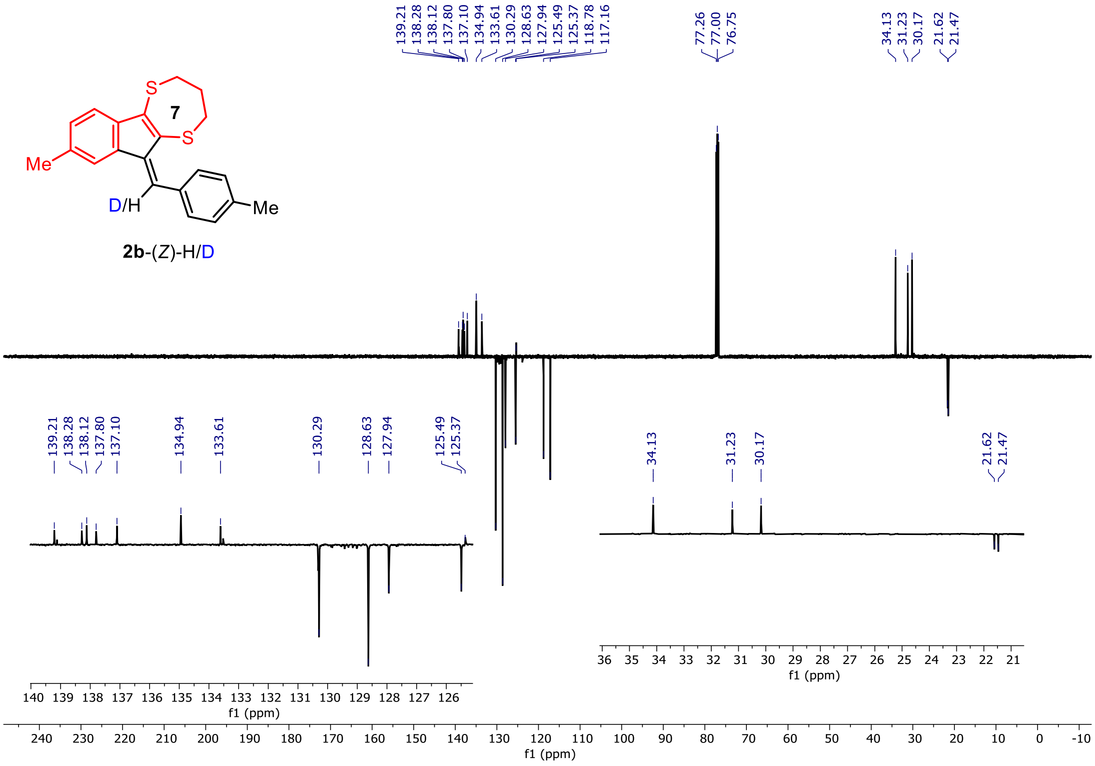

Figure S21. $^{13}C\{^1H\}$ NMR (126 MHz, $CDCl_3$, APT) spectra of **2b**-(*Z*)-H/D.

Scheme S7

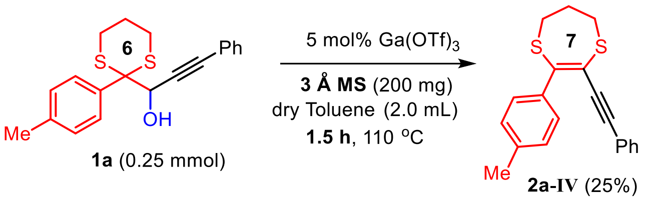

An oven-dried 15 mL screw-cap reaction vial equipped with a stirring bar was charged with propargyl alcohol (**1a**) (0.25 mmol, 85 mg). Then the vial was brought into a glovebox. The reaction vial was charged with $Ga(OTf)_3$ (0.0125 mmol, 6.5 mg), freshly activated molecular sieves (200 mg, 3 Å) and anhydrous toluene (2.0 mL). The vial was tightly closed, wrapped with a strip of Parafilm, and taken out of the glovebox. The reaction was stirred for 1.5 h at 110 °C in an oil bath. The vial was cooled to room temperature. The reaction mixture was taken into a 50 mL flask and the solvent was removed in a rotatory evaporator. The remaining residue was dissolved in CH_2Cl_2 and mixed with silica gel (about 0.5-1.0 g). After evaporating CH_2Cl_2, the remaining silica gel was directly loaded onto a column and purified by flash chromatography on silica gel using 60:1 hexanes/ethyl acetate and then 10:1 hexanes/ethyl acetate as eluent to isolate the product **2a-IV** (20 mg, 25%, yelowish oil) and propargyl alcohol **1a** (55 mg, recovered). **2a-(*E*)**: ^{1}H-NMR (500 MHz, $CDCl_3$) δ 7.50 (d, *J* = 8.1 Hz, 2H), 7.21−7.20 (m, 3H), 7.15−7.14 (m, 4H), 3.59 (t, *J* = 5.9 Hz, 2H), 3.55 (t, *J* = 6.0 Hz, 2H), 2.36 (s, 3H), 2.19 (p, *J* = 6.0 Hz, 2H) ppm. ^{13}C{^{1}H} NMR (126 MHz, $CDCl_3$, APT) δ 143.9, 138.5, 137.4, 131.1, 129.9, 128.5, 128.1, 127.9, 123.3, 111.3, 92.0, 89.3, 32.6, 32.3, 29.4, 21.3 ppm.

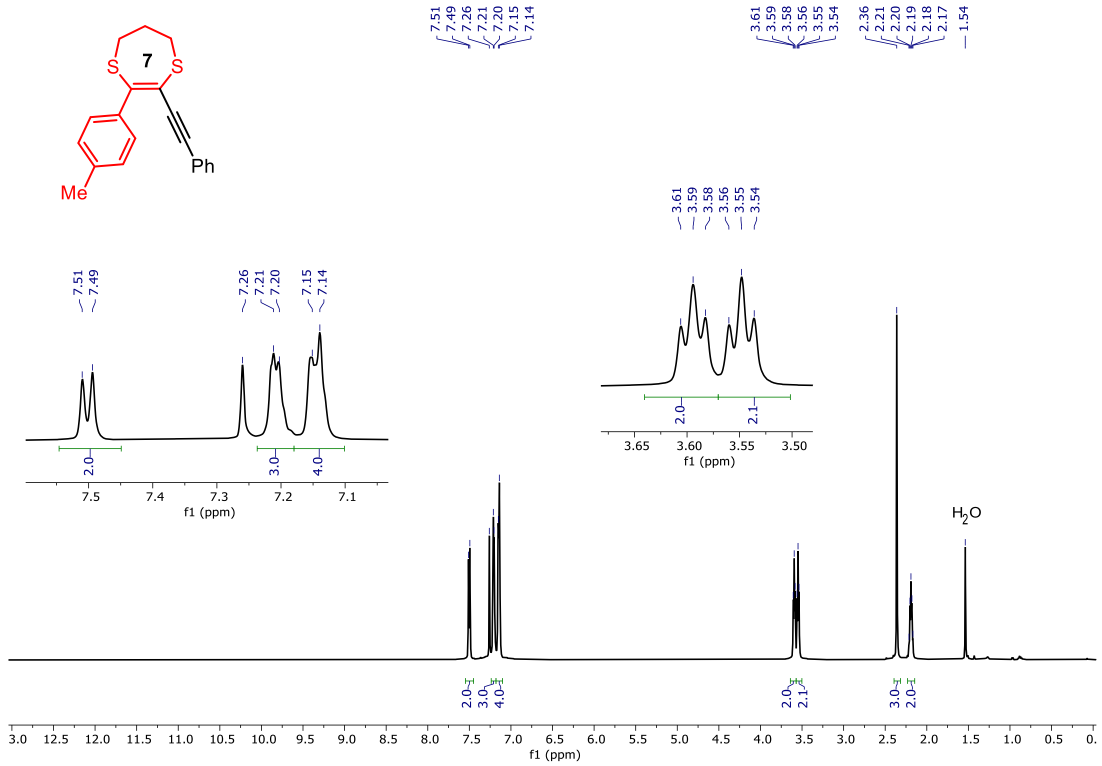

Figure S22. ^{1}H NMR (500 MHz, $CDCl_3$) spectra of **2a-IV**.

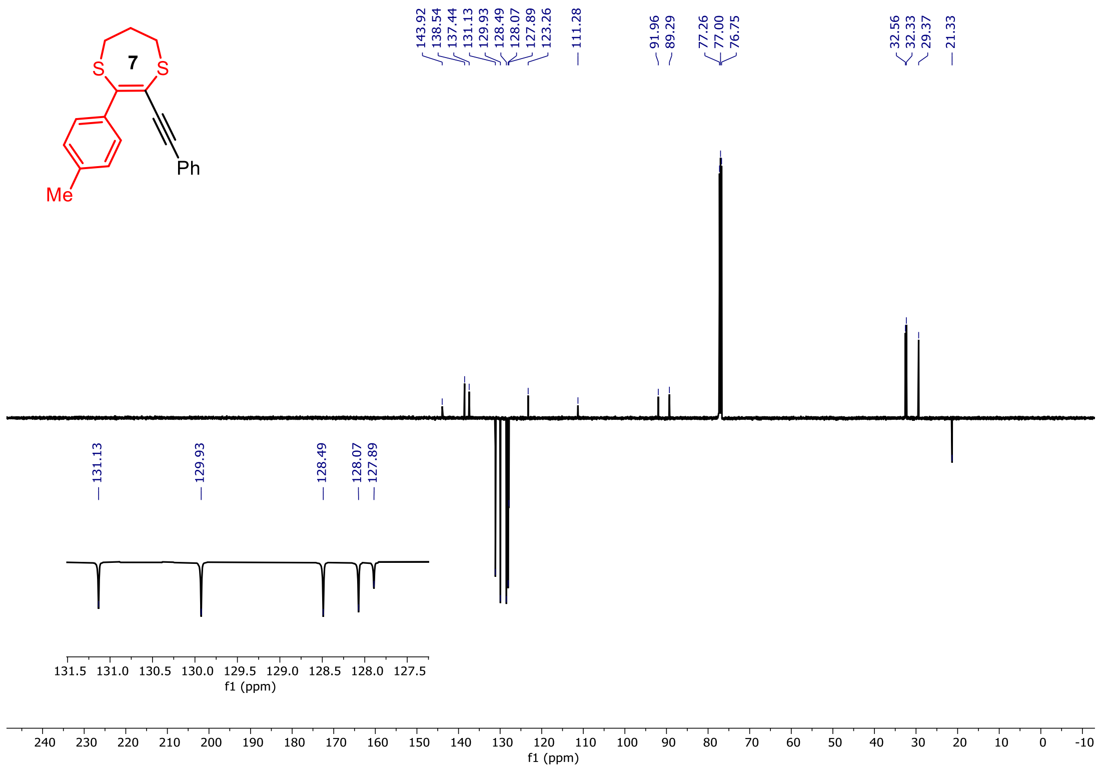

Figure S23. ^{13}C NMR (125 MHz, $CDCl_3$, APT) spectra of **2a-IV**.

Scheme S8.

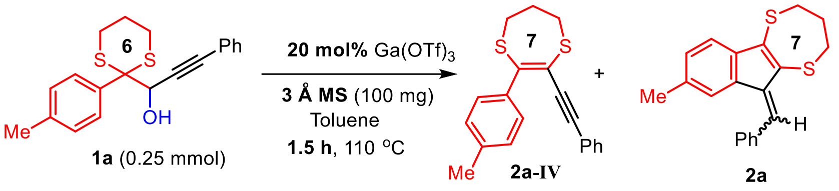

2a-IV + **2a**-(*E*/*Z*) (31%)
2a-IV:**2a**-(*E*):**2a**-(*Z*) = 4.2:1.4:1 (^{1}H NMR)

An oven-dried 15 mL screw-cap reaction vial equipped with a stirring bar was charged with propargyl alcohol (**1a**) (0.25 mmol, 85 mg). Then the vial was brought into a glovebox. The reaction vial was charged with $Ga(OTf)_3$ (0.05 mmol, 26 mg), freshly activated molecular sieves (100 mg, 3 Å) and anhydrous toluene (2.0 mL). The vial was tightly closed, wrapped with a strip of Parafilm, and taken out of the glovebox. The reaction was stirred for 1.5 h at 110 °C in an oil bath. The vial was cooled to room temperature. The reaction mixture was taken into a 50 mL flask and the solvent was removed in a rotatory evaporator. The remaining residue was dissolved in CH_2Cl_2 and mixed with silica gel (about 0.5-1.0 g). After evaporating CH_2Cl_2, the remaining silica gel was directly loaded onto a column and purified by flash chromatography on silica gel using 60:1 hexanes/ethyl acetate and then 10:1 hexanes/ethyl acetate as eluent to isolate the mixture of products **2a-IV** and **2a** (25 mg, 31%, red oil) and propargyl alcohol **1a** (43 mg, recovered).

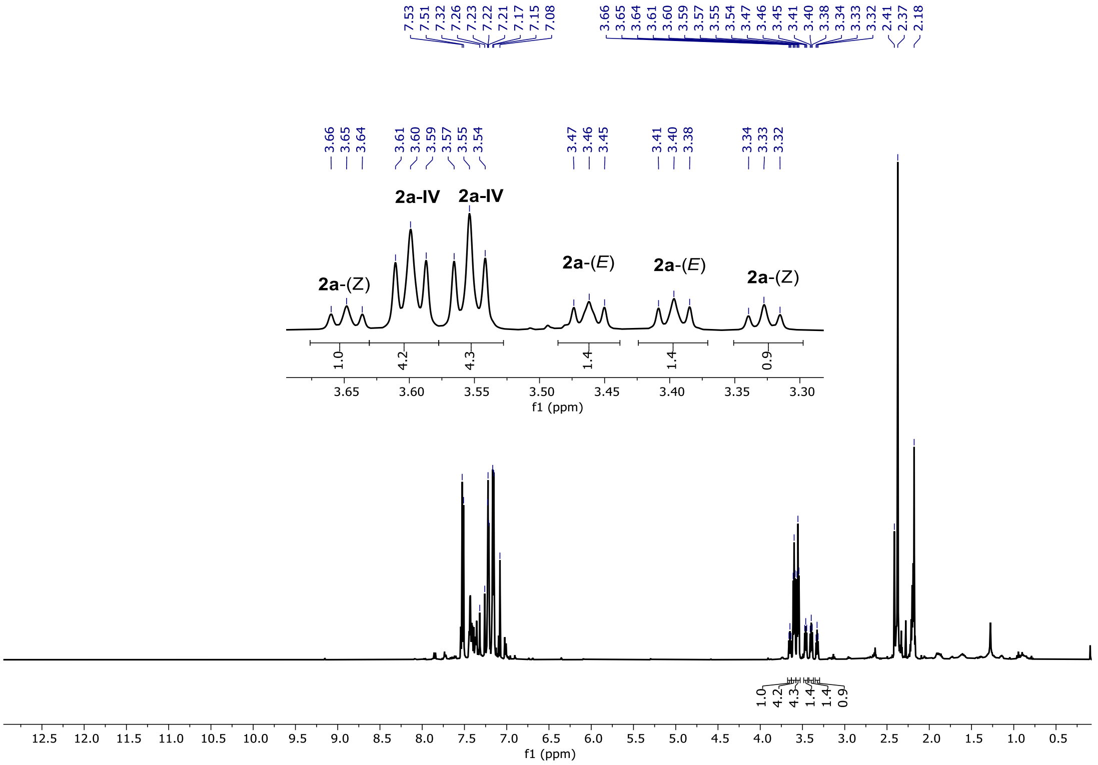

Figure S24. ^{1}H NMR (500 MHz, $CDCl_3$) spectra of the crude mixture of **2a**, **2a-IV**.

15. X-ray Crystallography Data for compound 2a-(*Z*), 2b-(*E*), 4b-(*E*) and 2a'

Crystals of **2a**-(*Z*), **2b**-(*E*), **4b**-(*E*) and **2a'**were mounted on a micromount and attached to a goniometer head on a Bruker D8 VENTURE diffractometer equipped with PHOTON100 detector and was measured with graphite monochromated Mo-Kα radiation (λ = 0.71073 Å) using 1.0° of Ω and ϕ rotation frames at room temperature (297 K). The structure has been solved by intrinsic method SHELXS-1997[S4] and refined using SHELXL2014/7.[S5] Molecular drawings are generated using OLEX2. Ver. 1.2-dev.[S6]

Crystallization: Crystals of compound **2a'** was grown from the solvent chloroform, crystals of **2a**-(*Z*), **2b**-(*E*) and **4b**-(*E*) were grown from the mixture of solvents hexane and dichloromethane (1:1, v/v).

Table S4. Crystal data and structure refinement for **2a-(*Z*)** and ORTEP diagram of **2a-(*Z*)** with ellipsoid contour at 50% probability level

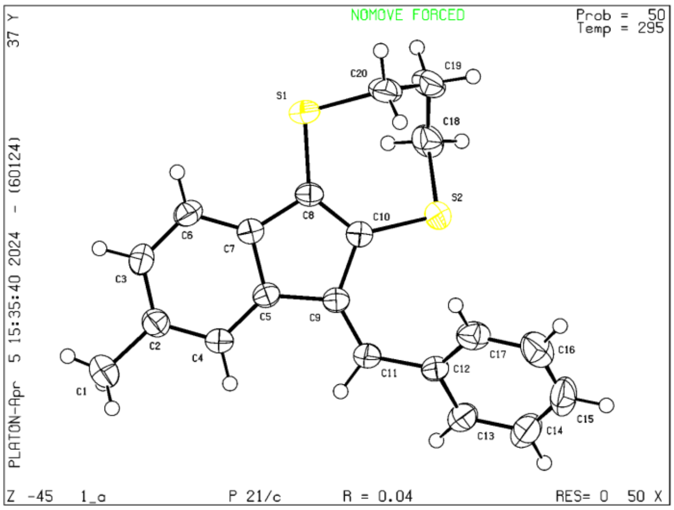

Chemical formula	$C_{20}H_{18}S_2$
Formula weight	322.46
Temperature/K	295(2)
Crystal system	monoclinic
Space group	P1 21/c1
a/Å	11.6152(9)
b/Å	8.4178(7)
c/Å	17.2220(14)
α/°	90
β/°	102.156(2)
γ/°	90
Volume/Å^3	1646.1(2)
Z	4
ρ_{calc}g/cm^3	1.301
μ/mm^{-1}	0.317
F(000)	680
Crystal size/mm^3	0.040 × 0.150 × 0.450
Radiation	Mo Kα (λ = 0.71073)
2Θ range for data collection/°	2.42 to 27.16
Index ranges	$-14 \le h \le 14$, $-10 \le k \le 10$, $-22 \le l \le 20$
Reflections collected	20294
Independent reflections	3642 [R_{int} = 0.00403]
Data/restraints/parameters	3642/0/272
Goodness-of-fit on F^2	1.013
Final R indexes [I>=2σ (I)]	R_1 = 0.0401, wR_2 = 0.0860
Final R indexes [all data]	R_1 = 0.0646, wR_2 = 0.0961
Largest diff. peak/hole / e Å^{-3}	0.259/-0.213

Table S5. Crystal data and structure refinement for **2a-(*E*)** and ORTEP diagram of **2a-(*E*)** with ellipsoid contour at 50% probability level

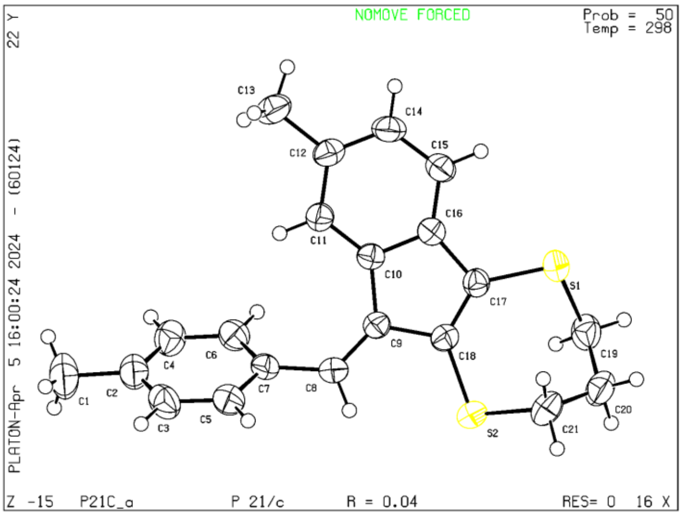

Chemical formula	$C_{21}H_{20}S_2$
Formula weight	336.49 g/mol
Temperature/K	298 (0)
Crystal system	monoclinic
Space group	P1 21/c 1
a/Å	16.909 (18)
b/Å	9.3562 (7)
c/Å	11.5194 (9)
α/°	90
β/°	105.278 (2)
γ/°	90
Volume/Å^3	1758.0(2) Å3
Z	4
ρ_{calc} g/cm^3	1.271
μ/mm^{-1}	0.300
F(000)	712
Crystal size/mm^3	0.050 x 0.200 x 0.560
2Θ range for data collection/°	2.50 to 26.45°
Index ranges	-21<=h<=21, -11<=k<=11, -14<=l<=14
Reflections collected	59942
Independent reflections	3623 [R(int) = 0.0417]
Data/restraints/parameters	3623 / 0 / 211
Goodness-of-fit on F^2	1.035
Final R indexes [I>=2σ (I)]	R_1 = 0.0377, wR_2 = 0.0888
Final R indexes [all data]	R_1 = 0.0529, wR_2 = 0.0966
Largest diff. peak/hole / e Å^{-3}	0.241 and -0.218

Table S6. Crystal data and structure refinement for **4b-**(*E*) and ORTEP diagram of **4b-**(*E*) with ellipsoid contour at 50% probability level

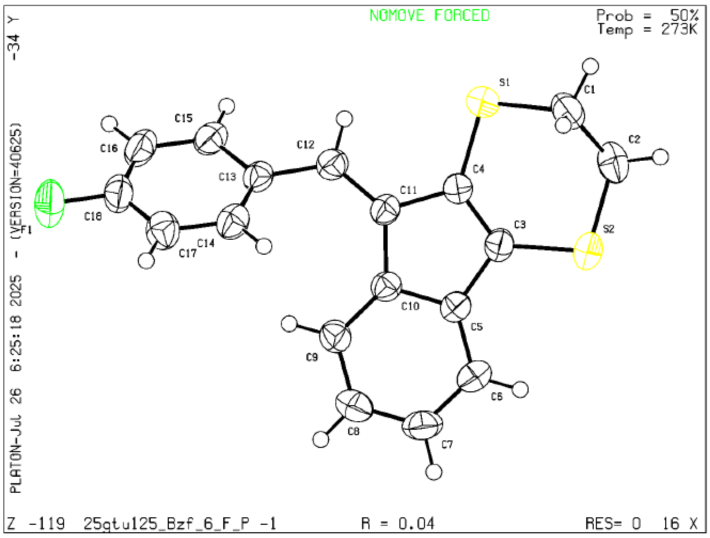

Chemical formula	$C_{18}H_{13}FS_2$
Formula weight	312.40
Temperature/K	273 (2)
Crystal system	Triclinic
Space group	P-1
a/Å	7.0909(13)
b/Å	9.0341(16)
c/Å	12.083(2)
α/°	73.722(3)
β/°	88.878(3)
γ/°	77.963(2)
Volume/Å^3	726.0(2)
Z	2
ρ_{calc}g/cm^3	1.429
μ/mm^{-1}	0.367
Crystal size/mm^3	0.24 x 0.12 x 0.10
2Θ range for data collection/°	1.76 to 25.00
Index ranges	-8<=h<=8, -10<=k<=10, -14<=l<=14
Reflections collected	9269
Independent reflections	2566 [R(int) = 0.0362]
Data/restraints/parameters	2566/ 0 / 191
Goodness-of-fit on F^2	1.031
Final R indexes [I>=2σ (I)]	R_1 = 0.0364
wR_2 [all data]	wR2 = 0.0962

Table S7. Crystal data and structure refinement for **2a'** and ORTEP diagram of **2a'** with ellipsoid contour at 50% probability level

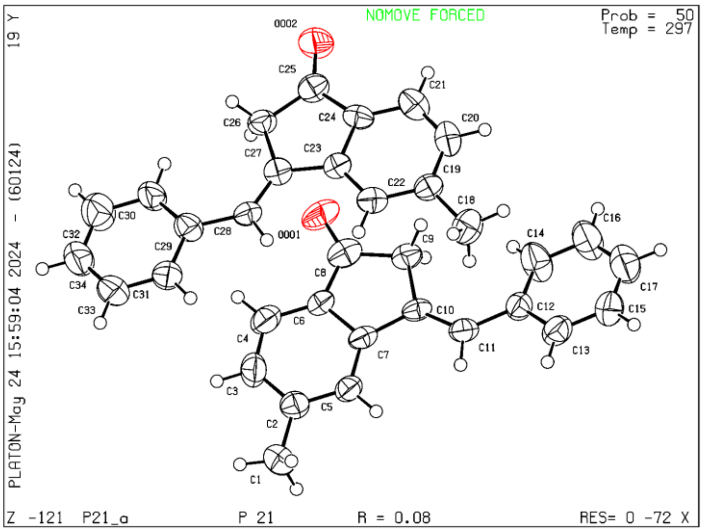

Chemical formula	$C_{17}H_{14}O$
Formula weight	234.28
Temperature/K	297 (0)
Crystal system	monoclinic
Space group	P1 21 1
a/Å	9.0335 (19)
b/Å	5.8534 (12)
c/Å	24.457 (5)
α/°	90
β/°	98.889 (6)
γ/°	90
Volume/Å^3	1277.7 (5)
Z	4
ρ_{calc}g/cm^3	1.218
μ/mm^{-1}	0.074
F(000)	496
Crystal size/mm^3	0.050 × 0.180 × 0.610
2Θ range for data collection/°	2.28 to 25.54
Index ranges	-10 ≤ h ≤ 10, -7 ≤ k ≤ 7, -29≤ l ≤ 29
Reflections collected	37908
Independent reflections	4695 [R_{int} = 0.0741]
Data/restraints/parameters	4695/1/328
Goodness-of-fit on F^2	1.078
Final R indexes [I>=2σ (I)]	R_1 = 0.0849, wR_2 = 0.2029
Final R indexes [all data]	R_1 = 0.1290, wR_2 = 0.2202
Largest diff. peak/hole / e Å^{-3}	0.233/-0.223

16. References

(S1) Ismailoglu, E.; Mert, Z.; Dinc, M.; Kaya, K.; Yucel, B. Synthesis of 3-Amino-4-iodothiophenes through Iodocyclization of 1-(1,3-Dithian-2-yl)propargylamines. *Eur. J. Org. Chem.* **2021**, 4107–4124.

(S2) Dinc, M.; Ismailoglu, E.; Mert, Z.; Kaya, K.; Tayanc, M.; Yucel, B. Base-Mediated Rearrangement of α-Dithioacetyl Propargylamines via Expansion of Dithioacetyl Ring: Synthesis of Medium-Sized *S,S*-Heterocycles. *Org. Lett.* **2023**, *25*, 4028-4032.

(S3) Li, F.; Korenaga, T.; Nakanishi, T.; Kikuchi, J.; Terada, M. Chiral Phosphoric Acid Catalyzed Enantioselective Ring Expansion Reaction of 1,3-Dithiane Derivatives: Case Study of the Nature of Ion-Pairing Interaction. *J. Am. Chem. Soc.* **2018**, *140*, 2629– 2642.

(S4) Sheldrick, G. M. SHELXS-97, Program for Crystal Structure Solution, University of Göttingen, Göttingen, 1997.

(S5) Sheldrick, G. M. Foundations of Crystallography. Acta Crystallogr. Sect. A **2008**, *64*, 112–122.

(S6) Dolomanov, O. V.; Bourhis, L. J.; Gildea, R. J.; Howard, J. A. K.; Puschmann, H. OLEX2: a complete structure solution, refinement and analysis program. *J. Appl. Crystallogr.* **2009**, 42, 339–341.

Figure S25. ^{1}H NMR ($CDCl_3$, 500 MHz) spectrum **S1d**

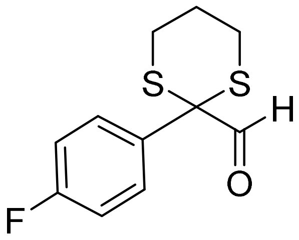

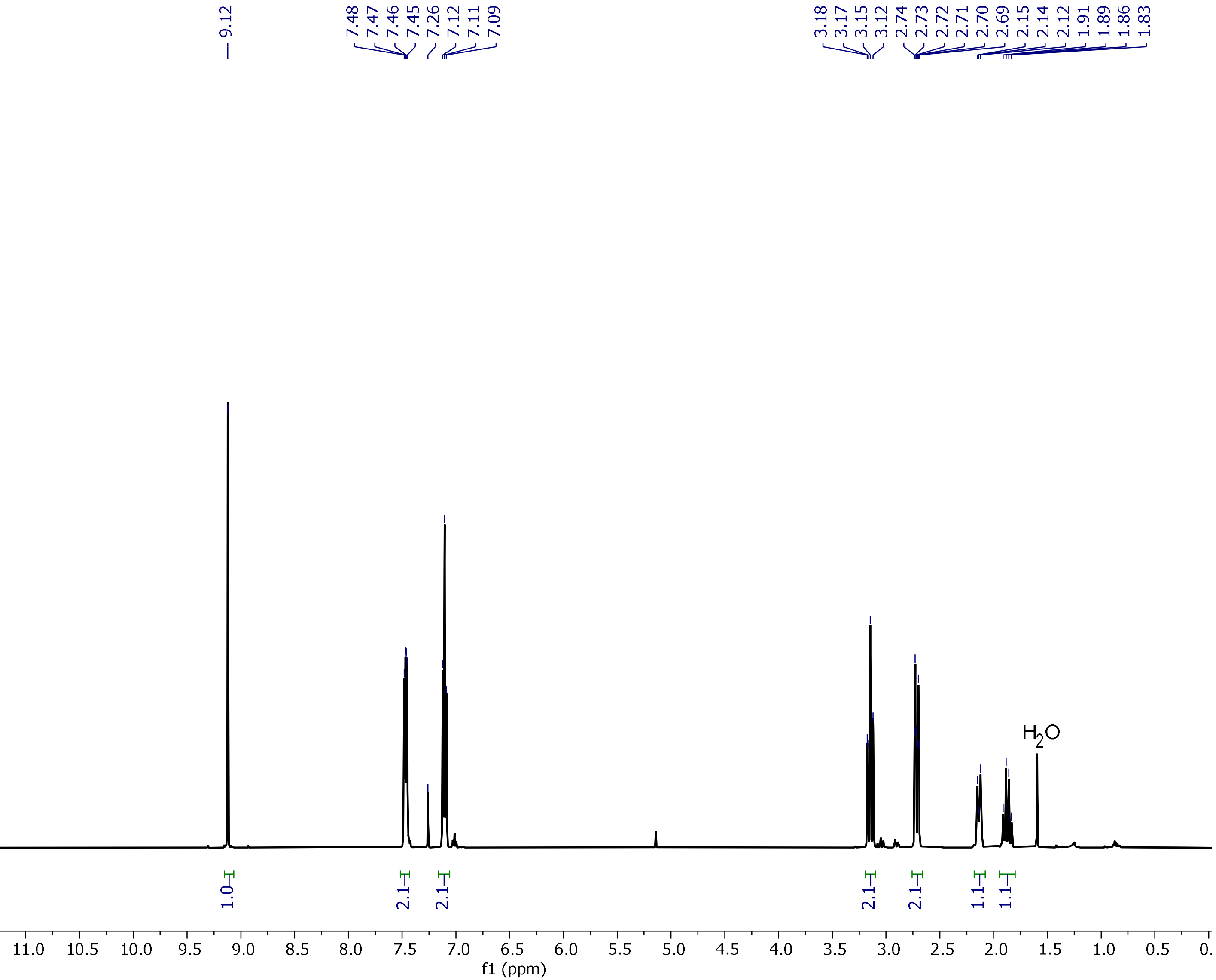

Figure S26. $^{13}C\{^1H\}$ NMR (126 MHz, $CDCl_3$, APT) spectrum **S1d**

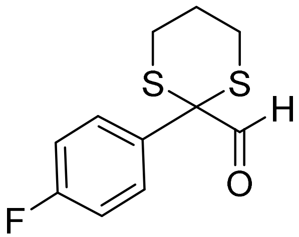

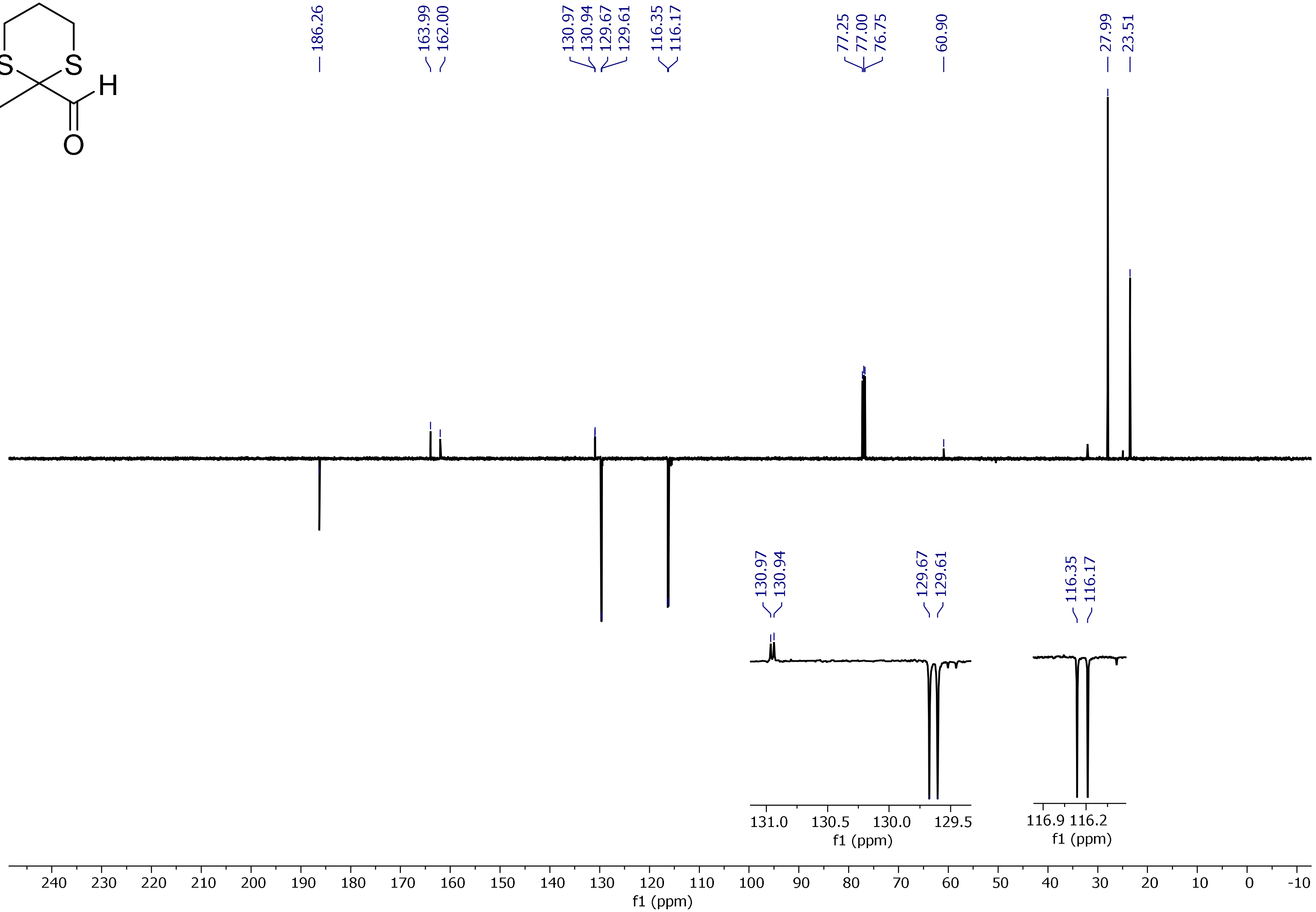

Figure S27. ^1H NMR ($CDCl_3$, 500 MHz) spectrum **S1g**

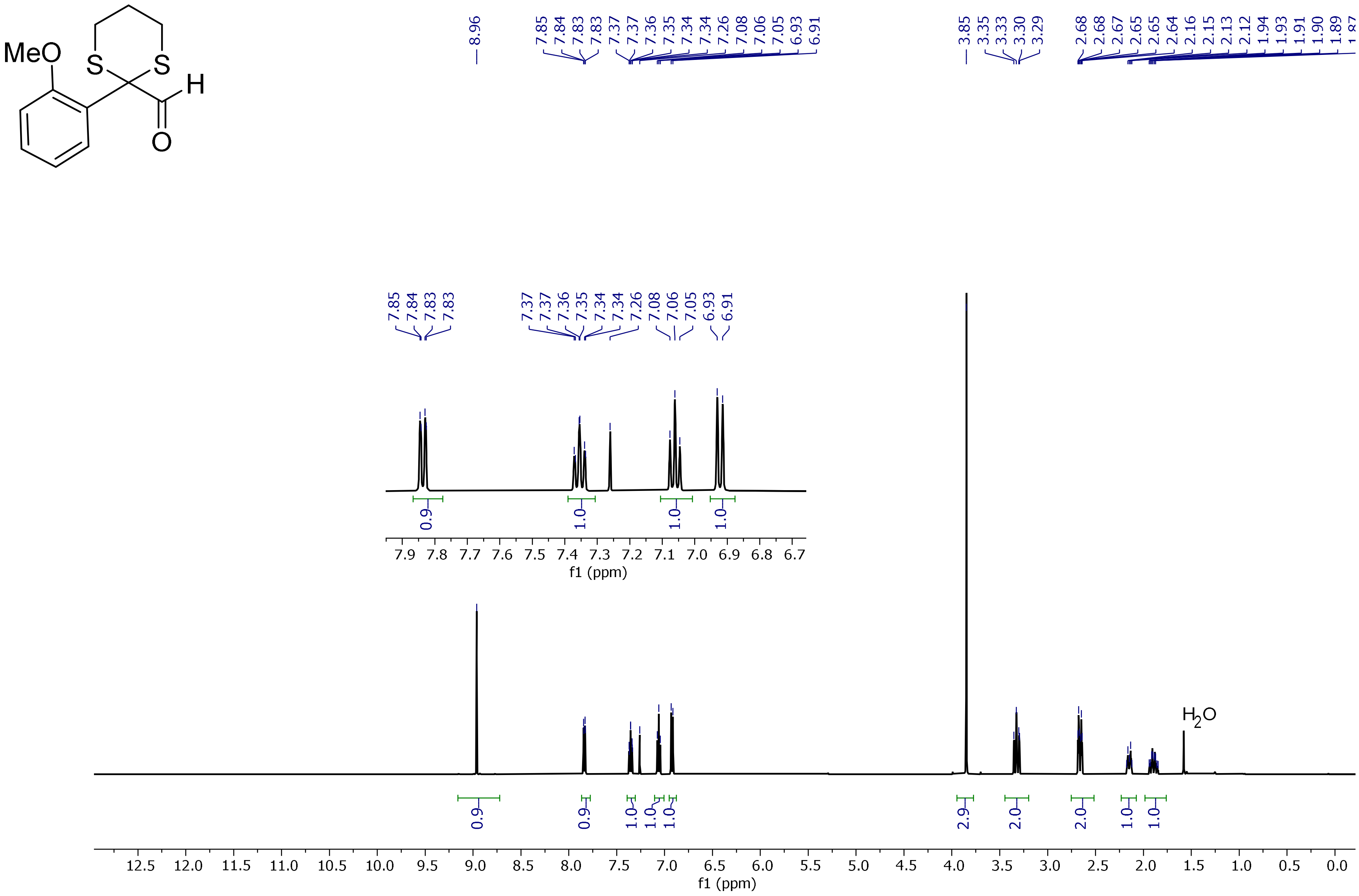

Figure S28. $^{13}C\{^{1}H\}$ NMR (126 MHz, $CDCl_3$, APT) spectrum **S1g**

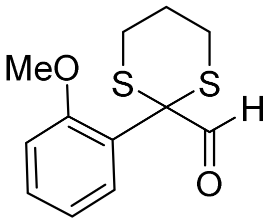

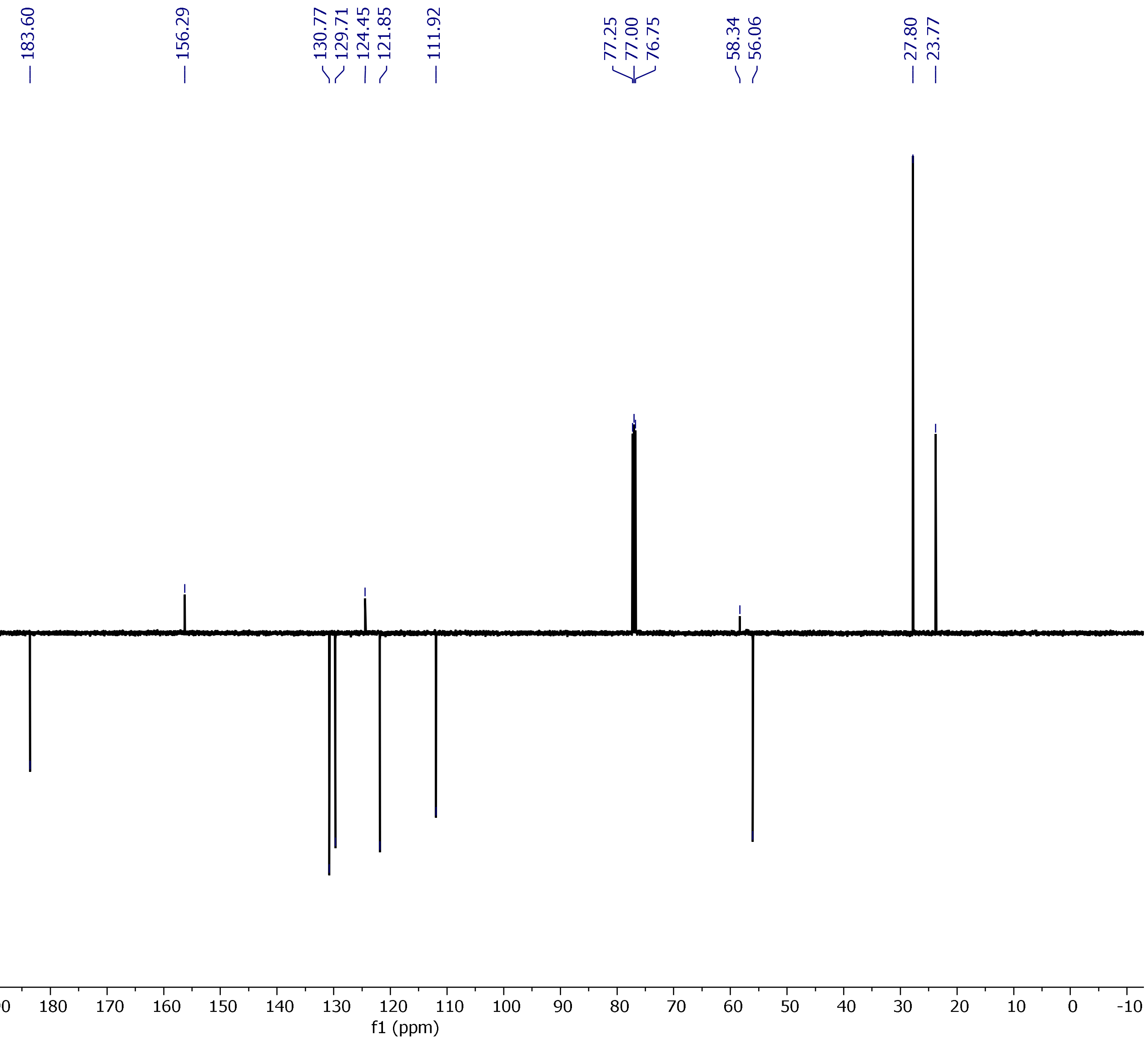

Figure S29. ^{1}H NMR ($CDCl_3$, 500 MHz) spectrum **S1h**

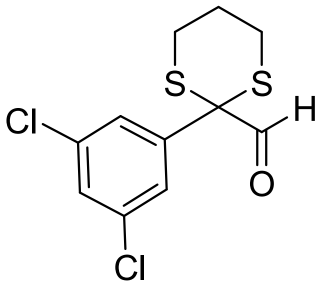

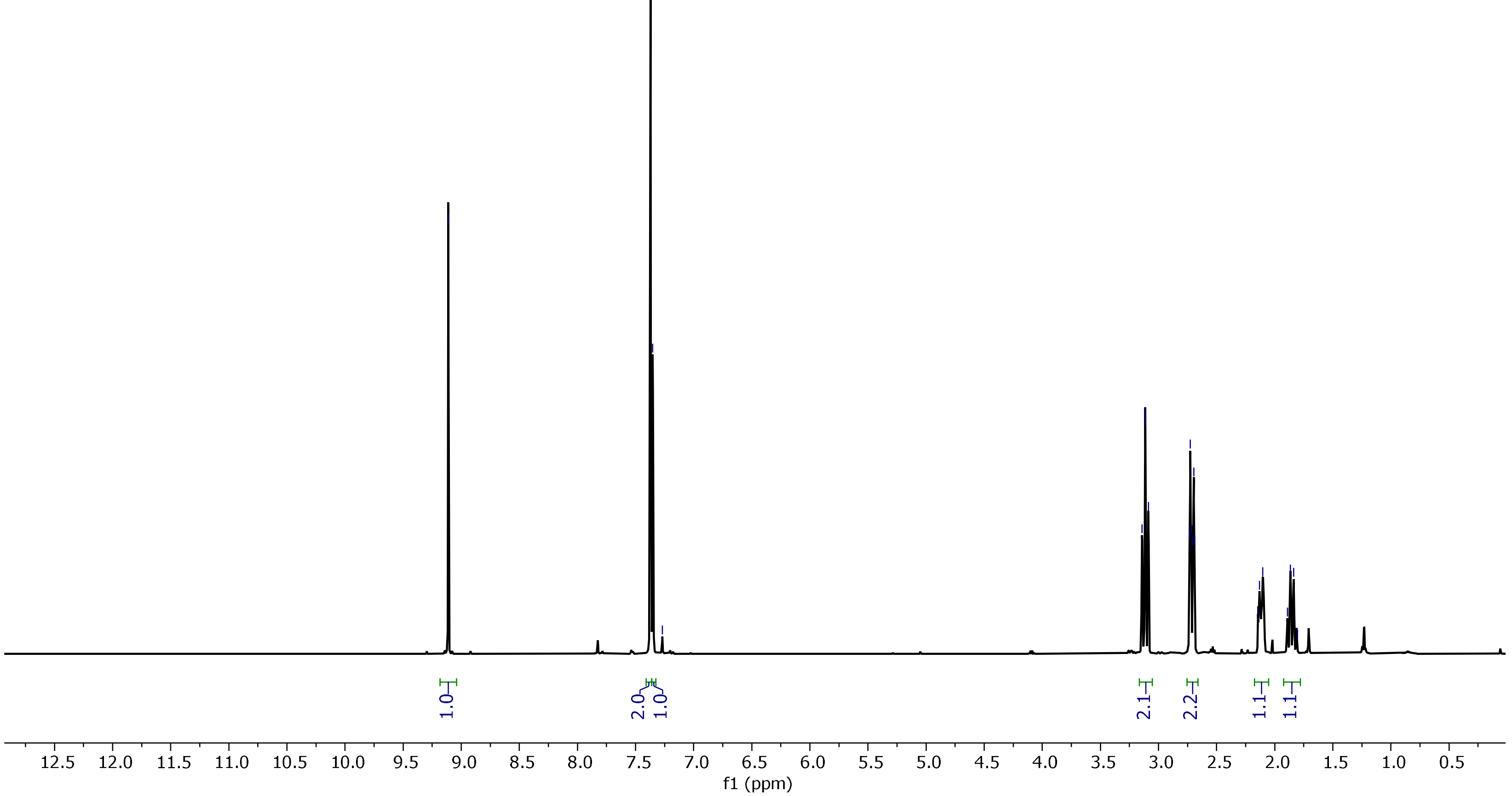

Figure S30. $^{13}C\{^{1}H\}$ NMR (126 MHz, $CDCl_3$, APT) spectrum **S1g**

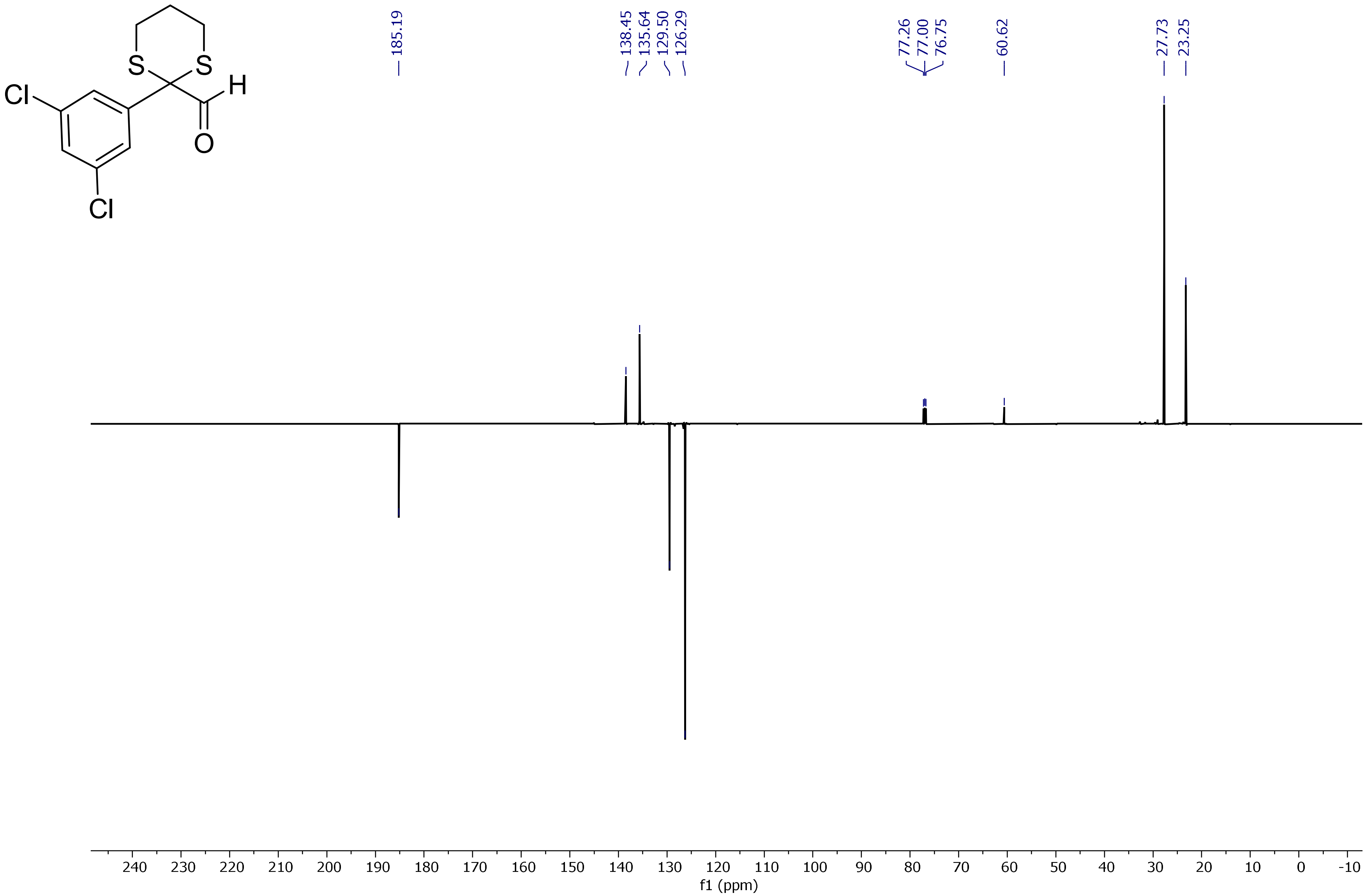

Figure S31. ^{1}H NMR ($CDCl_3$, 500 MHz) spectrum **S3b**

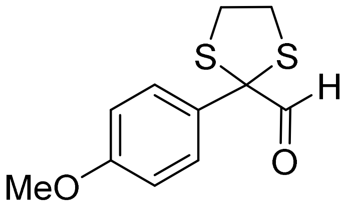

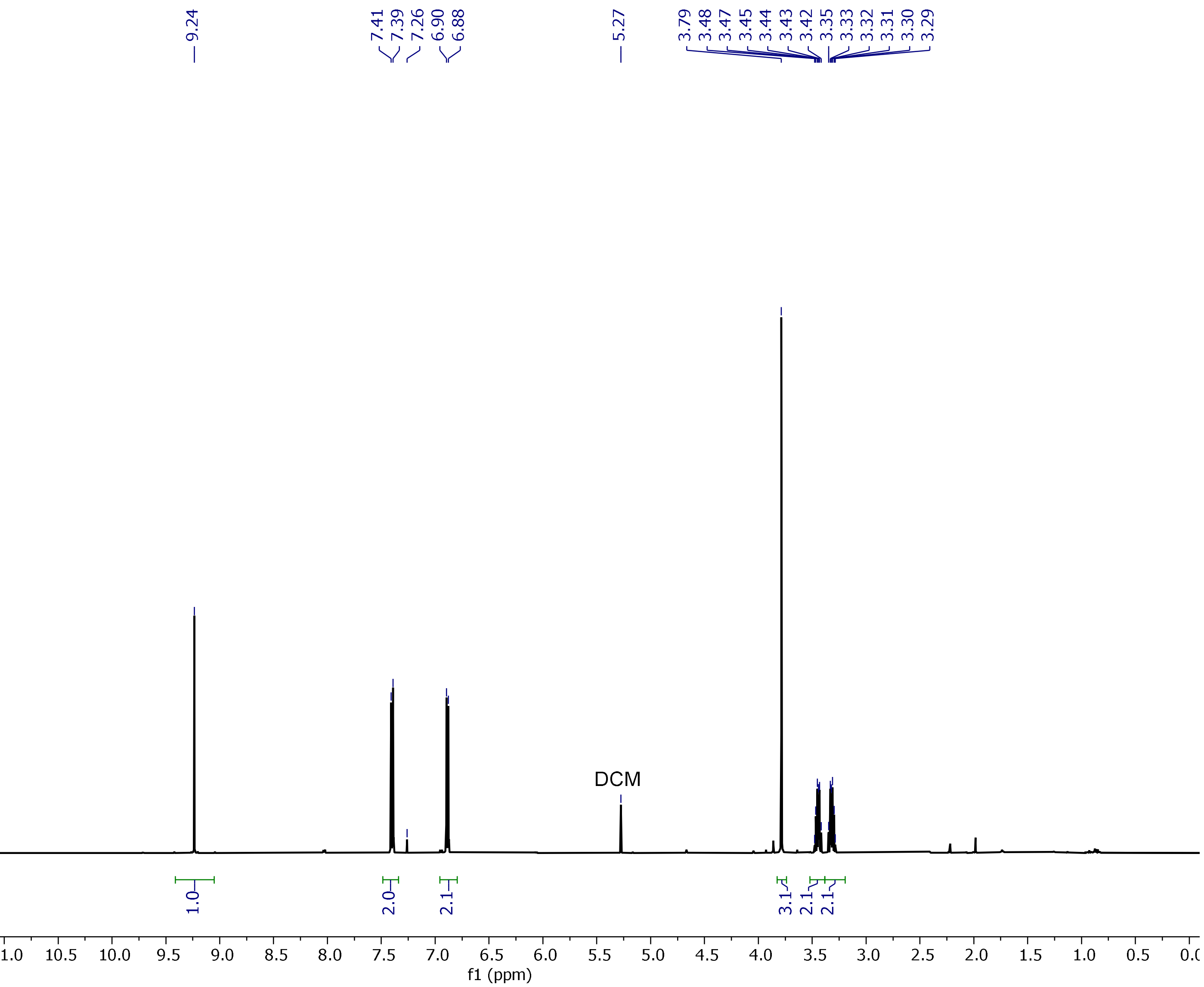

Figure S32. $^{13}C\{^1H\}$ NMR (126 MHz, $CDCl_3$, APT) spectrum **S3b**

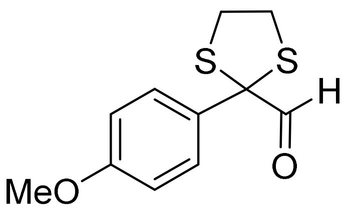

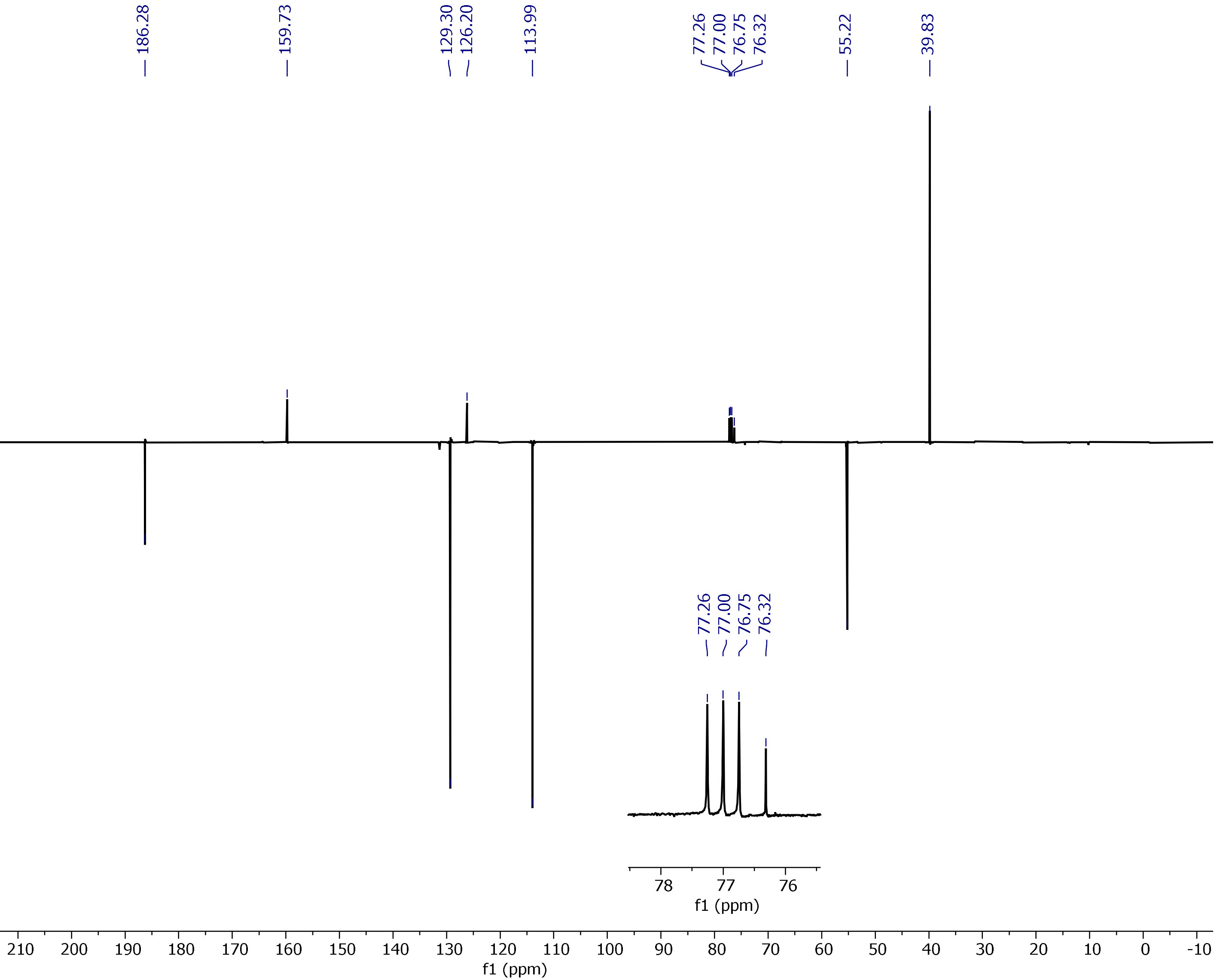

Figure S33. ^{1}H NMR ($CDCl_3$, 500 MHz) spectrum **S3c**

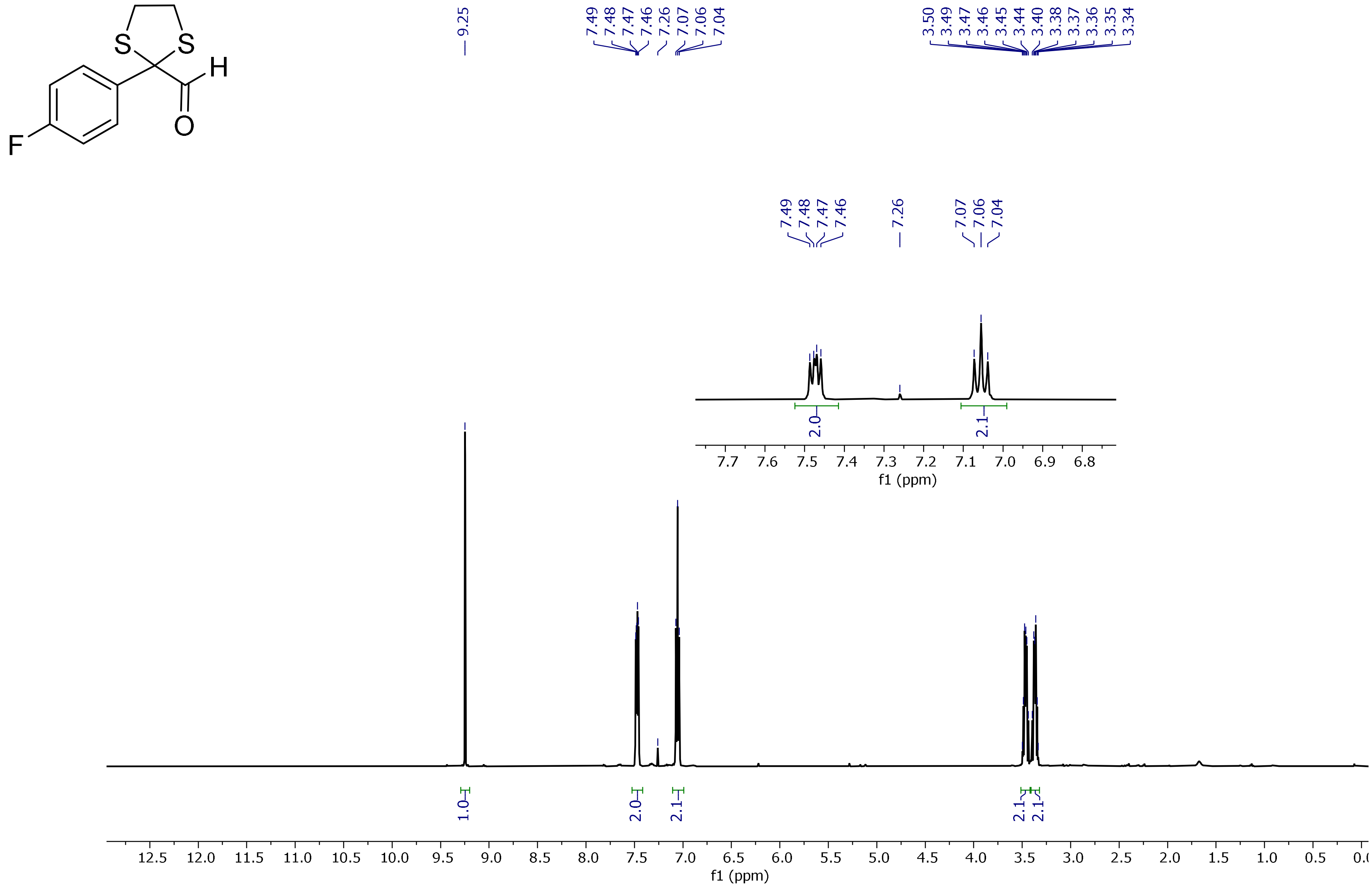

Figure S34. $^{13}C\{^{1}H\}$ NMR (126 MHz, $CDCl_3$, APT) spectrum **S3c**

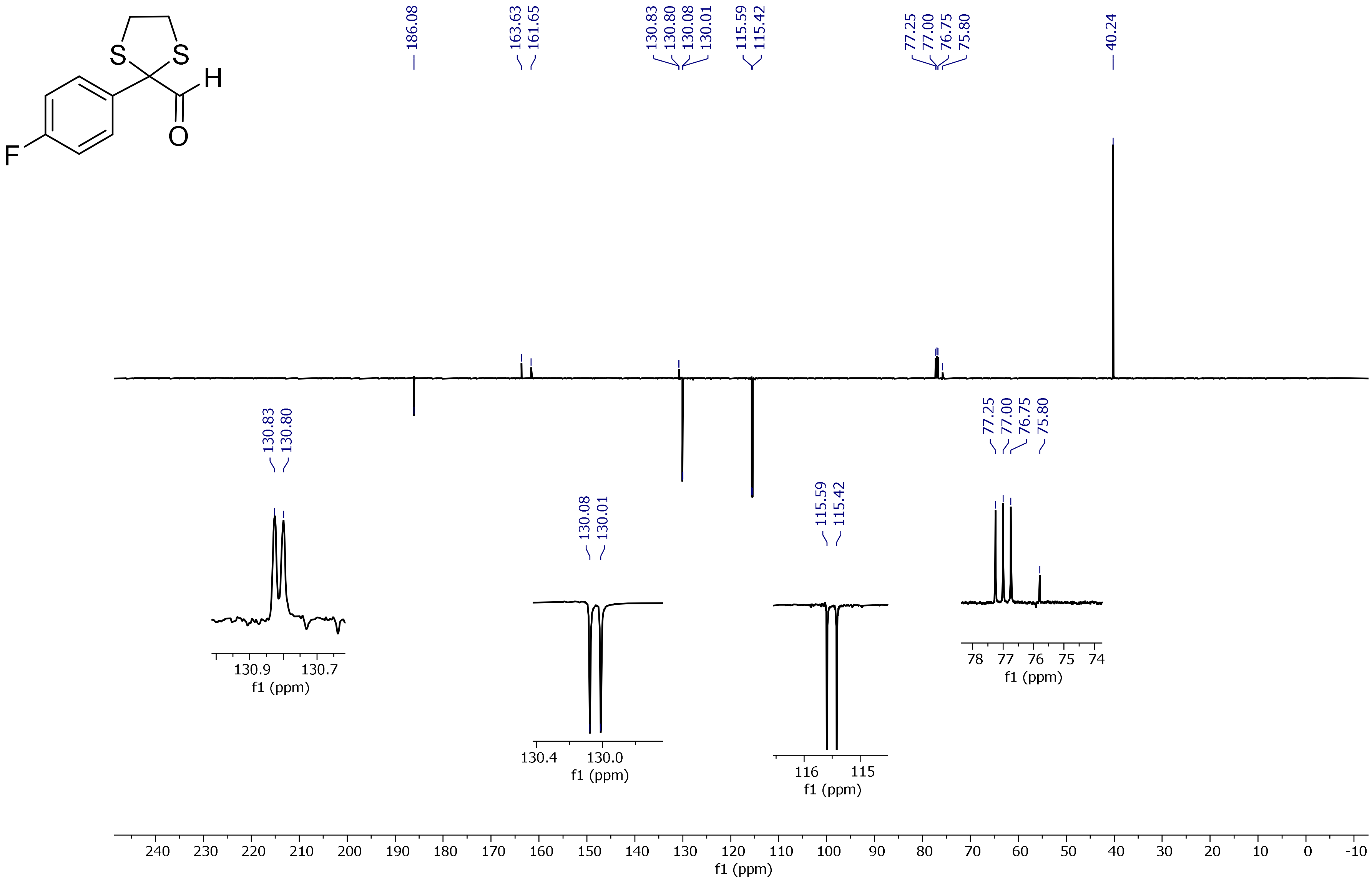

Figure S35. ^{1}H NMR ($CDCl_3$, 500 MHz) spectrum **1a**

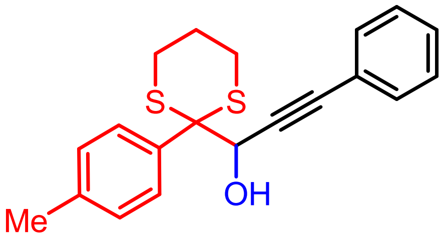

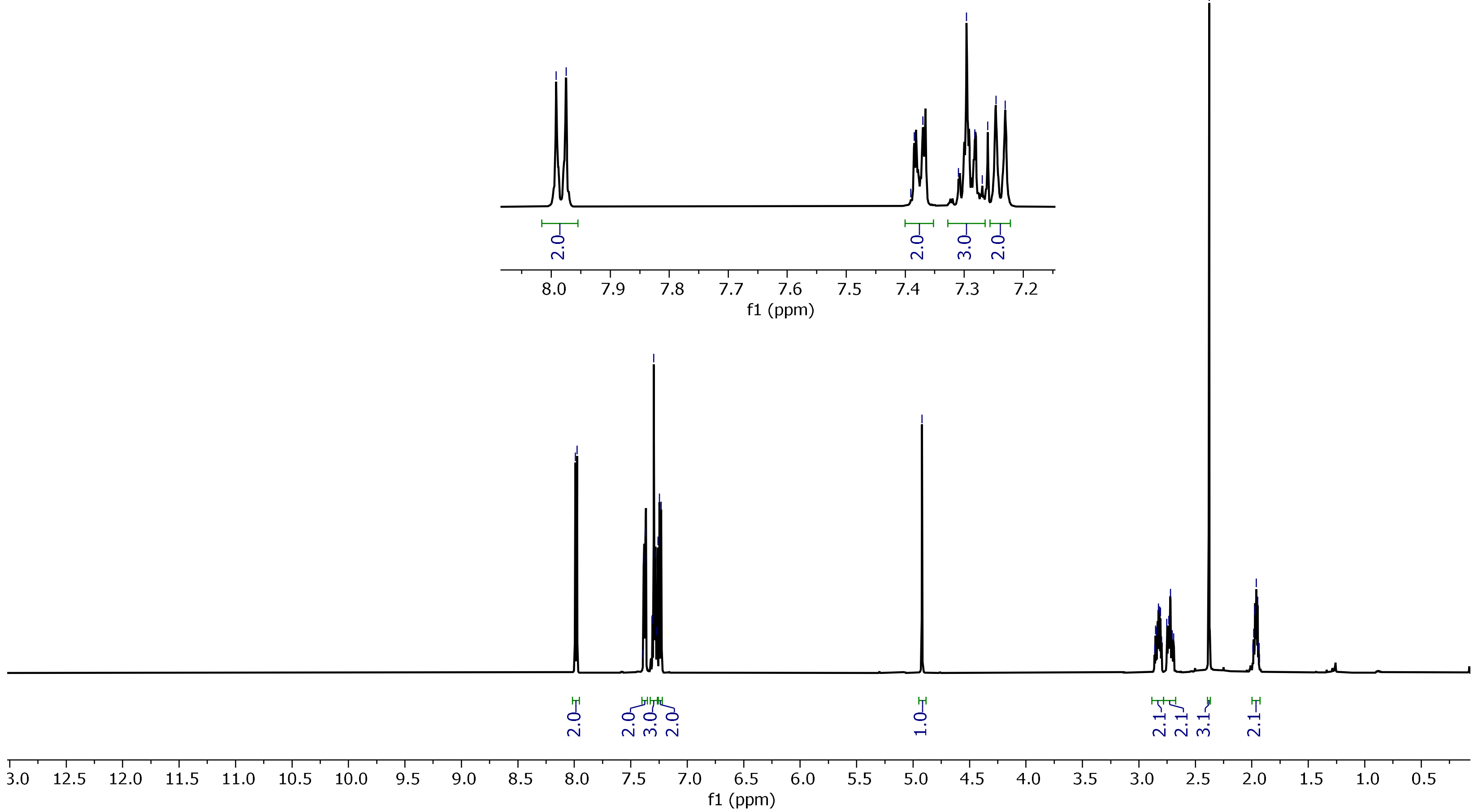

Figure S36. $^{13}C\{^1H\}$ NMR (126 MHz, $CDCl_3$, APT) spectrum **1a**

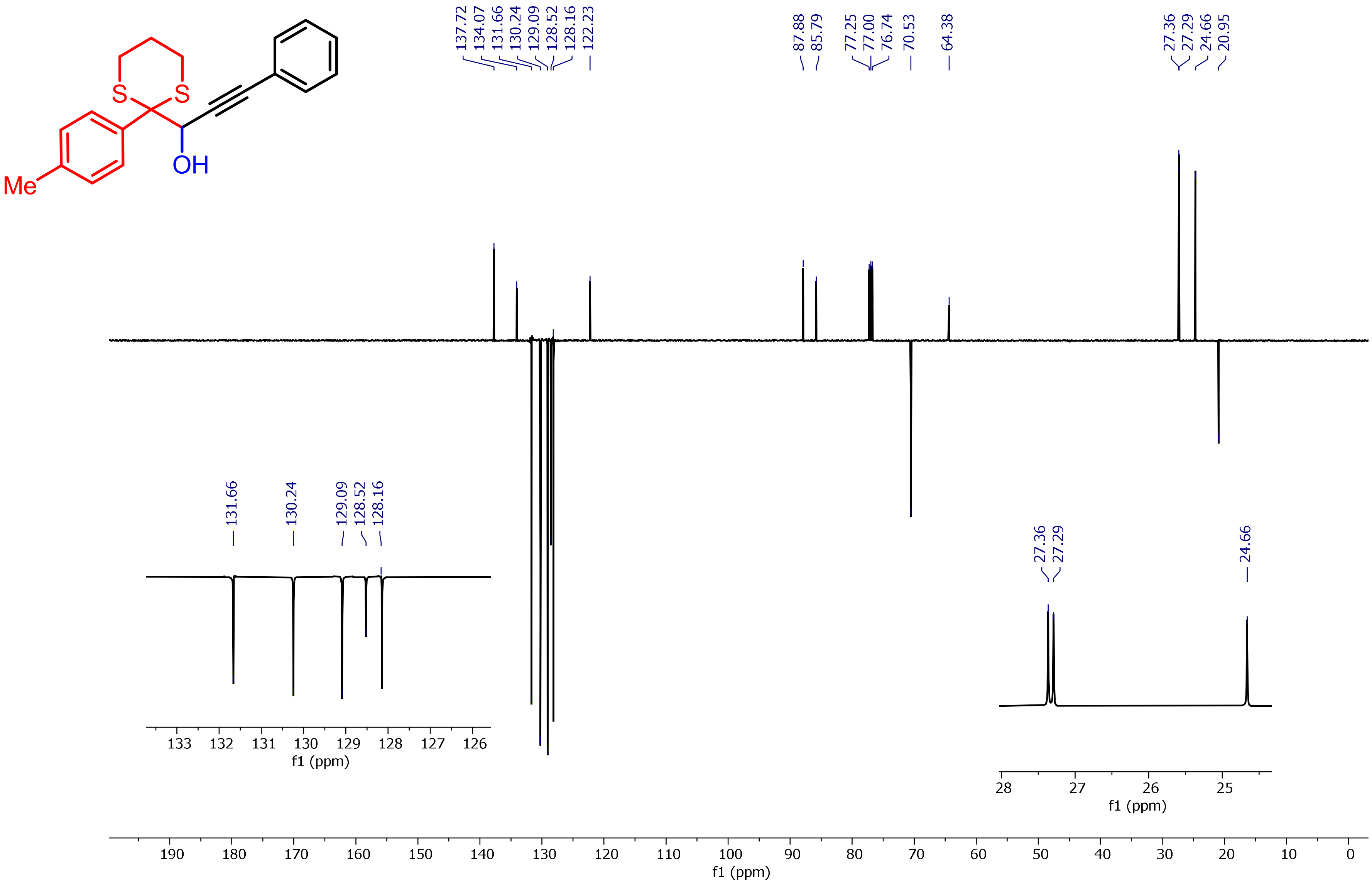

Figure S37. ^{1}H NMR ($CDCl_3$, 500 MHz) spectrum **1b**

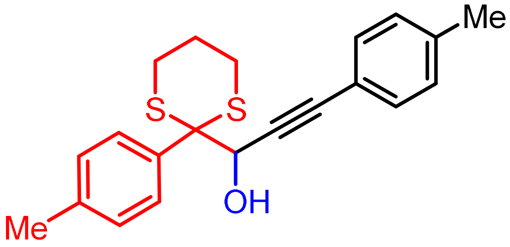

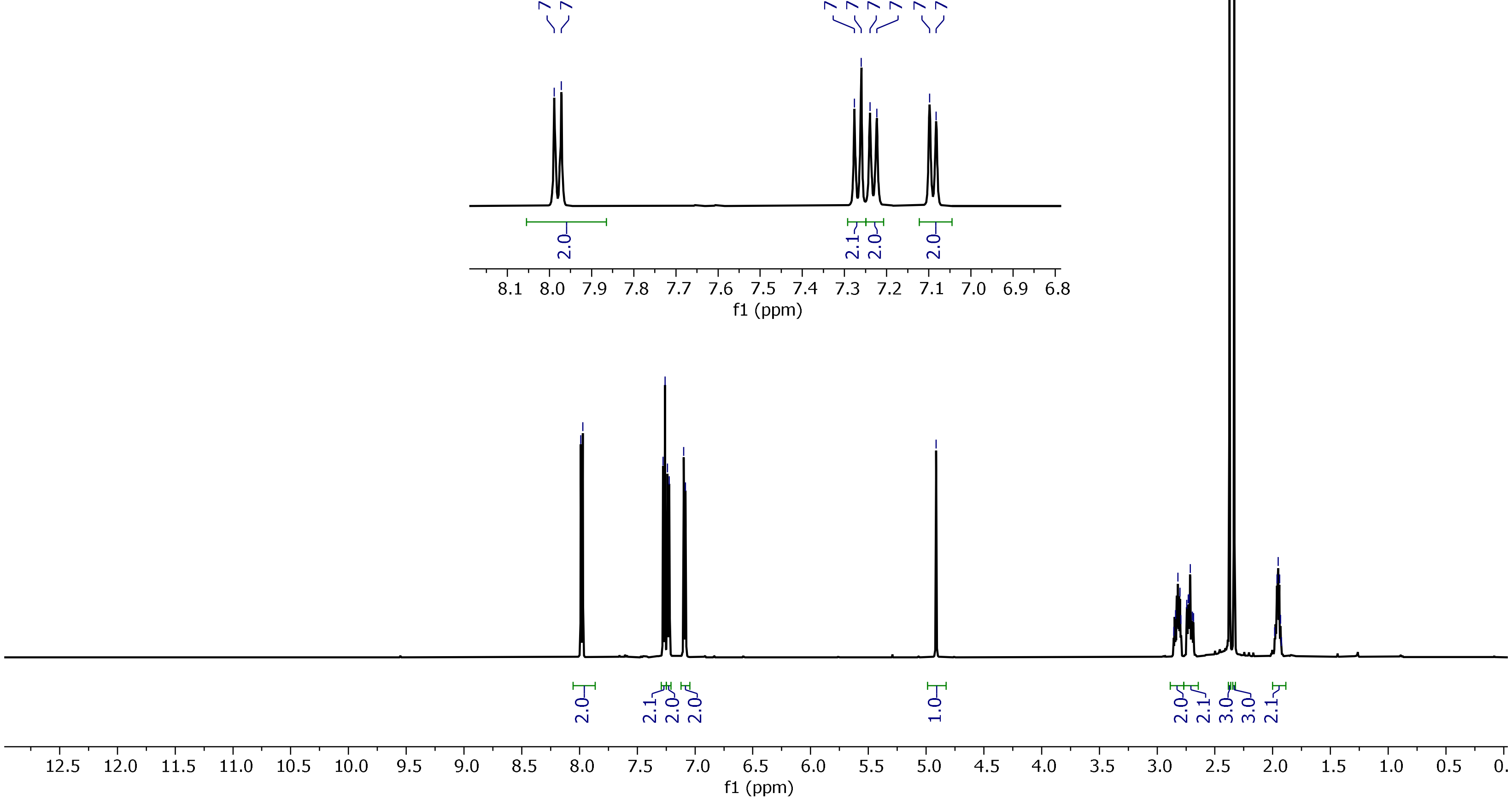

Figure S38. $^{13}C\{^1H\}$ NMR (126 MHz, $CDCl_3$, APT) spectrum **1b**

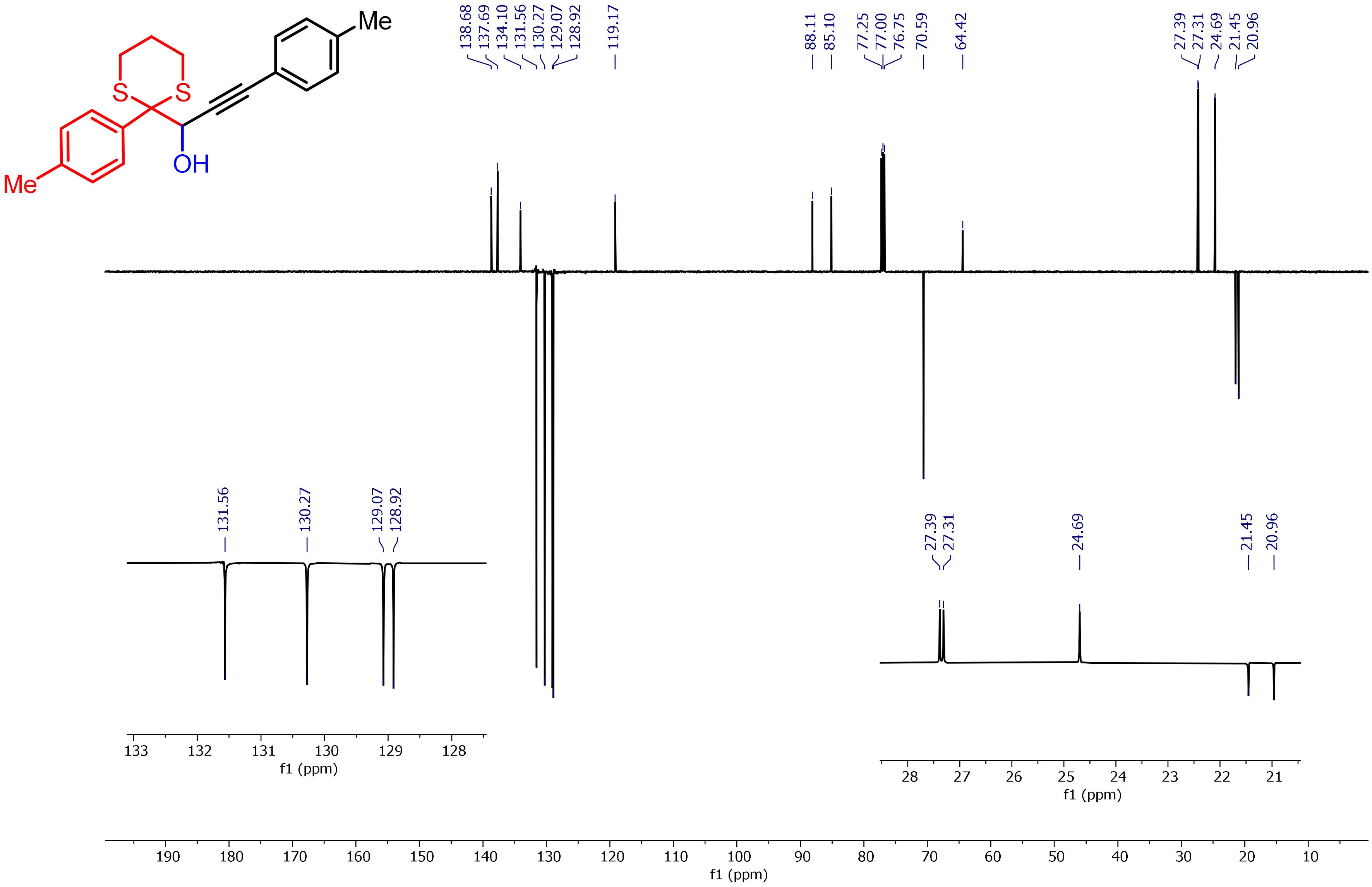

Figure S39. ^{1}H NMR ($CDCl_3$, 500 MHz) spectrum **1c**

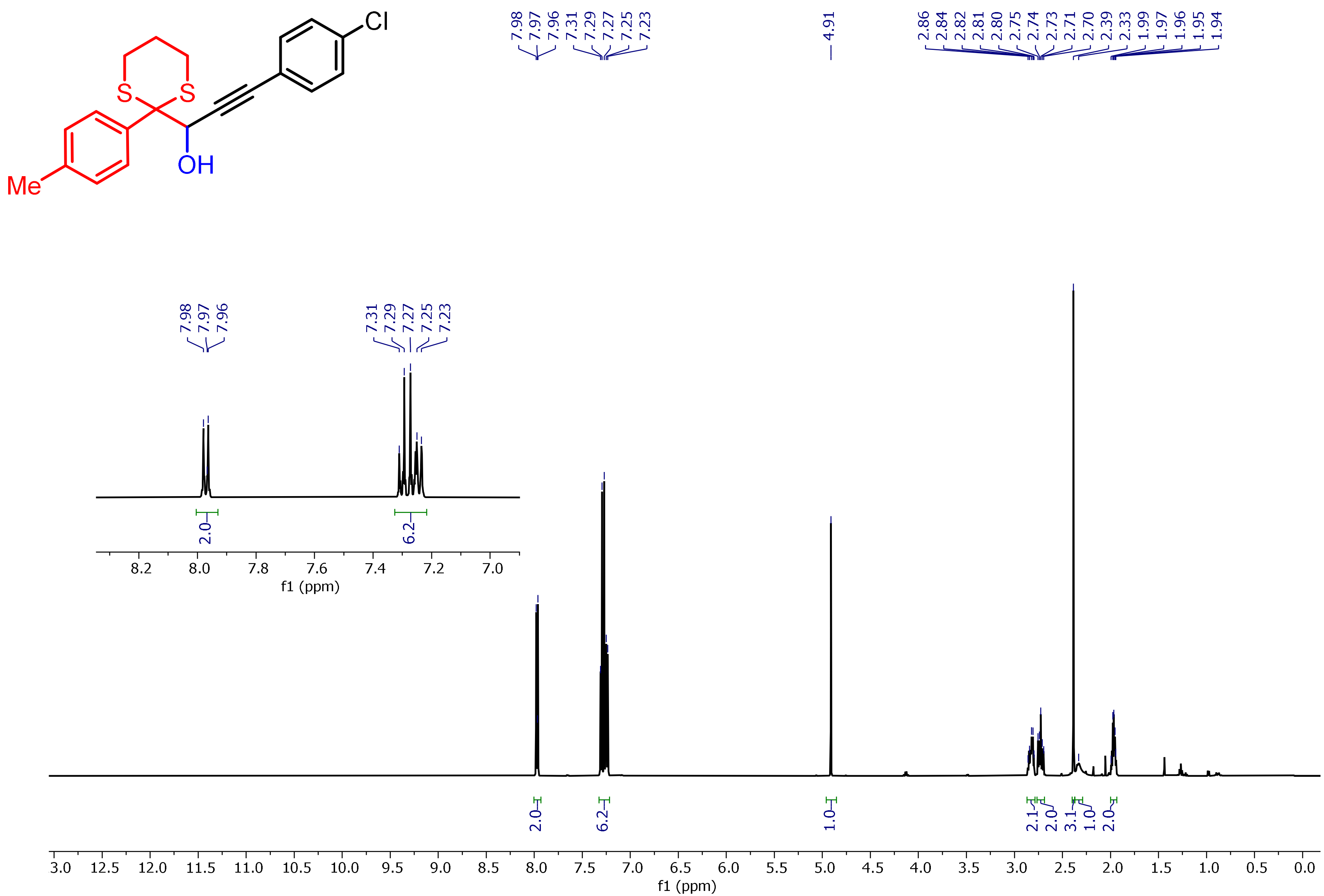

Figure S40. $^{13}C\{^1H\}$ NMR (126 MHz, $CDCl_3$, APT) spectrum **1c**

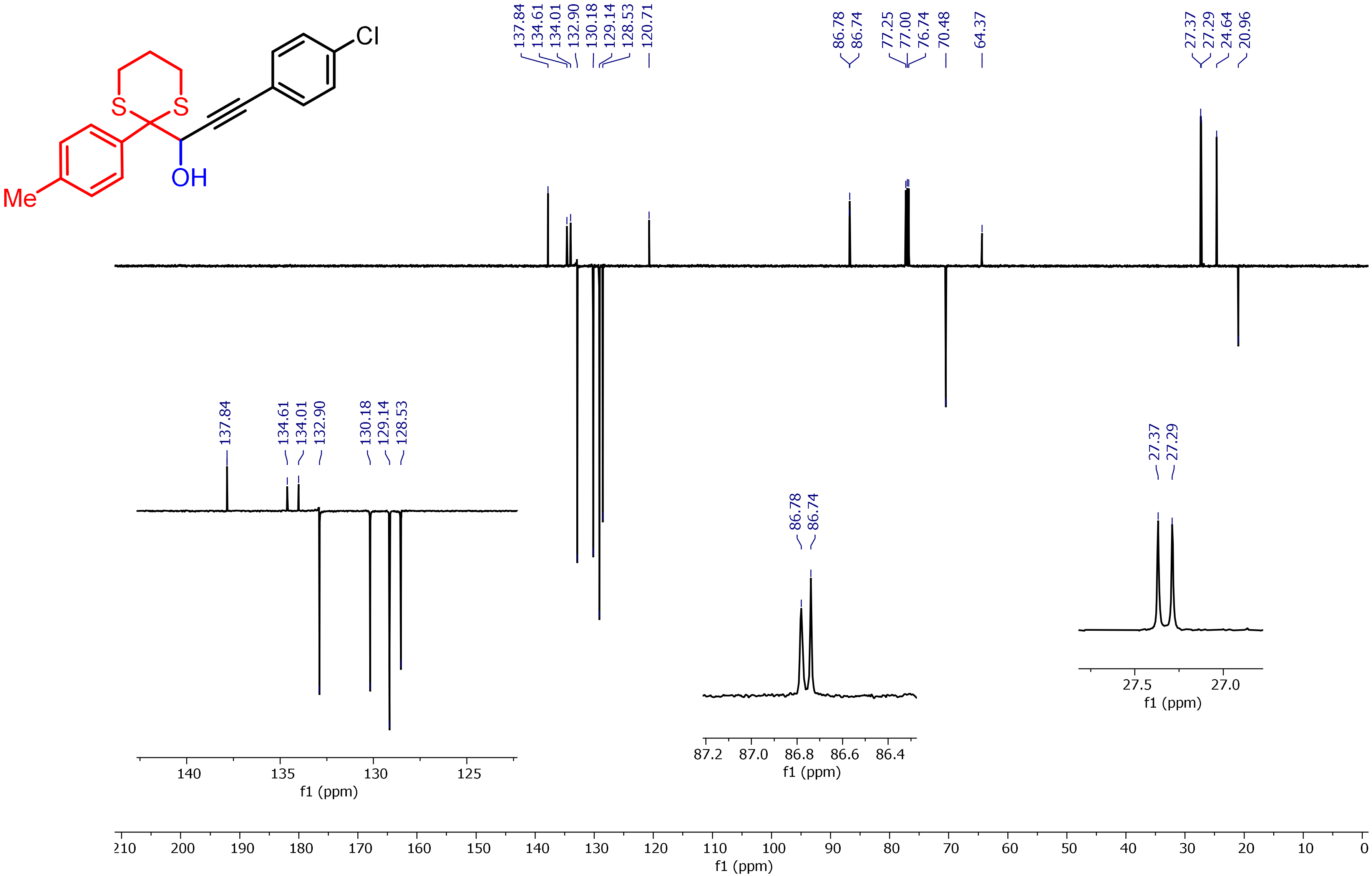

Figure S41. ^{1}H NMR ($CDCl_3$, 500 MHz) spectrum **1d**

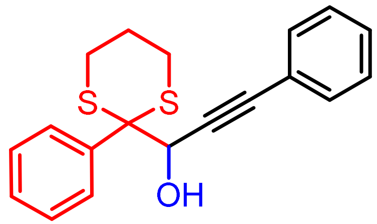

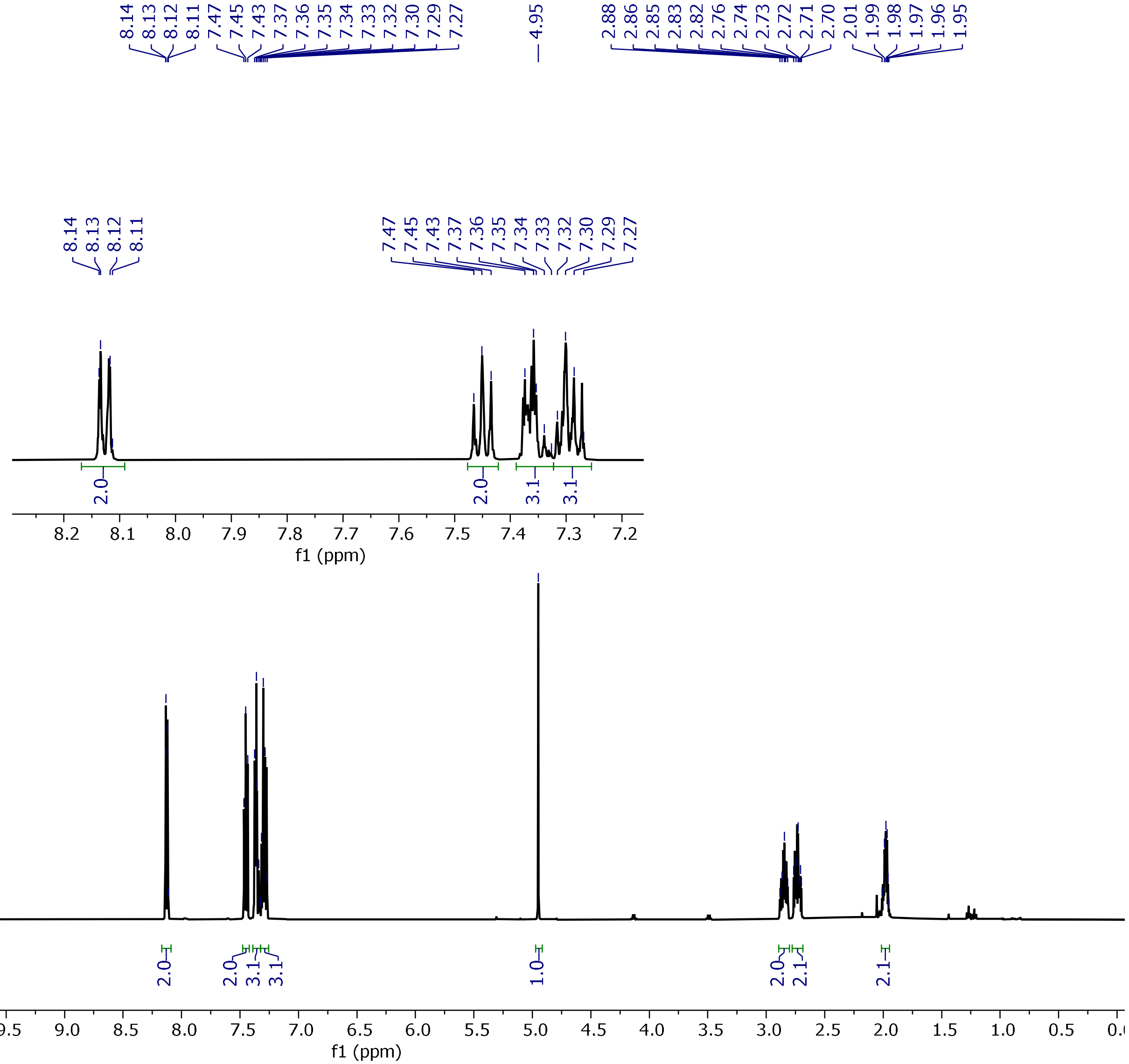

Figure S42. $^{13}C\{^{1}H\}$ NMR (126 MHz, $CDCl_3$, APT) spectrum **1d**

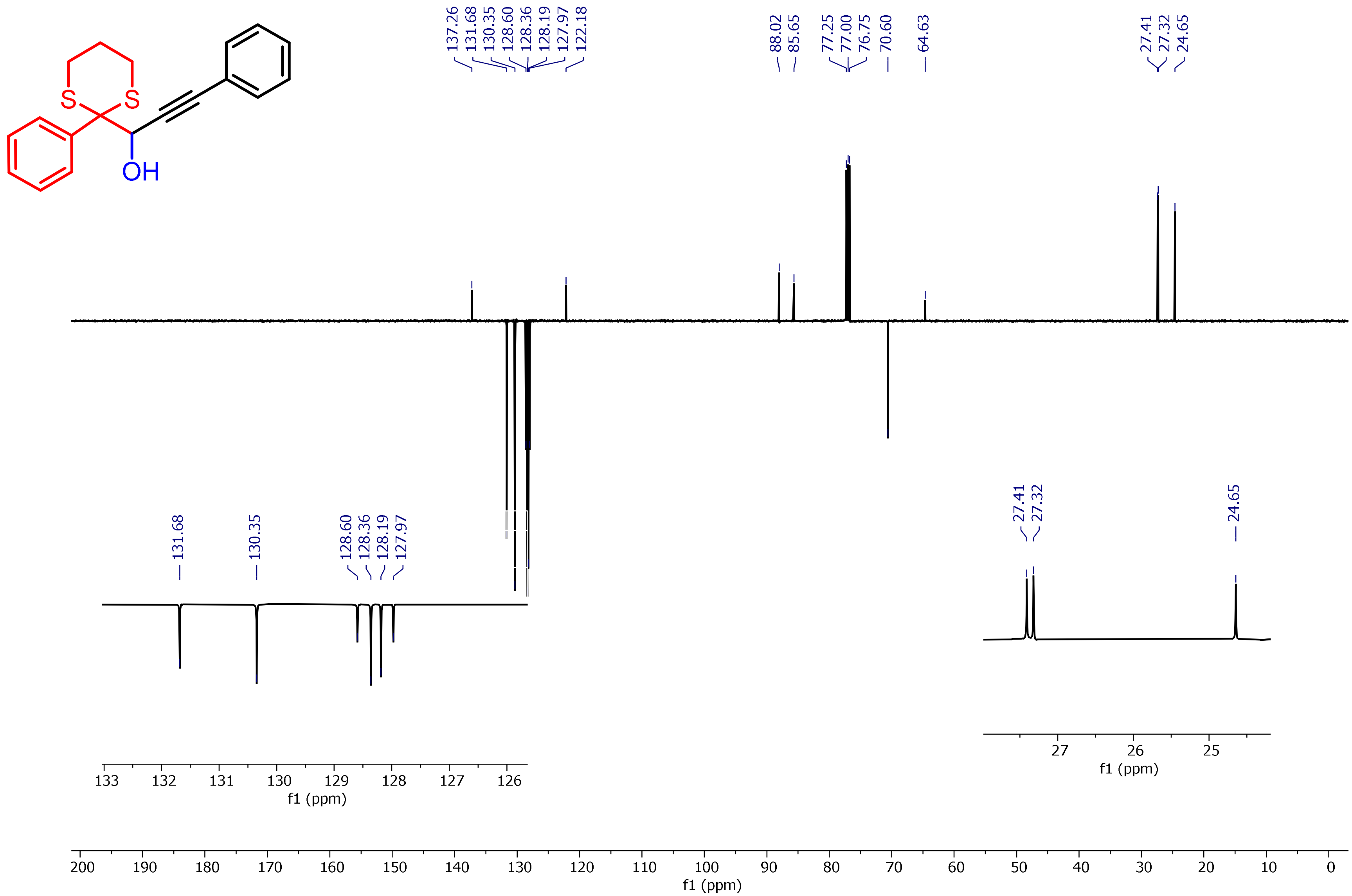

Figure S43. ^{1}H NMR ($CDCl_3$, 500 MHz) spectrum **1e**

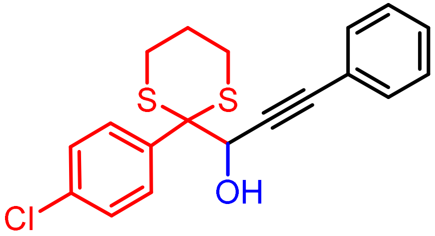

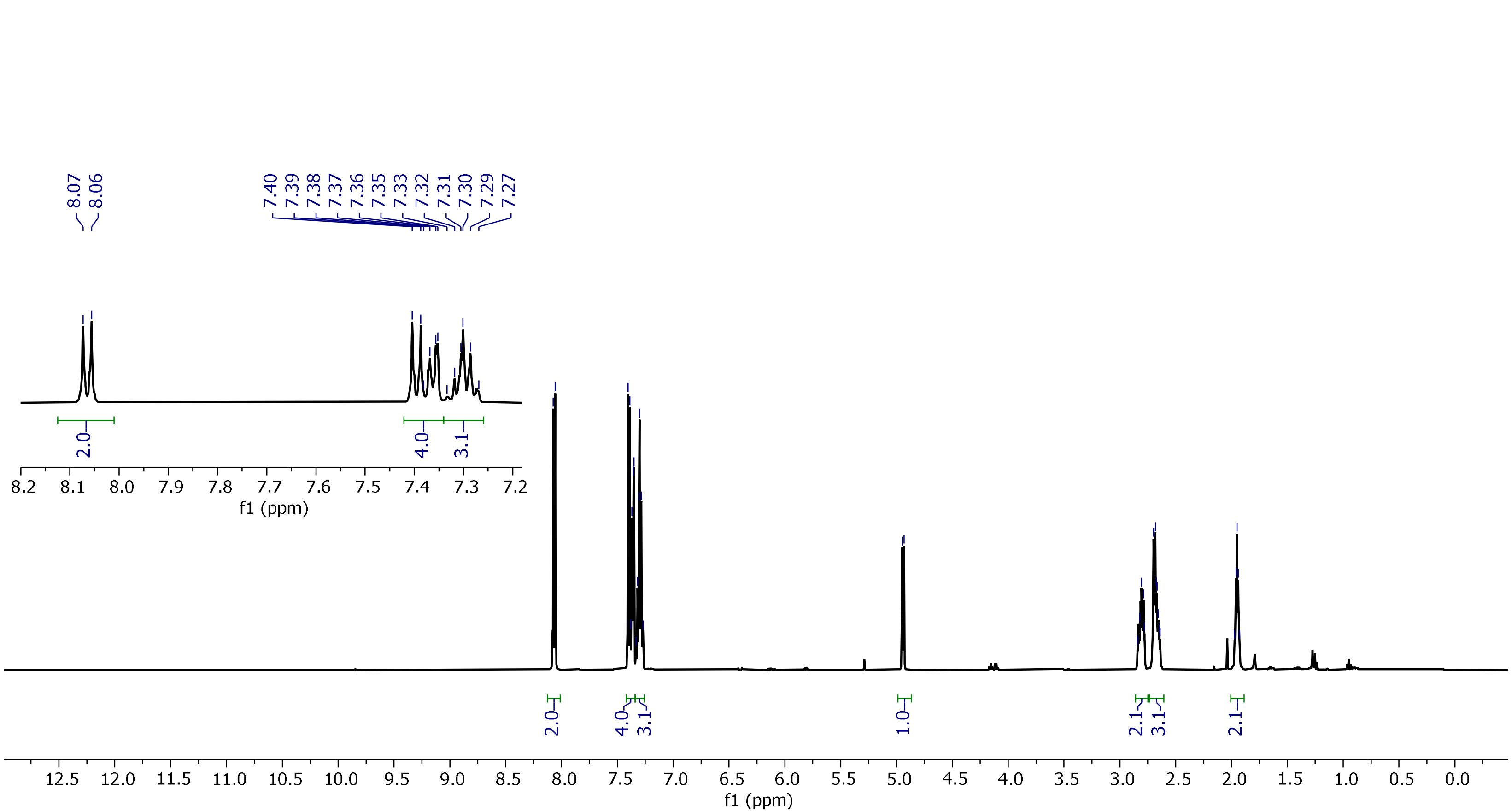

Figure S44. $^{13}C\{^1H\}$ NMR (126 MHz, $CDCl_3$, APT) spectrum **1e**

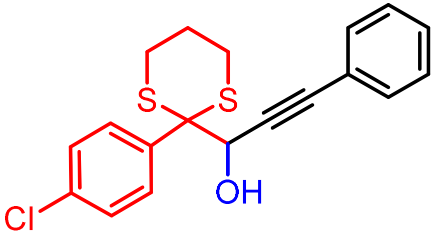

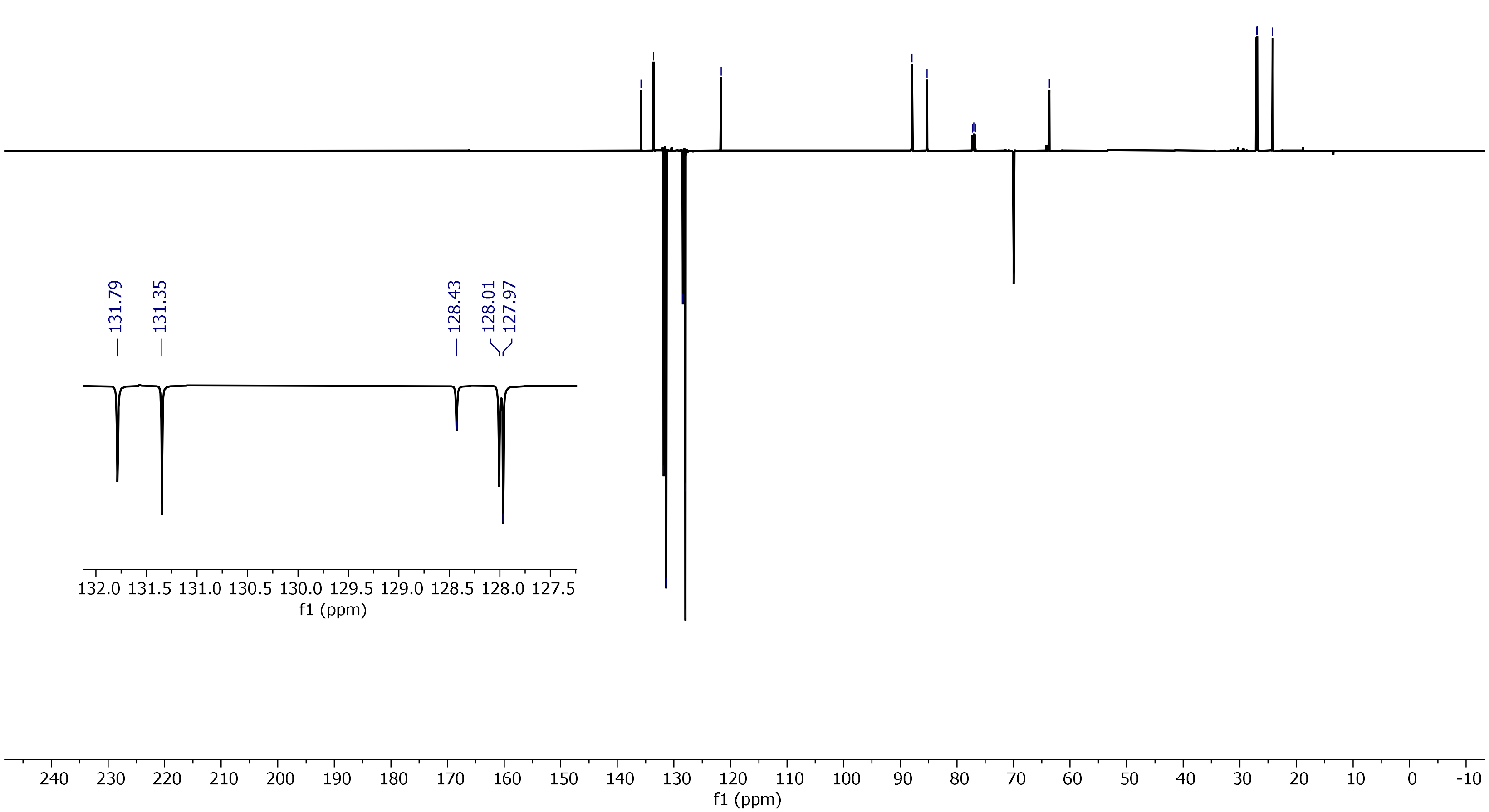

Figure S45. ^{1}H NMR ($CDCl_3$, 500 MHz) spectrum **1f**

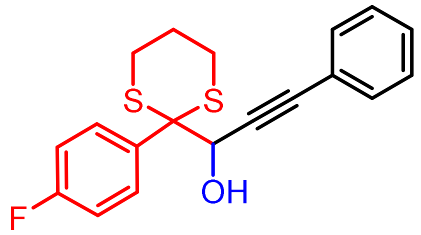

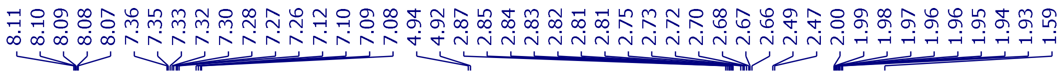

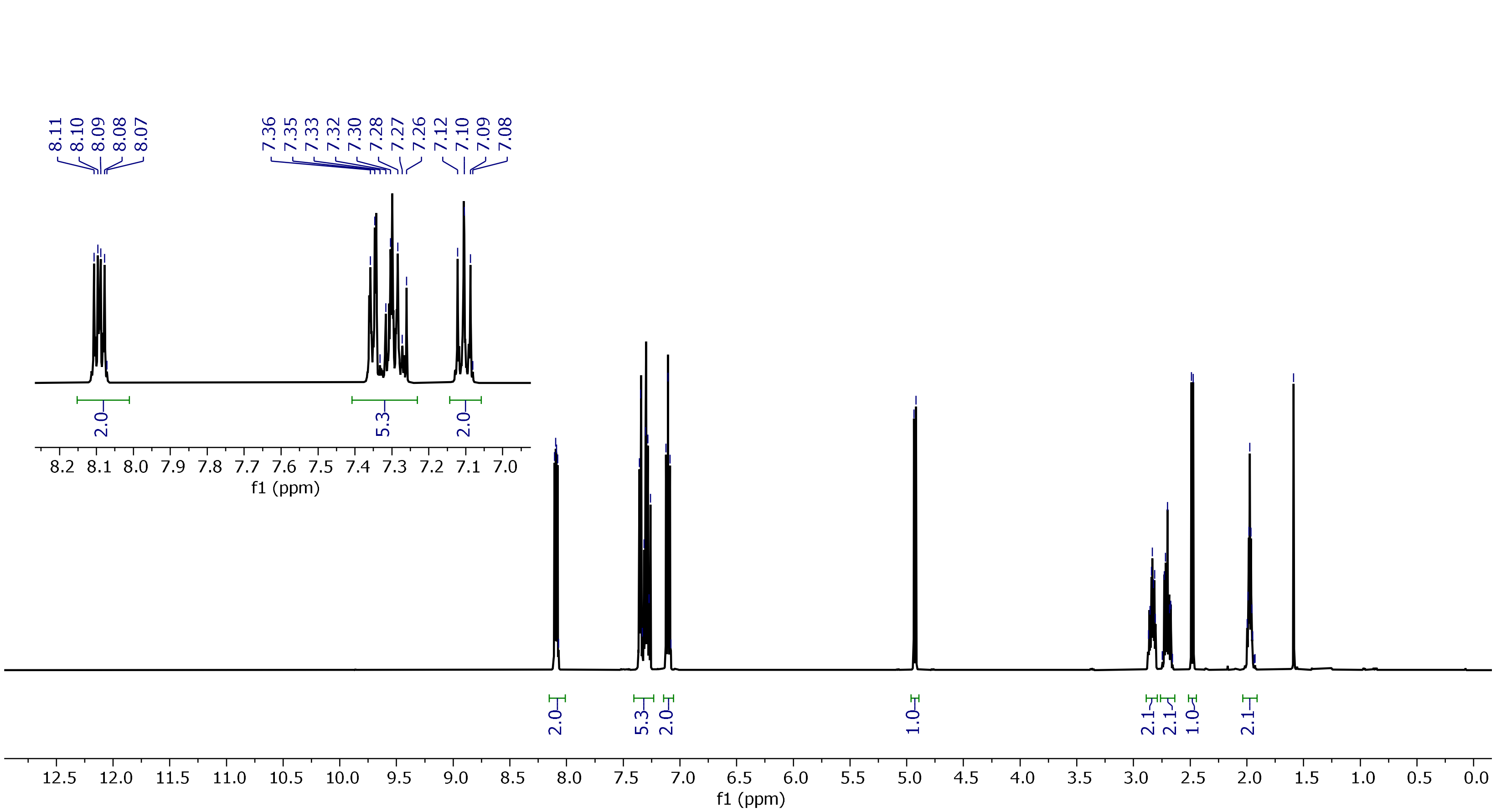

Figure S46. $^{13}C\{^1H\}$ NMR (126 MHz, $CDCl_3$, APT) spectrum **1f**

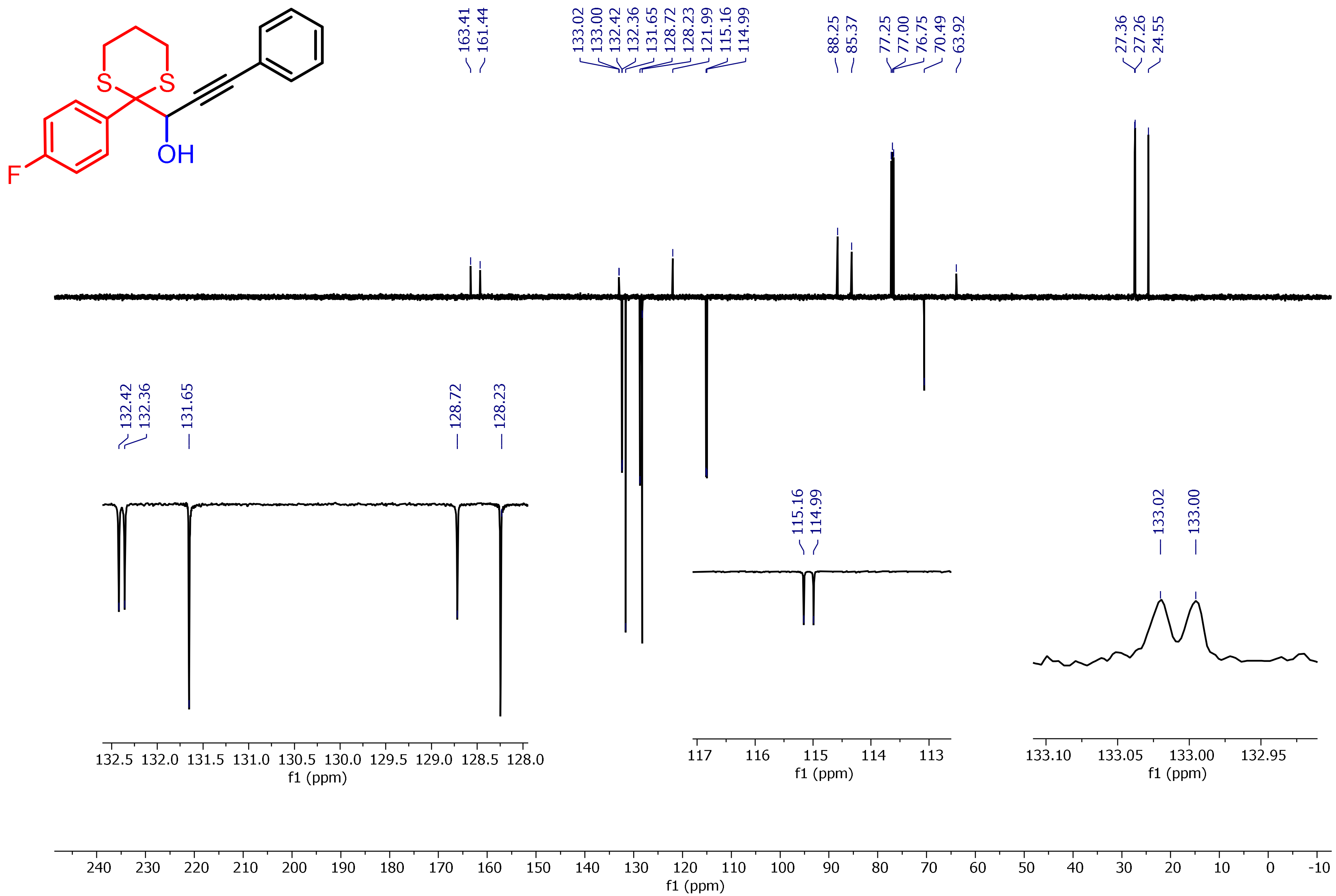

Figure S47. ^{1}H NMR ($CDCl_3$, 500 MHz) spectrum **1g**

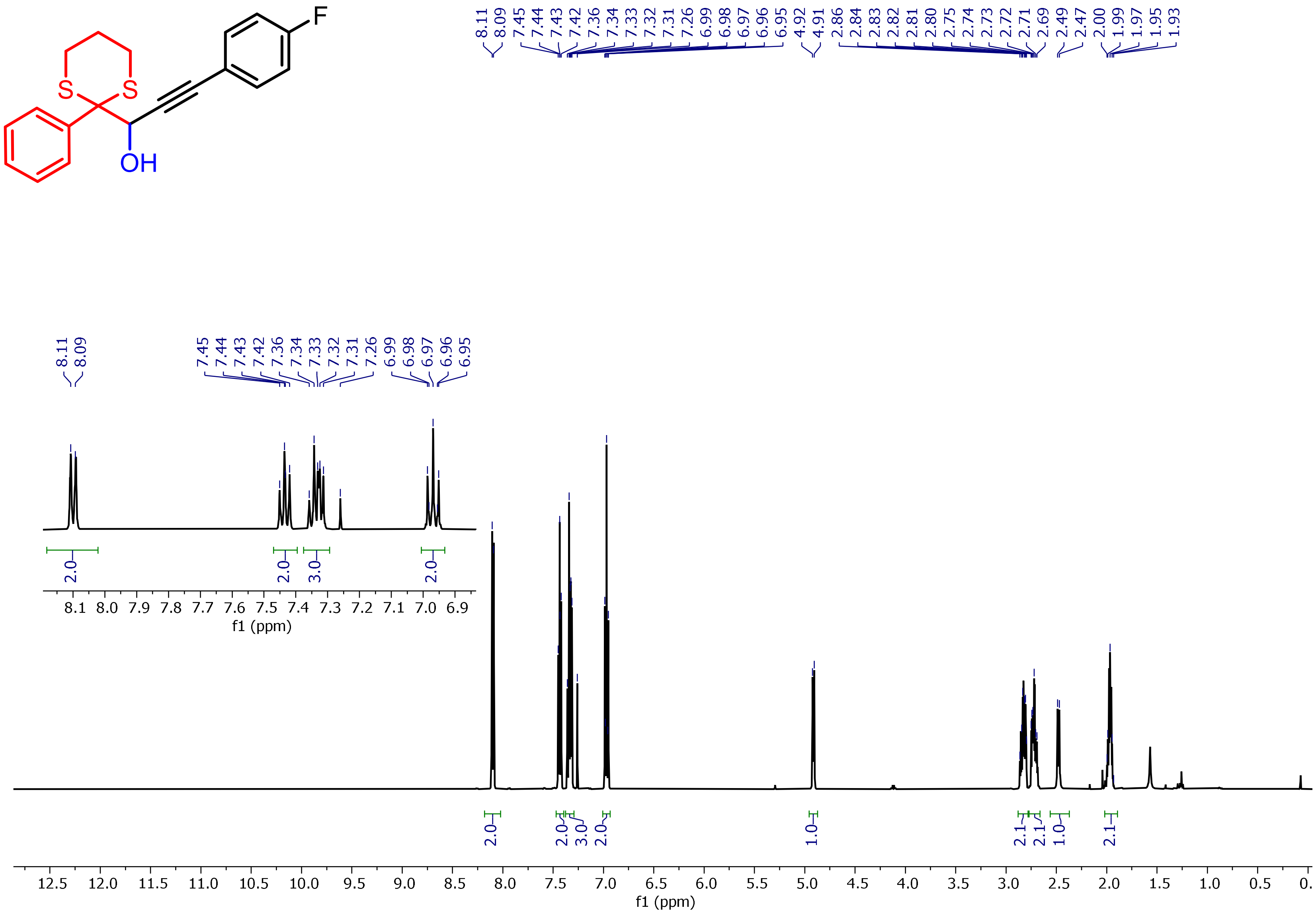

Figure S48. $^{13}C\{^{1}H\}$ NMR (126 MHz, $CDCl_3$, APT) spectrum **1g**

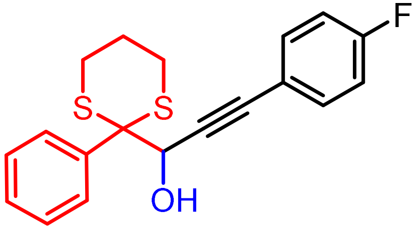

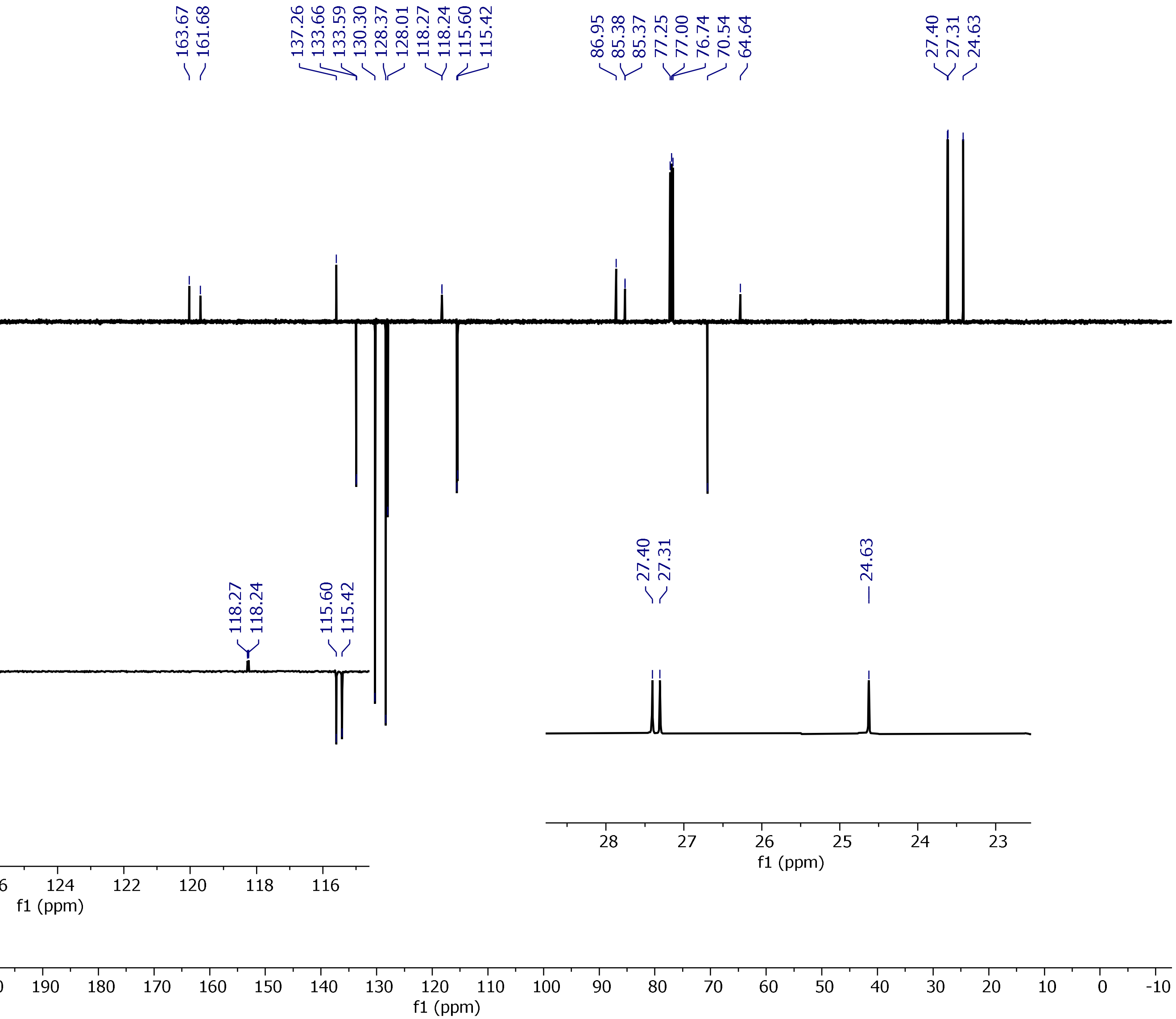

Figure S49. ^{1}H NMR ($CDCl_3$, 500 MHz) spectrum **1h**

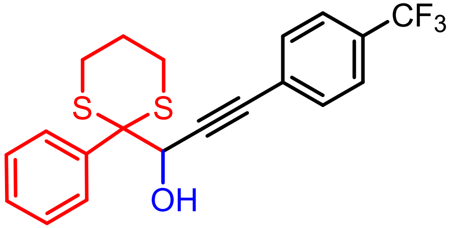

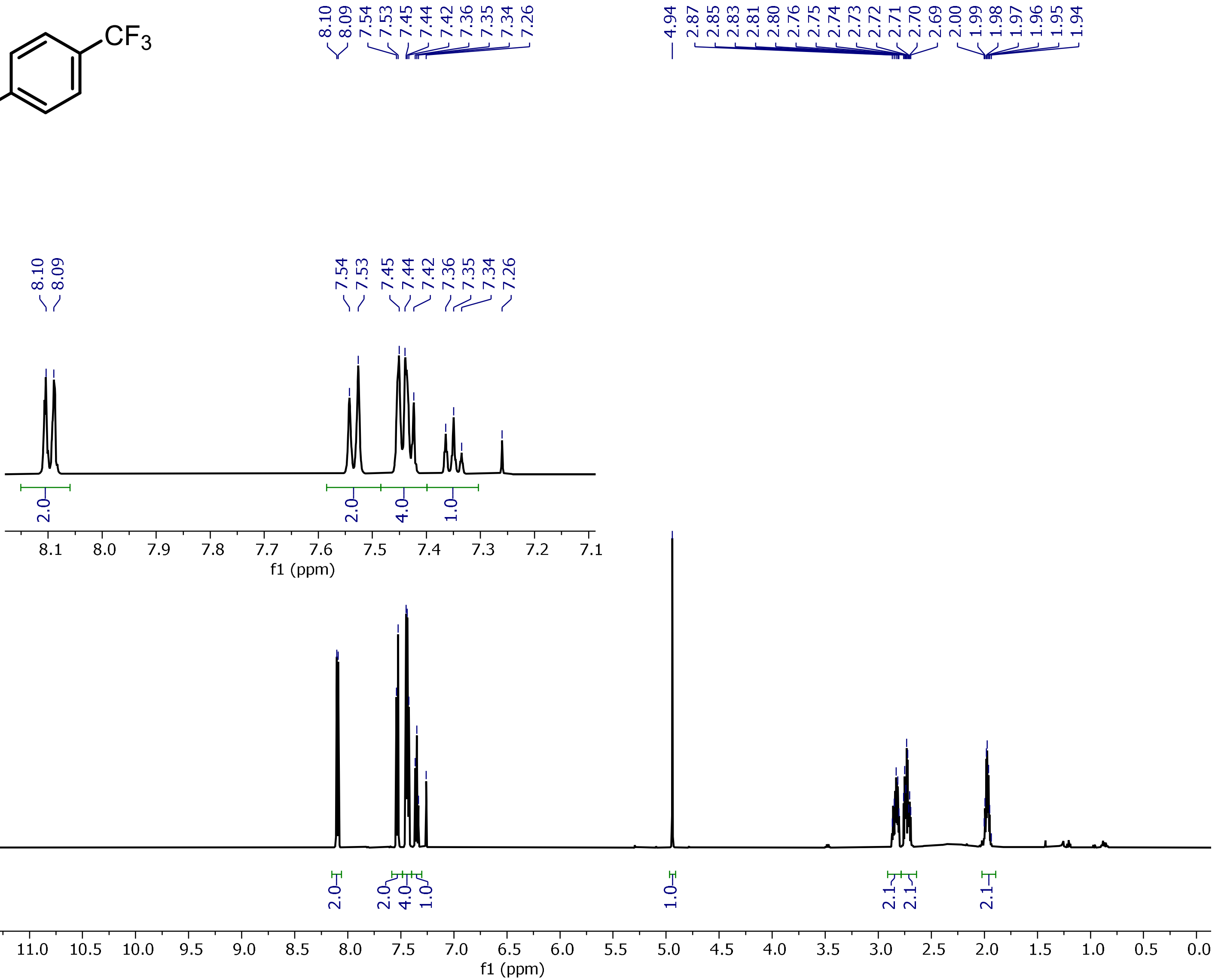

Figure S50. $^{13}C\{^1H\}$ NMR (126 MHz, $CDCl_3$, APT) spectrum **1h**

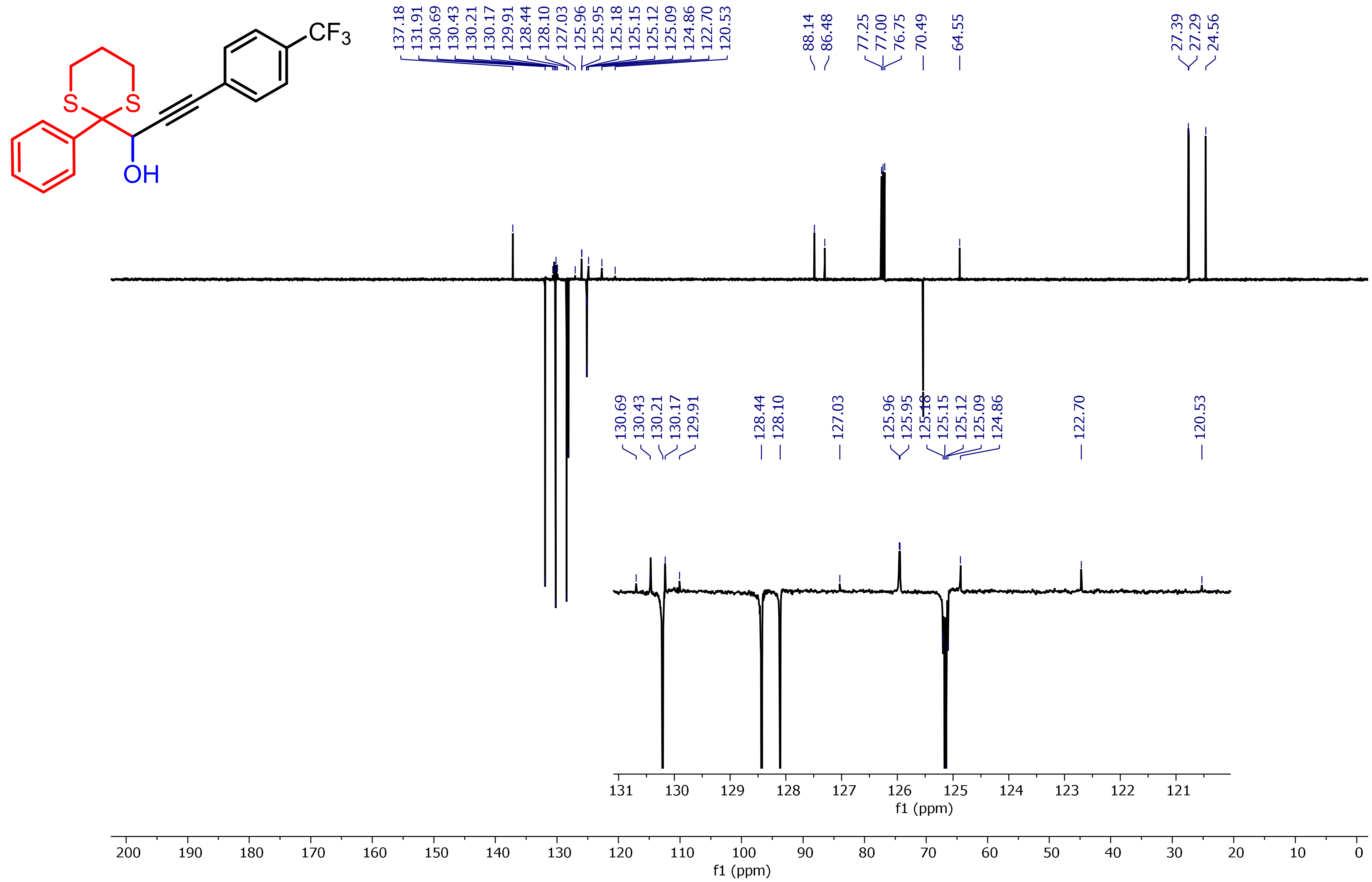

Figure S51. ^{1}H NMR ($CDCl_3$, 500 MHz) spectrum **1i**

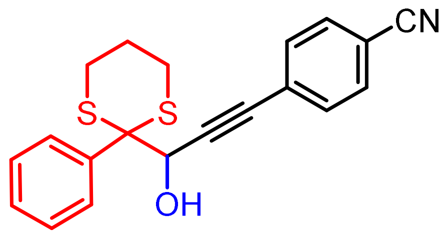

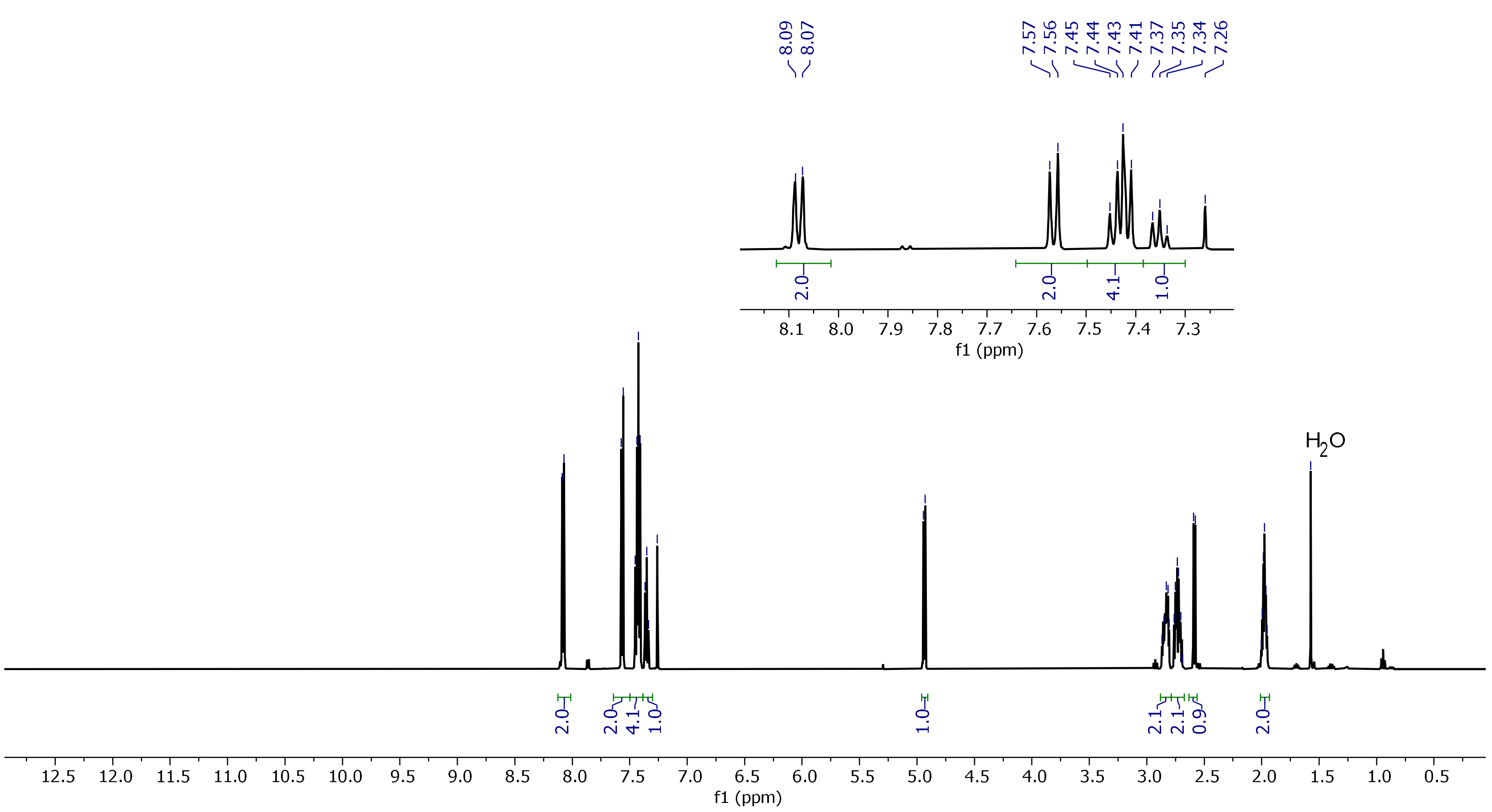

Figure S52. $^{13}C\{^{1}H\}$ NMR (126 MHz, $CDCl_3$, APT) spectrum **1i**

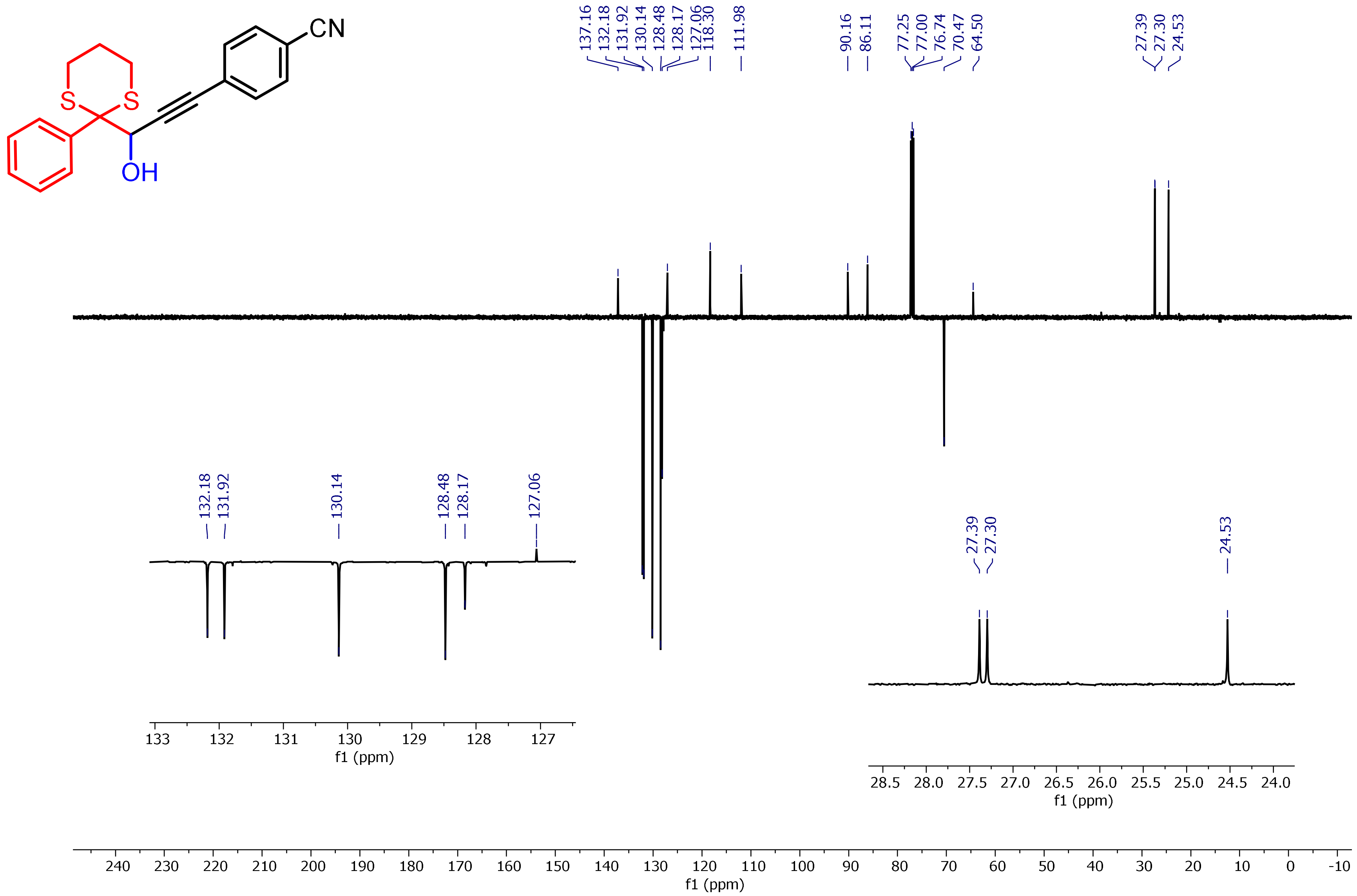

Figure S53. ^{1}H NMR ($CDCl_3$, 500 MHz) spectrum **1j**

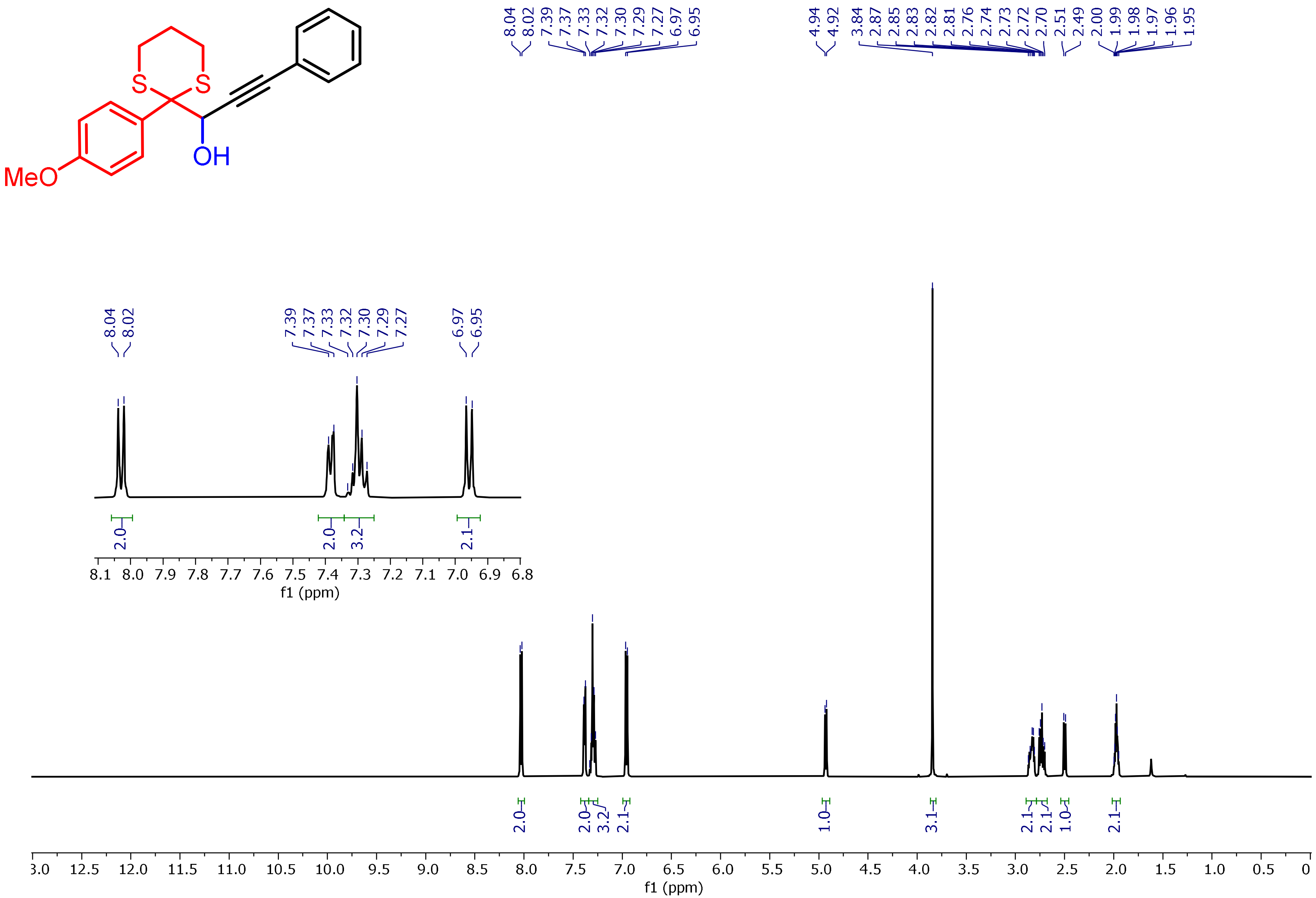

Figure S54. $^{13}C\{^1H\}$NMR (126 MHz, $CDCl_3$, APT) spectrum **1j**

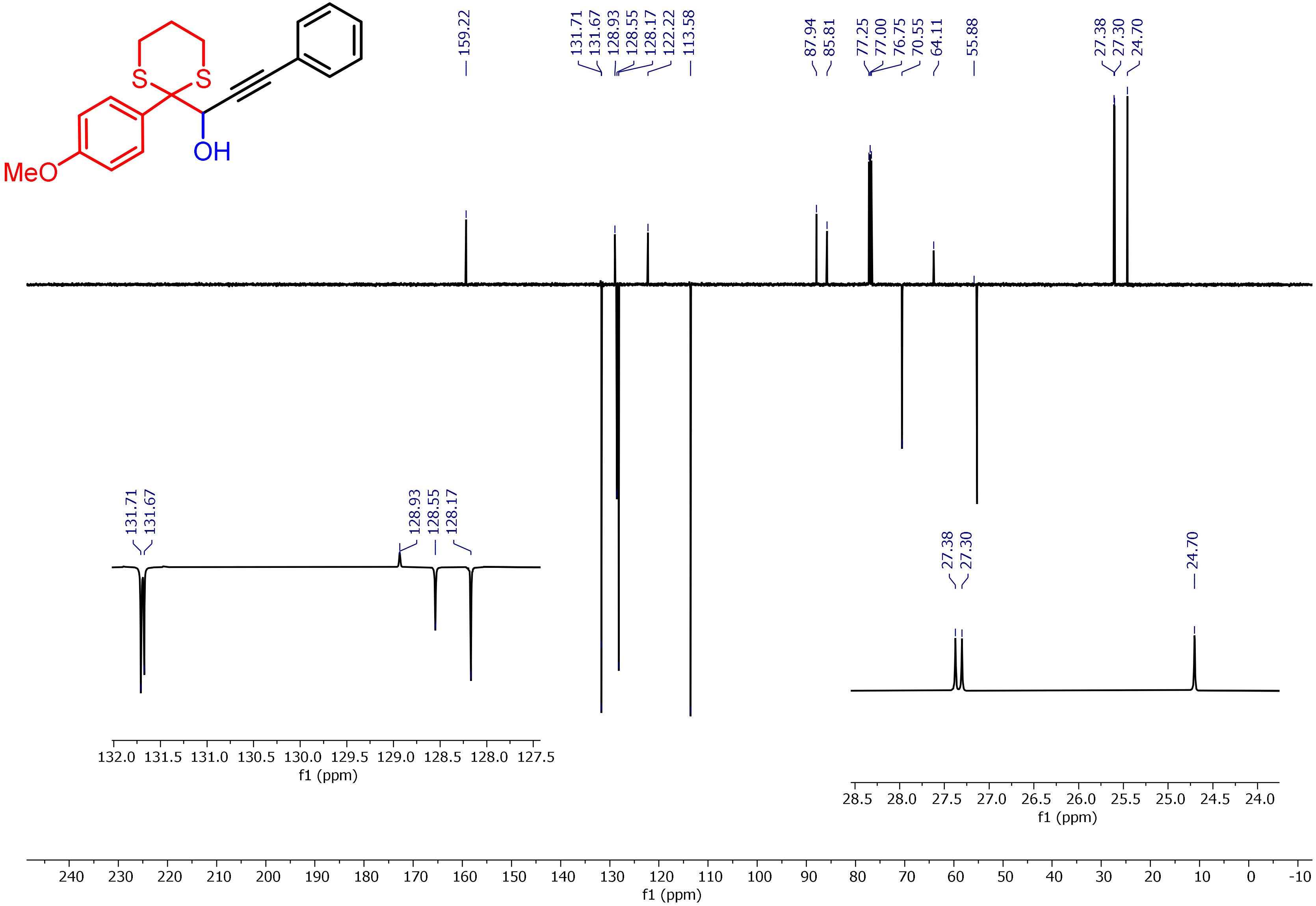

Figure S55. ^{1}H NMR ($CDCl_3$, 500 MHz) spectrum **1k**

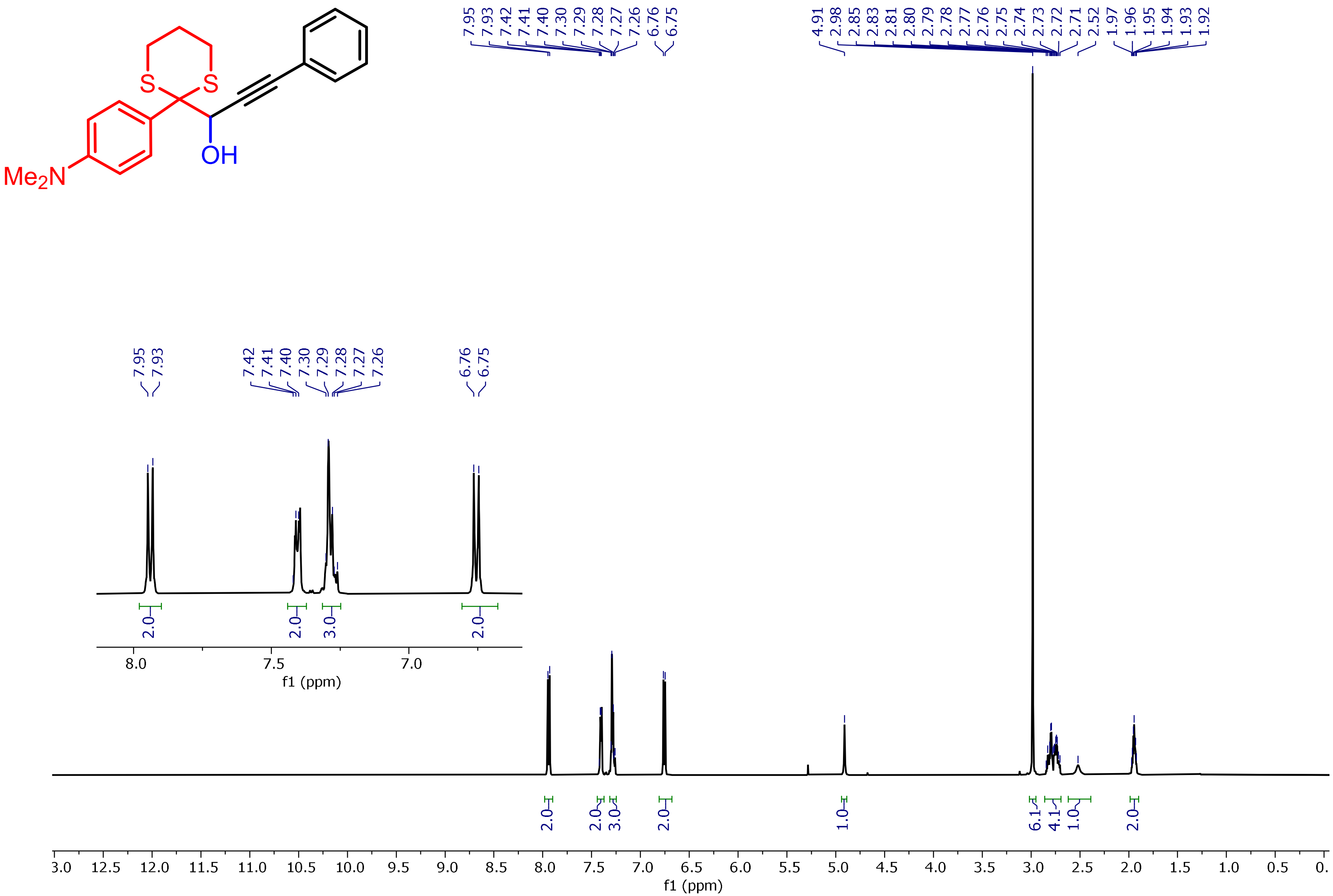

Figure S56. ${}^{13}C\{{}^{1}H\}$ NMR (126 MHz, $CDCl_3$, APT) spectrum **1k**

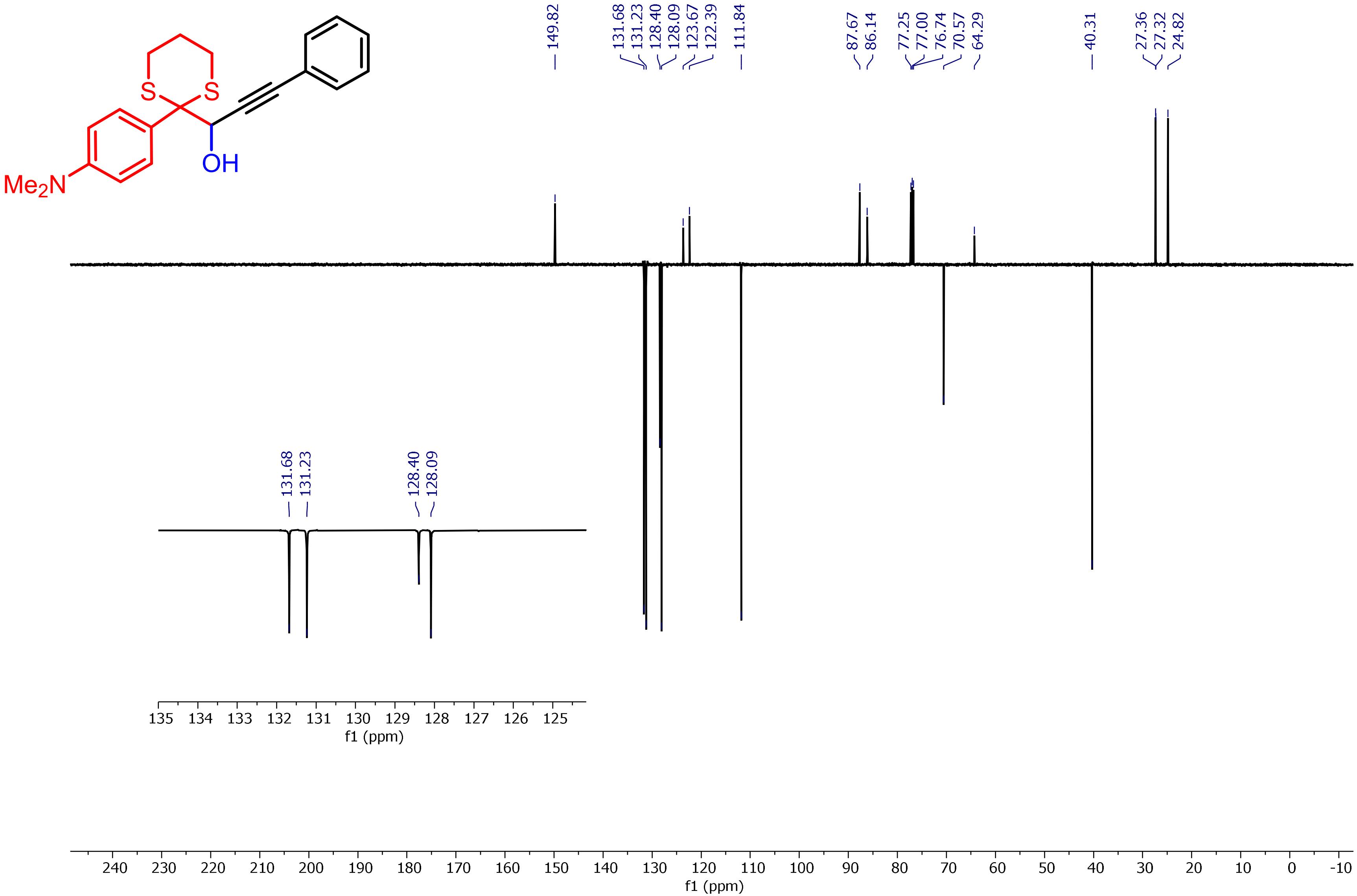

Figure S57. ^{1}H NMR ($CDCl_3$, 500 MHz) spectrum **1l**

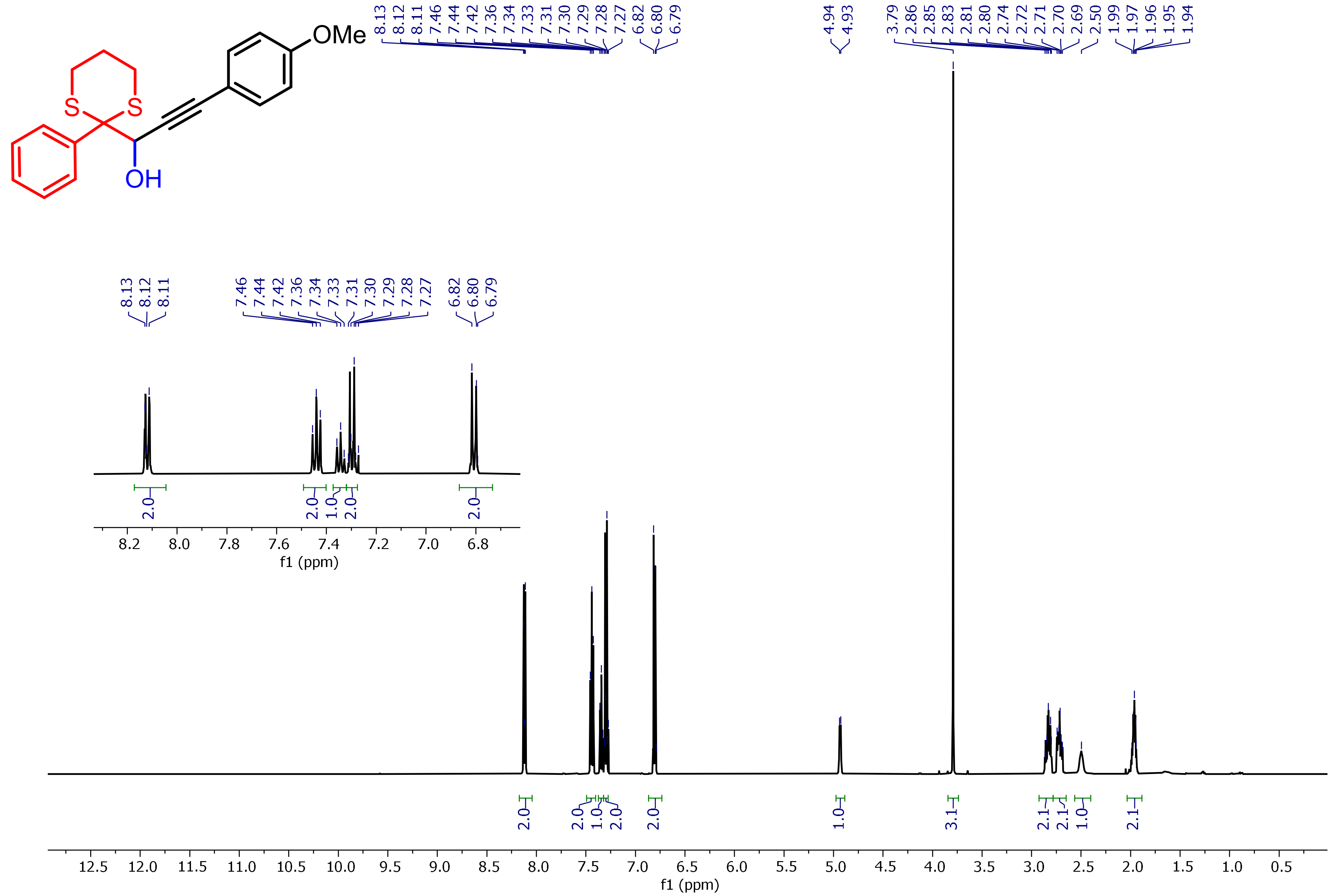

Figure S58. $^{13}C\{^{1}H\}$ NMR (126 MHz, $CDCl_3$, APT) spectrum **1l**

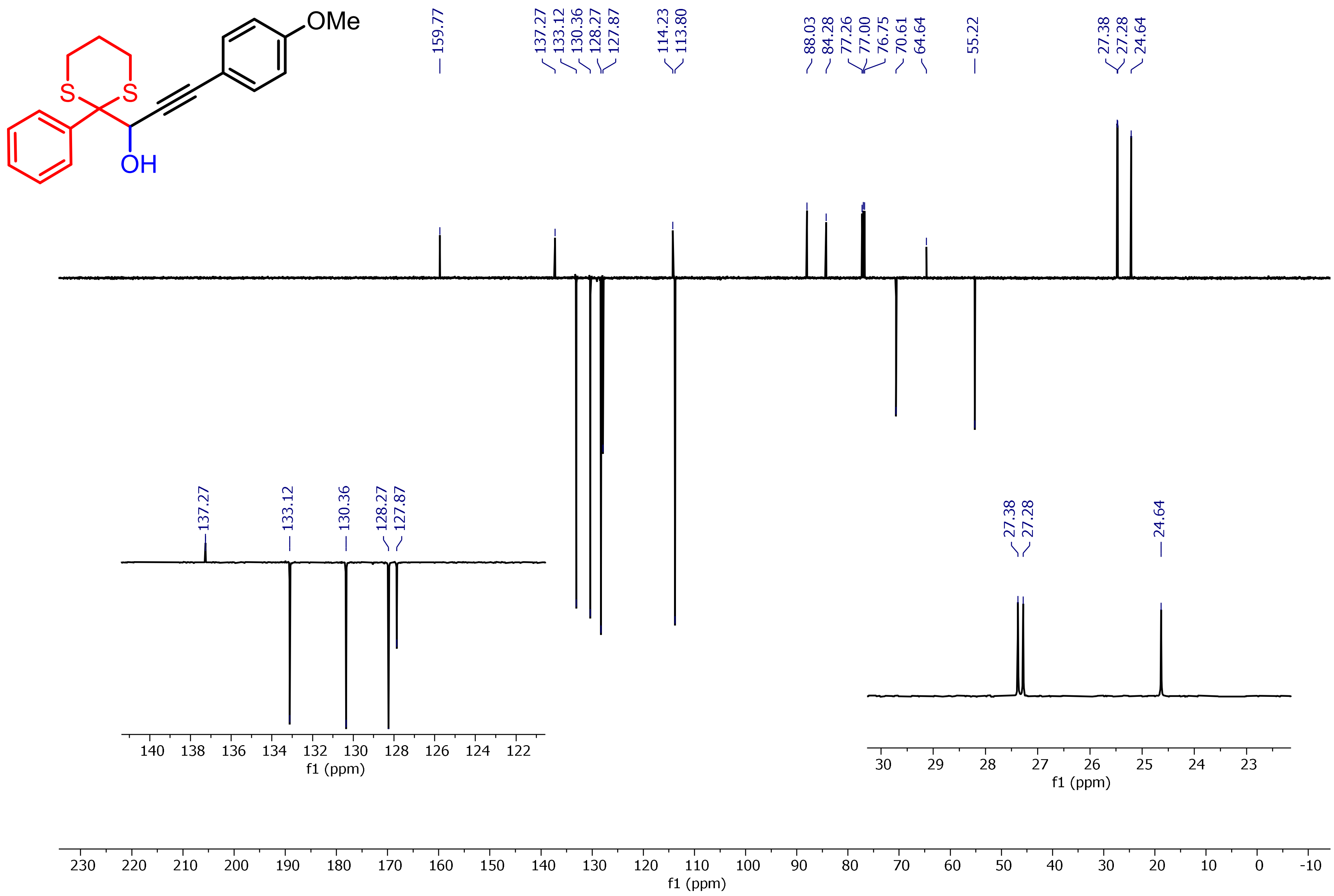

Figure S59. ^{1}H NMR ($CDCl_3$, 500 MHz) spectrum **1m**

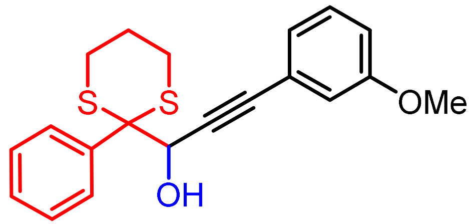

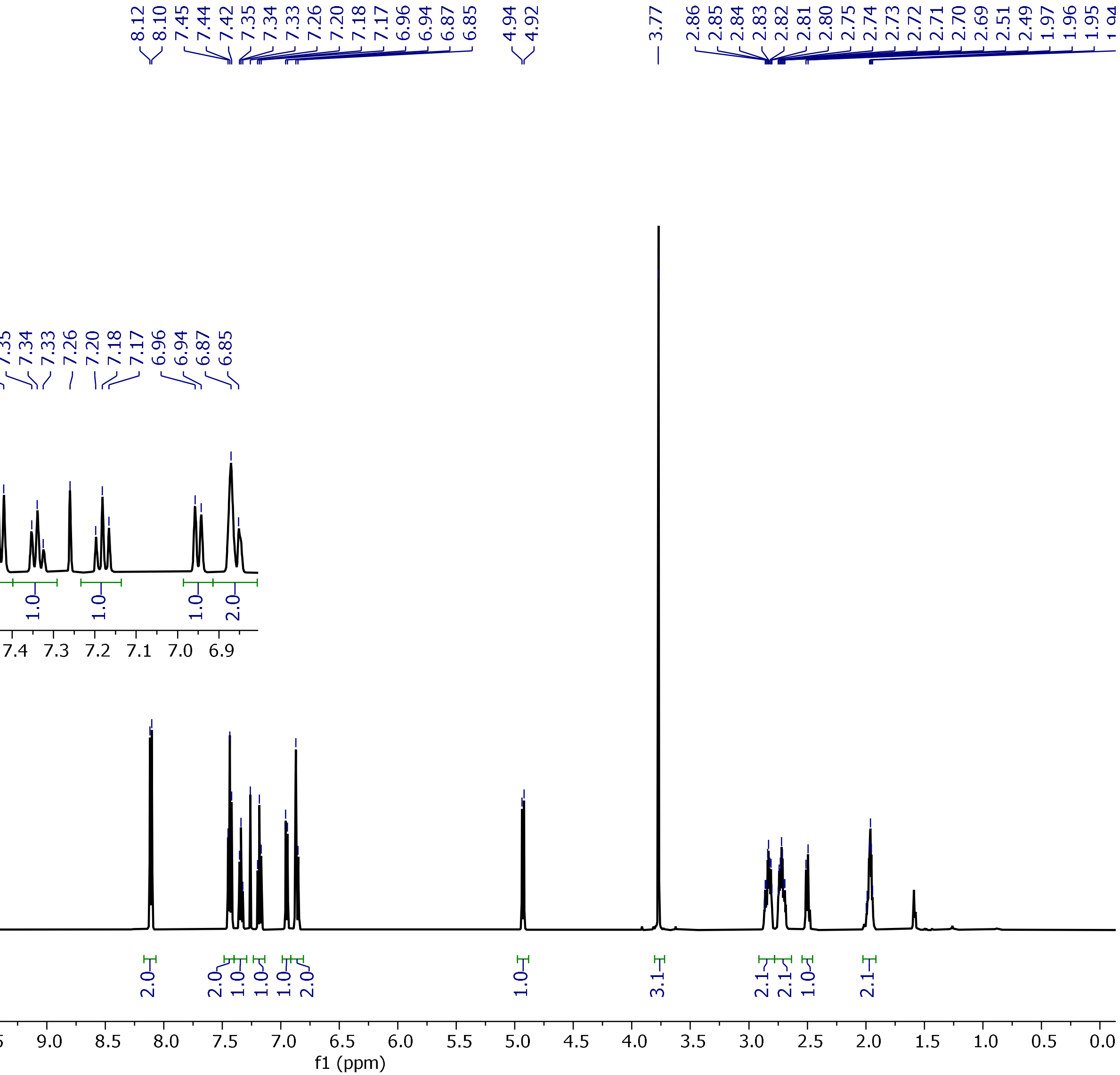

Figure S60. $^{13}C\{^{1}H\}$ NMR (126 MHz, $CDCl_3$, APT) spectrum **1m**

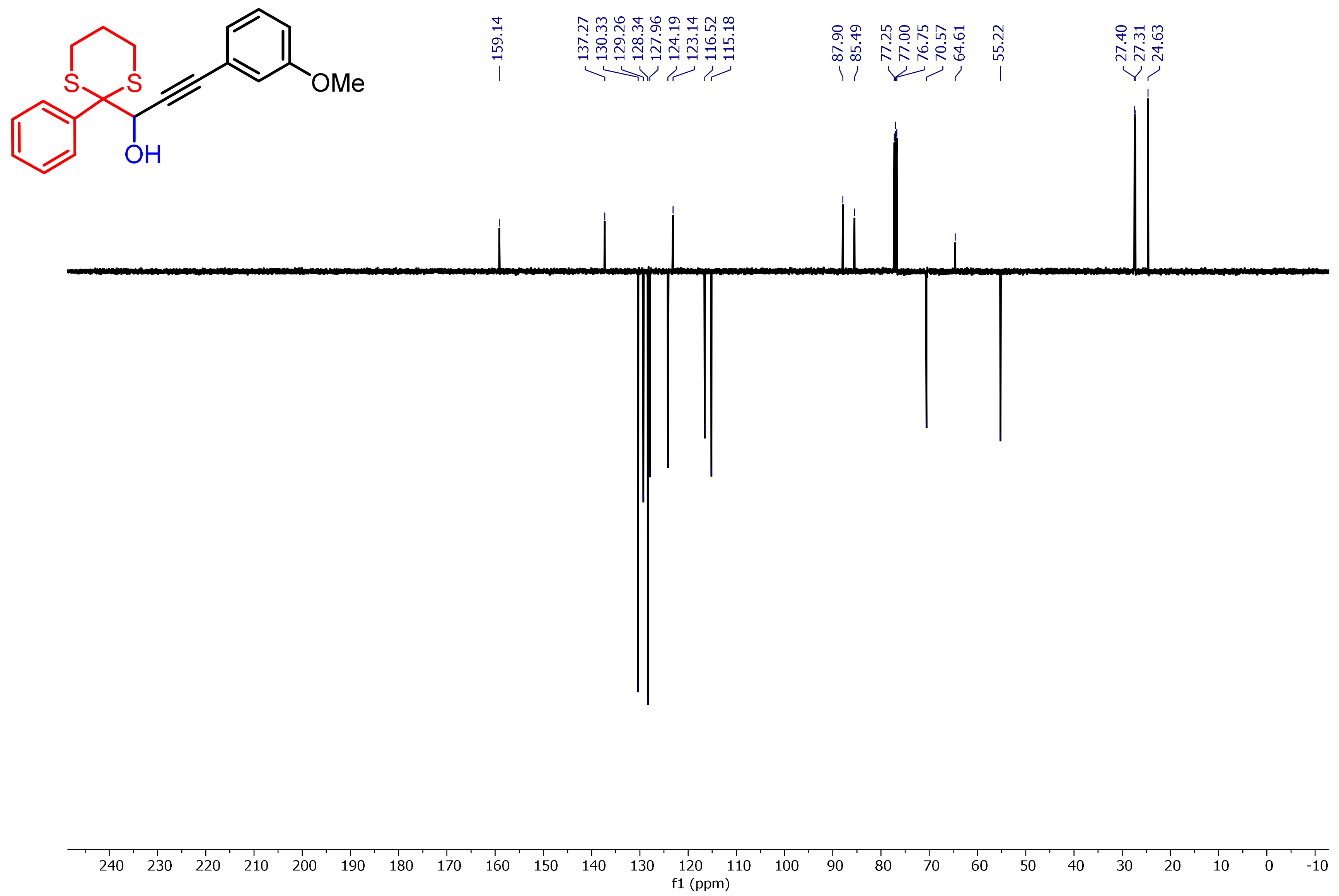

Figure S61. ^{1}H NMR ($CDCl_3$, 500 MHz) spectrum **1n**

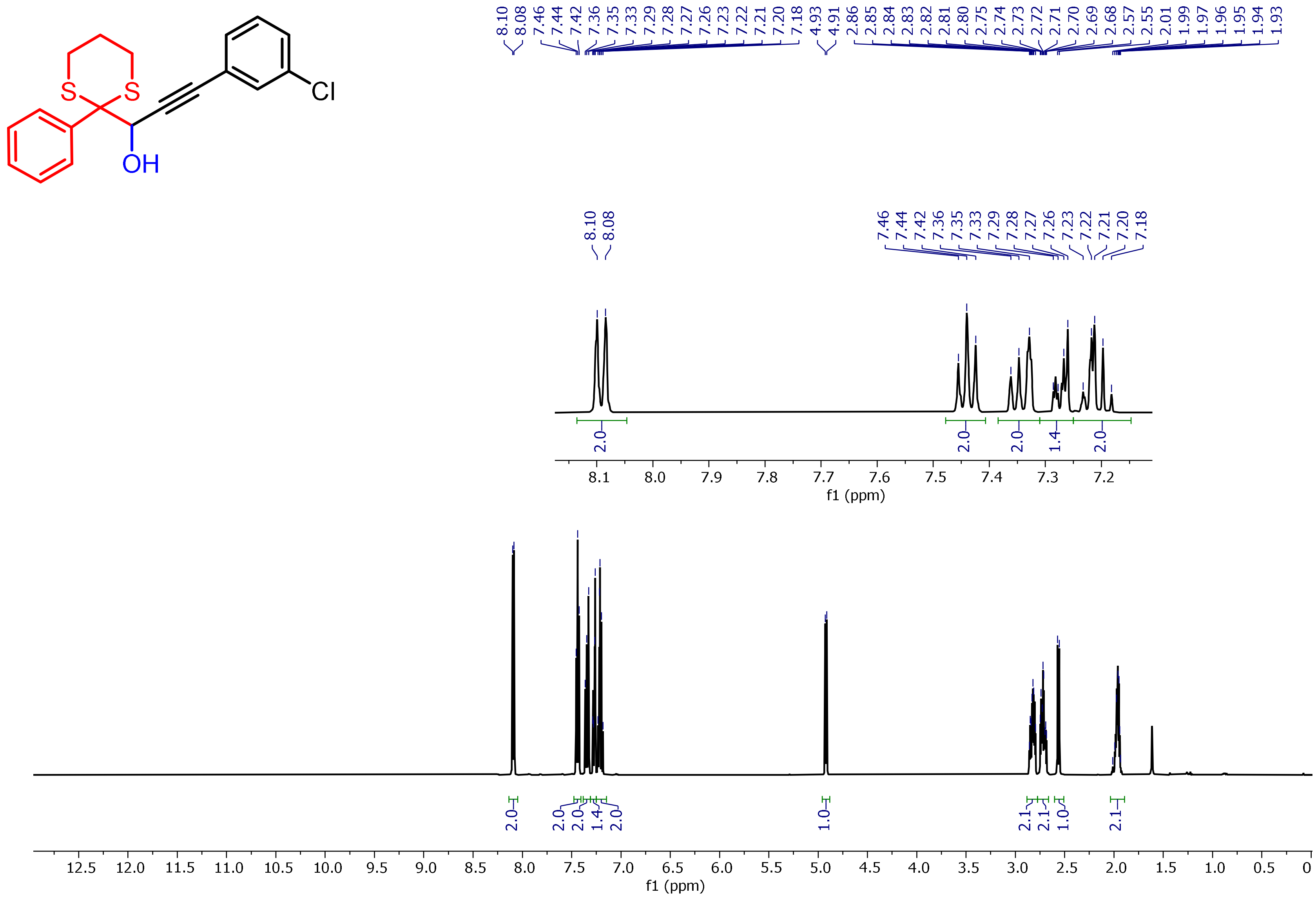

Figure S62. $^{13}C\{^{1}H\}$ NMR (126 MHz, $CDCl_3$, APT) spectrum **1n**

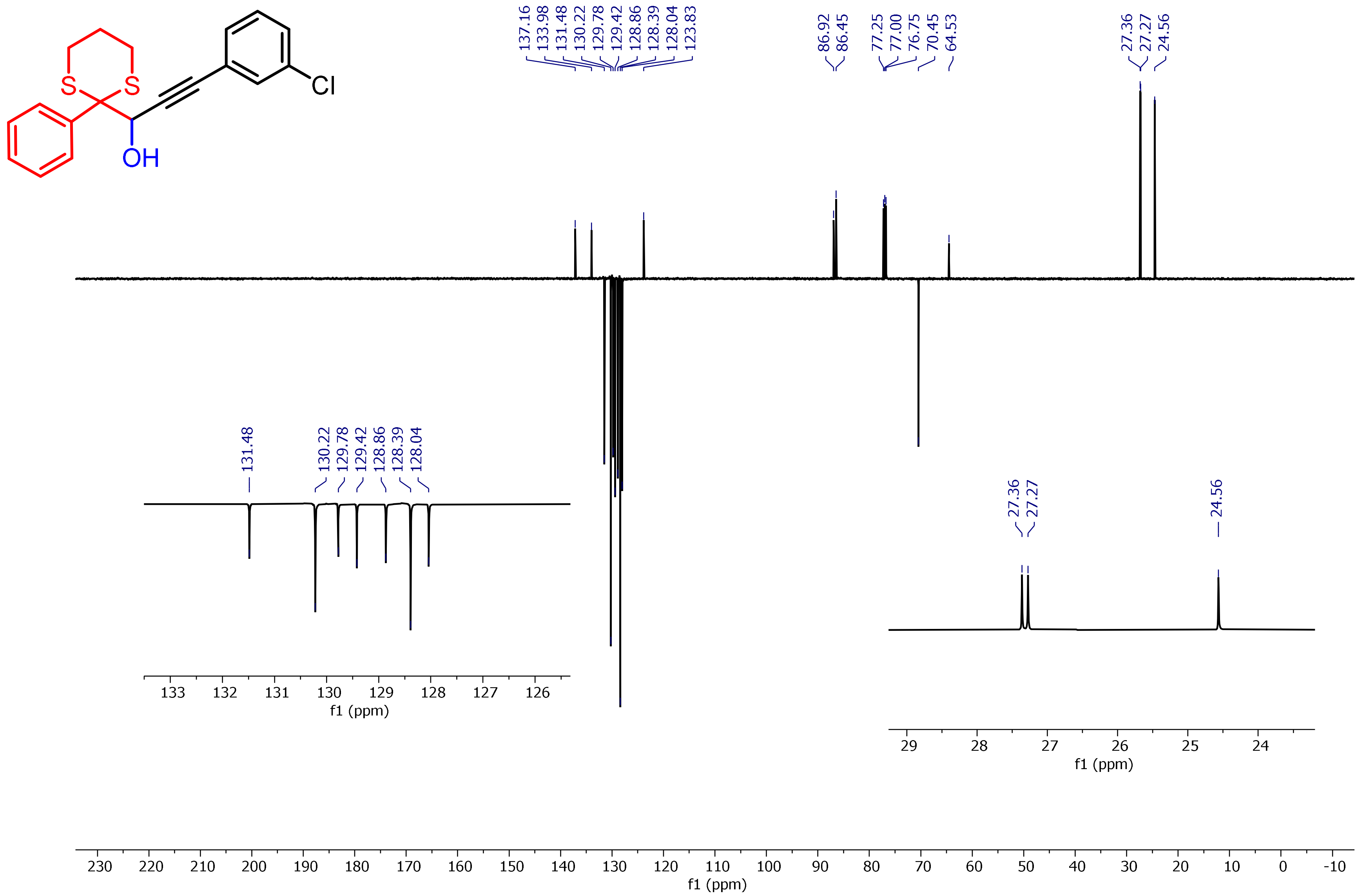

Figure S63. ^{1}H NMR ($CDCl_3$, 500 MHz) spectrum **1o**

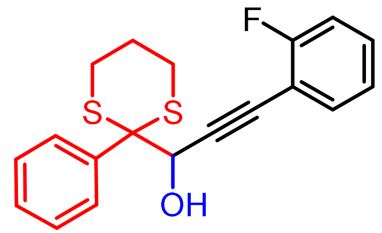

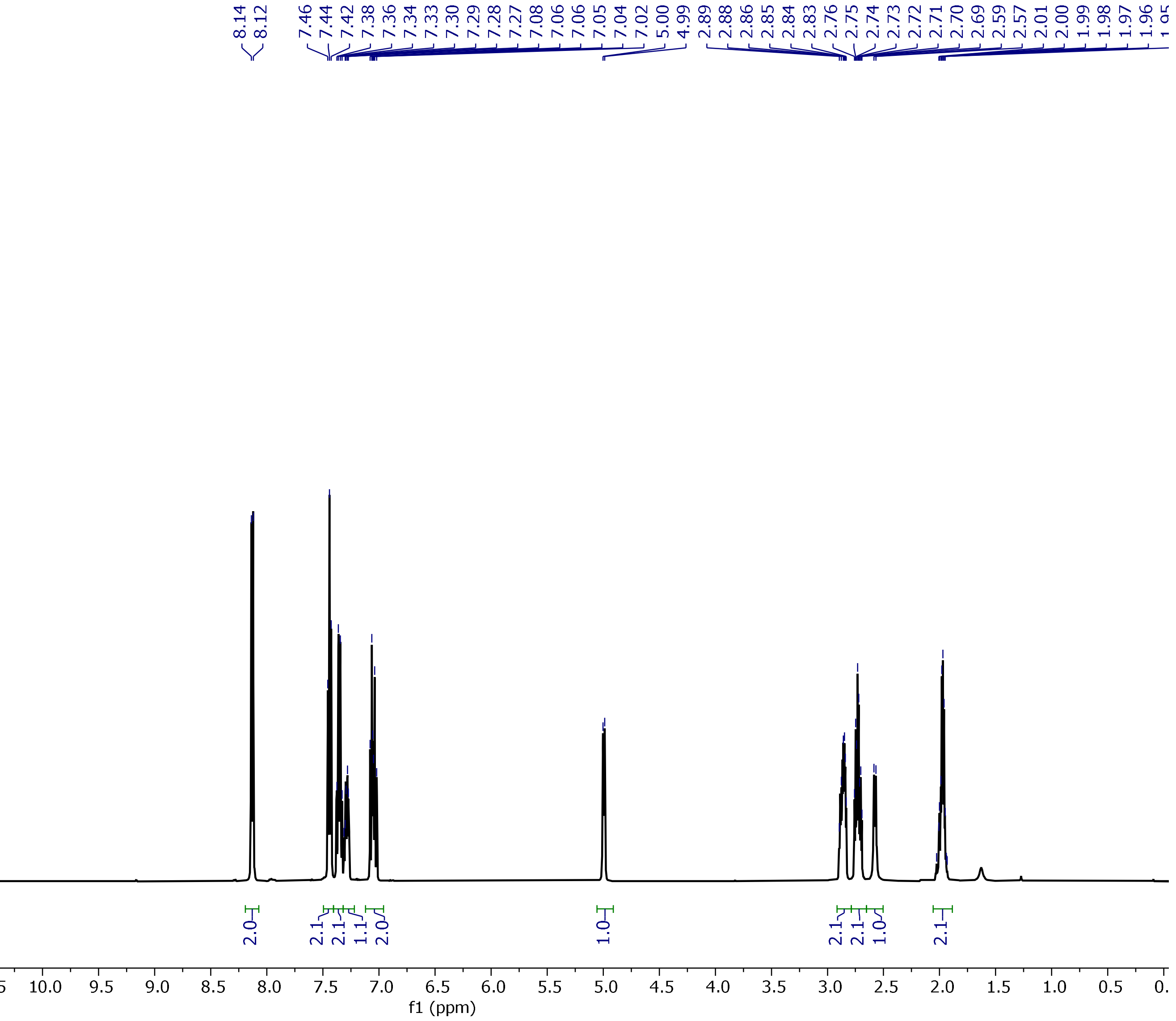

Figure S64. $^{13}C\{^1H\}$NMR (126 MHz, $CDCl_3$, APT) spectrum **1o**

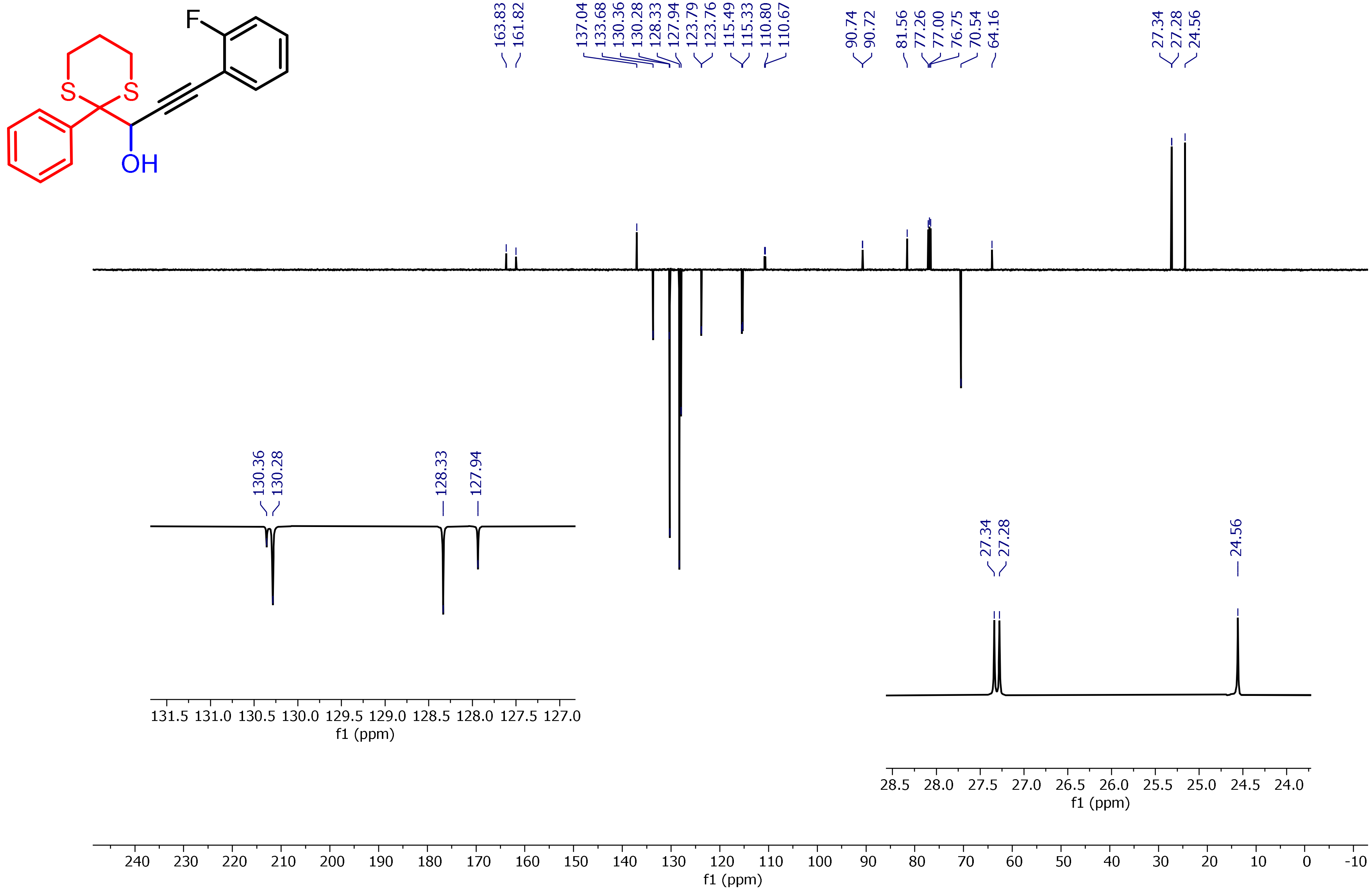

Figure S65. ^{1}H NMR ($CDCl_3$, 500 MHz) spectrum **1q**

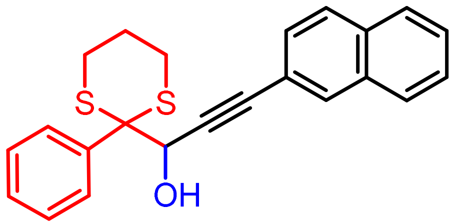

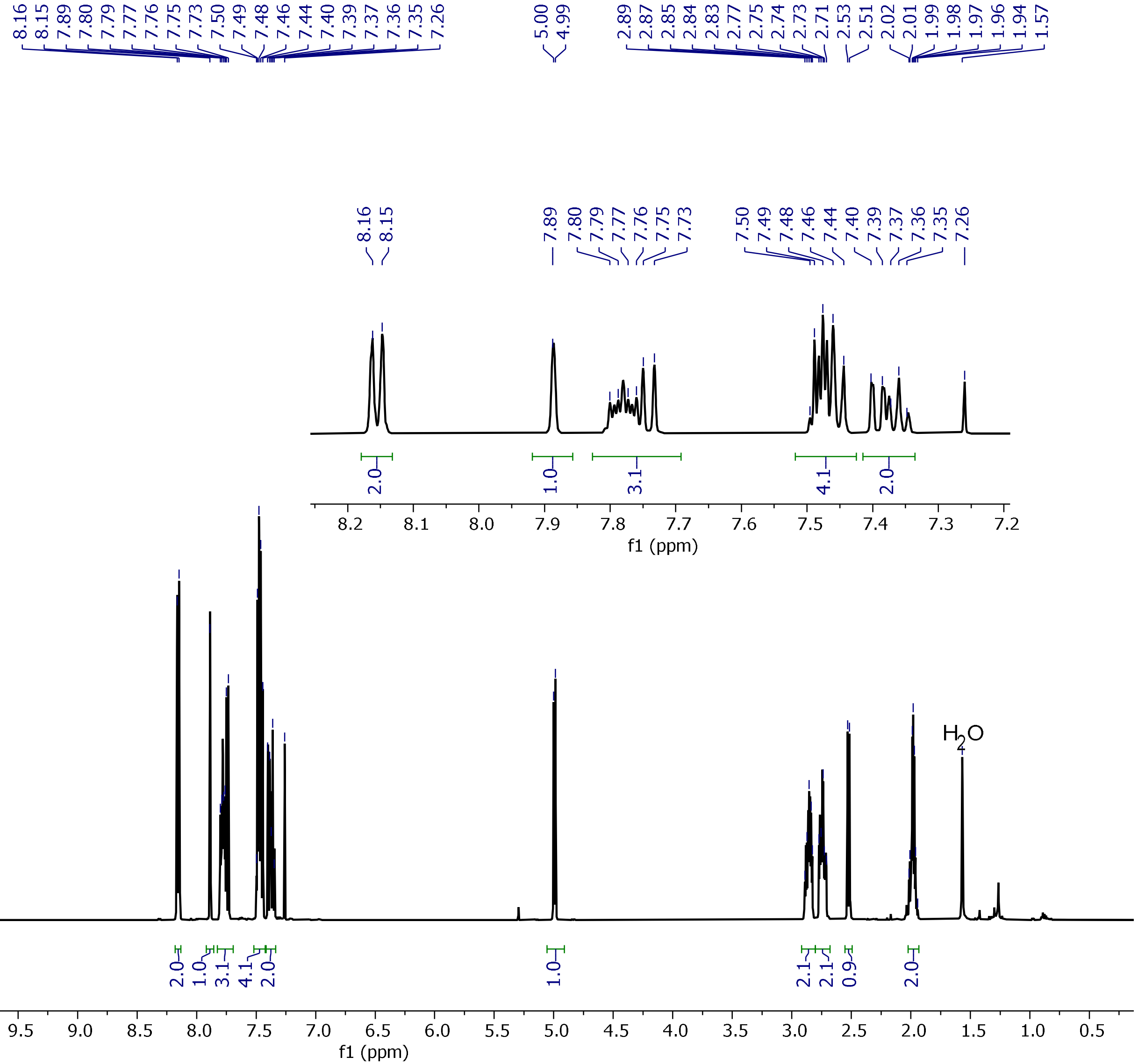

Figure S66. $^{13}C\{^1H\}$NMR (126 MHz, $CDCl_3$, APT) spectrum **1q**

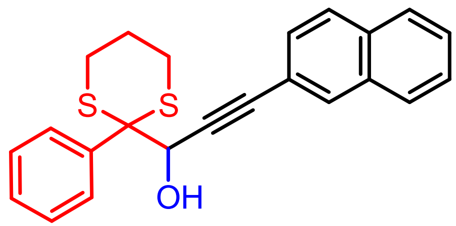

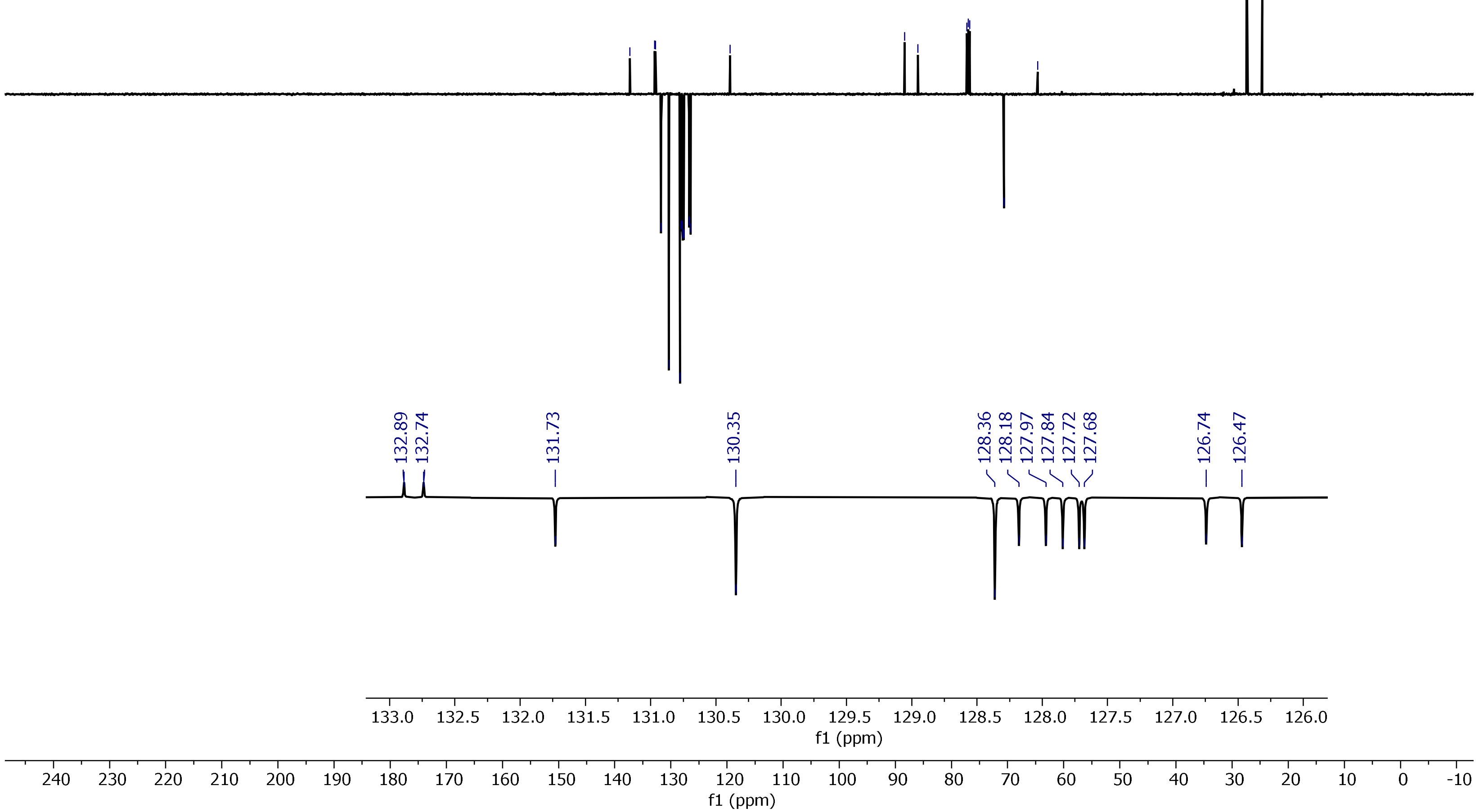

Figure S67. ^{1}H NMR ($CDCl_3$, 500 MHz) spectrum **1p**

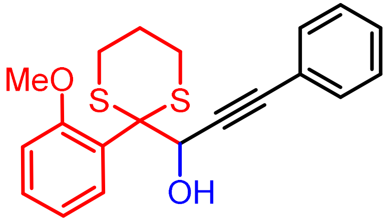

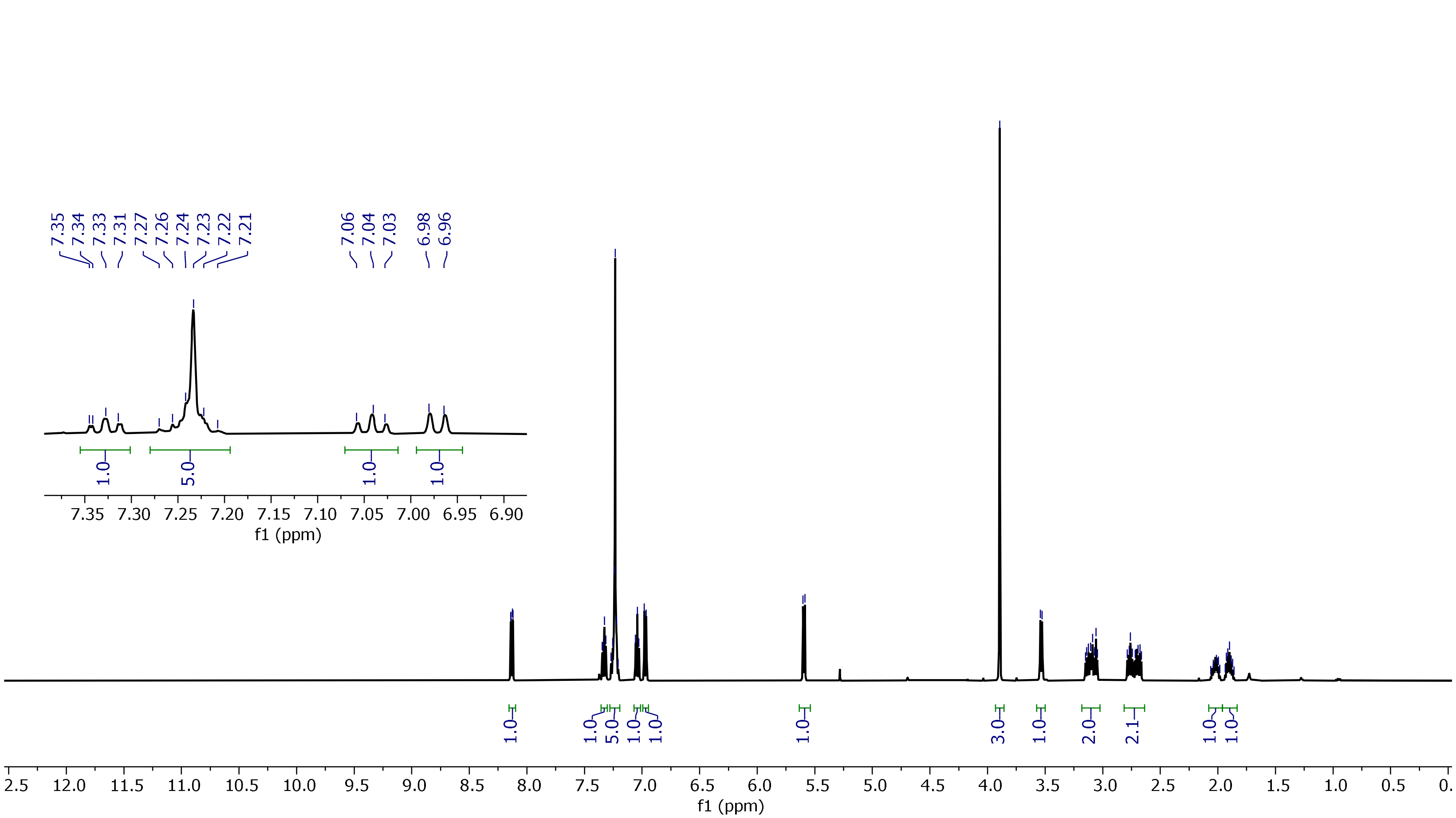

Figure S68. $^{13}C\{^{1}H\}$NMR (126 MHz, $CDCl_3$, APT) spectrum **1p**

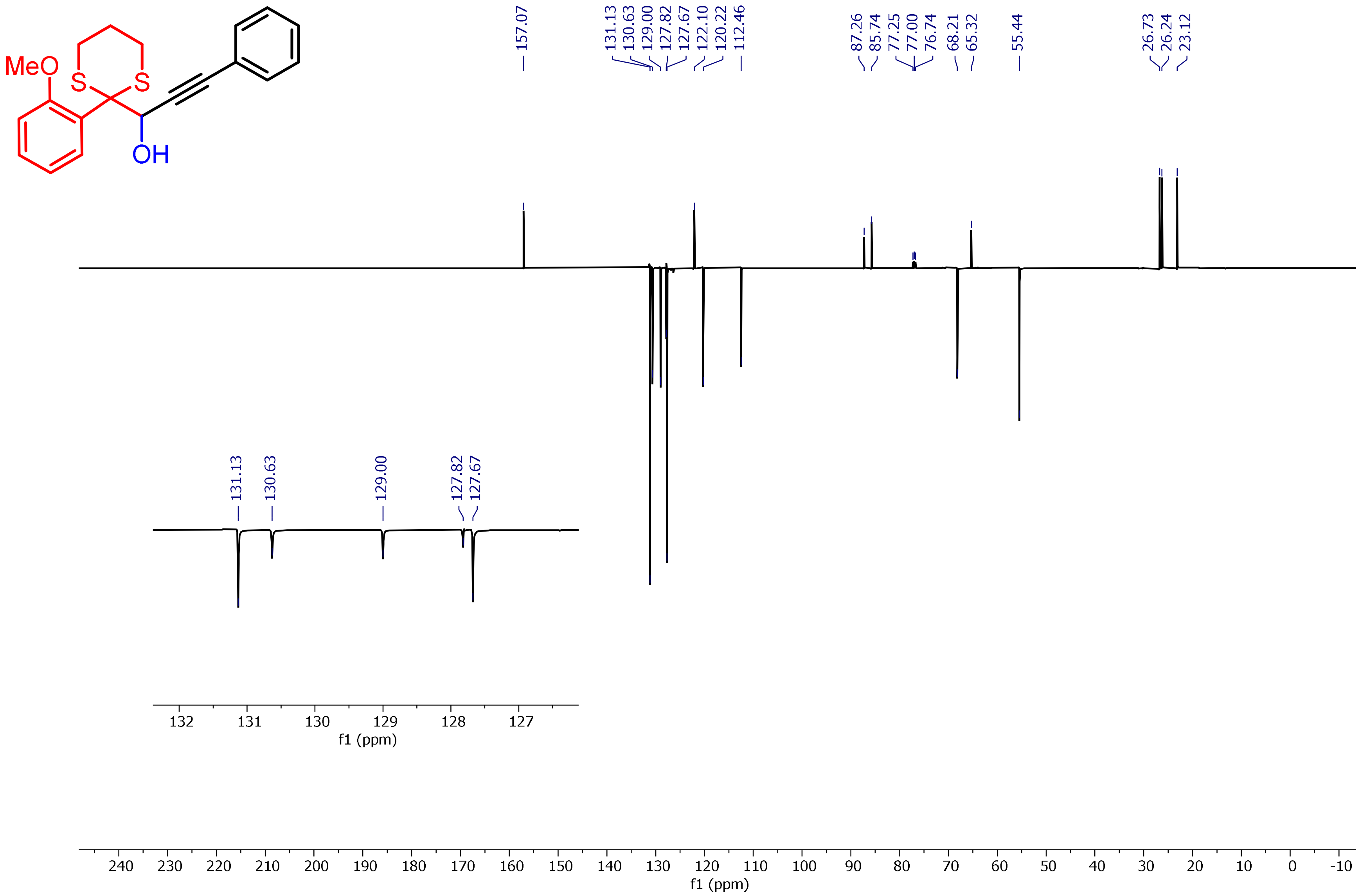

Figure S69. ^{1}H NMR ($CDCl_3$, 500 MHz) spectrum **1r**

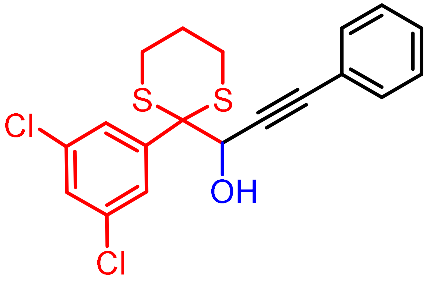

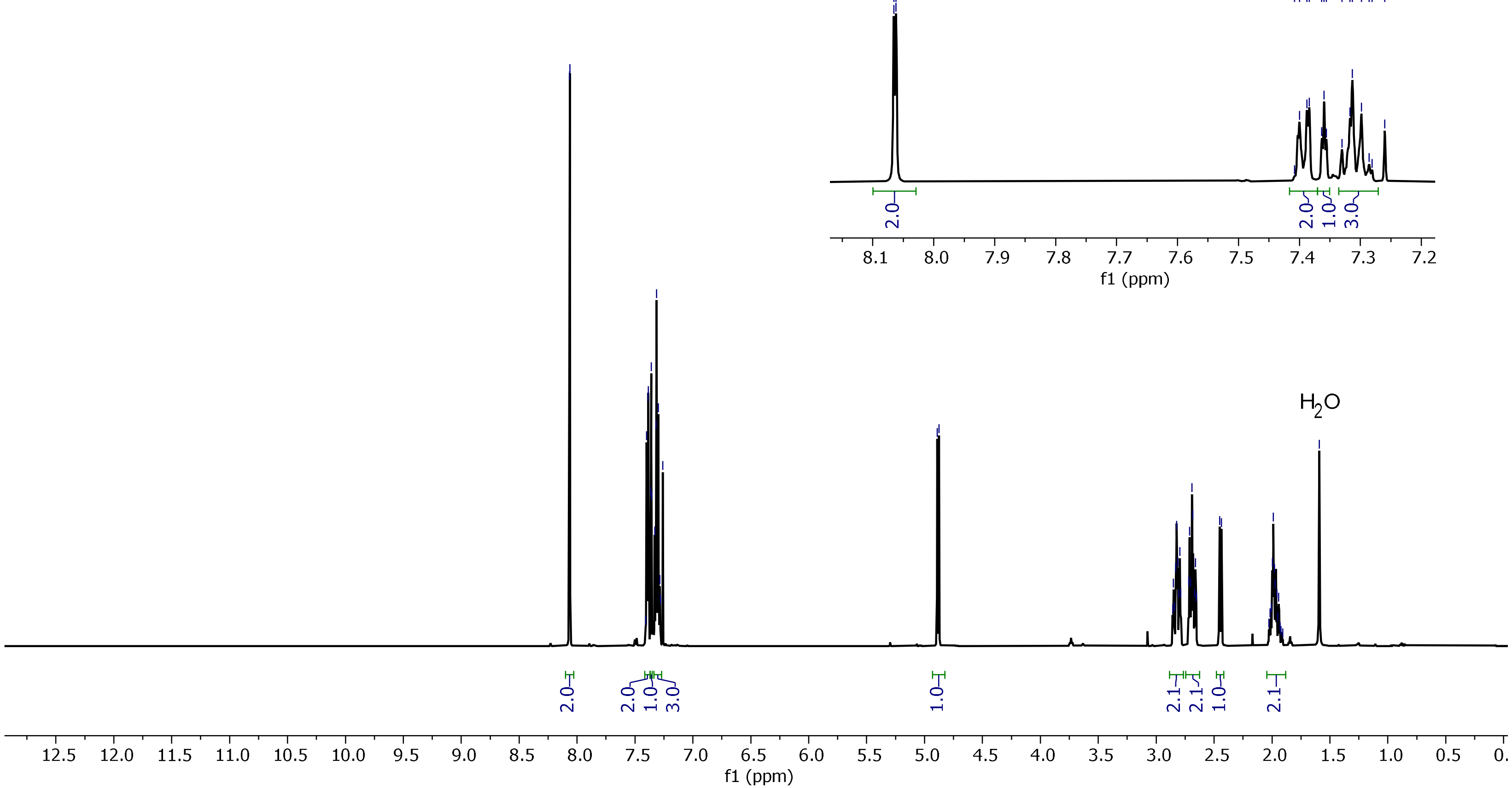

Figure S70. $^{13}C\{^{1}H\}$ NMR (126 MHz, $CDCl_3$, APT) spectrum **1r**

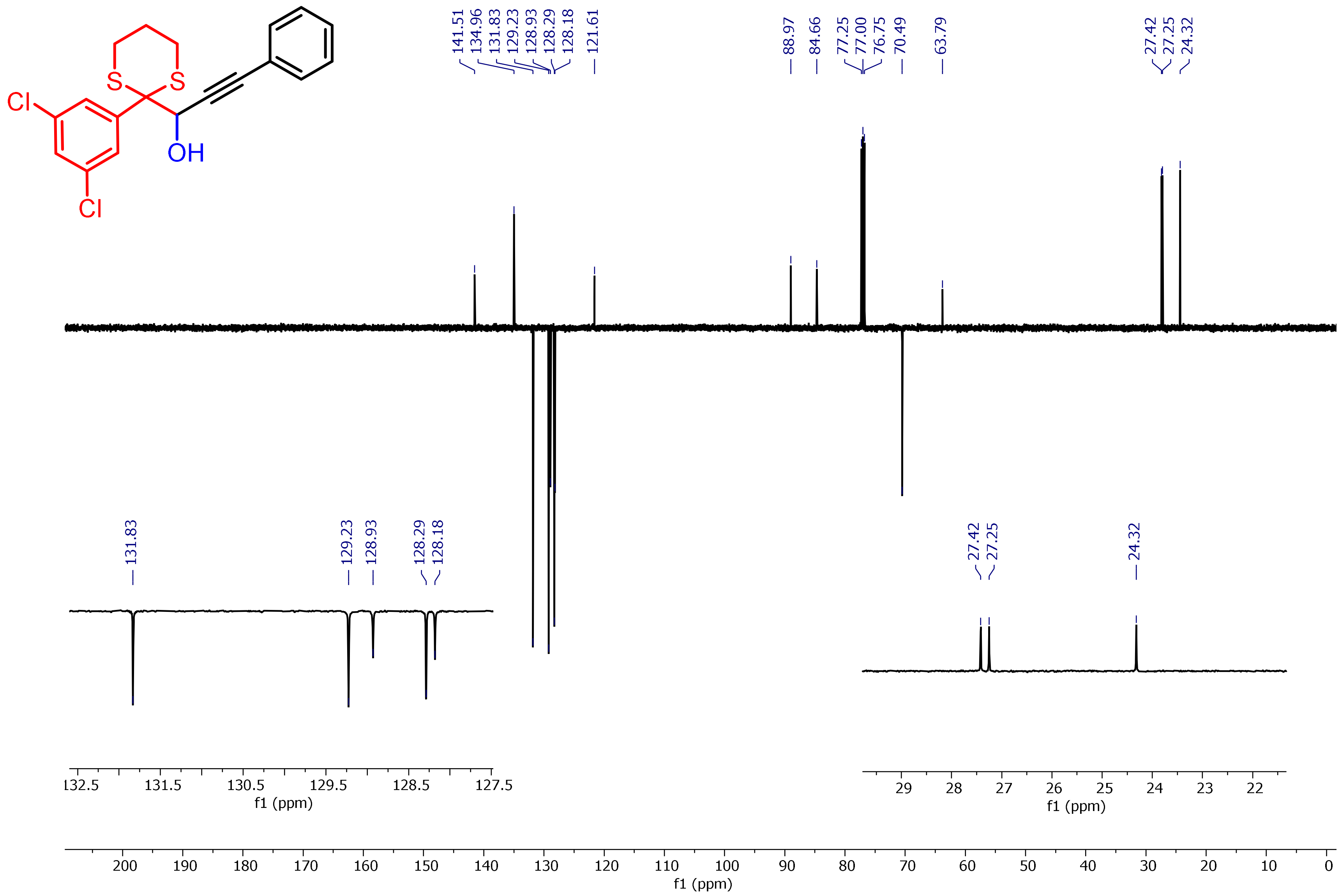

Figure S71. ^{1}H NMR ($CDCl_3$, 500 MHz) spectrum **1s**

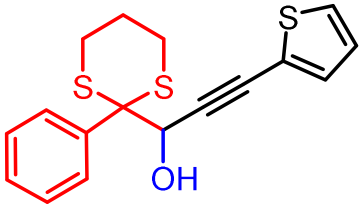

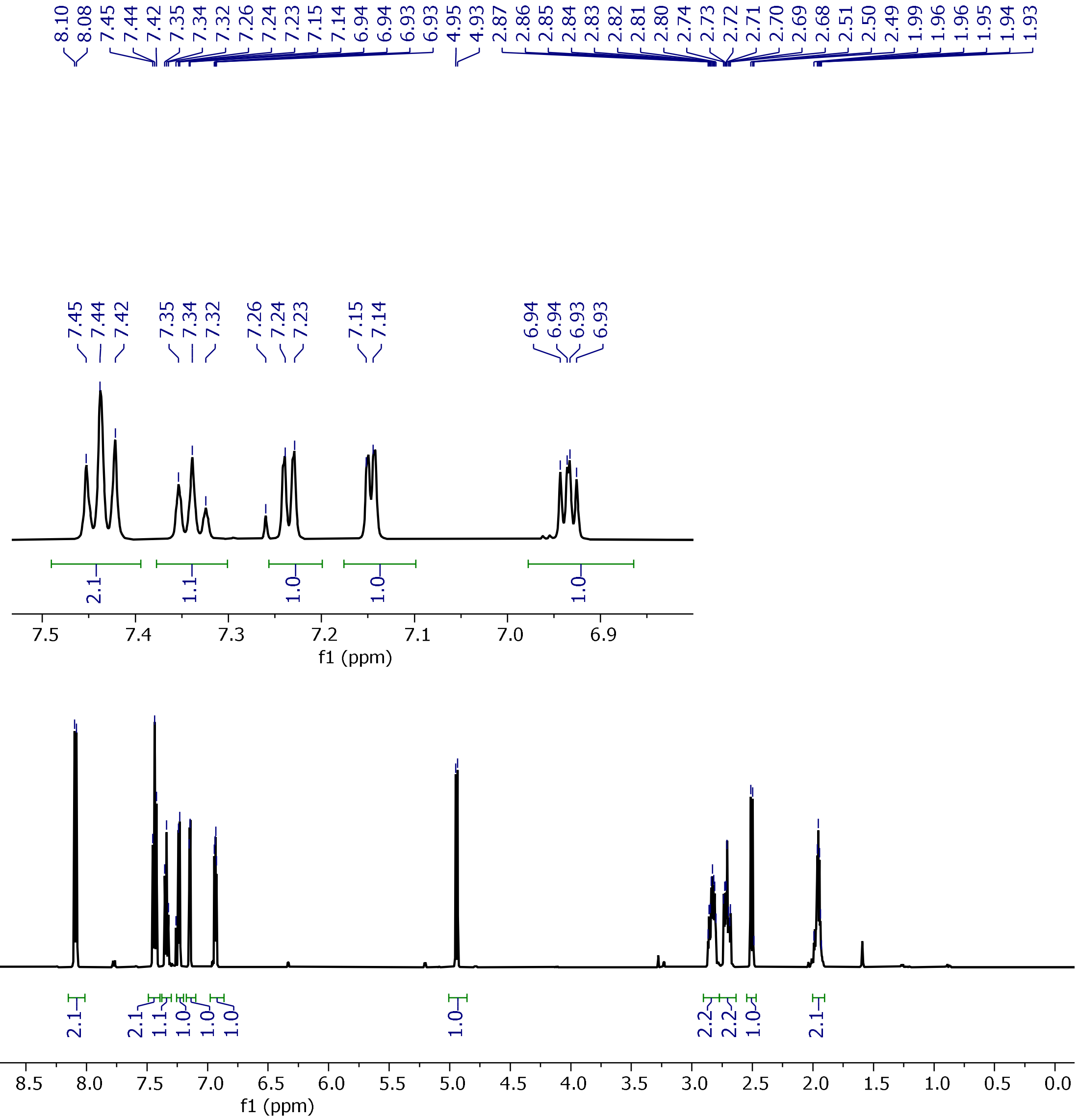

Figure S72. $^{13}C\{^{1}H\}$ NMR (126 MHz, $CDCl_3$, APT) spectrum **1s**

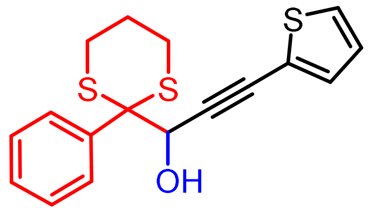

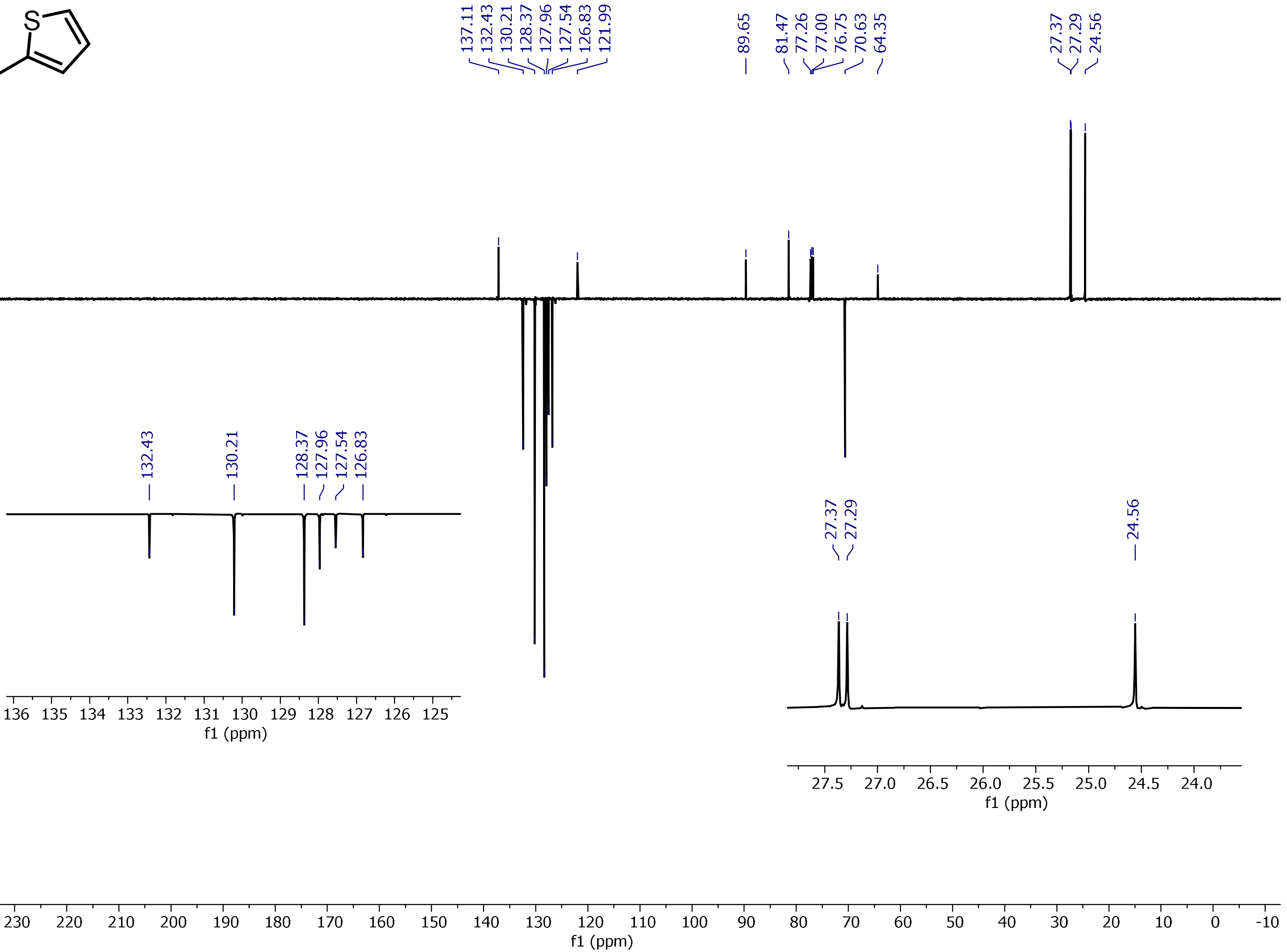

Figure S73. ^{1}H NMR ($CDCl_3$, 500 MHz) spectrum **1t**

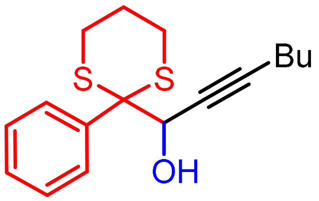

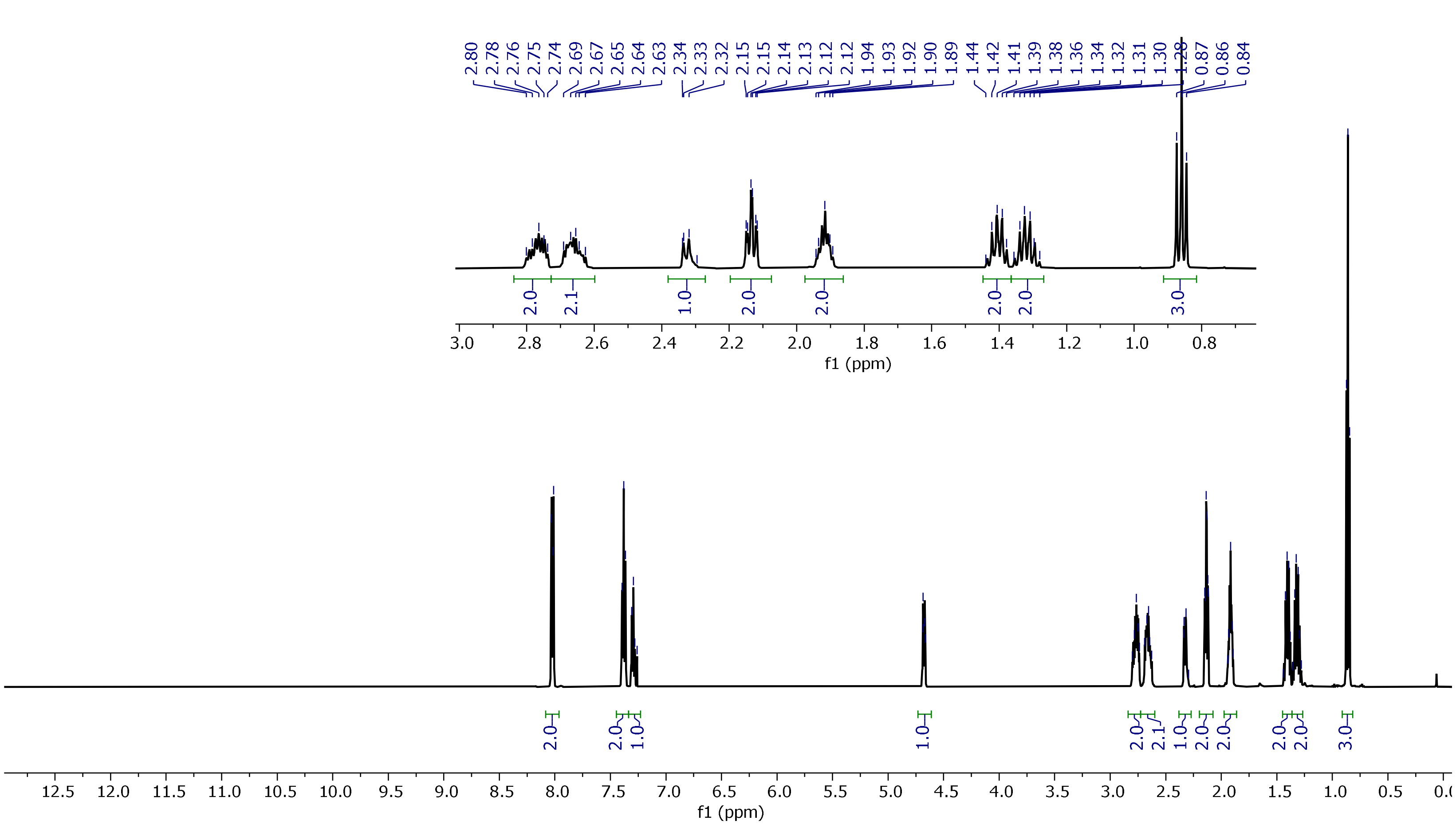

Figure S74. $^{13}C\{^{1}H\}$NMR (126 MHz, $CDCl_3$, APT) spectrum **1t**

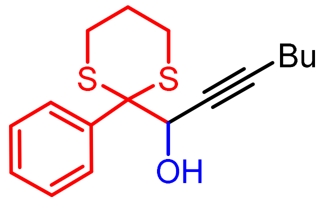

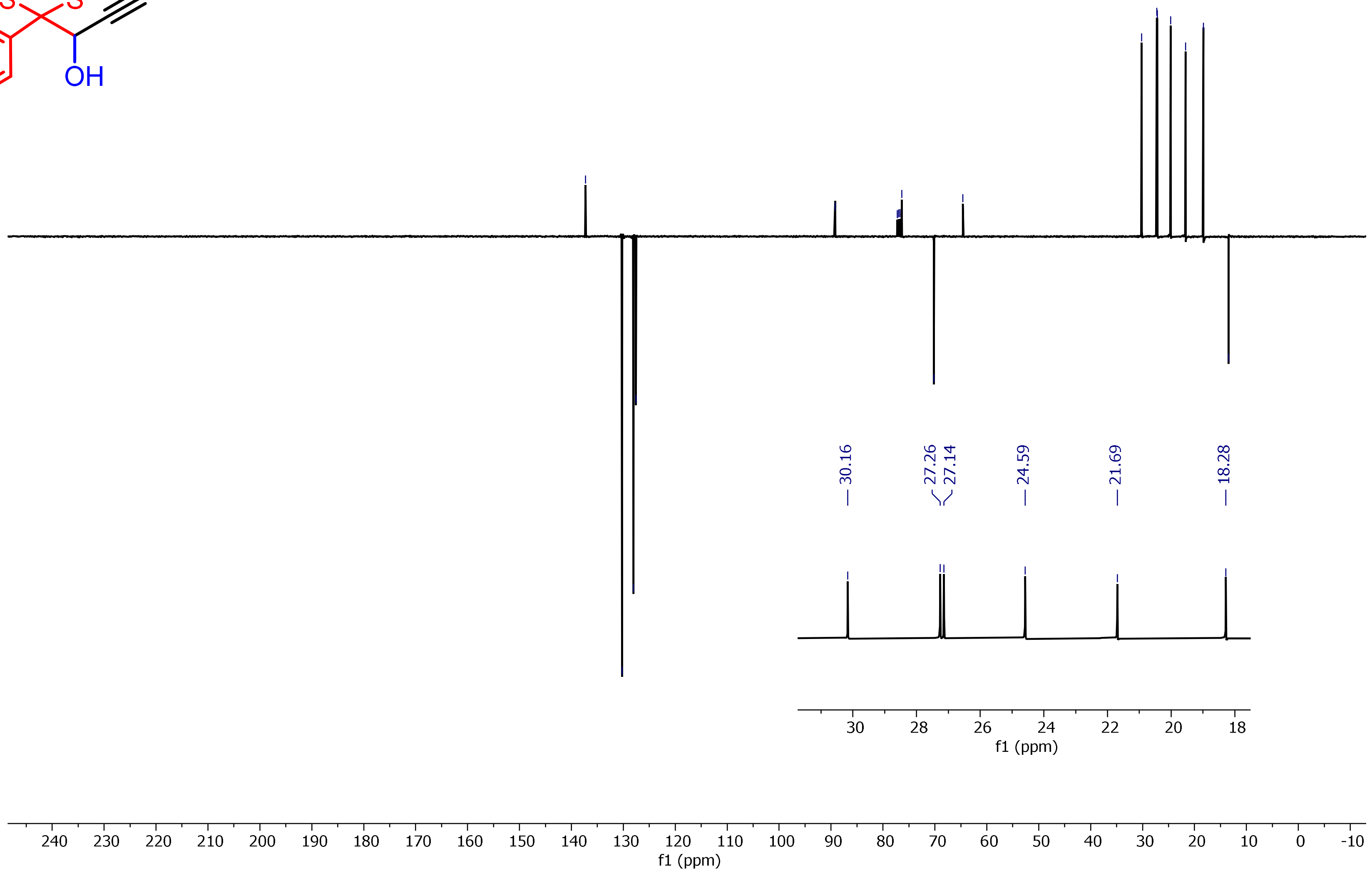

Figure S75. ^{1}H NMR ($CDCl_3$, 500 MHz) spectrum **1u**

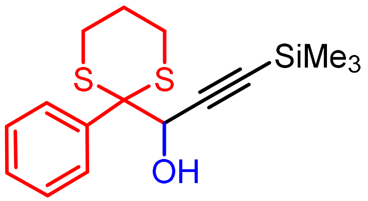

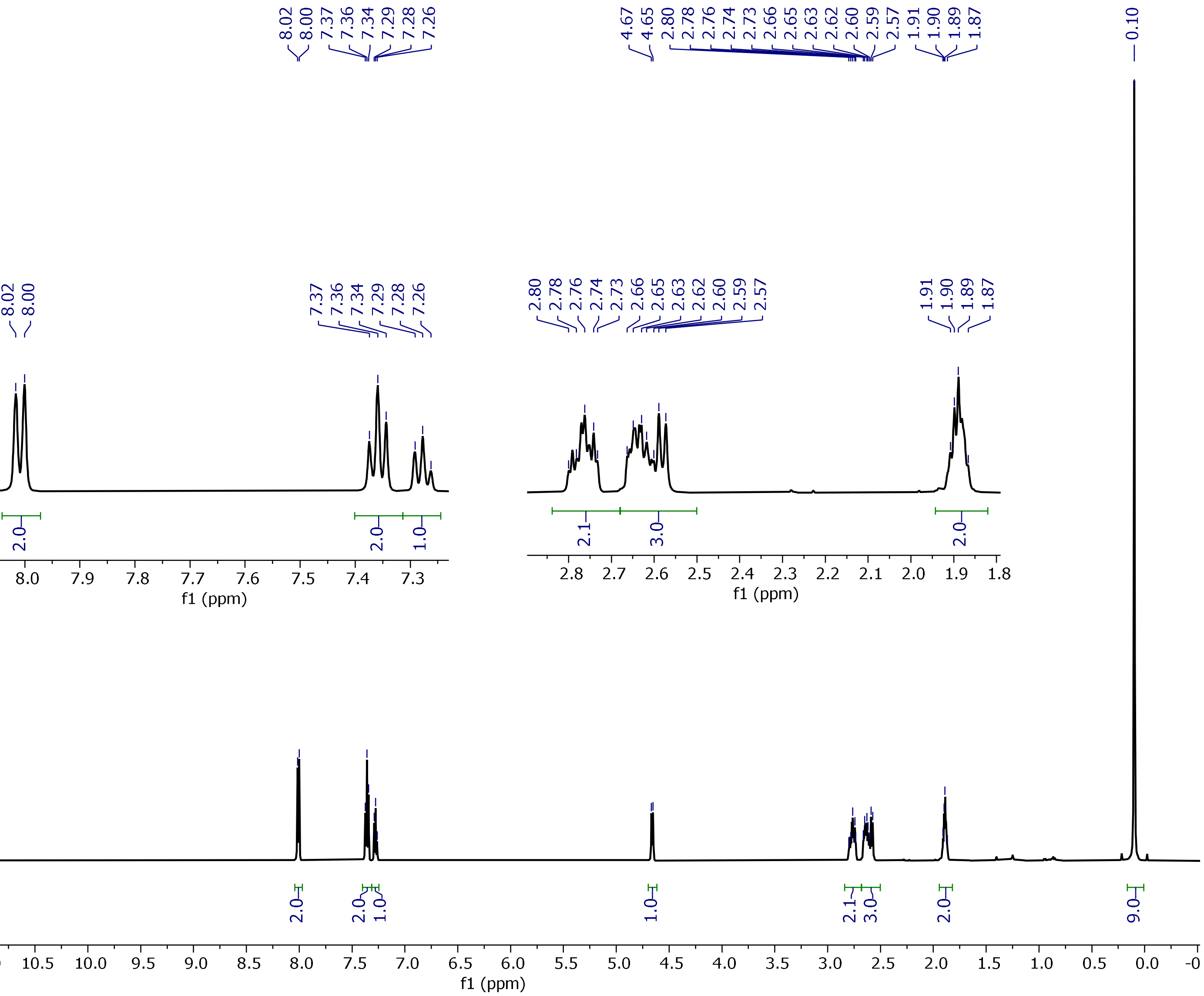

Figure S76. $^{13}C\{^{1}H\}$ NMR (126 MHz, $CDCl_3$, APT) spectrum **1u**

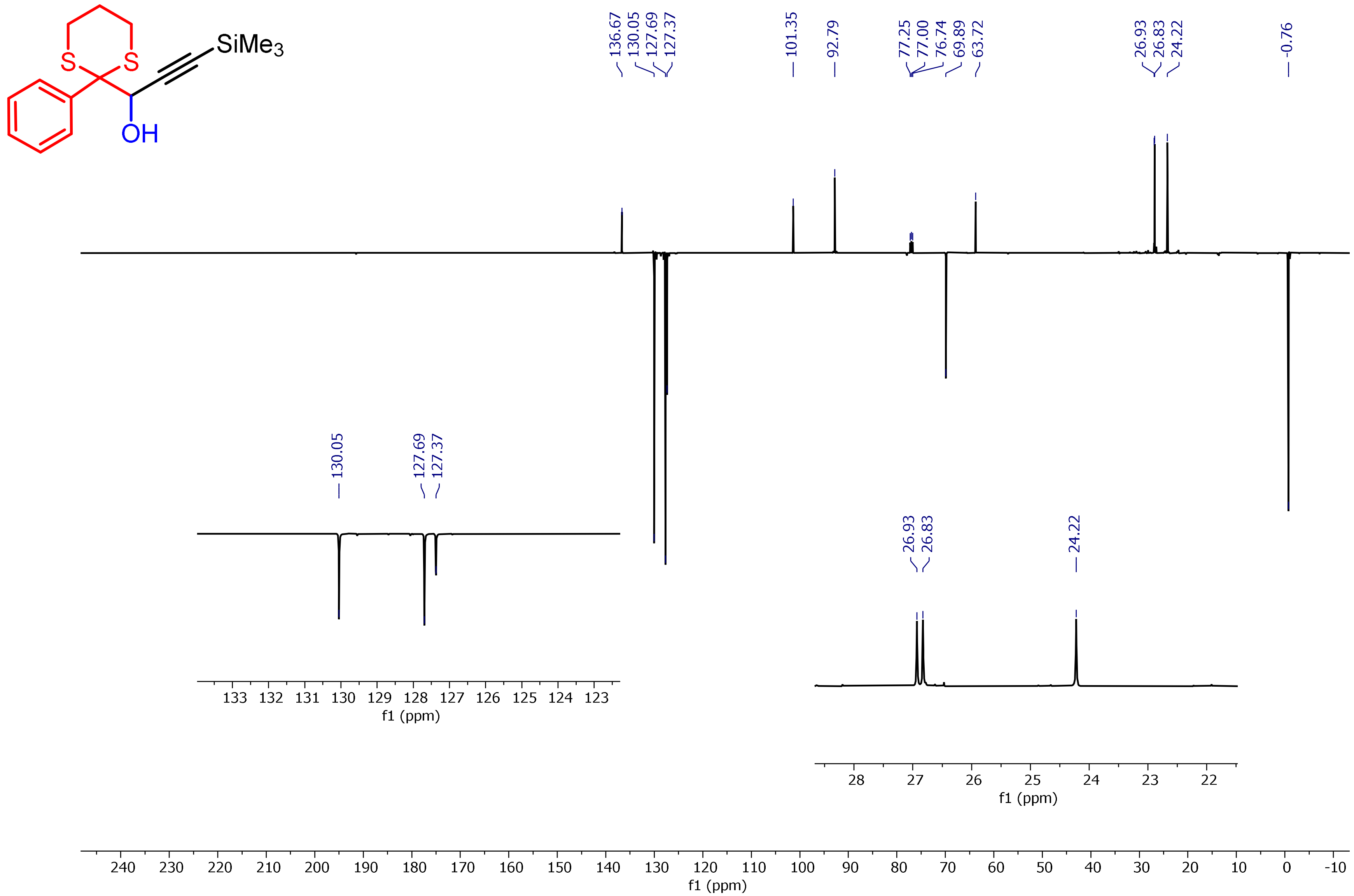

Figure S77. ^{1}H NMR ($CDCl_3$, 500 MHz) spectrum **1v**

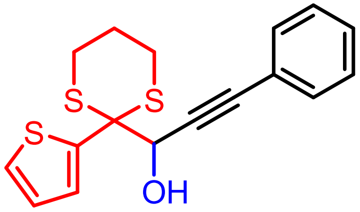

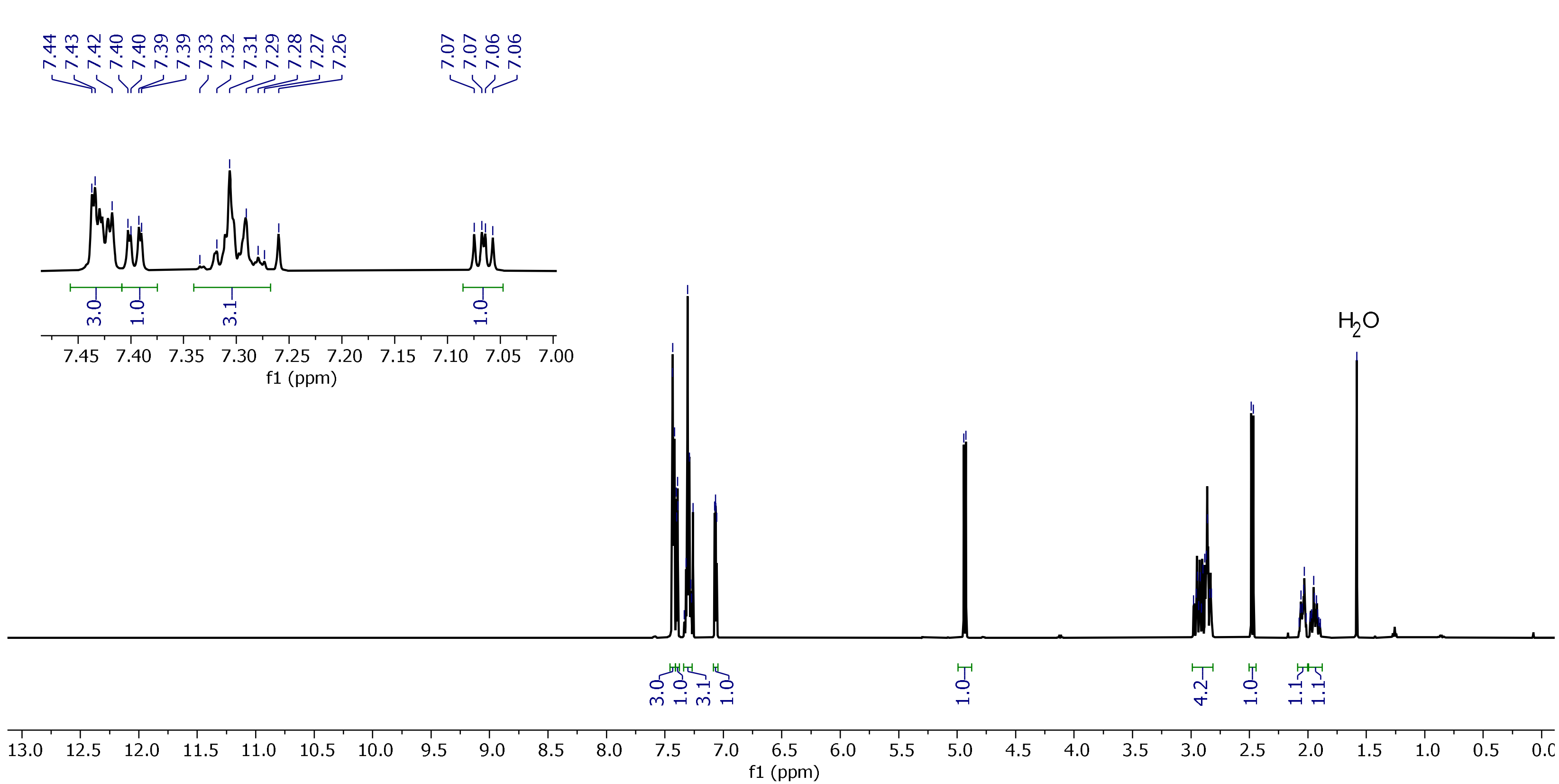

Figure S78. $^{13}C\{^1H\}$ NMR (126 MHz, $CDCl_3$, APT) spectrum **1v**

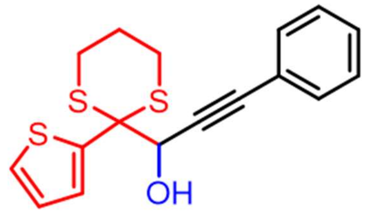

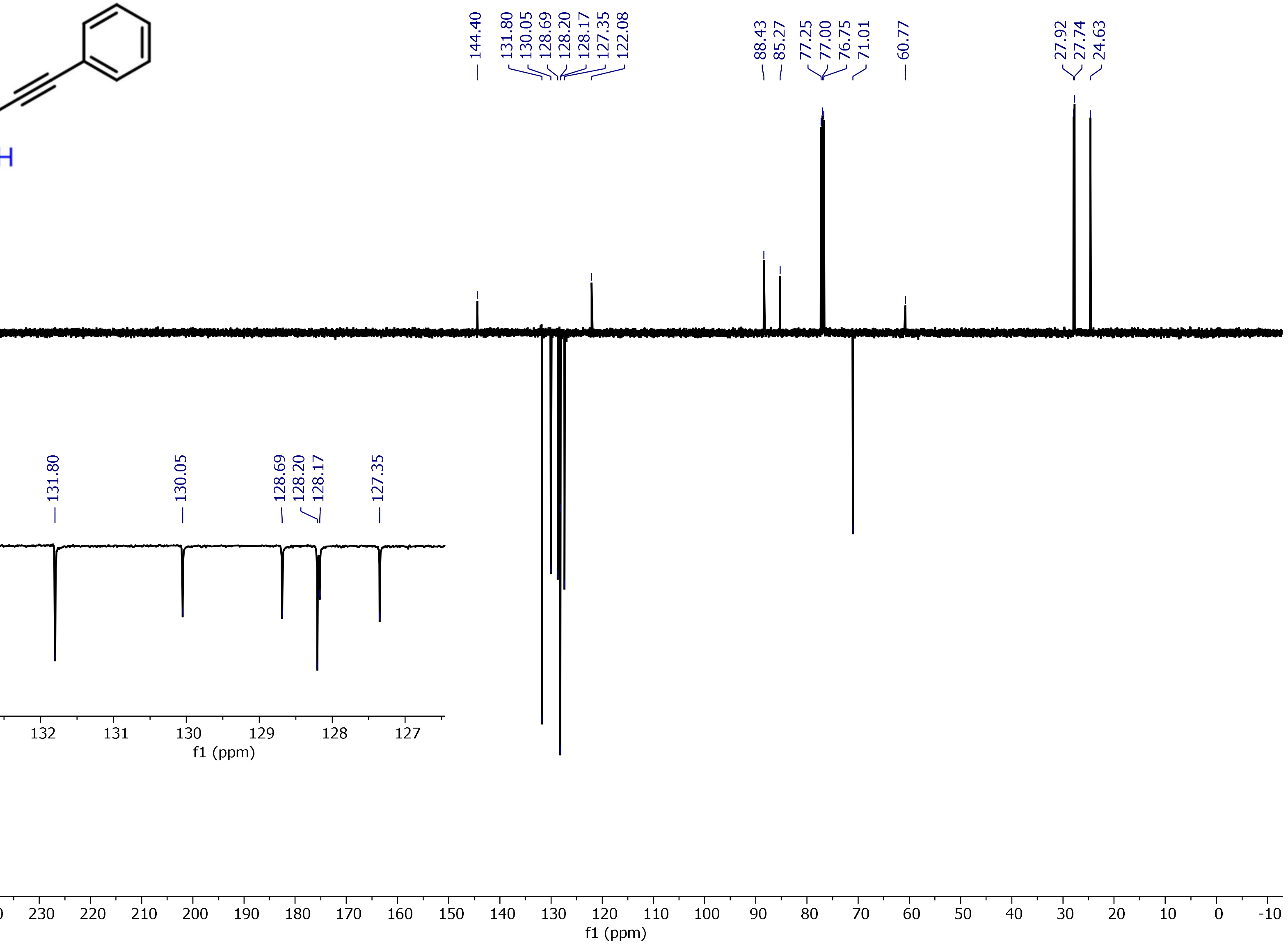

Figure S79. ^{1}H NMR ($CDCl_3$, 500 MHz) spectrum **3a**

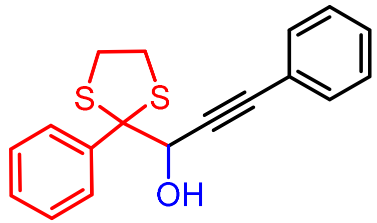

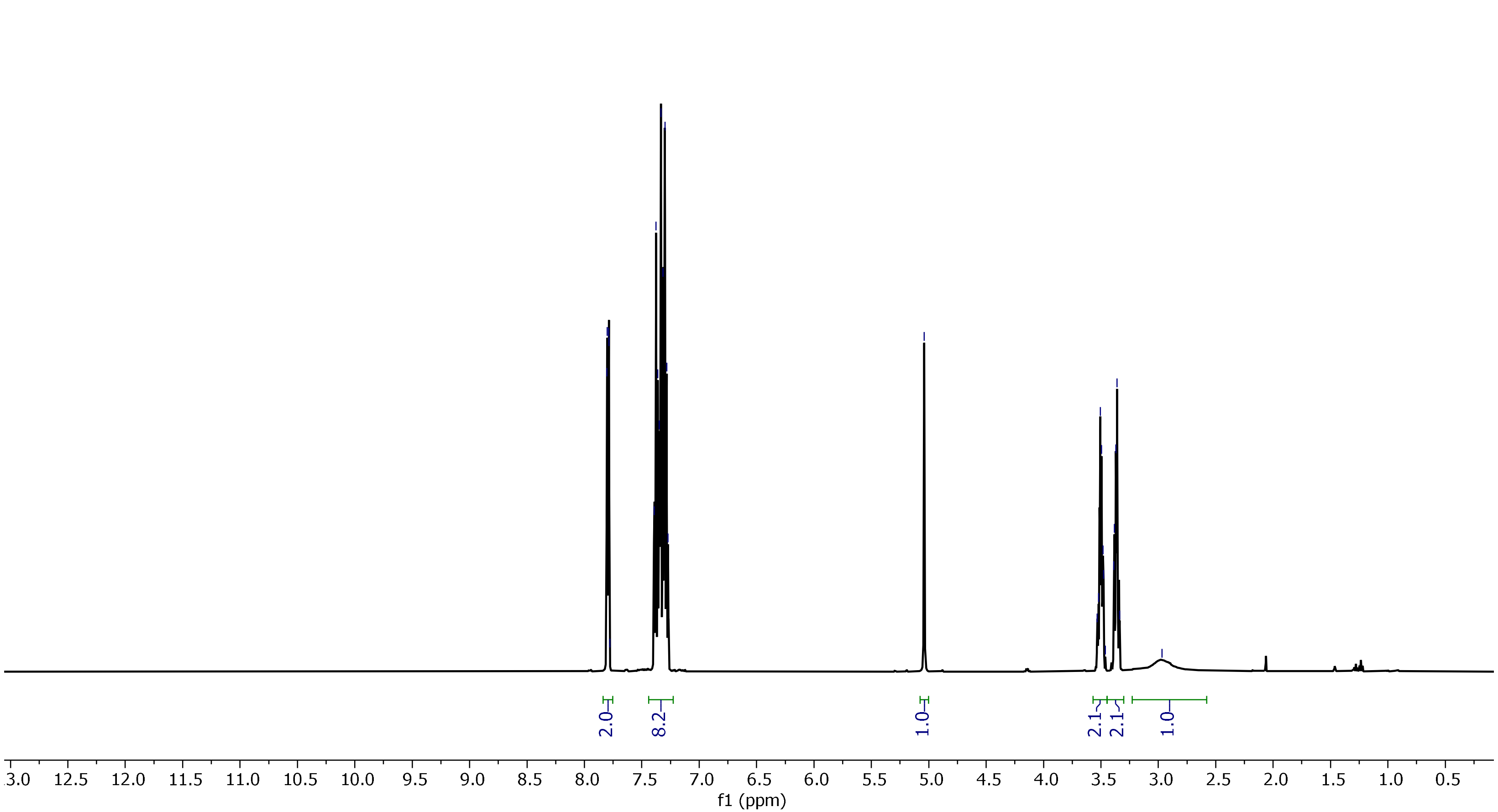

Figure S80. $^{13}C\{^{1}H\}$ NMR (126 MHz, $CDCl_3$, APT) spectrum **3a**

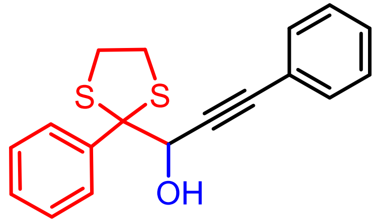

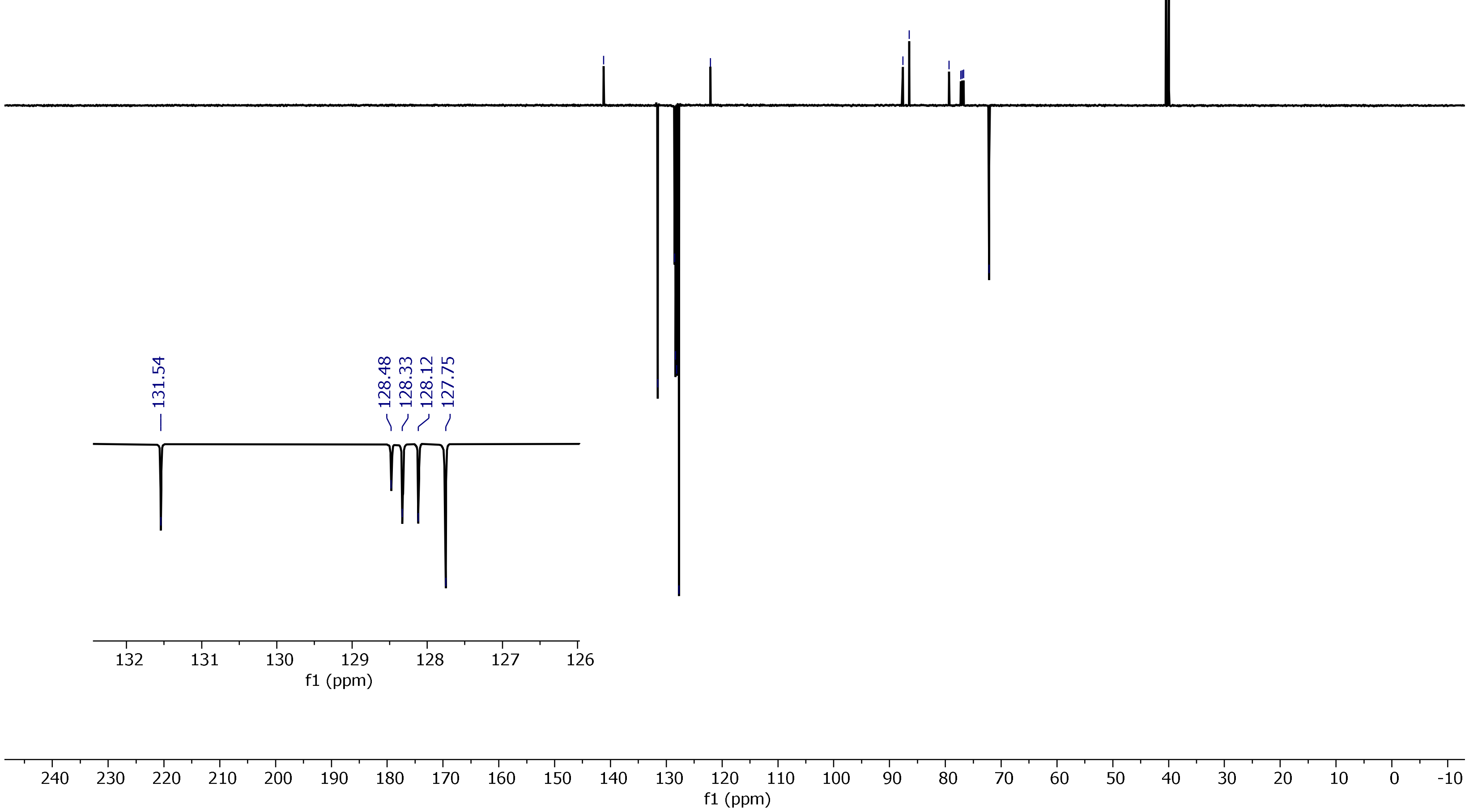

Figure S81. ^{1}H NMR ($CDCl_3$, 500 MHz) spectrum **3b**

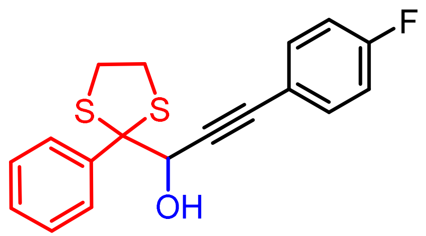

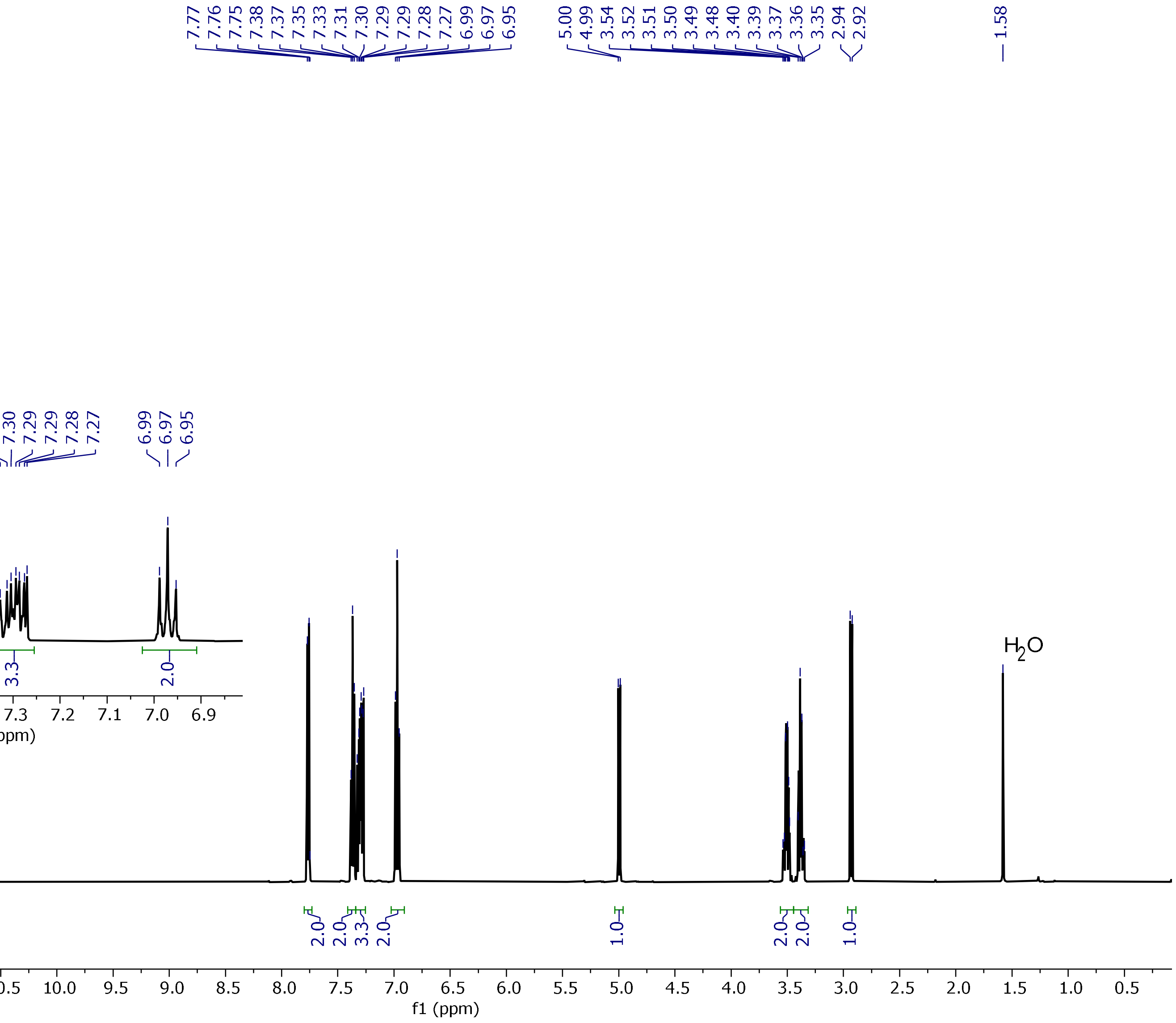

Figure S82. $^{13}C\{^1H\}$ NMR (126 MHz, $CDCl_3$, APT) spectrum **3b**

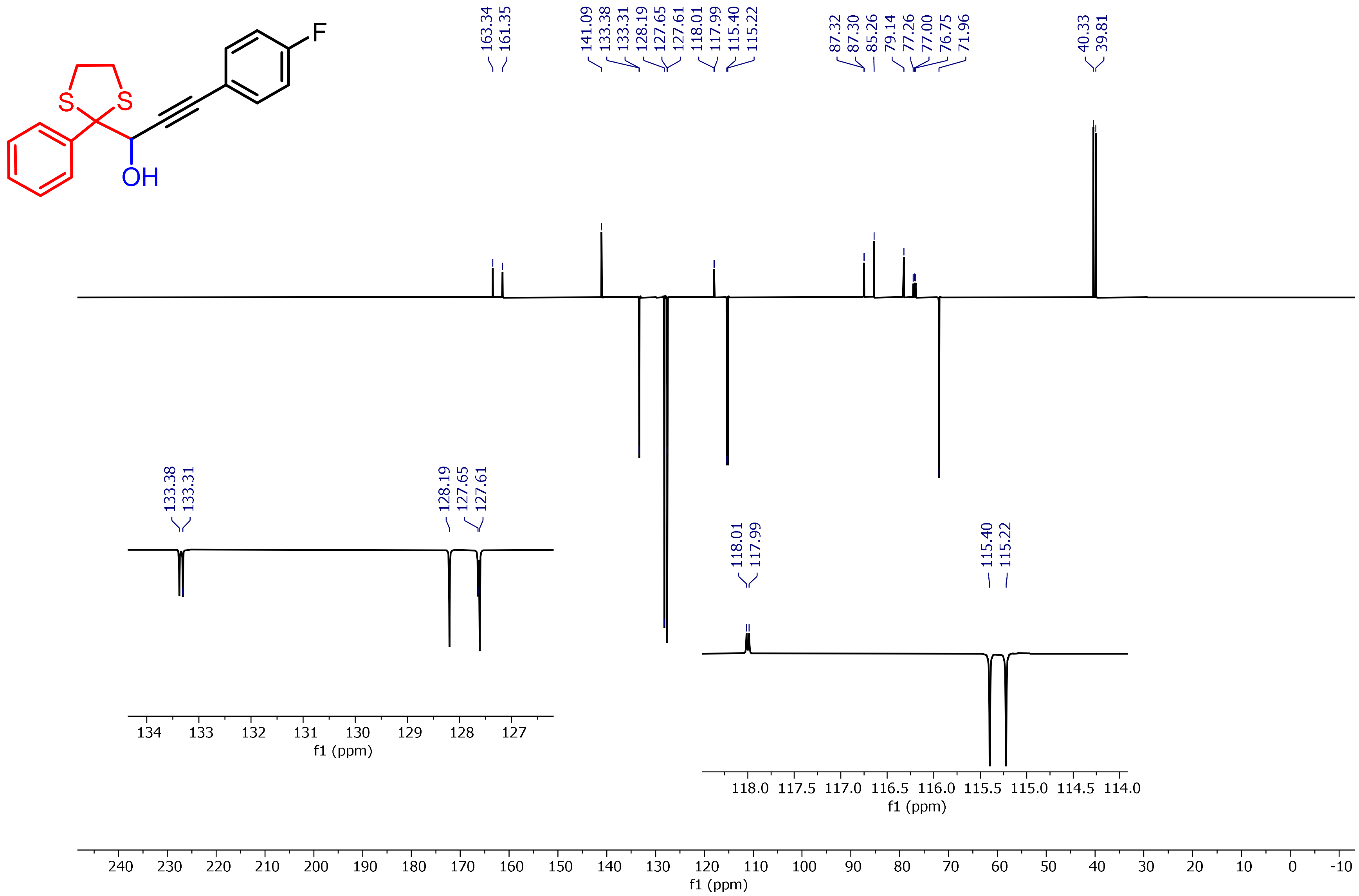

Figure S83. ^{1}H NMR ($CDCl_3$, 500 MHz) spectrum **3c**

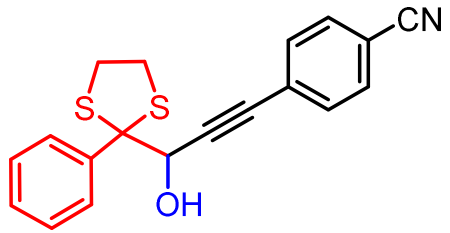

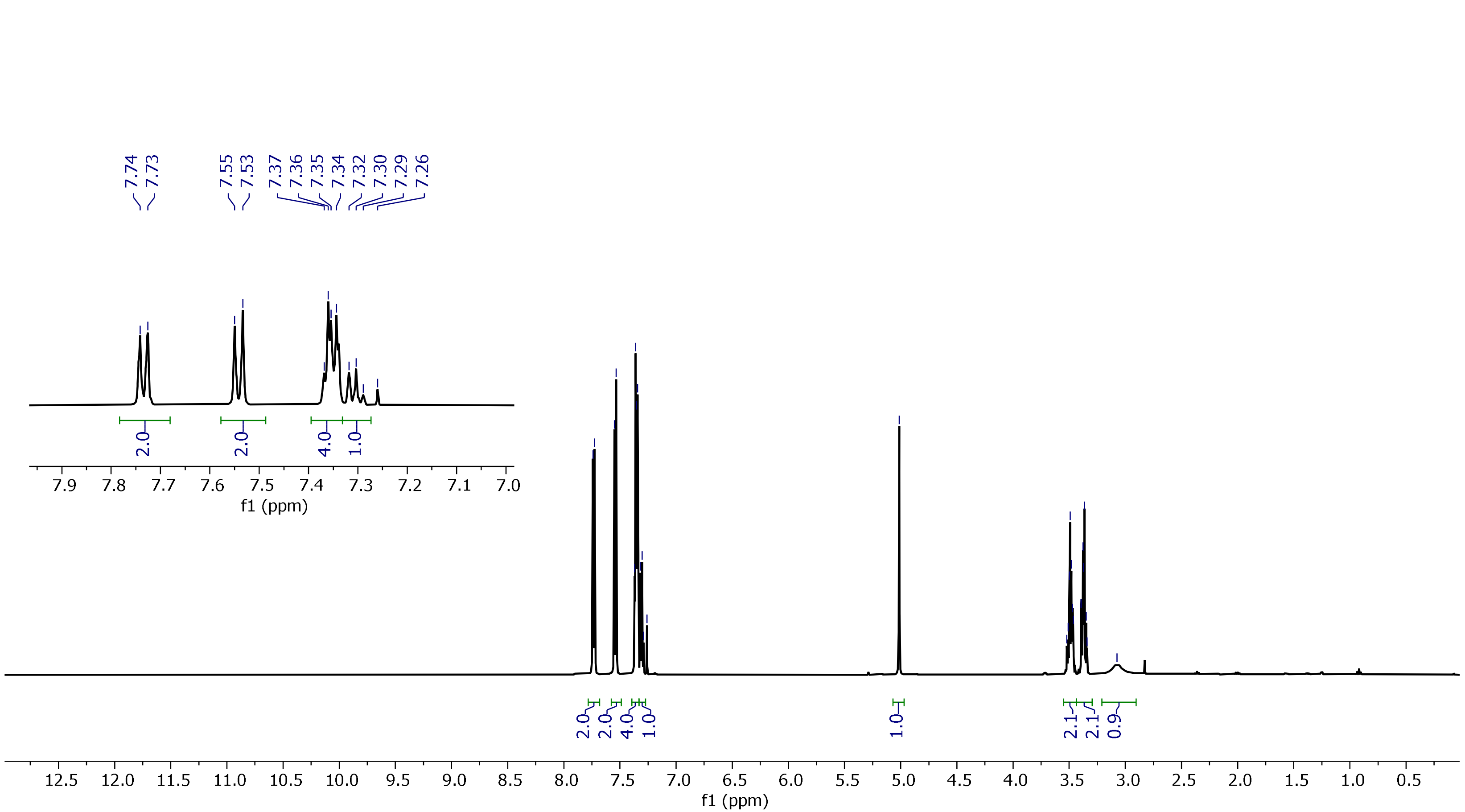

Figure S84. $^{13}C\{^{1}H\}$ NMR (126 MHz, $CDCl_3$, APT) spectrum **3c**

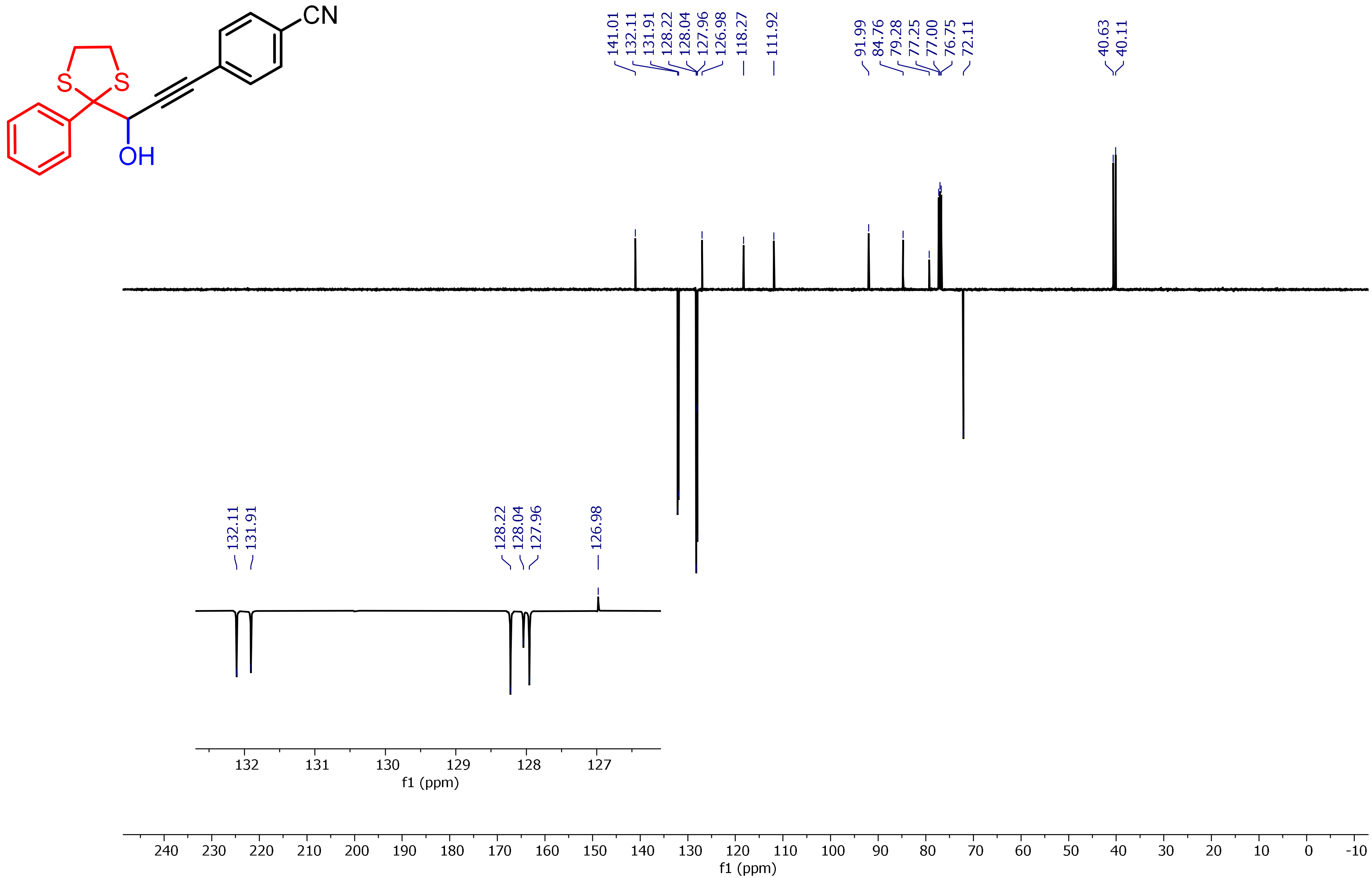

Figure S85. ^{1}H NMR ($CDCl_3$, 500 MHz) spectrum **3d**

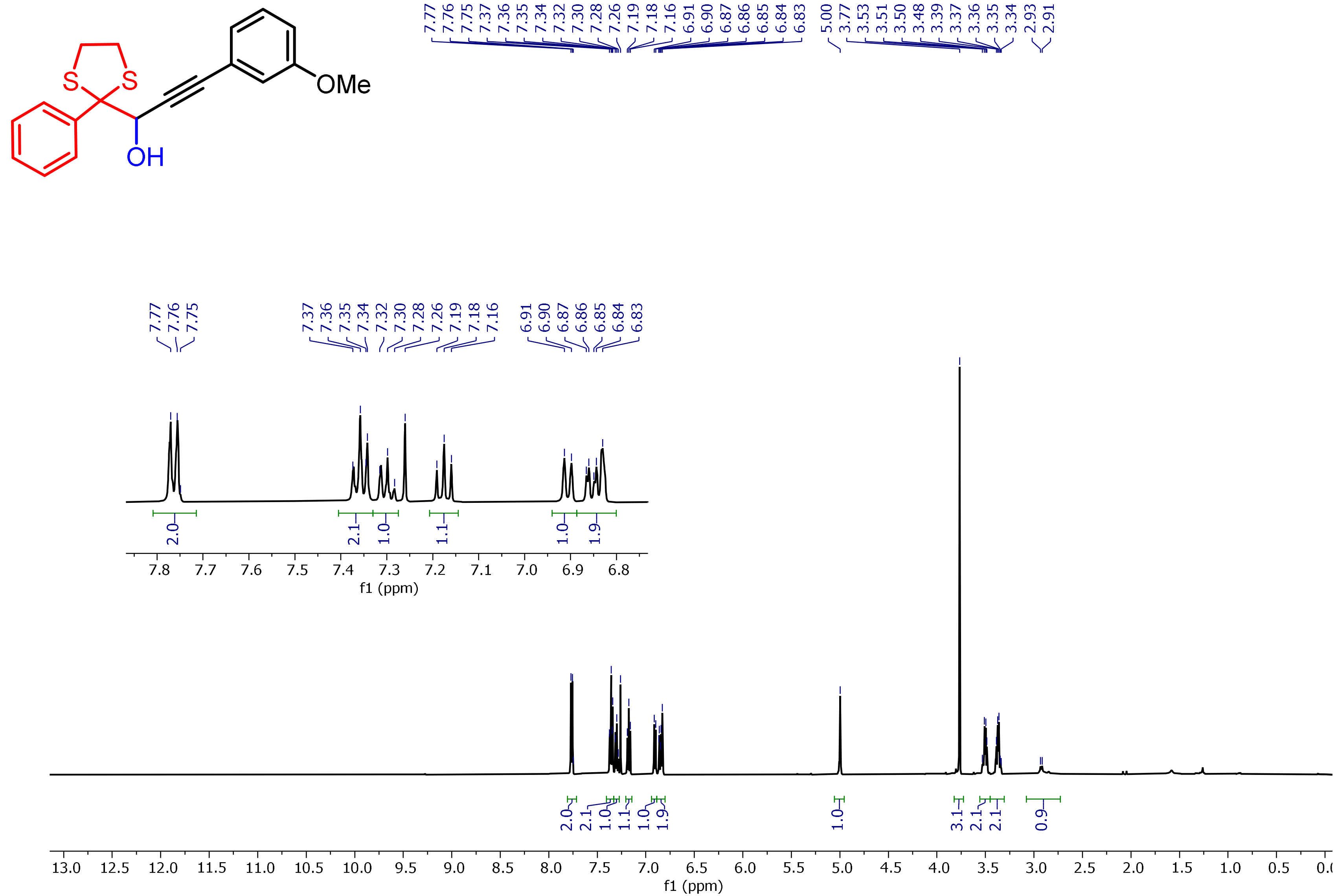

Figure S86. $^{13}C\{^{1}H\}$ NMR (126 MHz, $CDCl_3$, APT) spectrum **3d**

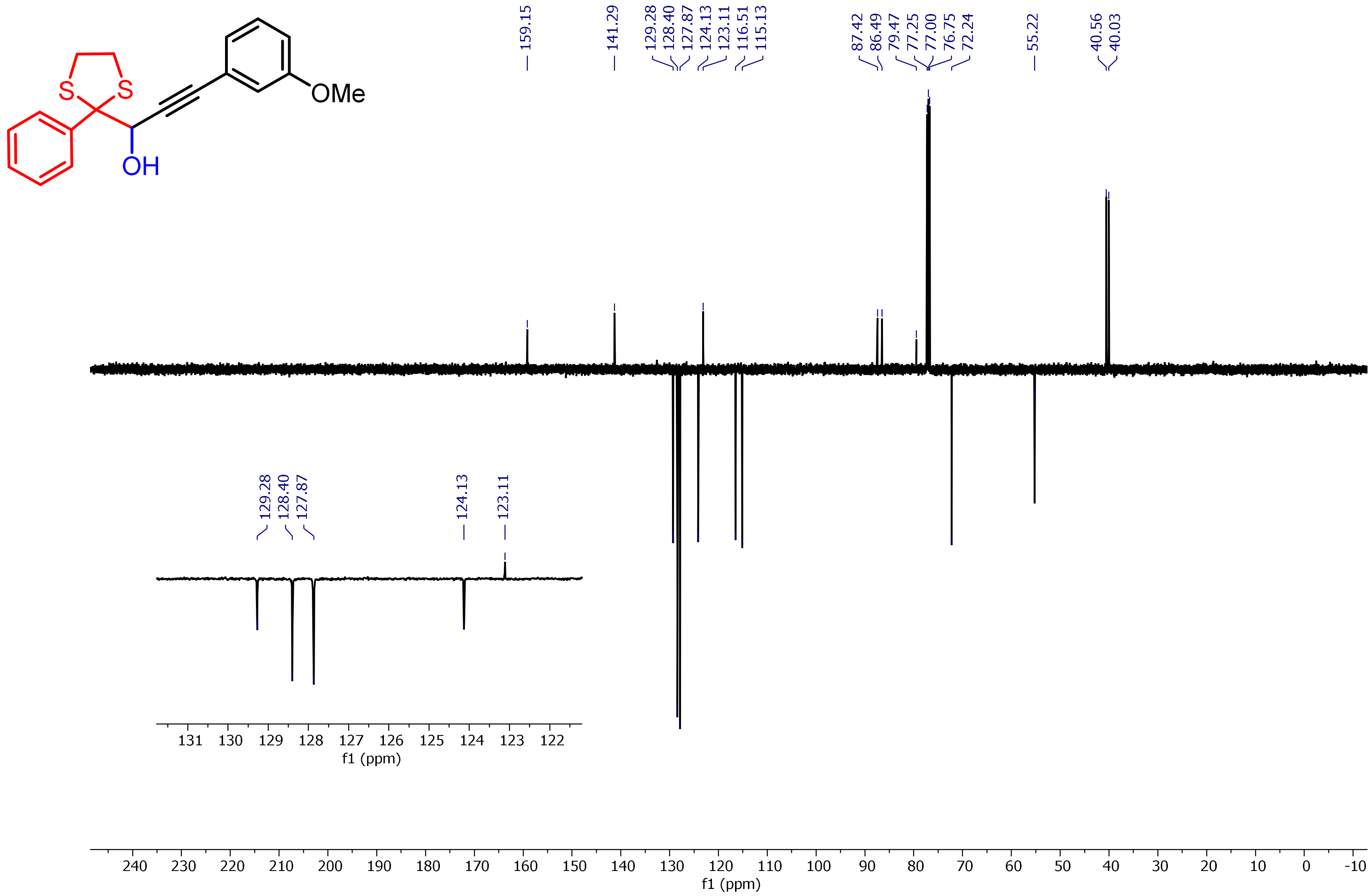

Figure S87. ^{1}H NMR ($CDCl_3$, 500 MHz) spectrum **3e**

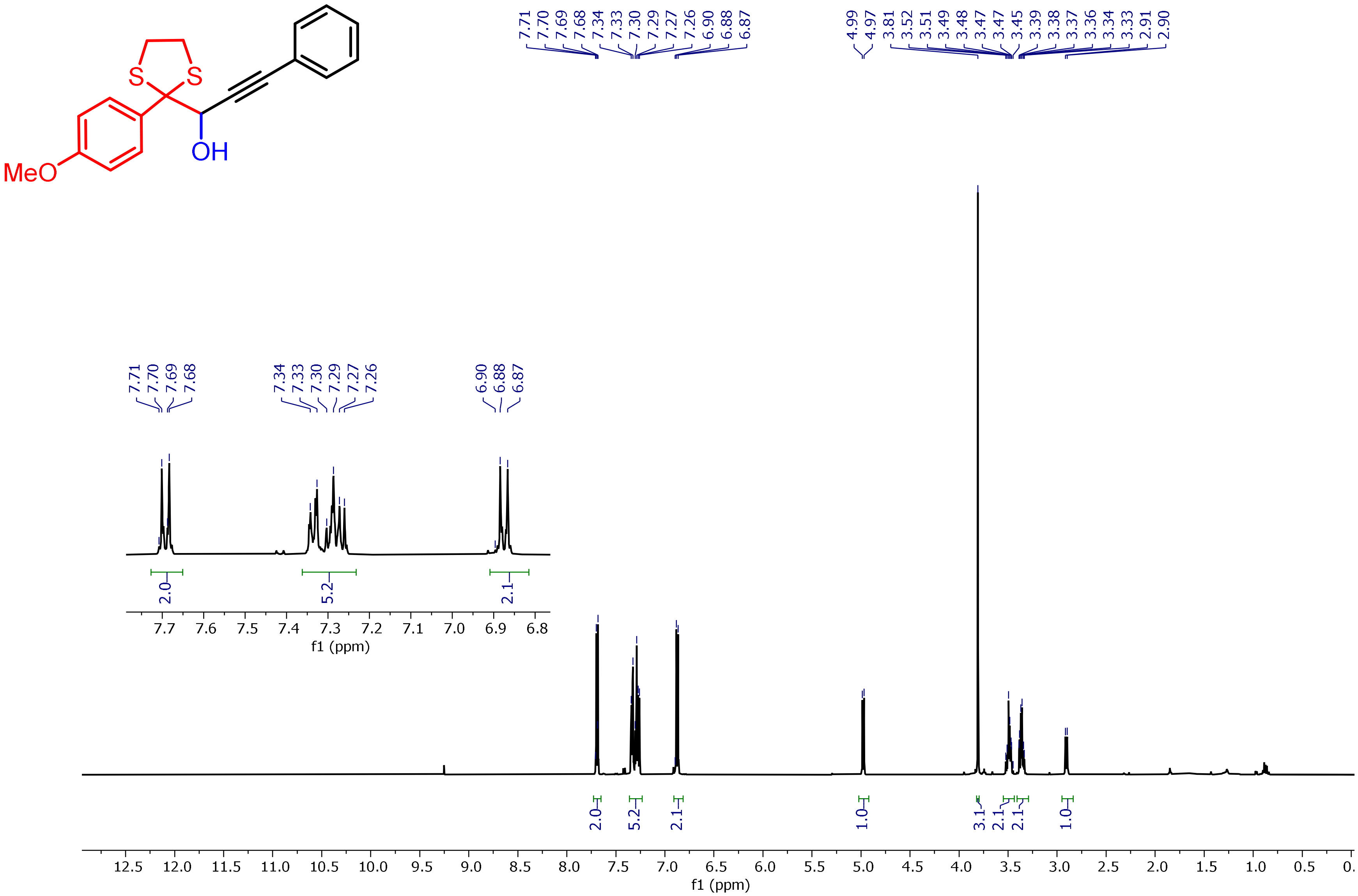

Figure S88. $^{13}C\{^1H\}$ NMR (126 MHz, $CDCl_3$, APT) spectrum **3e**

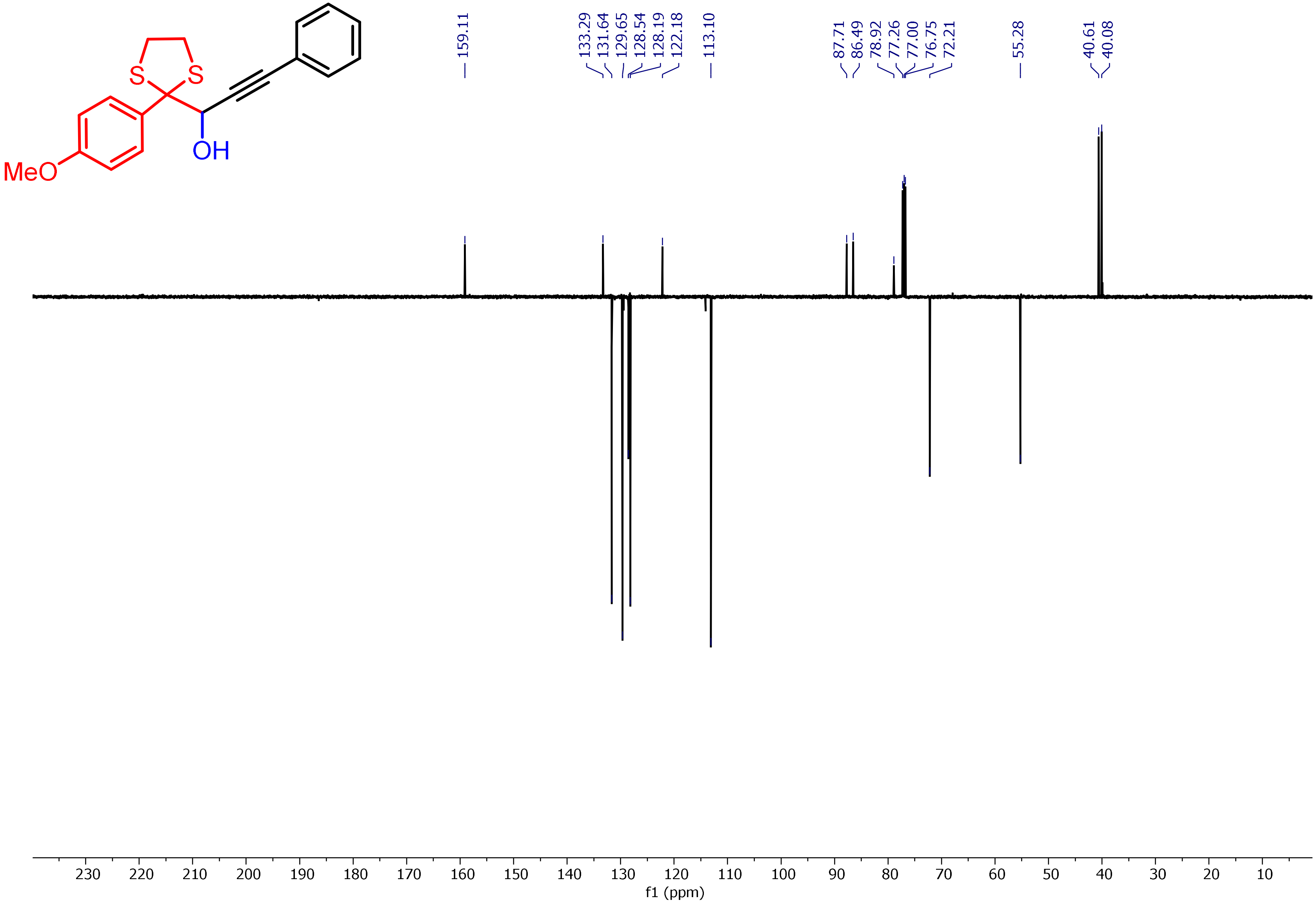

Figure S89. ^{1}H NMR ($CDCl_3$, 500 MHz) spectrum **3f**

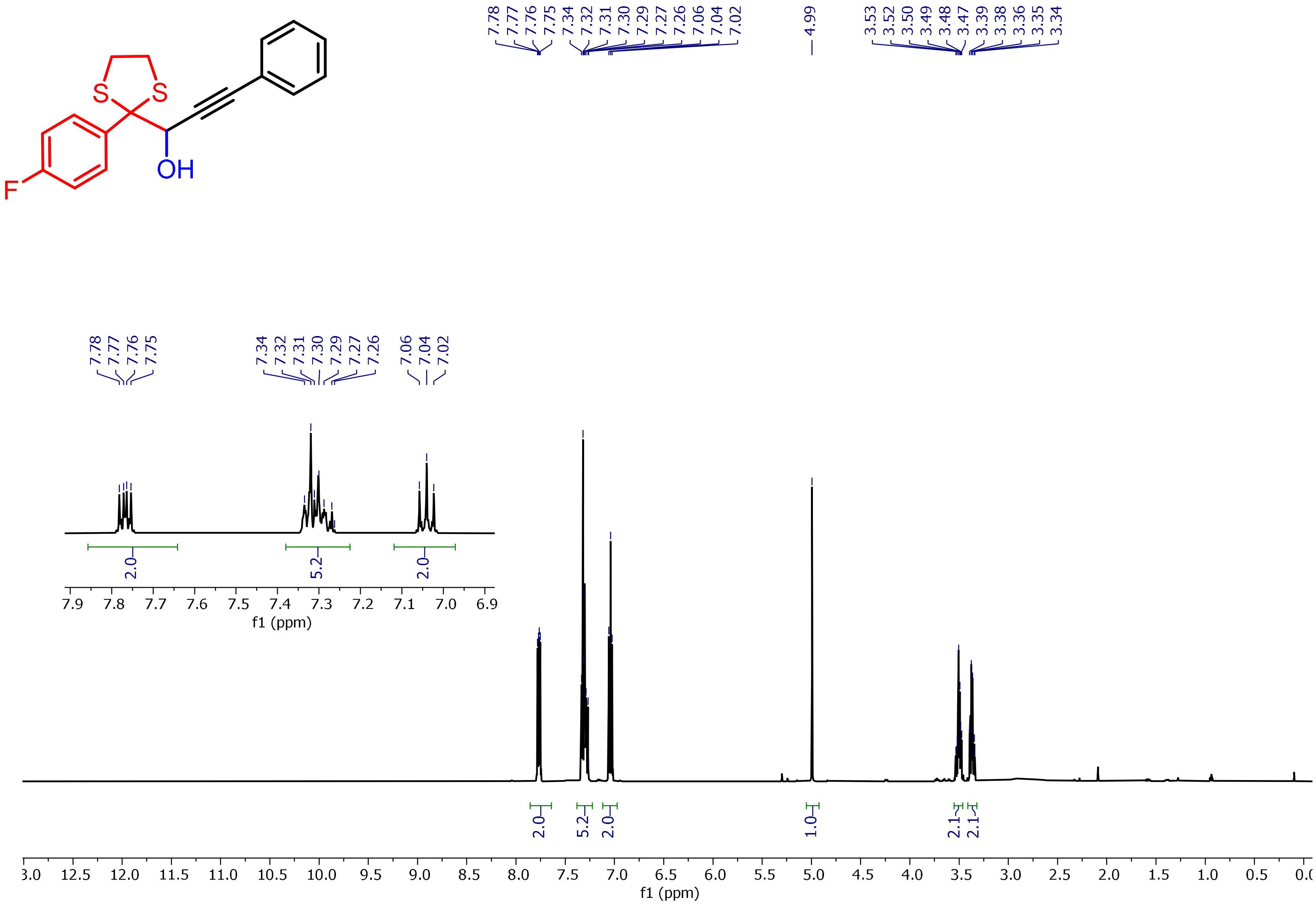

Figure S90. $^{13}C\{^1H\}$ NMR (126 MHz, $CDCl_3$, APT) spectrum **3f**

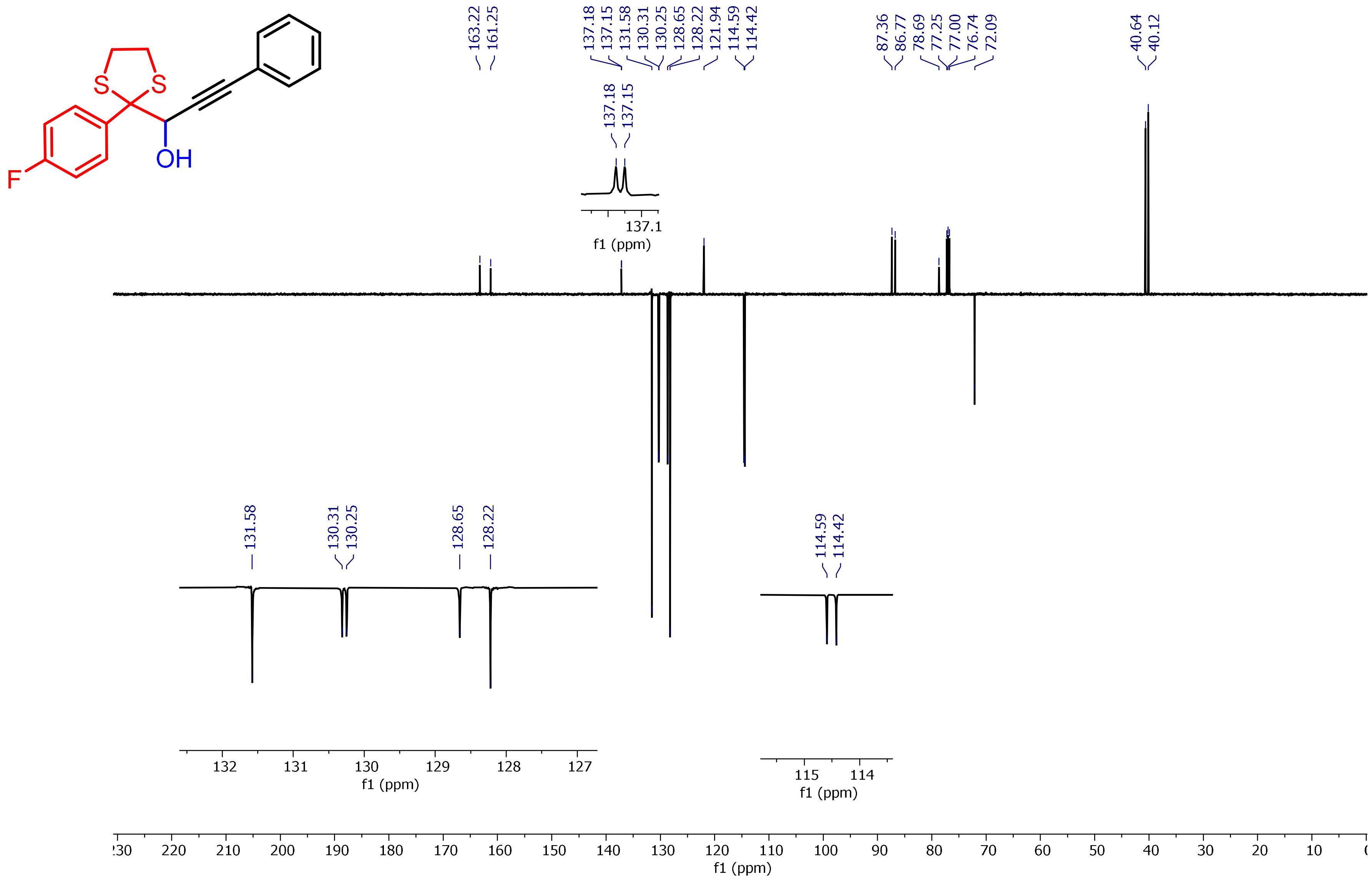

Figure S91. ^{1}H NMR ($CDCl_3$, 500 MHz) spectrum **5a**

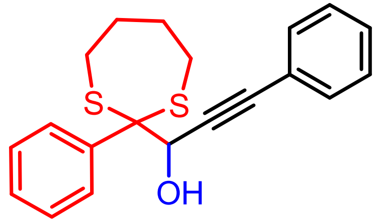

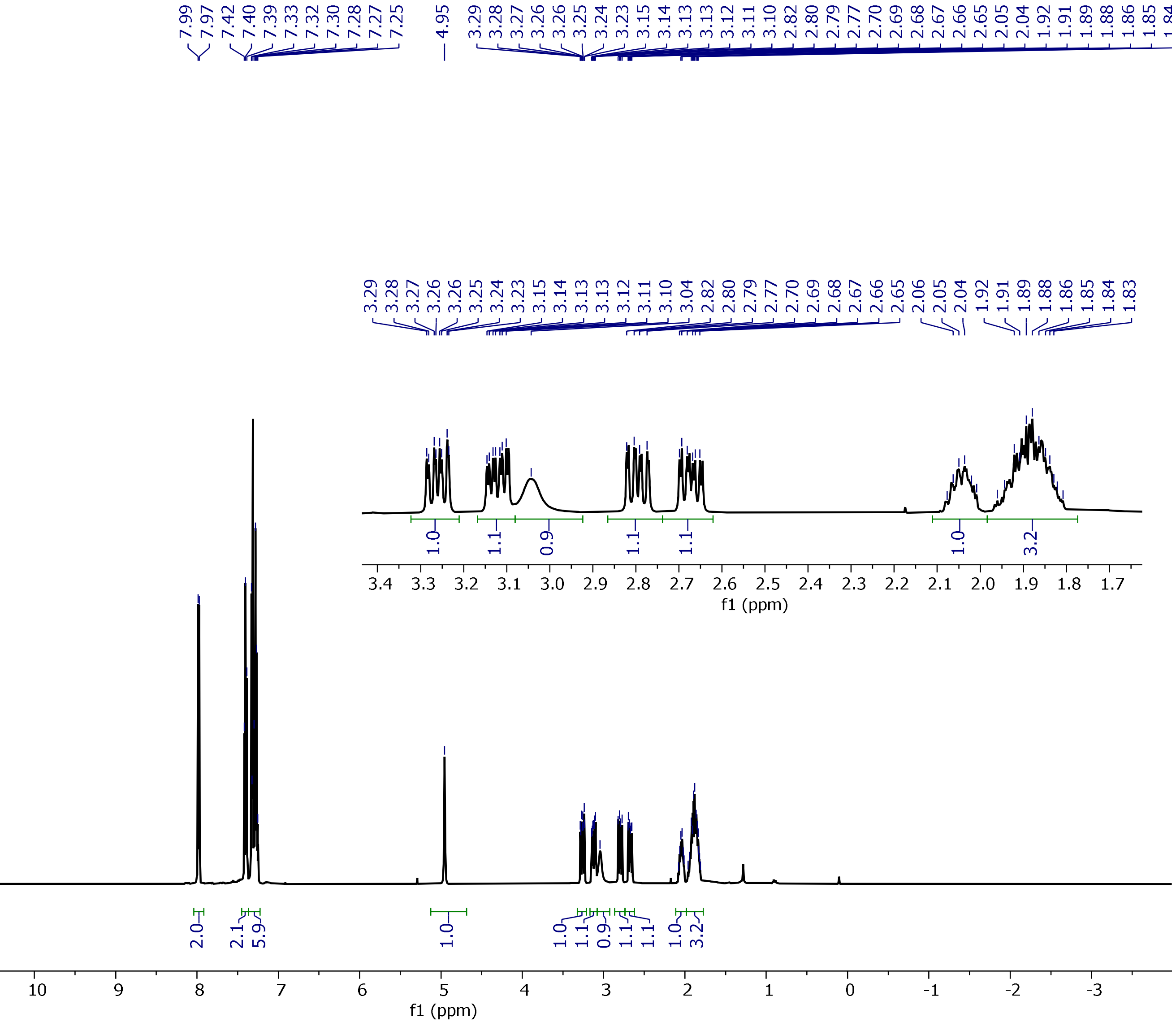

Figure S92. $^{13}C\{^{1}H\}$ NMR (126 MHz, $CDCl_3$, APT) spectrum **5a**

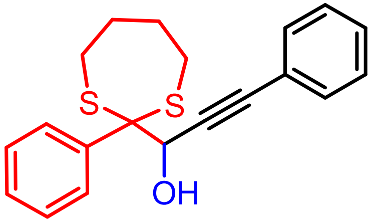

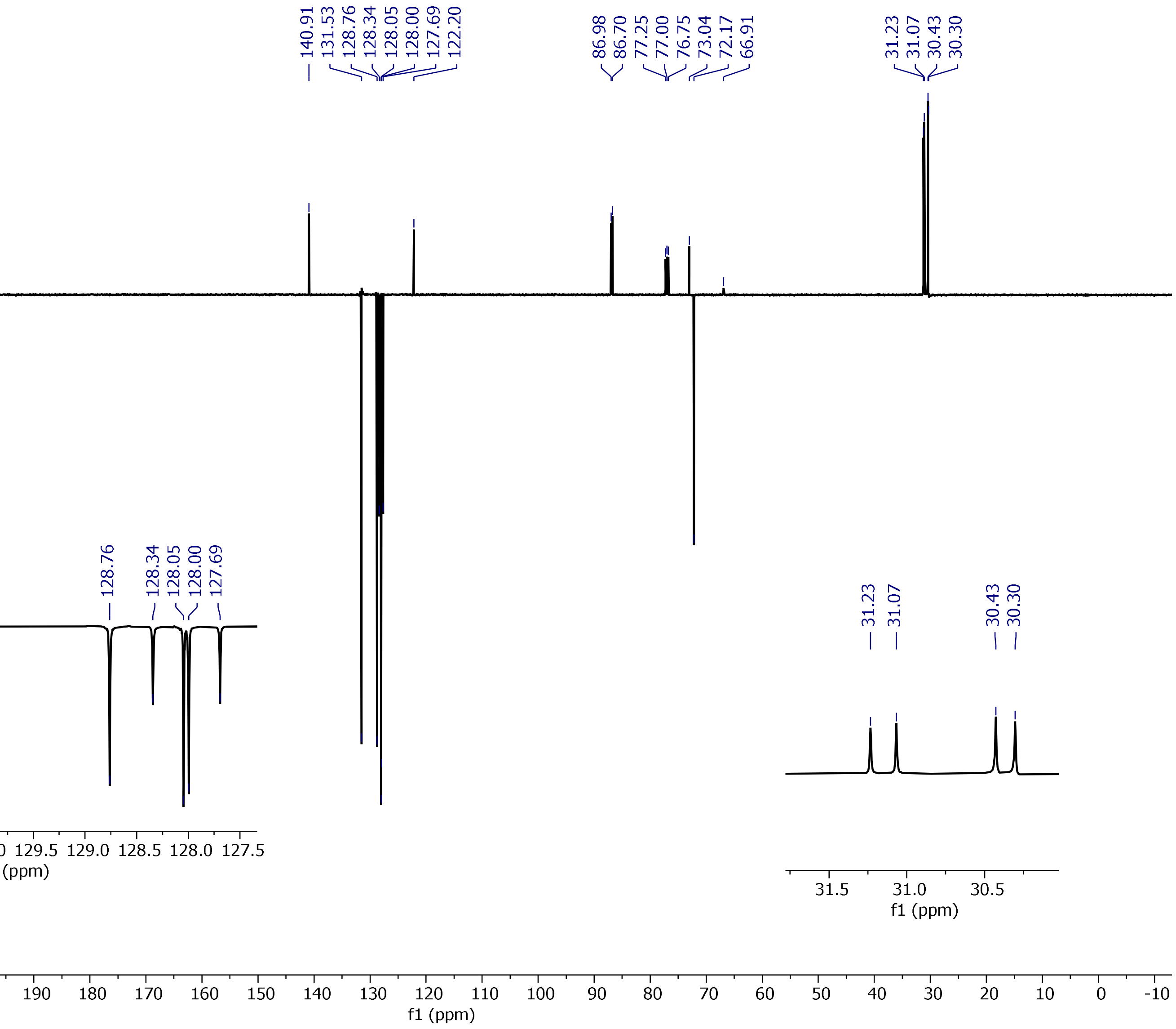

Figure S93. ^{1}H NMR ($CDCl_3$, 500 MHz) spectrum **2a**-(***E***)

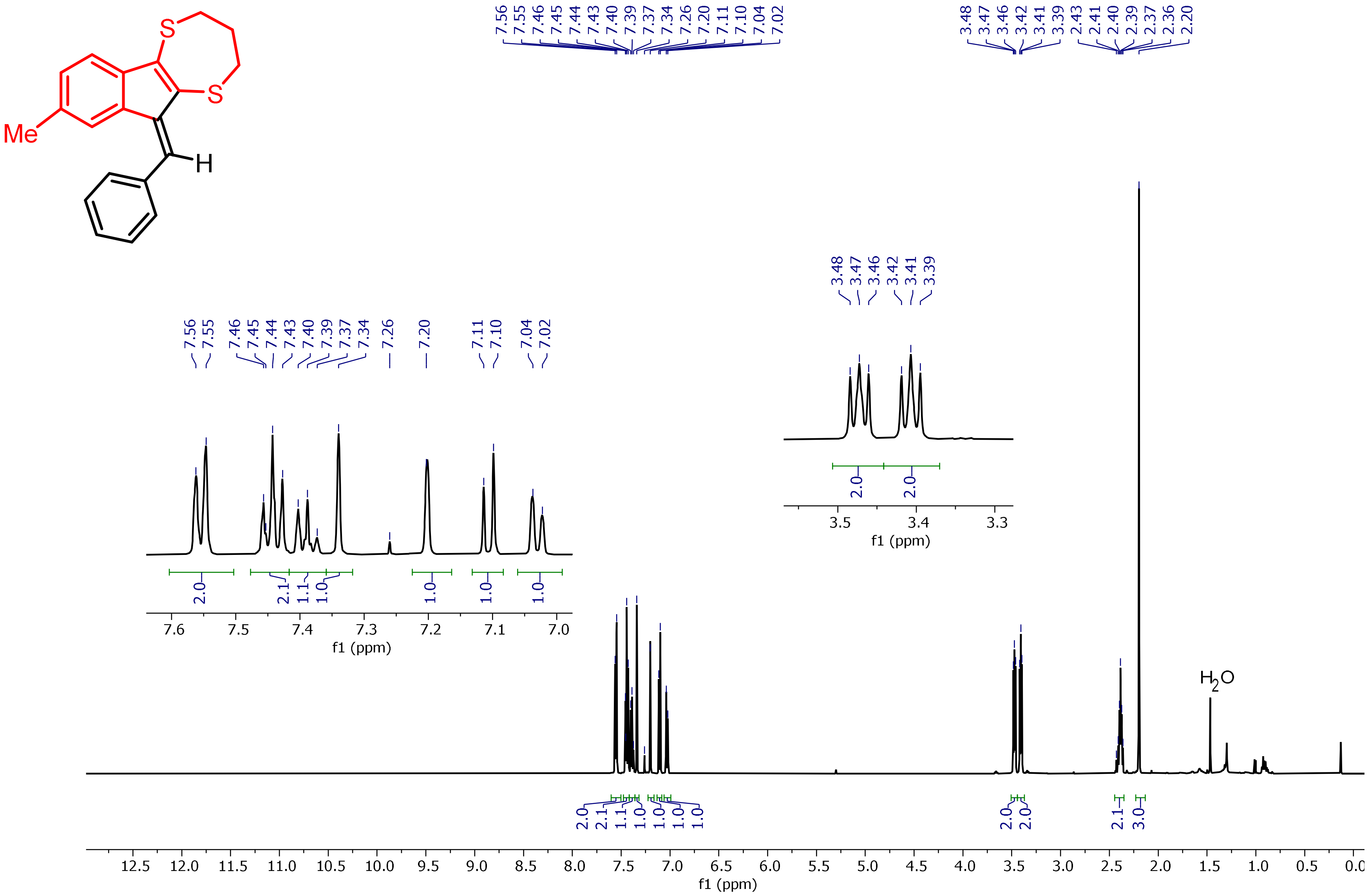

Figure S94. $^{13}C\{^1H\}$ NMR (126 MHz, $CDCl_3$, APT) spectrum **2a-(*E*)**

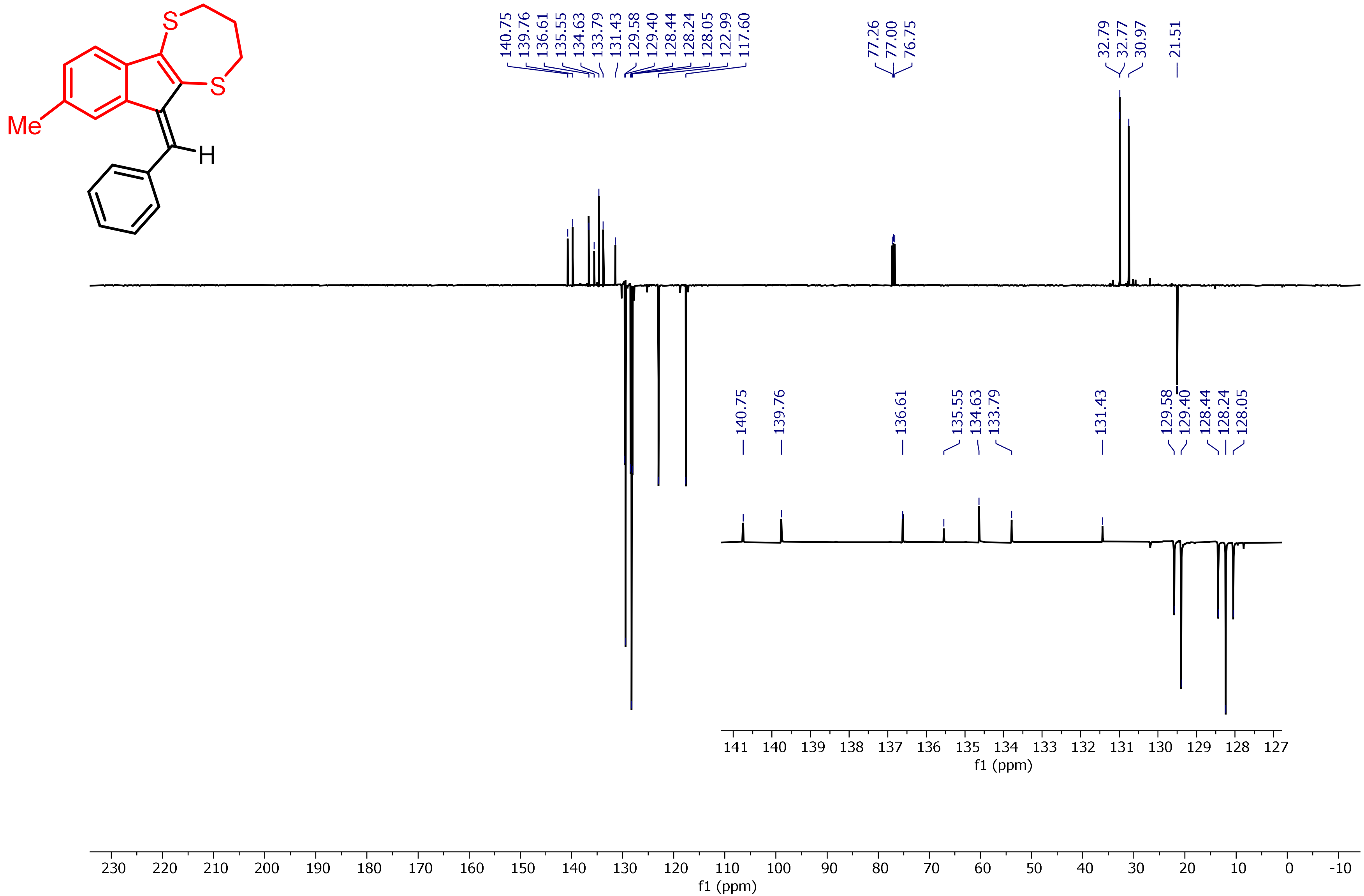

Figure S95. ^{1}H NMR ($CDCl_3$, 500 MHz) spectrum **2a**-(***Z***)

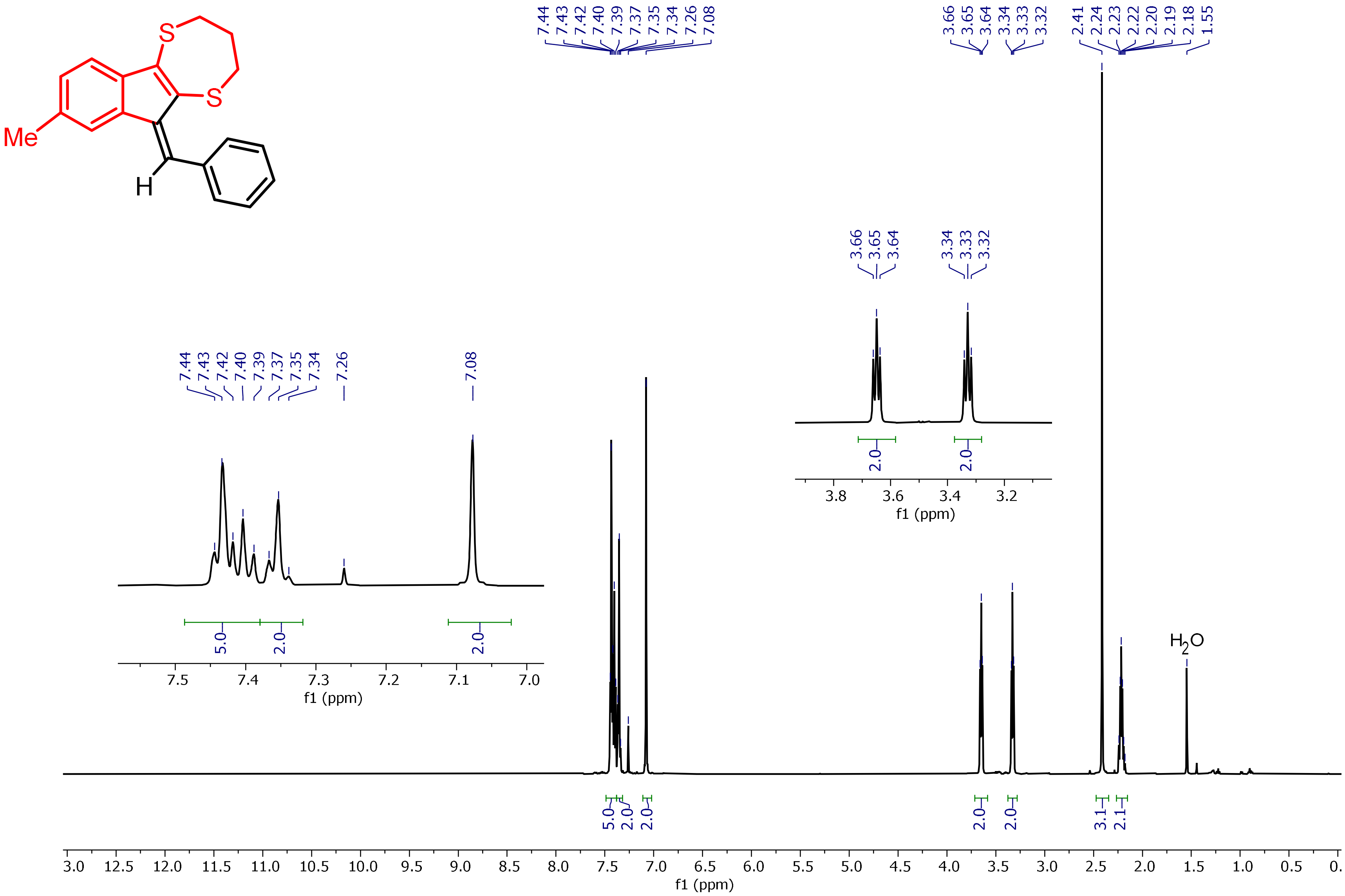

Figure S96. $^{13}C\{^1H\}$ NMR (126 MHz, $CDCl_3$, APT) spectrum **2a-(*Z*)**

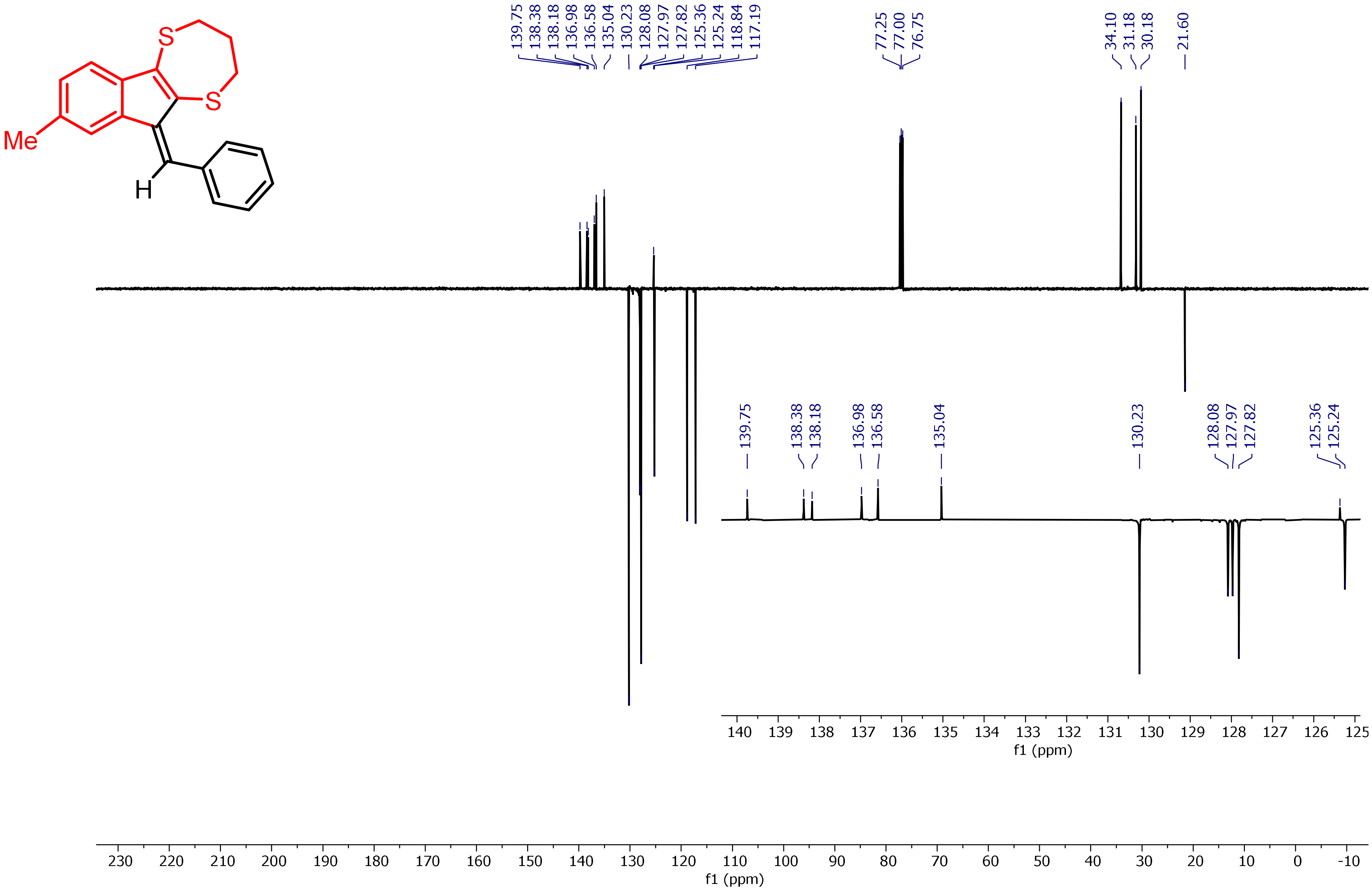

Figure S97. ^{1}H NMR ($CDCl_3$, 500 MHz) spectrum **2b**-(***E***)

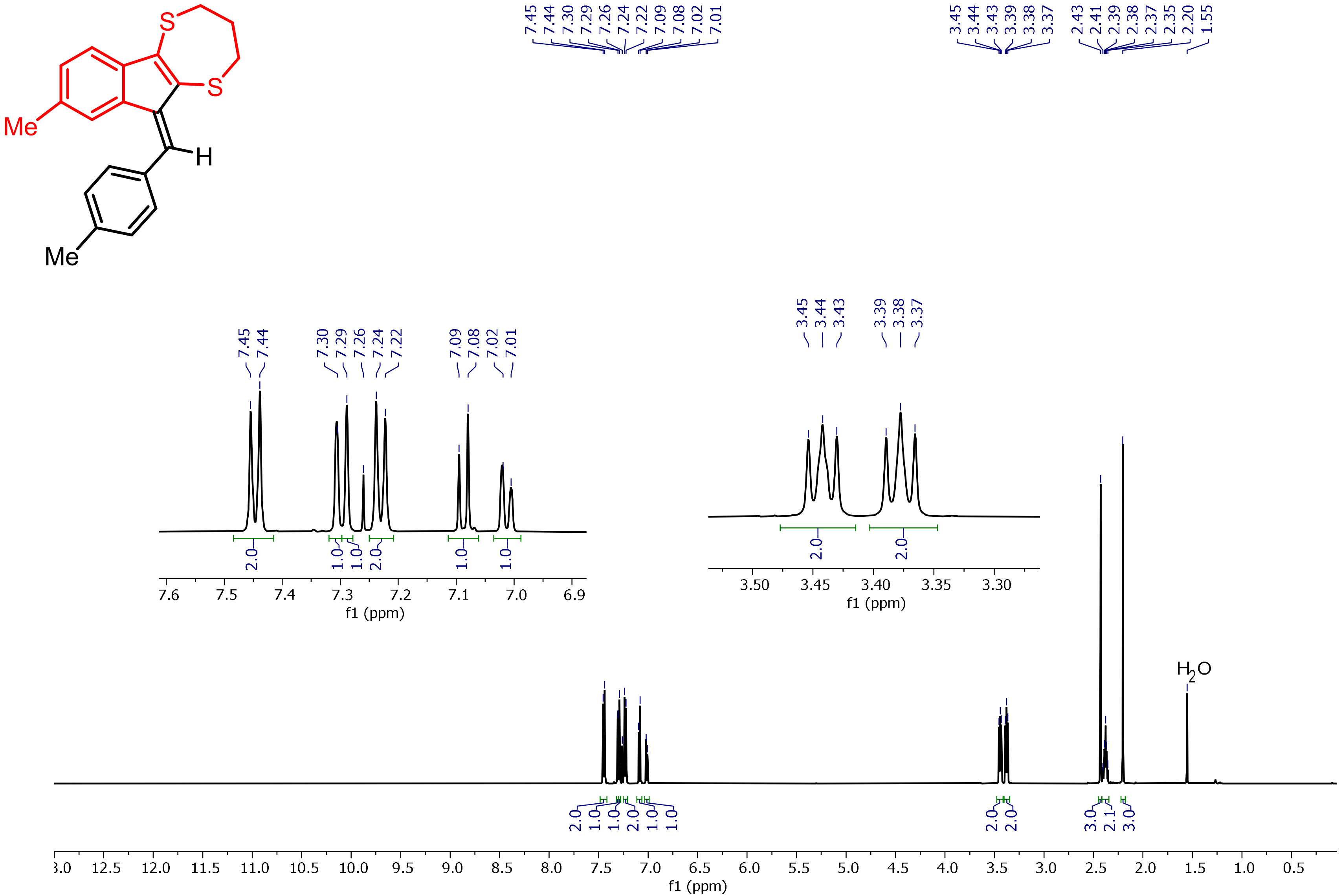

Figure S98. $^{13}C\{^{1}H\}$ NMR (126 MHz, $CDCl_3$, APT) spectrum **2b-(*E*)**

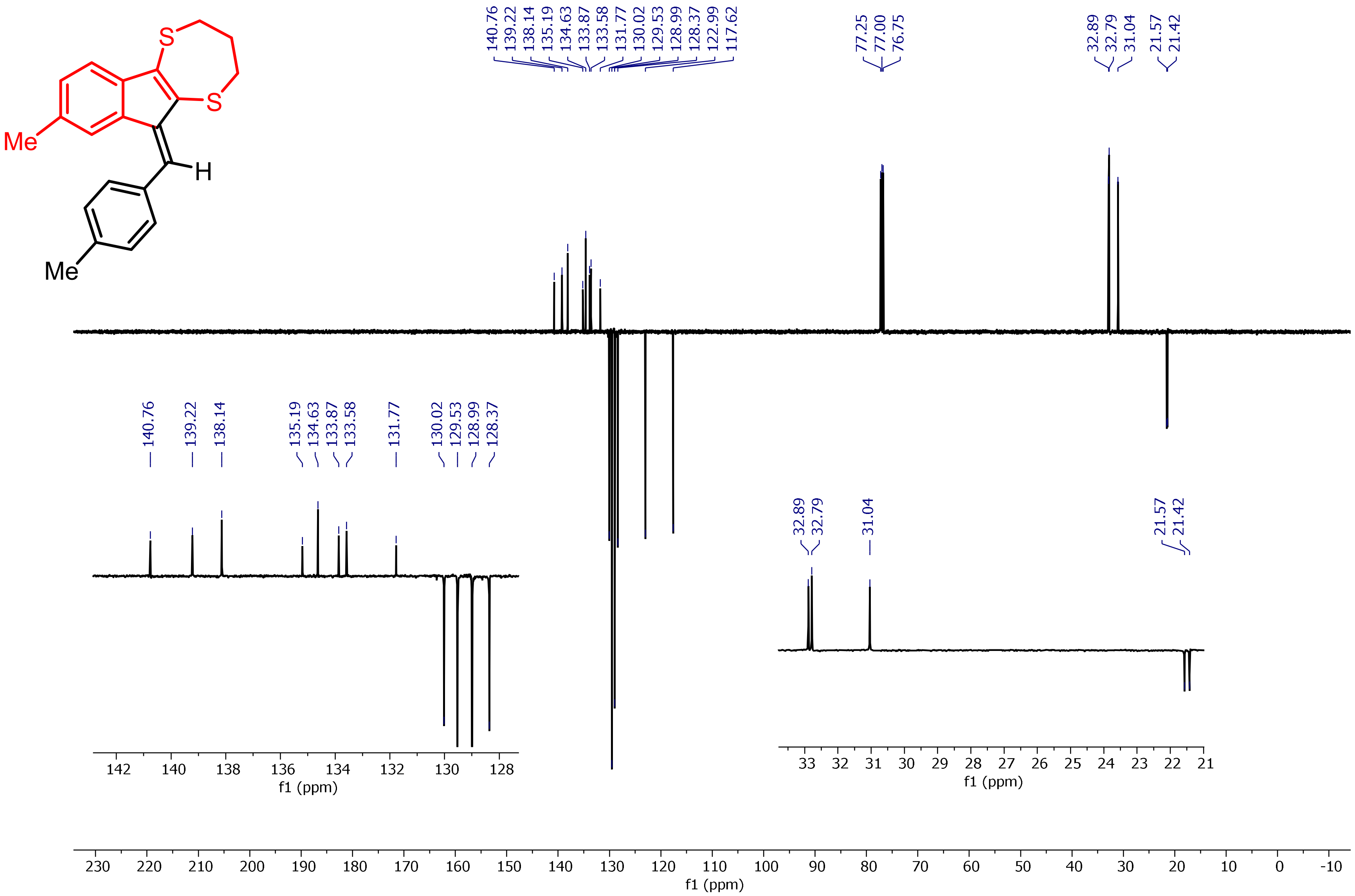

Figure S99. ^{1}H NMR ($CDCl_3$, 500 MHz) spectrum **2b**-(***Z***)

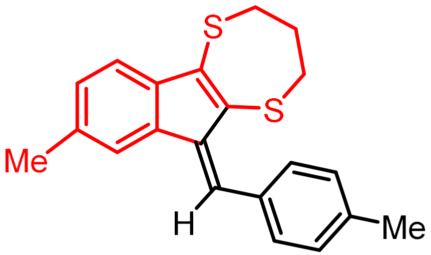

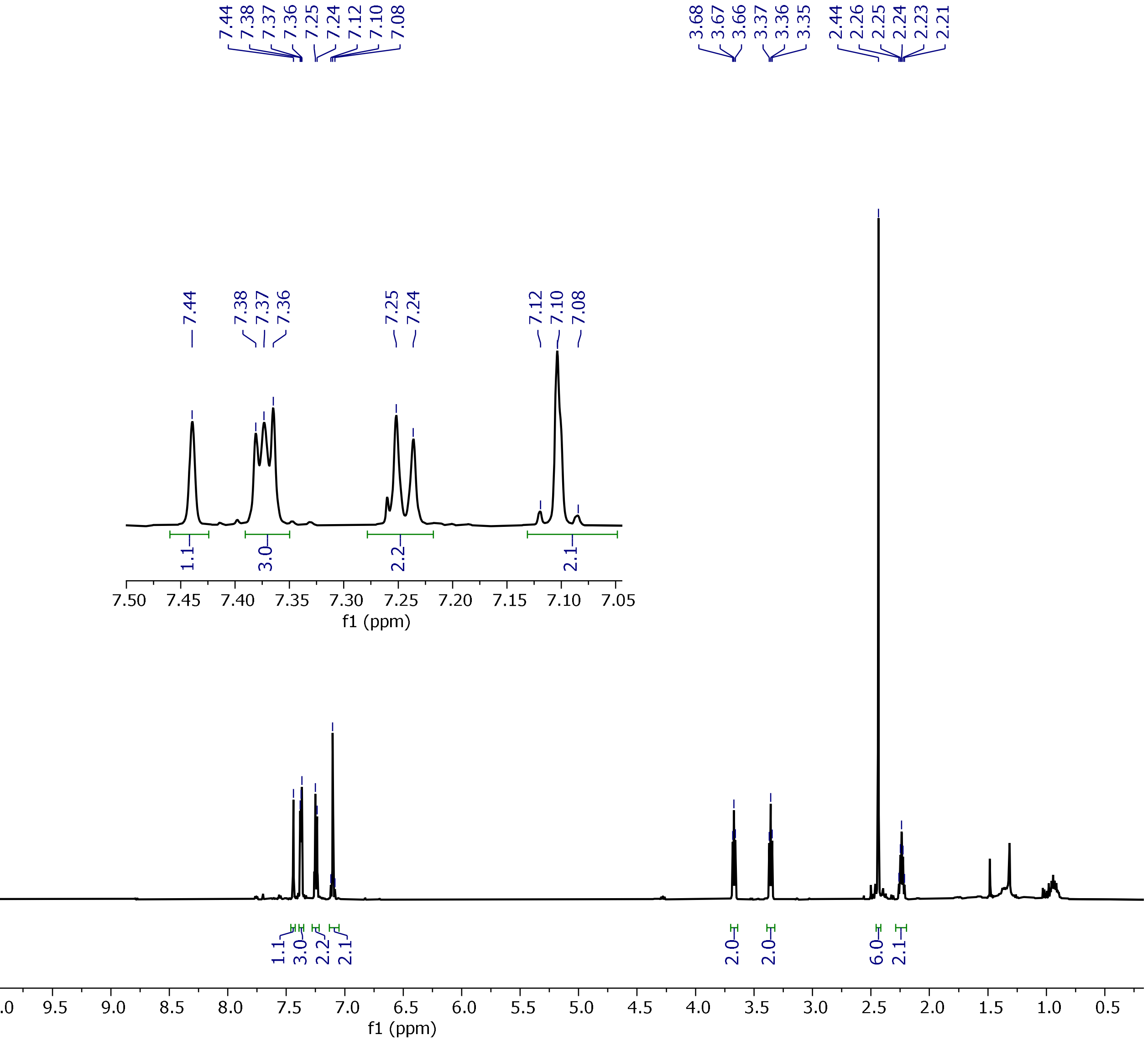

Figure S100. $^{13}C\{^{1}H\}$ NMR (126 MHz, $CDCl_3$, APT) spectrum **2b-(*Z*)**

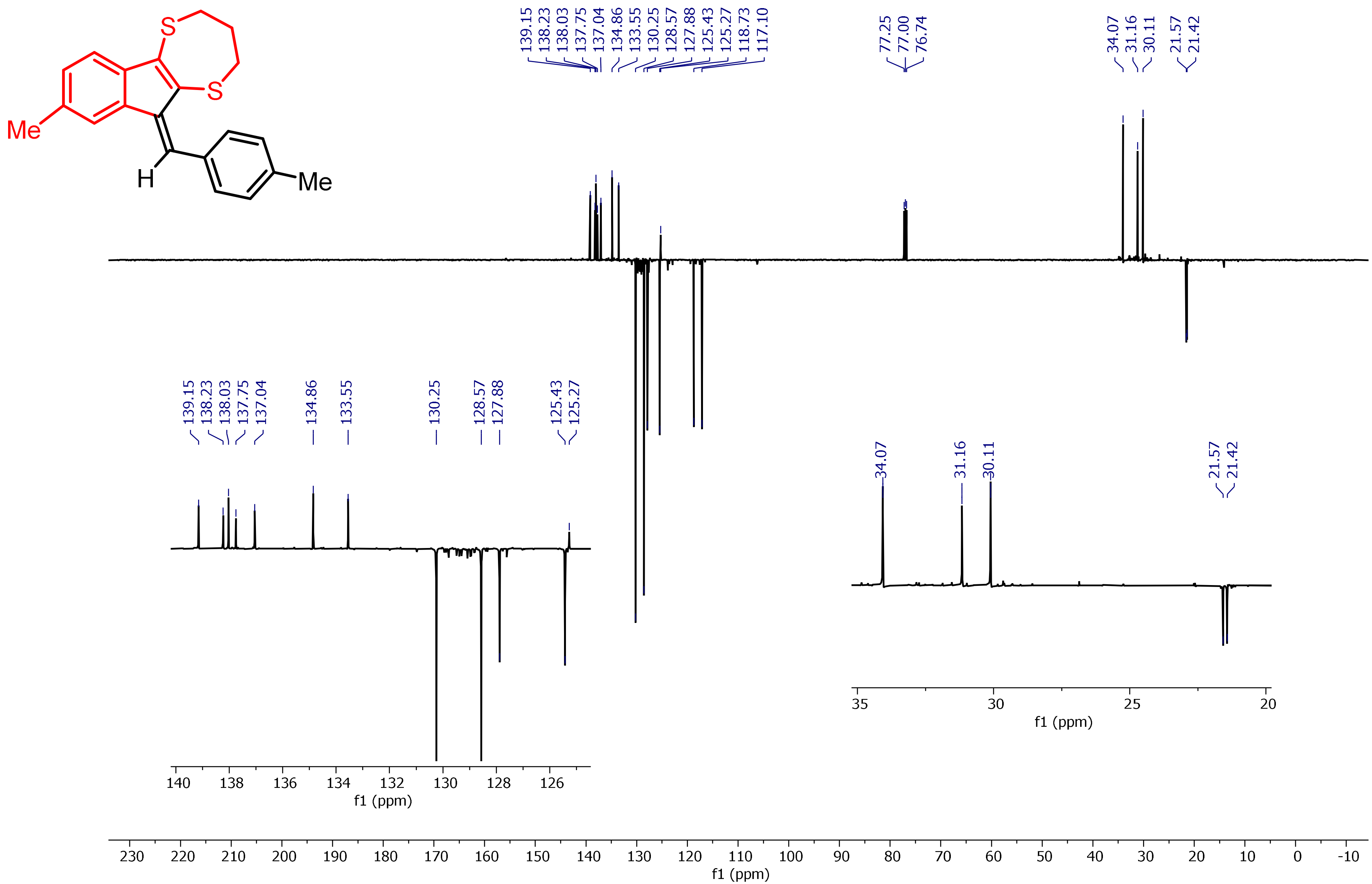

Figure S101. ^{1}H NMR ($CDCl_3$, 500 MHz) spectrum **2c**-(***E***)

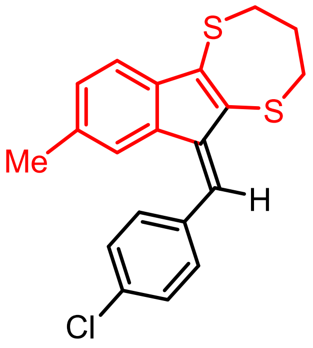

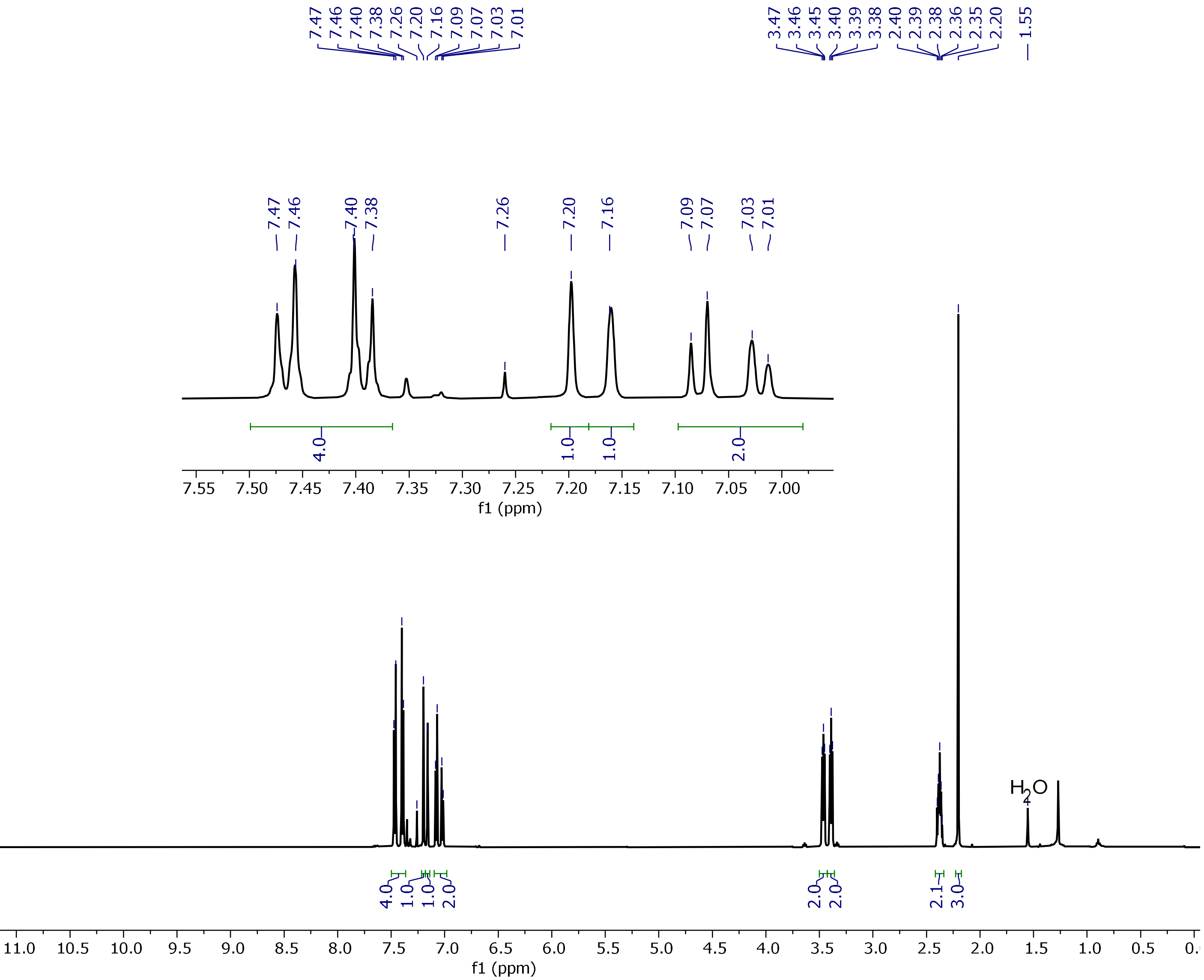

Figure S102. $^{13}C\{^{1}H\}$ NMR (126 MHz, $CDCl_3$, APT) spectrum **2c-(*E*)**

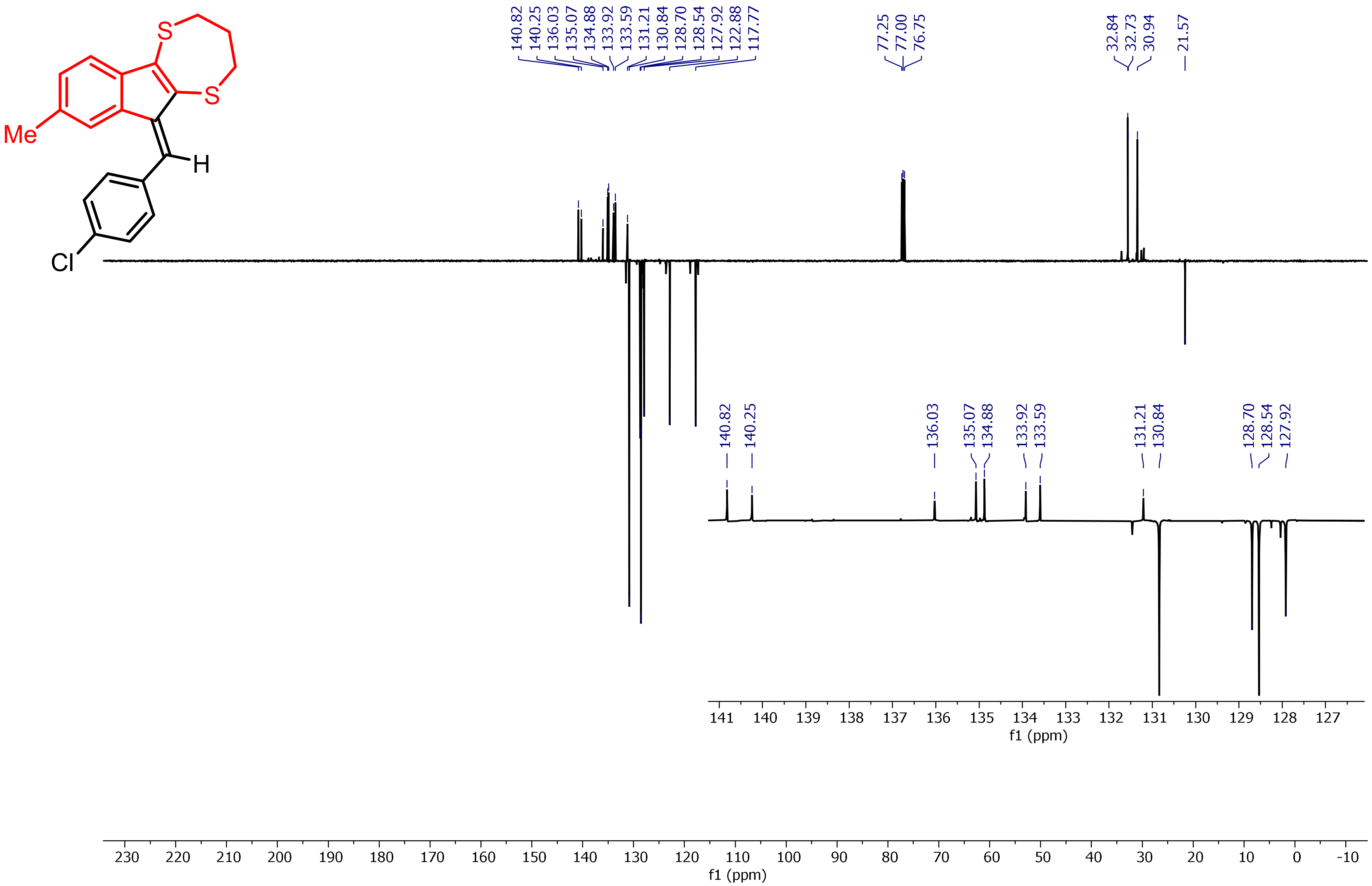

Figure S103. ^{1}H NMR ($CDCl_3$, 500 MHz) spectrum **2c**-(***Z***)

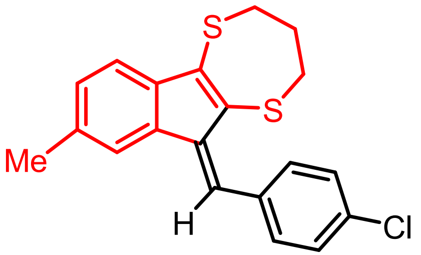

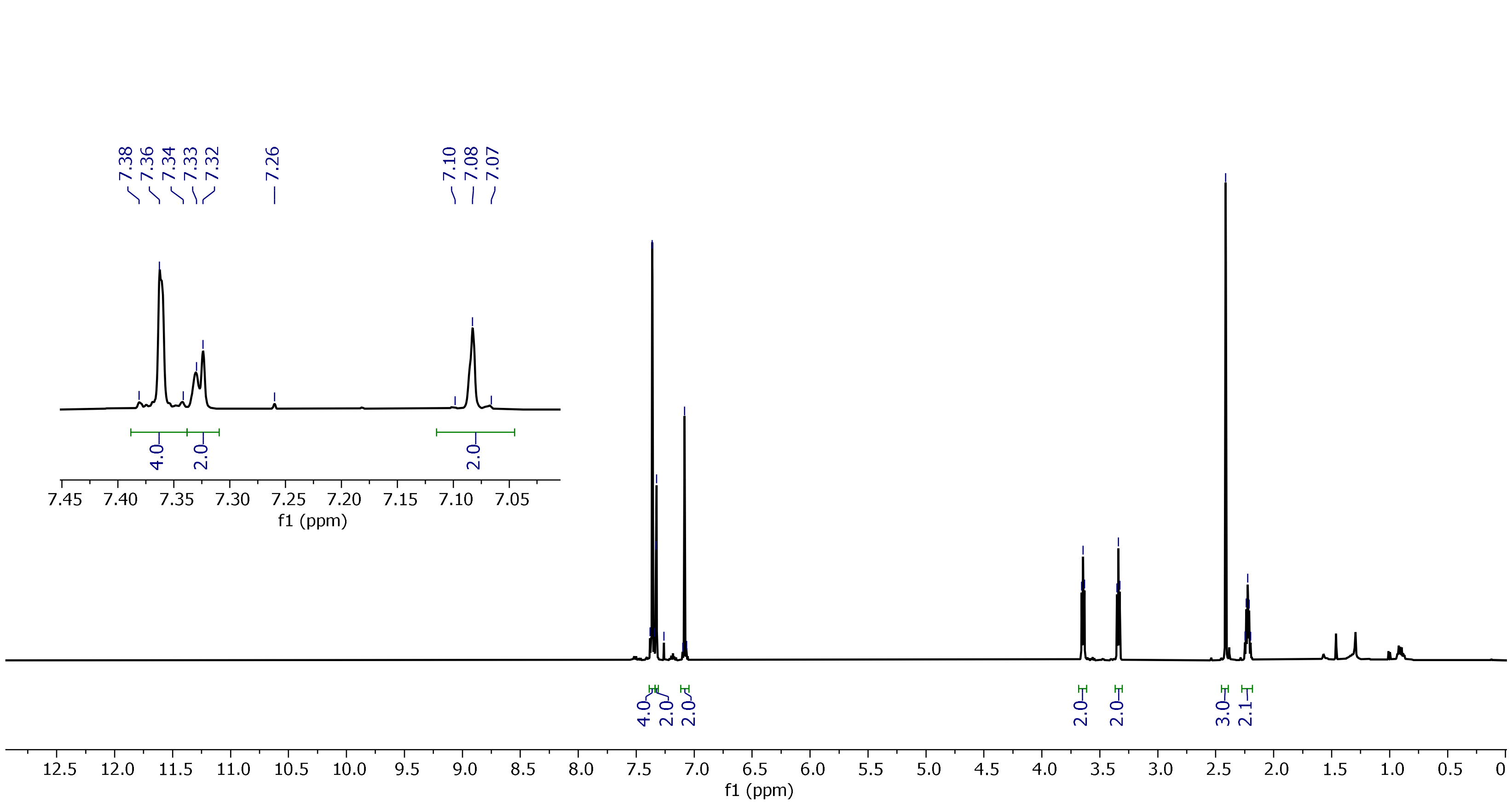

Figure S104. $^{13}C\{^1H\}$ NMR (126 MHz, $CDCl_3$, APT) spectrum **2c-(*Z*)**

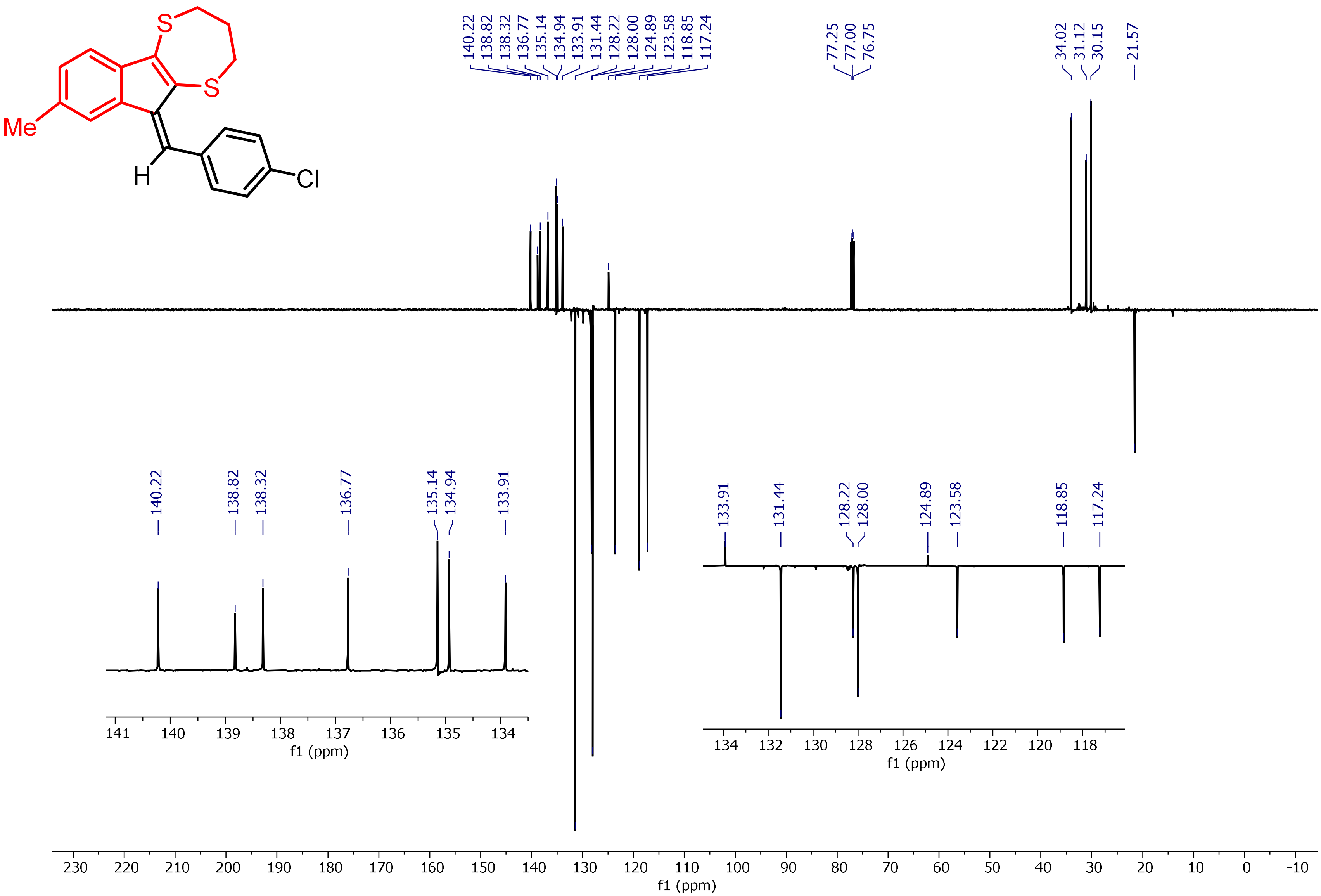

Figure S105. ^{1}H NMR ($CDCl_3$, 500 MHz) spectrum **2d**-(***E***)

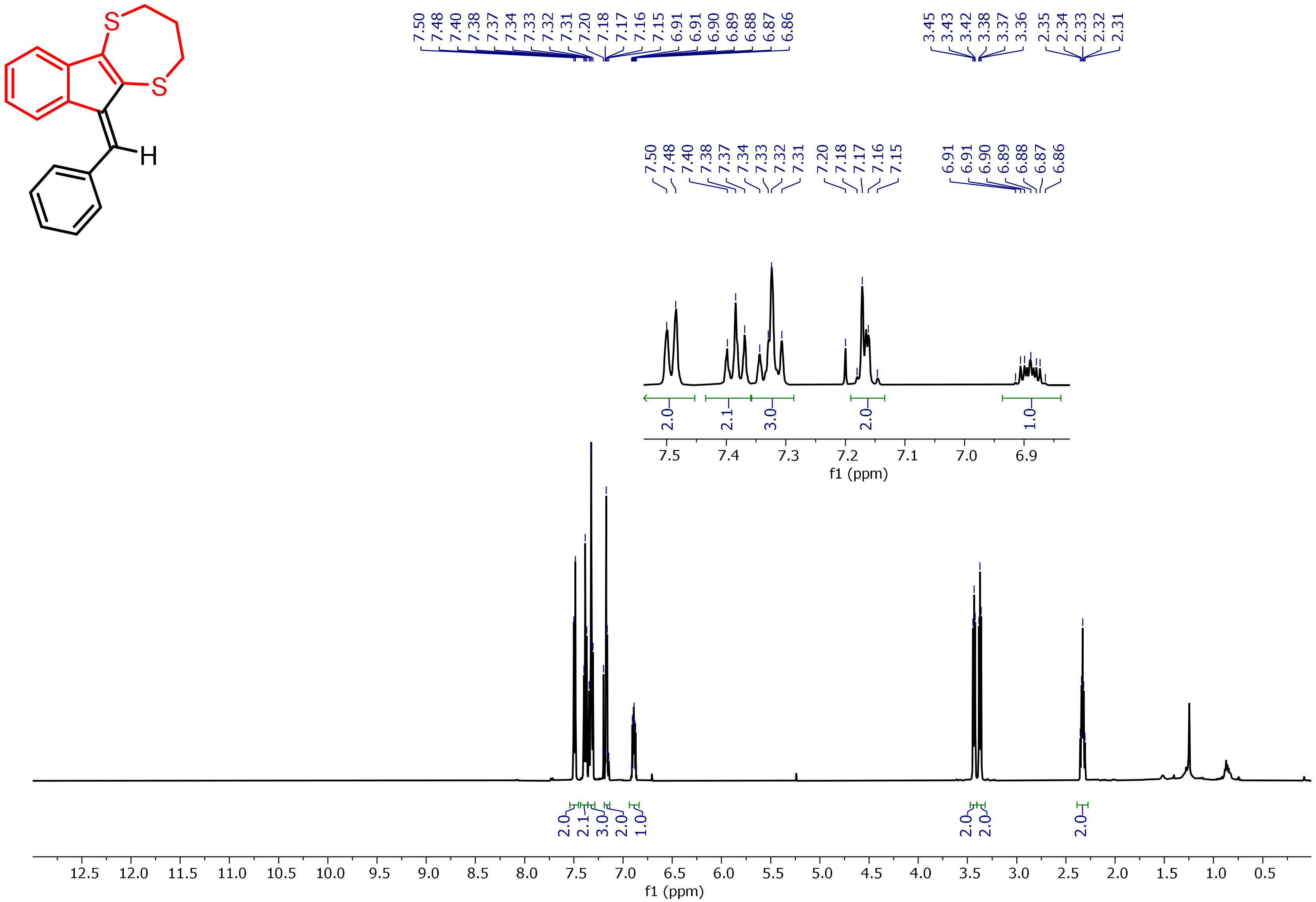

Figure S106. $^{13}C\{^{1}H\}$ NMR (126 MHz, $CDCl_3$, APT) spectrum **2d-(*E*)**

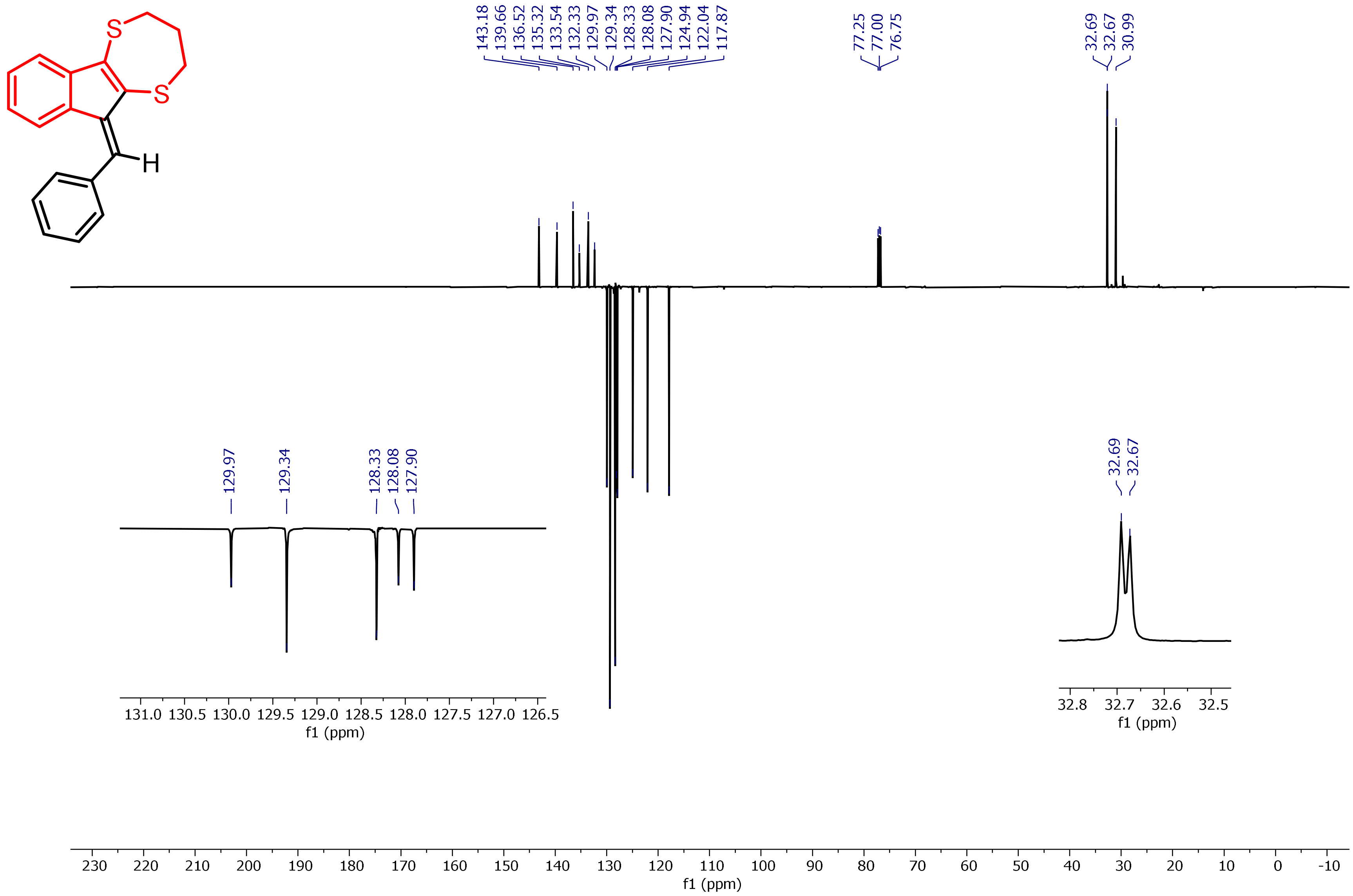

Figure S107. ^{1}H NMR ($CDCl_3$, 500 MHz) spectrum **2d**-(***Z***)

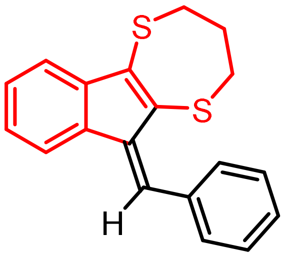

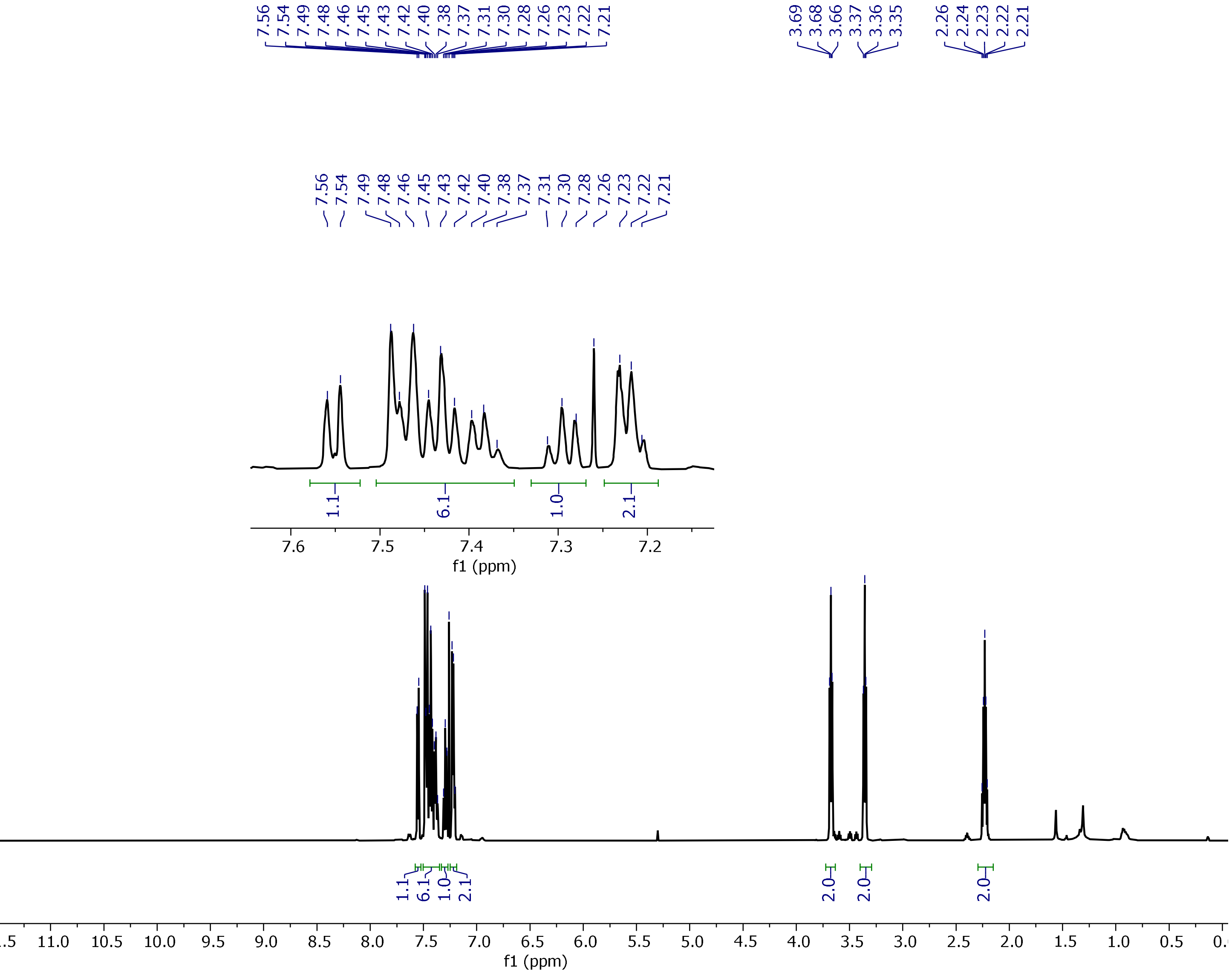

Figure S108. $^{13}C\{^{1}H\}$ NMR (126 MHz, $CDCl_3$, APT) spectrum **2d-(*Z*)**

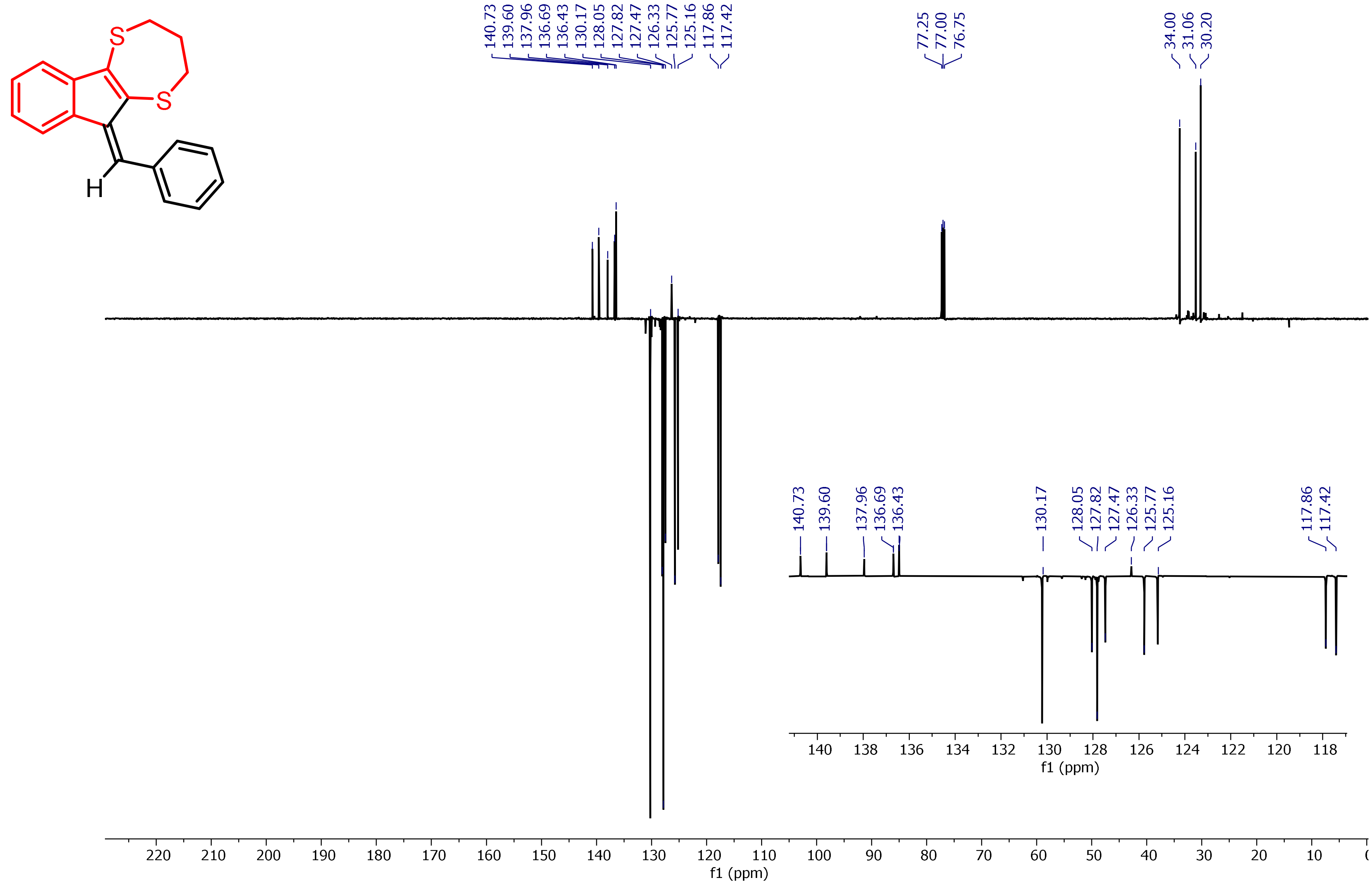

Figure S109. ^{1}H NMR ($CDCl_3$, 500 MHz) spectrum **2e**-(***E***)

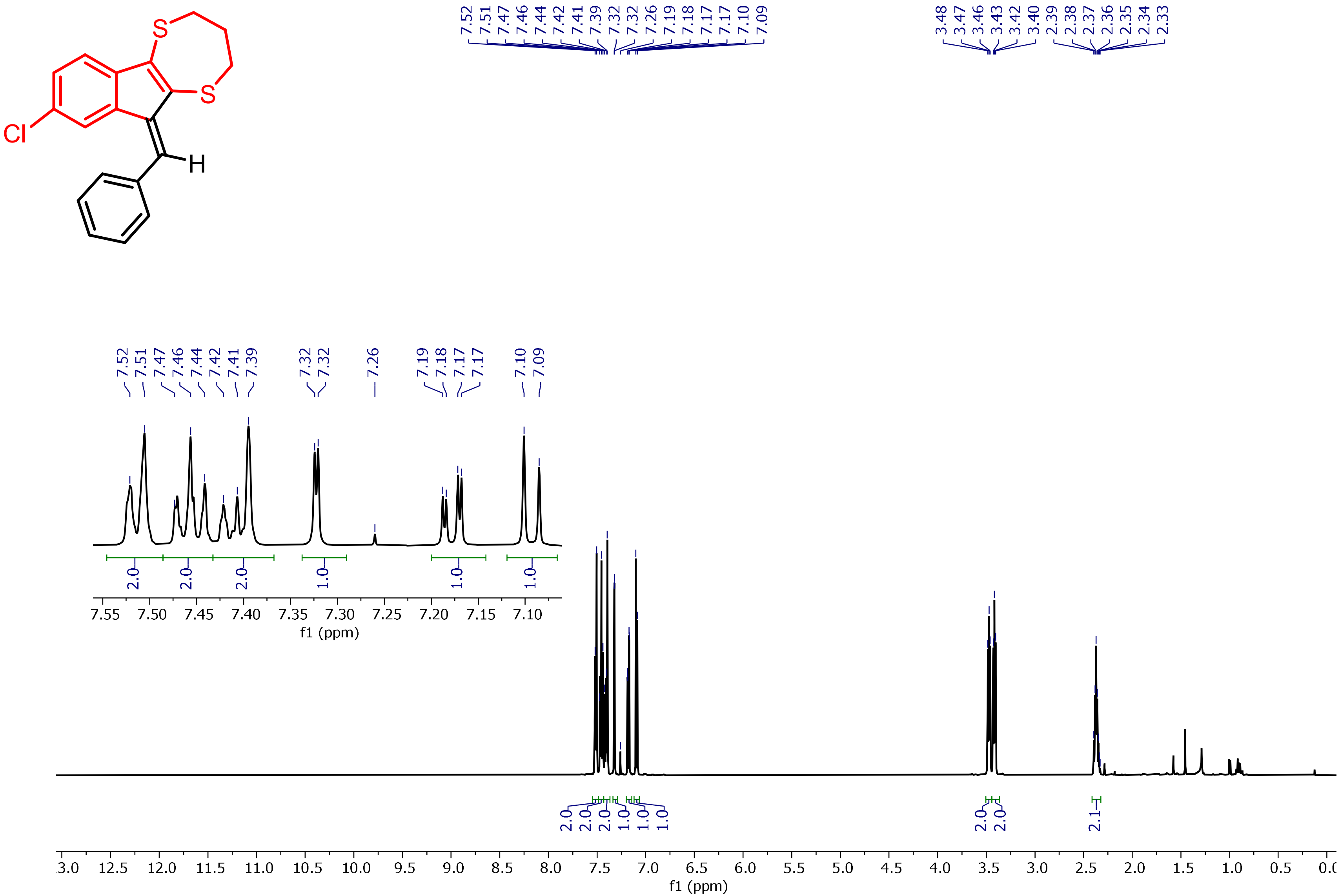

Figure S110. $^{13}C\{^1H\}$ NMR (126 MHz, $CDCl_3$, APT) spectrum **2e-(*E*)**

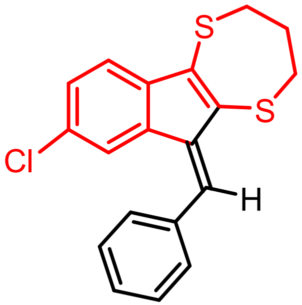

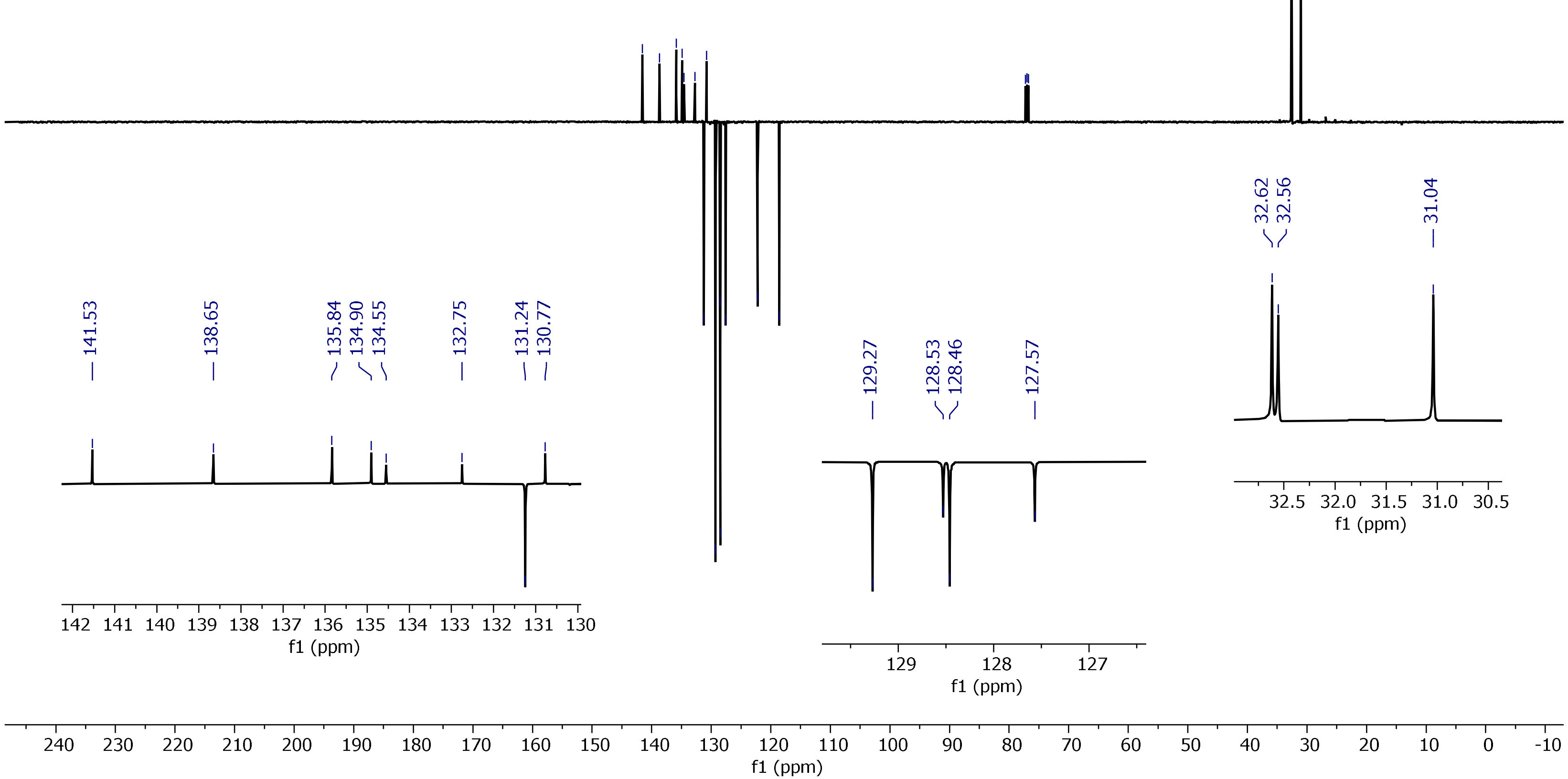

Figure S111. ^{1}H NMR ($CDCl_3$, 500 MHz) spectrum **2e**-(***Z***)

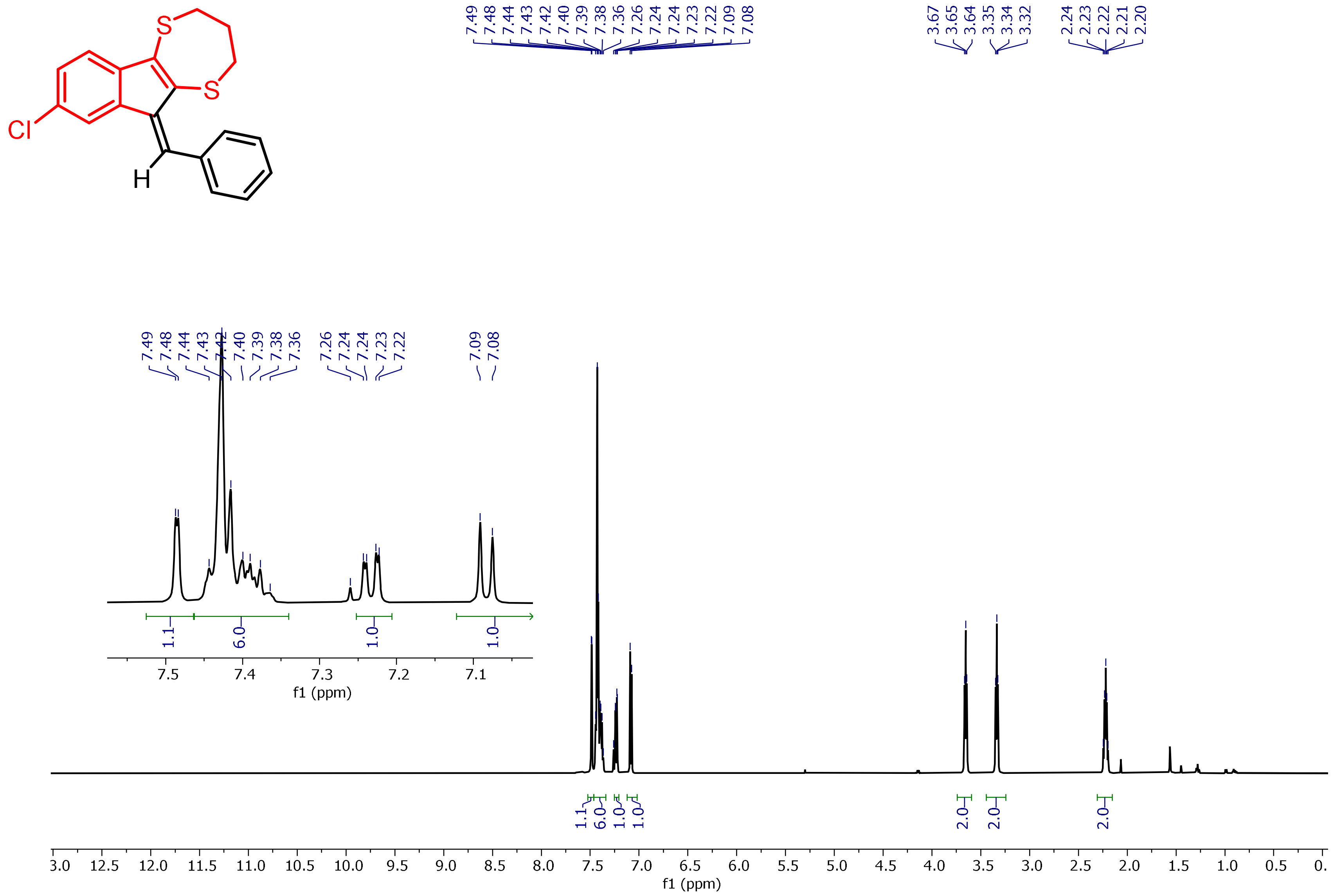

Figure S112. ${}^{13}C\{{}^{1}H\}$ NMR (126 MHz, $CDCl_3$, APT) spectrum **2e-(*Z*)**

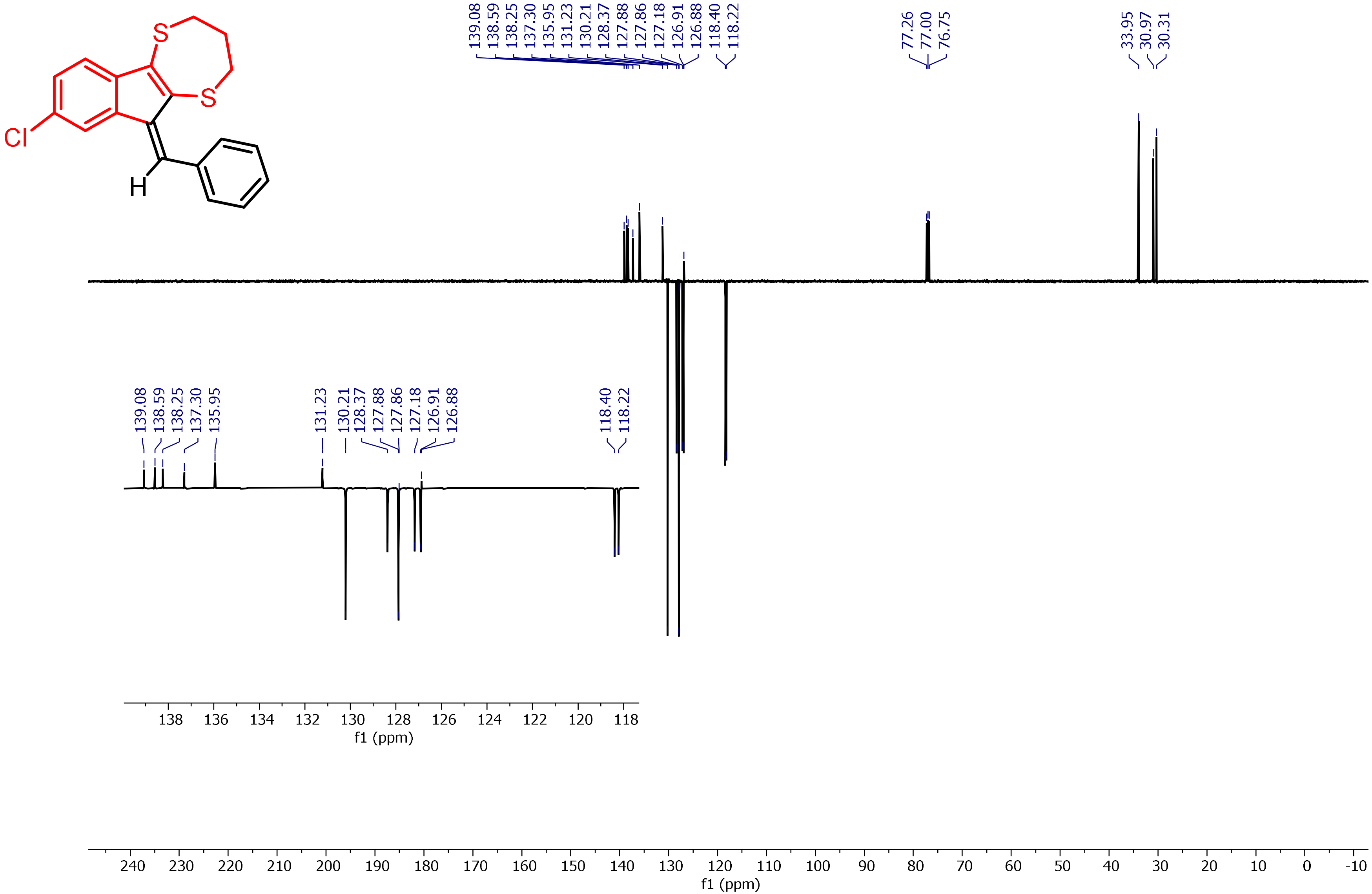

Figure S113. ^{1}H NMR ($CDCl_3$, 500 MHz) spectrum **2f-(*E*)**

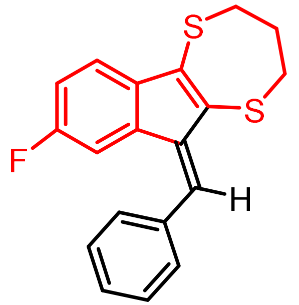

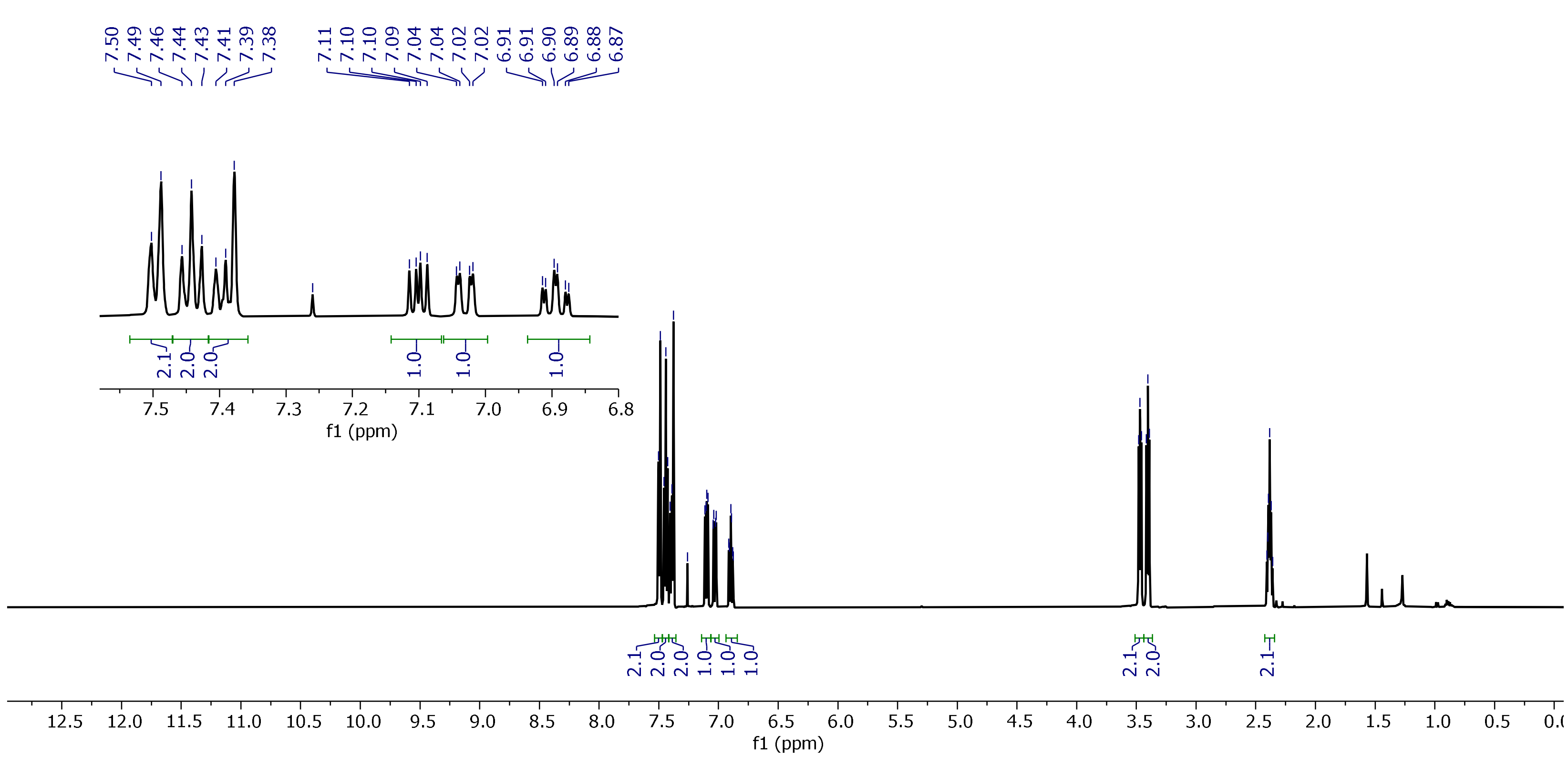

Figure S114. $^{13}C\{^1H\}$ NMR (126 MHz, $CDCl_3$, APT) spectrum **2f-(*E*)**

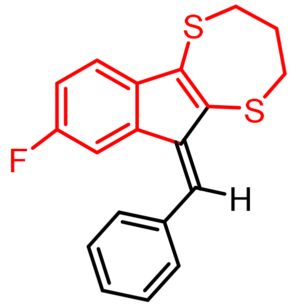

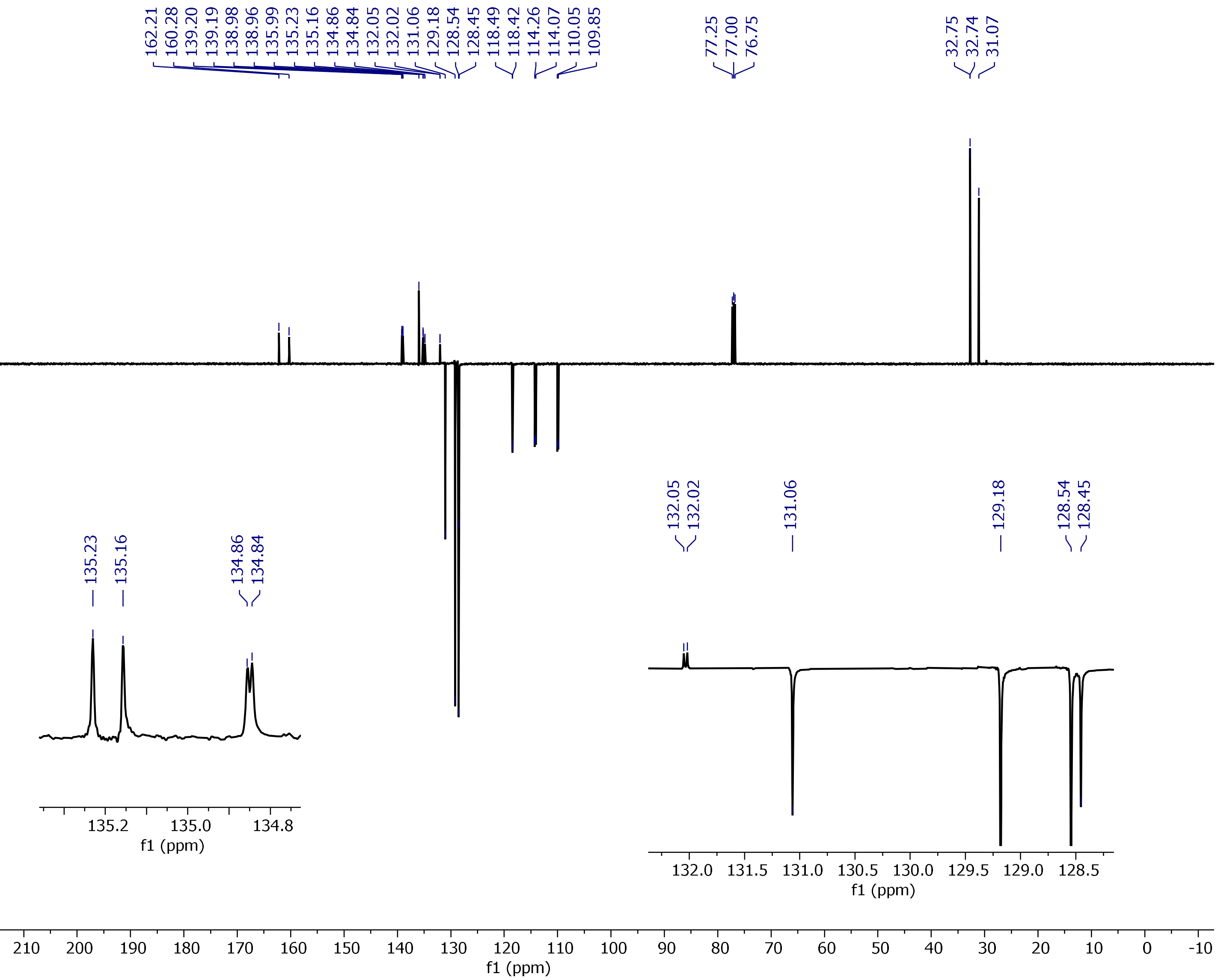

Figure S115. ^{1}H NMR ($CDCl_3$, 500 MHz) spectrum **2f**-(***Z***)

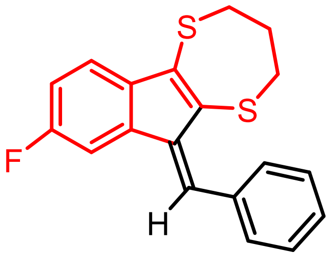

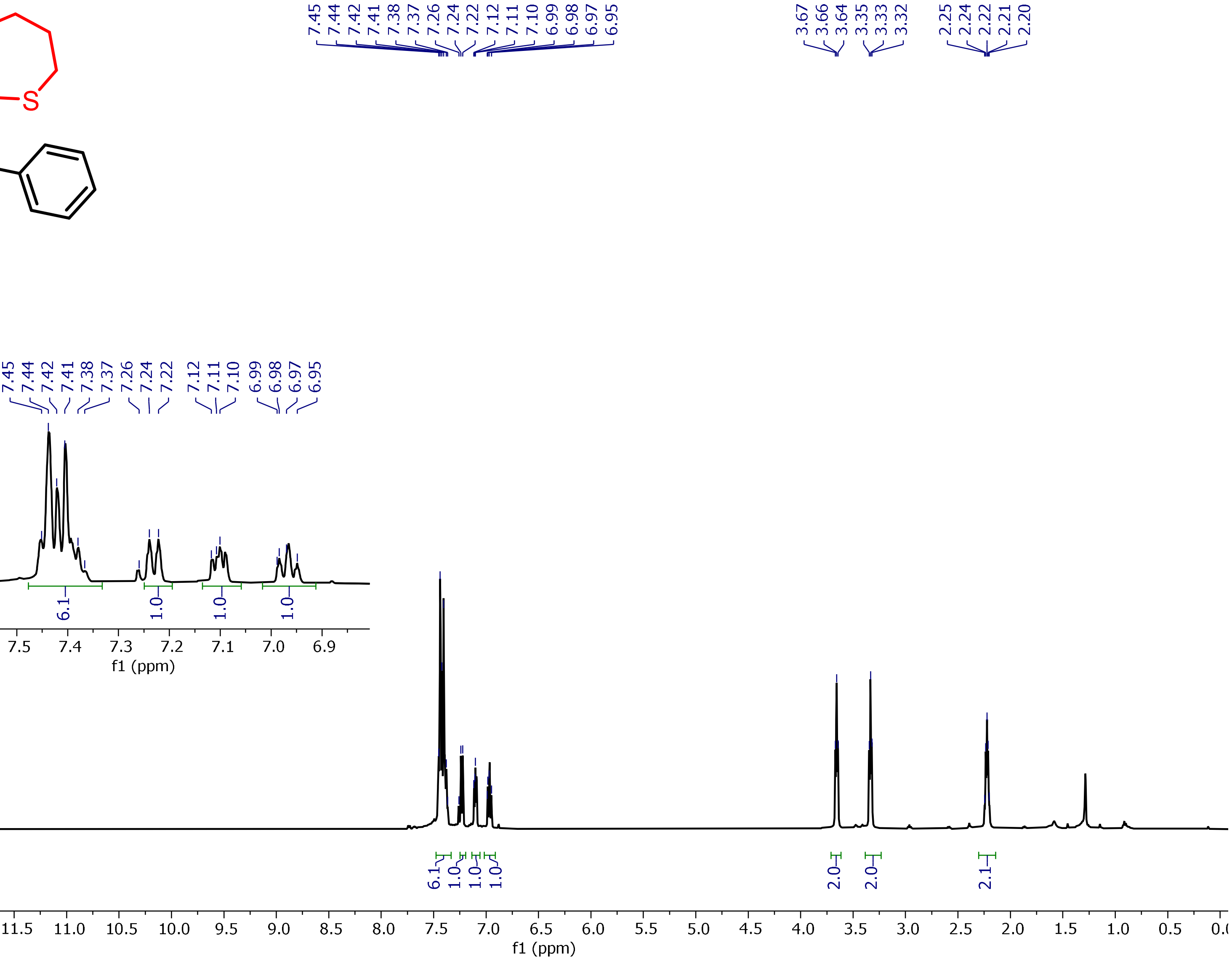

Figure S116. $^{13}C\{^{1}H\}$ NMR (126 MHz, $CDCl_3$, APT) spectrum **2f-(*Z*)**

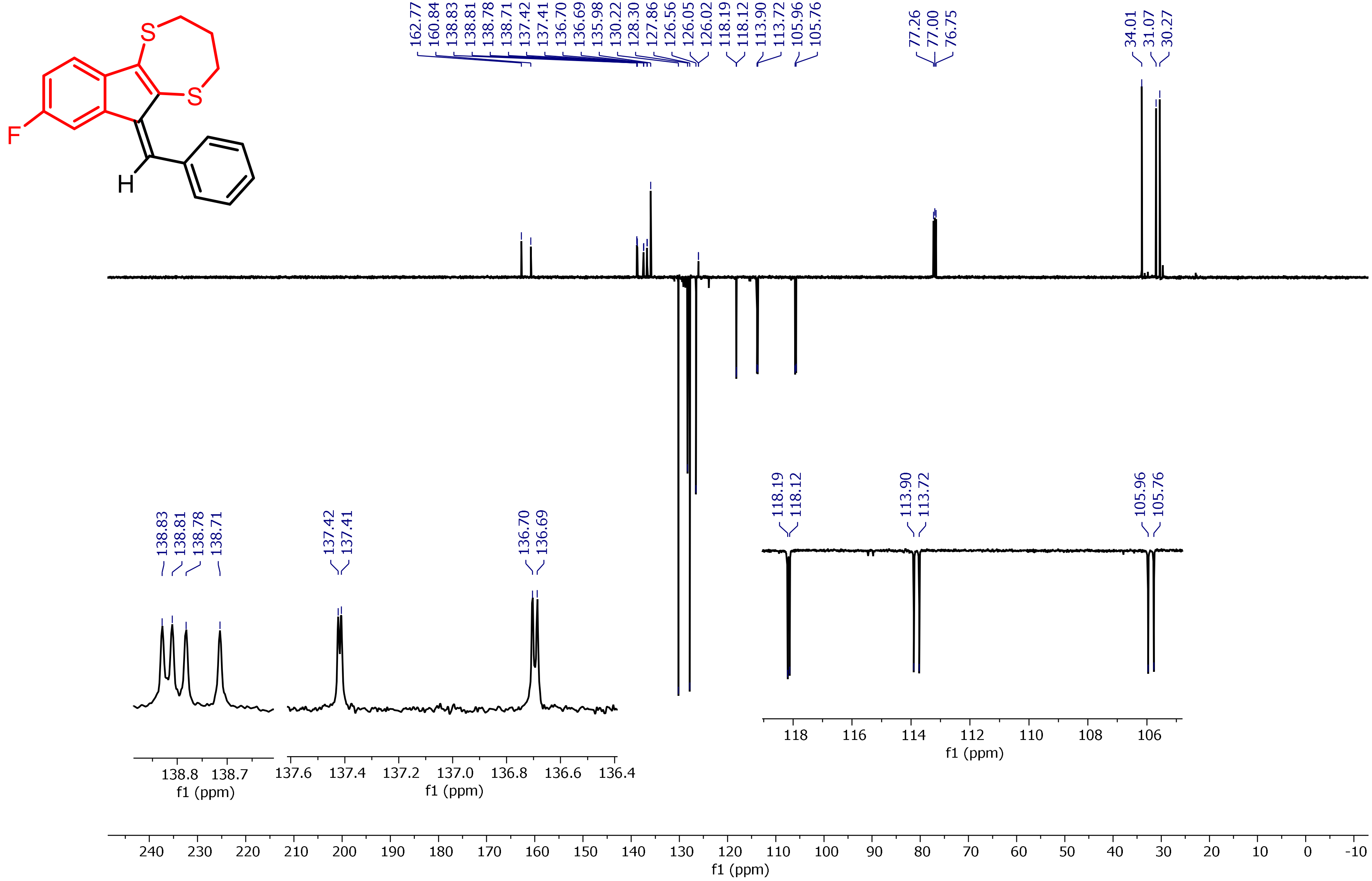

Figure S117. ^{1}H NMR ($CDCl_3$, 500 MHz) spectrum **2g**-(***E***)

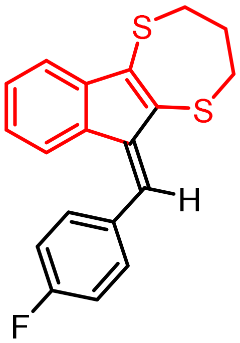

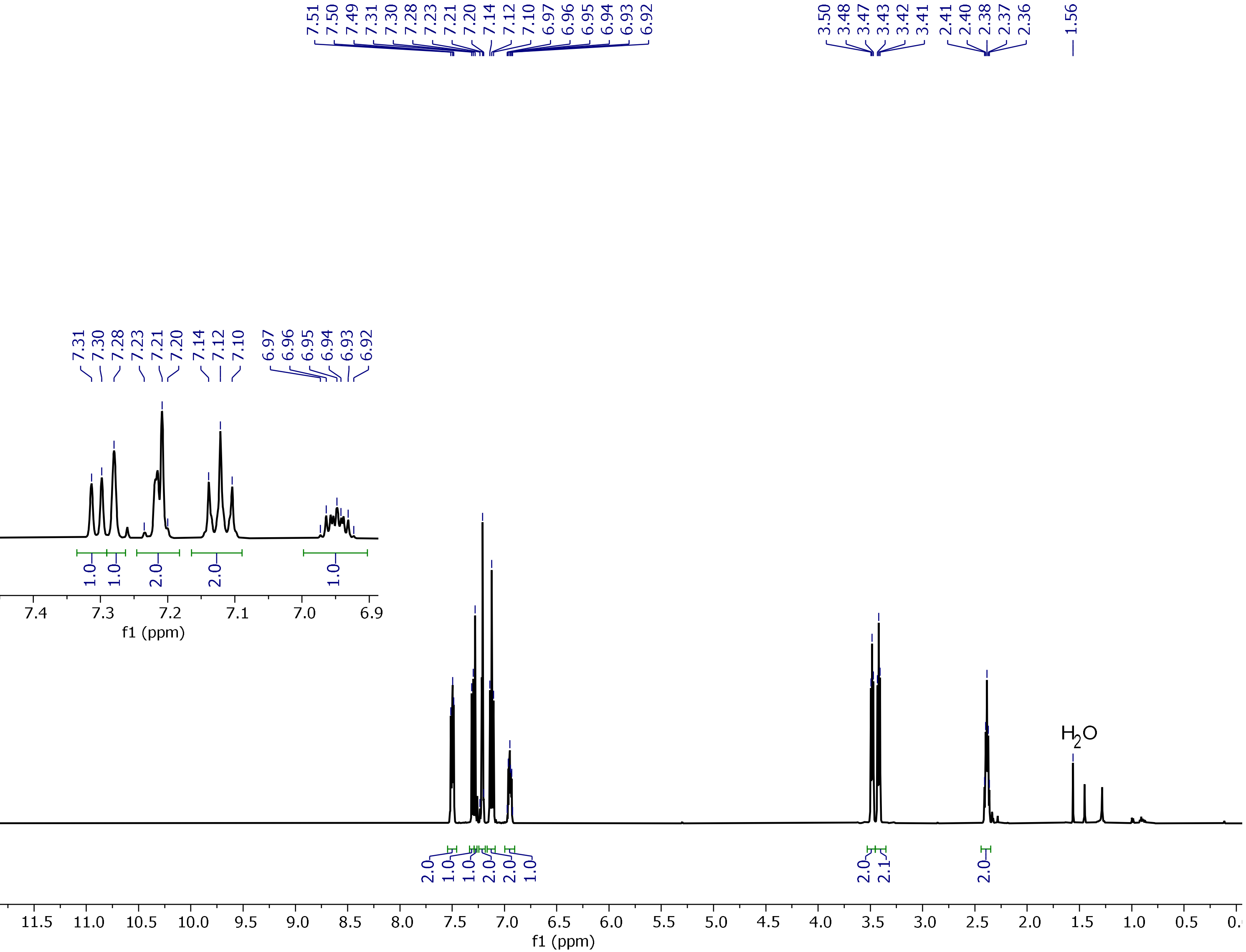

Figure S118. $^{13}C\{^1H\}$ NMR (126 MHz, $CDCl_3$, APT) spectrum **2g-(*E*)**

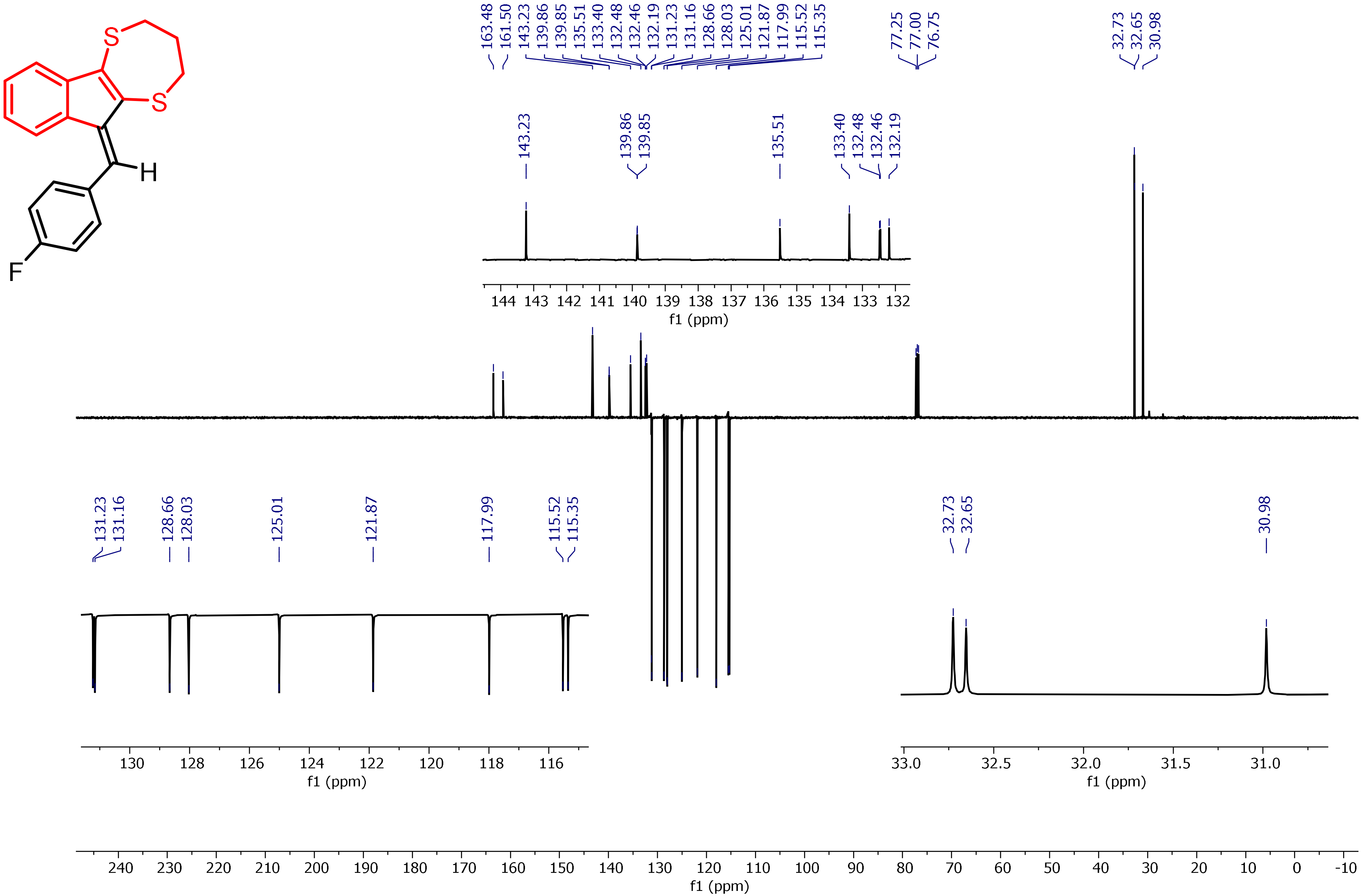

Figure S119. ^{1}H NMR ($CDCl_3$, 500 MHz) spectrum **2g-(*Z*)**

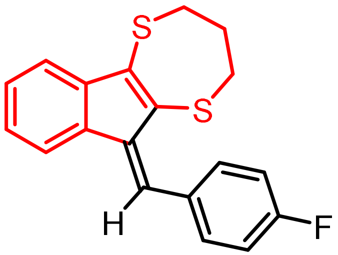

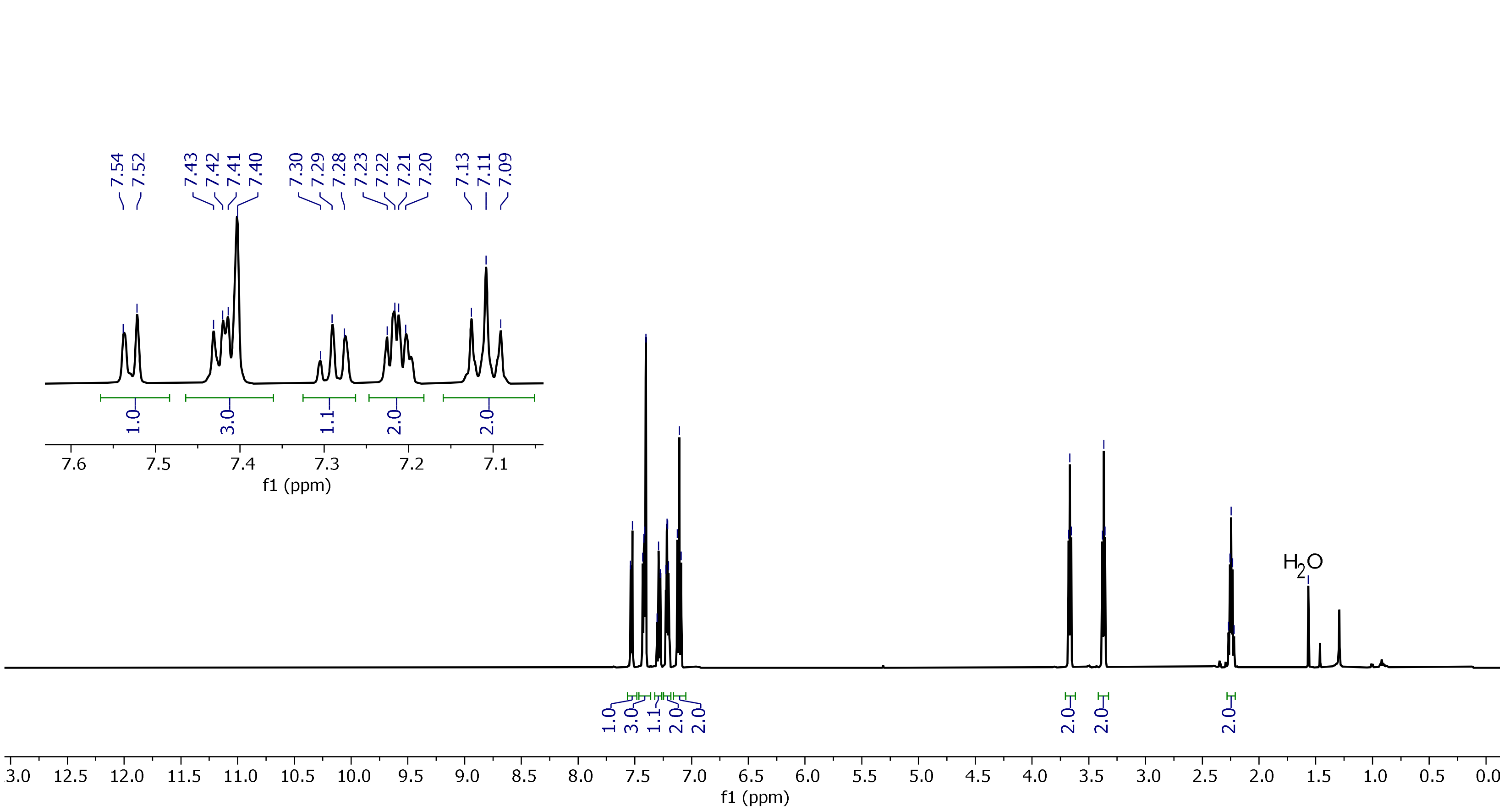

Figure S120. $^{13}C\{^1H\}$ NMR (126 MHz, $CDCl_3$, APT) spectrum **2g-(*Z*)**

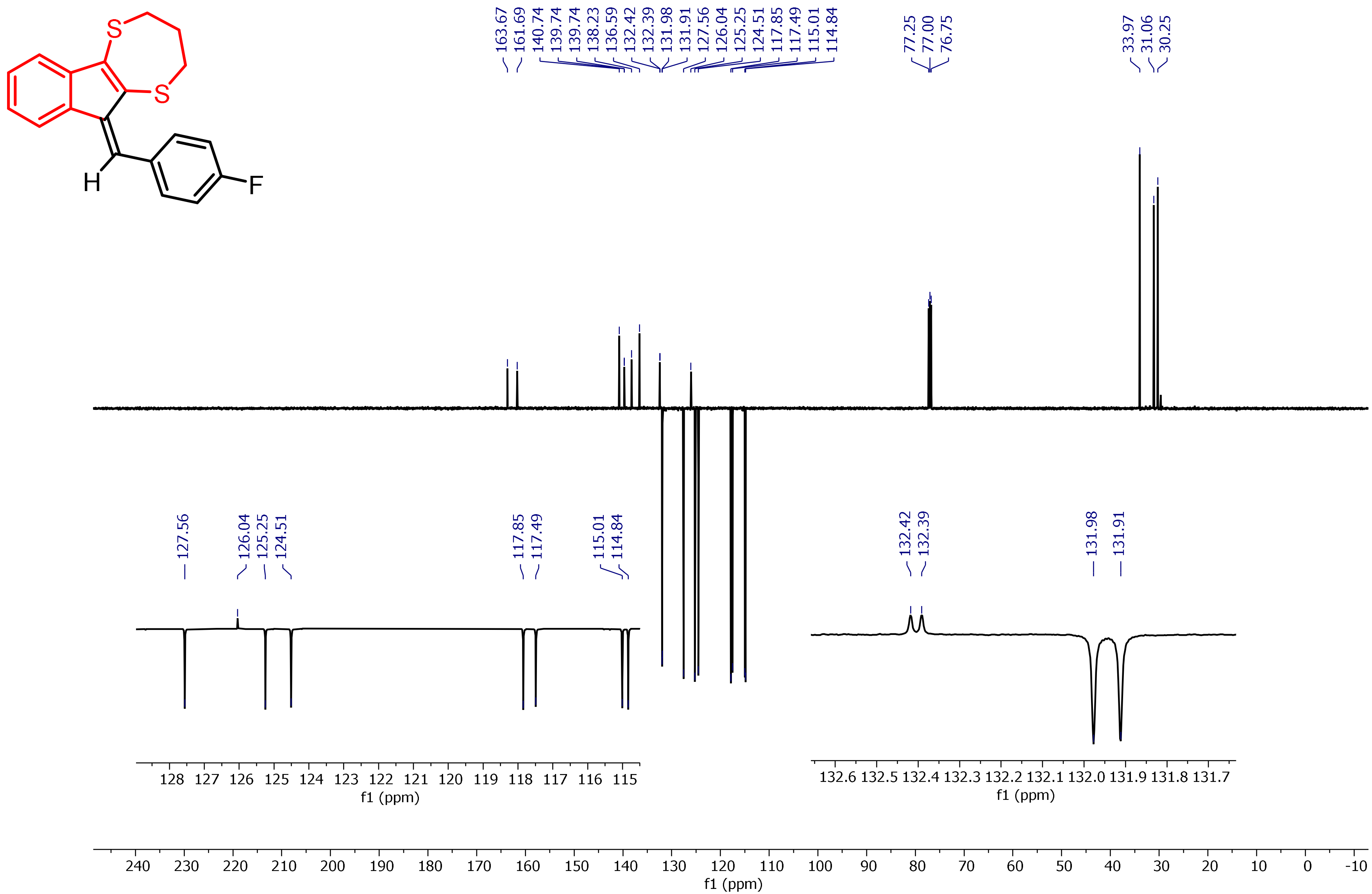

Figure S121. ^{1}H NMR ($CDCl_3$, 500 MHz) spectrum **2h**-(***E***)

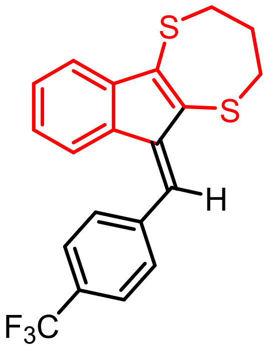

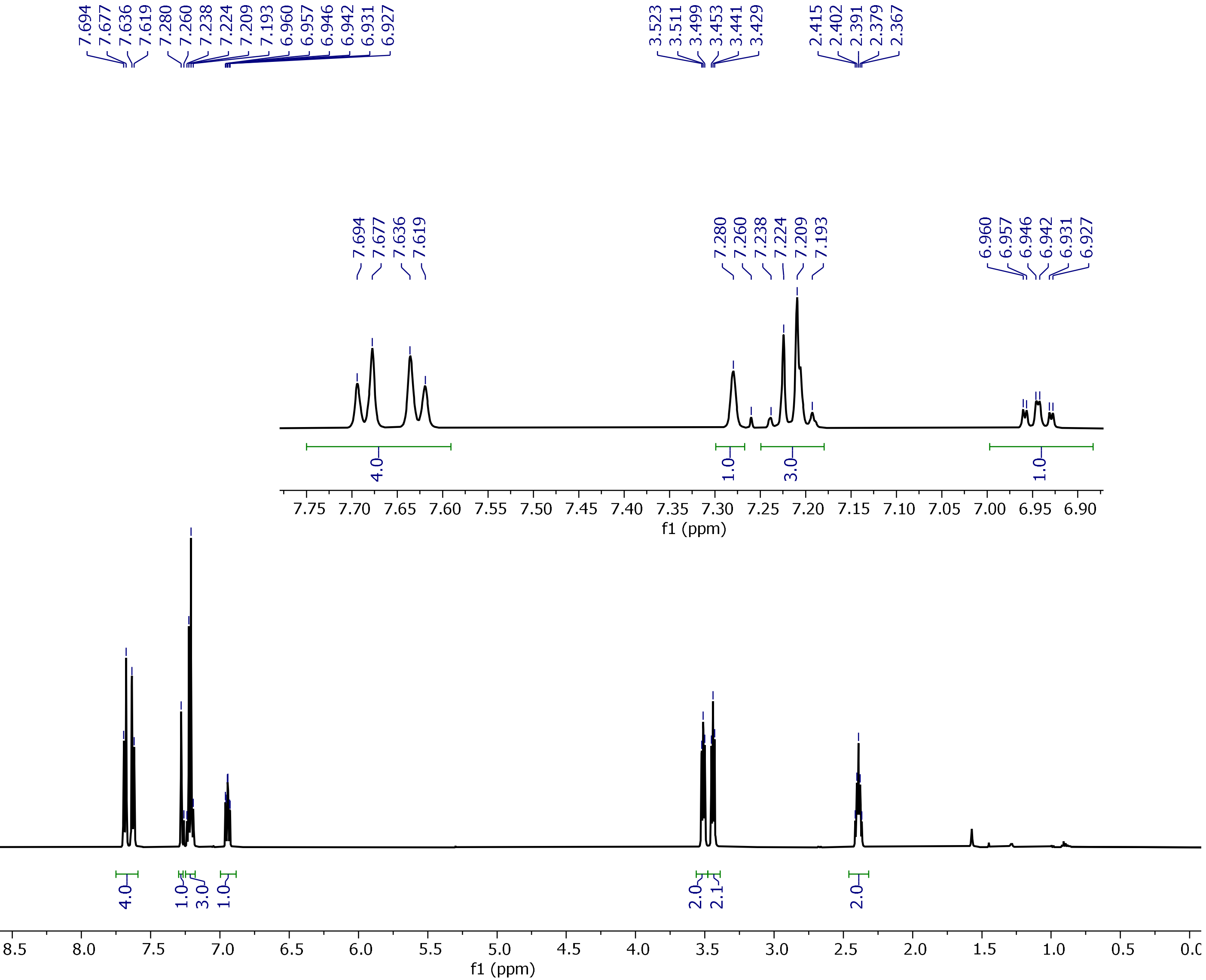

Figure S122. $^{13}C\{^1H\}$ NMR (126 MHz, $CDCl_3$, APT) spectrum **2h-(*E*)**

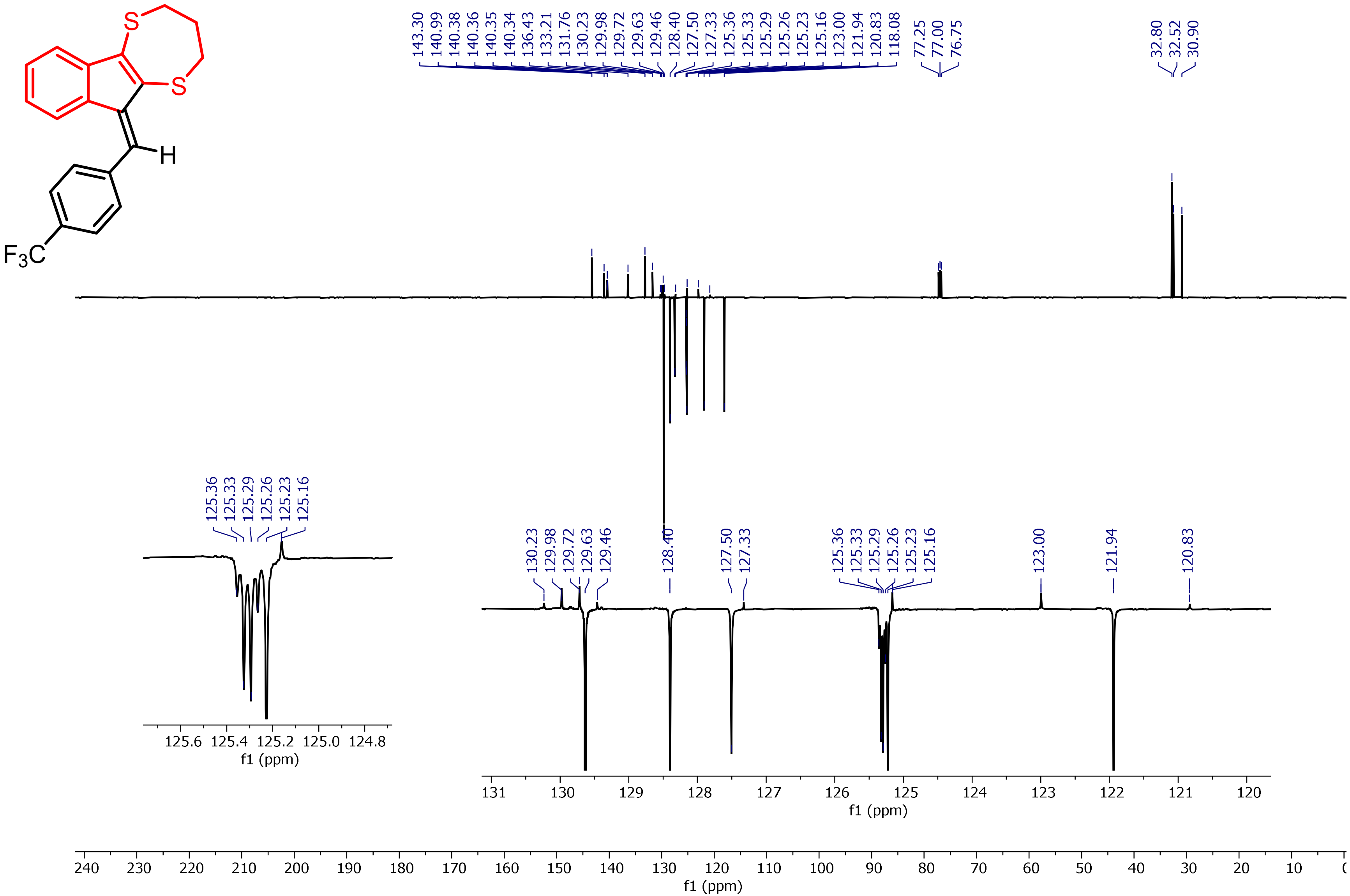

Figure S123. ^{1}H NMR ($CDCl_3$, 500 MHz) spectrum **2h**-(***Z***)

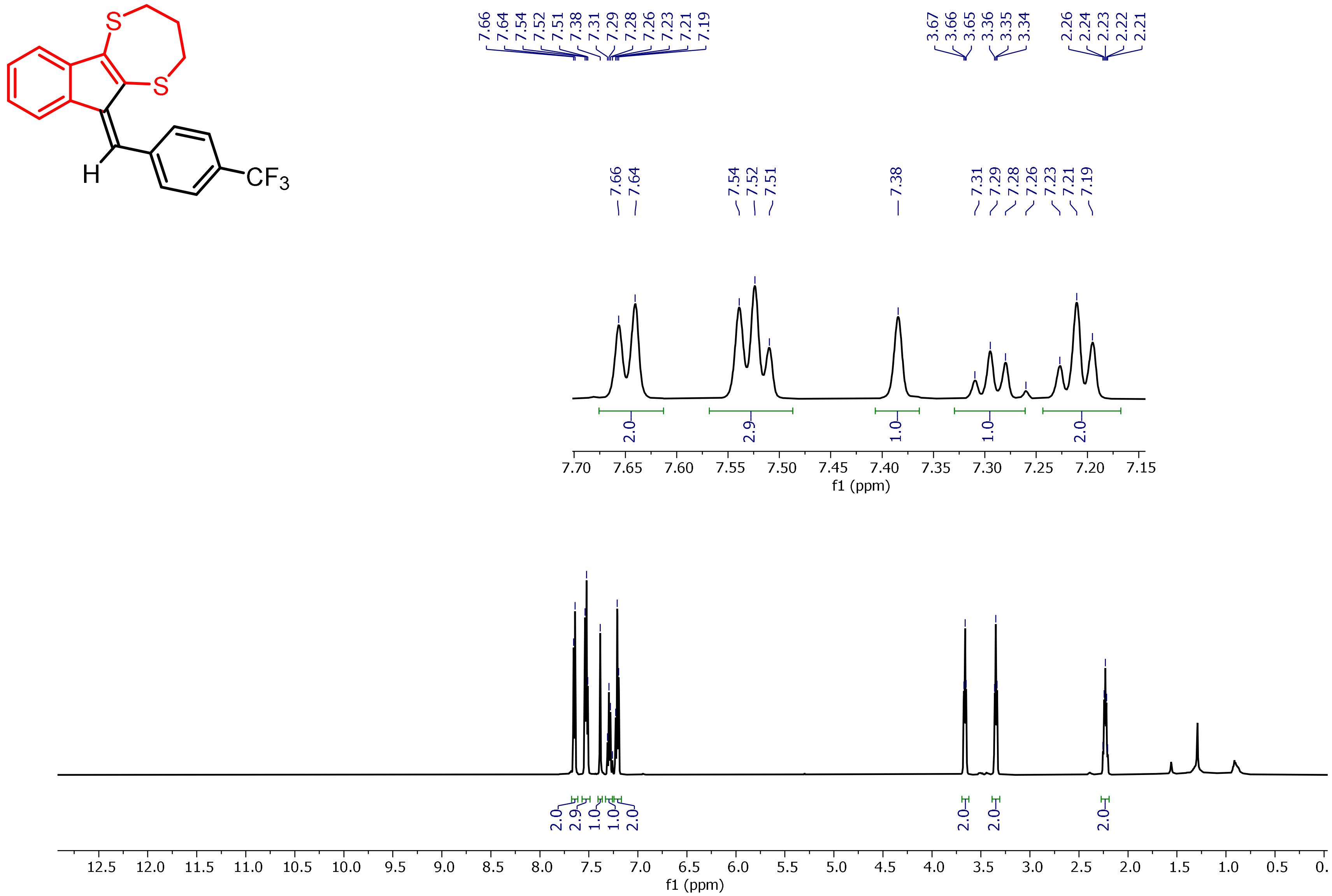

Figure S124. $^{13}C\{^{1}H\}$ NMR (126 MHz, $CDCl_3$, APT) spectrum **2h-(*Z*)**

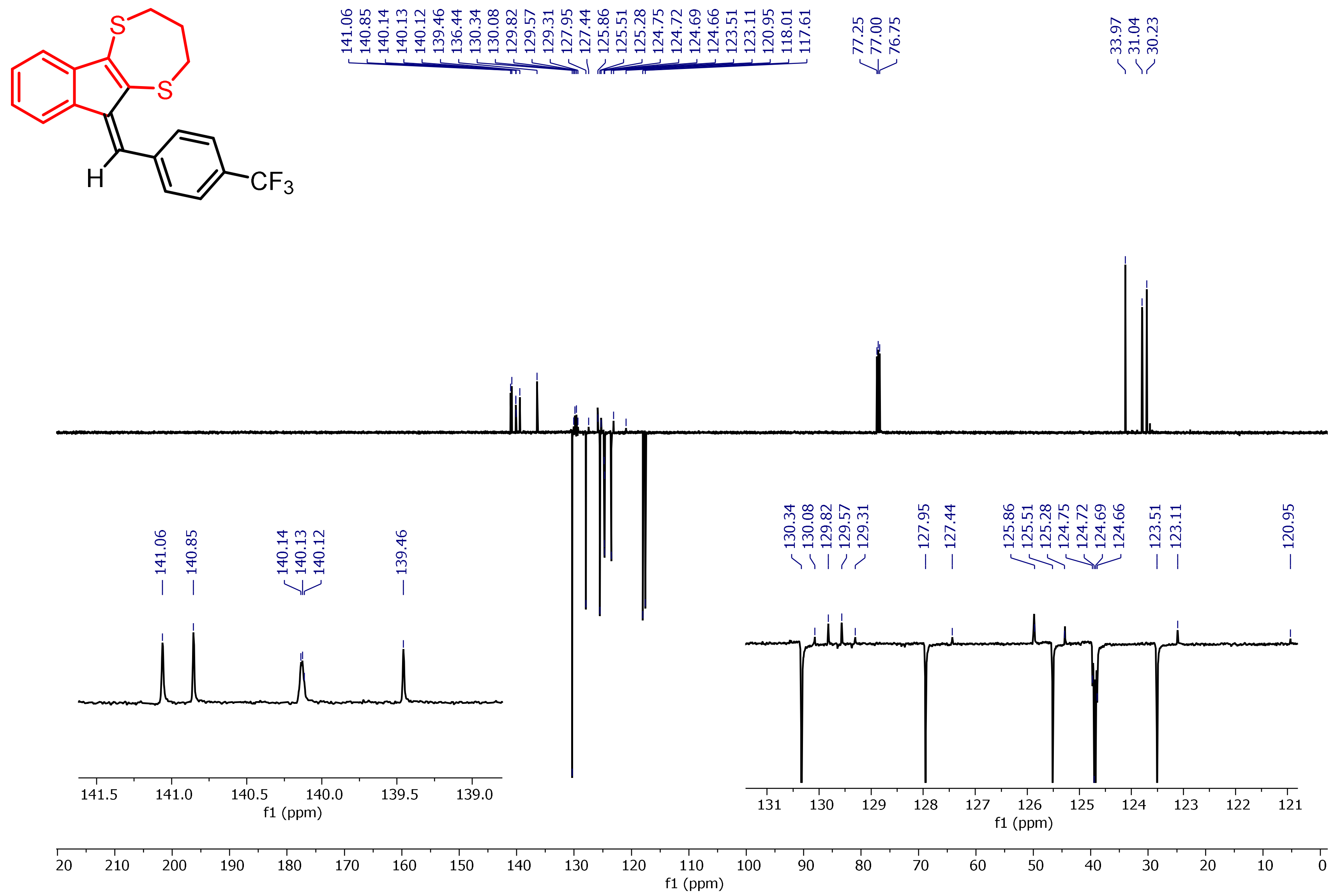

Figure S125. ^{1}H NMR ($CDCl_3$, 500 MHz) spectrum **2i**-(***E*/*Z***)

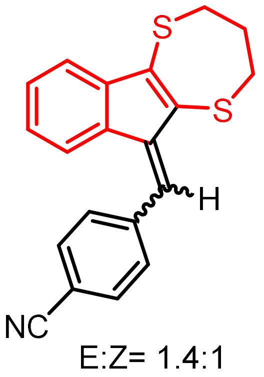

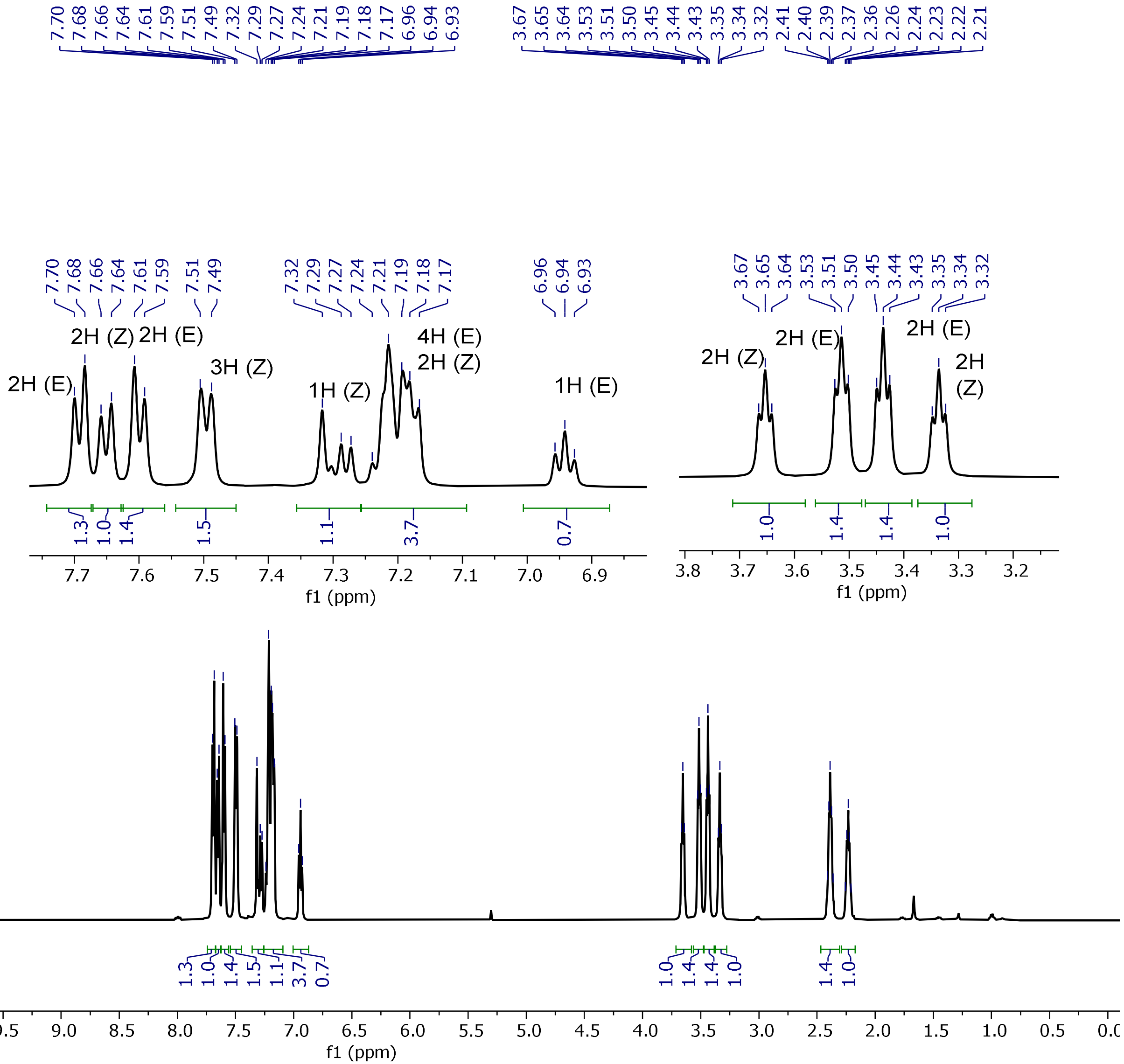

Figure S126. $^{13}C\{^{1}H\}$ NMR (126 MHz, $CDCl_3$, APT) spectrum **2i-(*E*/*Z*)**

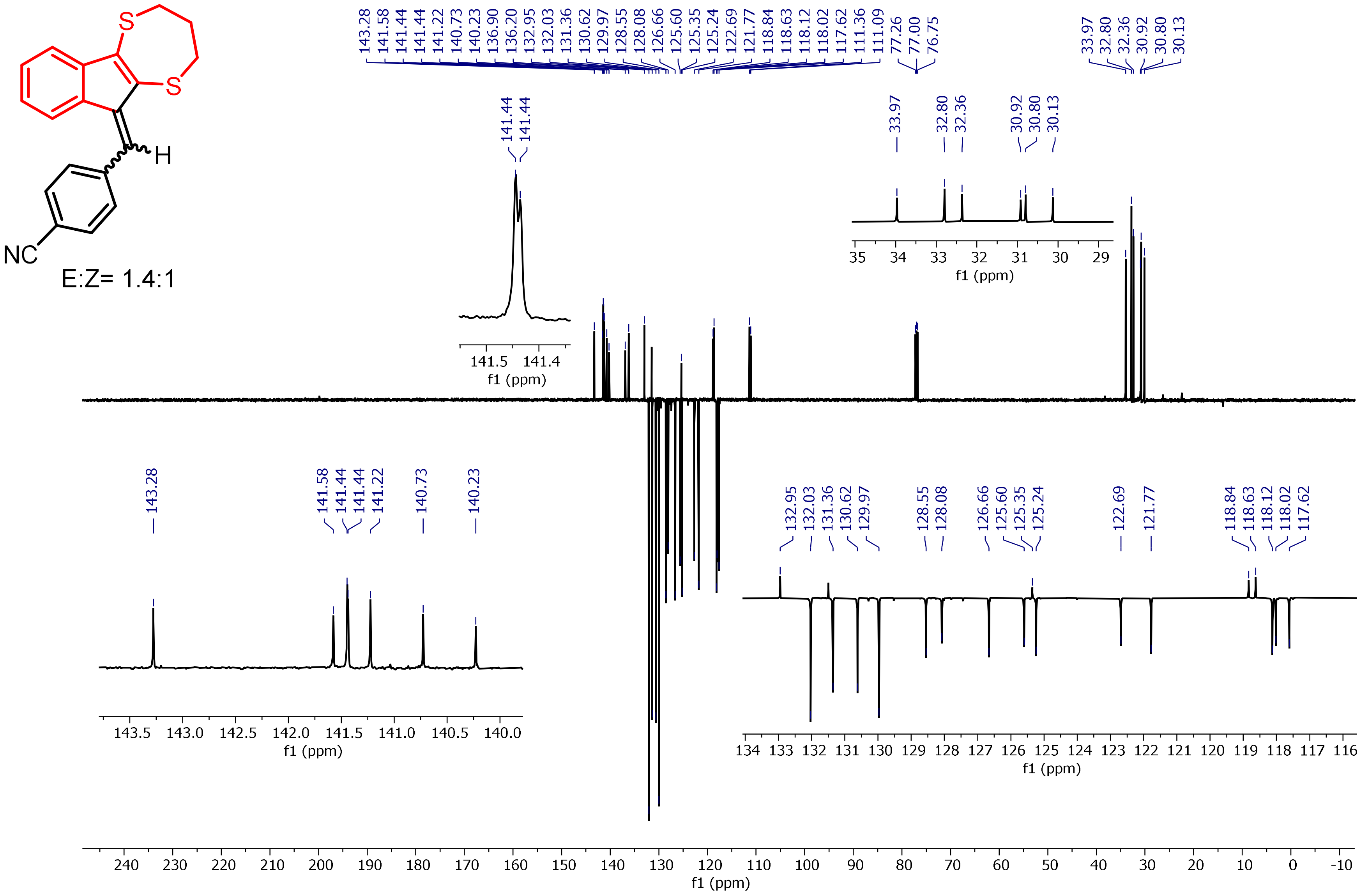

Figure S127. ^{1}H NMR ($CDCl_3$, 500 MHz) spectrum **2j**-(***E***)

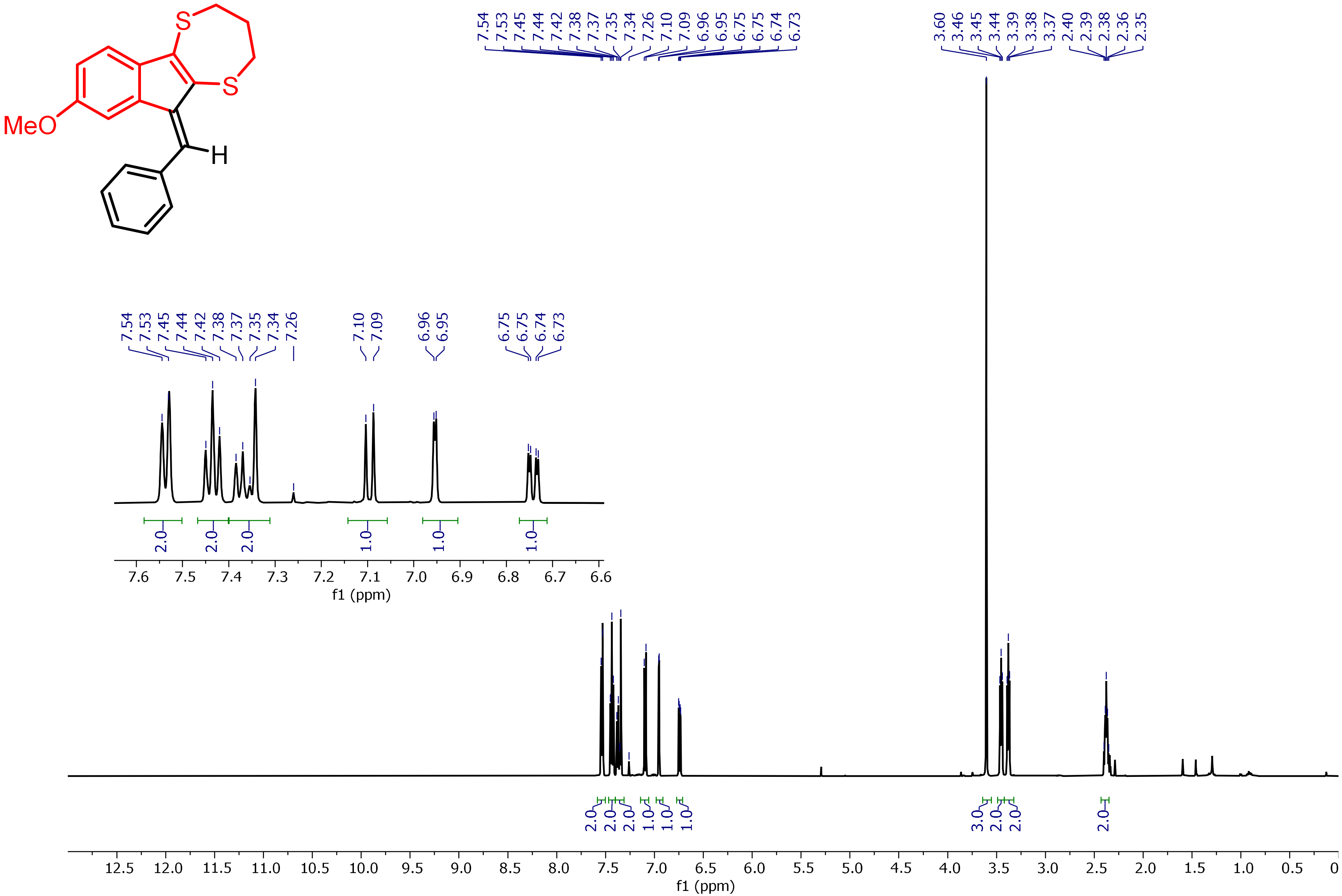

Figure S128. $^{13}C\{^1H\}$ NMR (126 MHz, $CDCl_3$, APT) spectrum **2j-(*E*)**

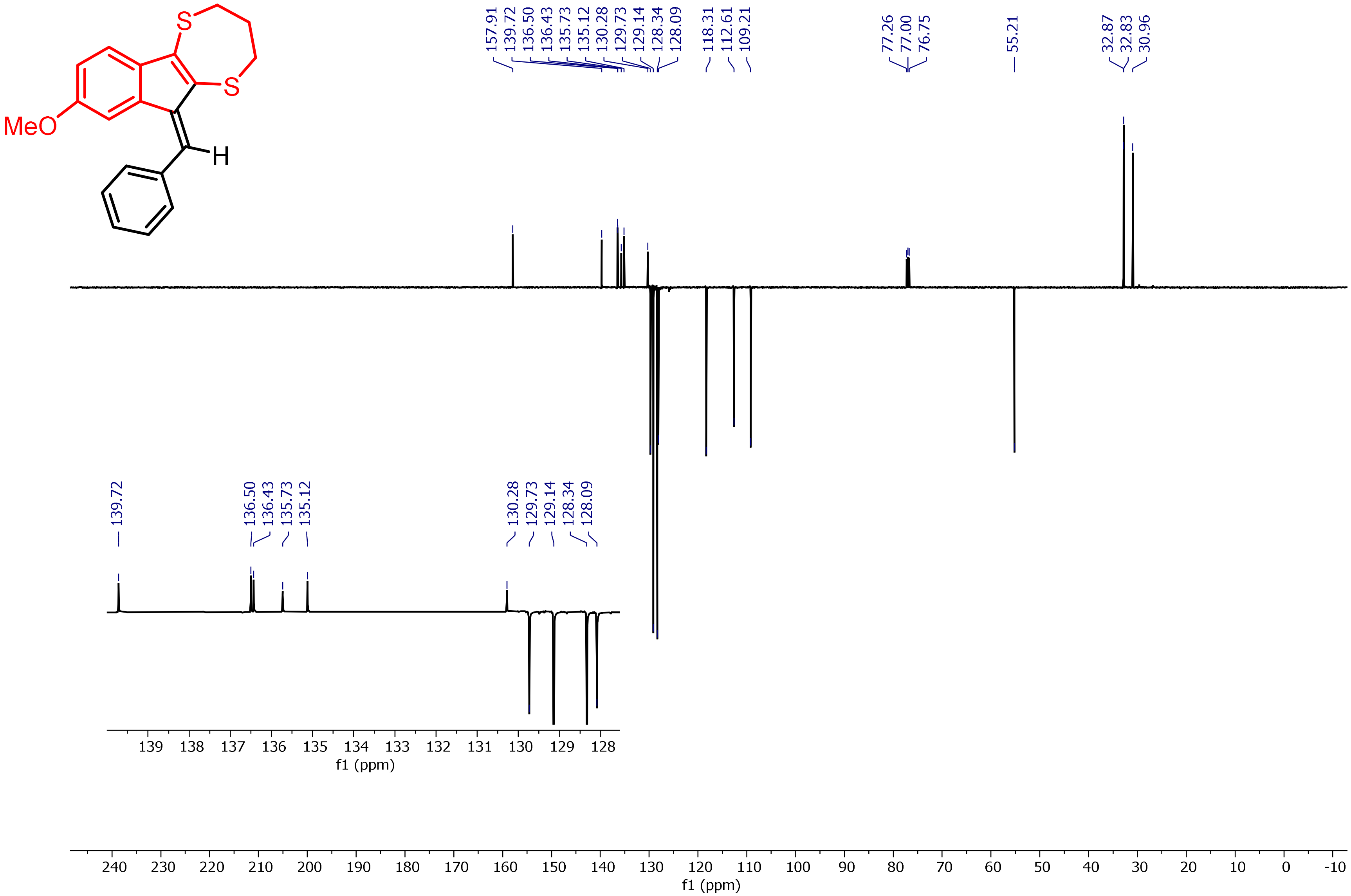

Figure S129. ^{1}H NMR ($CDCl_3$, 500 MHz) spectrum **2j**-(***Z***)

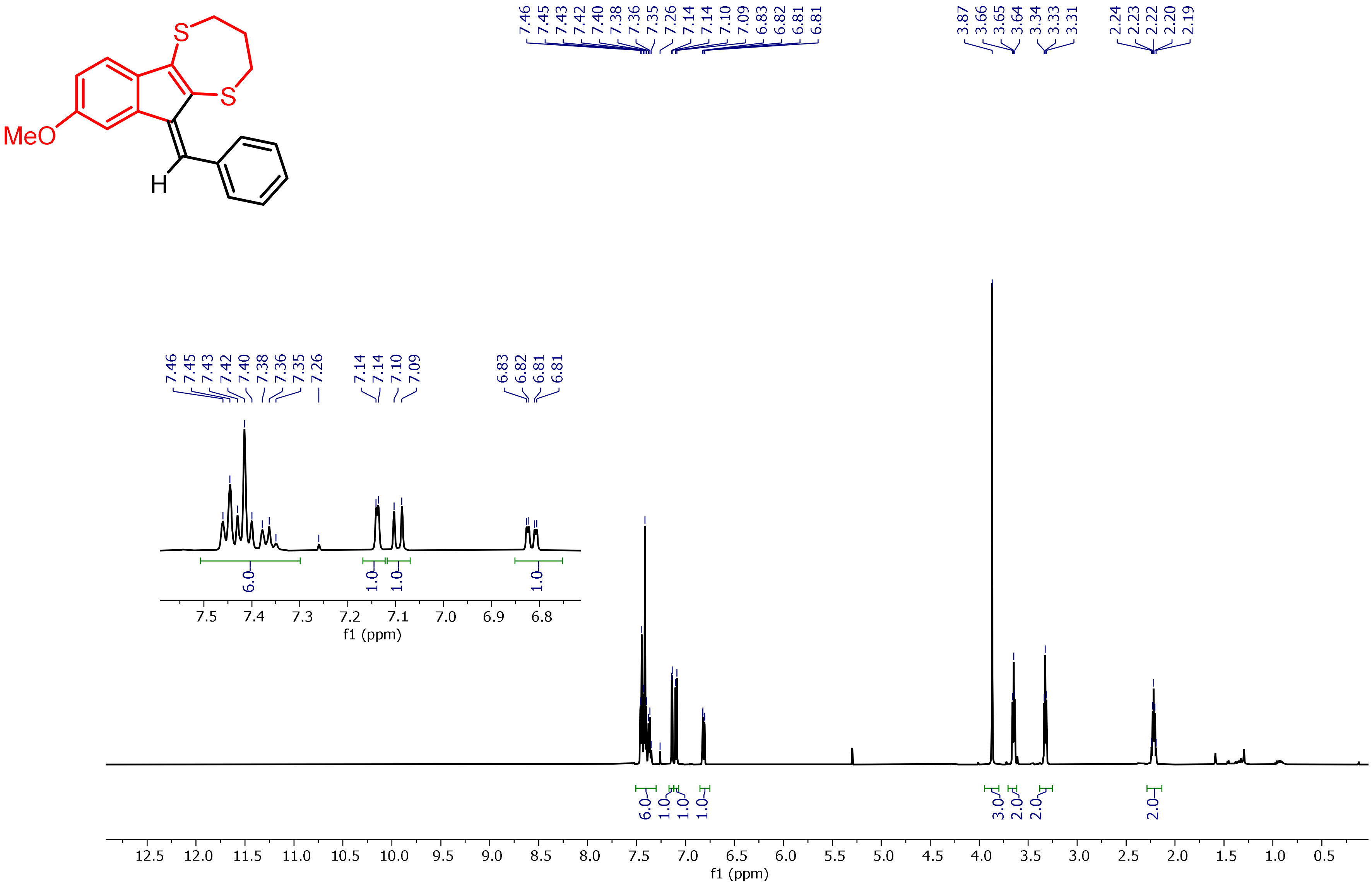

Figure S130. $^{13}C\{^{1}H\}$ NMR (126 MHz, $CDCl_3$, APT) spectrum **2j-(*Z*)**

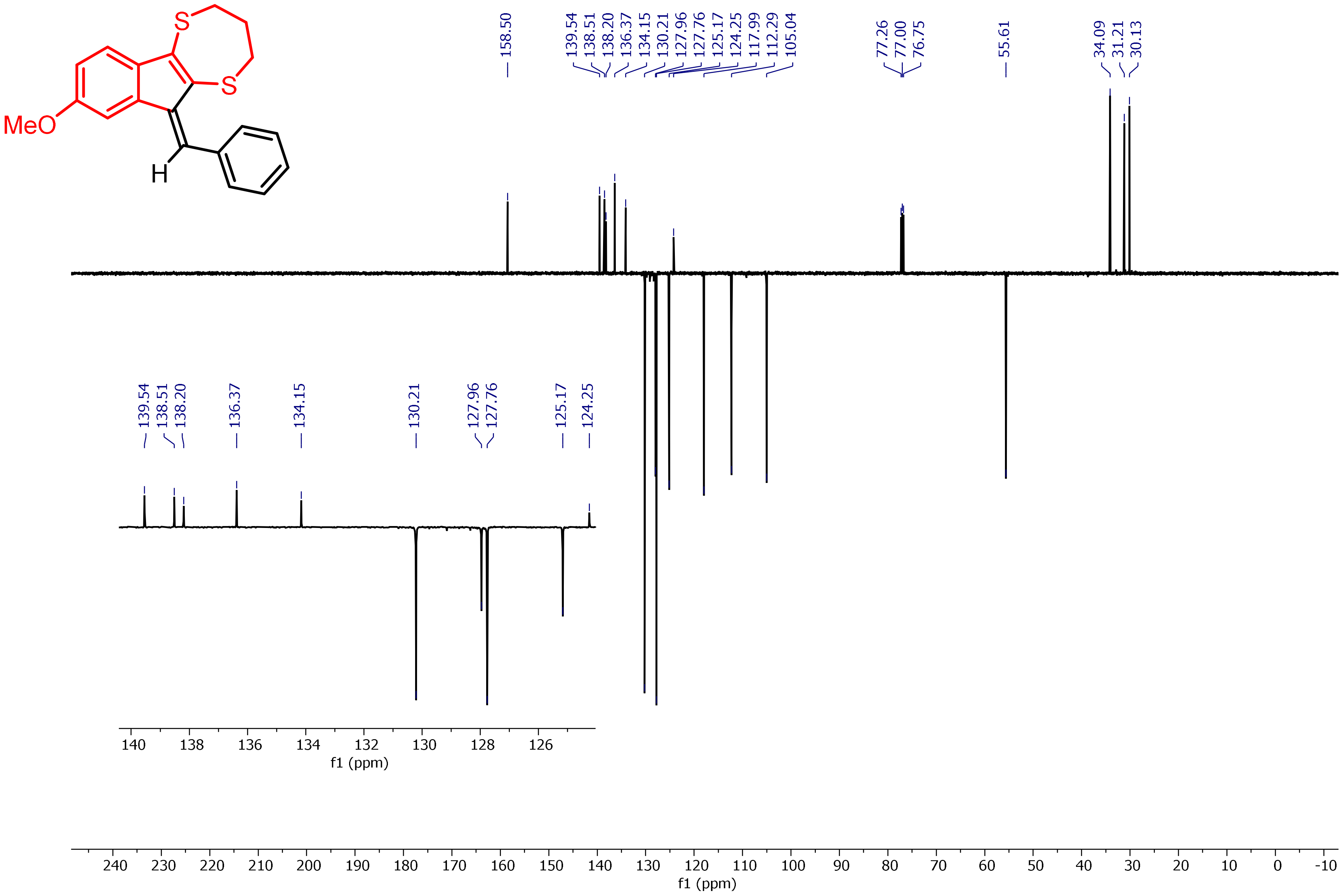

Figure S131. ^{1}H NMR ($CDCl_3$, 500 MHz) spectrum **2l**-(***E***)

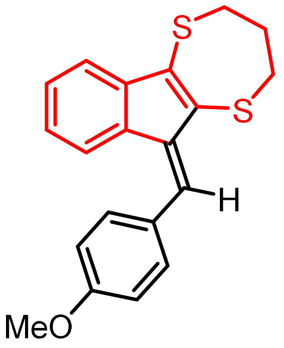

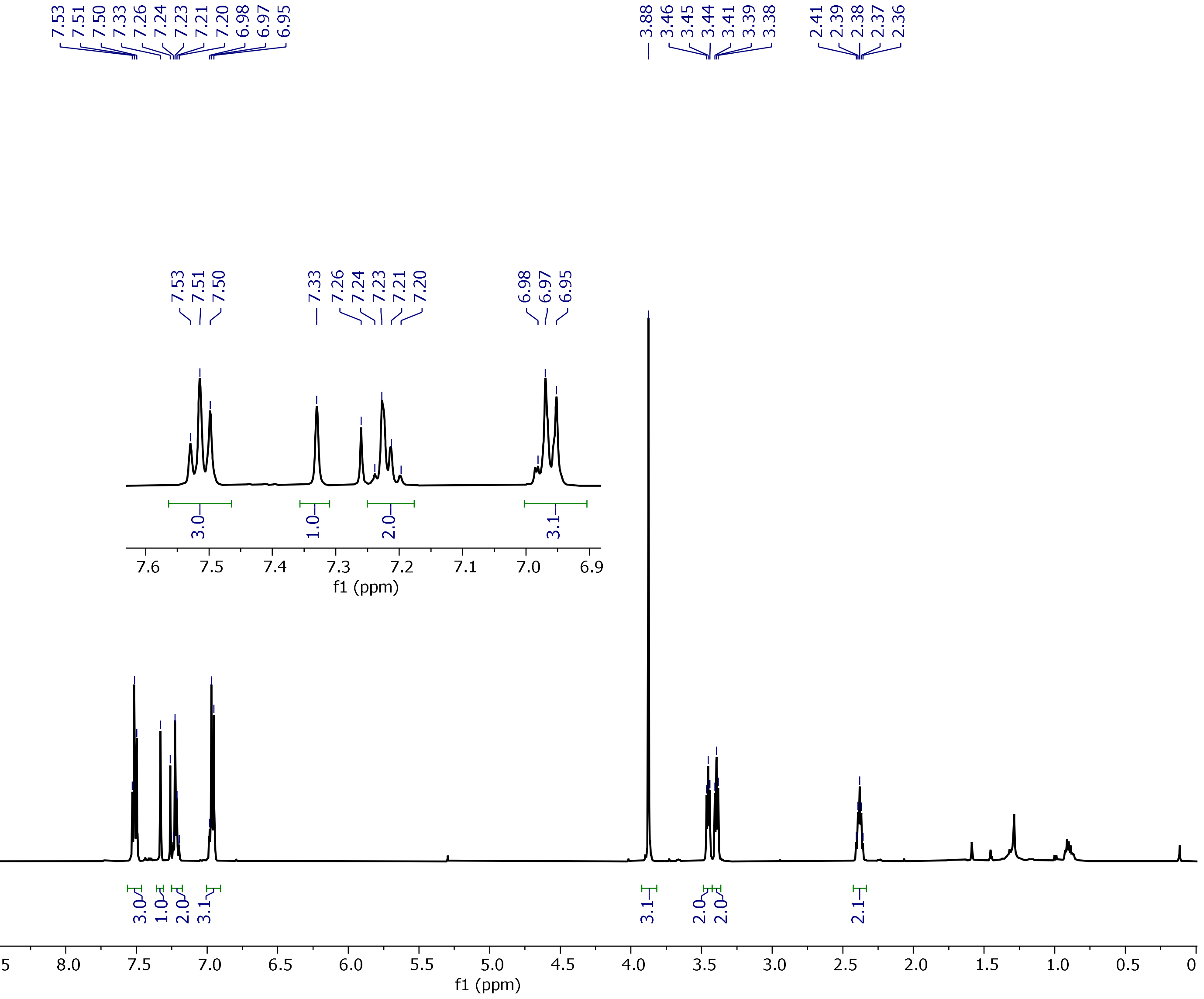

Figure S132. $^{13}C\{^1H\}$ NMR (126 MHz, $CDCl_3$, APT) spectrum **2l-(*E*)**

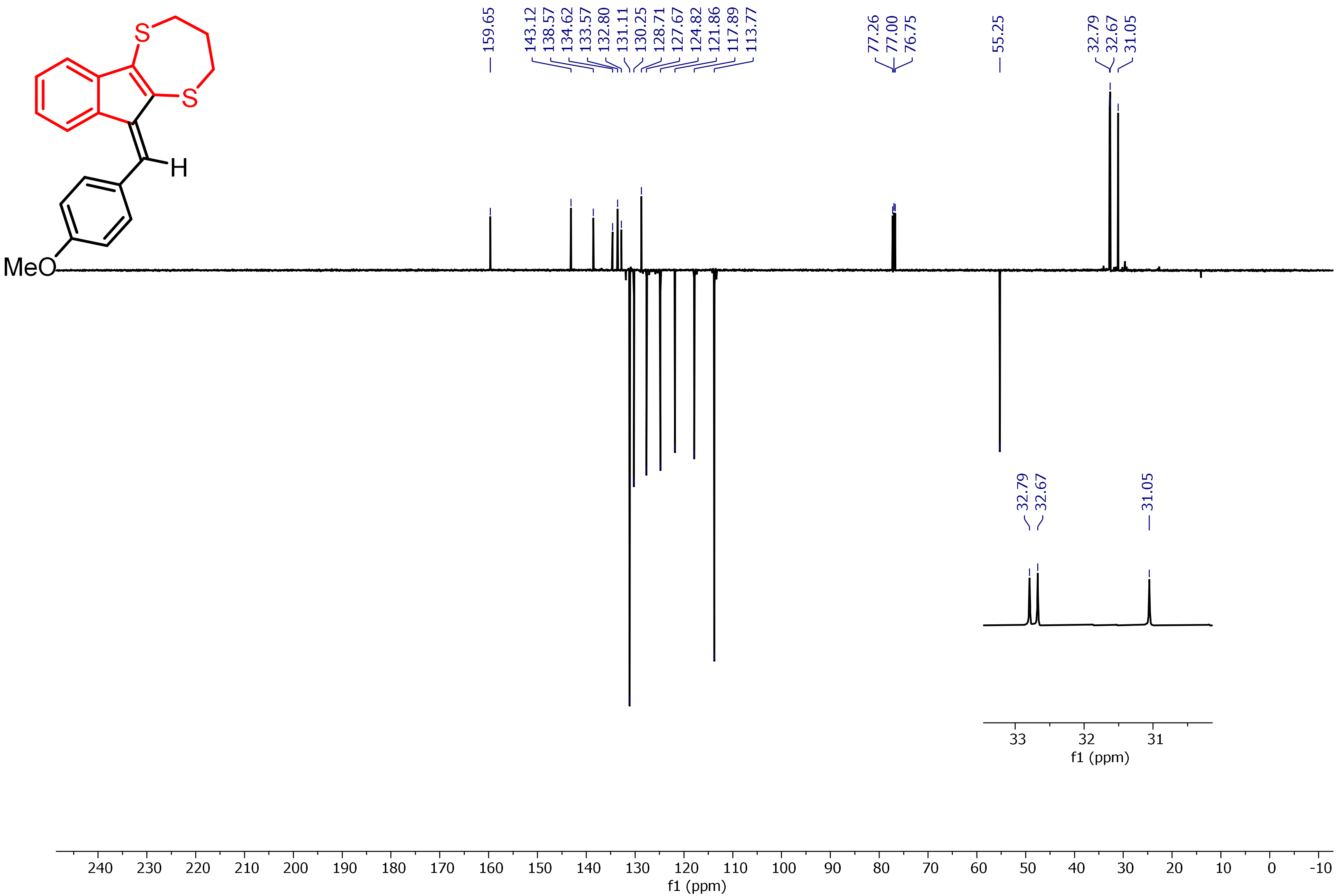

Figure S133. ^{1}H NMR ($CDCl_3$, 500 MHz) spectrum **2m**-(***E***)

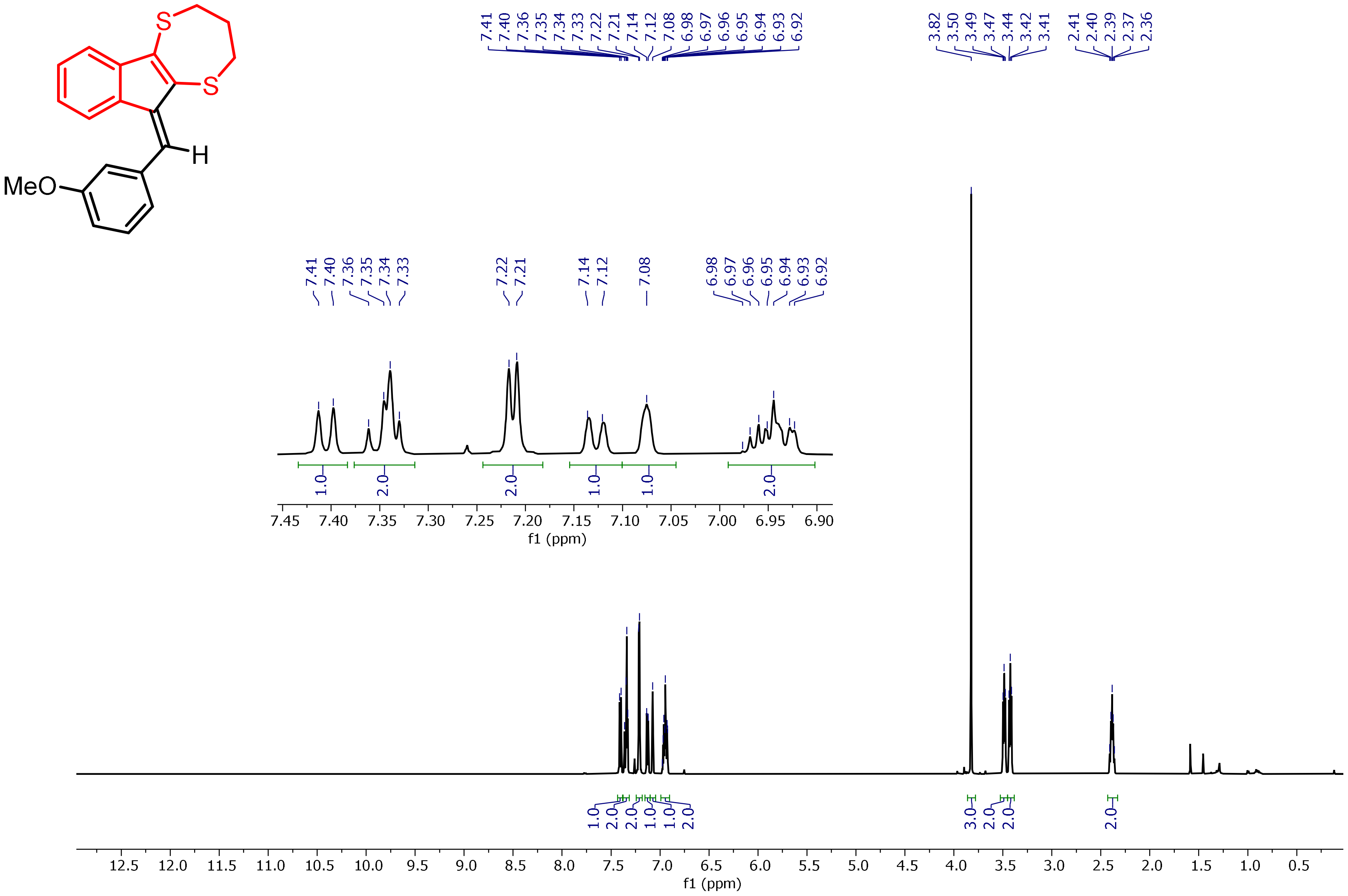

Figure S134. $^{13}C\{^{1}H\}$ NMR (126 MHz, $CDCl_3$, APT) spectrum **2m-(*E*)**

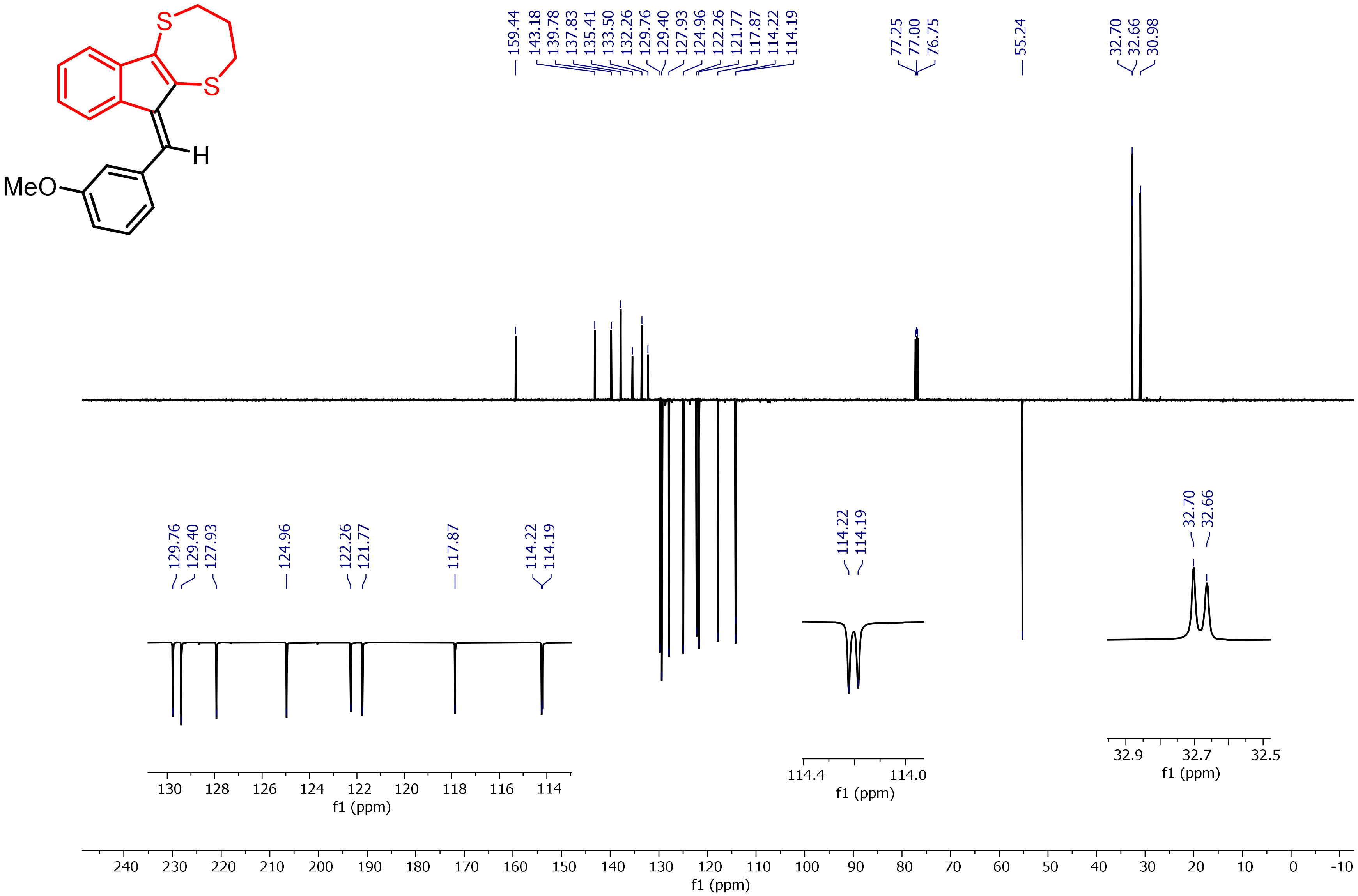

Figure S135. ^{1}H NMR ($CDCl_3$, 500 MHz) spectrum **2m-(*Z*)**

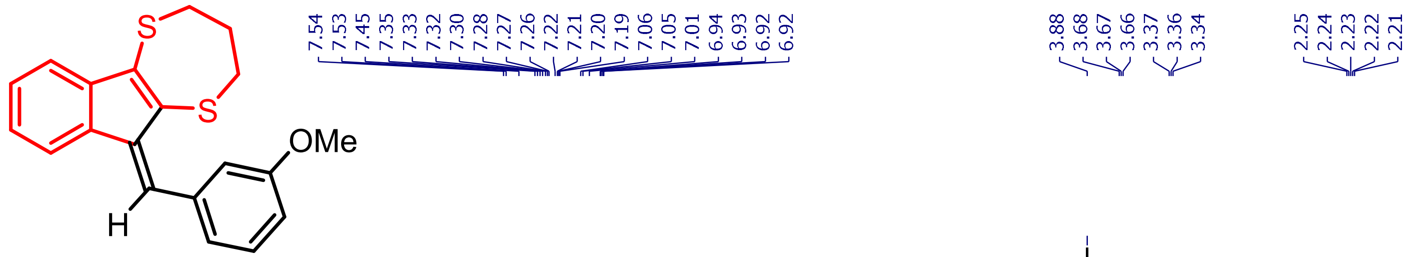

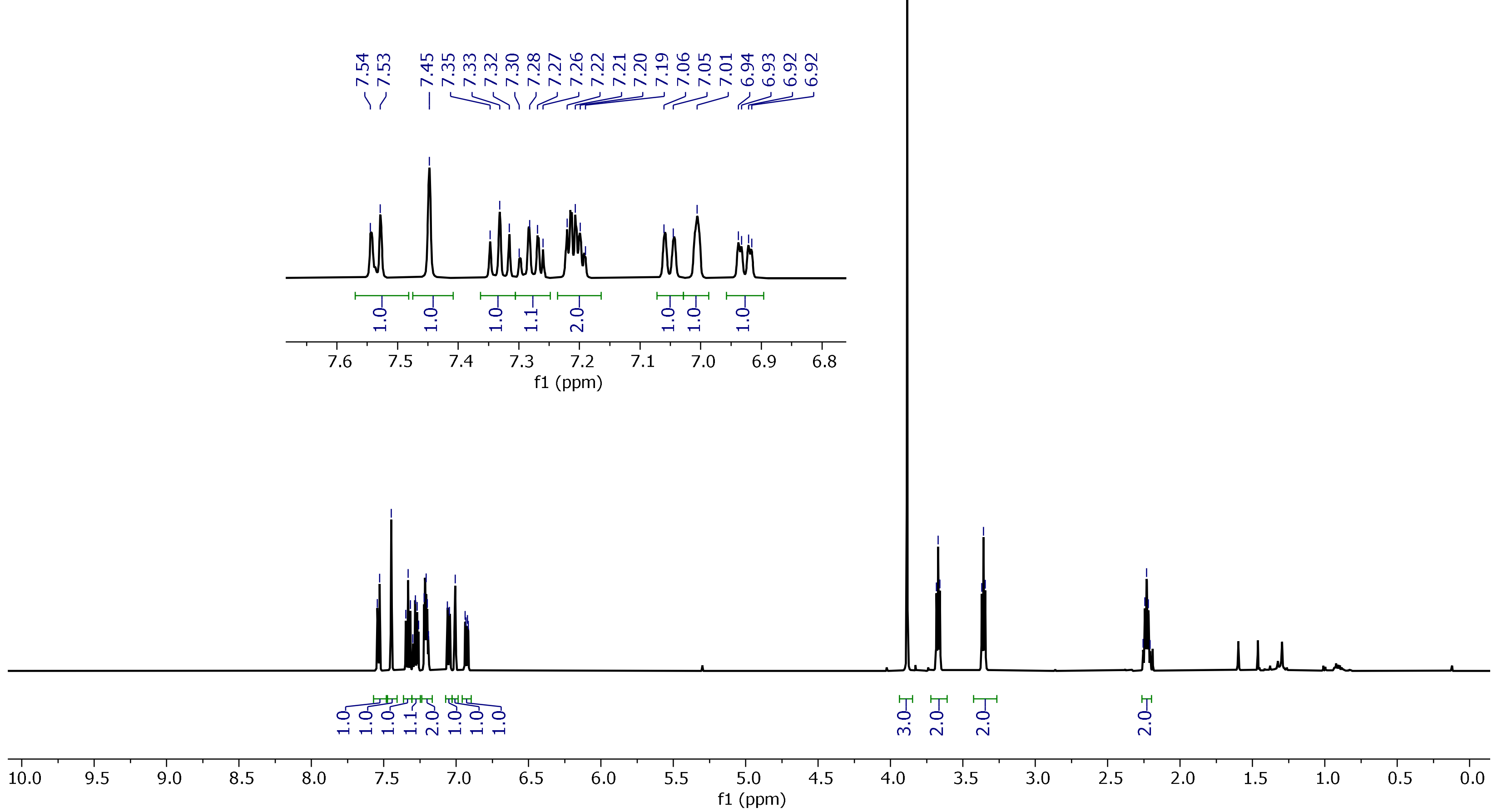

Figure S136. $^{13}C\{^1H\}$ NMR (126 MHz, $CDCl_3$, APT) spectrum **2m-(*Z*)**

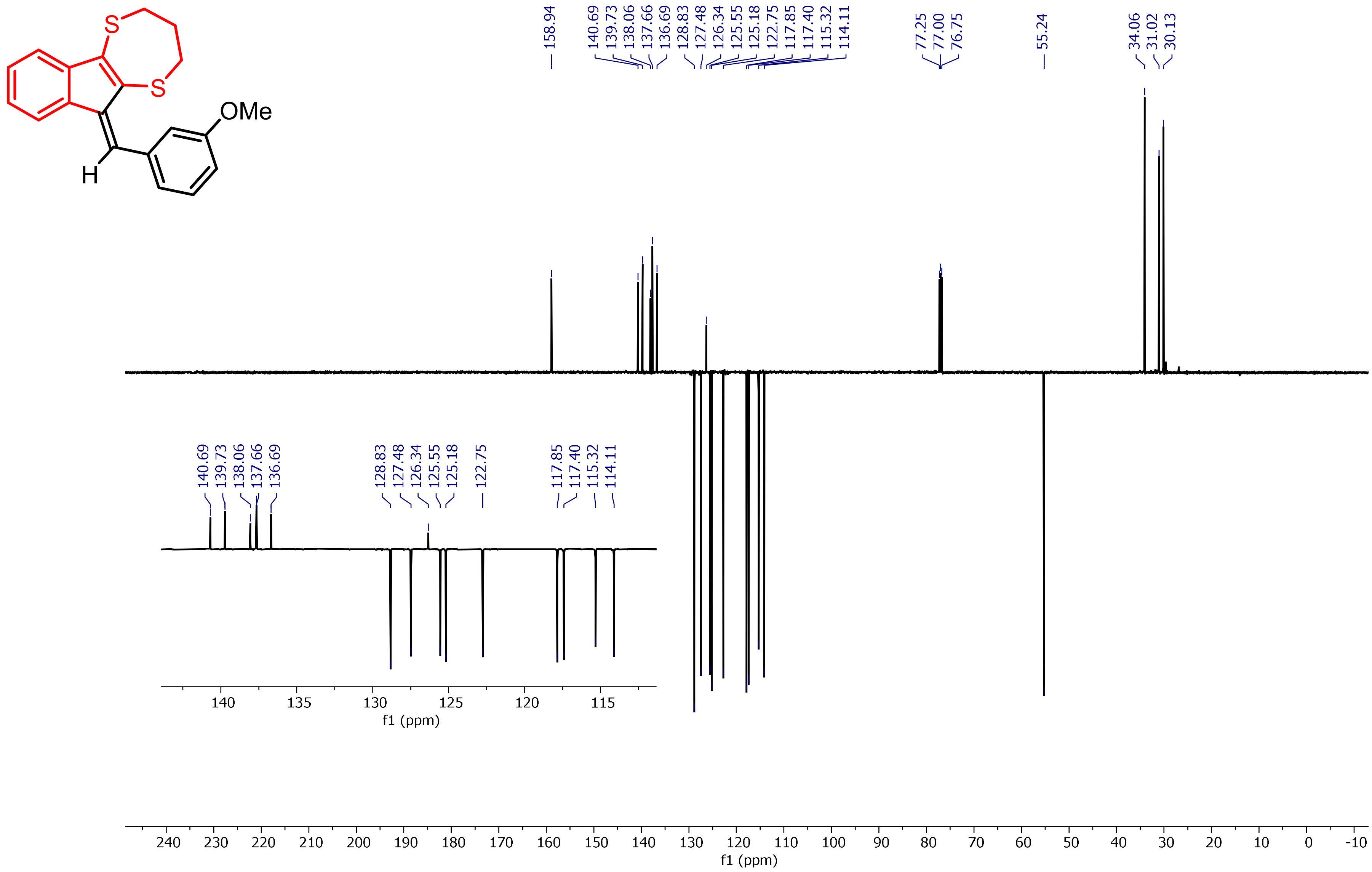

Figure S137. ^{1}H NMR ($CDCl_3$, 500 MHz) spectrum **2n**-(***E***)

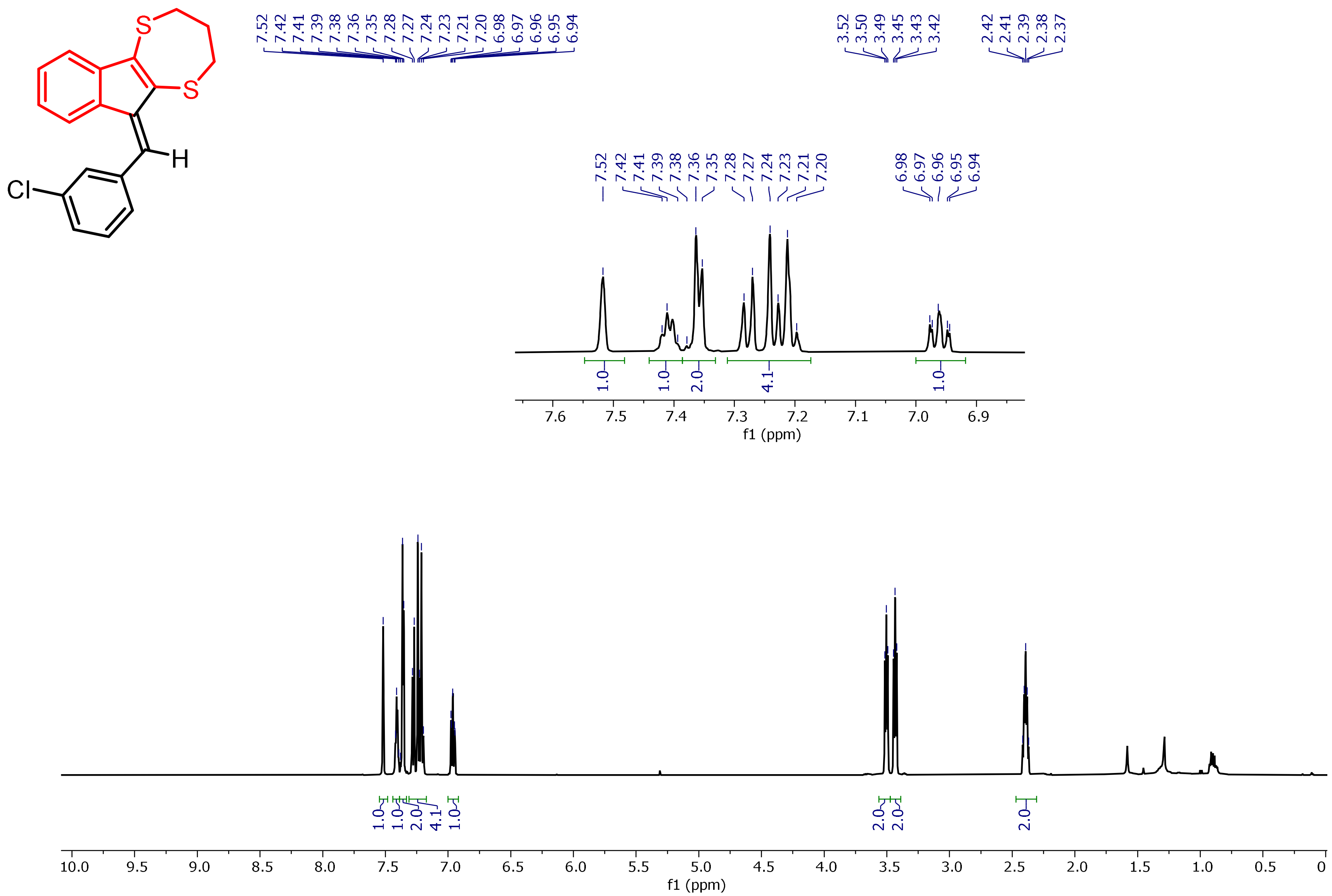

Figure S138. $^{13}C\{^1H\}$ NMR (126 MHz, $CDCl_3$, APT) spectrum **2n-(*E*)**

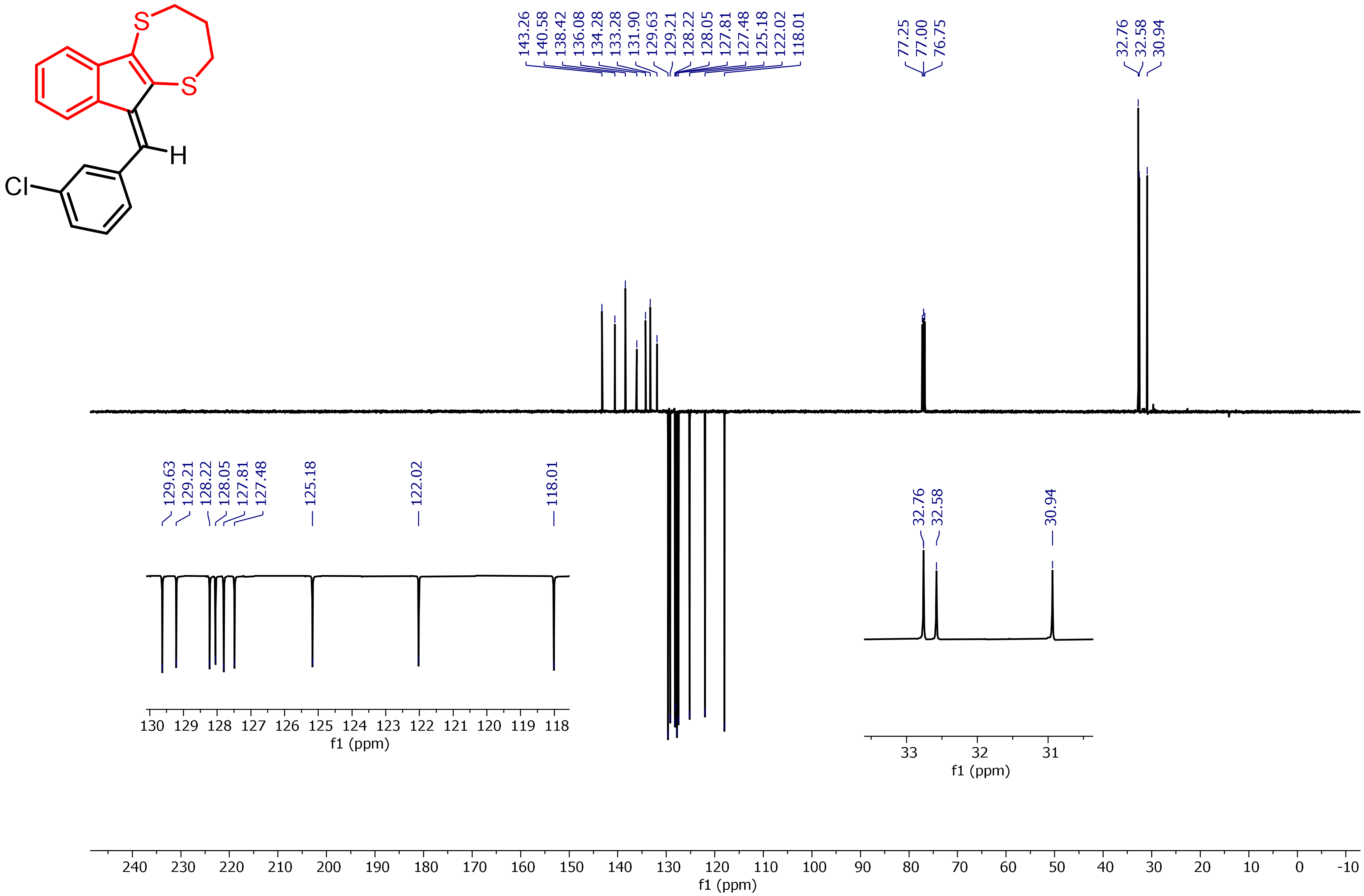

Figure S139. ^{1}H NMR ($CDCl_3$, 500 MHz) spectrum **2n**-(***Z***)

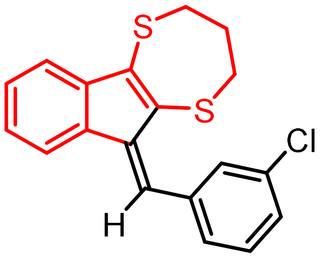

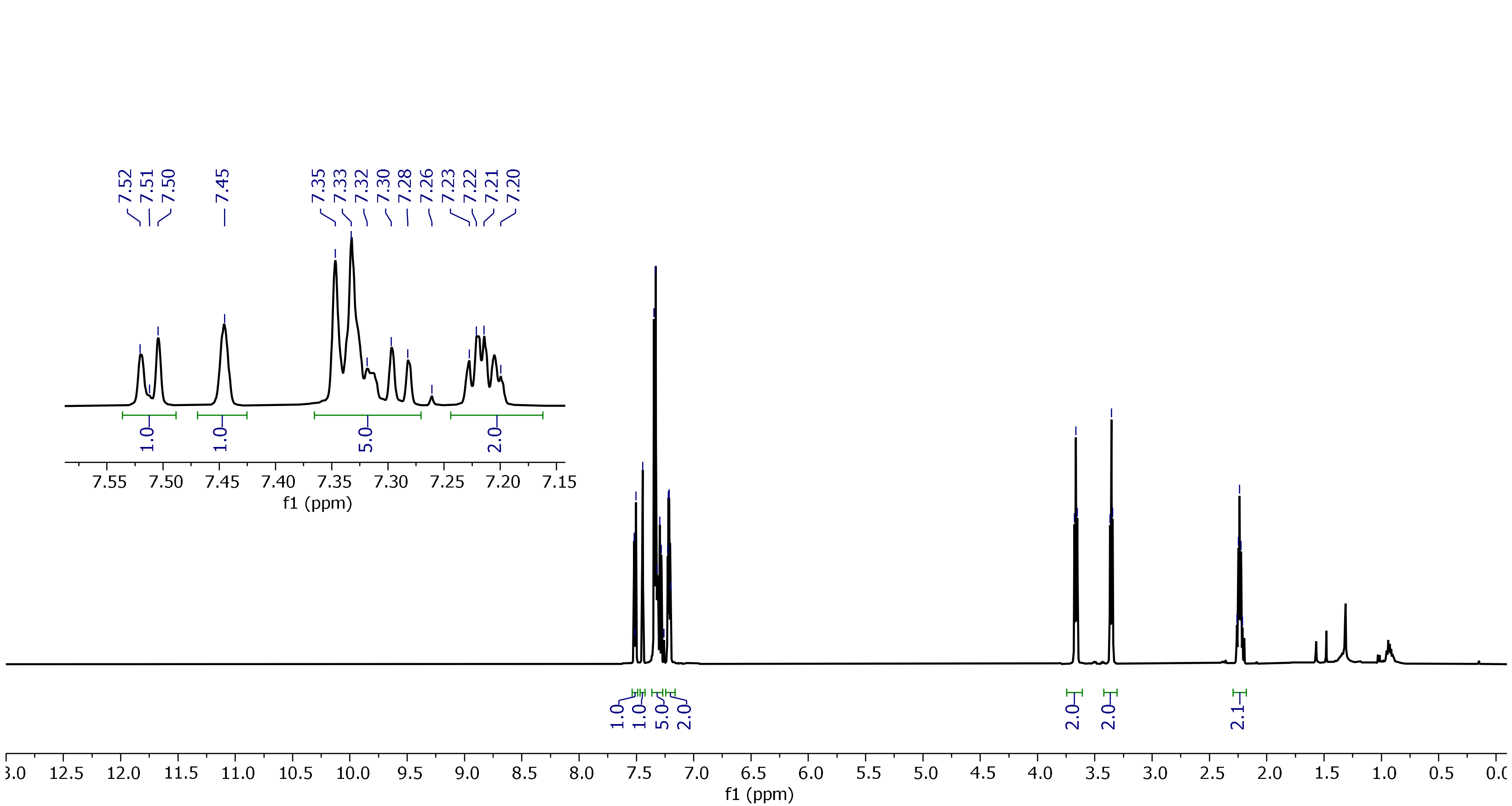

Figure S140. $^{13}C\{^{1}H\}$ NMR (126 MHz, $CDCl_3$, APT) spectrum **2n-(*Z*)**

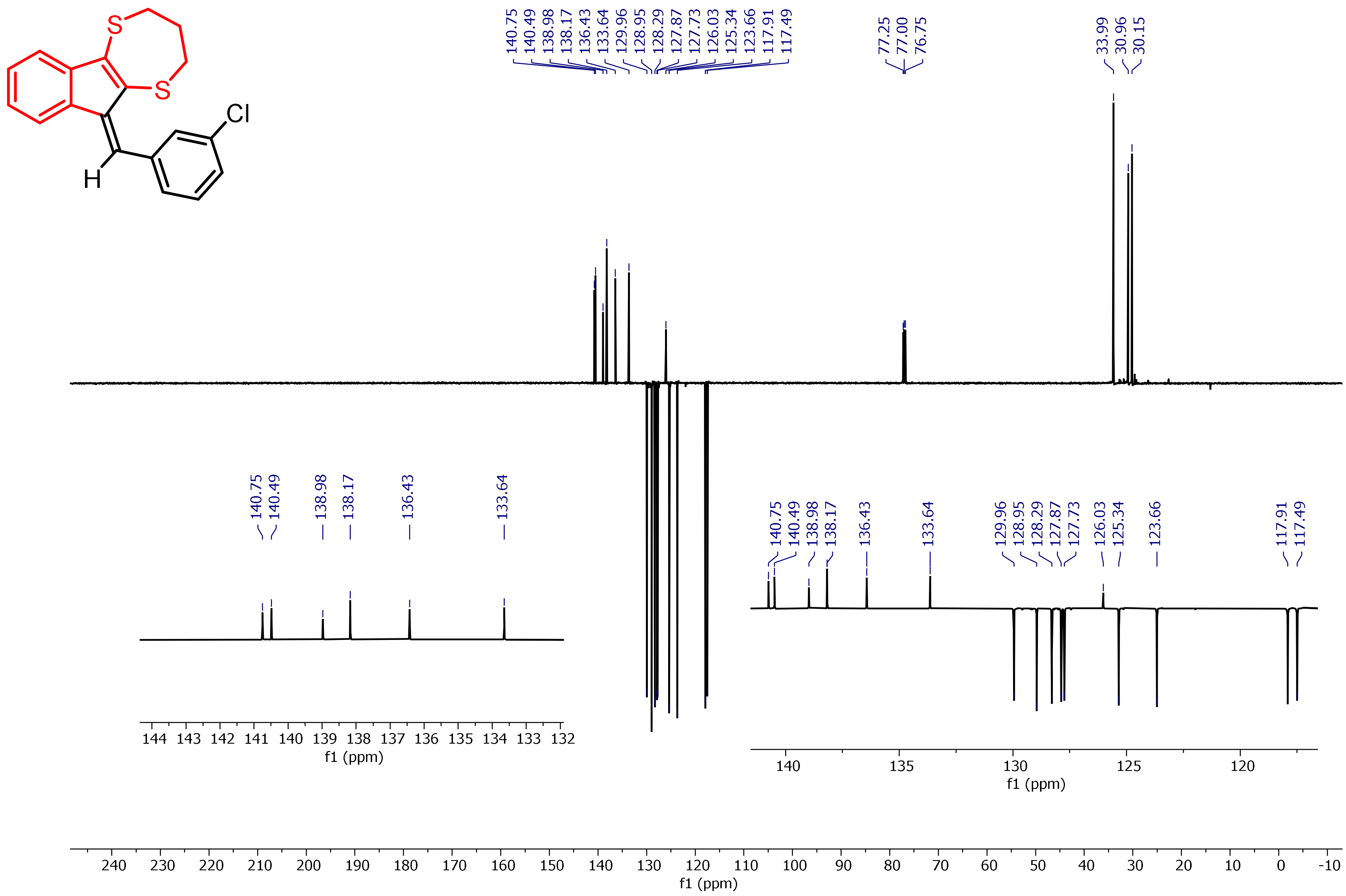

Figure S141. ^{1}H NMR ($CDCl_3$, 500 MHz) spectrum **2o**-(***E***)

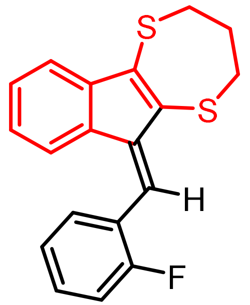

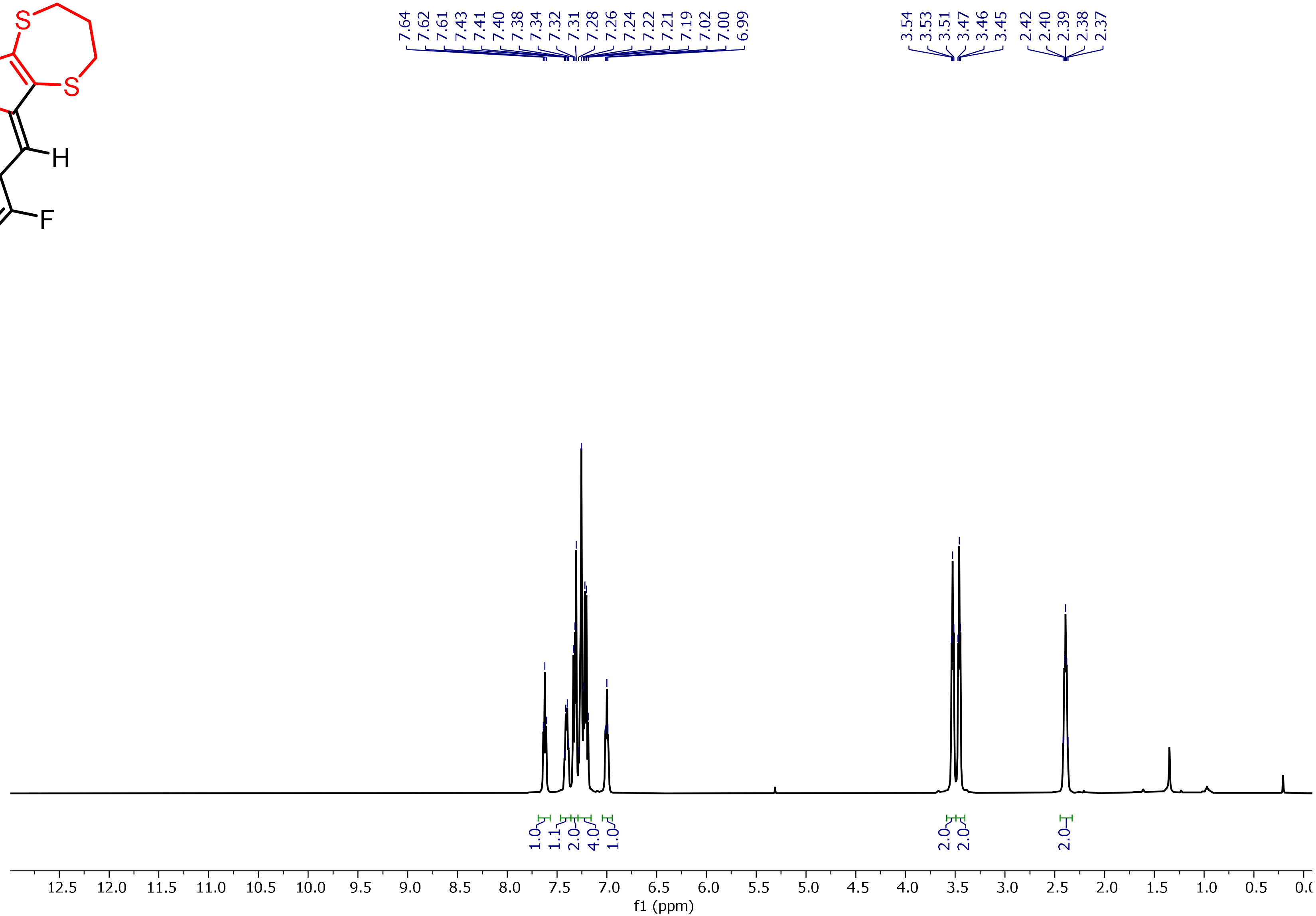

Figure S142. $^{13}C\{^1H\}$ NMR (126 MHz, $CDCl_3$, APT) spectrum **2o-(*E*)**

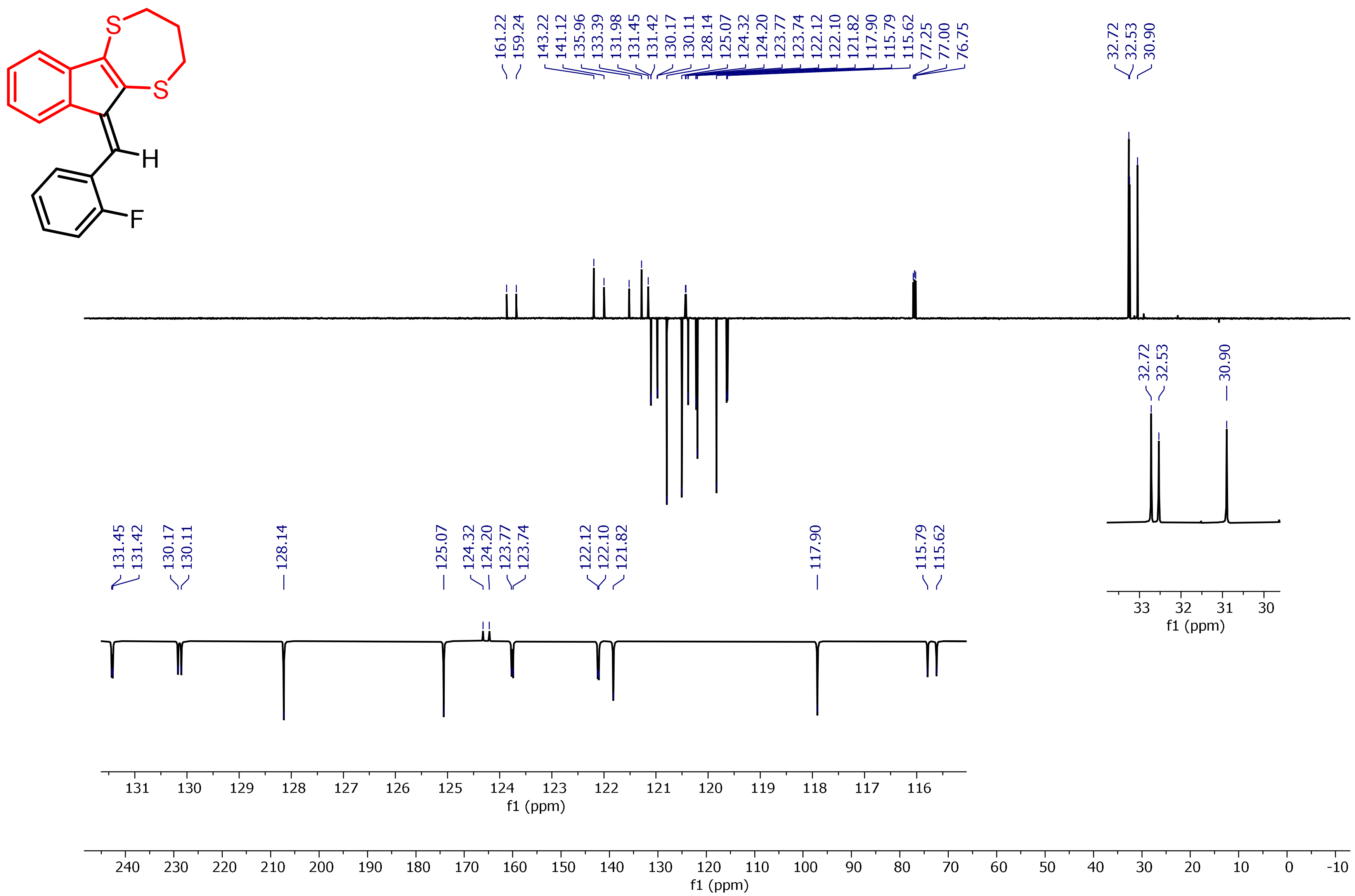

Figure S143. ^{1}H NMR ($CDCl_3$, 500 MHz) spectrum **2o**-(***Z***)

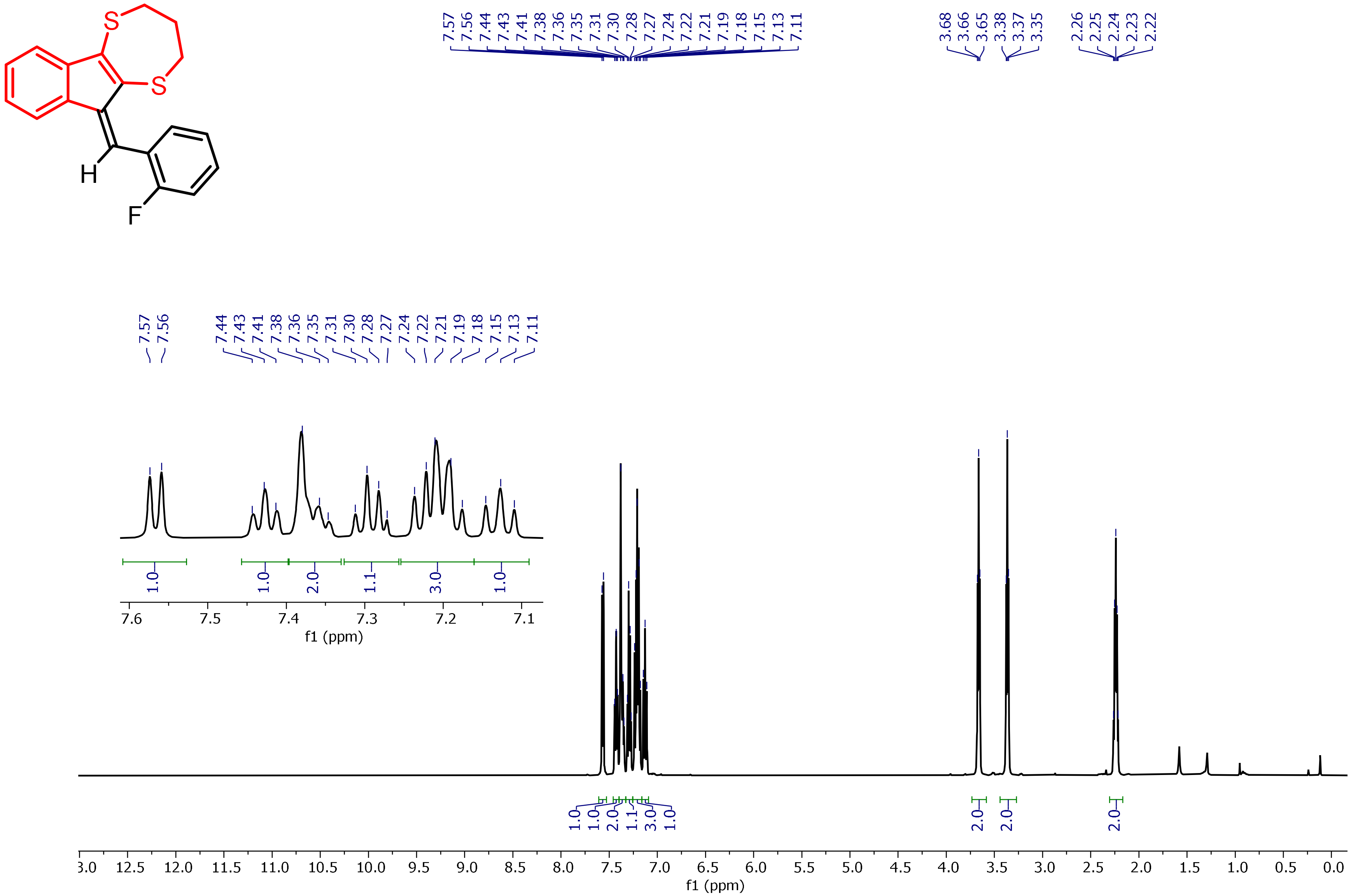

Figure S144. $^{13}C\{^1H\}$ NMR (126 MHz, $CDCl_3$, APT) spectrum **2o-(*Z*)**

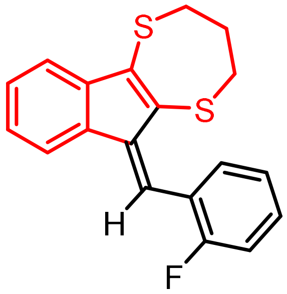

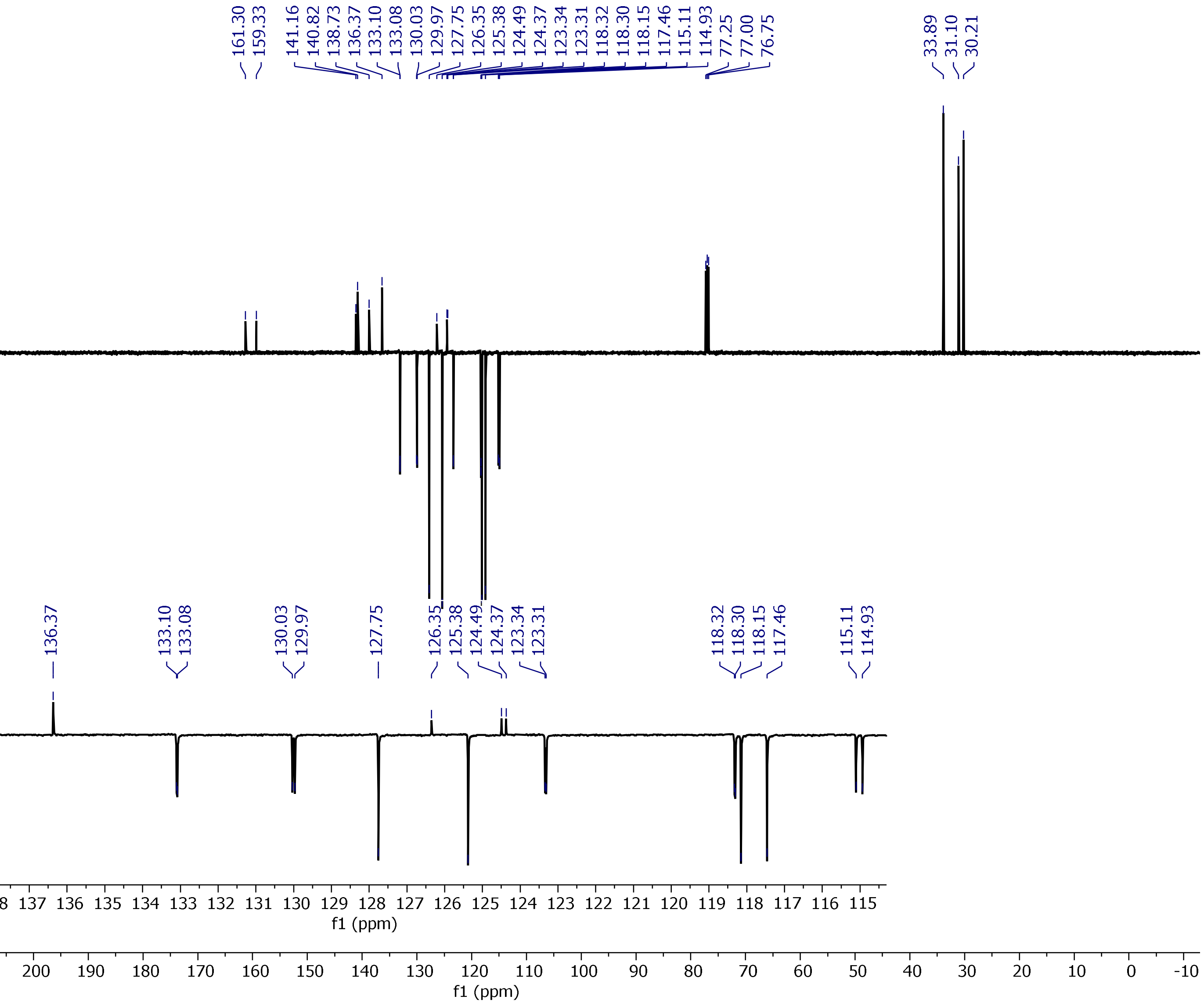

Figure S145. ^{1}H NMR ($CDCl_3$, 500 MHz) spectrum **2p**-(***E***)

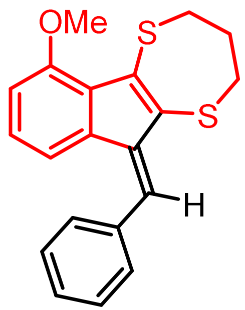

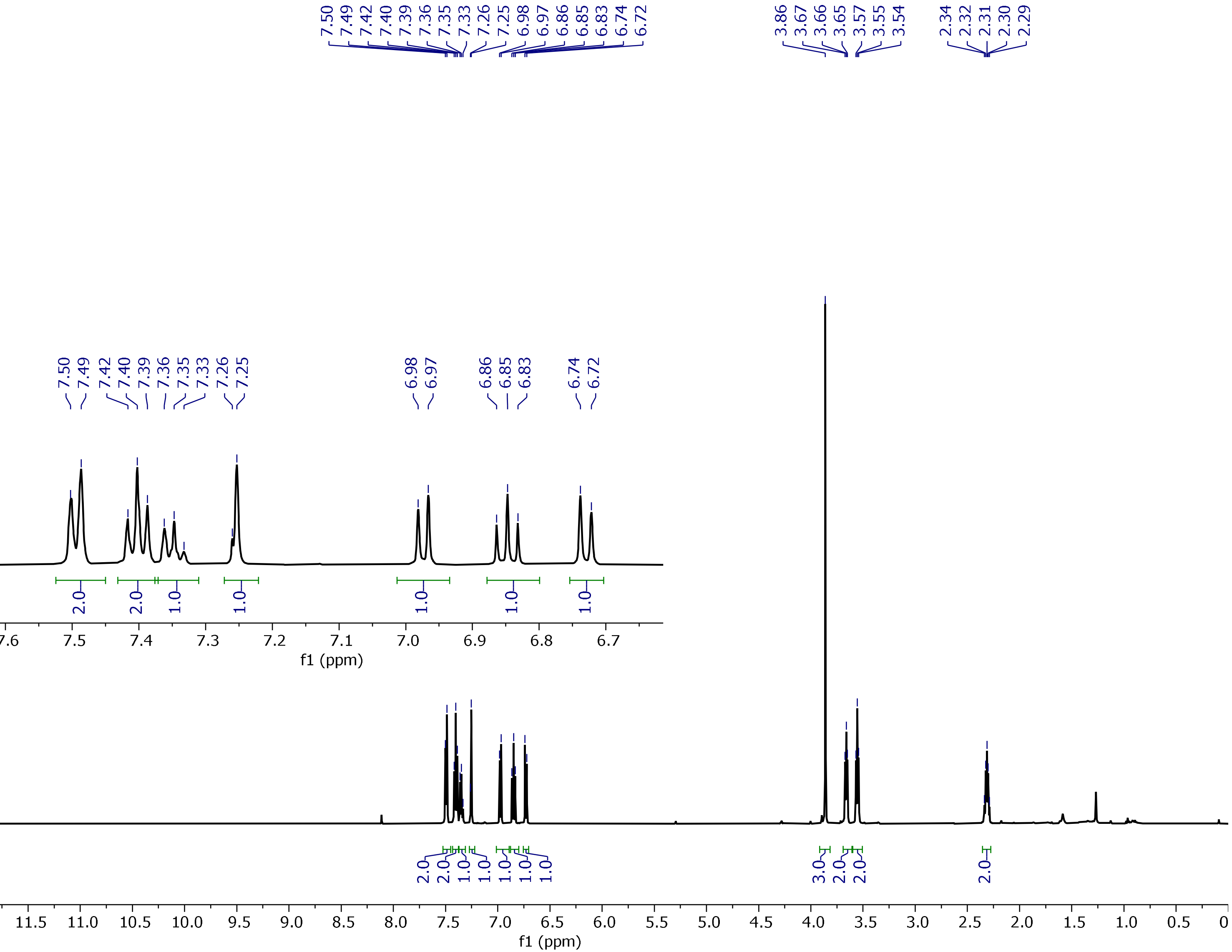

Figure S146. $^{13}C\{^1H\}$ NMR (126 MHz, $CDCl_3$, APT) spectrum **2p-(*E*)**

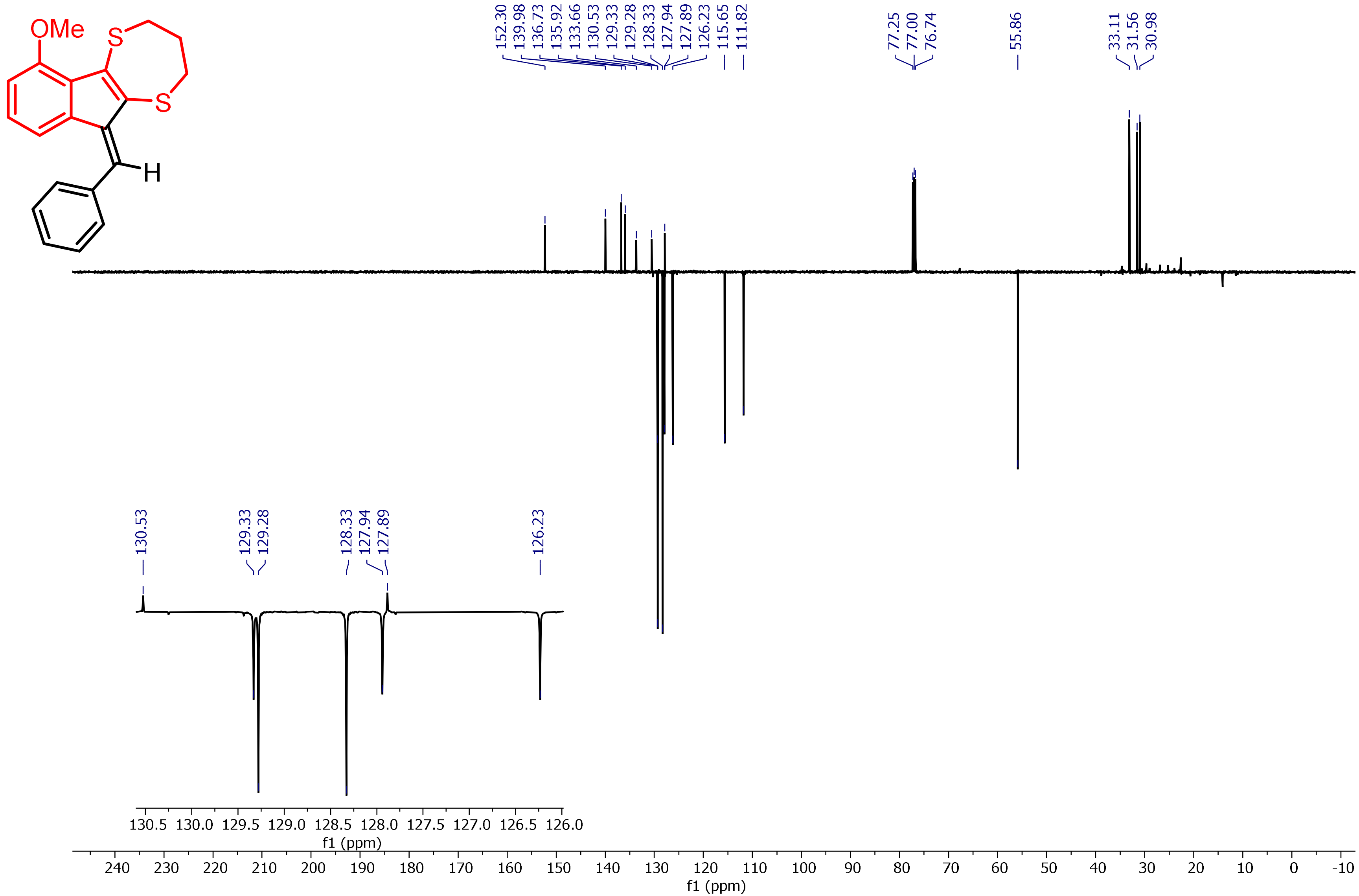

Figure S147. ^{1}H NMR ($CDCl_3$, 500 MHz) spectrum **2p**-(***Z***)

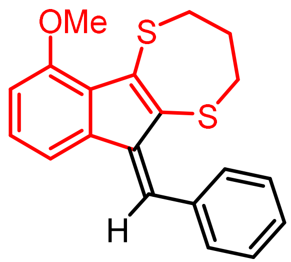

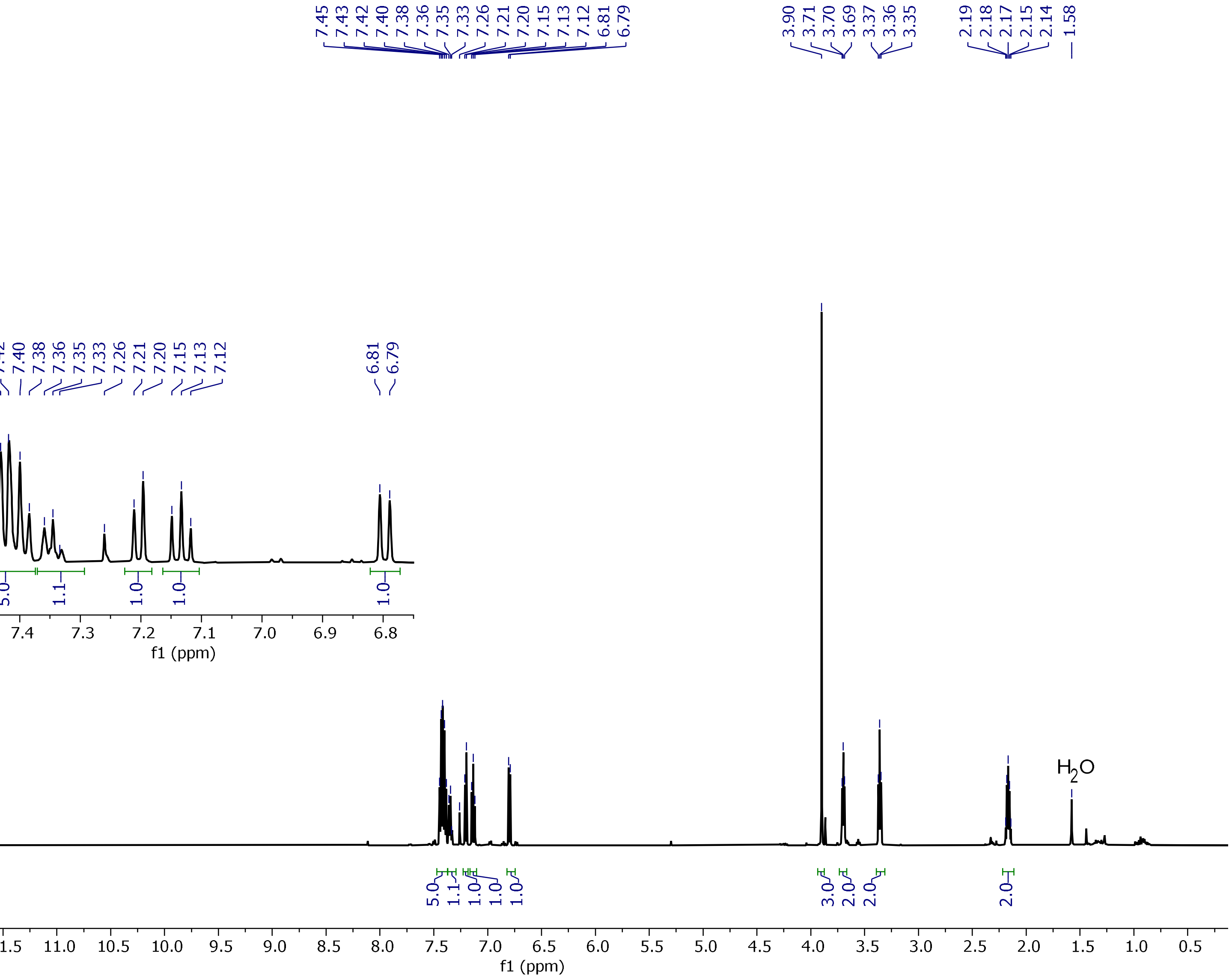

Figure S148. $^{13}C\{^1H\}$ NMR (126 MHz, $CDCl_3$, APT) spectrum **2p-(*Z*)**

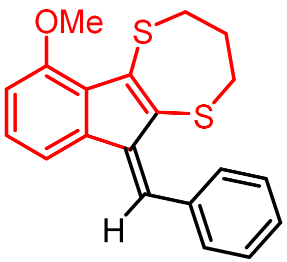
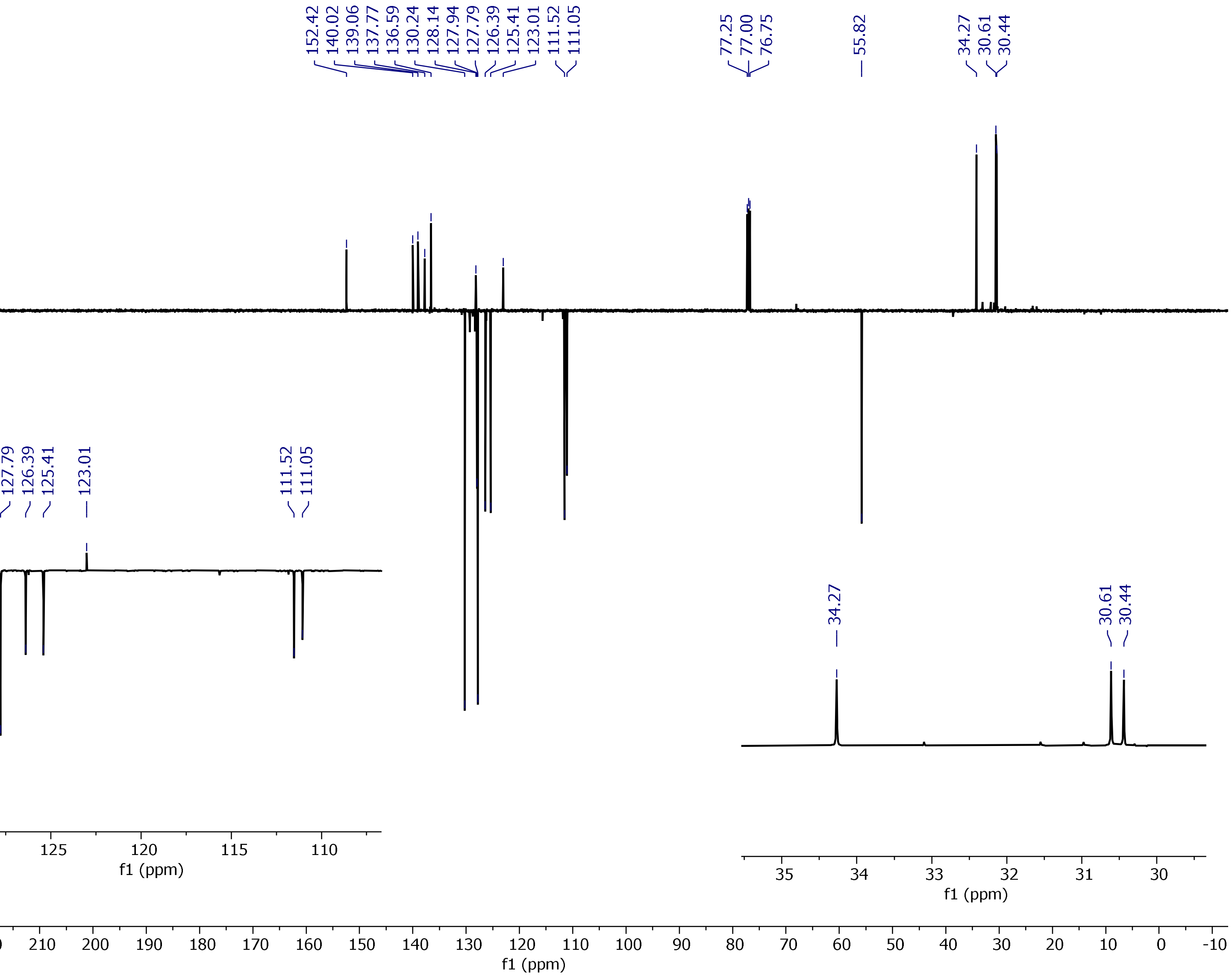

Figure S149. ^{1}H NMR ($CDCl_3$, 500 MHz) spectrum **2p-IV**

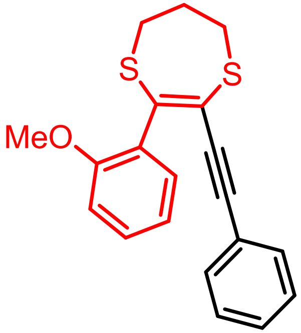

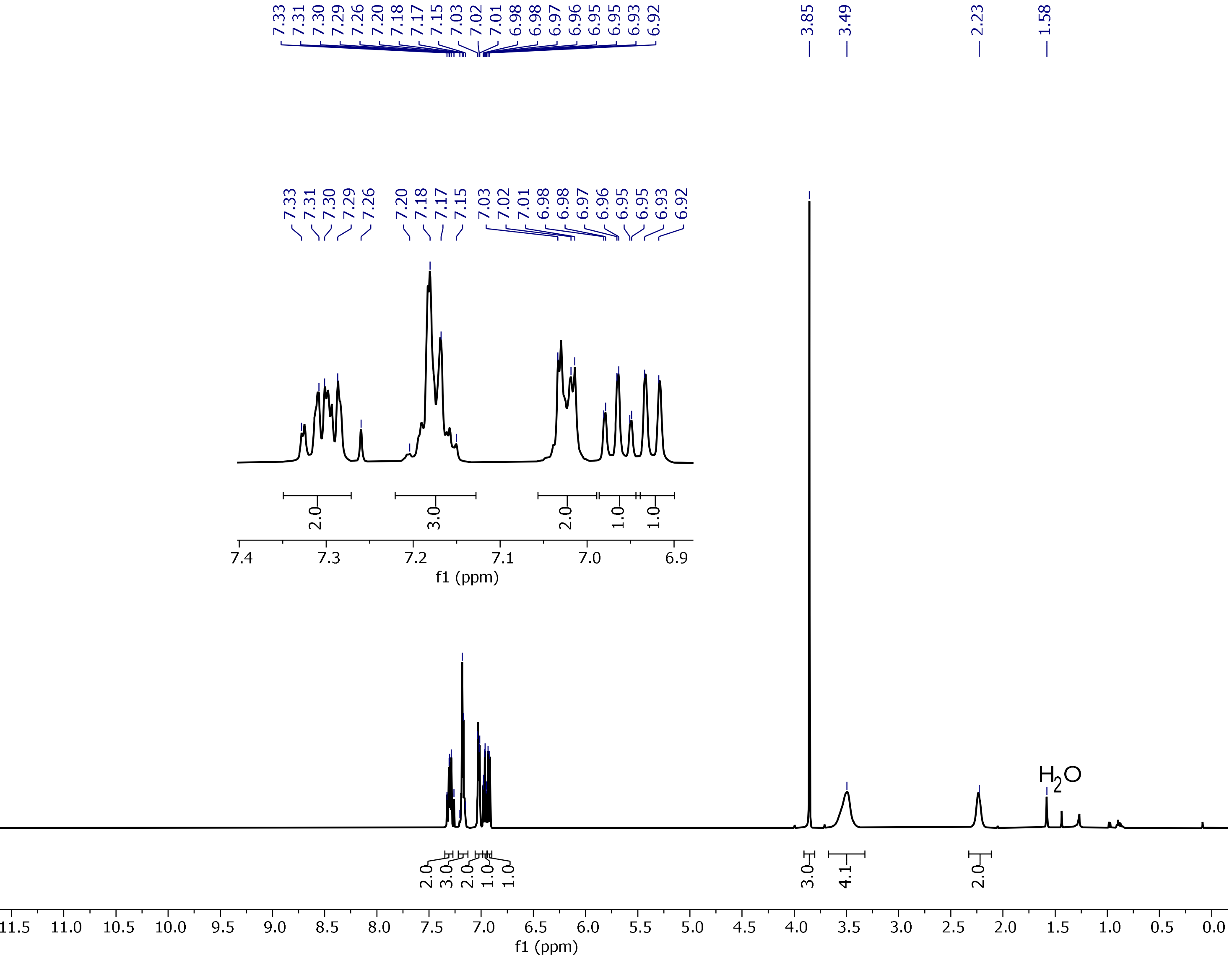

Figure S150. $^{13}C\{^{1}H\}$ NMR (126 MHz, $CDCl_3$, APT) spectrum **2p-IV**

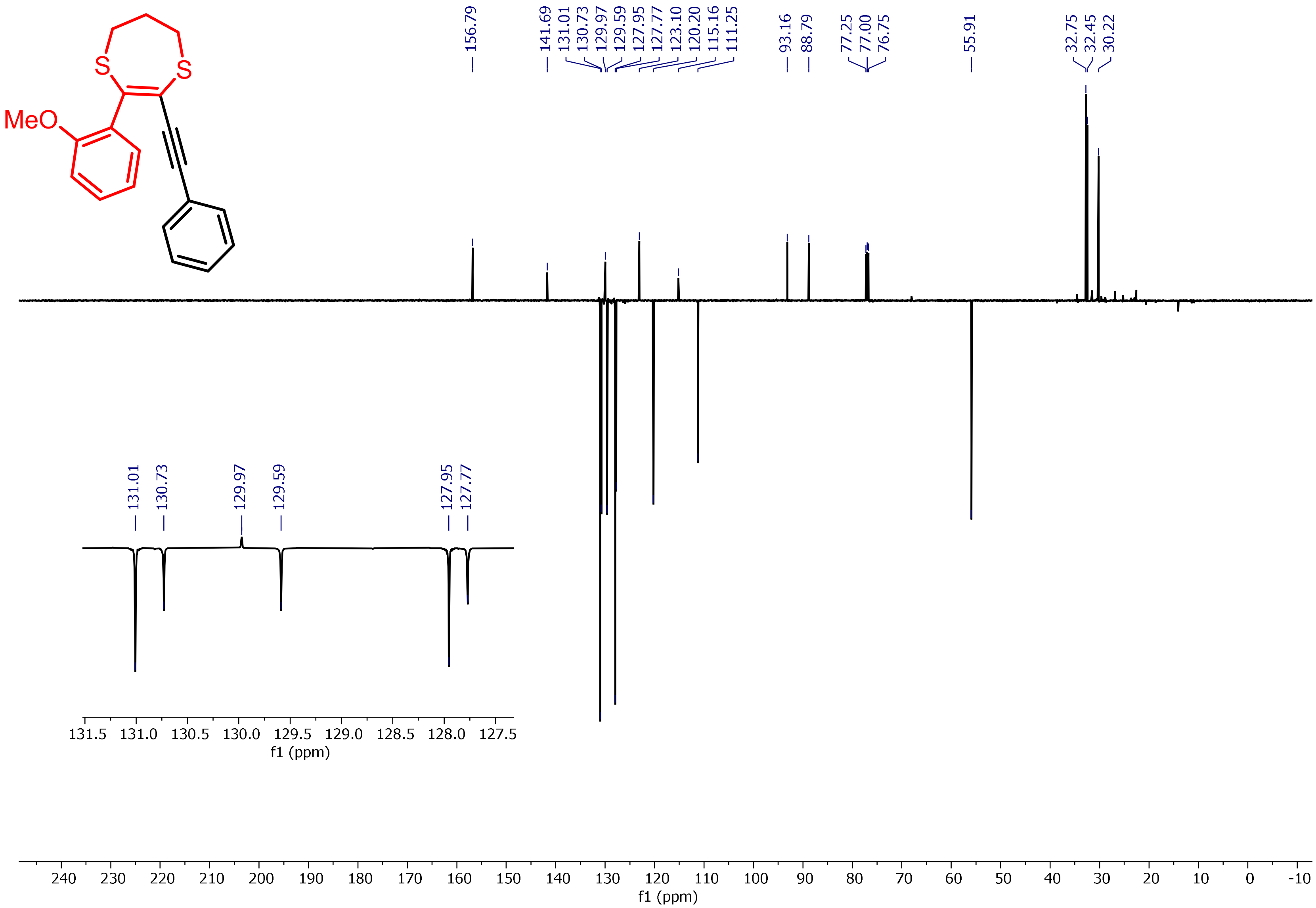

Figure S151. ^{1}H NMR ($CDCl_3$, 500 MHz) spectrum **2r**-(***Z***)

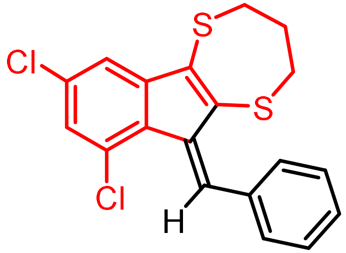

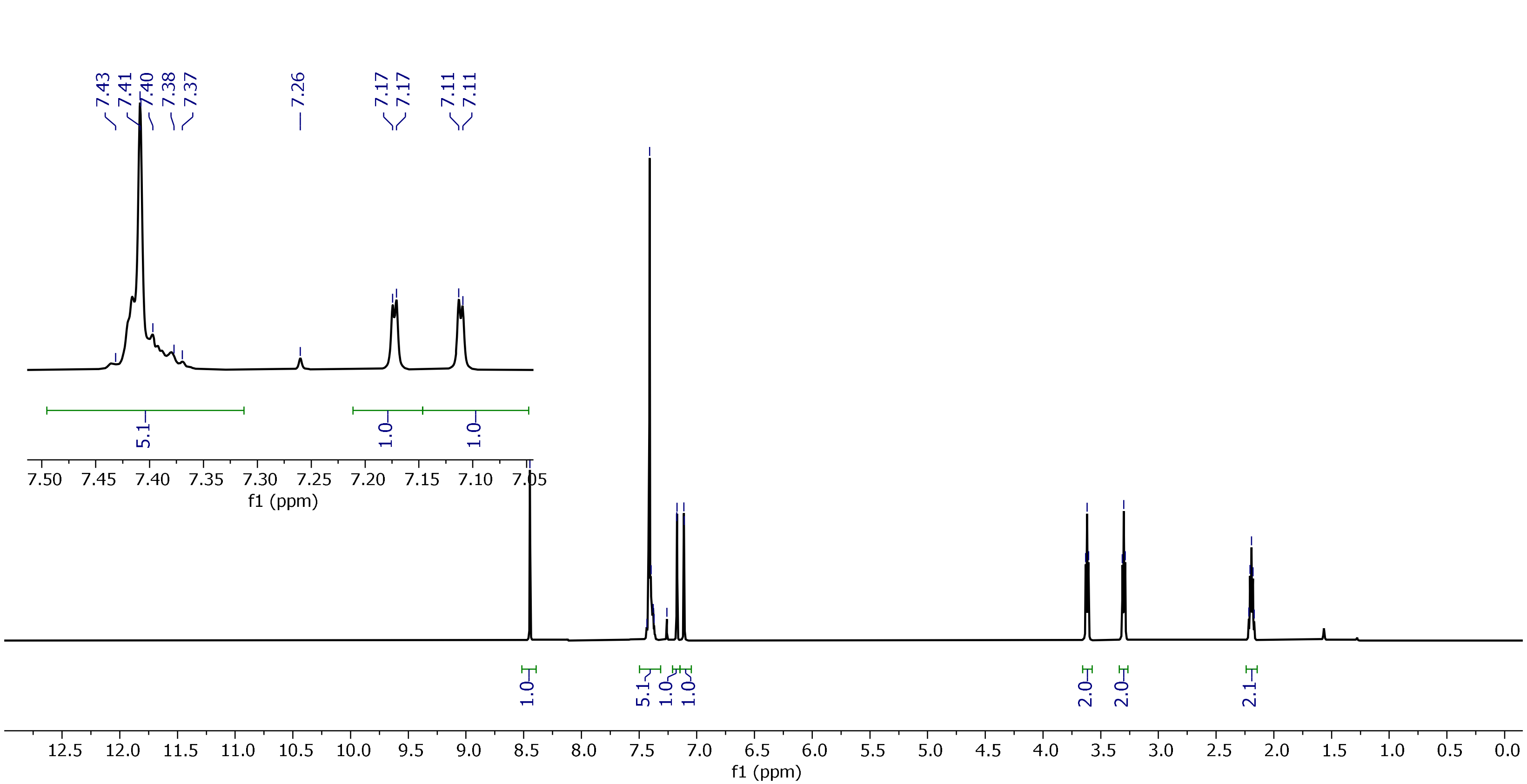

Figure S152. $^{13}C\{^1H\}$ NMR (126 MHz, $CDCl_3$, APT) spectrum **2r-(*Z*)**

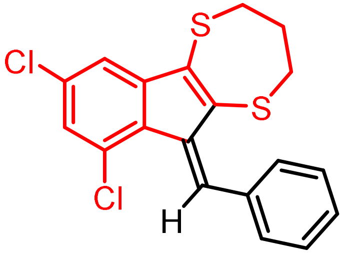

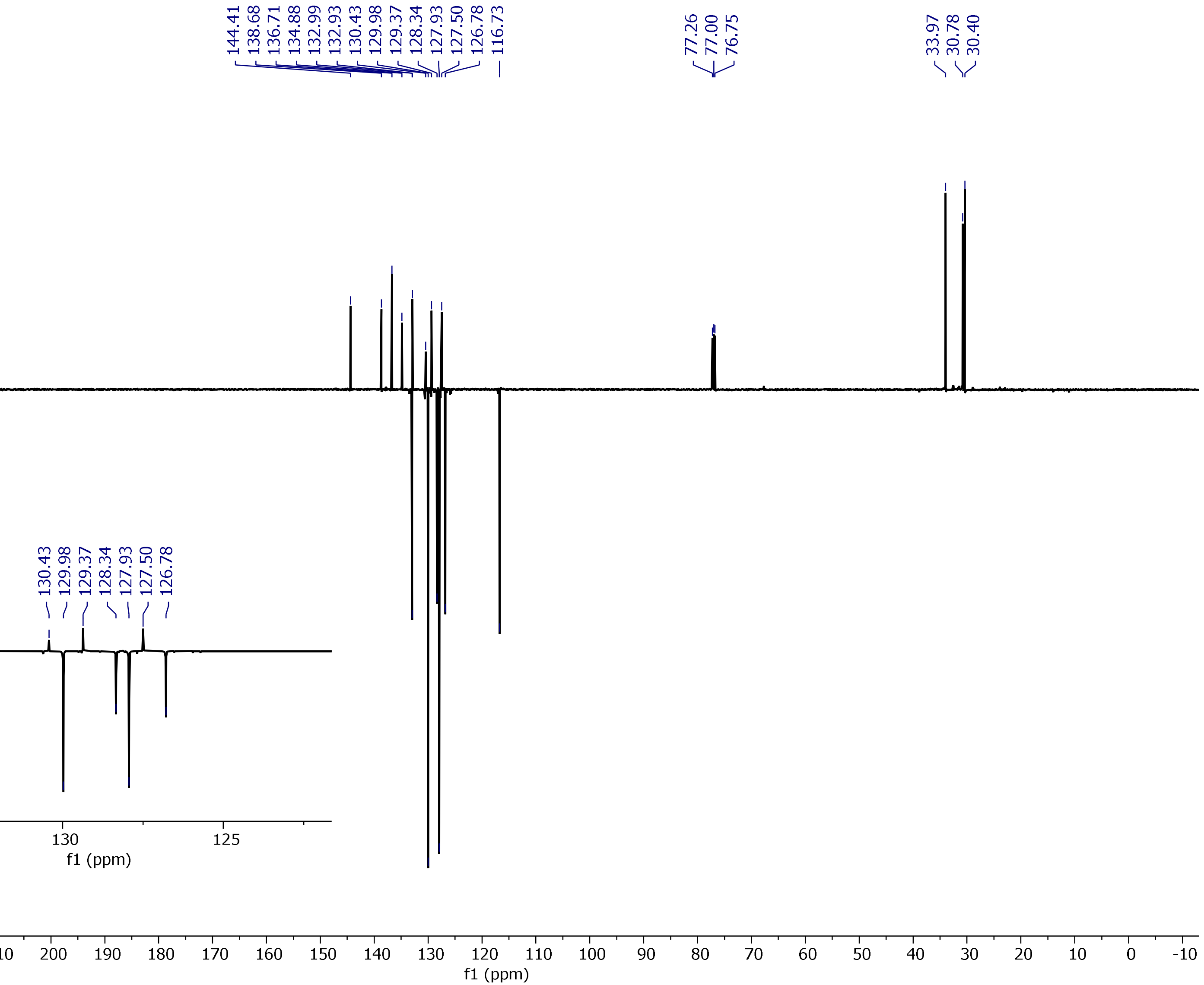

Figure S153. ^{1}H NMR ($CDCl_3$, 500 MHz) spectrum **2q**-(***E***)

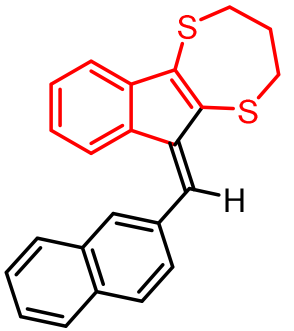

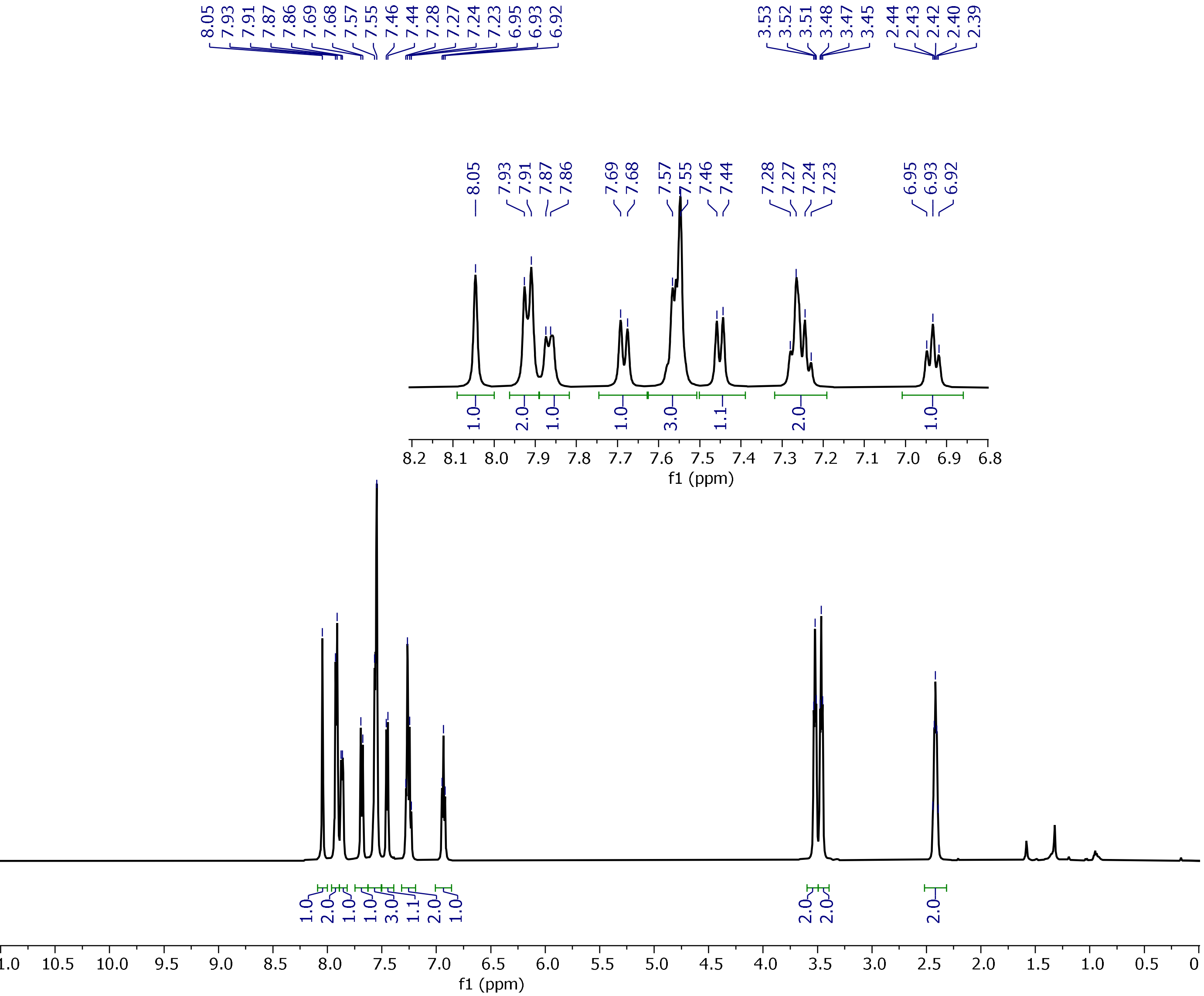

Figure S154. $^{13}C\{^{1}H\}$ NMR (126 MHz, $CDCl_3$, APT) spectrum **2q-(*E*)**

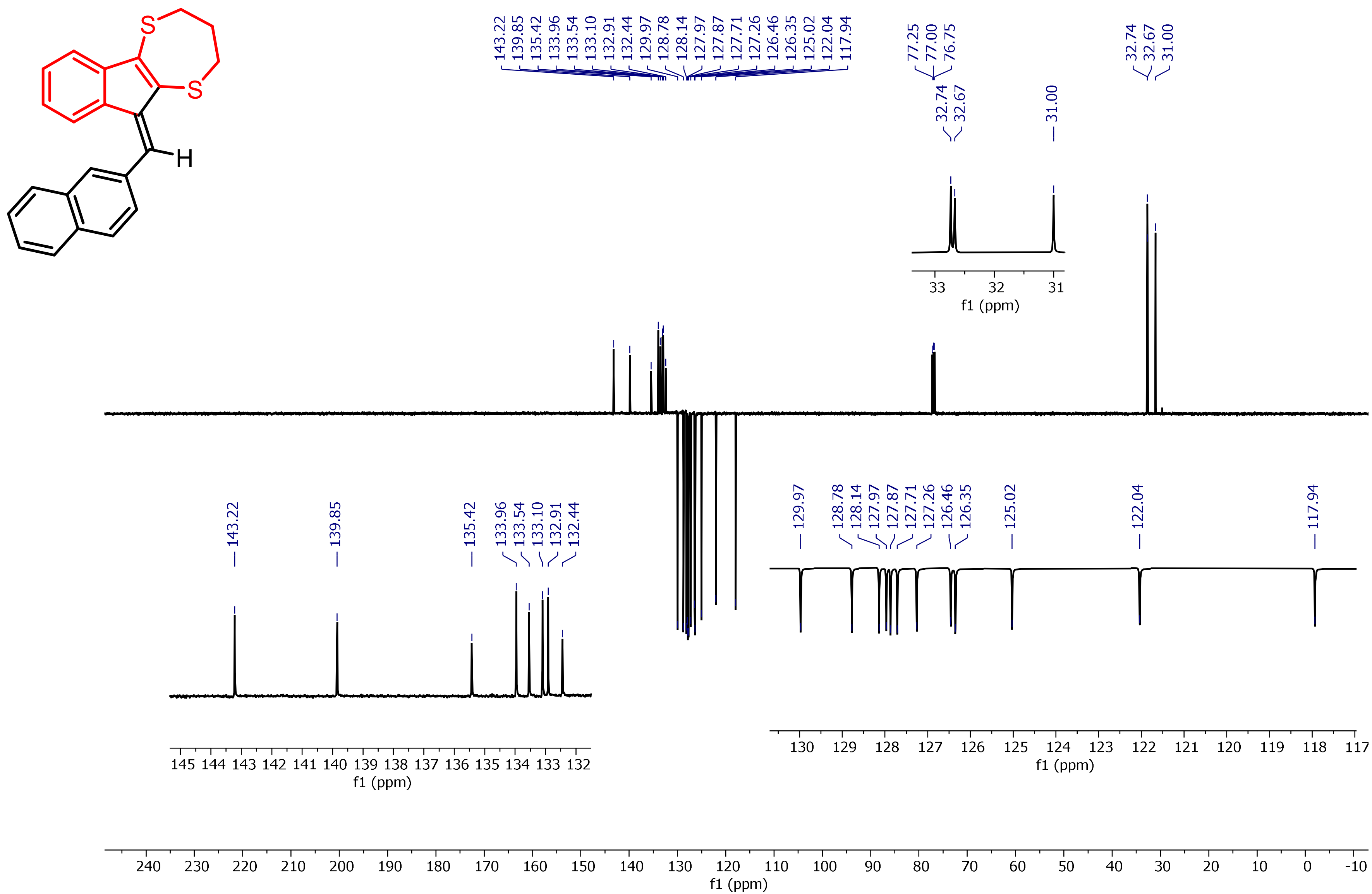

Figure S155. ^{1}H NMR ($CDCl_3$, 500 MHz) spectrum **2q**-(***Z***)

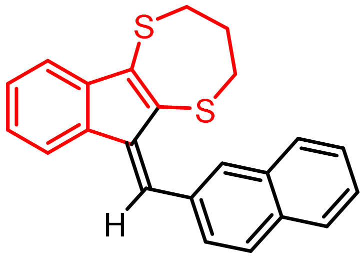

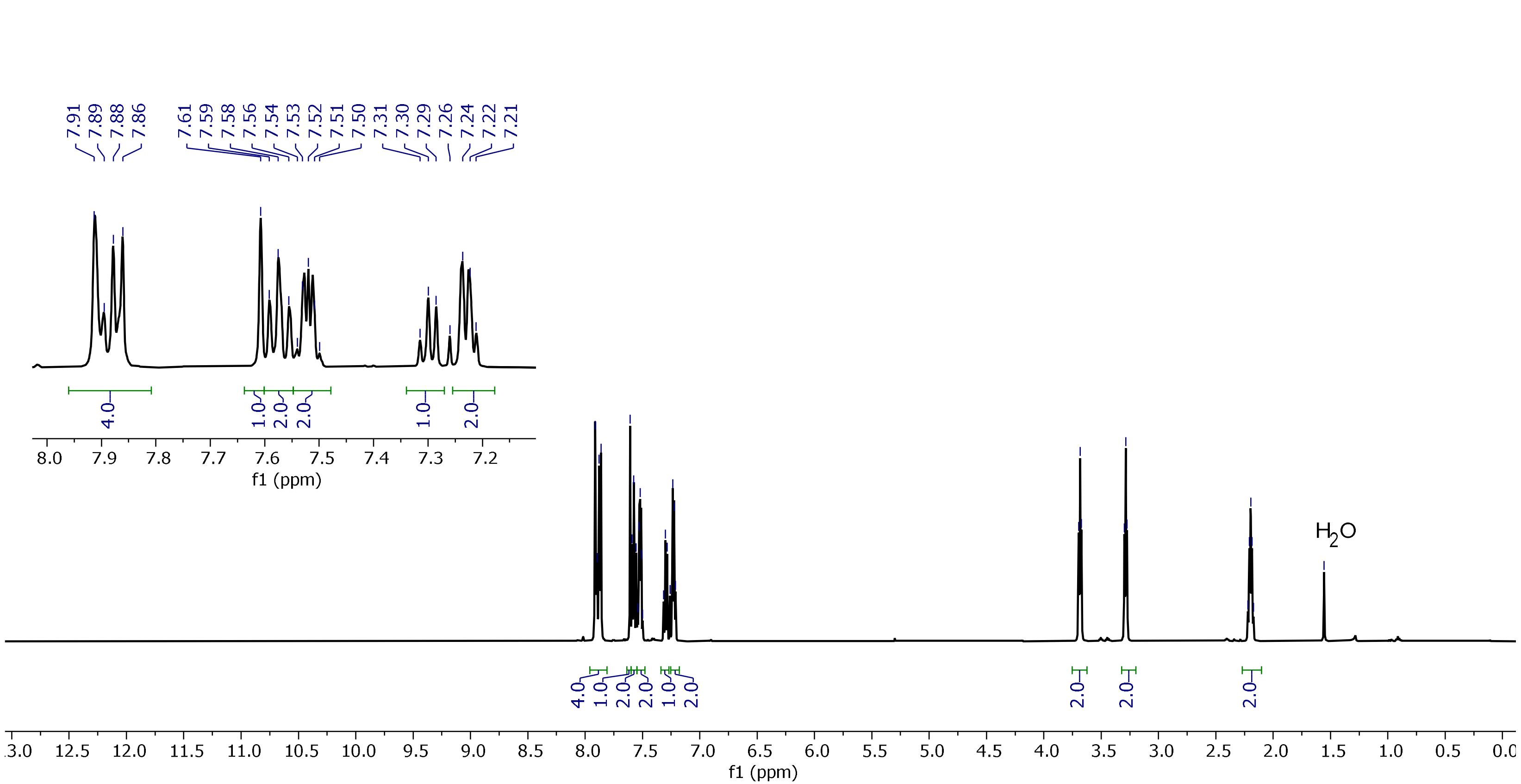

Figure S156. $^{13}C\{^{1}H\}$ NMR (126 MHz, $CDCl_3$, APT) spectrum **2q-(*Z*)**

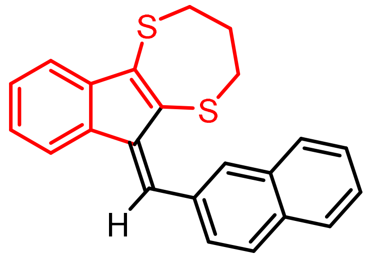

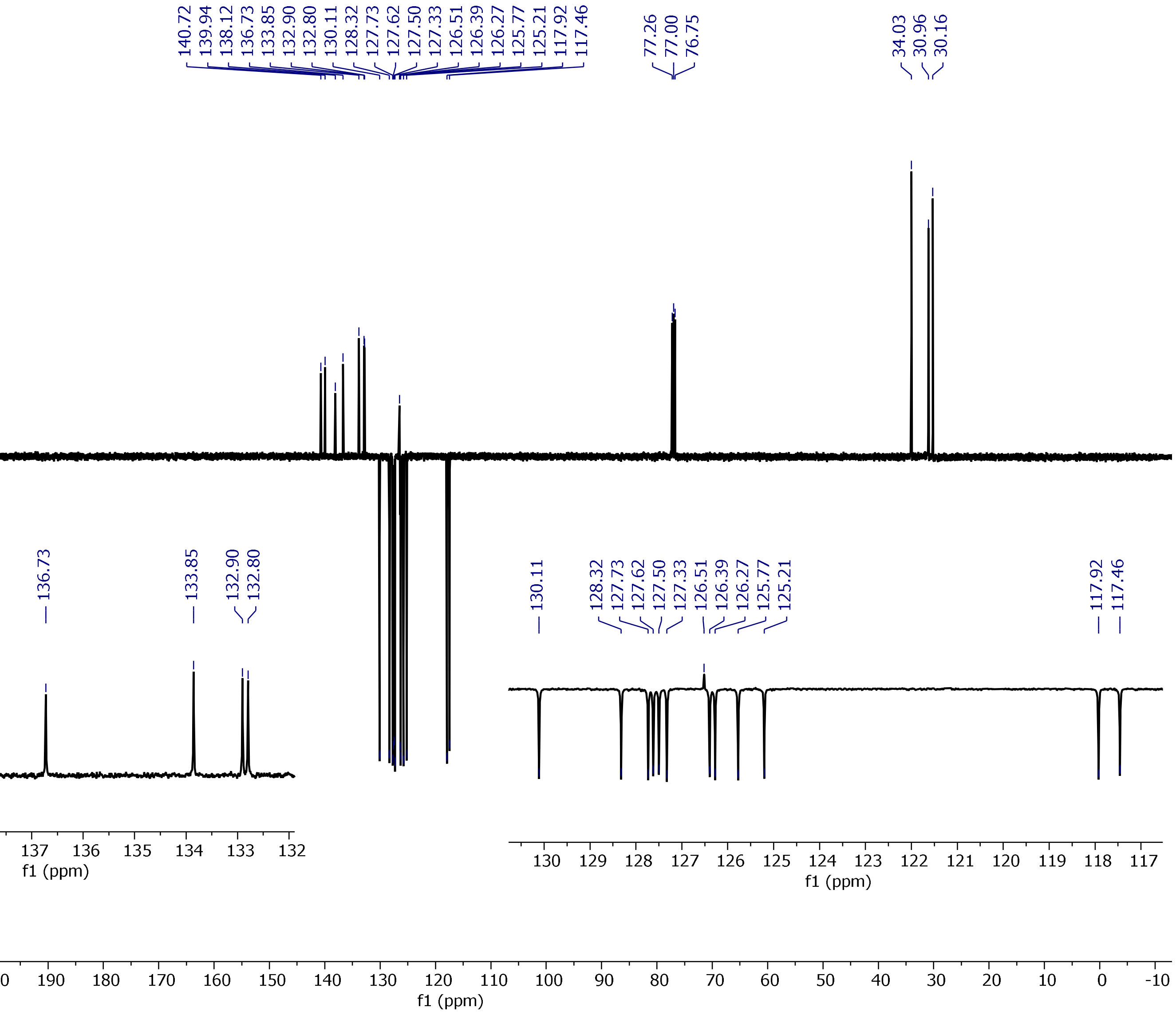

Figure S157. ^{1}H NMR ($CDCl_3$, 500 MHz) spectrum **2s**-(***E***)

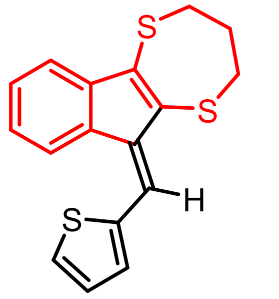

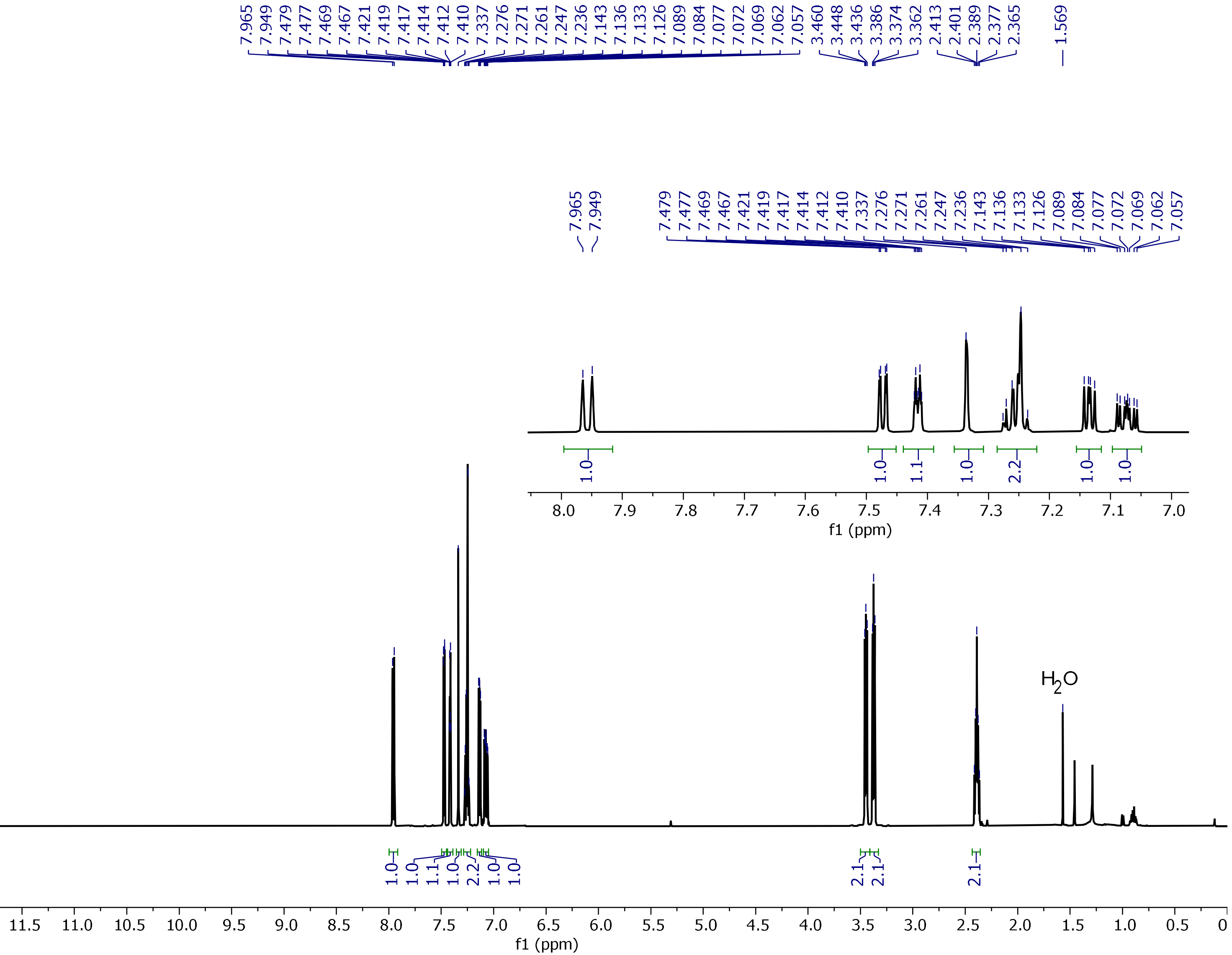

Figure S158. $^{13}C\{^1H\}$ NMR (126 MHz, $CDCl_3$, APT) spectrum **2s-(*E*)**

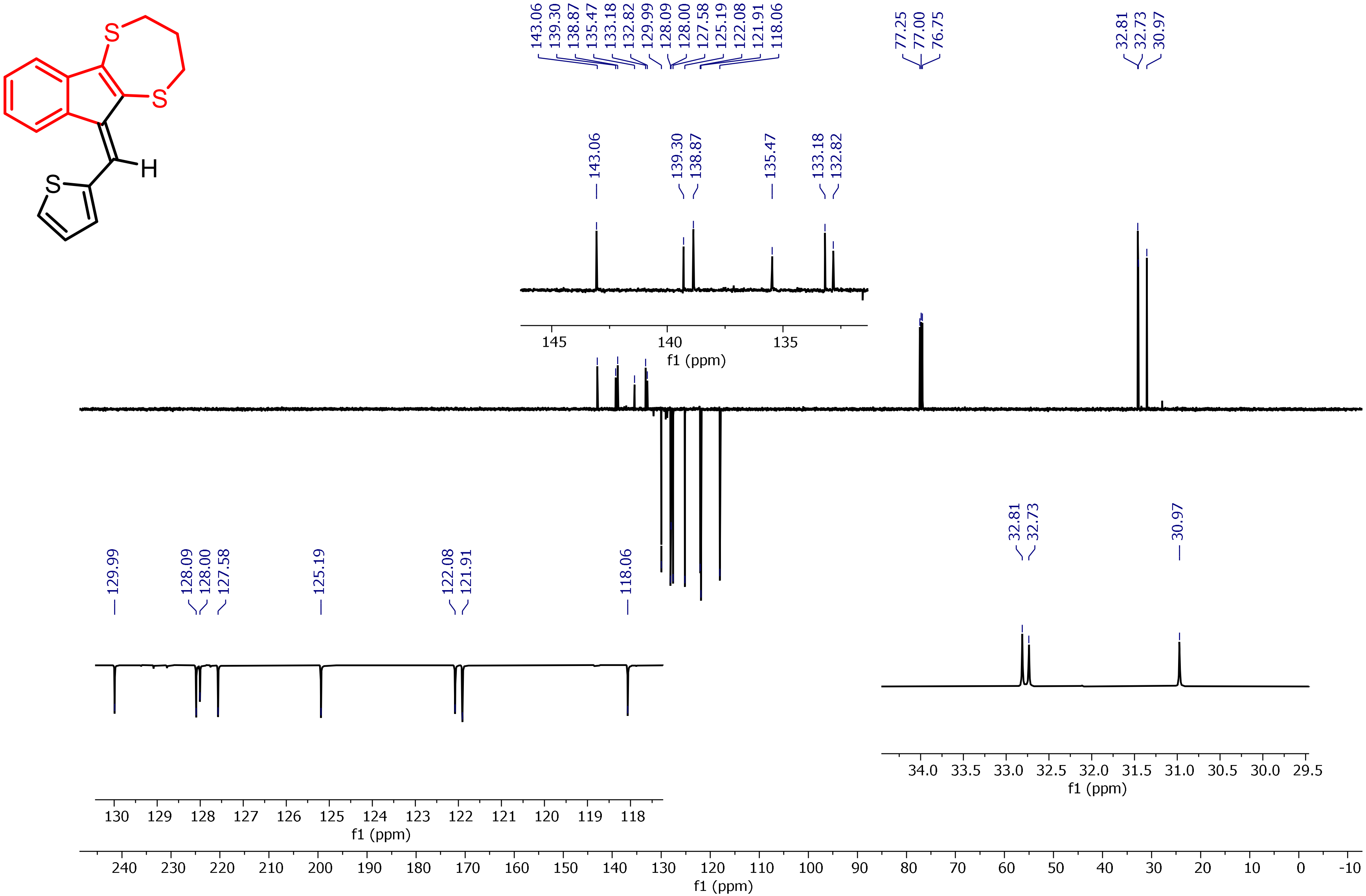

Figure S159. ^{1}H NMR ($CDCl_3$, 500 MHz) spectrum **2t**

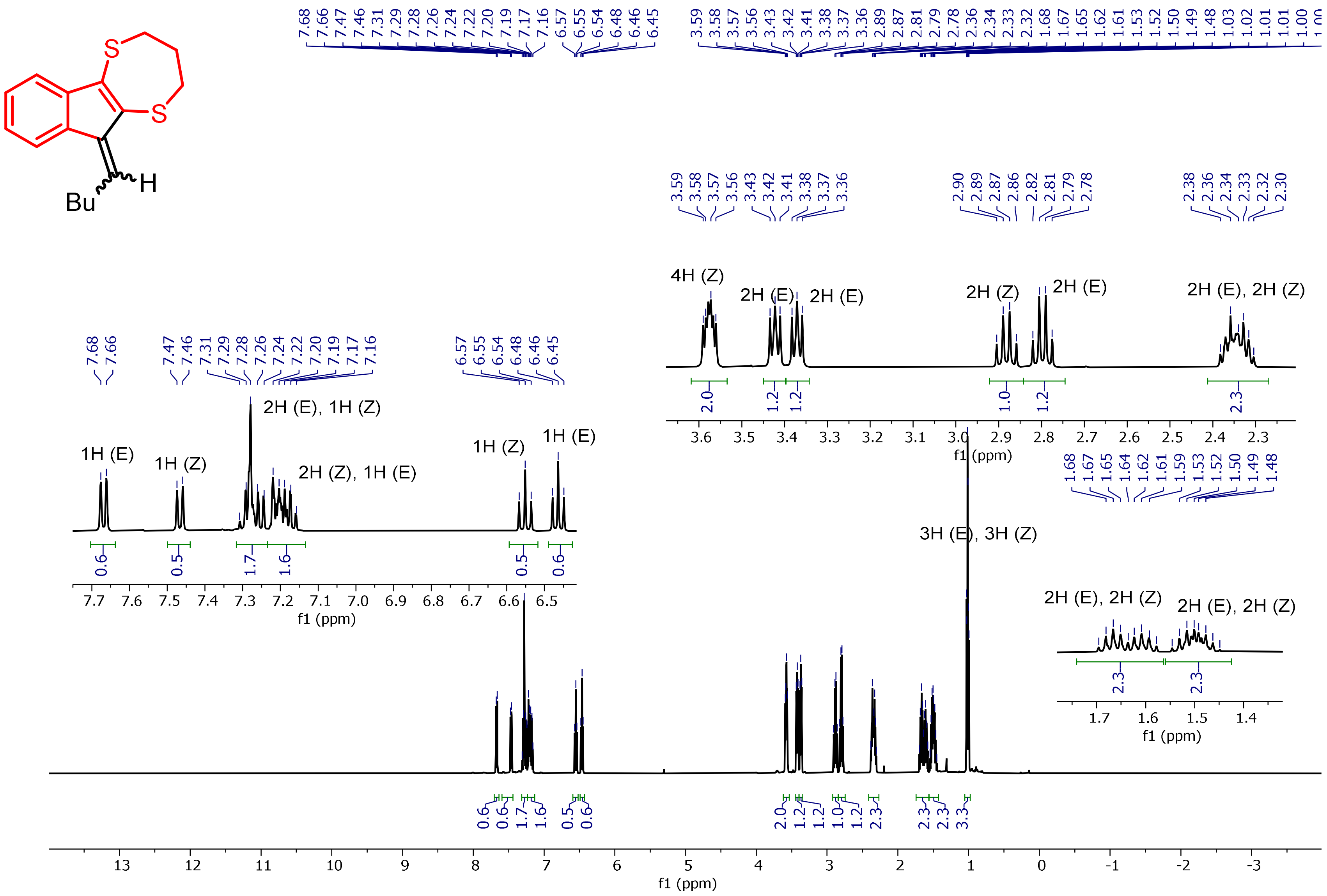

Figure S160. $^{13}C\{^1H\}$ NMR (126 MHz, $CDCl_3$, APT) spectrum **2t**

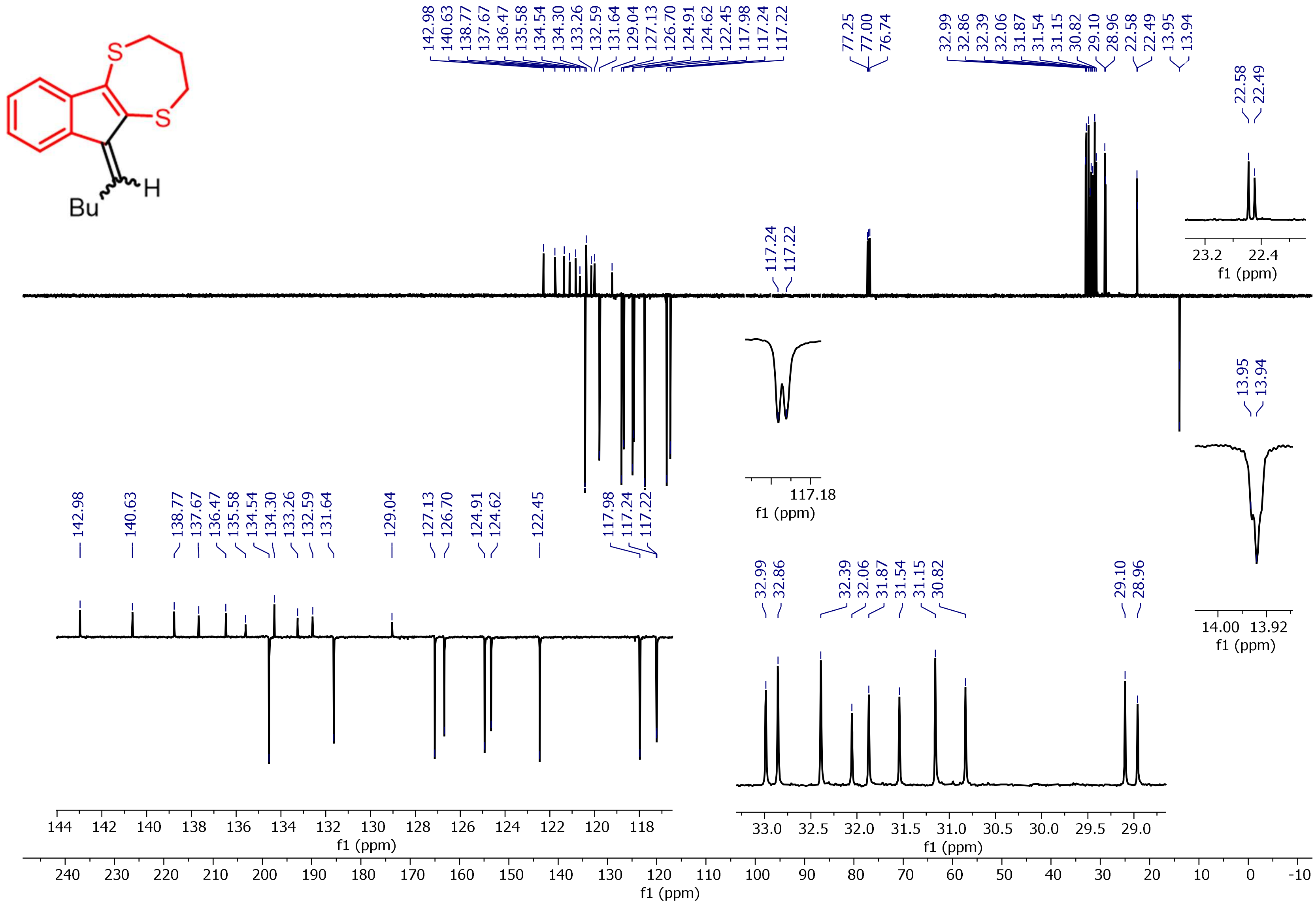

Figure S161. ^{1}H NMR ($CDCl_3$, 500 MHz) spectrum **4a**-(***E***)

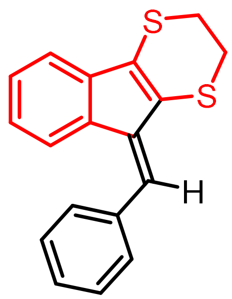

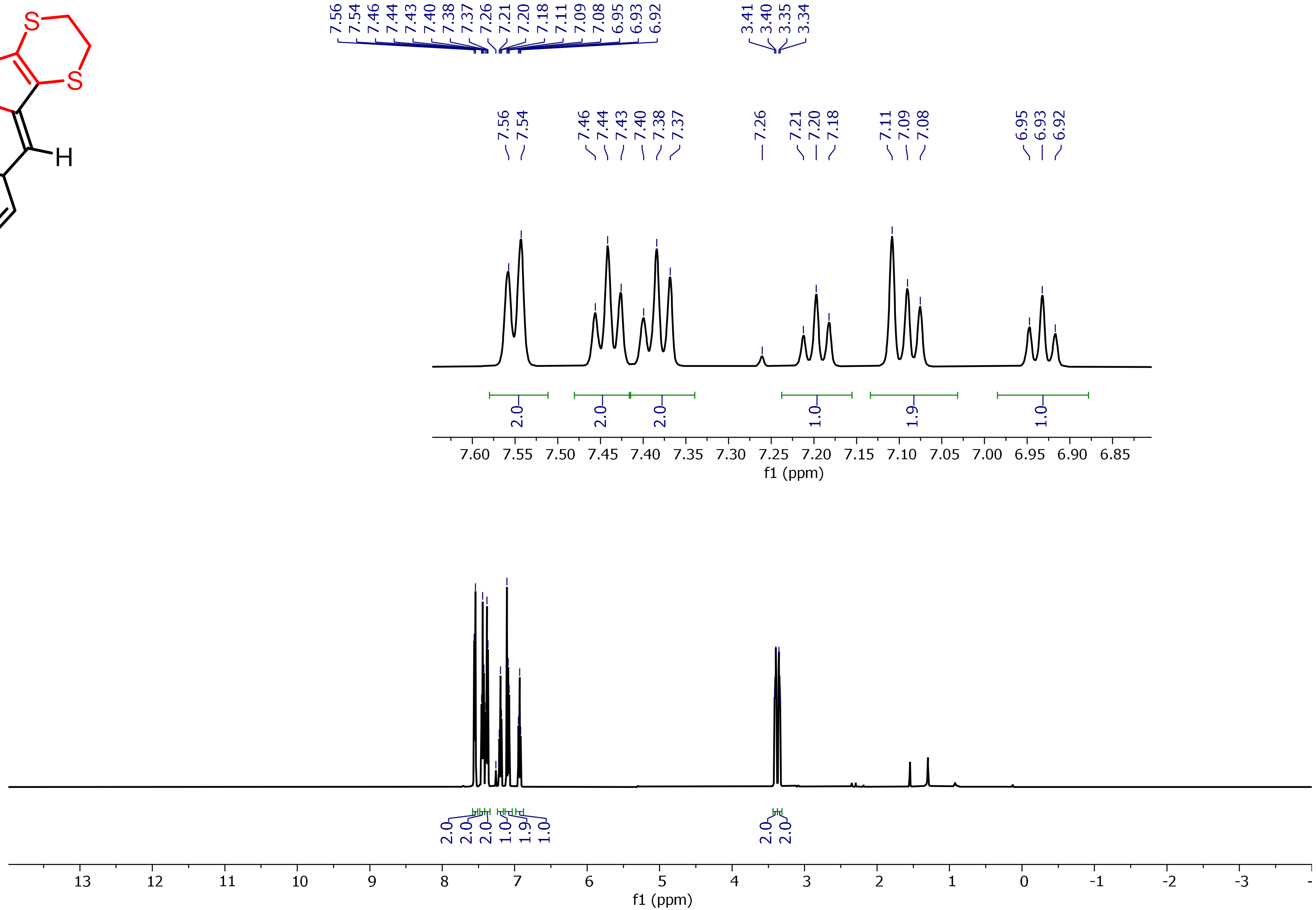

Figure S162. $^{13}C\{^1H\}$ NMR (126 MHz, $CDCl_3$, APT) spectrum **4a-(*E*)**

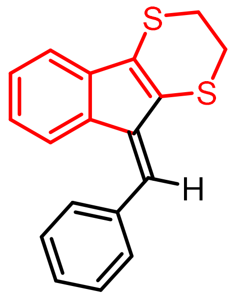

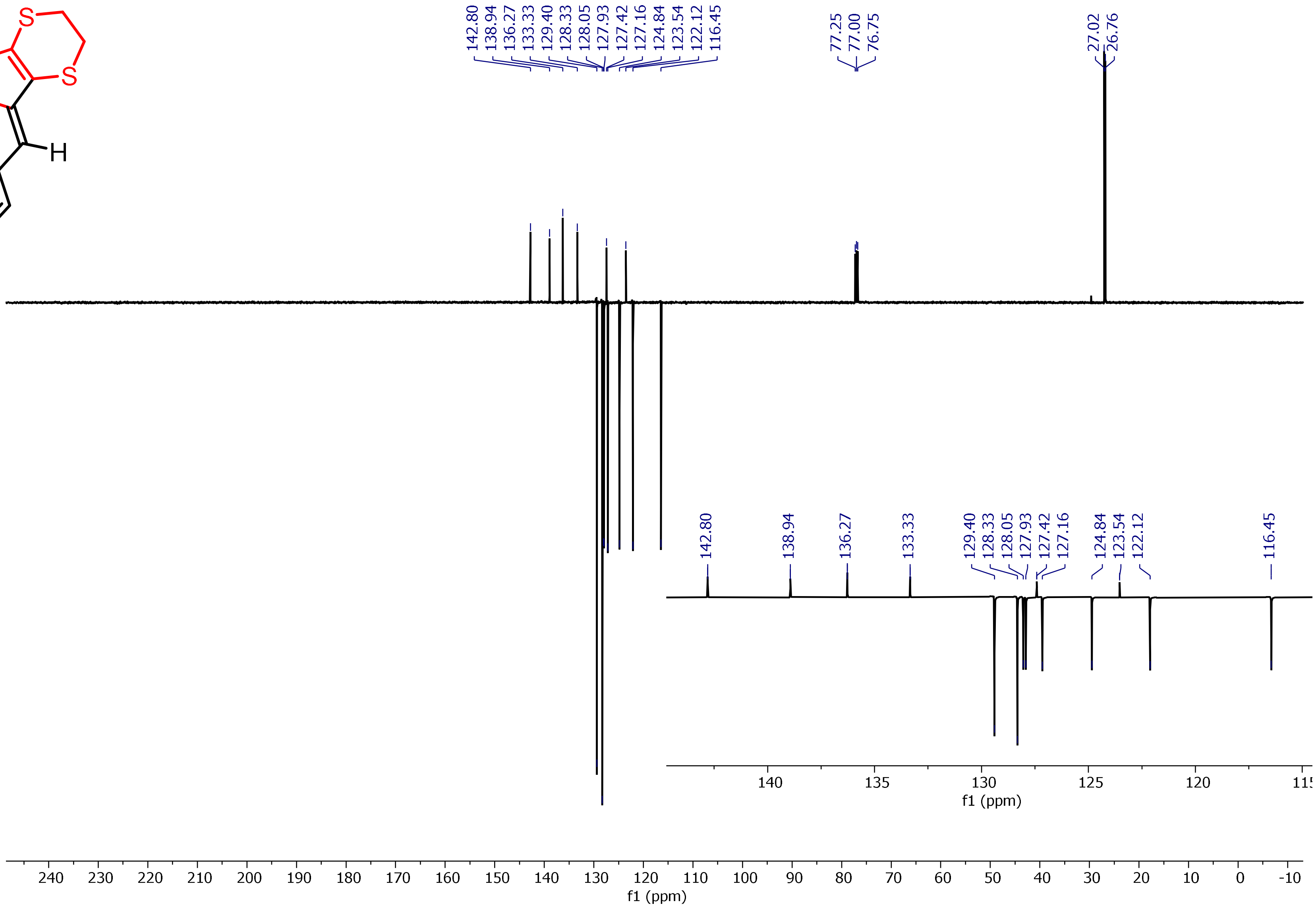

Figure S163. ^{1}H NMR ($CDCl_3$, 500 MHz) spectrum **4a**-(***E***/***Z***)

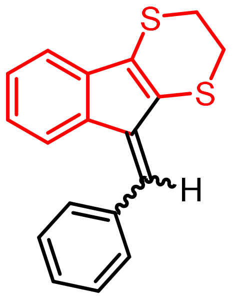

(*E*:*Z* = 3.4:1)

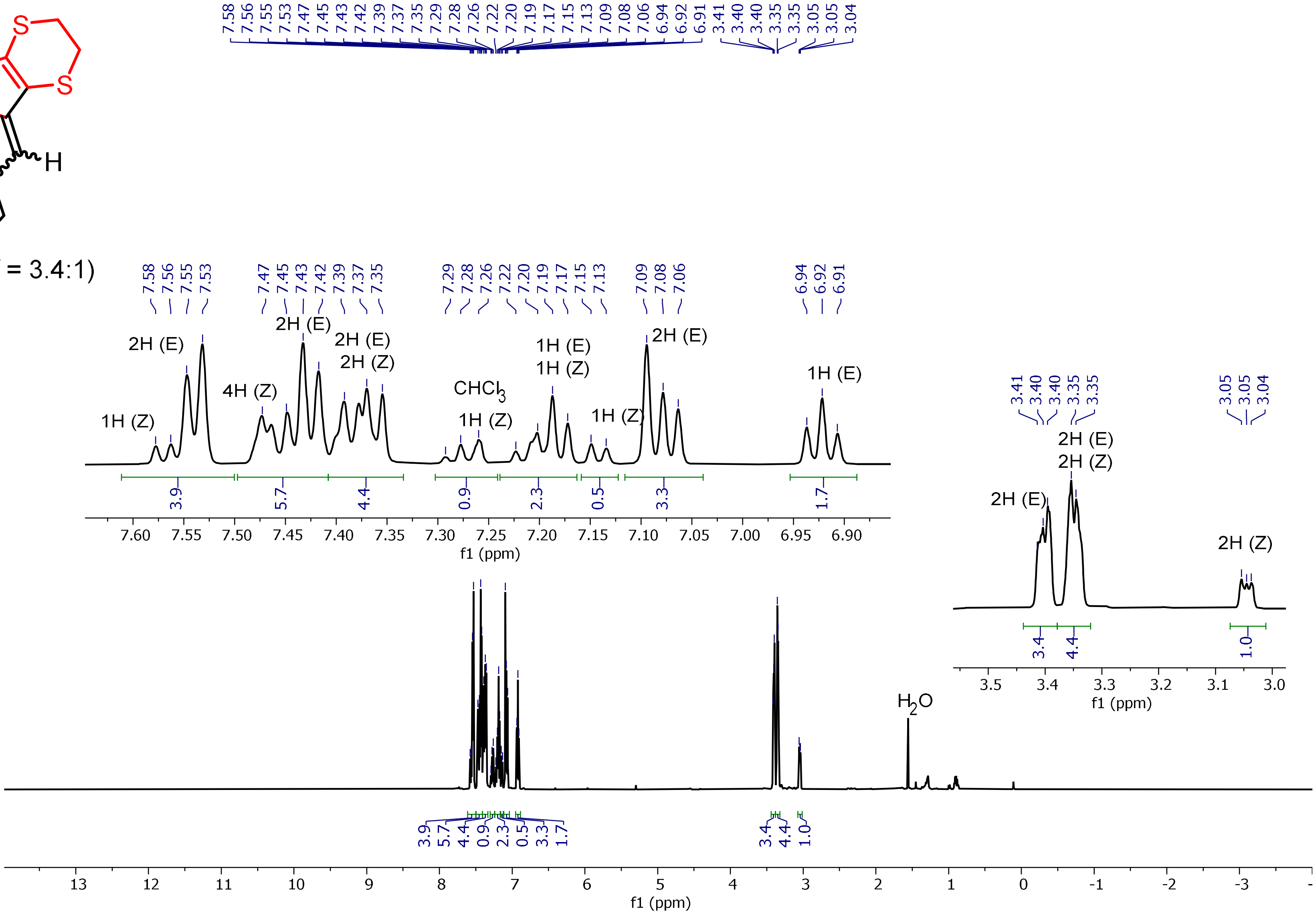

Figure S164. $^{13}C\{^{1}H\}$ NMR (126 MHz, $CDCl_3$, APT) spectrum **4a-(*E*/*Z*)**

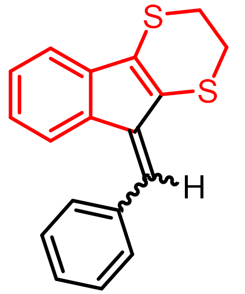

(*E*:*Z* = 3.4:1)

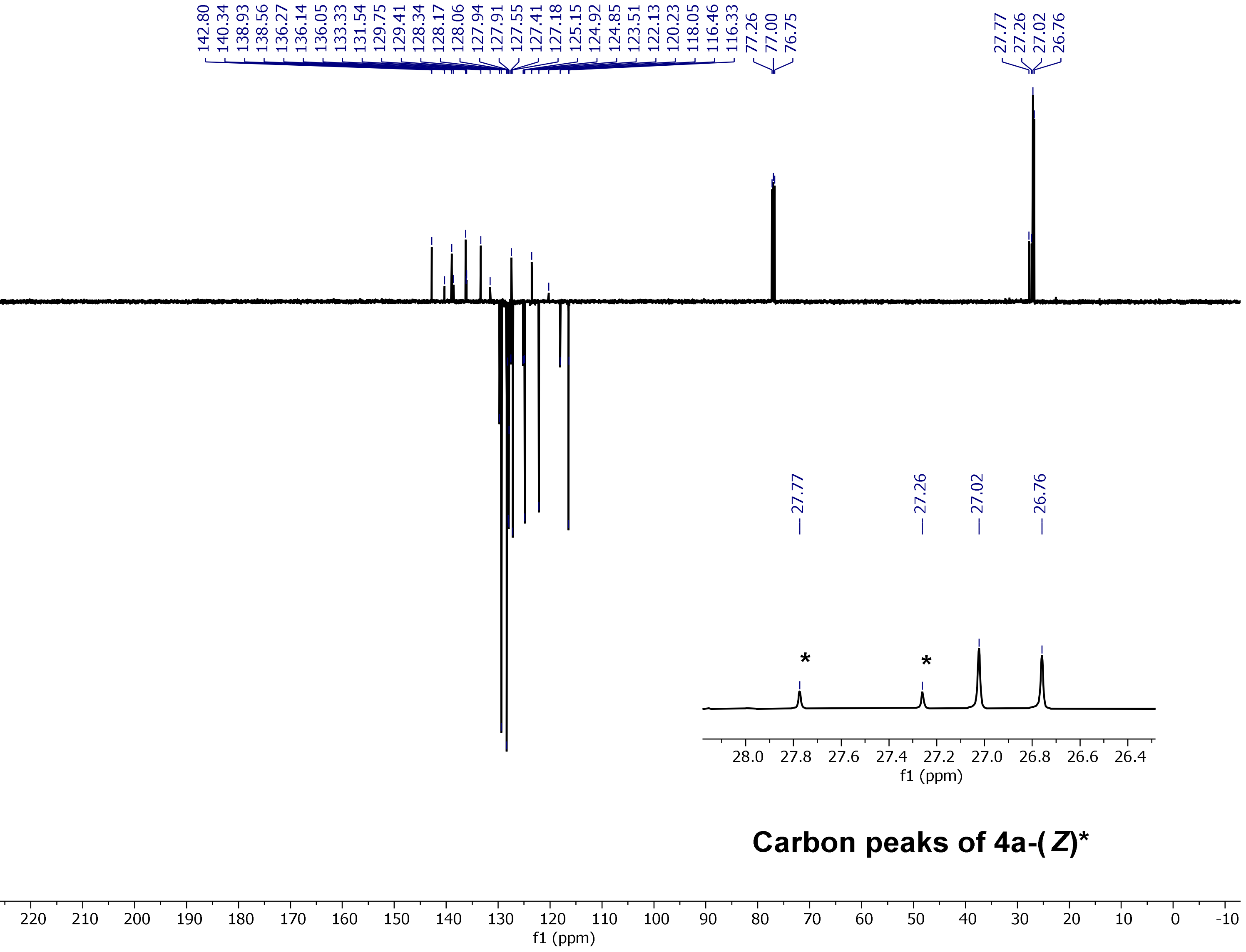

Figure S165. $^{13}C\{^{1}H\}$ NMR (126 MHz, $CDCl_3$, APT) spectrum **4a-(*E*/*Z*)** , [143-129 ppm]

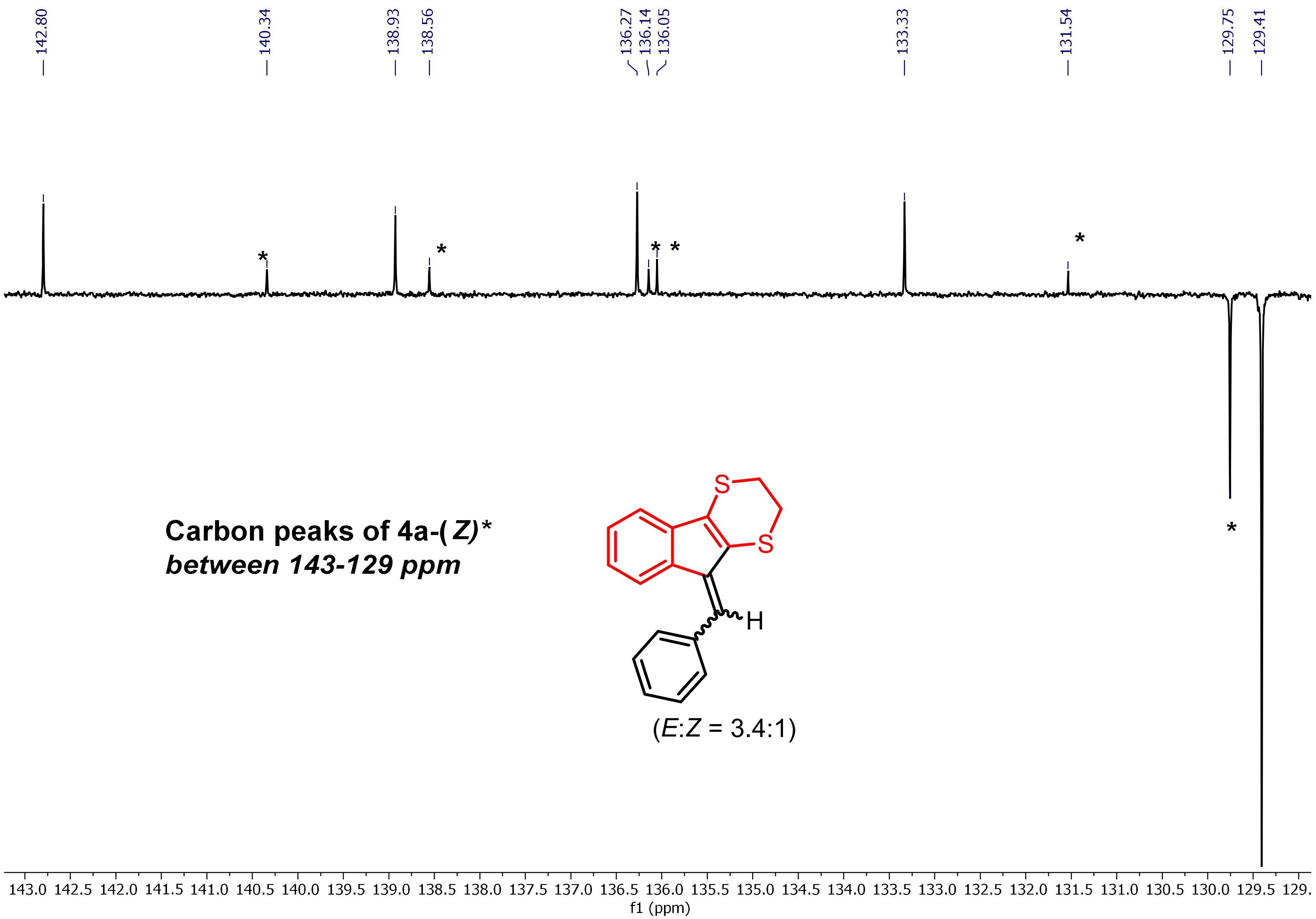

Figure S166. $^{13}C\{^{1}H\}$ NMR (126 MHz, $CDCl_3$, APT) spectrum **4a-(*E*/*Z*)** , [129-115 ppm]

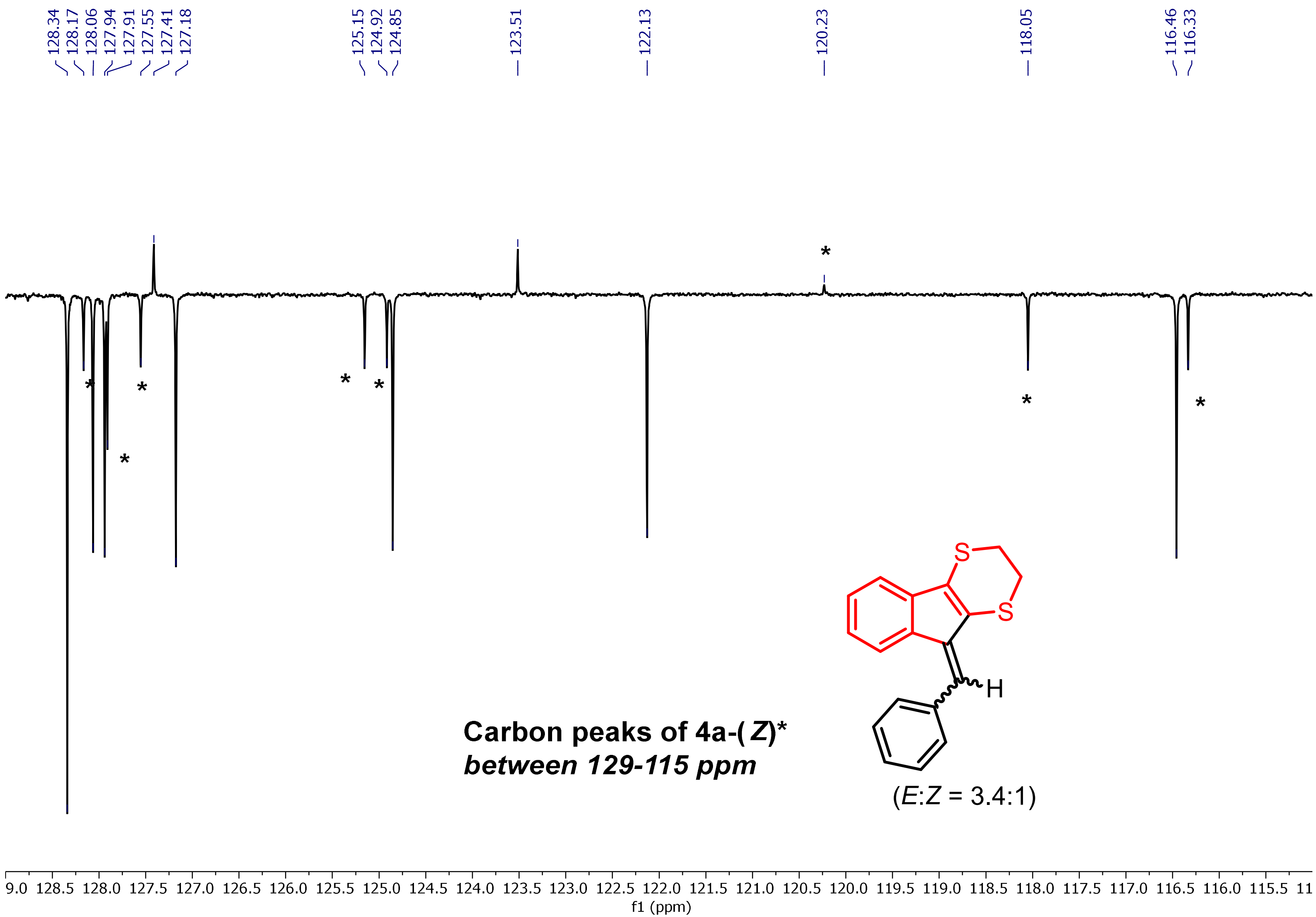

Figure S167. ^{1}H NMR ($CDCl_3$, 500 MHz) spectrum **4b**-(***E***)

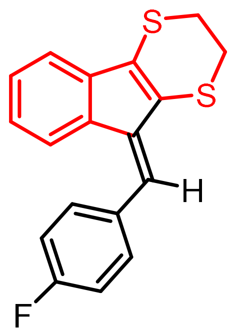

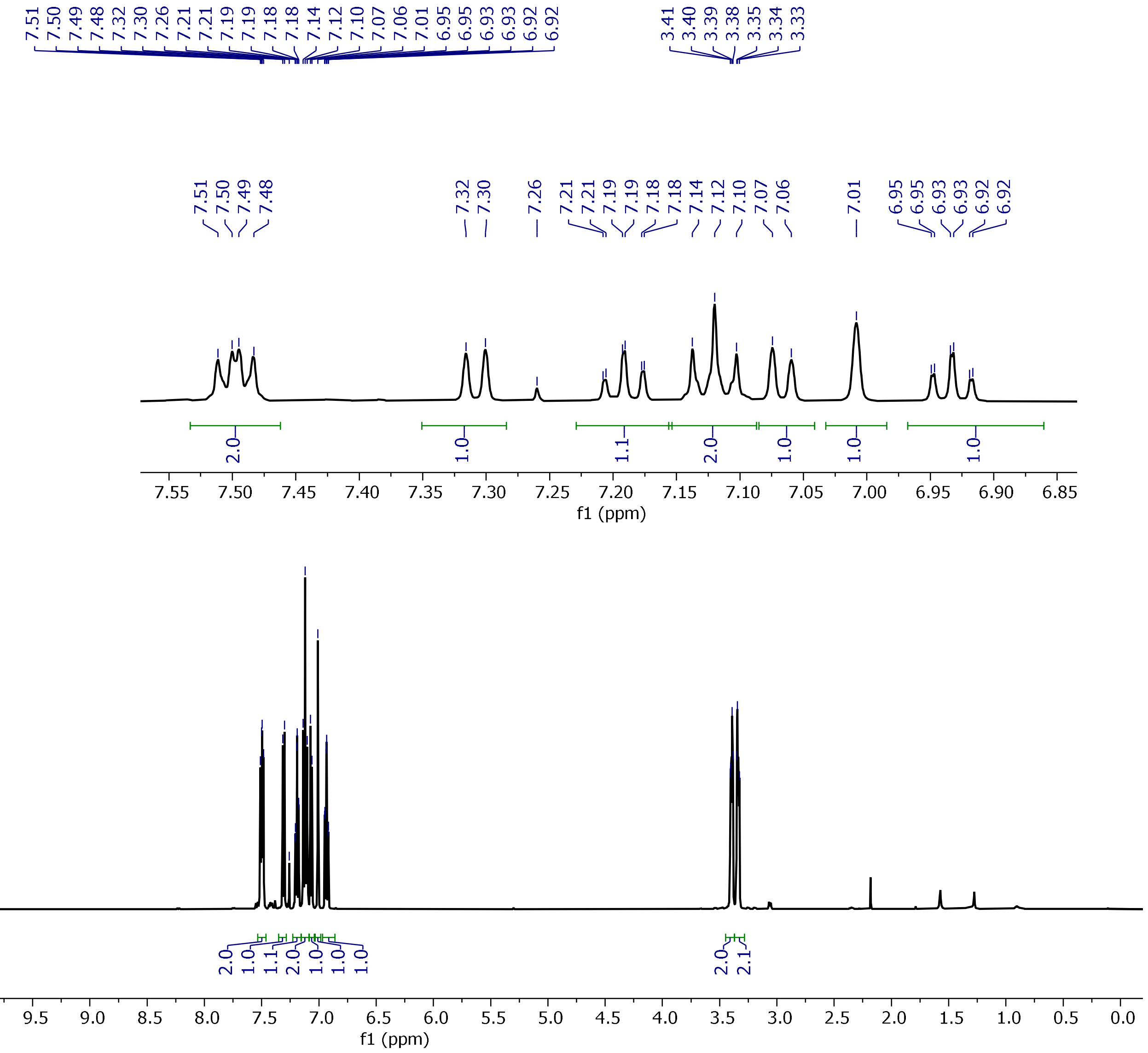

Figure S168. $^{13}C\{^{1}H\}$ NMR (126 MHz, $CDCl_3$, APT) spectrum **4b-(*E*)**

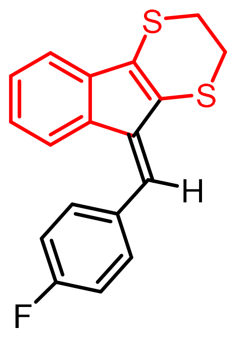

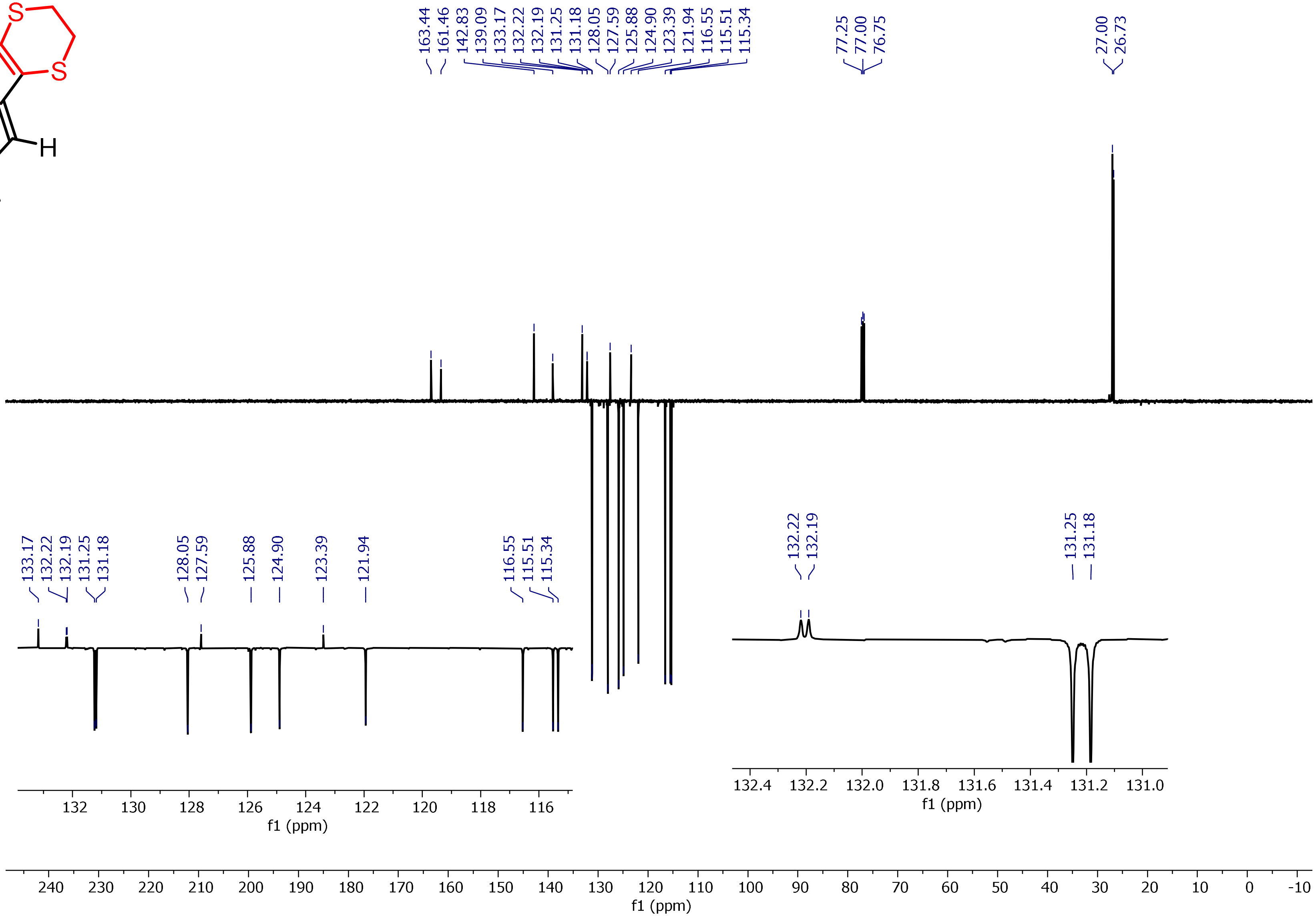

Figure S169. ^{1}H NMR (CDCl$_3$, 500 MHz) spectrum **4b**-(***E***/***Z***)

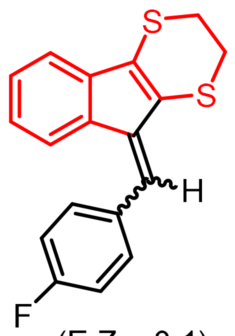

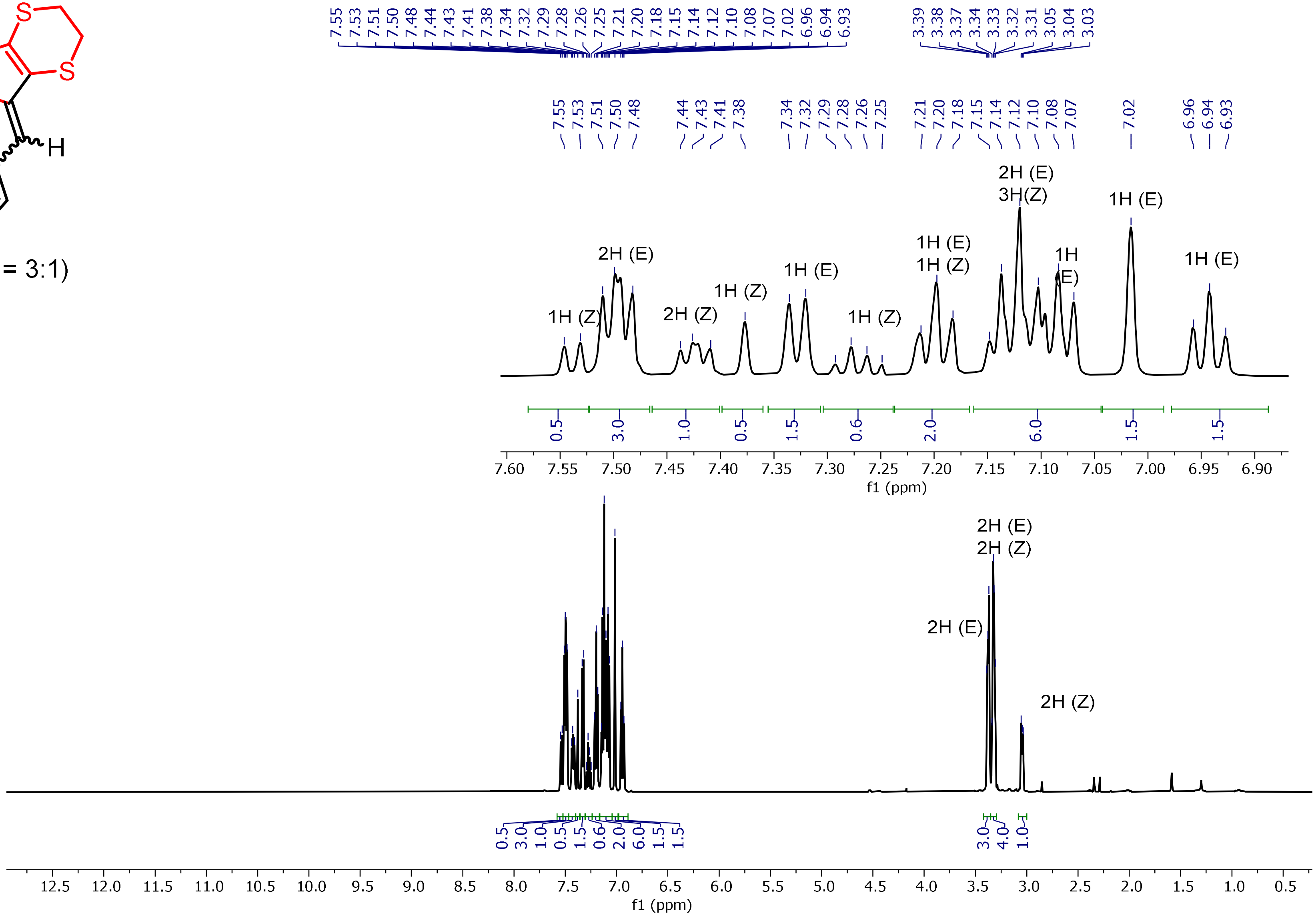

Figure S170. $^{13}C\{^1H\}$ NMR (126 MHz, $CDCl_3$, APT) spectrum **4b-(*E*/*Z*)**

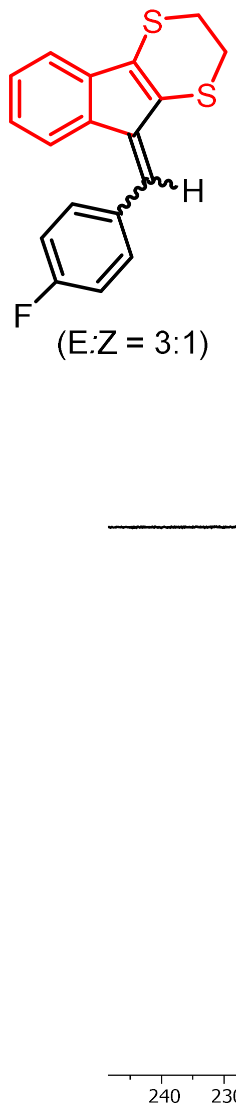
(E:Z = 3:1)

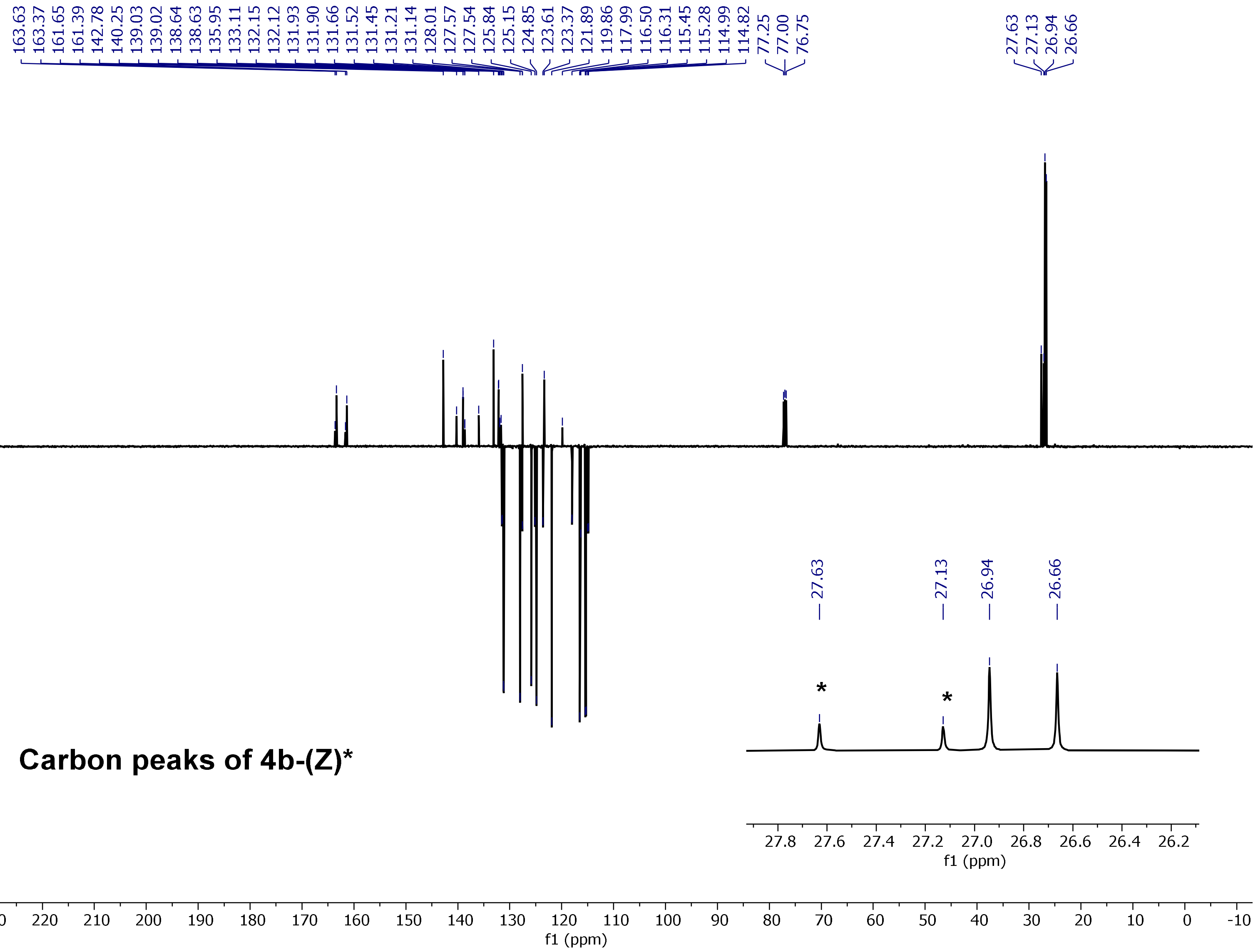

Figure S171. $^{13}C\{^{1}H\}$ NMR (126 MHz, $CDCl_3$, APT) spectrum **4b-(*E*/*Z*)**, [170-135 ppm]

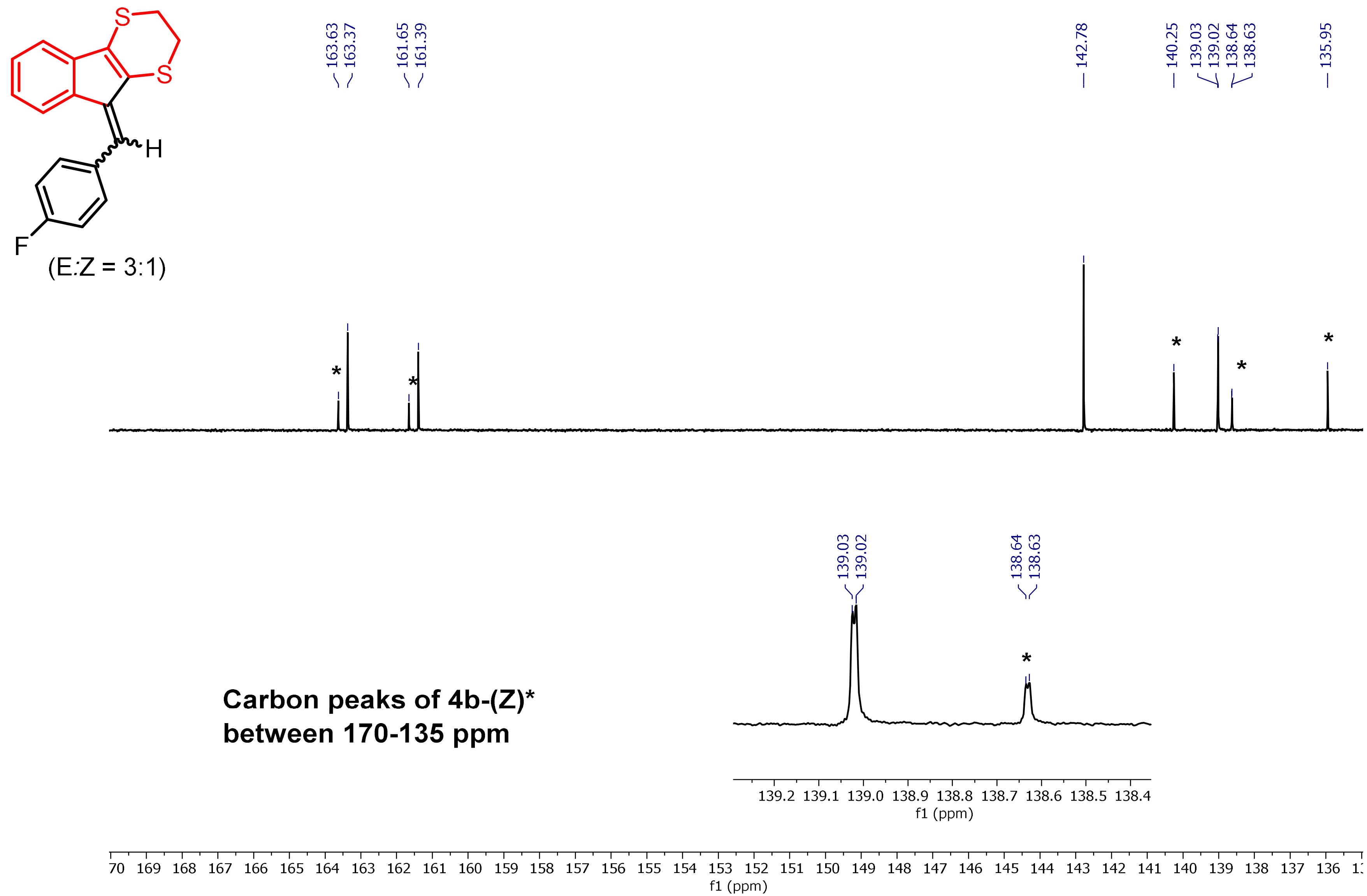

Figure S172. $^{13}C\{^{1}H\}$ NMR (126 MHz, $CDCl_3$, APT) spectrum **4b-(*E*/*Z*)**, [135-110 ppm]

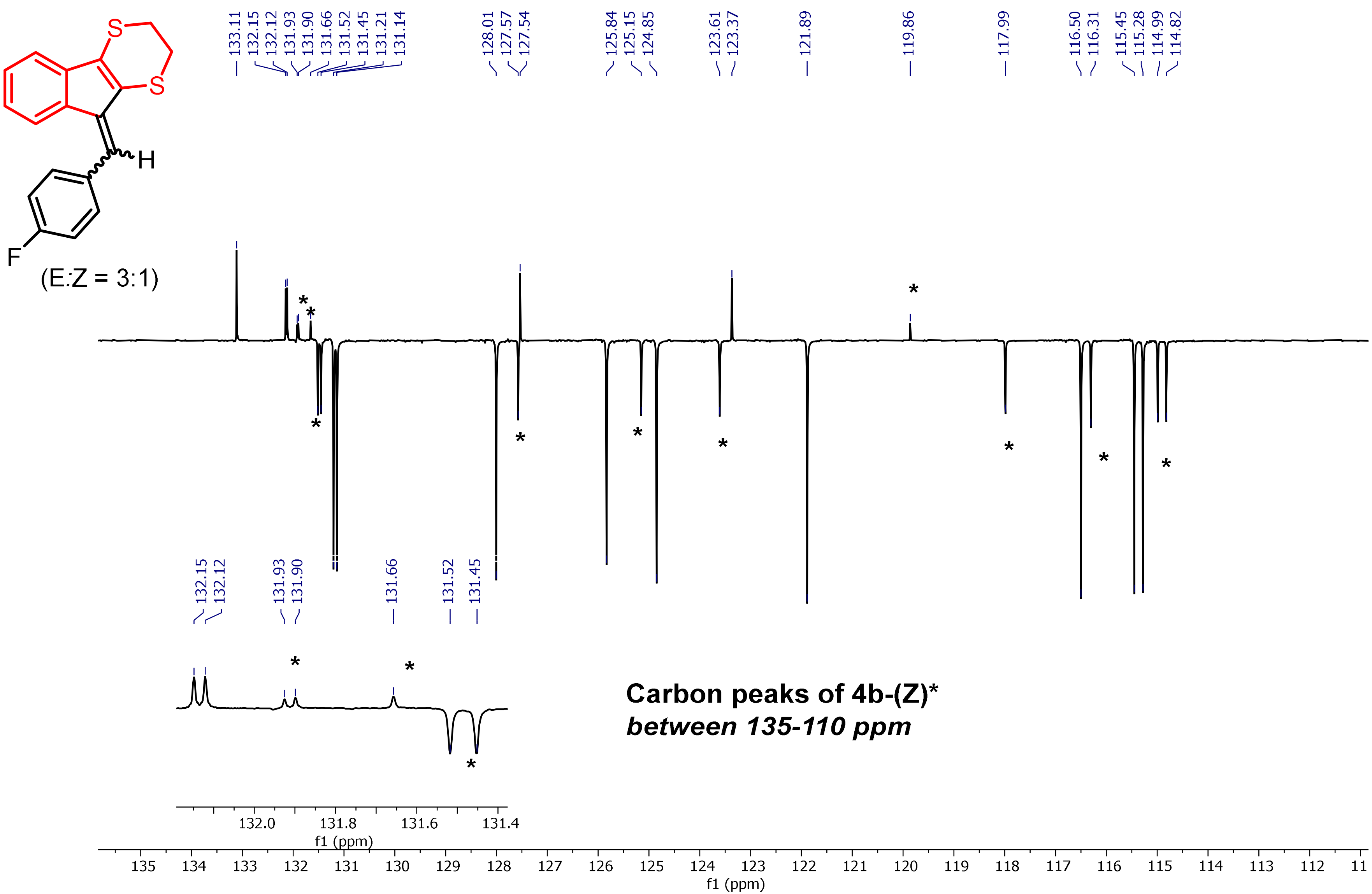

Figure S173. ^{1}H NMR ($CDCl_3$, 500 MHz) spectrum **4c-(*E*/*Z*)**

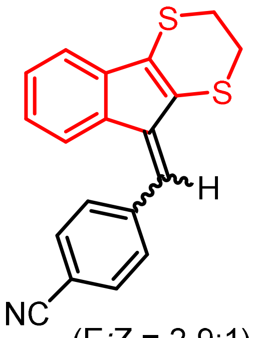

(E:Z = 2.9:1)

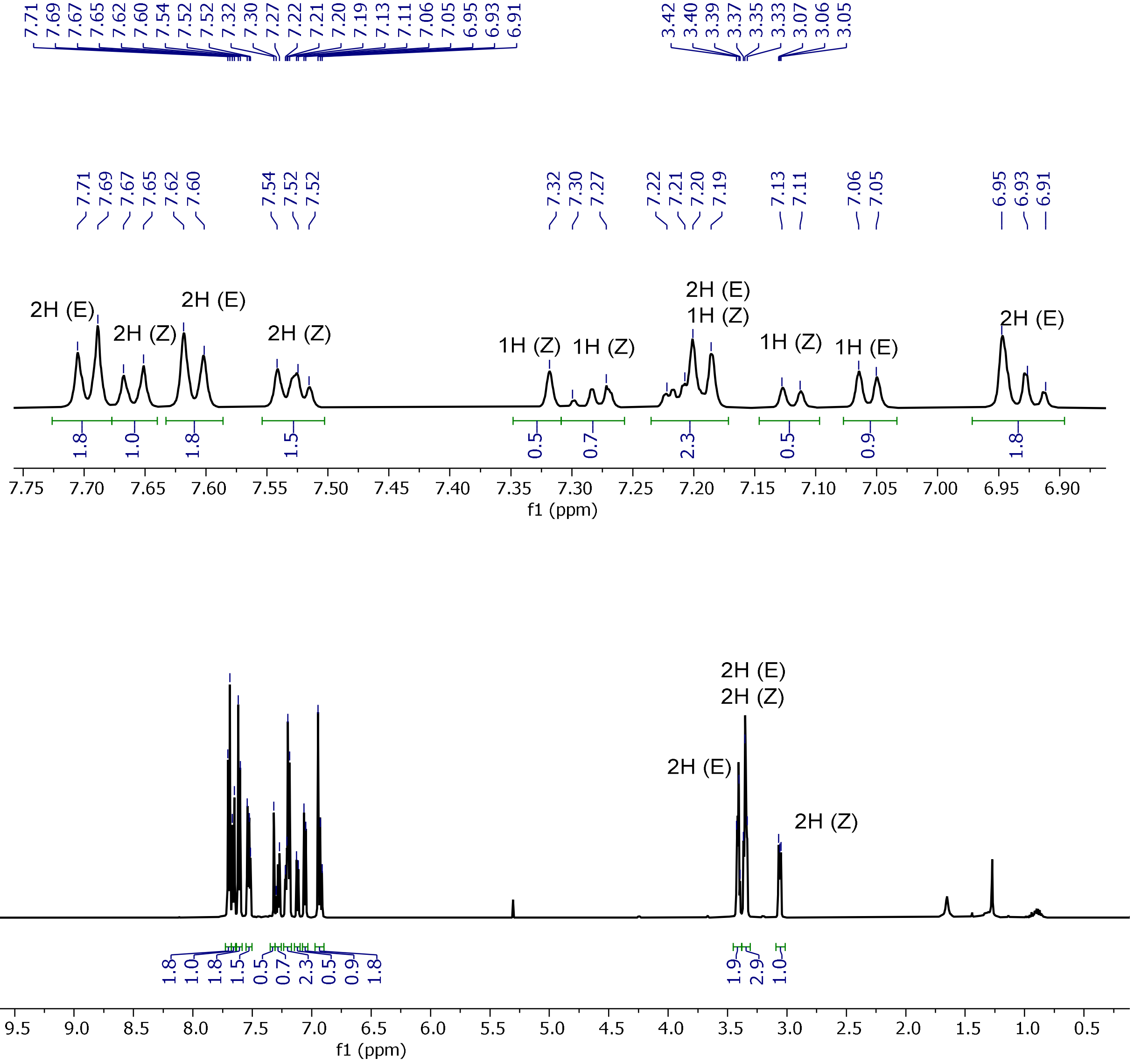

Figure S174. $^{13}C\{^{1}H\}$ NMR (126 MHz, $CDCl_3$, APT) spectrum **4c-(*E*/*Z*)**

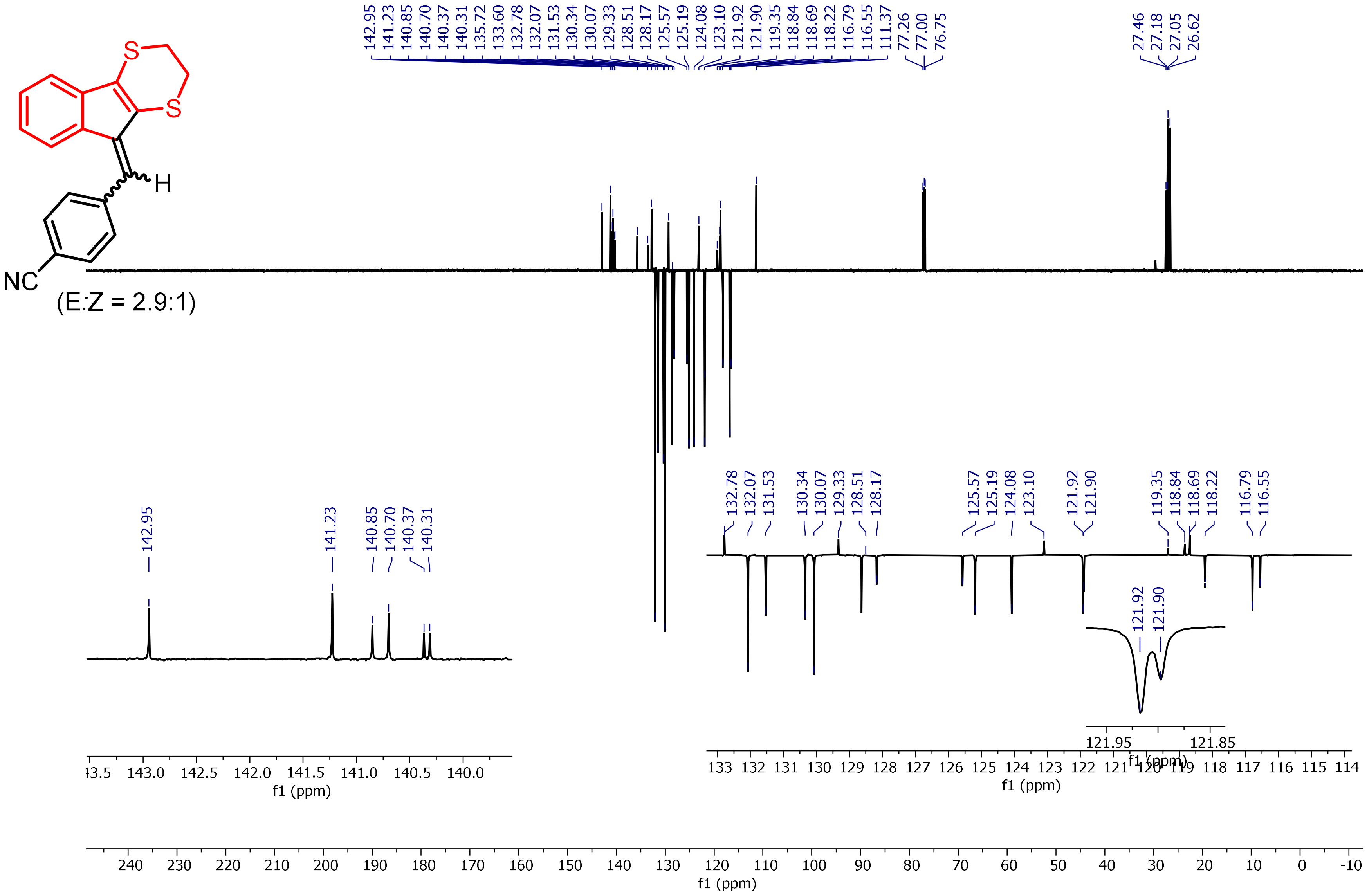

Figure S175. ^{1}H NMR (CDCl$_3$, 500 MHz) spectrum **4d**-(***E*/*Z***)

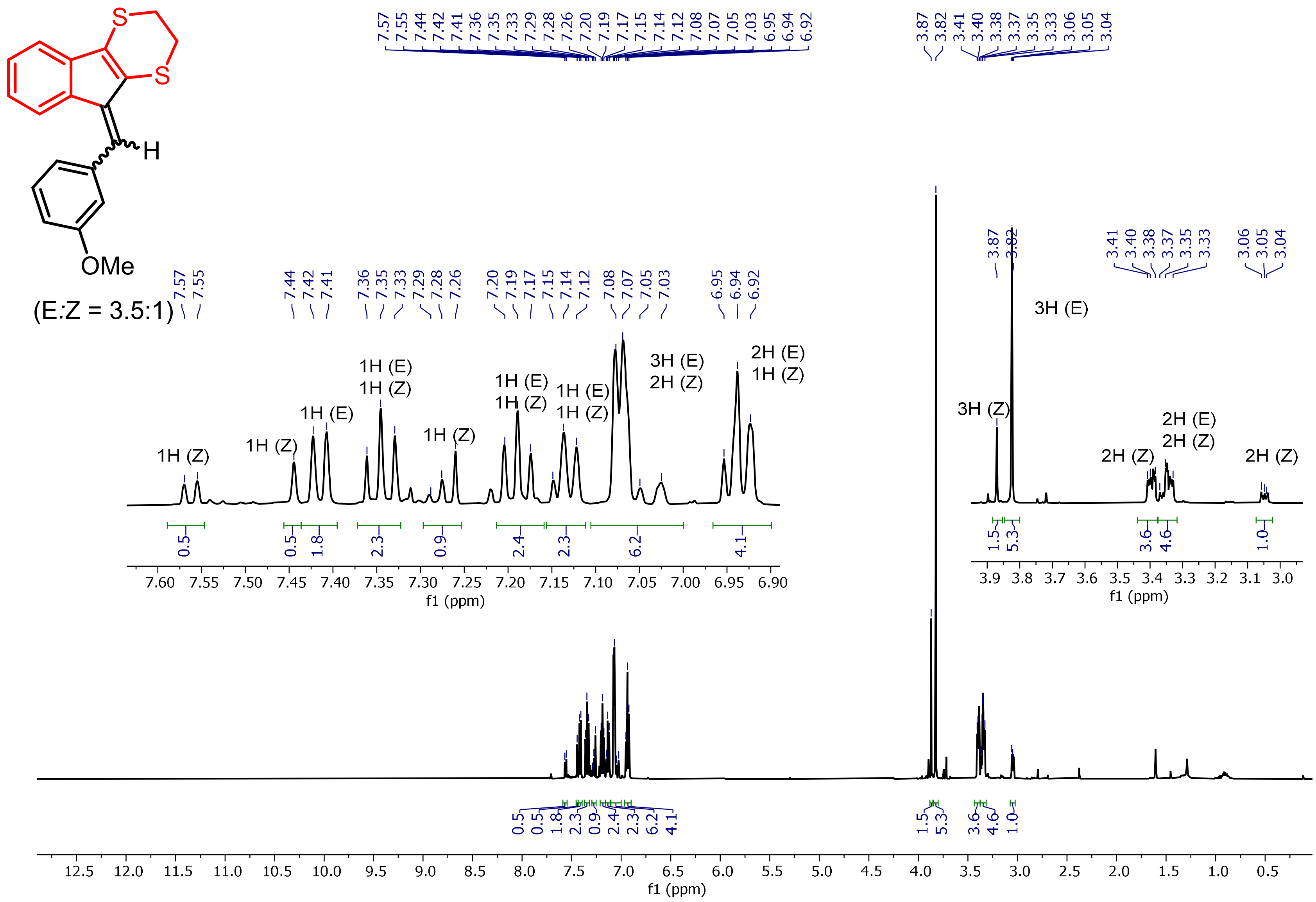

Figure S176. $^{13}C\{^1H\}$ NMR (126 MHz, $CDCl_3$, APT) spectrum **4d-(*E*/*Z*)**

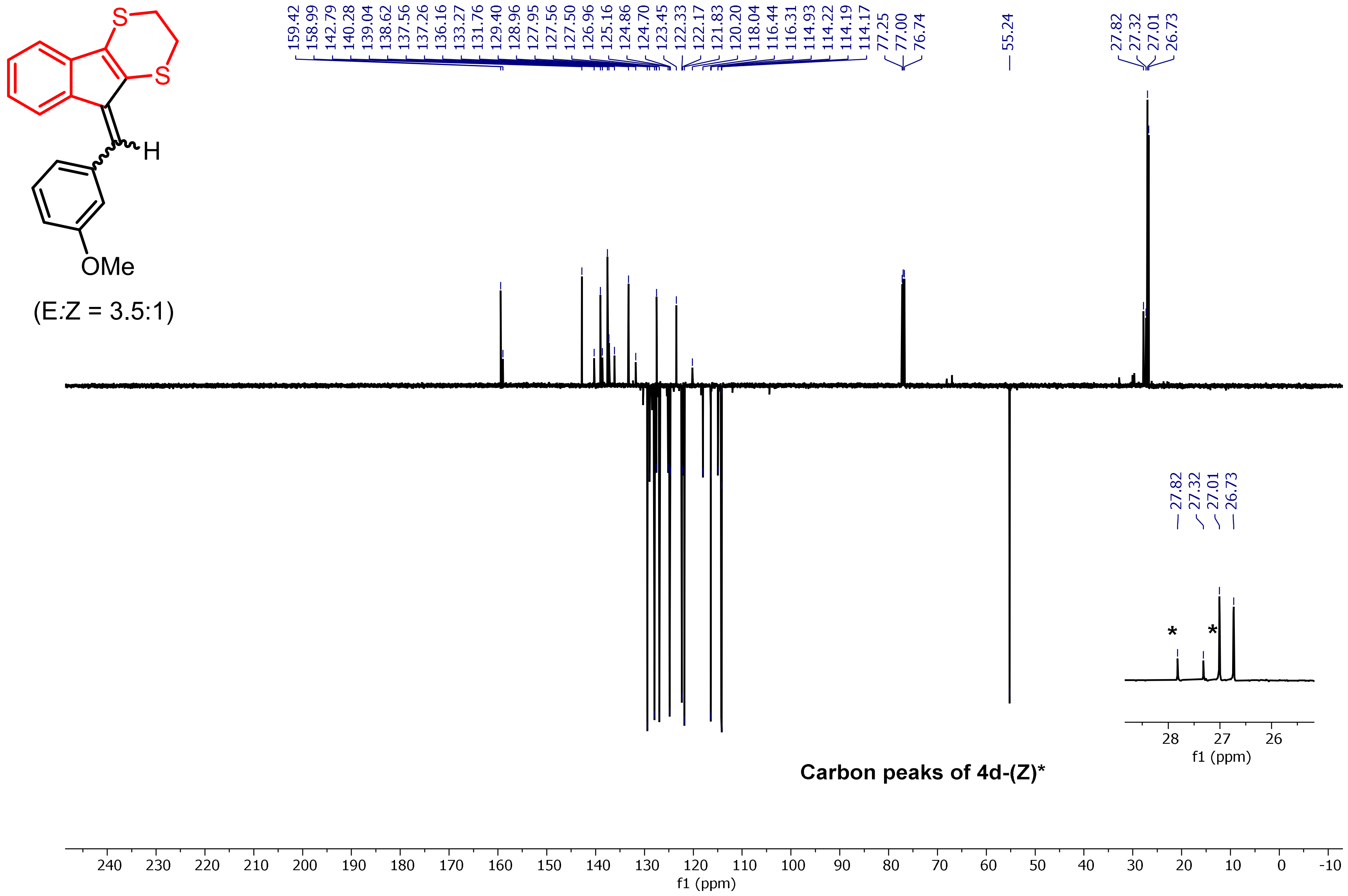

Figure S177. $^{13}C\{^{1}H\}$ NMR (126 MHz, $CDCl_3$, APT) **4d-(*E*/*Z*)**, [165-130 ppm]

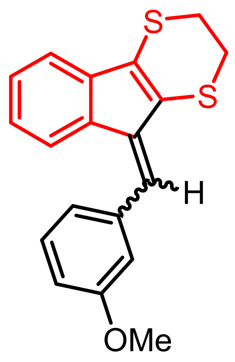

(E:Z = 3.5:1)

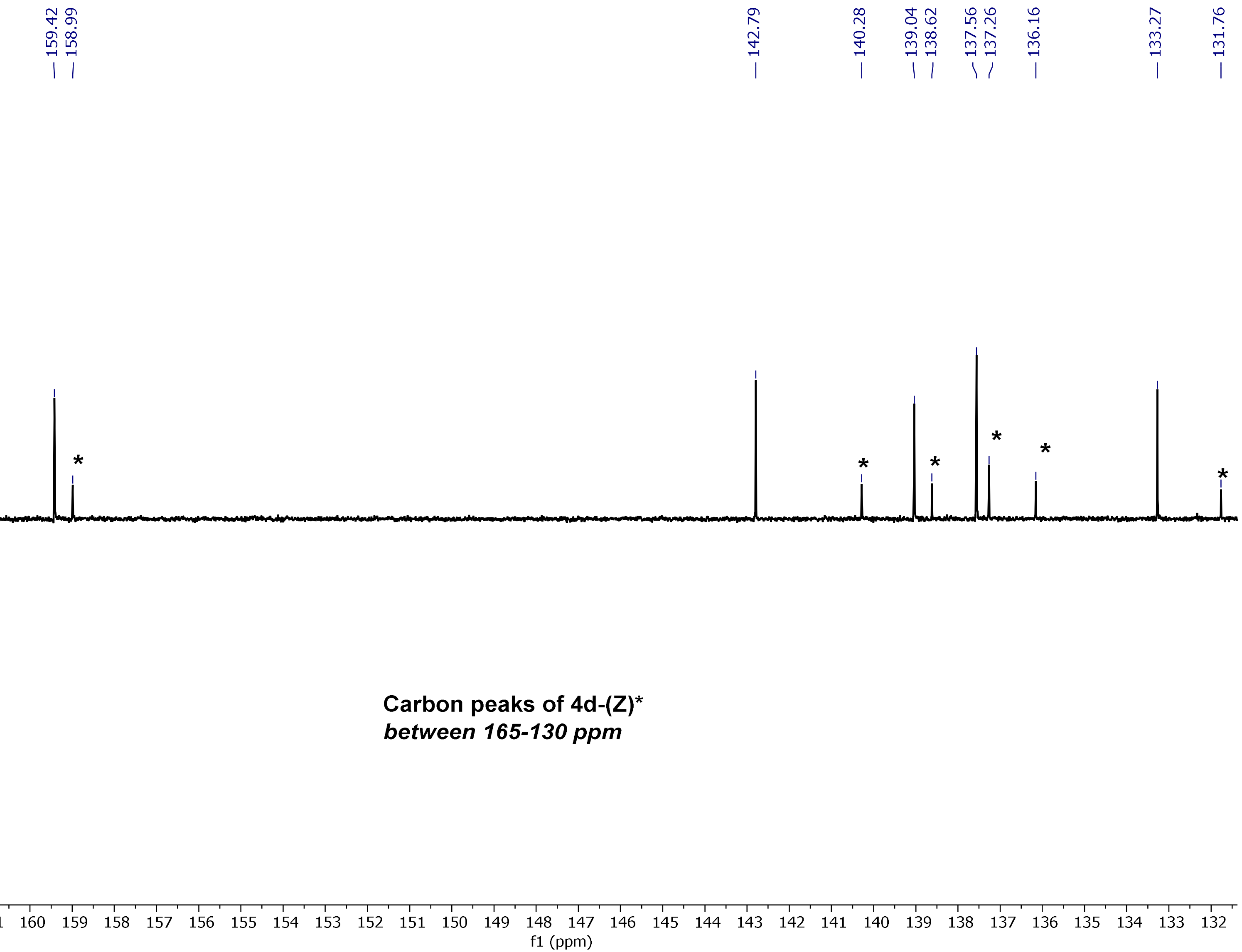

Carbon peaks of 4d-(Z)*
between 165-130 ppm

Figure S178. $^{13}C\{^{1}H\}$ NMR (126 MHz, $CDCl_3$, APT) spectrum **4d-(*E*/*Z*)** [130-110 ppm]

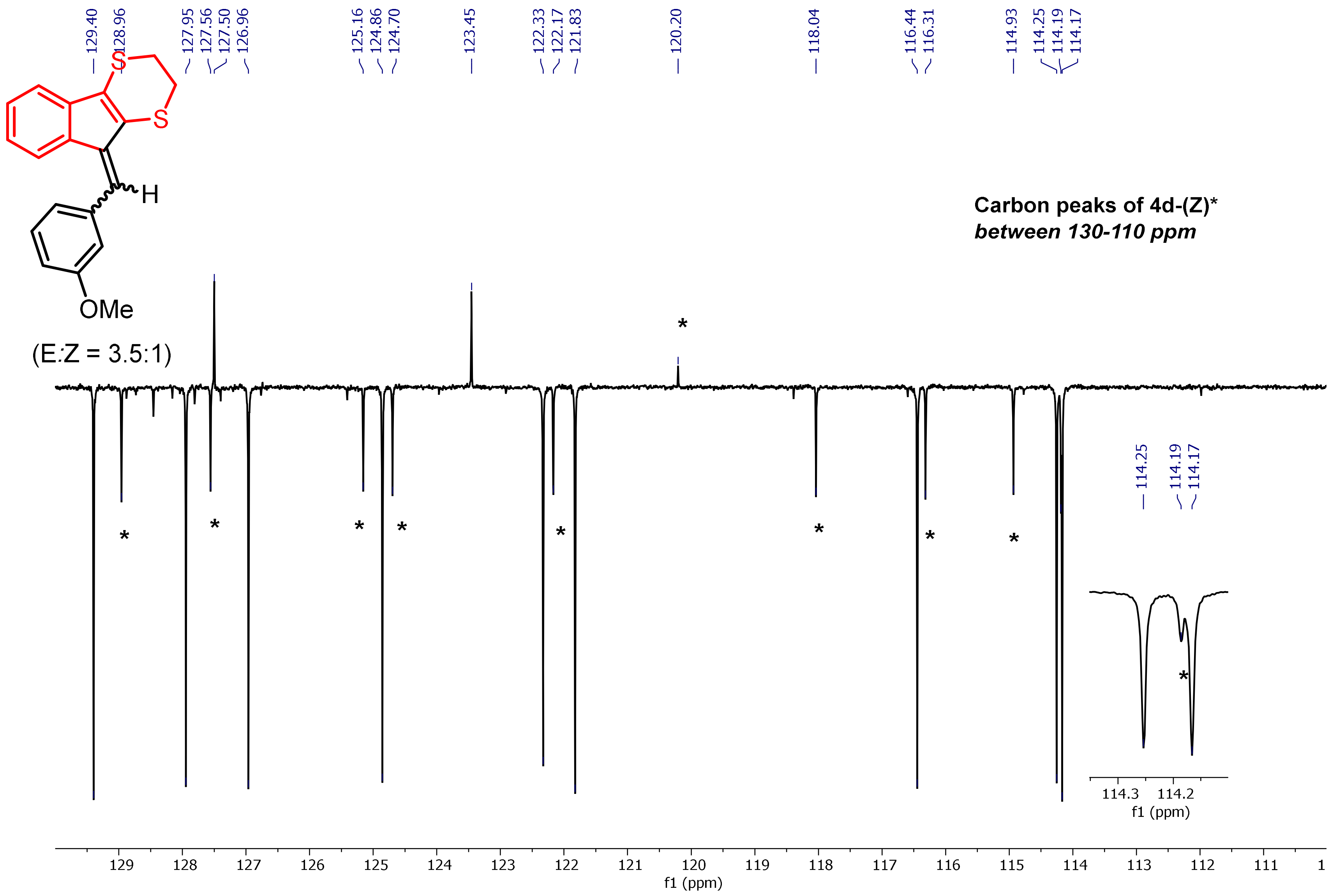

Figure S179. ^{1}H NMR ($CDCl_3$, 500 MHz) spectrum **4e-(*E*)**

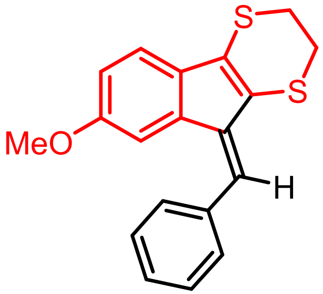

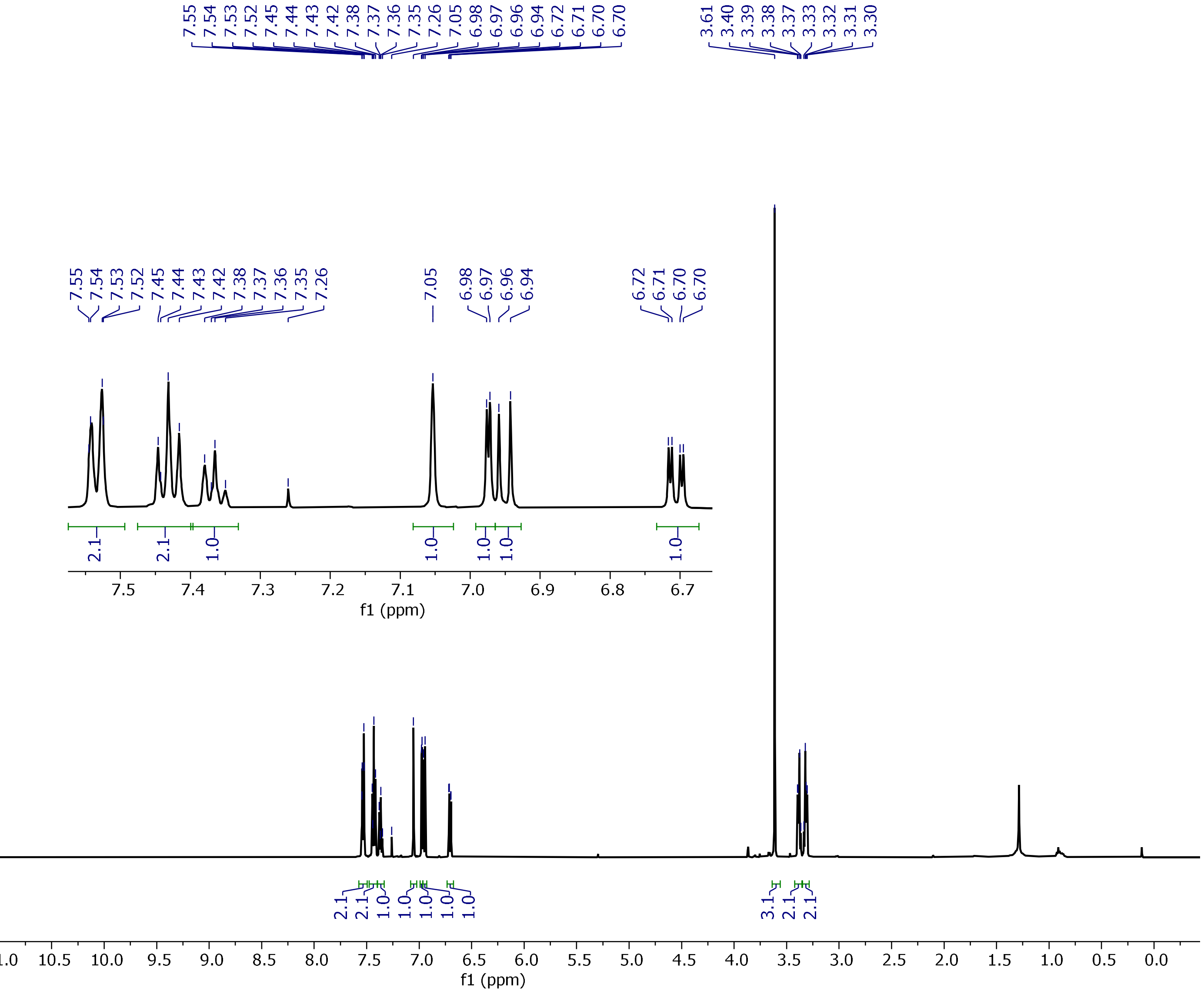

Figure S180. $^{13}C\{^1H\}$ NMR (126 MHz, $CDCl_3$, APT) spectrum **4e-(*E*)**

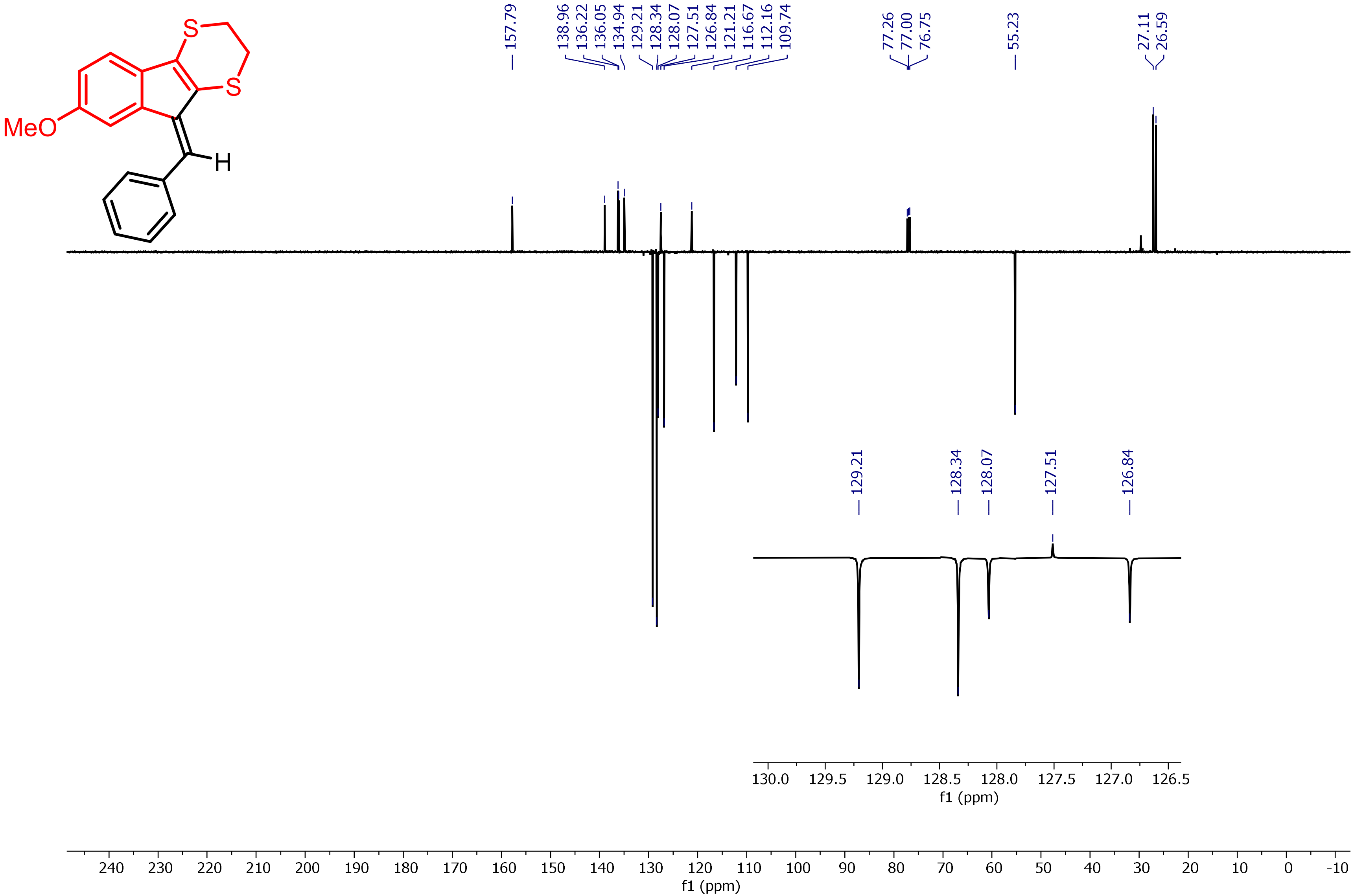

Figure S181. ^{1}H NMR ($CDCl_3$, 500 MHz) spectrum **4e-(*Z*)**

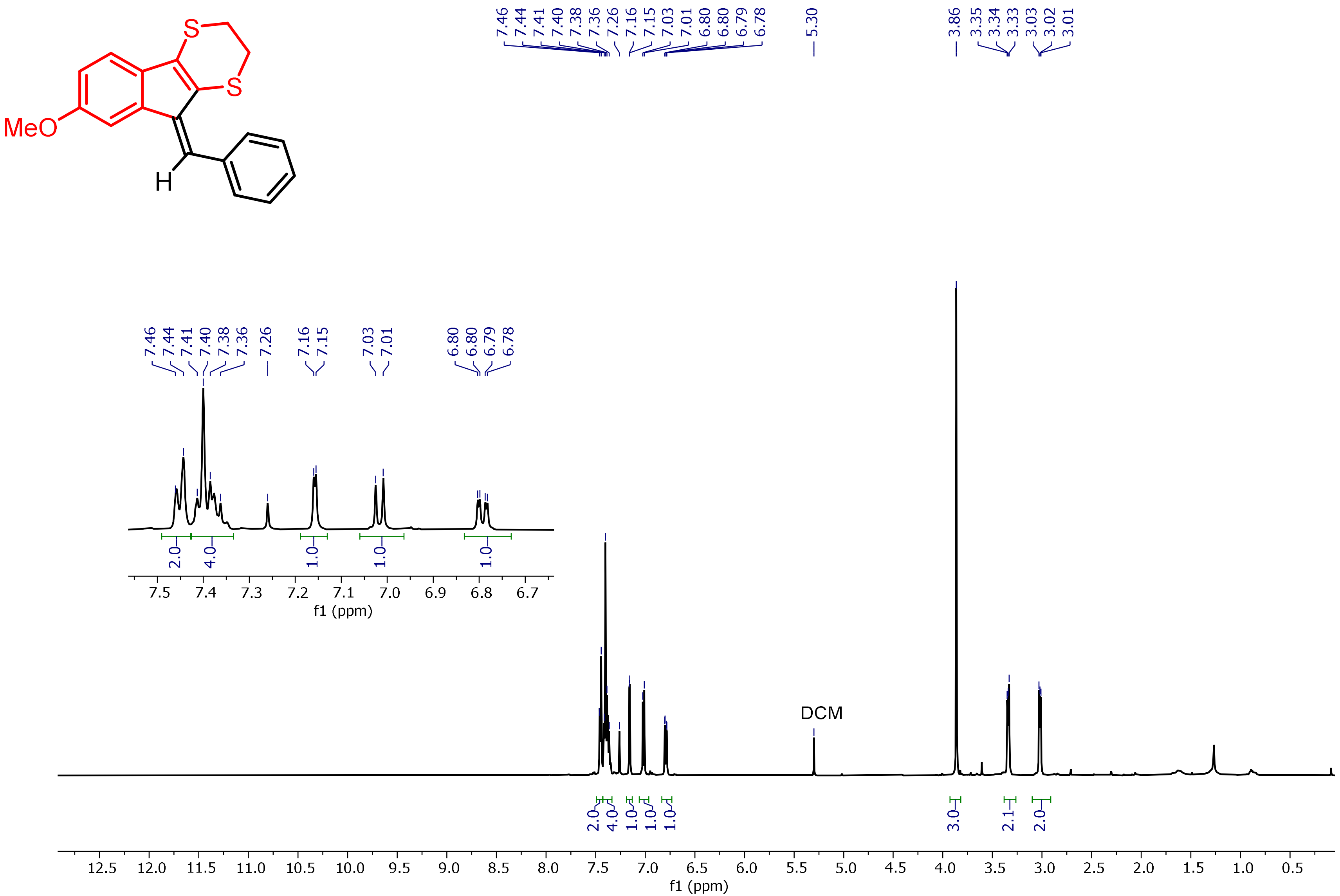

Figure S182. $^{13}C\{^{1}H\}$ NMR (126 MHz, $CDCl_3$, APT) spectrum **4e-(*Z*)**

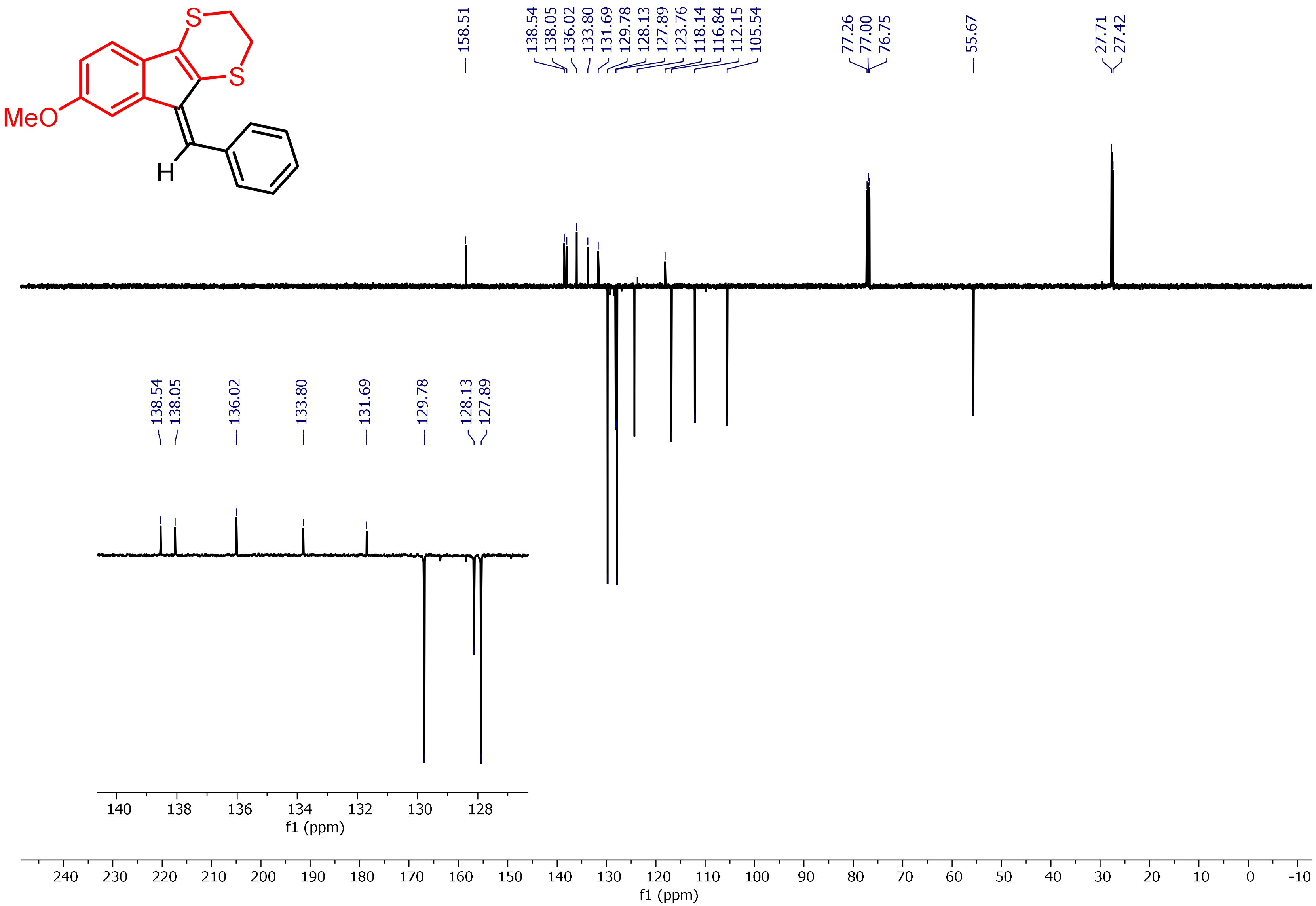

Figure S183. ^{1}H NMR ($CDCl_3$, 500 MHz) spectrum **4f-(*E*)**

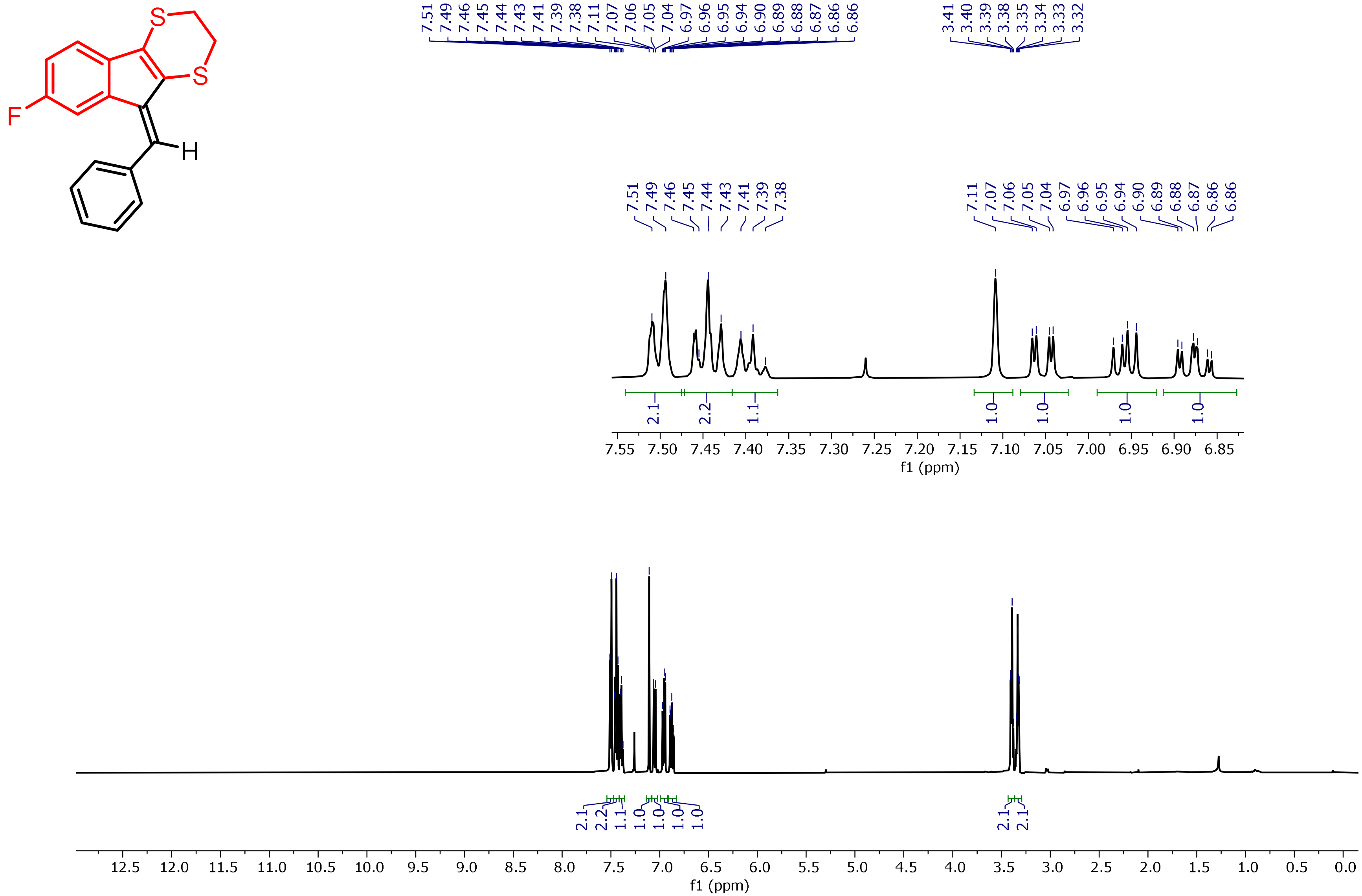

Figure S184. $^{13}C\{^1H\}$ NMR (126 MHz, $CDCl_3$, APT) spectrum **4f-(*E*)**

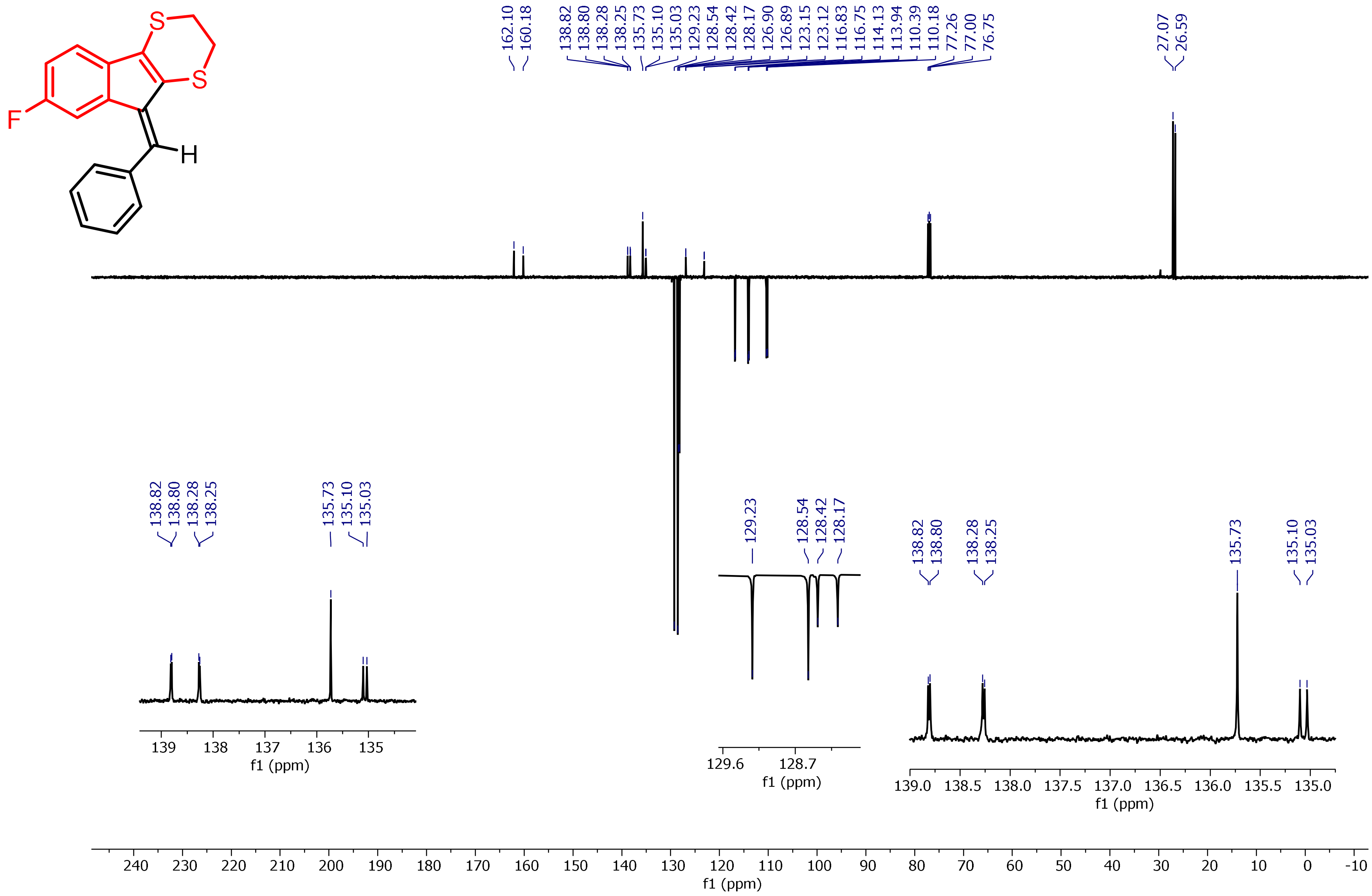

Figure S185. ^{1}H NMR ($CDCl_3$, 500 MHz) spectrum **4f-(*Z*)**

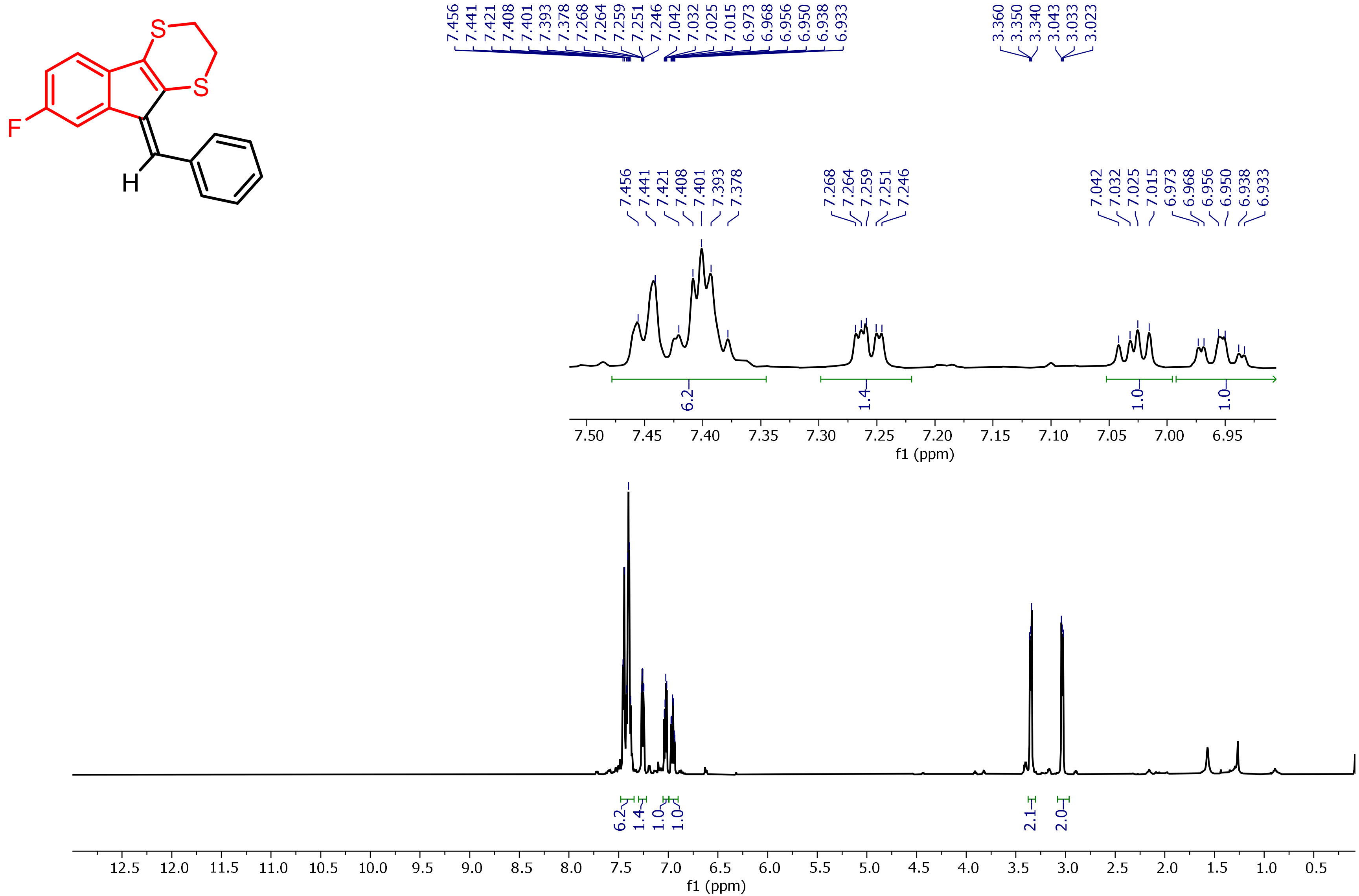

Figure S186. $^{13}C\{^{1}H\}$ NMR (126 MHz, $CDCl_3$, APT) spectrum **4f-(*Z*)**

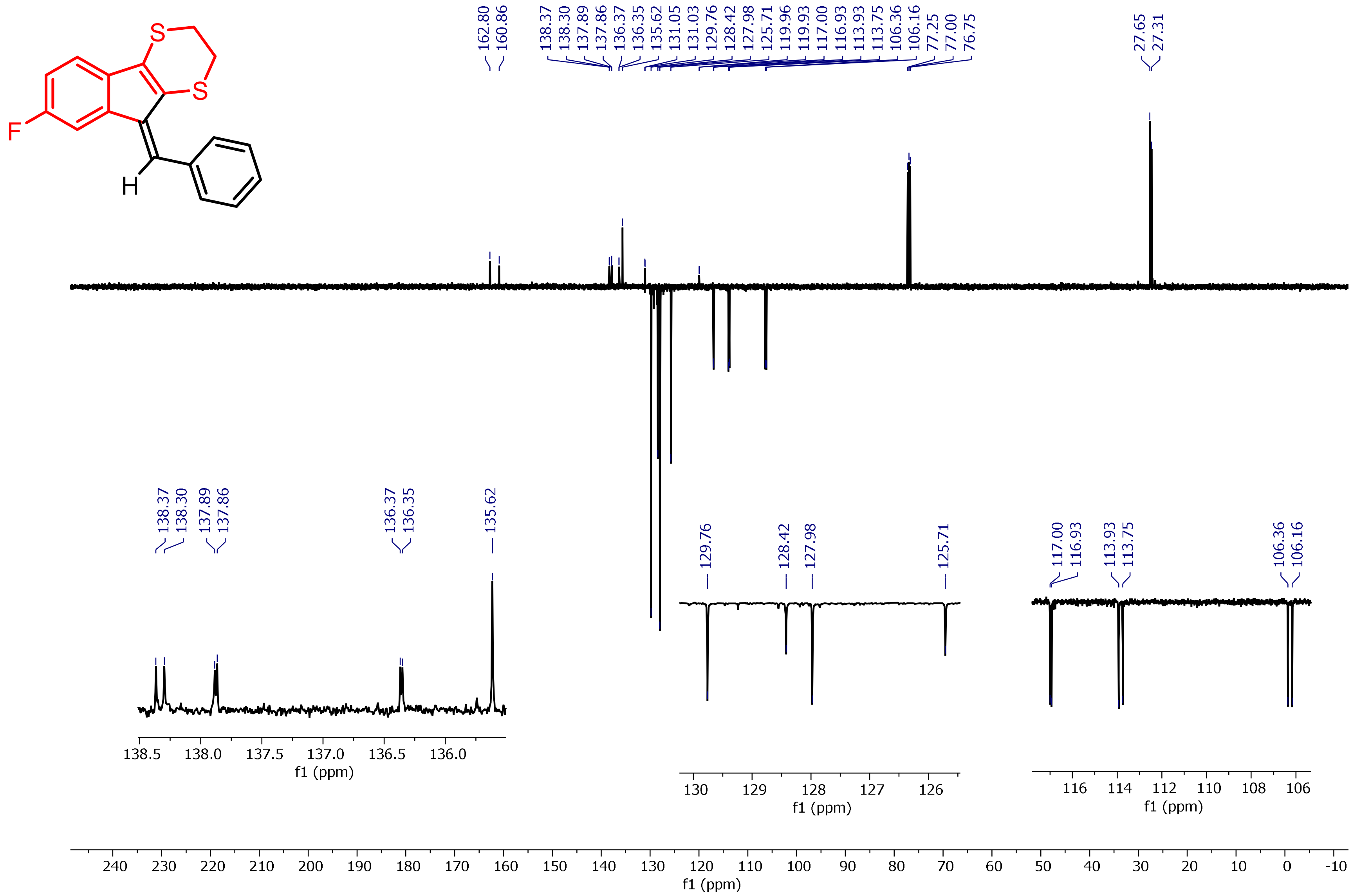

Figure S187. ^{1}H NMR ($CDCl_3$, 500 MHz) spectrum **6a**-(***E***)

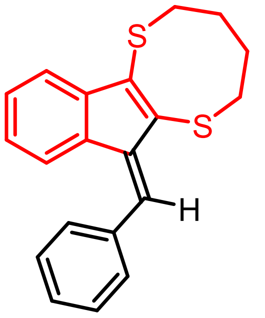

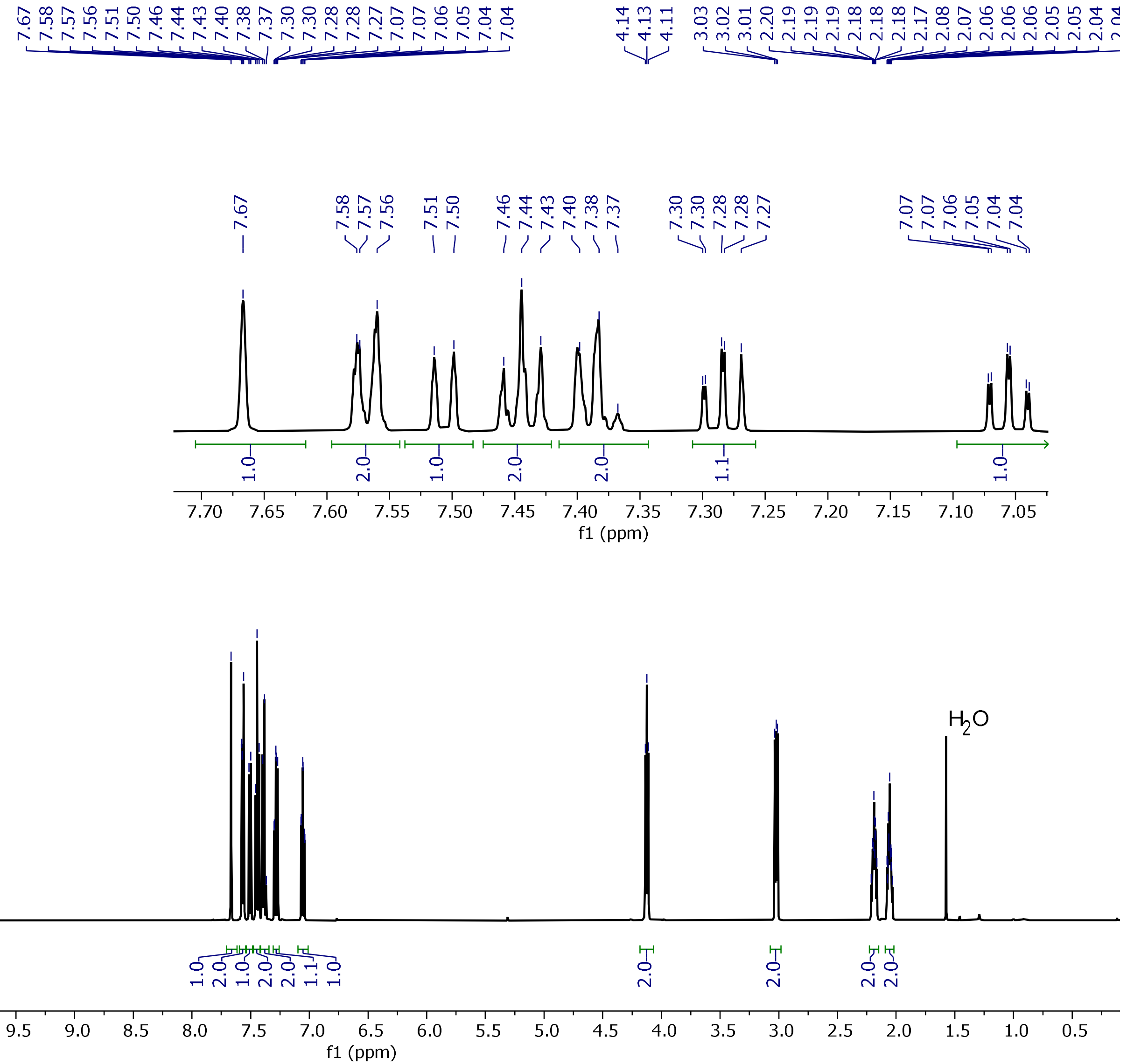

Figure S188. 1D NOE NMR ($CDCl_3$, 500 MHz) spectrum **6a-(*E*)** [irradiation of the signal at 7.67 ppm]

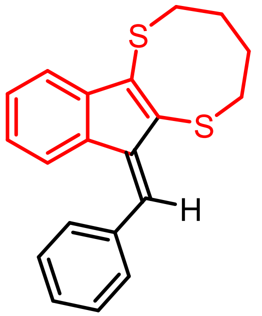

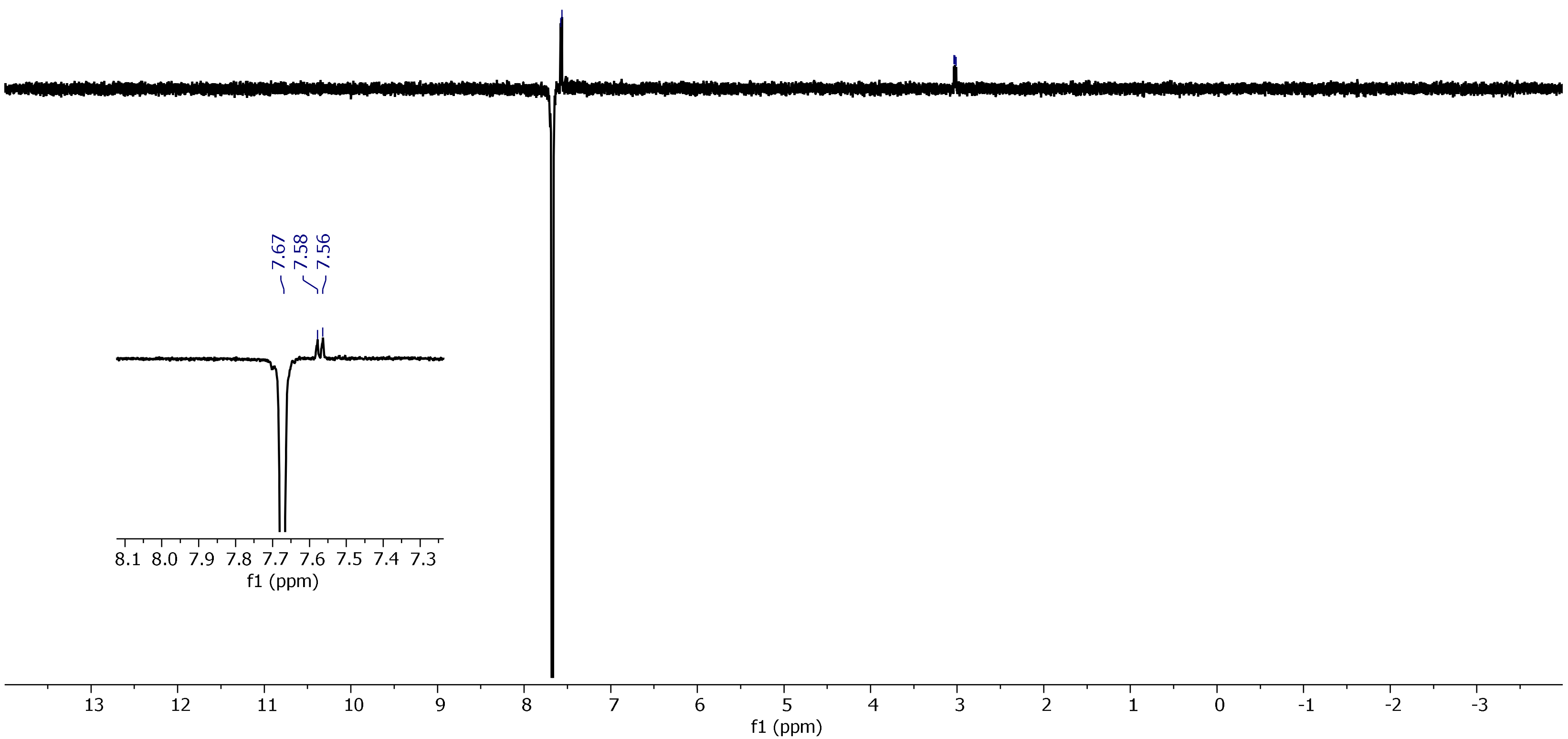

Figure S189. 1D NOE NMR ($CDCl_3$, 500 MHz) spectrum **6a**-(***E***) [irradiation of the signal at 3.02 ppm]

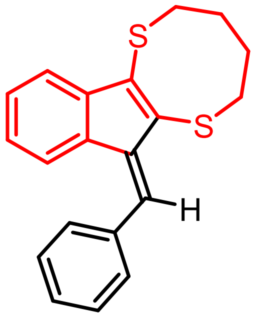

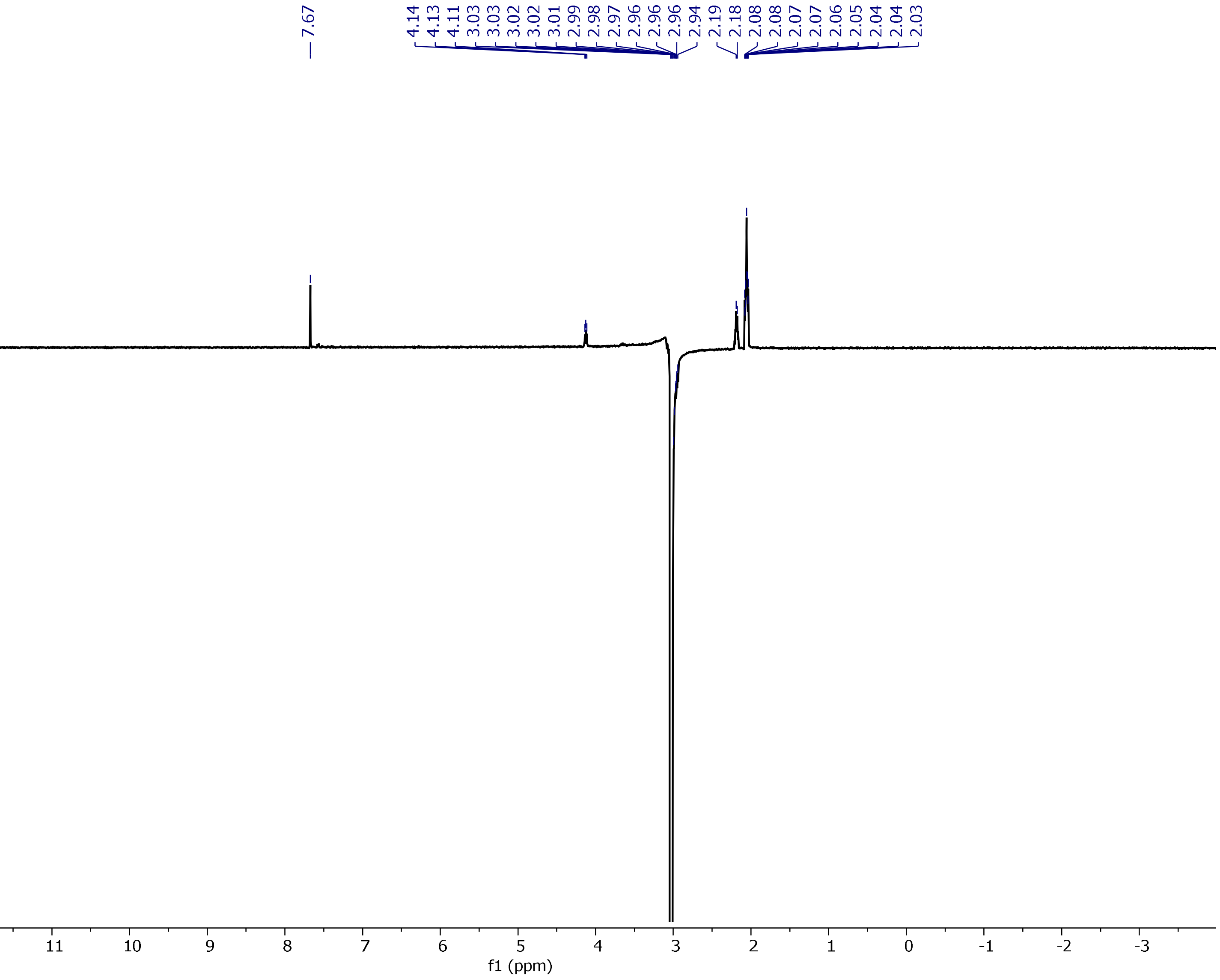

Figure S190. $^{13}C\{^1H\}$ NMR (126 MHz, $CDCl_3$, APT) spectrum **6a-(*E*)**

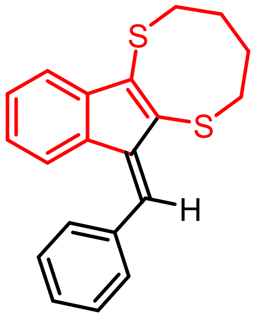

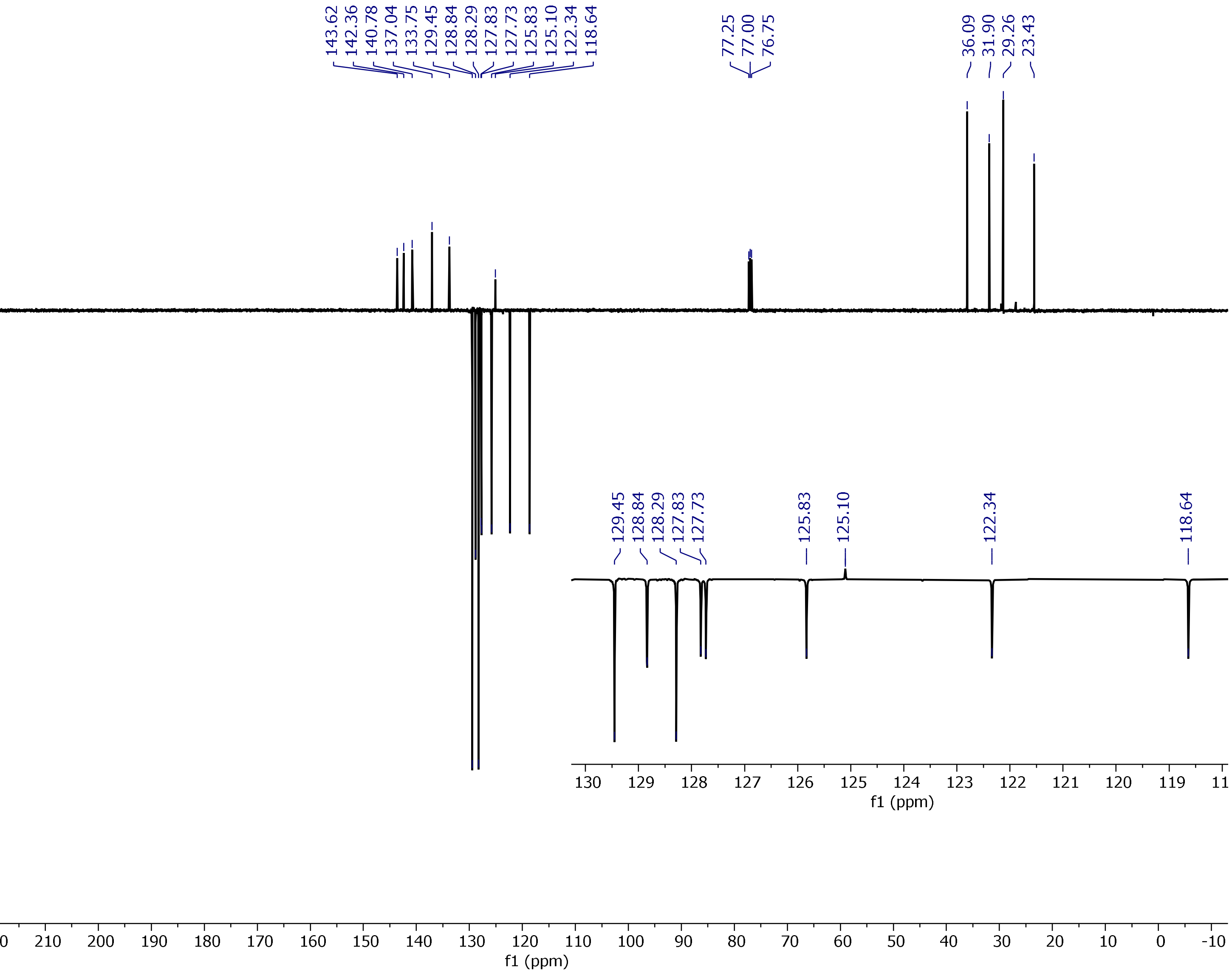

Figure S191. ^{1}H NMR ($CDCl_3$, 500 MHz) spectrum **6a**-(***Z***)

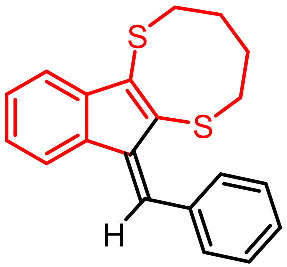

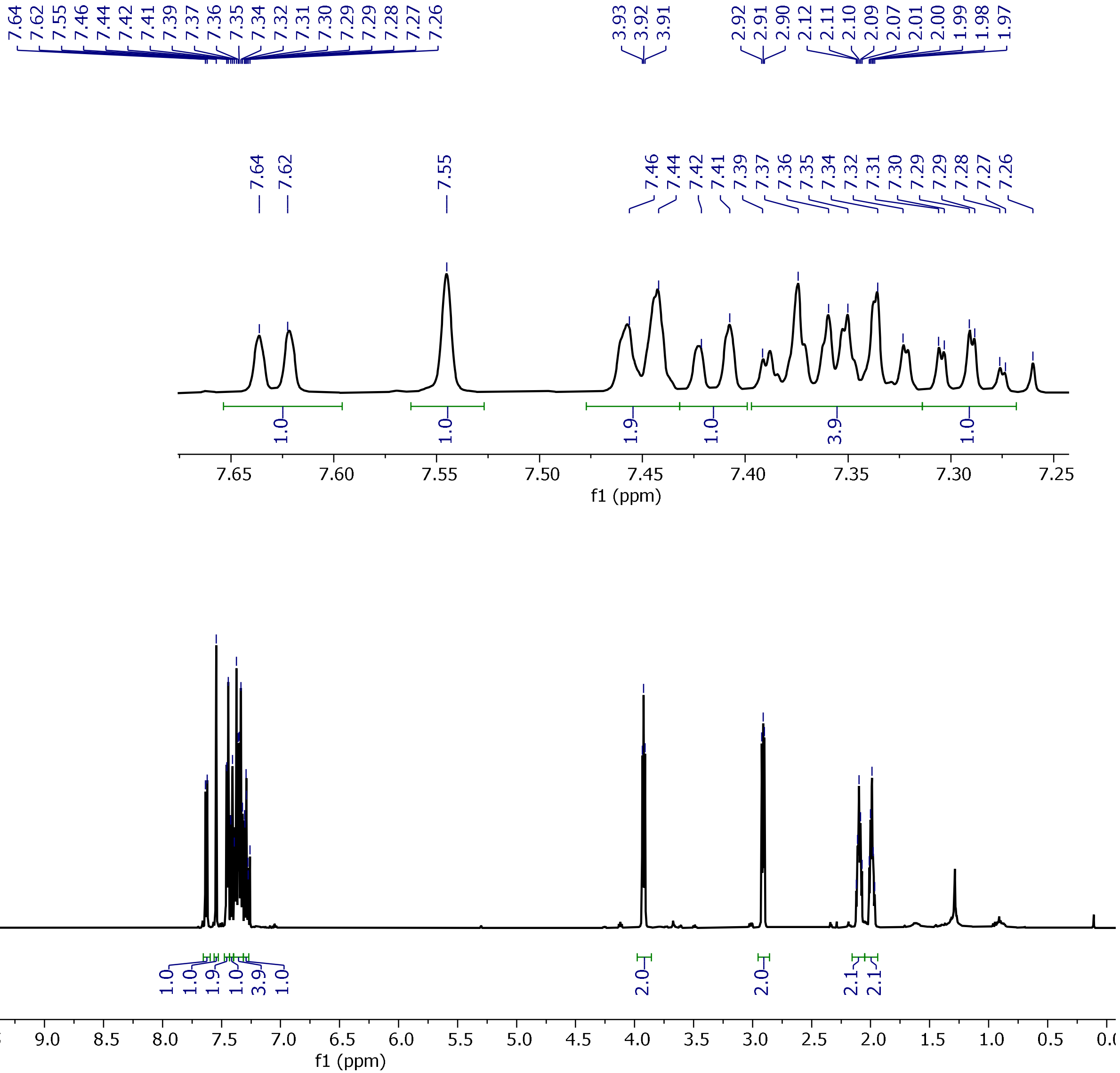

Figure S192. 1D NOE NMR ($CDCl_3$, 500 MHz) spectrum **6a**-(***Z***) [irradiation of the signal at 7.55 ppm]

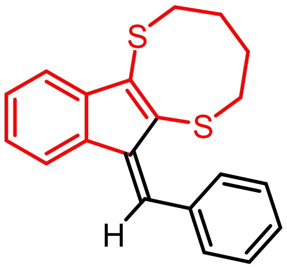

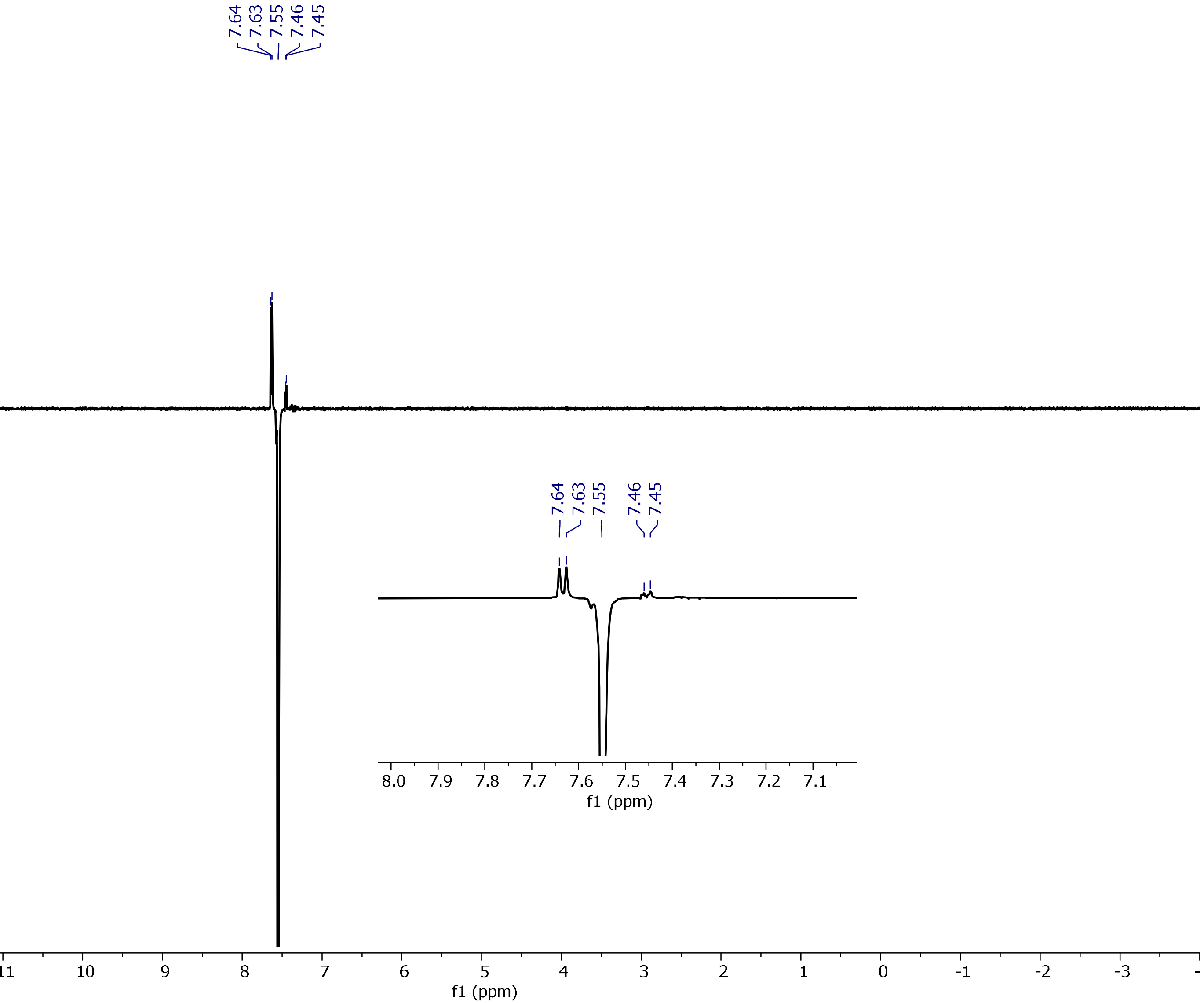

Figure S193. $^{13}C\{^{1}H\}$ NMR (126 MHz, $CDCl_3$, APT) spectrum **6a-(*Z*)**

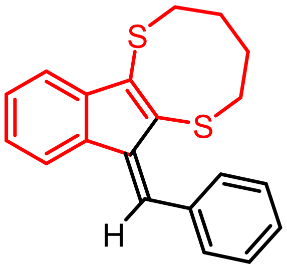

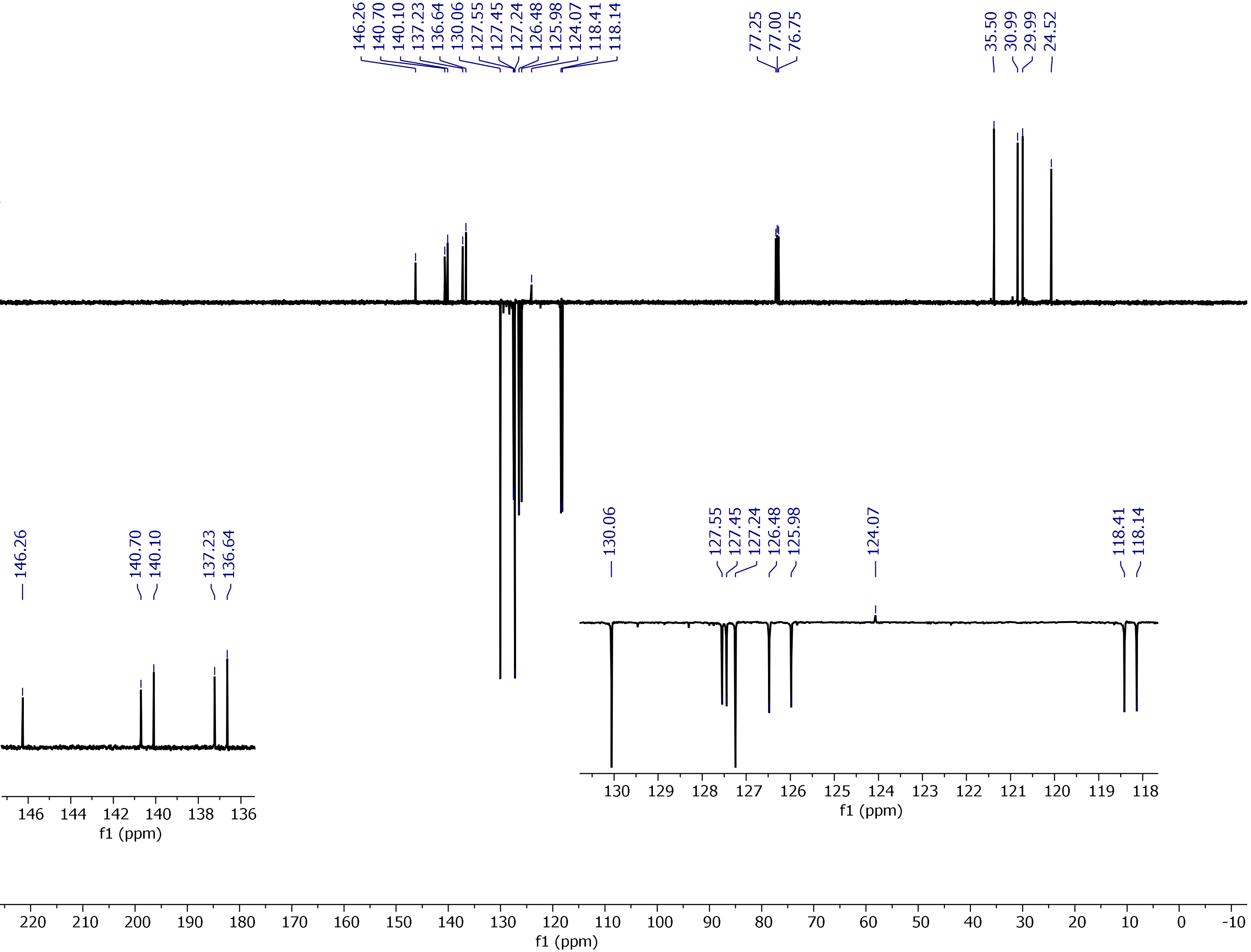

Figure S194. ^{1}H NMR ($CDCl_3$, 500 MHz) spectrum **2a'**

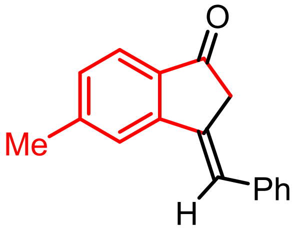

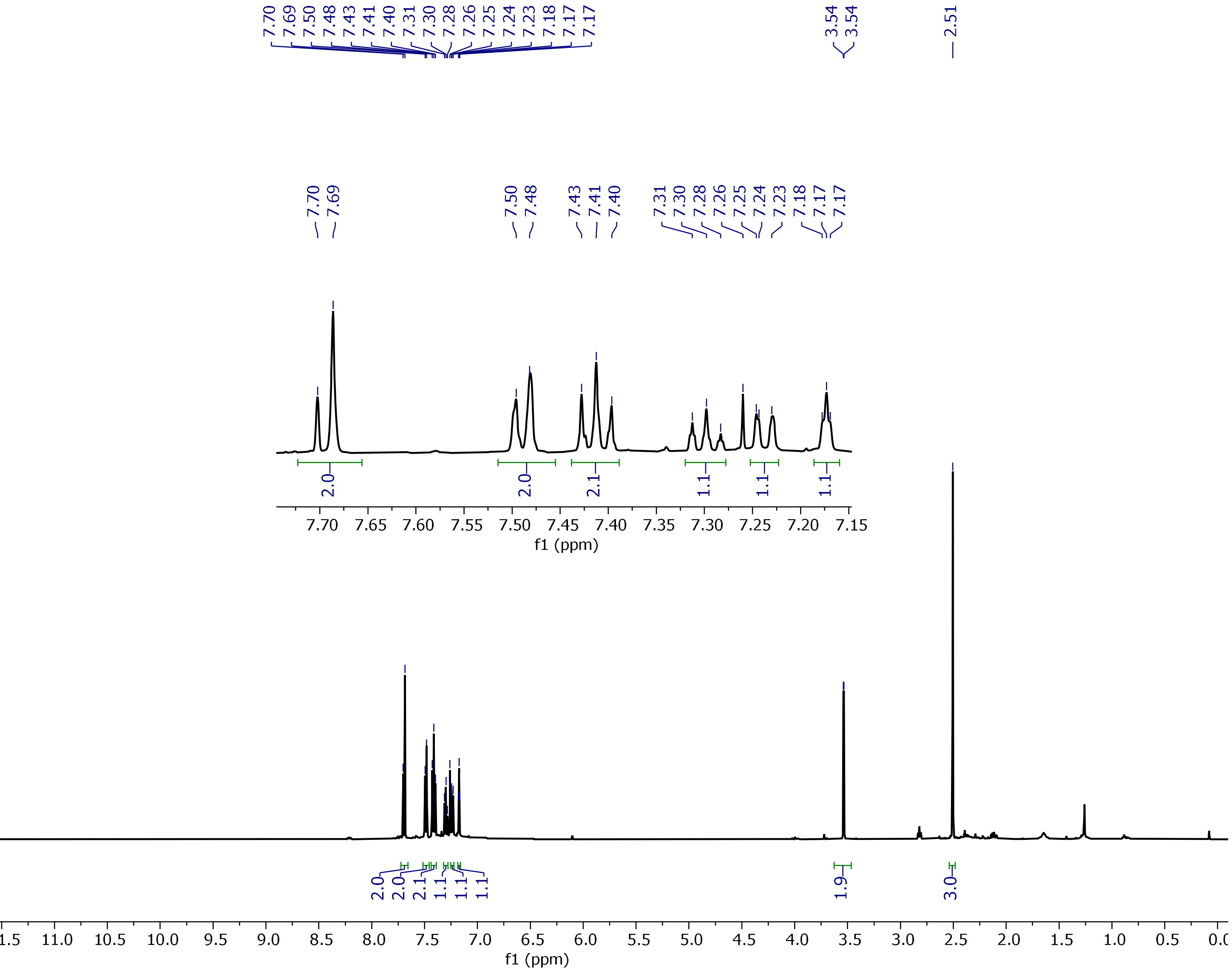

Figure S195. $^{13}C\{^1H\}$ NMR (126 MHz, $CDCl_3$, APT) spectrum **2a'**

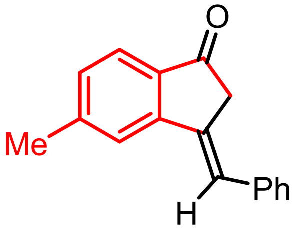

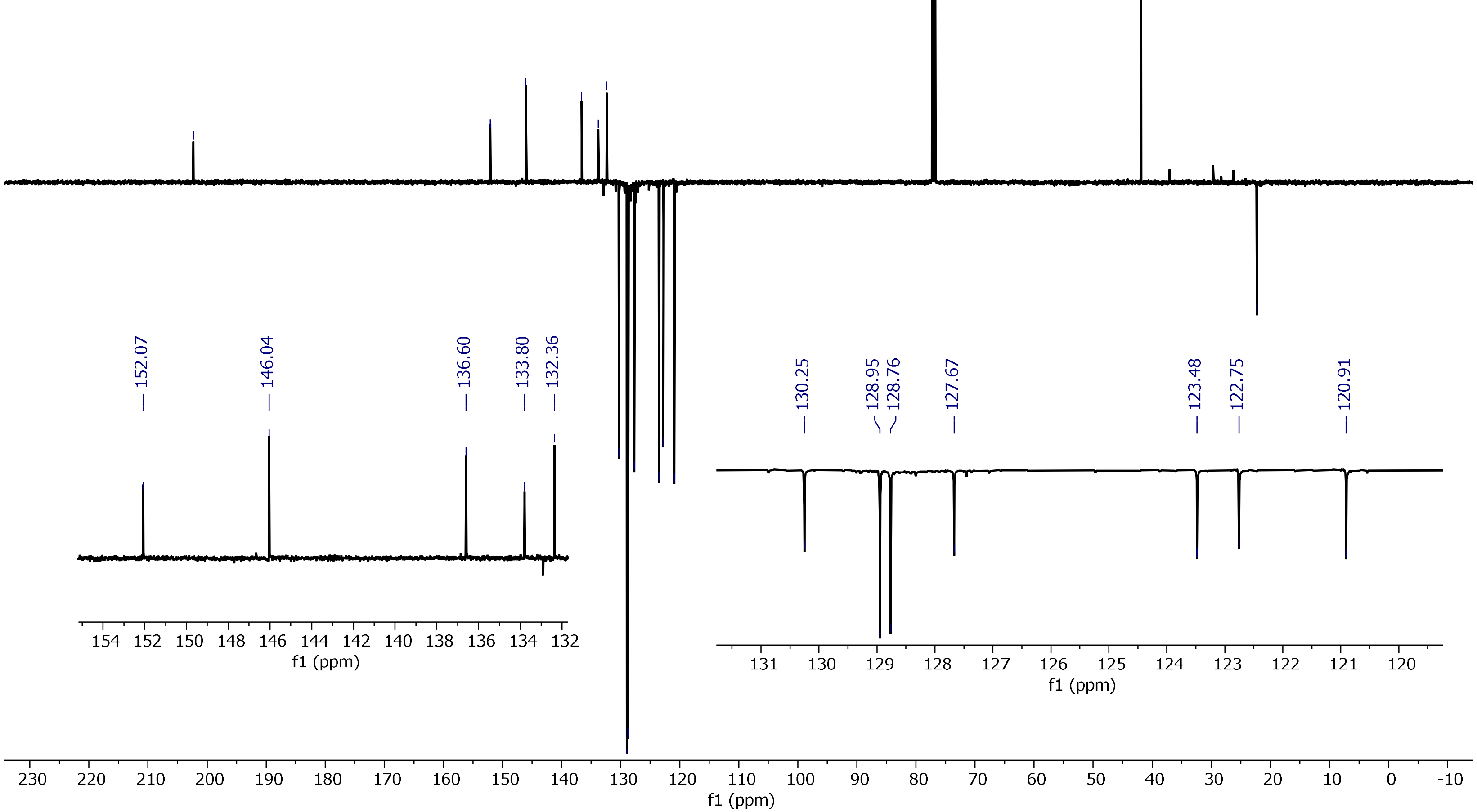

Figure S196. ^{1}H NMR ($CDCl_3$, 500 MHz) spectrum **2b'**

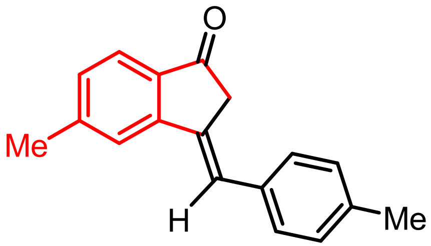

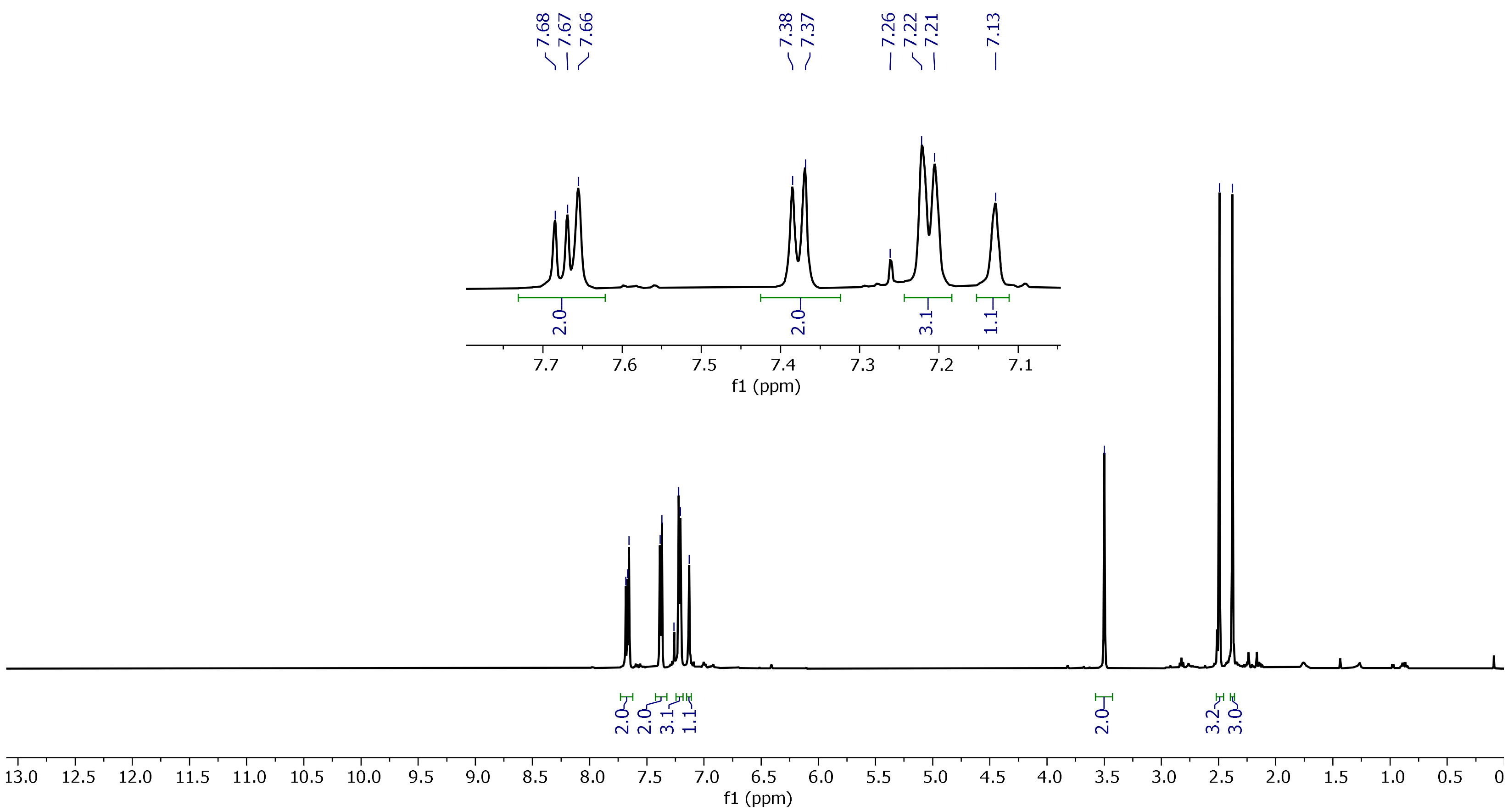

Figure S197. $^{13}C\{^{1}H\}$ NMR (126 MHz, $CDCl_3$, APT) spectrum **2b'**

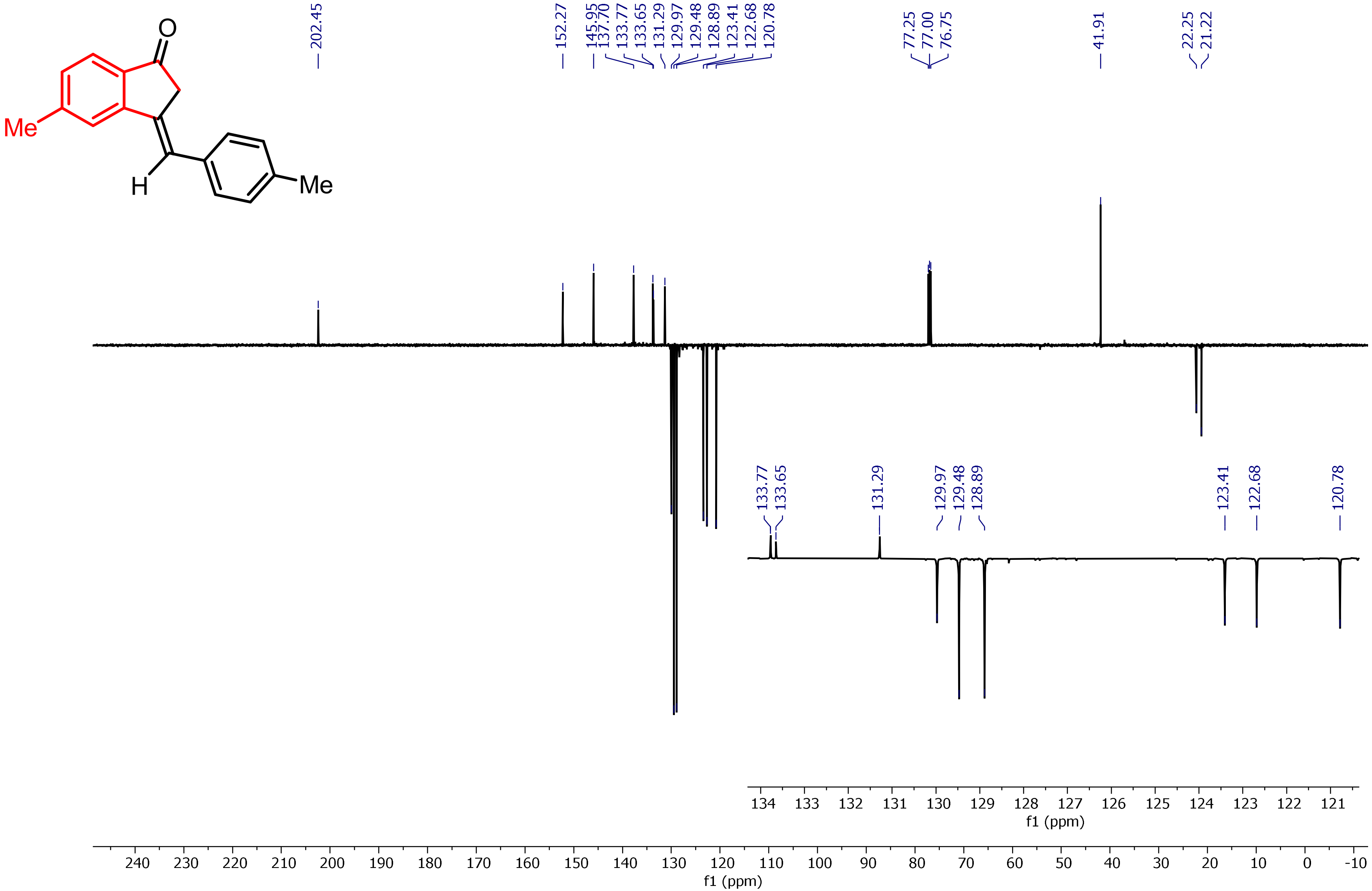

Figure S198. ^{1}H NMR ($CDCl_3$, 500 MHz) spectrum **2c'**

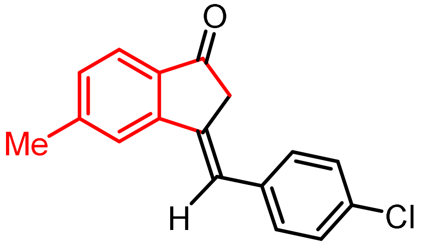

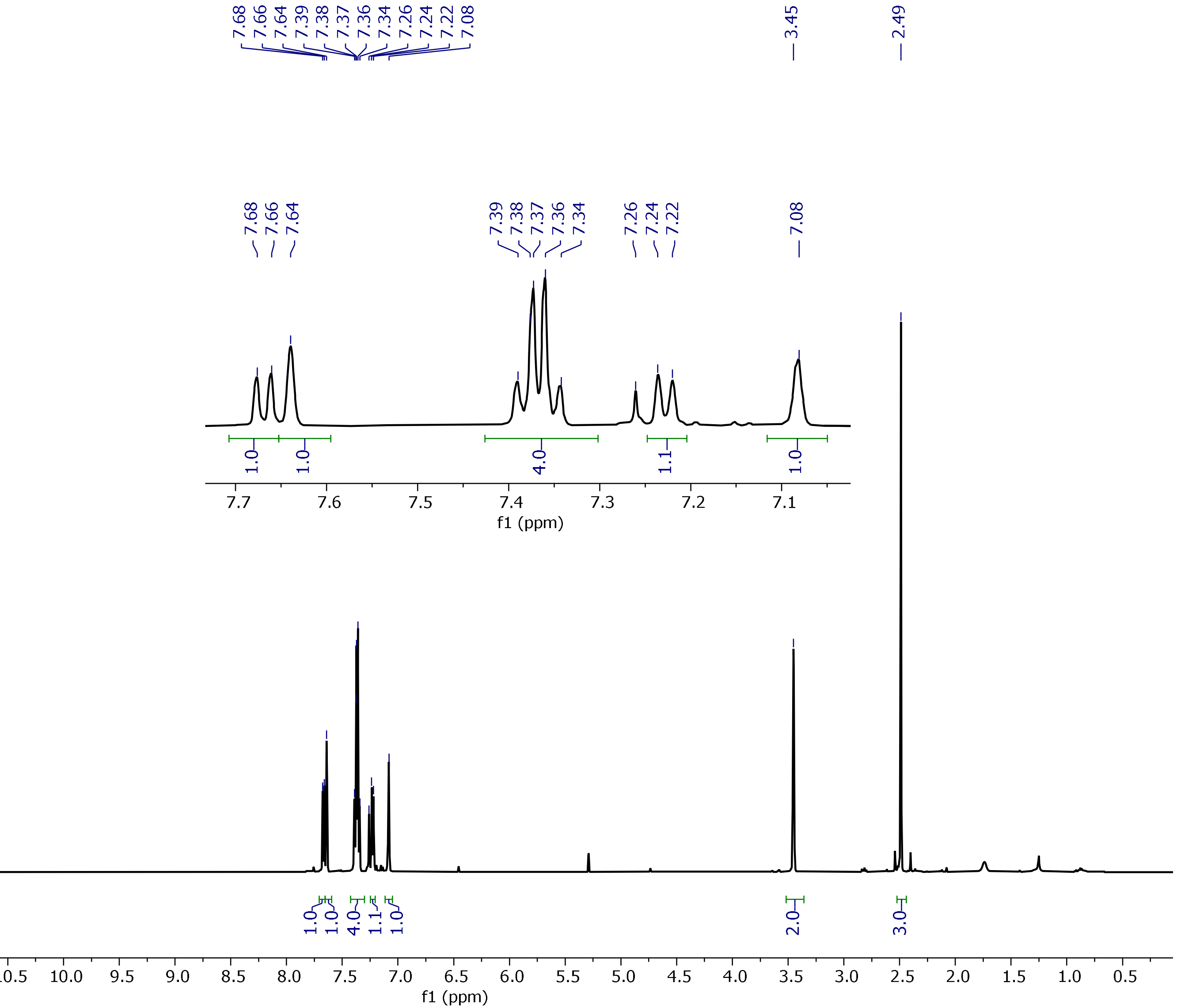

Figure S199. $^{13}C\{^1H\}$ NMR (126 MHz, $CDCl_3$, APT) spectrum **2c'**

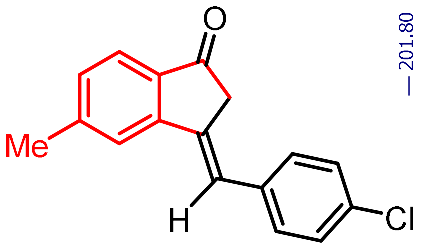

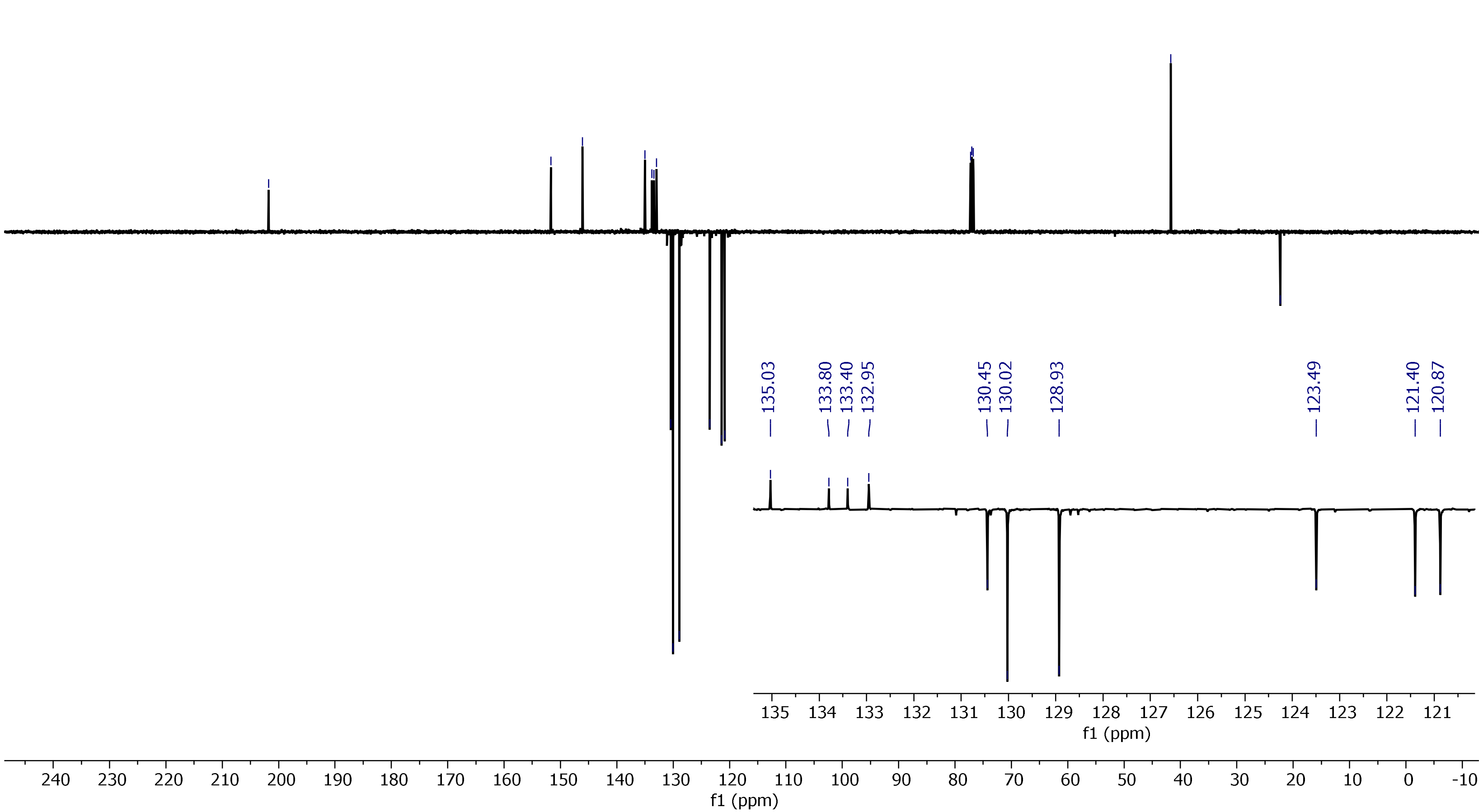

Figure S200. ^{1}H NMR ($CDCl_3$, 500 MHz) spectrum **2d'**

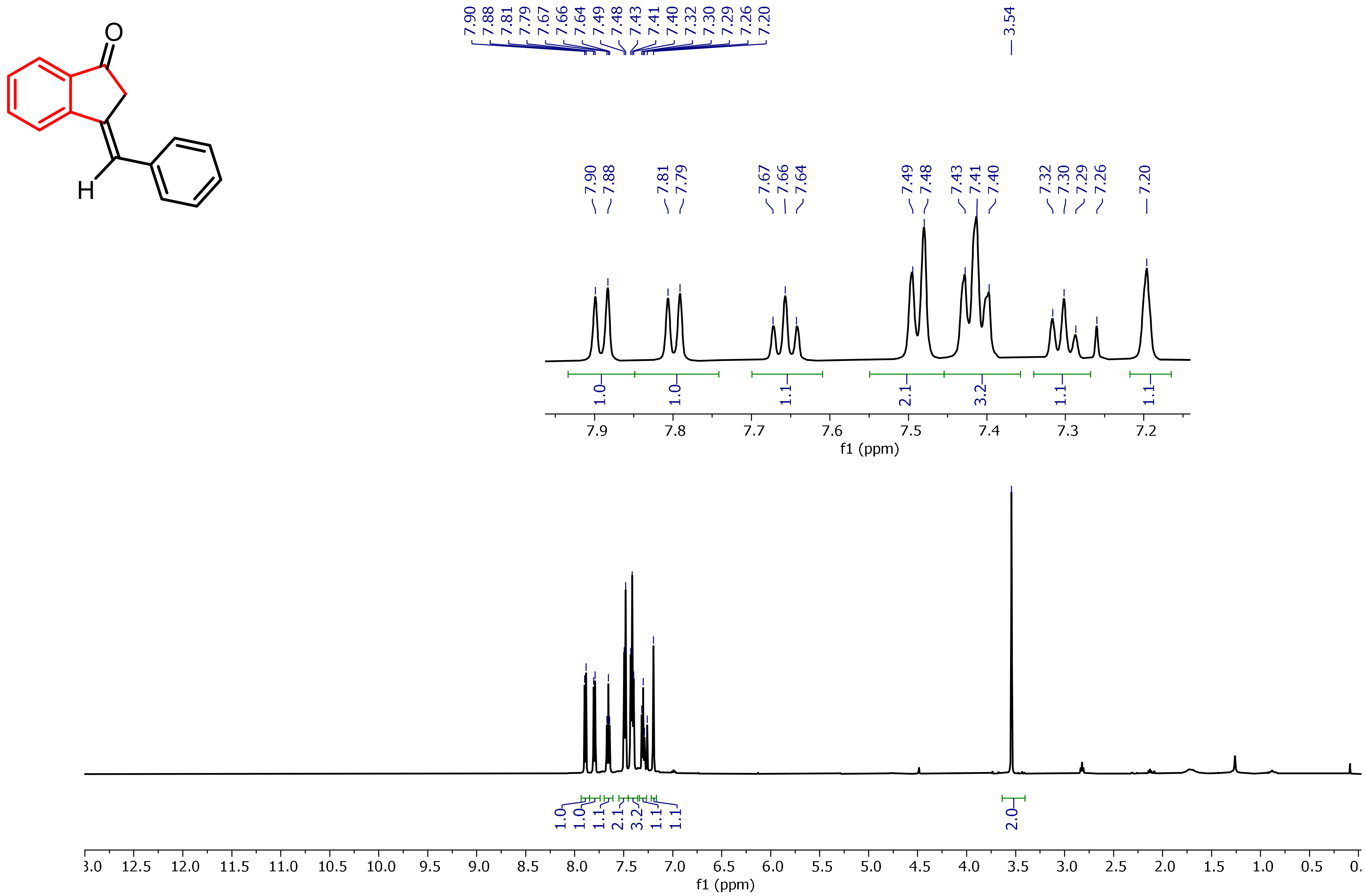

Figure S201. $^{13}C\{^{1}H\}$ NMR (126 MHz, $CDCl_3$, APT) spectrum **2d'**

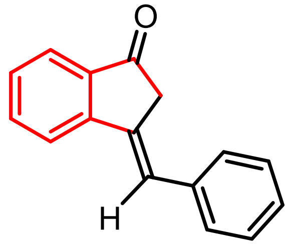

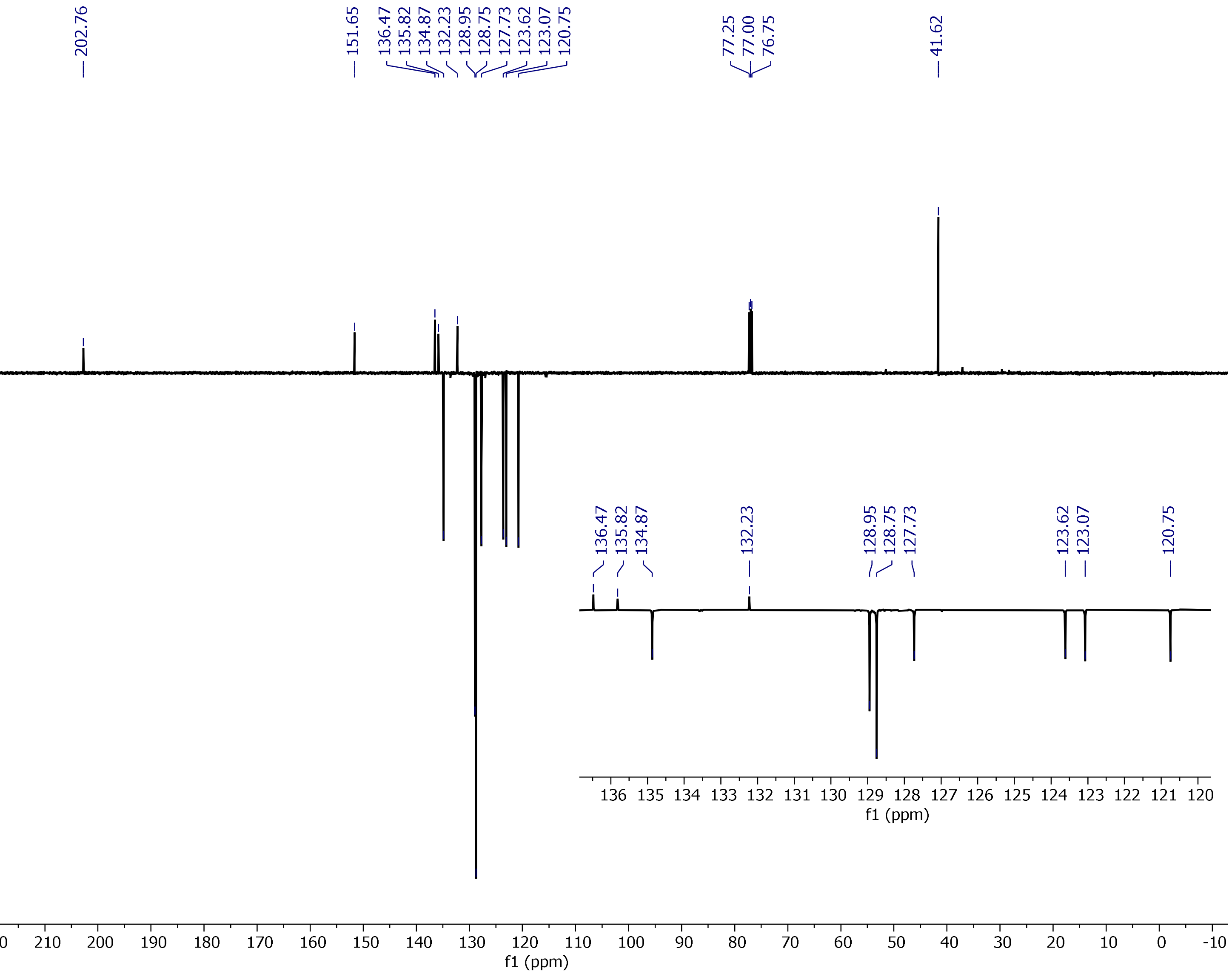

Figure S202. ^{1}H NMR ($CDCl_3$, 500 MHz) spectrum **2e'**

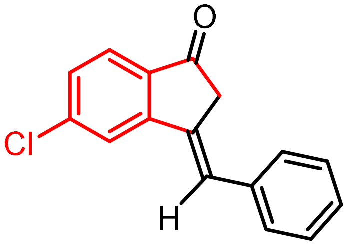

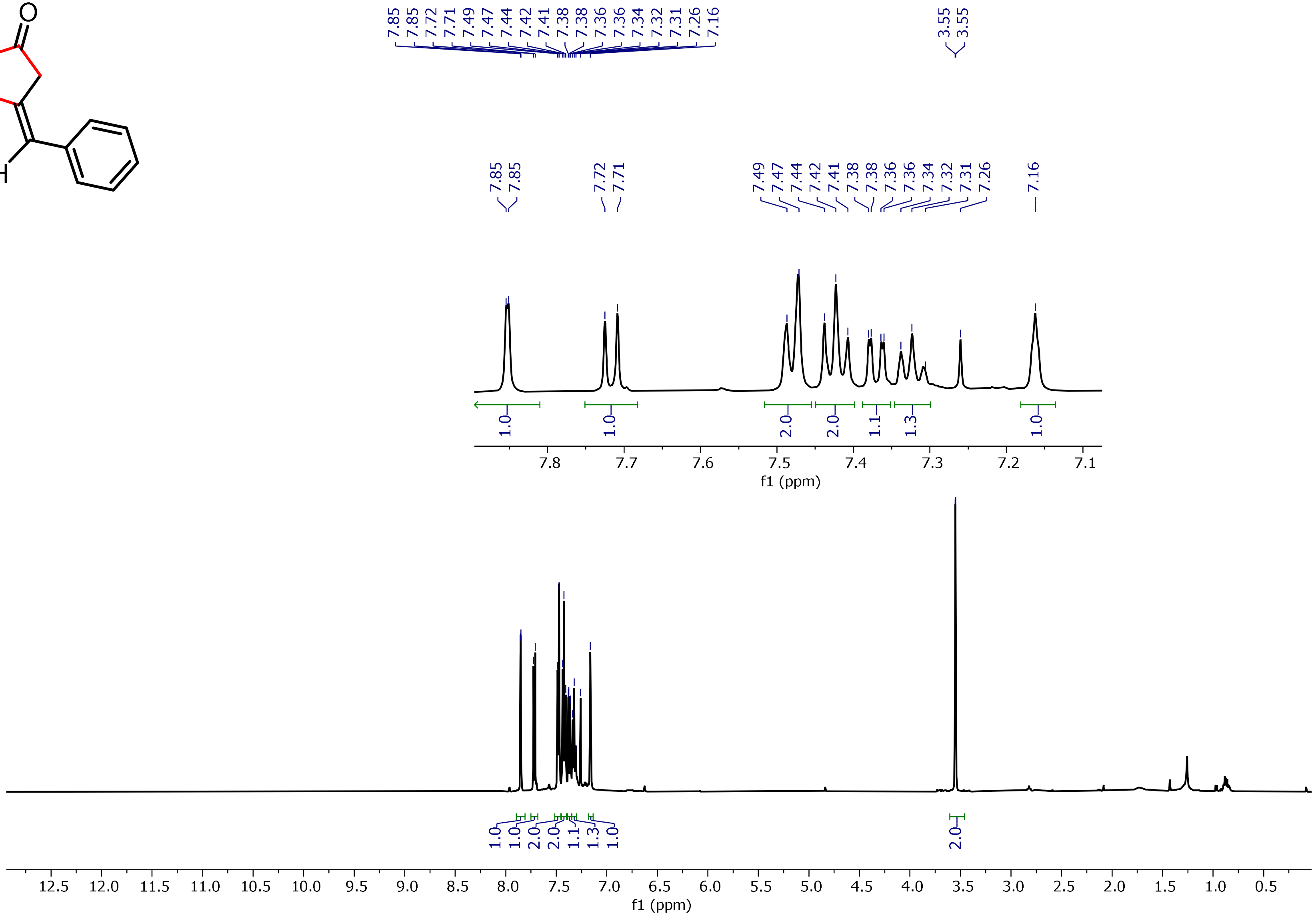

Figure S203. $^{13}C\{^{1}H\}$ NMR (126 MHz, $CDCl_3$, APT) spectrum **2e'**

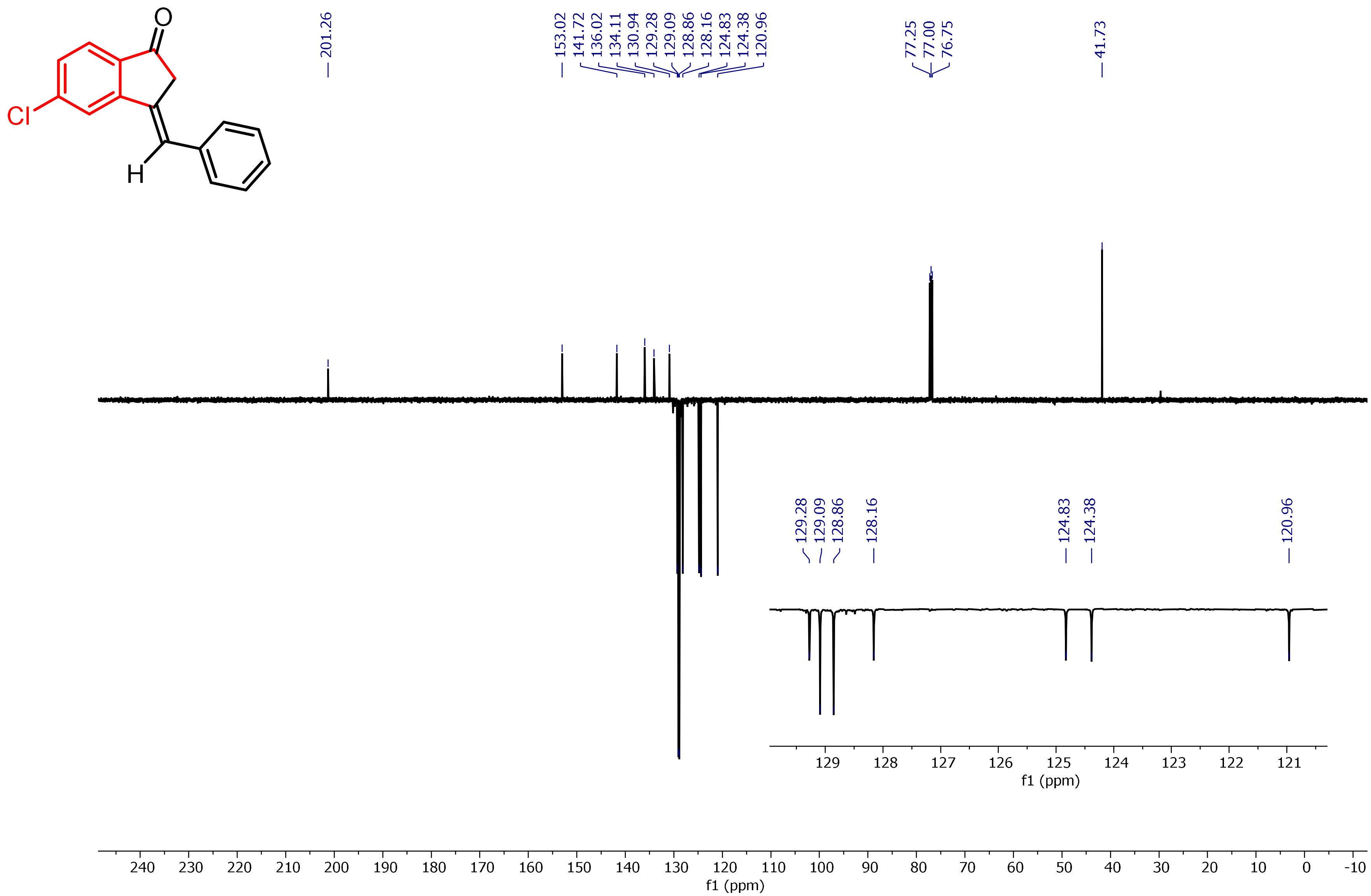

Figure S204. ^{1}H NMR ($CDCl_3$, 500 MHz) spectrum **2g'**

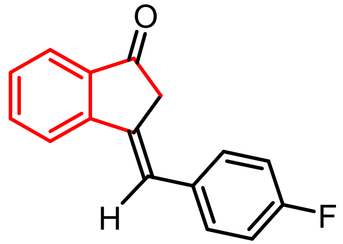

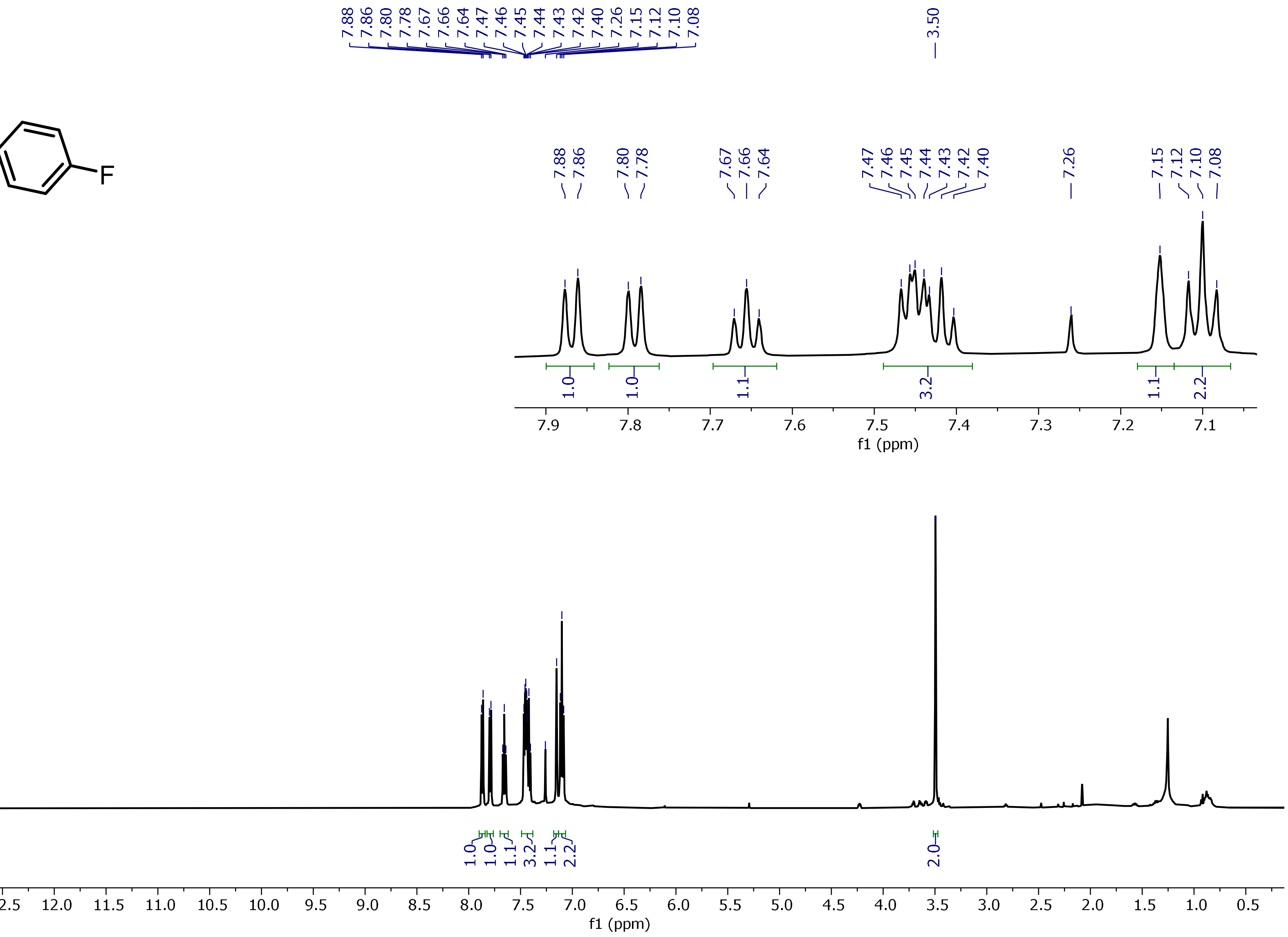

Figure S205. $^{13}C\{^{1}H\}$ NMR (126 MHz, $CDCl_3$, APT) spectrum **2g'**

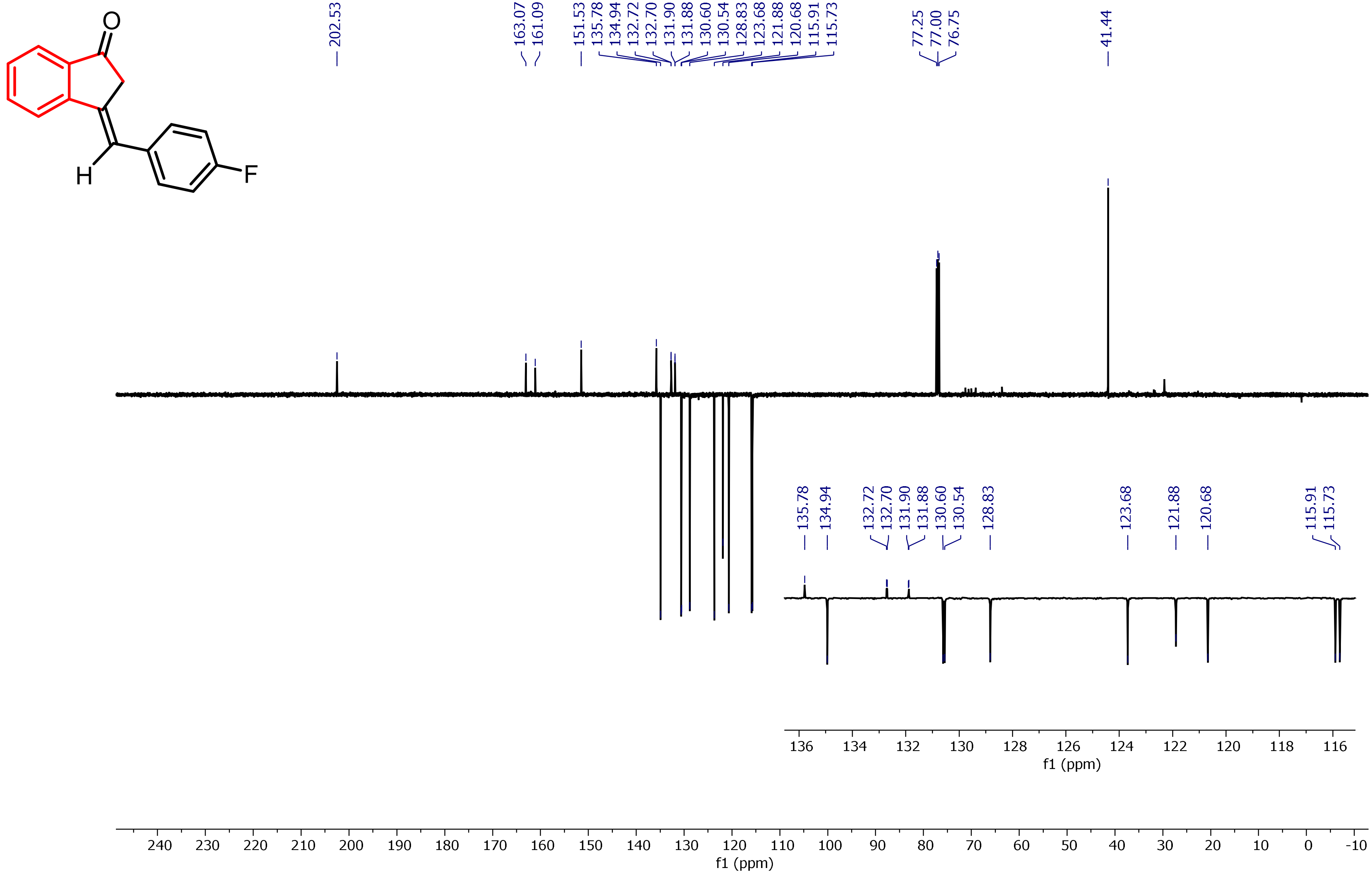

Figure S206. ^{1}H NMR ($CDCl_3$, 500 MHz) spectrum **2h'**

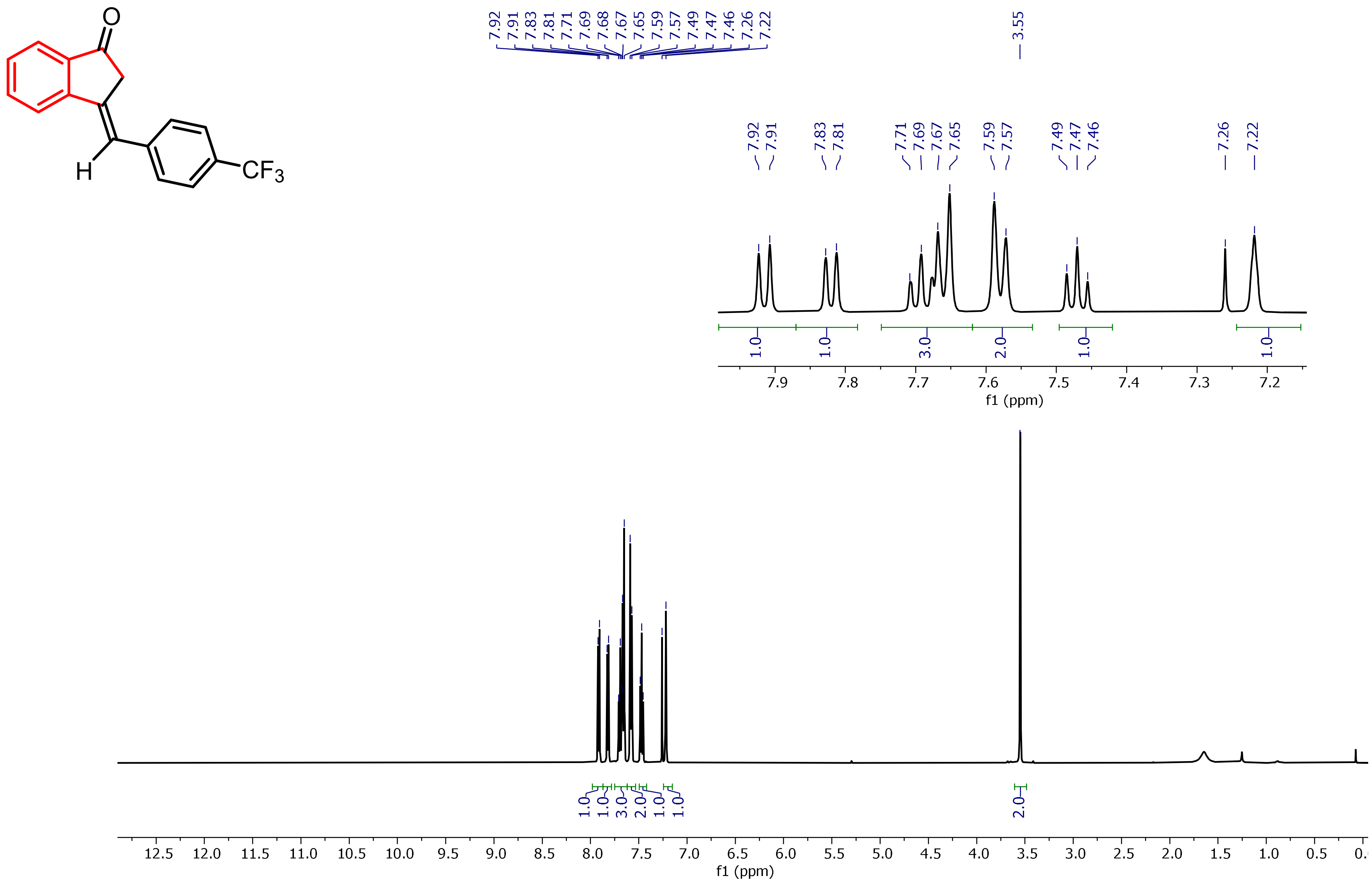

Figure S207. $^{13}C\{^{1}H\}$ NMR (126 MHz, $CDCl_3$, APT) spectrum **2h'**

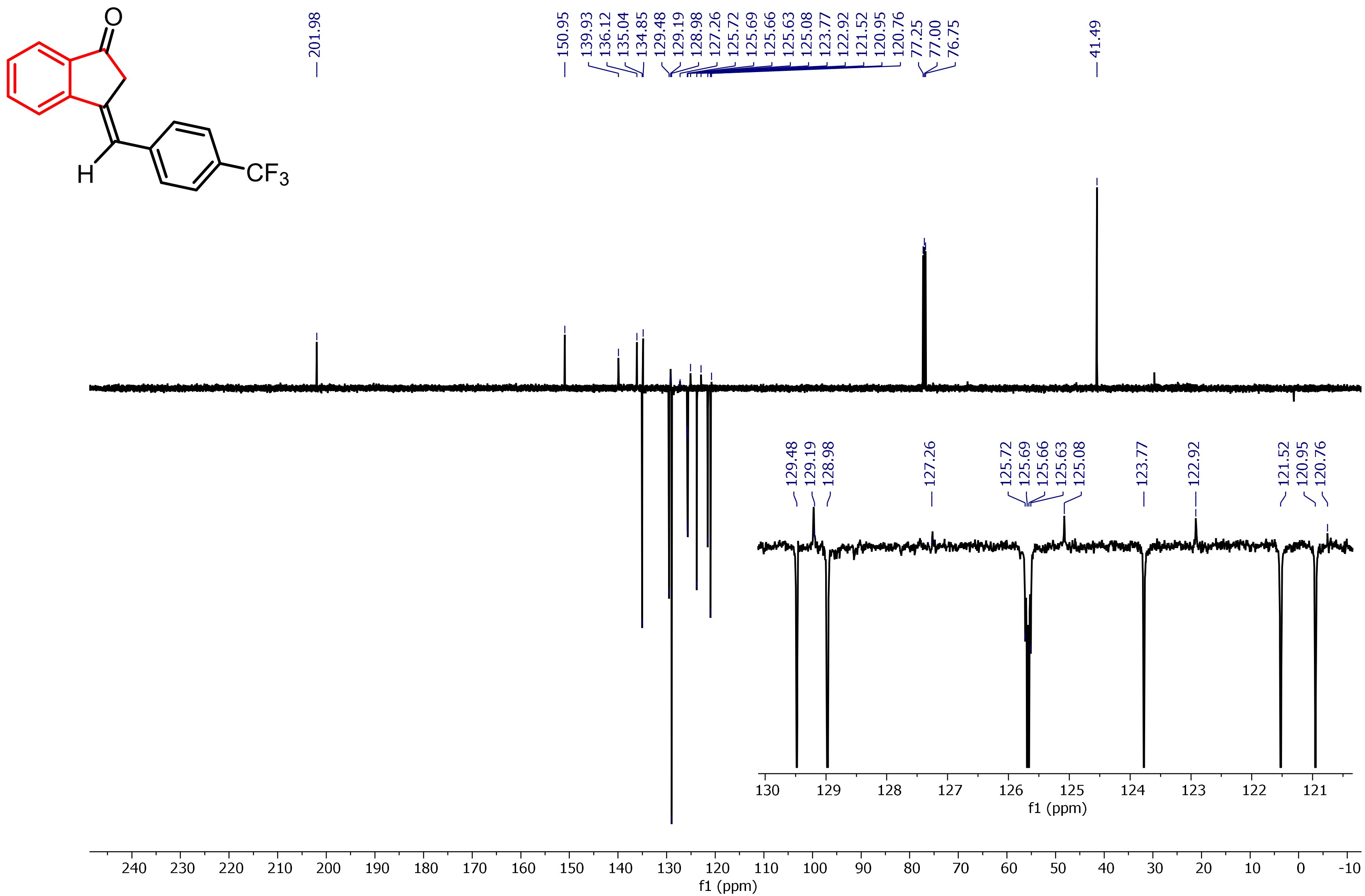

Figure S208. ^{1}H NMR ($CDCl_3$, 500 MHz) spectrum **2o'**

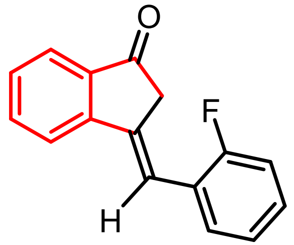

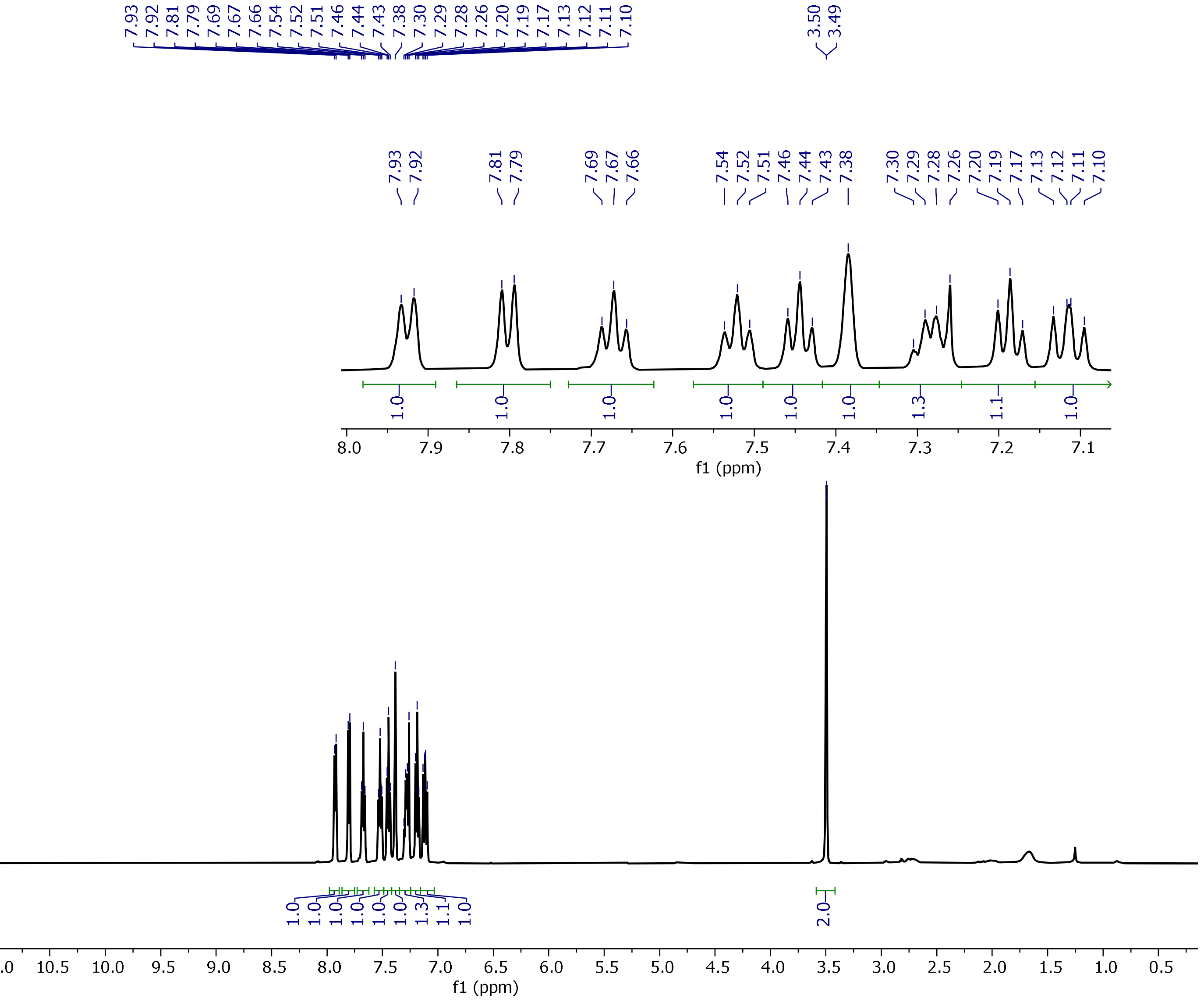

Figure S209. ${}^{13}C\{{}^{1}H\}$ NMR (126 MHz, $CDCl_3$, APT) spectrum **2o'**

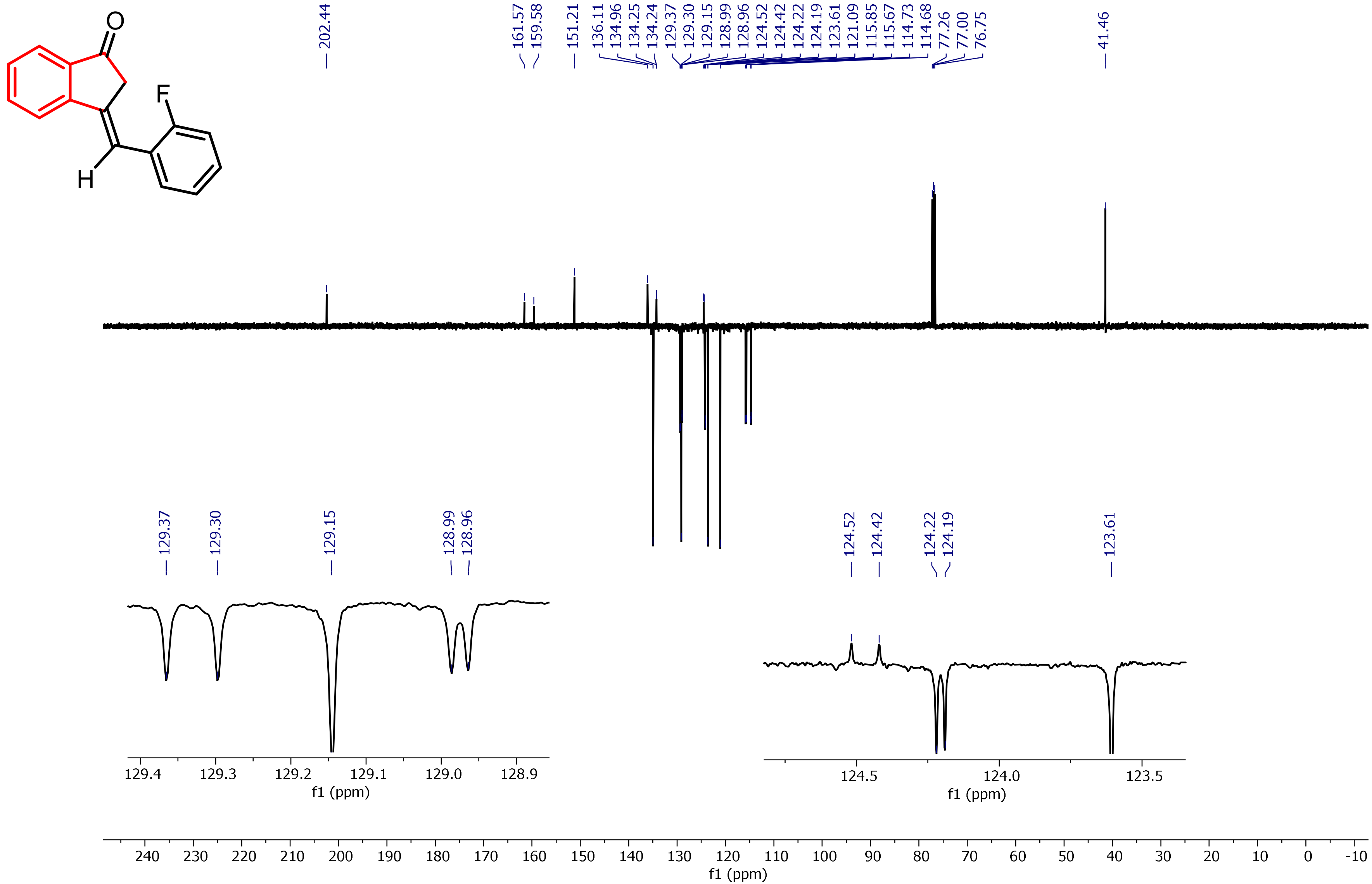

Figure S210. ^{1}H NMR ($CDCl_3$, 500 MHz) spectrum **2q'**

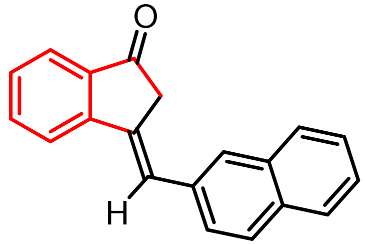

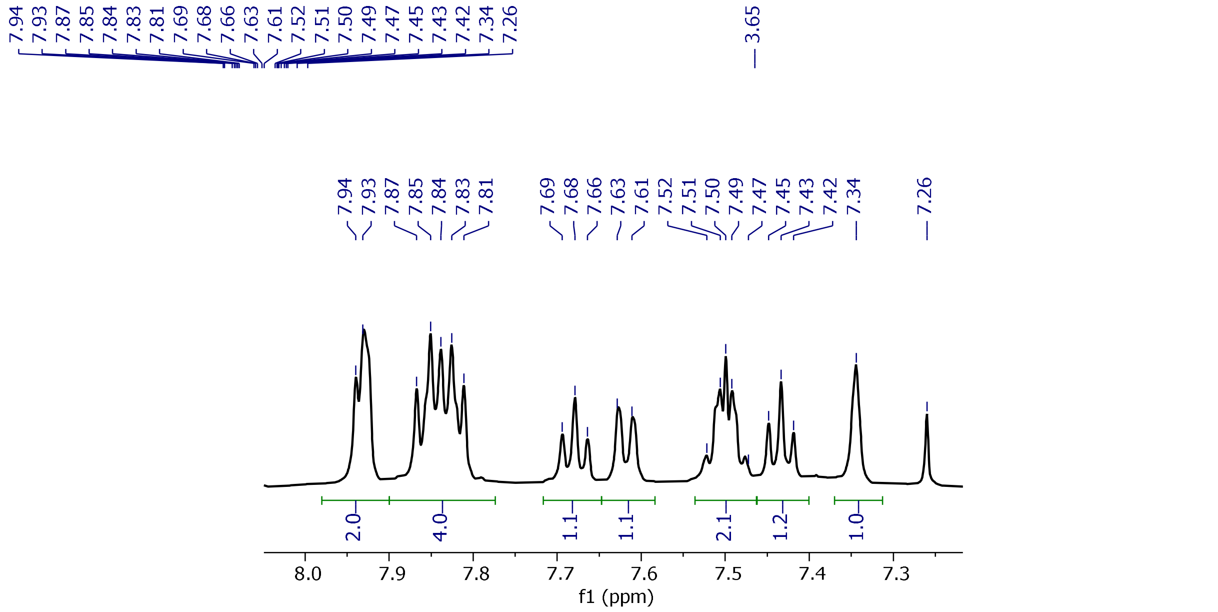

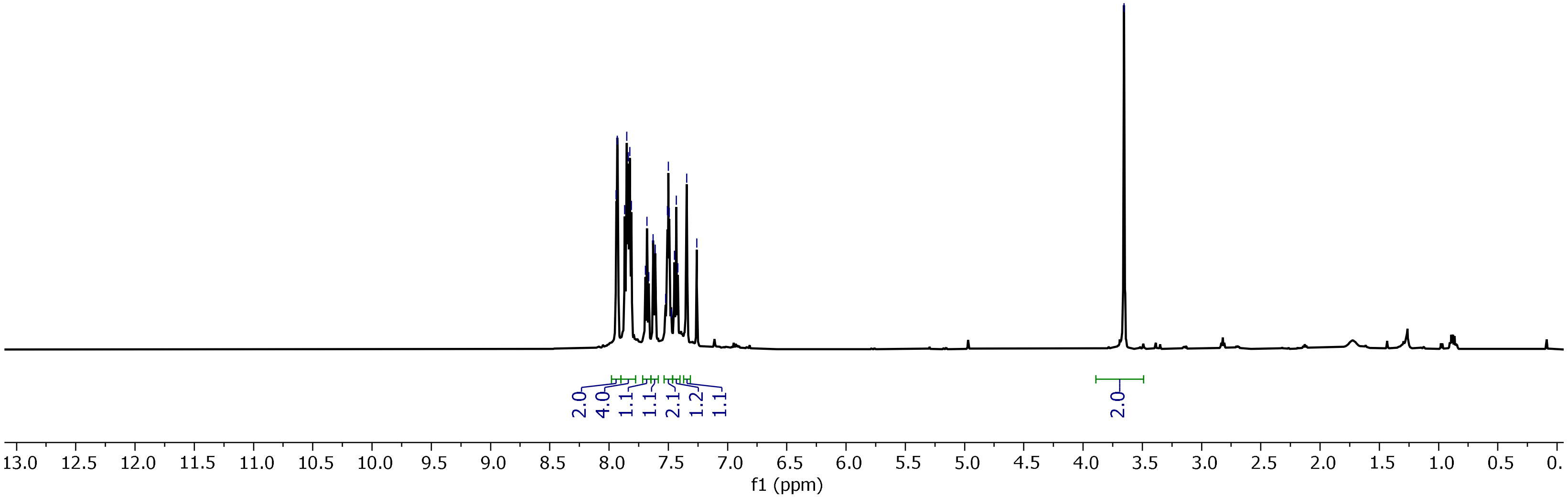

Figure S211. $^{13}C\{^1H\}$ NMR (126 MHz, $CDCl_3$, APT) spectrum **2q'**

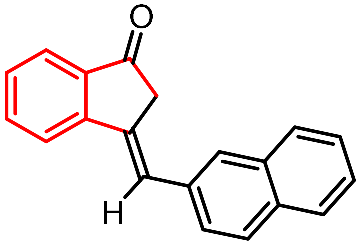

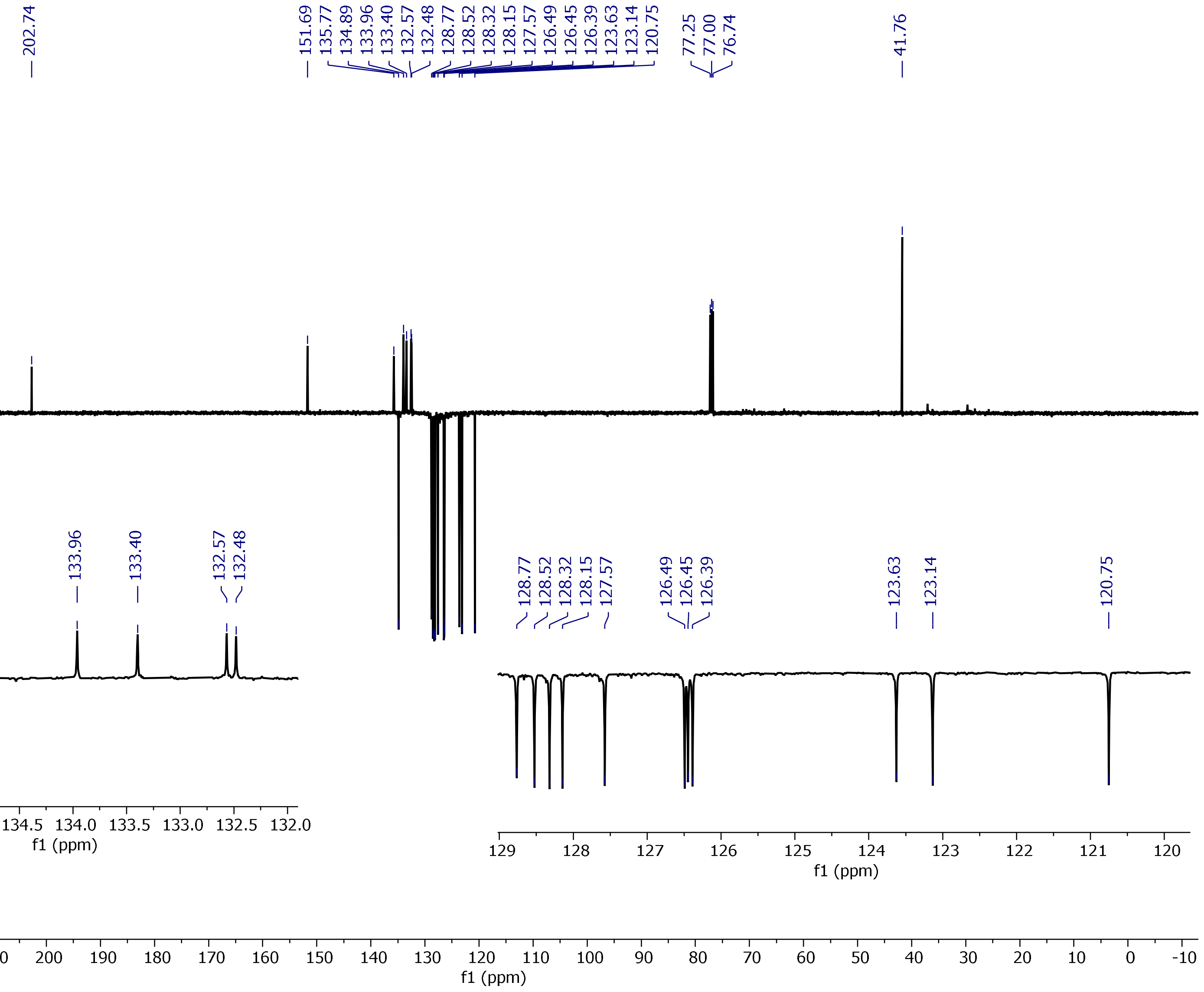

Supplement: Supplementary file 7 [file jo6c00685_si_007.pdf]
